# Supplementary material for: Climate change induced complex shifts in snake distributions expose people to snakebite and threaten biodiversity
Source: PLoS Negl Trop Dis. 2026 May 21;20(5):e0014030. doi: 10.1371/journal.pntd.0014030 (PMC13193456; doi:10.1371/journal.pntd.0014030)
Supplement: S1 Text — (DOCX) [file pntd.0014030.s001.docx]

**General documentation**

Environmental Niche Modelling and Data Processing Methodology

September 2024

Contact points:

Dr Anna F.V. Pintor, [PintorA@WHO.int](mailto:PintorA@WHO.int)

Table of Contents

[Study Species 3](#_Toc176884688)

[Occurrence Data 4](#_Toc176884689)

[Data sourcing 4](#_Toc176884690)

[Data cleaning 4](#_Toc176884691)

[Expert Derived Ranges 5](#_Toc176884692)

[**Fig A.** Examples of occurrence records and expert derived ranges 6](#_Toc176884693)

[Environmental Data 7](#_Toc176884694)

[Climate (10): 7](#_Toc176884695)

[Topography (2): 8](#_Toc176884696)

[**Fig B**. Examples of environmental Input variables 8](#_Toc176884697)

[Vegetation (4): 8](#_Toc176884698)

[Land Use (1): 9](#_Toc176884699)

[Soil (5): 9](#_Toc176884700)

[Water (1): 9](#_Toc176884701)

[Human Influence (1): 9](#_Toc176884702)

[Future Climate (10 x 7) 9](#_Toc176884703)

[Modelling Methods 10](#_Toc176884704)

[Model Preparation & Parameters 10](#_Toc176884705)

[**Fig C**. Examples of model background and occurrence records 11](#_Toc176884706)

[Variable Selection 12](#_Toc176884707)

[Final Model Processing 12](#_Toc176884708)

[Thresholding 12](#_Toc176884709)

[Cost Distance Calculations 13](#_Toc176884710)

[**Fig D**. Example of model output processing 14](#_Toc176884711)

[Summary Outputs 15](#_Toc176884712)

[Climate Change Summaries 16](#_Toc176884713)

[Statistics 16](#_Toc176884714)

[References 18](#_Toc176884715)

# Study Species

We used the existing list of WHO recognized medically relevant snake species and revised the taxonomy of all listings using published literature, recognition status by the Reptile Database^1^, and expert feedback. We included any currently recognized species that were either (i) explicitly listed^2,3^, (ii) implicitly listed by being part of a newly split up listed species complex, or (iii) part of a species complex that is undergoing frequent revisions and includes a listed species (e.g. we included all members of the *Atractaspis microlepidota* species complex even though only *A. fallax* was explicitly listed because of frequent revisions and because members of the complex are sometimes considered synonyms of each other, i.e. the taxonomy is poorly resolved). Wherever species status of taxa was equivocal based on discussions amongst our expert panel, we opted to maintain higher level lineages as one species rather than elevate subspecies to species status to avoid inflation of species numbers for which potentially different antivenoms are needed. The final list included 508 species. We combined these into 281 suggested new Listing Units (LUs), which combine any closely related species that are currently covered by the same antivenoms and have poorly resolved taxonomic and/or geographic delineation and should, in our opinion, be listed together for ease of antivenom labelling and production and to allow for ongoing revisions without affecting antivenom recommendations. These groups often also include restricted taxa that would not qualify for listing on their own based on the requirement for listed species to affect a large number of people, but that in combination with their sister species present taxa that cover a large range, affect large groups of people, and can often be treated with the same antivenom. Their inclusion avoids progressively ‘losing’ treatment options for relevant species due to taxonomic splitting into species with smaller and smaller geographic ranges. Examples of LUs are the *Trimeresurus albolabris* complex and some other *Trimeresurus* complexes with ongoing revisions, the *Atheris squamigera* complex, which includes several newly described species, or the *Bungarus candidus* complex (*Bungarus candidus*, *B. multicinctus*, *B. suzhenae*, *Bungarus wanghaotingi*, and potentially other cryptic species; Anita Malhotra, pers. com.). A complete list of all species is included in **Supp. Mat. 2**.

# Occurrence Data

## Data sourcing

Occurrence records were collated from public, private, and citizen science databases (e.g. GBIF^4^, VertNet^5^, ALA^6^, iDigBio^7^, Arctos^8^, iNaturalist^9^, HerpMapper^10^, The Snake Atlas for Namibia^11^, Thai National Parks^12^, Reptile Atlas of Africa^13^, Observations.org^14^, Herpetology of Ethiopia and Eritrea^15^, India Biodiversity Portal^16^, and Mark O’Shea’s Snakes on Islands^17^, Kenyan Reptile Atlas^18^), museum records, books^19-21^, and a broad array of scientific literature (references provided in data tables). The data were furthermore supplemented with personal observations from experts and the general public (e.g. personal communications with researchers, photos on facebook, etc.) for data-sparse regions & taxa. Descriptive localities were georeferenced, and their spatial uncertainty estimated using Google Maps^22^ (**Sup. Mat. 3**). A short coming of many previous studies is the data sparse nature of many species. Often data sparse species are excluded from analysis despite their medical relevance. The intensive data mining process in this study aimed to overcome this issue and we did not exclude any species in our analyses.

## Data cleaning

All occurrence records went through a repeated vetting process by an expert panel of >30 experts (from here on referred to as the ‘expert panel’) from around the world and any obvious outliers (e.g. records from wrong continents), dubious records (e.g. records far outside known range without supporting evidence) and records with location accuracy only at country or region level (e.g. records arbitrarily placed near the center of a country) were removed. If experts expressed doubt about any record’s validity, it was excluded unless we could find reliable evidence for it to be a true observation (e.g. a clearly verifiable museum specimen or photos existed). The taxonomy of the records was revised, if necessary, based on the available literature and advice from the expert panel (e.g. *Naja melanoleuca* records were split up into new taxa *N. melanoleuca sensu stricto*, *N. subfulva*, *N. savannula*, *N. peroescobari*, and *N. guineensis* based on record location, where definitive, or on evidence such as photos, which confirmed taxon identity). For most species, records for each species were further reduced to 75% of records with the highest location accuracy. However, for any species that displayed obvious geographic bias in record accuracy (e.g. all records from one country or region would have been excluded) or that would have been reduced to 20 or less unique recorded locations if using the 75% rule deficient (< 20 records; the number of records below which model accuracy increases most rapidly^23^), we used all available records. For some species, obvious spatial bias in data was reduced by only filtering out low accuracy data in densely sampled areas while keeping all records in sparsely sample areas (e.g. some species were extremely data rich in Taiwan but data poor in mainland China so only the Taiwan data was filtered by location accuracy). Intensive data mining was conducted for any data poor species to push them over our minimum ‘standard’ of 20 unique records for modelling. Where this was not possible, we combined several closely related & ecologically similar species into ‘modelling units’ (MUs), for which a multi-species model was run. Similarly, any closely related & ecologically similar species with ill-defined or controversial taxonomic and/or geographic separations were also modelled as MUs (**Sup. Mat. 2**). Note that the 281 LUs combine species in a way that minimizes the necessity for frequent revisions of official WHO listings and, consequently, antivenom labelling. The 314 MUs on the other hand combine species only when necessary for modelling purposes, i.e. if they are data deficient or too difficult to delineate from each other due to insufficient taxonomic certainty in many areas. Only unique occurrence locations per 0.01 x 0.01 decimal degree grid cell were used in models.

# Expert Derived Ranges

Range estimates for all WHO listed snake species were obtained from the original WHO database and aligned with current WHO accepted country boundaries on ArcGIS Pro to create country specific polygons attributed with the correct risk category (1 or 2) for each snake in each country where it is present. The cleaned high confidence occurrence records described above were overlayed in ArcGIS Pro and each species’ polygons were adjusted to incorporate all areas with verified (i.e. expert vetted) occurrence records outside the previous range estimate (**Fig A**). The expert panel was invited to suggest any additional alterations to the polygons based on their knowledge of the species’ distributions (e.g. known absences or anecdotal knowledge of presence in areas without records) and habitat requirements (e.g. presence or absence in certain vegetation types, climates, or elevations). For example, some areas in South Sudan were deemed to be occupied by some species that are present to the East and West in similar habitat despite sparse records in the country, because record sparsity is likely due to the political situation in the region rather than true species’ absence. On the other hand, certain areas in Uganda were deemed to be unoccupied by *Dendroaspis polylepis* according to several experts, despite unknown biological reasons and close proximity to known occurrences.

Note that the resulting distribution polygons reflect the most likely estimates of species’ presence and absence based on current best knowledge, and that these estimates will likely change as more information becomes available. The benefit of the iterative ingoing nature of our data collection is that we can adapt to such changes in subsequent versions of the database because expert derived range polygons will be continuously updated, and new versions of the dataset published in the WHO snakebite information portal.

Distribution polygons were created for each species in each country and aligned with country borders according to vector layers provided by the WHO GIS centre for health. These same layers were also used for any maps presented here.


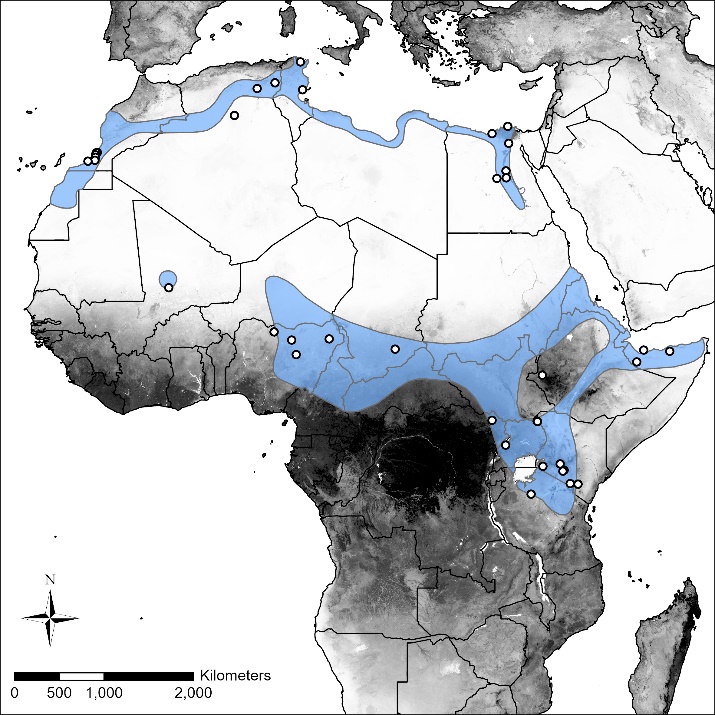

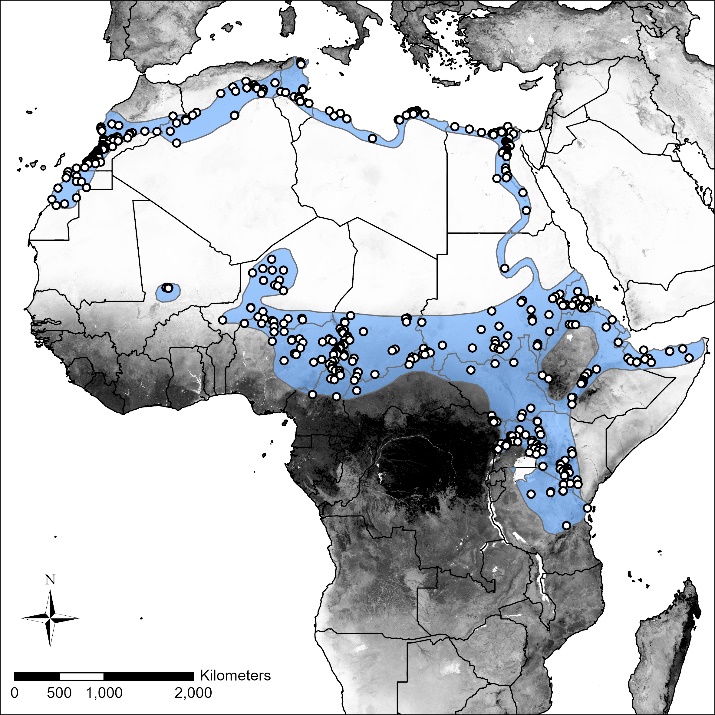

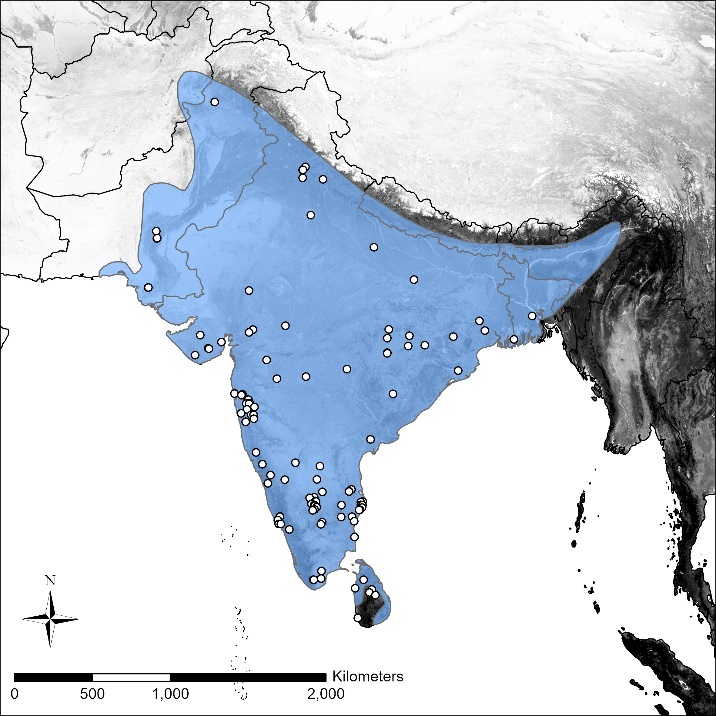

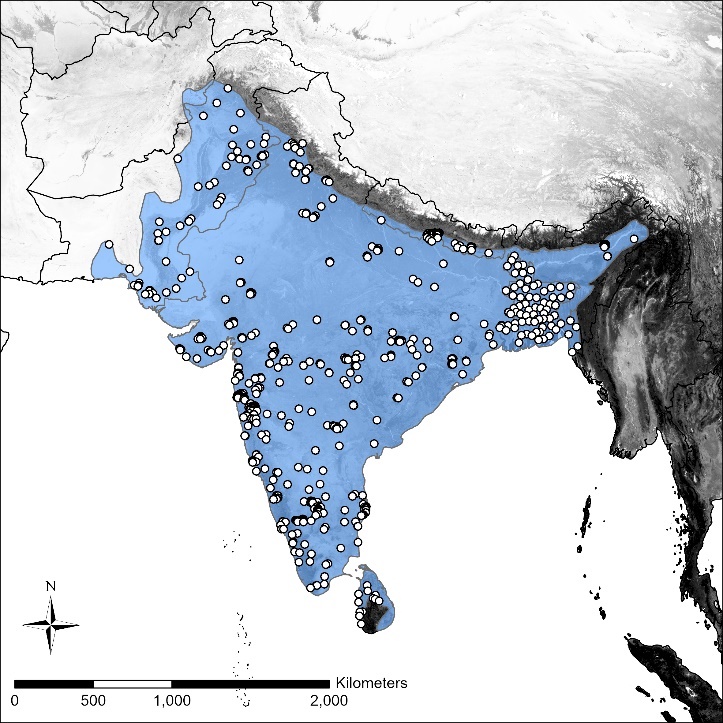


**Fig A.** Examples of occurrence records and expert derived ranges (blue) before (A & C) and after (B & D) our iterative review process for *Naja haje* (A & B), and *Bungarus caeruleus* (C & D). Points show original data from GBIF (A & C) and final datasets after data mining (B & D) for both species. Background shading shows vegetation ‘greenness’ (derived from Copernicus 10-daily time series of remotely sensed information on fraction photosynthetic active radiation summarized across years 2010-2019 (CC BY 4.0)^24^. Basemap shows WHO admin 0 country boundaries 2024 (CC BY 4.0). Maps were created in ESRI ArcPro 3.1.0^25^.

# Environmental Data

We used environmental data layers from a broad range of sources, including climate data from WorldClim 2.1^26^, topography^27^, vegetation characteristics^24,28,29^ and land use data^30^ from Copernicus, ISRIC soil properties^31,32^, water availability derived from HydroSHEDS’ HydroATLAS^33-36^, and human population density from WorldPop^37^. All original datasets were converted to raster where necessary and resampled or aggregated to the same 0.01 decimal degree resolution with Geographic Coordinate System World Geodetic System 1984 (GCS WGS84) projection and a global extent (see **Sup. Mat. 4** for a detailed description of data layers and their processing; layers are included in the data repository for this study and can be accessed by researchers for other projects and analyses).

The initial candidate variables described above were assessed for collinearity using Pearson’s correlation coefficient. If any variables had a correlation coefficient above 0.8 only one of the collinear variables was maintained. While this may be a high threshold for collinearity, it has been shown that Maxent is very robust to issues associated with collinearity^38^ and that including some colinear variables can explain important residual variation, especially when using variable selection, since colinear variables that do not provide improvements in model predictions are simply excluded in the process. Which collinear variables we dropped depended on a variety of factors including which variable was from higher quality data sources, was more biologically relevant, and was more commonly relevant to snakes’ biology based on expert knowledge. Only two sets of variables with correlation greater than 0.8 were maintained. These were mean dry matter productivity vs. mean fraction photosynthetic active radiation (0.86) and temperature seasonality vs. minimum temperature of coldest month (-0.88). In both cases the decision to maintain both variables was based on expert experience that they often explained residual variation (i.e. they often improved models substantially despite a collinear variable being already included; Pintor pers. obs.). Note that Maxent (i) deals very well with collinearity^38^, and (ii) any redundant variables would have been dropped in our variable selection process described below. The preliminary variable selection process resulted in the following set of 24 candidate variables (examples shown in **Fig B**) that were used for initial models and were later reduced further by MU-specific variable selection:

## Climate (10):

- Temperature Seasonality (Standard Deviation ×100)
- Max Temperature of Warmest Month
- Min Temperature of Coldest Month
- Annual Precipitation
- Precipitation Seasonality (Coefficient of Variation)
- Precipitation of Driest Quarter
- Precipitation of Warmest Quarter
- Radiation of lowest month
- Radiation of highest month
- Minimum Relative Humidity of Driest Month

*Notes:* note that mean annual temperature was excluded because it was highly correlated to minimum temperature, which was considered more biologically relevant because low temperatures are known to limit reptile distributions^39^.

## Topography (2):

- Topographic Ruggedness Index
- Modified ‘Northness’ (degree of orientation towards the equator based on northness/southness weighted by latitude)

Notes: slope was excluded because it was highly correlated with ruggedness; elevation was considered not biologically relevant since distribution limits along elevational and latitudinal gradients usually relate to colinear environmental variables such as temperature and not elevation/latitude itself.


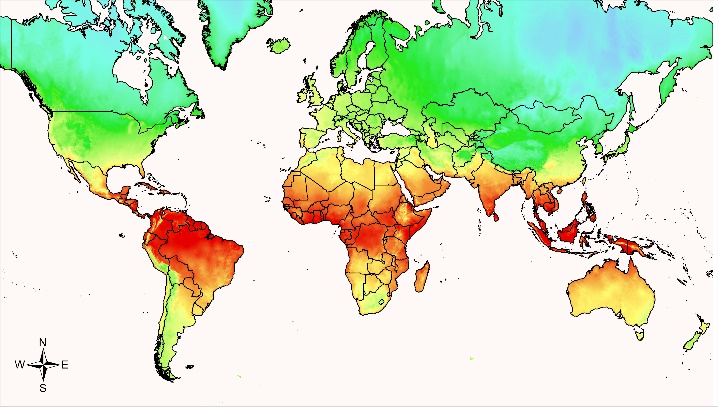

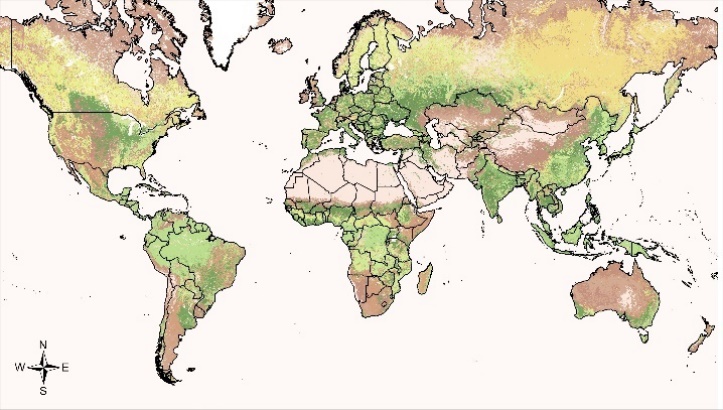

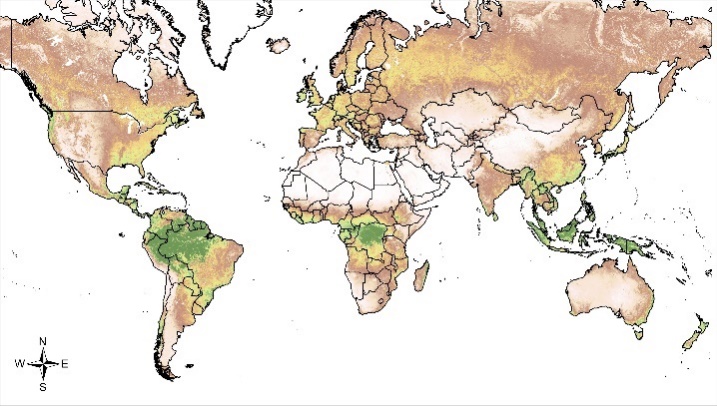

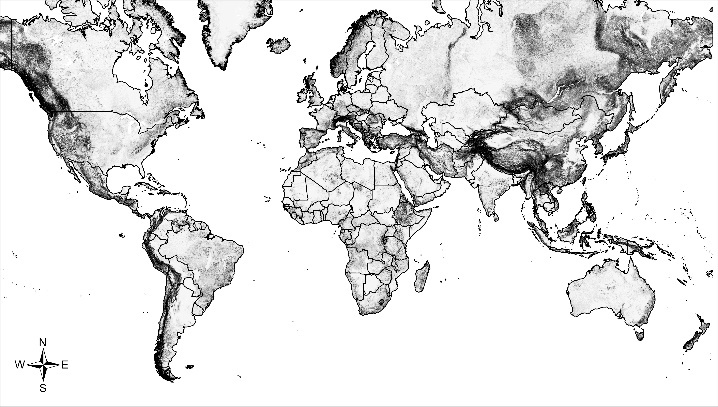

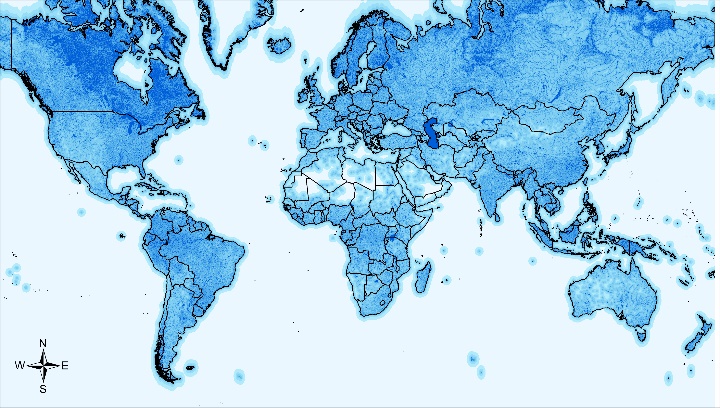

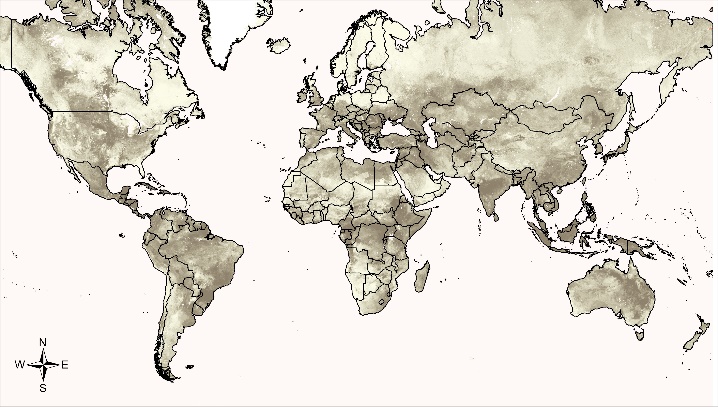


**Fig B**. Examples of environmental Input variables used for environmental niche models: minimum temperature (A)^26^, land use (B)^30^, mean fraction photosynthetic active radiation (C)^24^, topographic ruggedness index (D)^27^, distance to permanent freshwater (E)^30,40^, and percent clay in topsoil (F)^32,41^. Basemap shows WHO admin 0 country boundaries 2024 (CC BY 4.0). Maps were created in ESRI ArcPro 3.1.0^25^.

## Vegetation (4):

- Mean Dry Matter Productivity (how much biomass is produced)
- Annual Range of Dry Matter Productivity (how much does biomass production vary across the year)
- Mean Fraction Photosynthetic Active Radiation (how green is it)
- Annual Range of Fraction Photosynthetic Active Radiation (how much does greenness vary throughout the year)

## Land Use (1):

- ESA landcover type 2018 (how is the land currently used or what vegetation type is it)

## Soil (5):

- ISRIC topsoil bulk density (how dense is the soil)
- ISRIC topsoil fraction of coarse fragment (how coarse is the soil)
- ISRIC topsoil percent clay (proxy of soil water retention)
- ISRIC topsoil percent organic carbon (how organic is the soil)
- ISRIC most probable soil type

## Water (1):

- Distance to Permanent Water (euclidean distance to combined ESA & HydroSheds water bodies and HydroSheds rivers with more than 1m^3^/sec runoff in the driest month)

## Human Influence (1):

- WorldPop People per Grid Cell (how densely populated is it)

Notes: the degree to which humans modify a landscape can influence habitat suitability through direct influences (e.g. hunting) or indirect influences (e.g. habitat degradation or supply of food sources) and human population density was used as a proxy to model snake affinity or aversion to human occupied habitat; while population density may affect likelihood of snakes being observed, it is unlikely to confound models because detection bias is already accounted for in the model by using a target background (see modelling methods below).

## Future Climate (10 x 7)

- All WorldClim 2.1 climate variables were also created for two future conditions (2050 and 2090 centered) using CMIP6 pathway SSP5-8.5 (business as usual). SSP5-8.5 represents a more pessimistic ‘worst case scenario’. Reality is likely to lie somewhere between current conditions and SSP5-8.5. However, recent reports show that targets to reduce climate change are not currently being met^42^ and SSP5-8.5 is, therefore, most likely to represent the pathway the World is currently following. It is also more pragmatic to use a worst-case scenario for future proofing snakebite mitigation strategies since it is better to be prepared for this option, even if reality ends up being closer to current conditions and less change in strategy is eventually needed. Future climate for relative humidity and radiation were not available from WorldClim and were instead calculated from change grids sourced from Copernicus^43^. Because Copernicus only had this data available for a subset of general circulation models (GCMs), only those 7 scenarios available from both WorldClim and Copernicus were used in our projections:: (1) CanESM5-CanOE (Canada); (2) CMCC-ESM2 (Italy); (3) EC-Earth3-Veg-LR (Europe); (4) FIO-ESM-2-0 (China); (5) INM-CM4-8 (Russia); (6) INM-CM5-0 (Russia); (7) MPI-ESM1-2-LR (Germany).

# Modelling Methods

## Model Preparation & Parameters

The environmental niche of each MU was modelled using Maxent 3.4.4 software^44^, which has repeatedly been shown to outperform most other distribution modelling techniques^45^ even for data poor species and especially for species for which only presence data (no true absence data) is available. Maxent uses a machine learning algorithm to model habitat suitability as a function of environmental data at each presence location compared to background data. Under the null model, species are assumed to be equally likely to occur anywhere in the landscape. The background data ideally represents the space that has been available for colonization by the modelled species throughout its recent evolutionary history^45-48^, a subset of which has been successfully colonized based on the species’ environmental tolerances and requirements (presences).

Model performance is usually improved by using a ‘target group’ background^46,48,49^ to reduce the effect of spatial sampling bias, i.e. by using background data from selected locations that represent the differences in sampling effort across the background area, especially when data are collected from disparate sources, and when correcting universally for sampling is not possible because sampling effort is unknown^47^. Usually, a target background is created by combining all locations where species similar to the modelled one have been observed, i.e. sampling has taken place, and the species would have therefore likely been recorded if it was present. This target background includes the occurrence records for the modelled species, i.e. it does not present ‘pseudo-absences’. To create a target group background suitable for our MUs, we first combined all occurrence data for any Colubridae (Colubrinae & Natricinae), Viperidae, Elapidae, and Lamprophiidae found in any of the databases we accessed, i.e. data for all snake families that contained medically relevant species. We further supplemented the dataset with all other snake records (irrespective of family) available in GBIF^4^. This general background was then cut down to a buffer around all occurrences of each MU (**Fig C**). To approximate the area which each MU could have reasonably had access to in their recent evolutionary history, the size of the buffer was determined by the maximum range width of each MU in either latitudinal or longitudinal direction, whichever was bigger. However, the minimum buffer size was kept at 1000km around occurrences for highly restricted species and the maximum at 3000km for very wide-ranging species to avoid unrealistically large or small constraints that could lead to over- or underfitting of models. Ideally, one would use dispersal rates adjusted to an evolutionarily relevant historical time frame, but these are poorly known for almost all snakes. However, range size is often related to dispersal ad hence it was used as a proxy^50^.


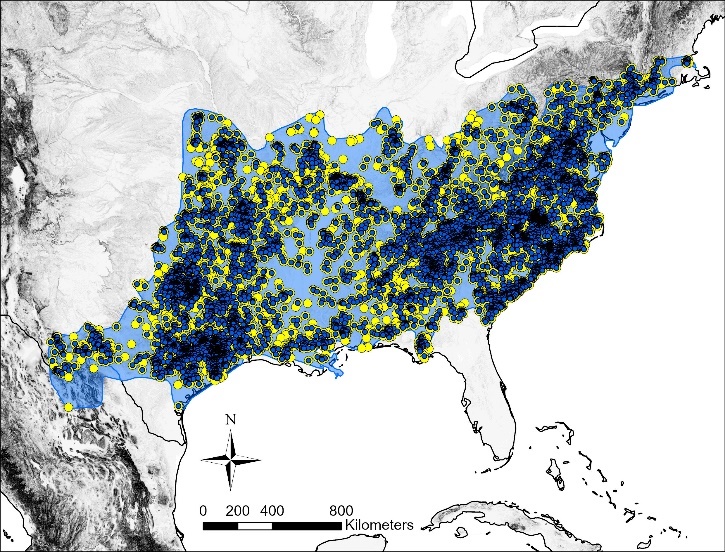

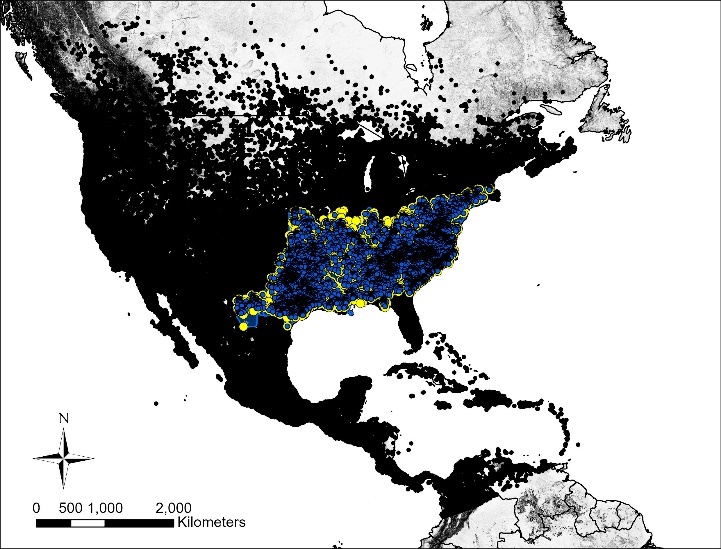

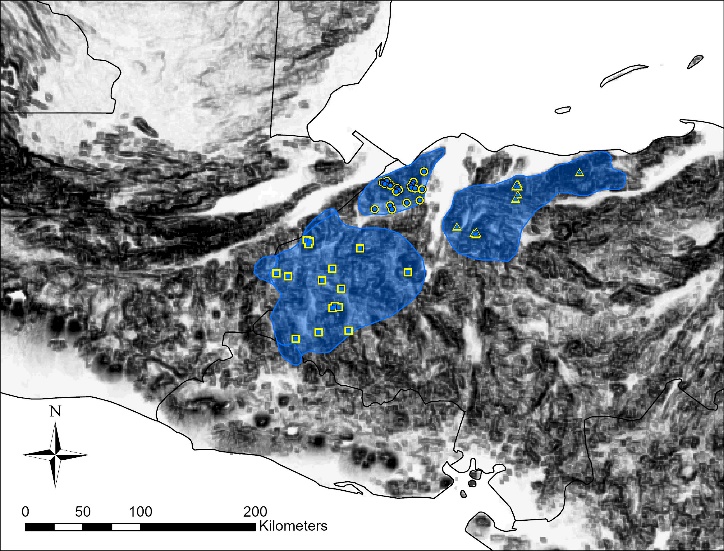

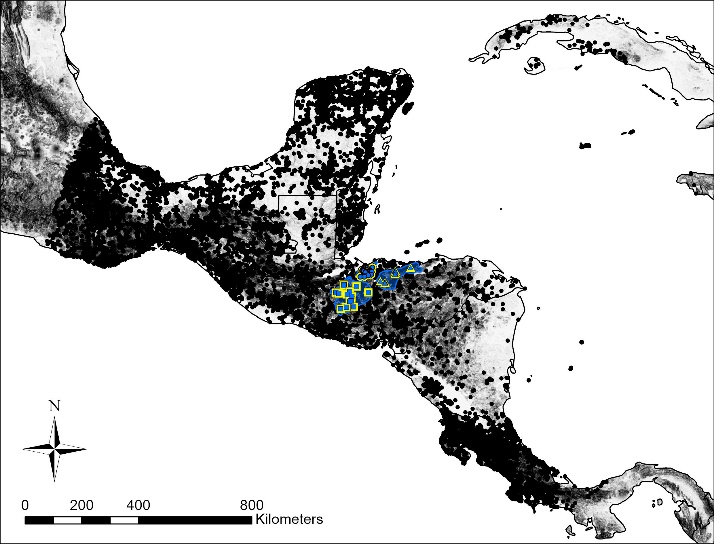


**Fig C**. Examples of model background and occurrence records for a wide ranging (A & B; *Agkistrodon contortrix*) and restricted (C & D*; Bothriechis marchi*, *B. thalassinus*, and *B. guifarroi*) MU. Black points in zoomed out views (B & D) show background points; large yellow points, triangles, and squares show full occurrence data sets for each species; smaller blue symbols show corresponding final datasets used in distribution models. Note that for the data rich species in A & B the model background was correspondingly larger than for the restricted MU, and that the restricted MU combines three closely related, ecologically similar species occurring in close proximity to each other and includes all available data for them to fulfill minimum data requirements for the model. Background shading shows topographic ruggedness based on Amatulli et al. 2018^27^. Basemap shows WHO admin 0 country boundaries 2024 (CC BY 4.0). Maps were created in ESRI ArcPro 3.1.0^25^.

Data from all 24 environmental raster layers was extracted for each latitude and longitude of the MU presence and background samples and fed into Maxent to create ‘starting’ models. Maxent was run with 10-fold cross-validation using 70% of randomly subsampled presence locations as training data and 30% as test data, without extrapolation. We allowed for linear, quadratic, product, and hinge features except for species that were both, data sparse (<80 records^47^) and restricted (background buffer <1000km). For these, no product features were allowed to avoid overfitting. No threshold features were used. All other Maxent parameters were kept at default values. The default logistic model Maxent parameters were used instead of species-specific tuning (i.e. the default convergence threshold, regulation, and count of iterations) to ensure that methods were comparable between species, and that output relative probability estimates were ranged between 0 and 1.

## Variable Selection

Permutation importance of each variable on each initial ‘starting’ model was averaged across all 10 subsampled runs and variables ranked accordingly within each model. For each MU variables with a mean permutation importance (which has been shown to correlate with empirically measured biological relevance of variables^51^) less than 1% were excluded and variable lists were further reduced to a maximum of 1/20 of the number of occurrence records (e.g. an MU with 200 records would have had no more than 10 final variables), to avoid model overfitting by balancing numbers of data points and predictors. However, the 5 variables with highest permutation importance were retained even for data poor species and even if their permutation importance was below 1% to avoid oversimplification of niche requirements^52^. The variable selection process resulted in the most parsimonious model, i.e. the model with limited and most contributing predictors, being selected.

Model results for very data poor species (<20 records) need to be examined critically, require ongoing improvements, and should be used cautiously until more information for such MUs becomes available. Despite combining data poor taxa into MUs where possible, a few MUs (~10) remained for which this was not sufficient (e.g. not enough closely related & ecologically similar species were available to combine them with). Even MUs with 20 to 50 records (~60) should be considered relatively data poor and require ongoing improvements. However, the intensive data mining conducted for this study led to the majority of MUs reaching much more satisfactory sample sizes.

Once a final variable list was compiled for each MU, a second set of ‘reduced’ cross-validated models was run for final model evaluation, using the same Maxent settings as before and 70/30% subsampling to test model performance. Finally, a ‘full’ model using all available records for training was run and projected to the landscape at 0.01 decimal degrees in GCS WGS84. All maxent outputs (response curves, jackknife tests, Maxent results .csv files, HTML model summaries, etc.) are supplied in our final database. Lastly, all models were projected to future conditions using the 7 future GCMs for SSP5-8.5 for 2050 and 2090. Future projection for each MU were summarized across the 7 GCMs by calculating the median (most likely), 90% quantile (~maximum likely) and 10% quantile (~minimum likely) future habitat suitability. Only climate was varied between current and future conditions and all other variables were kept constant. While some variables are not static in reality, they are often heavily influenced by human decision-making processes (e.g. land use and vegetation changes) and varying them would have to be based on singular predictions available in the literature (i.e. no uncertainty estimates would be possible). Hence, we opted to avoid making assumptions about their future patterns.

# Final Model Processing

## Thresholding

We used one of the more generous thresholds suggested by Maxent: ‘balance training omission, predicted area and threshold value’. This threshold was chosen because species’ range overprediction was considered more favourable for our purposes than underprediction, because it let us predict marginal areas where human exposure to snakebite is possible even if rare. Snake detection ability tends to be low compared to other animals^53,54^ and it is, therefore, a reasonable precaution to assume rare occurrences in marginal habitat. This was further justified by using a cost distance restriction to remove any suitable habitat that is unlikely to be occupied from our final distribution estimates (see below) and therefore reduce any potential overestimation caused by a more relaxed threshold. Models finalized with a threshold were rescaled to 0-1 to make outputs for different species with different threshold values comparable (See **Sup. Mat. 2** for species-specific threshold values).

## Cost Distance Calculations

For each MU we calculated the cost distance from each known reliable occurrence point, using habitat suitability as a ‘cost raster’, also known as ‘friction surface’.^55,56^ A cost raster assumes that every grid cell has a cost associated with traveling through it proportionate to its habitat suitability. Essentially, a cell with habitat suitability close to 1 is easy to access and likely occupied if a nearby grid cell is known to be occupied, but a cell with low habitat suitability closer to 0 likely presents a limitation to the MU expanding its range in that direction. The cost distance from known occurrences then functions as a measure of how certain we are that any grid cell is occupied by the MU based on how much resistance it has to overcome to reach it (in cost distance terms). Likelihood of occupancy decreases faster across a series of low-suitability cells, and suitable cells separated from known occurrences by unsuitable habitat are considered inaccessible and likely unoccupied. This cost-distance approach has previously been used to assess spatial separation of genetic lineages based on known locations of each lineage and habitat suitability^55,56^.

Using this cost distance approach, we excluded any areas from each final MU model that were more than 500 cost distance units (500 cost distance ‘km’) away from known occurrences (**Fig D**). This cut out any completely disconnected suitable areas, such as land masses separated by water or other dispersal barriers, as well as any marginal areas (lower suitability areas connected to, but far from known occurrences). Outputs were additionally cut to within the model’s target background area to avoid projecting habitat outside of the area used as model inputs.

Lastly, we separated habitat suitability models of any species that had been modelled jointly in an MU, by assigning each grid cell to that species for which cost distance was lowest at that location. We allowed individual species’ distributions within the same modelling unit to overlap by the same 500 cost distance units used above, but faded out habitat suitability in overlap areas proportional to overlap ratio (e.g. at a cost distance of 250 for each of two overlapping species, both had their suitability downweighed by 50%, but at a 400:100 cost distance ratio the MU species further away would have been downweighed by 80% and the other MU species closer to the grid cell only by 20%). The downweighed output was then re-thresholded with the original Maxent threshold, thereby eliminating any cells of low suitability close to another species in the MU. This novel overlap and fade-out method was used to capture potential contact zones and allow for some uncertainty in the range delimitation between species that were modelled as one MU.


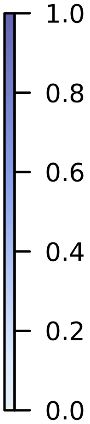

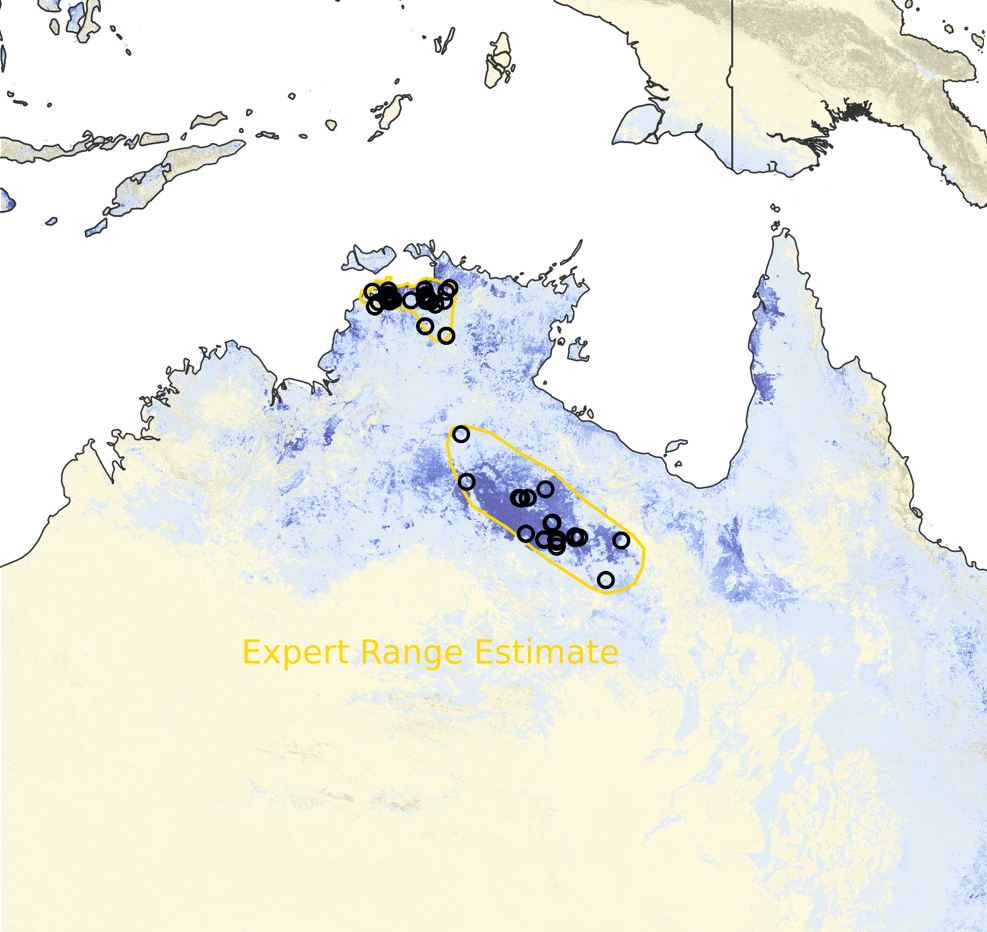

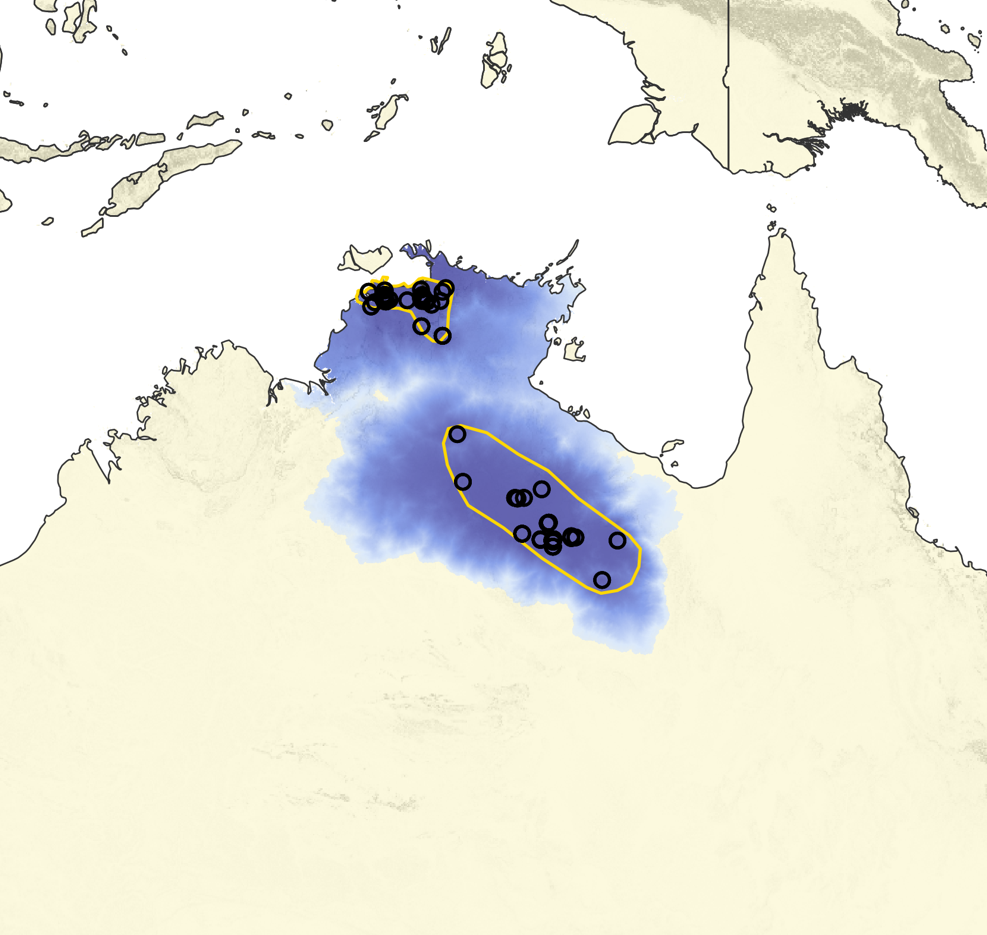

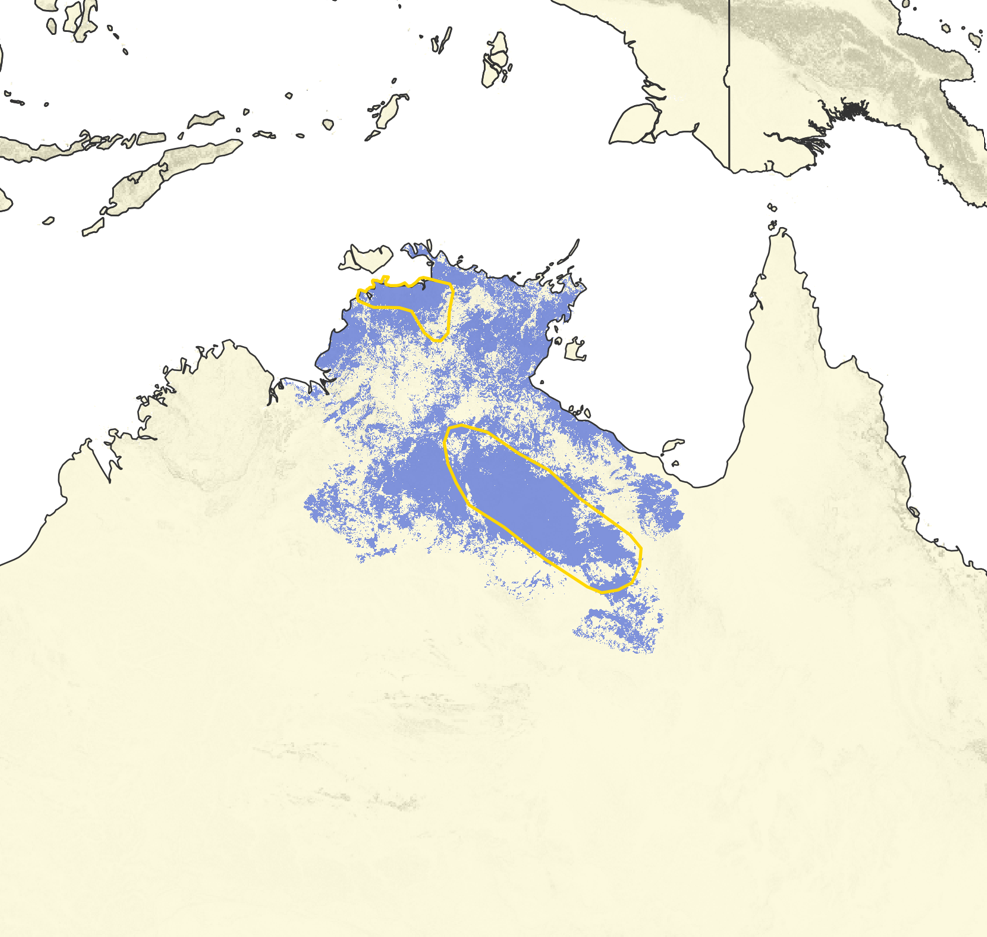

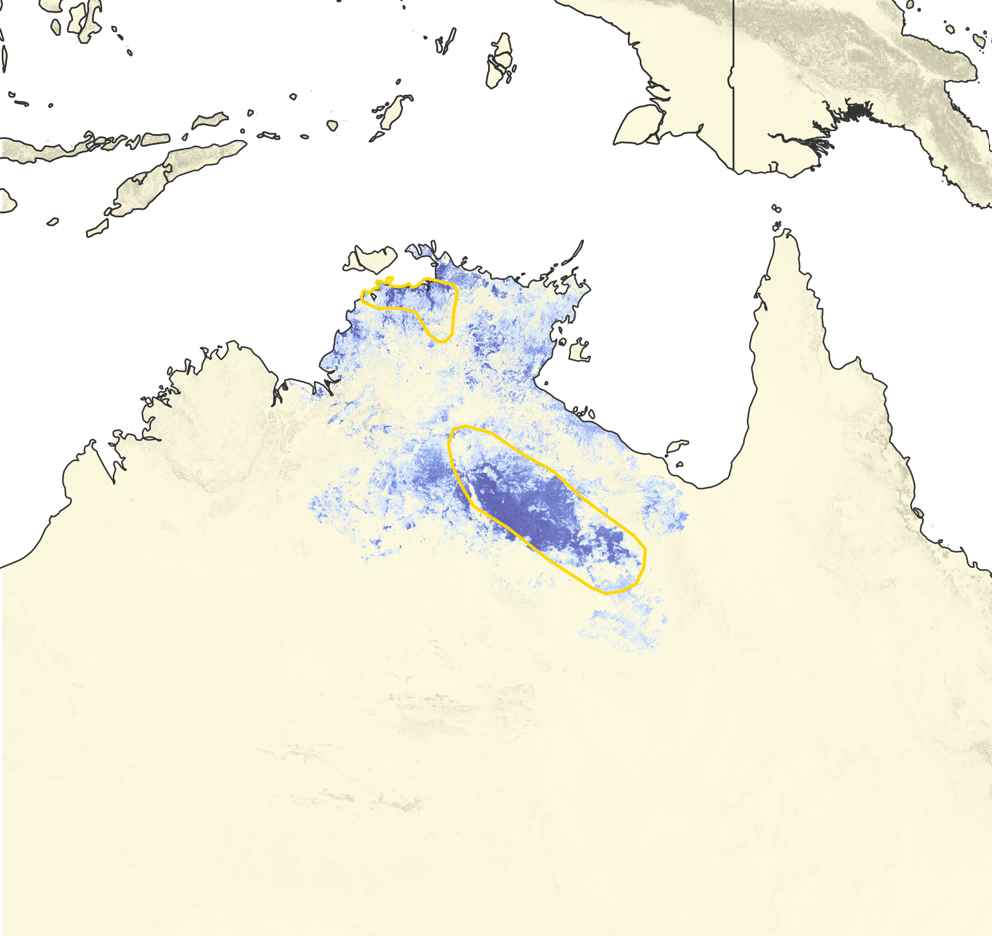


A

B

C

D

E

F


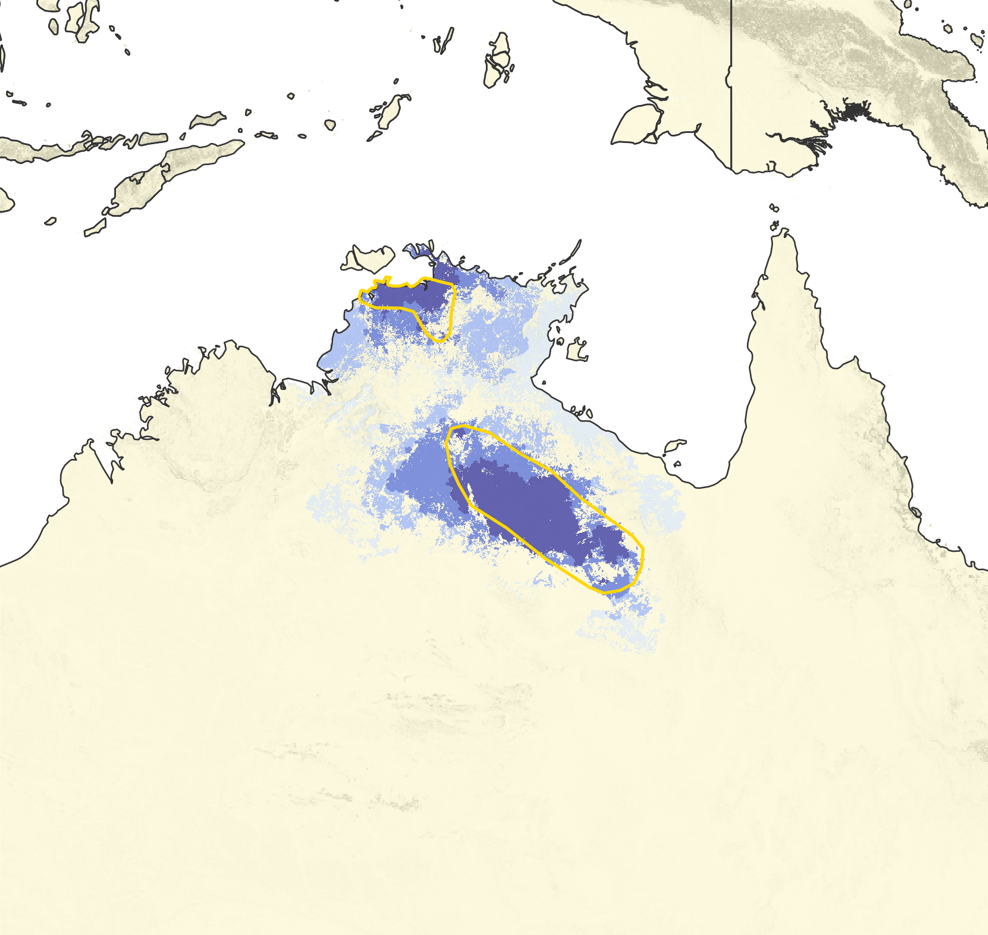

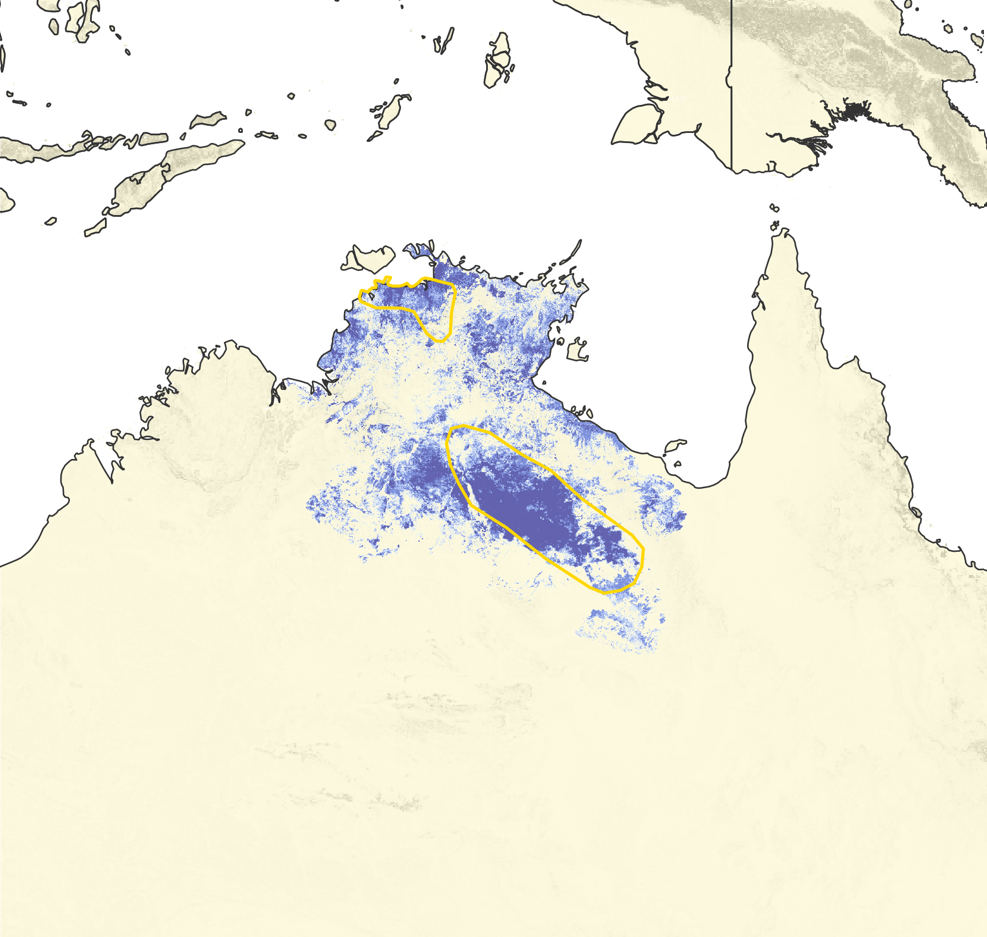


**Fig D**. Example of model output processing – raw environmental niche model with no threshold applied (A), inverted relative cost distance within 500 cost distance units (B), binary (C) and continuous (D) final thresholded and cost distance restricted habitat suitability, quartiles of cost distance (E) and quartiles of suitability (F) for *Acanthophis hawkei* across northern Australia. The expert derived range is shown in yellow for reference. Background shading shows topographic ruggedness based on Amatulli et al. 2018^27^. Basemap shows WHO admin 0 country boundaries 2024 (CC BY 4.0). Maps were created in ESRI ArcPro 3.1.0^25^

The resulting database of species’ habitat suitability estimates comprises the raw as well as the thresholded suitability rasters for each modelling unit, the absolute cost distance rasters for each individual species, the relative inverted cost distance (0-500 absolute cost distance converted to 1-0 with anything past 500 being 0) rasters for each individual species, and the final, thresholded, and cost distance restricted individual species’ habitat suitability rasters as continuous and binary versions. Individual species outputs were also converted to shapefiles showing (i) habitat suitability categorized into 4 bins according to quartiles of suitable cell values, (ii) habitat suitability categorized into 4 equal bins (0-0.25; 0.25-0.5; 0.5-0.75; 0.75-1), (iii) relative cost distance categorized into 4 bins according to quartiles of cell values, and (iv) known areas of occurrence (any suitable areas within 25 km of known occurrences).

## Summary Outputs

In addition to the species outputs described above, we created summary rasters for closely related species groups or clades (e.g. all African spiting cobras from the ‘*Afronaja’* clade, or all carpet vipers [*Echis* spp.]; **Table 1**). These rasters show the maximum value of habitat suitability for any species in the relevant group, based on the final individual species outputs. Groups were compiled by combining all closely related species that are unlikely to truly overlap in their distributions, i.e. where any apparent overlap zones are more likely to represent transition zones, meaning their suitability is not additive in contact zones. In most cases we expect one of these species to dominate in each grid cell and the species for which habitat suitability is greatest in any grid cell is likely to be the dominant one. See **Sup. Mat. 2** for details of which species were included in each of these groups.

Furthermore, we created ‘hotspot’ maps that show species richness (both for EDRs and based on distribution models) and cumulative relative suitability across the globe. We used the outputs for groups of species (see paragraph above) rather than individual species to create these, to avoid artificially inflating species richness in transition zones between species (i.e. the further a species is split up into less wide ranging species the more transition zones between these new species are created and the more species richness is artificially inflated in these zones because of uncertainty of which member of the group is dominant there). Species richness shows the sum of all species groups present based on binary suitability maps (suitable [1] vs. unsuitable [0]), while cumulative relative suitability is the sum of all suitability maps (0-1), i.e. high values mean high suitability for many different species. High cumulative suitability could be considered a proxy of total abundance in some cases. However, this output has to be considered with caution, because different snake taxa have different maximum abundances (i.e. some populate their ideal habitat more densely than others) and relative suitability may, therefore, not always be additive. Ideally, each relative suitability map should be weighed by known values for species specific maximum abundance in the future, if such data become available. Species richness and/or cumulative suitability have previously been used successfully as predictors of snakebite incidence, especially if further weighed by relevant species traits^57,58^.

Lastly, we multiplied the suitability across the predicted distribution for each individual species with the natural logarithm of human population density to get an estimate of how much snake populations and human populations overlap (Snake-Human Overlap Index; SHOI). We also created a cumulative global SHOI similar to the global cumulative relative suitability. However, this output should be treated with the same caution as mentioned above for cumulative suitability due to the difficulties weighing contributions from individual snake groups.

All outputs are available for current climate conditions and Median, 10% quantile, and 90% quantile for the year 2050 and 2090 under an SSP5-8.5 pathway, calculated across the 7 different GCMs.

## Climate Change Summaries

In addition to the outputs for current and future habitat suitability for individual species, species groups, and global patterns across all species, we also created an additional set of rasters that specifically summarize how climate change will affect snake distributions and diversity. These include the absolute change in suitability for individual species (i.e. the difference between current and future habitat suitability), areas with predicted species distribution expansions and contractions, and how global patterns in suitability, species richness, and exposure will change. We also provide a raster of the sum of all distribution expansions and contractions, and of species turnover rate [(lost species + gained species) / (total species present currently or in future).

# Statistics

All statistical analyses were performed in R^59^. Circular statistics and distance calculations were performed using packages circular and gdistance^60,61^ GIS analyses and data processing were either performed in R^59^, ArcGIS Pro^25^, and ArcMap 10.7^62^.

Model performance was assessed using area under the curve (test and training AUC) and partial receiver operating characteristic (ROC)^63^. Additionally, models were validated by comparing them to EDRs and assessing omission (false negative; model predicts unsuitable habitat within EDR), commission (false positive; model predicts suitable area outside EDR), and congruence (both models and EDR predict occupied area). Combining EDRs with distribution models has previously proven advantageous^57^.

For AUC, Maxent outputs were used. Partial ROC compares 1-omission error of sample points at different percentages of predicted area at different threshold values to 1-omission error of random points at the same percentages of predicted are, using the area under both curves. To calculate partial ROC, we ran 39 iterations comparing the ROC of 50% sub-sampled occurrence points to the ROC of the same number of randomly selected points (null expectation). The number of thresholds at which 1-omission error was calculated for the percent of predicted area at that threshold was varied depending on the total area used in the final model and the number of occurrence points. MUs that occurred over very large areas or had very large numbers of records used less threshold steps due to limitations in computation resources. The maximum number of ‘steps’ was kept at 100 for data poor or restricted taxa. The lowest number of ‘steps’ used was 11 for the most wide-spread, data rich taxon (i.e. 1-ommission error was only assessed at 0, 0.1, 0.2, 0.3, 0.4, 0.5, 0.6, 0.7, 0.8, 0.9, and 1% predicted area for *Vipera berus* but at 0.01% intervals for *Eristicophis macmahonii*).

Trends in species’ range size, total suitability, total exposure, and range shift vectors were summarized individually and by biogeographic region. For our purposes, we defined biogeographic regions as sets of WHO recognized countries that share a substantial number of snake species. The ten regions we use are Australasia, Southeast Asia, South Asia, East Asia, Middle East and Central Asia, Europe and Russia, North America, South America, Central America, and Africa. Many of these regions show overlaps in snake species but they all possess sets of species that are very typical of the region, such as the elapids of the genera *Acanthophis*, *Pseudonaja* and *Oxyuranus* in Australasia; *Dendroaspis*, *Bitis* and the sub-genera *Afronaja*, *Boulengerina*, and *Uraeus* in Africa; *Macrovipera*, *Montivipera*, *Walterinnesia*, *Eristicophis* and *Pseudocerastes* in the Middle East; *Ophiophagus*, *Calloselasma*, *Calliophis*, and Asian spitting cobras (*Naja* spp.), along with many *Bungarus* and *Trimeresurus* species in South East Asia; the big four, *Echis* *carinatus*, *Naja* *naja*, *Bungarus* *caeruleus*, and *Daboia* *russelii* in South Asia; *Gloydius*, *Deinagkistrodon*, *Rhabdophis*, and *Protobothrops* in East Asia; different dominant species of *Crotalus*, *Agkistrodon*, *Bothrops*, and *Micrurus* in South, Central, and North America; and *Vipera* in Europe and Russia. Summaries for regions always included all species present in the region, not only the dominant or unique ones.

Individual species trends in distribution size, total habitat suitability, and SHOI with climate change were assessed by calculating the number of occupied grid cells (range size), number of newly occupied cells (range expansion) and newly unoccupied cells (range contraction), sum of all suitable cells (total suitability), and sum of SHOI in all occupied cells (total exposure) at each time step (2050 and 2090).

Direction of range shift vectors were calculated based on the current and future distribution centroid (mean of latitudinal and longitudinal coordinates of all suitable grid cells). Vector length (shift distance in meters) was calculated as

*D1 = R * (2 * atan2 (*

*sqrt ((sine ((Latfuture-Latcurrent)/2) * sine ((Latfuture-Latcurrent)/2)) + (cosine (Latcurrent) * cosine (Latfuture) * sine ((Lonfuture-Loncurrent)/2) * sine ((Lonfuture-Loncurrent)/2))),*

*sqrt( 1 - ((sine ((Latfuture-Latcurrent)/2) * sine ((Latfuture-Latcurrent)/2)) + (cosine (Latcurrent) * cosine (Latfuture) * sine ((Lonfuture-Loncurrent)/2) * sine ((Lonfuture-Loncurrent)/2))))))*

Where D1 is distance in meters, R is the radius of the Earth in meters (6371000), and Lat and Lon are the latitudinal and longitudinal position of point on sphere in radians.

Vector direction was calculated as

*D2 = (180/pi) * atan2(*

*(sine (Lonfuture-Loncurrent) * cosine (Latfuture)) ,*

*(cosine (Latcurrent) * sine (Latfuture)) – (sine (Latcurrent) * cosine (Latfuture) * cosine (Lonfuture-Loncurrent)))*

Where D2 is the circular direction in degrees.

# References

1 Uetz, P. *et al.* The Reptile Database (<http://www.reptile-database.org>). (2024).

2 WHO. Annex 5 to Guidelines for the Production, Control and Regulation of Snake Antivenom Immunoglobulins (<https://www.who.int/publications/m/item/snake-antivenom-immunoglobulins-annex-5-trs-no-1004>). (2018).

3 Longbottom, J. *et al.* Vulnerability to snakebite envenoming: a global mapping of hotspots. *The Lancet* **392**, 673-684 (2018).

4 GBIF.org. GBIF Home Page. Available from: <https://www.gbif.org> [13 January 2020]. (2024).

5 VertNet [2020]. <http://vertnet.org/>.

6 ALA [2020]. <https://www.ala.org.au/>.

7 iDigBio [2023]. <https://www.idigbio.org/>.

8 Arctos [2020]. <https://arctosdb.org/>.

9 iNaturalist [2020]. <https://www.inaturalist.org/>

10 HerpMapper [2020] <https://www.herpmapper.org/>.

11 The Snake Atlas for Namibia [2020]. <http://www.the-eis.com/atlas/?q=atlas-of-snakes>.

12 Thai National Parks [2021]. <https://www.thainationalparks.com/>.

13 Reptile Atlas of Africa [2021]. <http://vmus.adu.org.za>.

14 Observations.org [2021]. <https://www.observations.org/>.

15 Herpetology of Ethiopia and Eritrea [2021]. <http://www.reptiles-of-ethiopia-and-eritrea.com/elapidae.html>.

16 India Biodiversity Portal [2021]. <https://indiabiodiversity.org/>.

17 Mark O’Shea’s Snakes on Islands [2021] - accessed through Mark O'Shea.

18 Kenyan Reptile Atlas [2024]. <https://kenyareptileatlas.com/>.

19 Spawls, S., Mohammad, A. & Mazuch, T. *Handbook of Amphibians and Reptiles of North-east Africa*. (Bloomsbury Publishing, 2023).

20 Chippaux, J.-P. & Jackson, K. *Snakes of central and western Africa*. (JHU Press, 2019).

21 Chirio, L. & LeBreton, M. *Atlas des reptiles du Cameroun*. Vol. 67 (IRD Editions, 2007).

22 Google. Google Maps (<https://www.google.com/maps/>). (2024).

23 Stockwell, D. R. & Peterson, A. T. Effects of sample size on accuracy of species distribution models. *Ecological modelling* **148**, 1-13 (2002).

24 Copernicus. Land Monitoring Service: Fraction of Absorbed Photosynthetically Active Radiation 1999-2020 (raster 1 km), global, 10-daily – version 2 (<https://land.copernicus.eu/en/products/vegetation/fraction-of-absorbed-photosynthetically-active-radiation-v2-0-1km>). (2020).

25 ESRI. ArcGIS Pro 3.1.0 (2023).

26 Fick, S. E. & Hijmans, R. J. WorldClim 2: new 1‐km spatial resolution climate surfaces for global land areas (<https://www.worldclim.org/data/worldclim21.html>). *International journal of climatology* **37**, 4302-4315 (2017).

27 Amatulli, G. *et al.* A suite of global, cross-scale topographic variables for environmental and biodiversity modeling. *Scientific data* **5**, 1-15 (2018).

28 Copernicus. Land Monitoring Service: Dry Matter Productivity 1999-2020 (raster 1 km), global, 10-daily – version 2 (<https://land.copernicus.eu/en/products/vegetation/dry-matter-productivity-v2-0-1km>). (2020).

29 Copernicus. Land Mopnitoring Service: Leaf Area Index 1999-2020 (raster 1 km), global, 10-daily – version 2 (<https://land.copernicus.eu/en/products/vegetation/leaf-area-index-v2-0-1km>). (2020).

30 Copernicus. Climate Change Service, Climate Data Store; Land cover classification gridded maps from 1992 to present derived from satellite observation. Copernicus Climate Change Service (C3S) Climate Data Store (CDS). DOI: 10.24381/cds.006f2c9a. (2019).

31 Batjes, N. H., Ribeiro, E. & Van Oostrum, A. Standardised soil profile data to support global mapping and modelling (WoSIS snapshot 2019). *Earth System Science Data* **12**, 299-320 (2020).

32 Poggio, L. *et al.* SoilGrids 2.0: producing soil information for the globe with quantified spatial uncertainty (<https://soilgrids.org/>). *Soil* **7**, 217-240 (2021).

33 Lehner, B. *et al.* in *EGU General Assembly Conference Abstracts.* EGU21-9277.

34 Wickel, B., Lehner, B. & Sindorf, N. in *AGU Fall Meeting Abstracts.* H11H-05.

35 Linke, S. *et al.* Global hydro-environmental sub-basin and river reach characteristics at high spatial resolution. *Scientific data* **6**, 283 (2019).

36 Lehner, B., Messager, M. L., Korver, M. C. & Linke, S. Global hydro-environmental lake characteristics at high spatial resolution. *Scientific Data* **9**, 351 (2022).

37 WorldPop. Estimated total number of people per grid-cell unconstrained global mosaic at 1 km resolution for 2020 - School of Geography and Environmental Science, University of Southampton; Department of Geography and Geosciences, University of Louisville; Departement de Geographie, Universite de Namur) and Center for International Earth Science Information Network (CIESIN), Columbia University. Global High Resolution Population Denominators Project - Funded by The Bill and Melinda Gates Foundation (OPP1134076). <https://dx.doi.org/10.5258/SOTON/WP00647> (2020).

38 Feng, X., Park, D. S., Liang, Y., Pandey, R. & Papeş, M. Collinearity in ecological niche modeling: Confusions and challenges. *Ecology and evolution* **9**, 10365-10376 (2019).

39 Pintor, A. F., Schwarzkopf, L. & Krockenberger, A. K. Rapoport's Rule: Do climatic variability gradients shape range extent? *Ecological Monographs* **85**, 643-659 (2015).

40 Keller, C. *et al.* The new hydrographic HydroSHEDS database derived from the TanDEM-X DEM (<https://www.hydrosheds.org/>). (2023).

41 de Sousa, L. M. *et al.* SoilGrids 2.0: producing quality-assessed soil information for the globe. *Soil discussions* **2020**, 1-37 (2020).

42 Liu, P. R. & Raftery, A. E. Country-based rate of emissions reductions should increase by 80% beyond nationally determined contributions to meet the 2 C target. *Communications earth & environment* **2**, 29 (2021).

43 Copernicus. Climate Change Service, Climate Data Store: CMIP6 climate projections. Copernicus Climate Change Service (C3S) Climate Data Store (CDS). DOI: 10.24381/cds.c866074c. (2021).

44 Philips, S., Dudík, M. & Schapire, R. (2018).

45 Elith, J. *et al.* A statistical explanation of MaxEnt for ecologists. *Diversity and distributions* **17**, 43-57 (2011).

46 Barber, R. A., Ball, S. G., Morris, R. K. & Gilbert, F. Target‐group backgrounds prove effective at correcting sampling bias in Maxent models. *Diversity and Distributions* **28**, 128-141 (2022).

47 Merow, C., Smith, M. J. & Silander Jr, J. A. A practical guide to MaxEnt for modeling species' distributions: what it does, and why inputs and settings matter. *Ecography* **36**, 1058-1069 (2013).

48 VanDerWal, J., Shoo, L. P., Graham, C. & Williams, S. E. Selecting pseudo-absence data for presence-only distribution modeling: how far should you stray from what you know? *Ecological modelling* **220**, 589-594 (2009).

49 Ranc, N. *et al.* Performance tradeoffs in target‐group bias correction for species distribution models. *Ecography* **40**, 1076-1087 (2017).

50 Alzate, A. & Onstein, R. E. Understanding the relationship between dispersal and range size. *Ecology Letters* **25**, 2303-2323 (2022).

51 Searcy, C. A. & Shaffer, H. B. Do ecological niche models accurately identify climatic determinants of species ranges? *The American Naturalist* **187**, 423-435 (2016).

52 Wan, J.-Z., Wang, C.-J. & Yu, F.-H. Effects of occurrence record number, environmental variable number, and spatial scales on MaxEnt distribution modelling for invasive plants. *Biologia* **74**, 757-766 (2019).

53 Boback, S. M., Nafus, M. G., Yackel Adams, A. A. & Reed, R. N. Use of visual surveys and radiotelemetry reveals sources of detection bias for a cryptic snake at low densities. *Ecosphere* **11**, e03000 (2020).

54 Durso, A. M., Willson, J. D. & Winne, C. T. Needles in haystacks: estimating detection probability and occupancy of rare and cryptic snakes. *Biological Conservation* **144**, 1508-1515 (2011).

55 Rosauer, D. *et al.* Phylogeography, hotspots and conservation priorities: an example from the Top End of Australia. *Biological Conservation* **204**, 83-93 (2016).

56 Rosauer, D. F., Catullo, R. A., VanDerWal, J., Moussalli, A. & Moritz, C. Lineage range estimation method reveals fine-scale endemism linked to Pleistocene stability in Australian rainforest herpetofauna. *PLoS One* **10**, e0126274 (2015).

57 Martín, G. *et al.* Integrating snake distribution, abundance and expert‐derived behavioural traits predicts snakebite risk. *Journal of Applied Ecology* **59**, 611-623 (2022).

58 Yañez-Arenas, C., Townsend Peterson, A., Rodríguez-Medina, K. & Barve, N. Mapping current and future potential snakebite risk in the new world. *Climatic Change* **134**, 697-711 (2016).

59 R Core Team 2021. R: A language and environment for statistical computing. R Foundation for Statistical Computing, Vienna, Austria (<https://www.R-project.org/>).

60 Lund, U. & Agostinelli, C. R package 'circular': Circular Statistics (version 0.5-0). URL <https://CRAN.R-project.org/package=circular>. (2023).

61 van Etten, J. R package gdistance: Distances and routes on geographical grids. *Journal of Statistical Software* **76**, 1-21 (2017).

62 ESRI. ArcMap 10.7. (2021).

63 Peterson, A. T., Papeş, M. & Soberón, J. Rethinking receiver operating characteristic analysis applications in ecological niche modeling. *Ecological modelling* **213**, 63-72 (2008).
