# Supplementary material for: Climate change induced complex shifts in snake distributions expose people to snakebite and threaten biodiversity
Source: PLoS Negl Trop Dis. 2026 May 21;20(5):e0014030. doi: 10.1371/journal.pntd.0014030 (PMC13193456; doi:10.1371/journal.pntd.0014030)
Supplement: S2 Text — (DOCX) [file pntd.0014030.s003.docx]

**Occurrence Locality Geo-referencing Guide**

This guide describes the standardized method for geo-referencing occurrence records of venomous snakes for WHO snake distribution models. Below are several examples for different kinds of occurrence records. The aim is to use Google Maps (<https://www.google.com/maps>) to derive a latitude and longitude value in decimal degrees WGS84 as well as a coordinate uncertainty estimate in meters for each of the locality descriptions obtained from the literature.

**Example 1: Detailed locality information available**

In this example, the locality information given is very detailed, i.e. a town, such as “Cabulja (road to rosne poljane) s. Ielo”. Type the locality information provided into Google Maps.

In this specific example, there is only one result for the locality and it is in the correct country, as noted in the data source. Select the locality from the drop-down menu and zoom into it. Place a marker somewhere near the locality in natural looking vegetation or, if no natural vegetation is available, in farmland by long right-clicking on that spot in the map. A little window appears with latitude/ longitude information. Click on it and the map will zoom into it. Copy the lat/long from the google search window on the left and paste them into the correct columns in your spreadsheet. If there are several records in your spreadsheet with the same locality, copy/ paste the lat/long for all of them.

Now imagine a circle encompassing your place marker and the original locality found by google. Estimate the diameter of that circle by comparing it to the scale bar at the bottom right of google maps. In this specific case it was approximately 150-200m. The most accurate relevant coordinate uncertainty we used for this project was 250m so record 250m as your confidence in meters for this record.

**Example 2: Locality refers to an approximate location such as a large city or ‘near city xyz’**

In this example there are several records with slightly different locality descriptions that clearly refer to the same city, eg. “Agra Uttar Pradesh India”, “Agra”, “Agra India”, etc. Thi city covers an area approximately 20km in diameter so we do not know where exactly in the city the snake was recorded. Snakes are most likely to occur in natural vegetation somewhere near the city rather than inside the city. Even if they were seen inside the city, they likely got there from surrounding areas with better habitat. Zoom into a natural looking area near the city. Place a marker somewhere near the city in natural habitat, such as savannah, or forest, or, if no natural habitat is visible, in farmland. Record the latitude/ longitude in decimal degrees as for the previous example for all records with this location.

Imagine a circle encompassing the whole city area and your new location marker. The snake was probably seen somewhere in this circle originally. This is your location uncertainty. Estimate the diameter of the circle using the google maps scale bar (in this case it was ~40km = 40,000 m) and record this as your location uncertainty.

**Example 3: Locality refers to a large, general area such as a district, province or country**

In this example we use several records that all refer to a province in Thailand (“Nakhon Ratchasima Prov Thailand”) as the occurrence locality. Google maps will show you a red outline of the area. We don’t know where the snake was seen exactly but it was somewhere within that area. Find natural vegetation somewhere reasonably central in the area and place a marker. Record that latitude/ longitude.

Now estimate how far your location is from the furthest edge of the red-outlined area, in this case it was about 100 km (=100,000 m). This is your uncertainty. IF YOUR UNCERTAINTY IS GREATER THAN 500 KM THE LOCALITY IS NOT USEFUL! RECORD IT AS ‘NA’ FOR LATITUDE/ LONGITUDE AND CONFIDENCE

**Example 4: Locality refers to a distance from a precise location**

In this example, a snake was seen 4 miles North North East of a town (“4 mi NNE Buenavista Mexico”). Find the town in Google maps and place a marker approximately 4 miles North North East of it in natural vegetation. Often this is near a road that leads out of town in that direction. This is you latitude/ longitude. Your confidence depends on how well you think you estimated the correct distance and direction from the town… it is usually 2-5km for short distance as in this example. If the location is given as 100km or greater from a place, your confidence is usually worse, closer to 10 or even 20km.

**Example 5: Locality information is too unclear to be useful**

Sometimes the locality information given is not useful. This is the case if the description refers to many different potential locations, such as very common town names without country or province information, or the description is not a location at all, such as an altitude or random text. In this case populate record latitude, longitude, and uncertainty as ‘NA’.

Sometimes the location information is very general such as ‘Africa’. If the location has an uncertainty of over 500km it is not useful. Record latitude, longitude, and uncertainty as ‘NA’.

If the locality information is theoretically useful, such as a farm name (e.g. “Farm Alkmaar”), but you cannot find it on google maps, you have two options:

1. Do a normal google search. Sometimes the location can be found in documents or in other mapping websites such as <https://www.mindat.org/>. Double check that the latitude/longitude you found makes sense in google maps and record it for your record. Your uncertainty depends on how sure you are of the location accuracy but for a farm it is usually at least 2-5km because named farms can be very large.
2. Leave the information as ‘unknown’. Somebody else might find the location in the future.
