# Supplementary material for: Climate change induced complex shifts in snake distributions expose people to snakebite and threaten biodiversity
Source: PLoS Negl Trop Dis. 2026 May 21;20(5):e0014030. doi: 10.1371/journal.pntd.0014030 (PMC13193456; doi:10.1371/journal.pntd.0014030)
Supplement: S3 Text — (DOCX) [file pntd.0014030.s004.docx]

**Potential Maxent Variable Categories, Sources, and Descriptions**

The below layers were created to represent typical attributes that are relevant to reptile species’ habitat suitability. They broadly fall into the categories of climate, topography, substrate and soil characteristics, vegetation characteristics or type, access to water, and human influences on natural habitats. Not all layers were eventually used for models because of collinearity of some, or because they made models too complex without adding new information, or because their biological relevance was not deemed high enough. However, the full set of layers is described and made available to enable other researchers to choose those most adequate for their purposes.

**Climate layers based on WorldClim:**

wc2.1_bio_30s_1_1kmWW.asc - wc2.1_bio_30s_19_1kmWW.asc; wc2.1_bio_30s_RADmin_1kmWW.asc; wc2.1_bio_30s_RADmax_1kmWW.asc; wc2.1_bio_30s_RADmean_1kmWW.asc; wc2.1_bio_30s_RADcv_1kmWW.asc; wc2.1_bio_30s_RHmin_1kmWW.asc; wc2.1_bio_30s_RHmax_1kmWW.asc; wc2.1_bio_30s_RHmean_1kmWW.asc; wc2.1_bio_30s_RHcv_1kmWW.asc

*Description:* Derived from WorldClim Version 2.1 bioclimatic variables^1^ #1-19. Monthly values for wind speed (wc2.1_30s_wind_01_No1of1_1kmWW.asc - wc2.1_30s_wind_12_No1of1_1kmWW.asc), vapour pressure (wc2.1_30s_vapr_01_No1of1_1kmWW.asc -wc2.1_30s_vapr_12_No1of1_1kmWW.asc), minimum temperature (wc2.1_30s_tmin_01_No1of1_1kmWW.asc - wc2.1_30s_tmin_12_No1of1_1kmWW.asc), maximum temperature (wc2.1_30s_tmax_01_No1of1_1kmWW.asc - wc2.1_30s_tmax_12_No1of1_1kmWW.asc), mean temperature (wc2.1_30s_tavg_01_No1of1_1kmWW.asc - wc2.1_30s_tavg_12_No1of1_1kmWW.asc), solar radiation (wc2.1_30s_srad_01_No1of1_1kmWW.asc - wc2.1_30s_srad_12_No1of1_1kmWW.asc), precipitation (wc2.1_30s_prec_01_No1of1_1kmWW.asc - wc2.1_30s_prec_12_No1of1_1kmWW.asc), and elevation (wc2.1_30s_elev_No1of1_1kmWW.asc) as used by WorldClim were also downloaded.

Radiation variables (wc2.1_bio_30s_RADmin_1kmWW.asc, wc2.1_bio_30s_RADmax_1kmWW.asc, wc2.1_bio_30s_RADmean_1kmWW.asc, wc2.1_bio_30s_RADcv_1kmWW.asc) were created by calculating the maximum, minimum, mean, and cv of radiation from monthly radiation layers in accordance with ANUCLIM^2^ bioclimatic variables. Minimum, maximum, mean, and cv of relative humidity (wc2.1_bio_30s_RHmin_1kmWW.asc, wc2.1_bio_30s_RHmax_1kmWW.asc, wc2.1_bio_30s_RHmean_1kmWW.asc, wc2.1_bio_30s_RHcv_1kmWW.asc) were calculated from monthly values for water vapour pressure, minimum, and maximum temperature: relative humidity was calculated for the lowest and highest temperature of each month (2 values per month or 24 values across the year) based on the ratio of the water vapour pressure to saturation vapour pressure at the relevant temperature (RH = P_w_/P_ws_ x 100%) and summarized across the 24 values.

All layers were resampled from 30 sec (~0.0083 decimal degrees) to 0.01 decimal degrees using a snap raster to achieve consistent resolution and cell line-up with other data layers.

Future climate data from WorldClim 2.1 was sourced for 2041-2060 and 2081-2100 (2050 and 2090 centred). (CMIP6^3^ pathway SSP5-8.5 ‘business as usual’). Future climate for relative humidity and radiation were not available from WorldClim and were instead calculated from change grids sourced from Copernicus^4^ at the same resolution. Seven Global Climate Models (GCMs) were available from both WorldClim and Copernicus: (1) CanESM5-CanOE (Canada); (2) CMCC-ESM2 (Italy); (3) EC-Earth3-Veg-LR (Europe); (4) FIO-ESM-2-0 (China); (5) INM-CM4-8 (Russia); (6) INM-CM5-0 (Russia); (7) MPI-ESM1-2-LR (Germany).

*Relevance:* Climate variables are important abiotic predictors of habitat suitability for most organisms, which usually have an optimum range of temperatures (either ambient air temperature or temperature changes caused by absorption of radiation), ‘dryness’, and precipitation under which they can persist.

**Topography layers based on Amatulli et al. 2018:**

Amat2018_elevmed_WW1km.asc; Amat2018_elevsd_WW1km.asc; cat_Amat2018_geom_WW1km.asc; Amat2018_northn_WW1km.asc; Amat2018_slope_WW1km.asc; Amat2018_tri_WW1km.asc; Amat2018_vrm_WW1km.asc; Amat2018_northmod3_WW1km.asc.gz; Amat2018_aspcos_WW1km.asc

*Description:* Derived from Amatulli et al. 2018^5^, these variables describe median elevation (Amat2018_elevmed_WW1km.asc), several different measures of topographic ruggedness (Amat2018_elevsd_WW1km.asc; Amat2018_tri_WW1km.asc; Amat2018_vrm_WW1km.asc), northness of aspect and slope (Amat2018_northn_WW1km.asc), slope (Amat2018_slope_WW1km.asc), aspect cosine (Amat2018_aspcos_WW1km.asc), and land form (cat_Amat2018_geom_WW1km.asc). Additionally we derived the layer (Amat2018_northmod3_WW1km.asc.gz) by multiplying northness with log10 of latitude *-1 to derive an adjusted layer that describes the extent to which slopes are oriented towards the equator, i.e. the degree to which a slope improves exposure to the sun, which is proportional to northness in the southern hemisphere and to southness in the northern hemisphere. Higher orientation of the aspect towards the equator, higher slope degrees towards the equator, and higher latitude equate to a higher value i.e. a higher increase in sun exposure from these qualities. The log transform allows for values near the equator become 0; at latitudes below 10 they are multiplied by 0-0.99 (reduced) and at latitudes above 10 they ae multiplied by 1-1.95 (emphasized the higher the latitude), i.e. at low latitudes where the sun is almost exactly above the effect of orientation for changes in sun exposure is less severe. Reptiles commonly use basking to increase body temperature, so orientation towards the sun may affect habitat suitability.

Source layers of medians for all variables were used, except for Amat2018_elevsd_WW1km.asc (standard deviation), and cat_Amat2018_geom_WW1km.asc (majority). All layers were resampled from 30 sec (~0.0083 decimal degrees) to 0.01 decimal degrees using a snap raster to achieve consistent resolution and cell line-up with other data layers.

*Relevance:* most macro-organisms can only occur in terrain that fits their mode of dispersal (e.g. flat terrain that is easy to cross), provides shelter (e.g. valleys with low exposure to harsh winds or rugged terrain with caves), and is sufficiently but not overly exposed to radiation (e.g. a certain northness that provides optimal sun exposure to warm up in colder seasons).

**Soil layers based on ISRIC:**

ISRIC_bdod_0-5cm_Med_WW1km.asc; ISRIC_cfvo_0-5cm_Med_WW1km.asc; ISRIC_clay_0-5cm_Med_WW1km.asc; ISRIC_soc_0-5cm_Med_WW1km.asc; cat_ISRIC_MostProbable_WW1km.asc

*Description:* Derived from ISRIC 250m SoilGrids^6-8^. These layers describe bulk density (ISRIC_bdod_0-5cm_Med_WW1km.asc), coarse fragment fraction (ISRIC_cfvo_0-5cm_Med_WW1km.asc), clay percentage (ISRIC_clay_0-5cm_Med_WW1km.asc), soil organic carbon content (ISRIC_soc_0-5cm_Med_WW1km.asc), and most probable Reference Soil Groups (RSG; ISRIC_MostProbable_WW1km.asc) in the top 5cm of soil.

All layers were aggregated from 0.0025 decimal degrees to 0.01 decimal degrees by median using a snap raster for continuous variables and by mode for categorical variables to achieve consistent resolution and cell line-up with other data layers.

*Relevance:* Soil characteristics define many habitat qualities that can restrict organisms. For example, fraction of coarse fragments and bulk density describe substrate structure that an organism will manoeuvre across in their daily activities, clay content and organic carbon content influence many water holding characteristics and finer scale substrate structure and are determinants of vegetation type, and many soil characteristic influence thermal soil properties in conjunction with air temperature and radiation.

**Lithology layers based on GLiM :**

cat_GLIM_DomlithFill_2015_WW1km.asc

*Description:* derived from the Global Lithology map database^9^. This layer describes the 16 main lithological classes of the Earth’s surface. The layer was rasterized at 0.01 decimal degrees by majority using a snap raster to achieve consistent resolution and cell line-up with other data layers.

*Relevance:* the lithology of the land surfaces influences vegetation communities, soil depth and structure, and land form. Many organisms have affinities to certain substrates, such as sandstone or volcanic rock (e.g. some lizards are adapted specifically to limestone outcrops by matching their body colour and climbing abilities to the substrate).

**Vegetation layers based on Copernicus time series:**

FAPAR_nn_cv_2010_2019FILL.asc; FAPAR_nn_range_2010_2019FILL.asc; FAPAR_nn_min_2010_2019FILL.asc; FAPAR_nn_mean_2010_2019FILL.asc; FAPAR_nn_max_2010_2019FILL.asc;

DMP_nn_cv_2014_2019FILL.asc; DMP_nn_range_2014_2019FILL.asc; DMP_nn_min_2014_2019FILL.asc; DMP_nn_mean_2014_2019FILL.asc; DMP_nn_max_2014_2019FILL.asc;

*Description:* derived from 10-daily time series of remotely sensed information on fraction photosynthetic active radiation (FAPAR; vegetation “greenness”)^10^, and dry matter productivity (DMP, vegetation “growth rate”)^11^. Layers were summarized by minimum, mean, maximum, coefficient of variation (=seasonality; “cv”), and range (maximum-minimum) across all available layers for each year. These preliminary yearly summary layers were then summarized as mean minimum, mean mean, mean maximum, mean cv, and mean range across all available years. Complete data was available for years 2010-2019 for FAPAR V2 and 2014-2019 for DMP V2 at time of download (2020).

All layers were resampled from ~1km to 0.01 decimal degrees using a snap raster to achieve consistent resolution and cell line-up with other data layers.

*Relevance:* Vegetation greenness and growth rate are important vegetation characteristics that may relate to whether the habitat is used by a certain species or not. For example, some species will only occur in dry savannahs with low greenness but substantial annual dry matter production from grasses, while other species are arboreal and only occur in thick, green rainforests.

**Land use layers based on ESA landcover:**

cat_ESA_Landcover_1992_NEW_WW1km.asc; cat_ESA_Landcover_2018_NEW_WW1km.asc; ESA_disbarebiX_WW1km.asc; ESA_discropbiX_WW1km.asc.gz; ESA_dismosbiX_WW1km.asc; ESA_distreebiX_WW1km.asc; ESA_disvegbiX_WW1km.asc; ESA_disurbbiX_WW1km.asc; cat_ESA_lusimplef_2018_WW1km.asc

*Description:* derived from the ~250m land cover classification from the European space agency (ESA) for 1992 (earliest available; cat_NEW_ESA_Landcover_1992_WW1km.asc) and 2018 (most recent available at time of access; cat_NEW_ESA_Landcover_2018_WW1km.asc)^12^. These layers describe the two most distant available time steps for land cover and represent the variation in land cover for each pixel over that time period. Land cover classes include vegetation classification of native vegetation, agricultural land use classes of crop land and mosaic crop land, and other land uses such as water areas, bare areas, and urban areas. Layers were aggregated to 0.01 decimal degrees using a snap raster to achieve consistent resolution and cell line-up with other data layers.

The 2018 layer was additionally simplified to describe broad land use categories (forests, short vegetation, bare areas, crop land, mosaic crop land, urban areas, and other; ESA_lusimplef_WW1km.asc). Additional layers were created that describe the Euclidean distance from any grid cell that contains each of the broad categories described above (ESA_disbarebiX_WW1km.asc; ESA_discropbiX_WW1km.asc.gz; ESA_dismosbiX_WW1km.asc; ESA_distreebiX_WW1km.asc; ESA_disvegbiX_WW1km.asc; ESA_disurbbiX_WW1km.asc). Areas where the relevant category of land cover was present (i.e. where Euclidean distance was initially calculated as 0) were further reclassified as (i) areas where land class is present but does not represent the majority of 250m cells in the 1km cell (=fragmented conditions; 0), (ii) areas where the land class represents the majority but which are within 2 grid cells of cells classed as ‘0’(=edge of core conditions; -0.01 to -0.02), and (iii) areas where the land class represents the majority and which are more than 2 grid cells from cells classed as ‘0’ (=core conditions; -0.03). These classes represent a ‘reverse’ distance measure of cells that would all show up as ‘0’ in a standard Euclidean distance layer, to distinguish between e.g. forest that is fragmented, and the edge of a solid patch of forest or within a larger solid patch of forest.

*Relevance:* land cover or distance from suitable land cover defines whether an organism has access to its preferred habitat. E.g. some species are savannah species, some are capable to co-occur with humans in anthropogenic landscapes, while others prefer pristine forest habitats or bare areas such as sand dunes.

**Human Impact layers based on WorldPop:**

WorldPop_peoplepercell_WW1km.asc

Description: derived from the WorldPop data set of people per 30 second grid cell^13^. This layer was resampled from 30 sec (~0.0083 decimal degrees) to 0.01 decimal degrees using a snap raster to achieve consistent resolution and cell line-up with other data layers.

*Relevance*: many organisms only occur in environments up to a certain level of anthropogenic influence. For example some snakes are known to occur in natural and semi-disturbed environments but will only rarely disperse into cities or other areas with high population density. Intermediate levels of population density can also increase numbers of rodents and other easy prey items for snakes and increase snake numbers.

**Water layers based on WWF HydroATLAS, HydroLAKES, and ESA landcover:**

ESA_HA_HL_permwatdist_WW1km.asc; ESA_HA_HL_freshwatdist_WW1km.asc

*Description:* these layers are derived from a combination of WWF HydroSHEDS products^14-18^ and ESA land cover products^12^. The layers show Euclidean distance from any freshwater features (including very small creeks, etc.) and from permanent freshwater features. “Any freshwater features” are a combination of stream segments with a total annual runoff of at least 1m^3^/sec from HydroATLAS, permanent lakes and reservoirs from HydroLAKES, and grid cells classified as ‘water’ by ESA. ‘Permanent’ freshwater is defined as any HydroATLAS river segments with a minimum month runoff of 1m^3^/sec (i.e. runoff exists in the driest month), as well as HydroLAKES lakes and reservoirs and ESA water areas. Vector data was rasterized before calculating Euclidean distance at 0.01 decimal degrees using a snap raster to achieve consistent resolution and cell line-up with other data layers.

*Relevance:* many organisms only occur close to water or within certain travel times of water, especially during drier times of the year because open water is often crucial for survival. Some organisms are also aquatic or semi aquatic.

**References:**

1 Fick, S. E. & Hijmans, R. J. WorldClim 2: new 1‐km spatial resolution climate surfaces for global land areas (<https://www.worldclim.org/data/worldclim21.html>). *International journal of climatology* **37**, 4302-4315 (2017).

2 Xu, T. & Hutchinson, M. ANUCLIM version 6.1 user guide. *The Australian National University, Fenner School of Environment and Society, Canberra* **90** (2011).

3 Eyring, V. *et al.* Overview of the Coupled Model Intercomparison Project Phase 6 (CMIP6) experimental design and organization. *Geoscientific Model Development* **9**, 1937-1958 (2016).

4 Copernicus. Climate Change Service, Climate Data Store: CMIP6 climate projections. Copernicus Climate Change Service (C3S) Climate Data Store (CDS). DOI: 10.24381/cds.c866074c. (2021).

5 Amatulli, G. *et al.* A suite of global, cross-scale topographic variables for environmental and biodiversity modeling. *Scientific data* **5**, 1-15 (2018).

6 Batjes, N. H., Ribeiro, E. & Van Oostrum, A. Standardised soil profile data to support global mapping and modelling (WoSIS snapshot 2019). *Earth System Science Data* **12**, 299-320 (2020).

7 de Sousa, L. M. *et al.* SoilGrids 2.0: producing quality-assessed soil information for the globe. *Soil discussions* **2020**, 1-37 (2020).

8 Poggio, L. *et al.* SoilGrids 2.0: producing soil information for the globe with quantified spatial uncertainty (<https://soilgrids.org/>). *Soil* **7**, 217-240 (2021).

9 Hartmann, J. & Moosdorf, N. The new global lithological map database GLiM: A representation of rock properties at the Earth surface. *Geochemistry, Geophysics, Geosystems* **13** (2012).

10 Copernicus. Land Monitoring Service: Fraction of Absorbed Photosynthetically Active Radiation 1999-2020 (raster 1 km), global, 10-daily – version 2 (<https://land.copernicus.eu/en/products/vegetation/fraction-of-absorbed-photosynthetically-active-radiation-v2-0-1km>). (2020).

11 Copernicus. Land Monitoring Service: Dry Matter Productivity 1999-2020 (raster 1 km), global, 10-daily – version 2 (<https://land.copernicus.eu/en/products/vegetation/dry-matter-productivity-v2-0-1km>). (2020).

12 Copernicus. Climate Change Service, Climate Data Store; Land cover classification gridded maps from 1992 to present derived from satellite observation. Copernicus Climate Change Service (C3S) Climate Data Store (CDS). DOI: 10.24381/cds.006f2c9a. (2019).

13 WorldPop. Estimated total number of people per grid-cell unconstrained global mosaic at 1 km resolution for 2020 - School of Geography and Environmental Science, University of Southampton; Department of Geography and Geosciences, University of Louisville; Departement de Geographie, Universite de Namur) and Center for International Earth Science Information Network (CIESIN), Columbia University. Global High Resolution Population Denominators Project - Funded by The Bill and Melinda Gates Foundation (OPP1134076). <https://dx.doi.org/10.5258/SOTON/WP00647> (2020).

14 Keller, C. *et al.* The new hydrographic HydroSHEDS database derived from the TanDEM-X DEM (<https://www.hydrosheds.org/>). (2023).

15 Lehner, B., Messager, M. L., Korver, M. C. & Linke, S. Global hydro-environmental lake characteristics at high spatial resolution. *Scientific Data* **9**, 351 (2022).

16 Lehner, B. *et al.* in *EGU General Assembly Conference Abstracts.* EGU21-9277.

17 Linke, S. *et al.* Global hydro-environmental sub-basin and river reach characteristics at high spatial resolution. *Scientific data* **6**, 283 (2019).

18 Wickel, B., Lehner, B. & Sindorf, N. in *AGU Fall Meeting Abstracts.* H11H-05.
