# Supplementary figures and images for: Climate change induced complex shifts in snake distributions expose people to snakebite and threaten biodiversity (part 2 of 4)
Source: PLoS Negl Trop Dis. 2026 May 21;20(5):e0014030. doi: 10.1371/journal.pntd.0014030 (PMC13193456; doi:10.1371/journal.pntd.0014030)

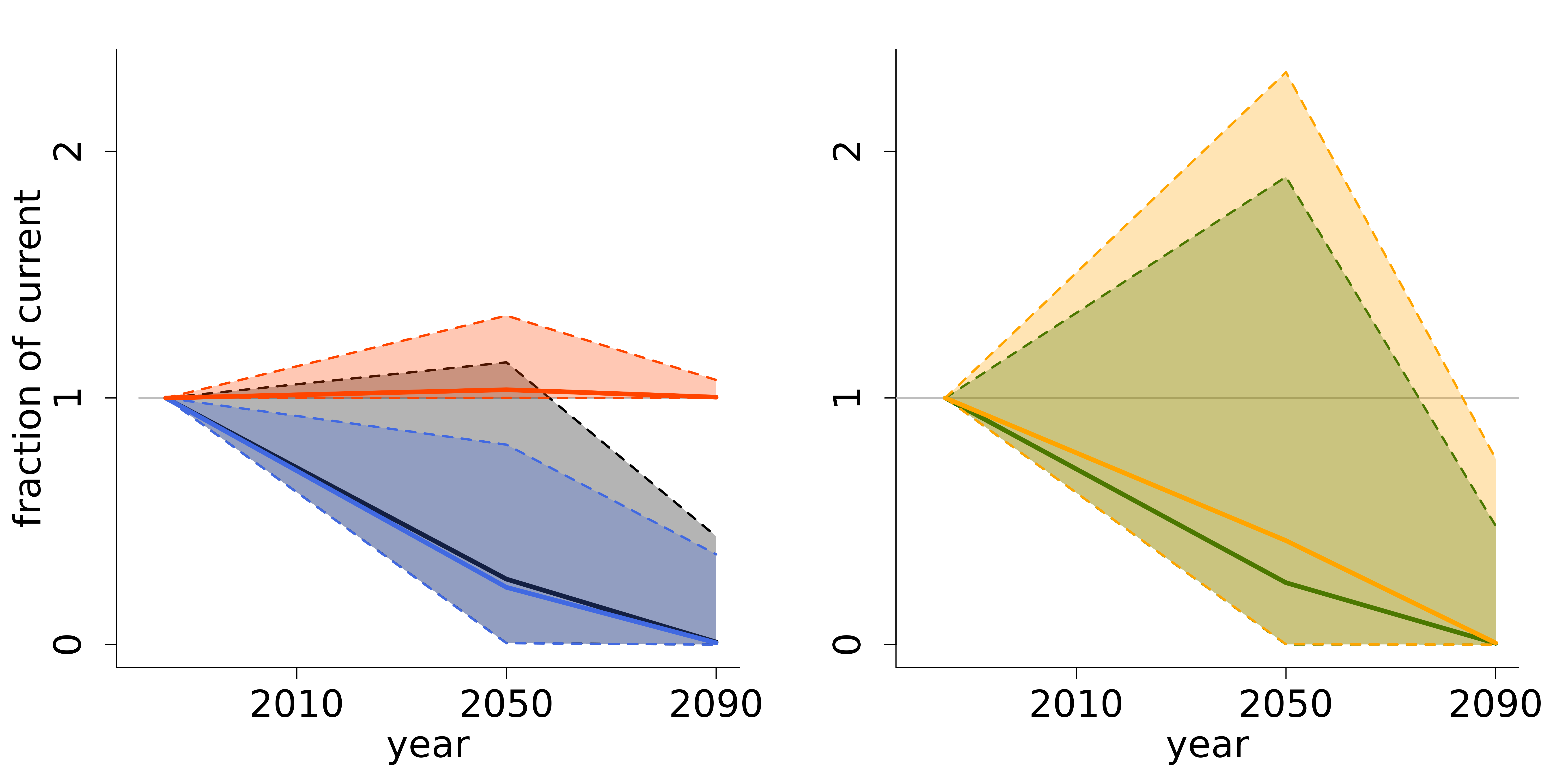

Supplement: S2 Appendix — (ZIP) [file pntd.0014030.s006.zip › Sup. Mat. 6-1 A-L - Species Trends/Bothrops_pauloensis_CCTrends.png]

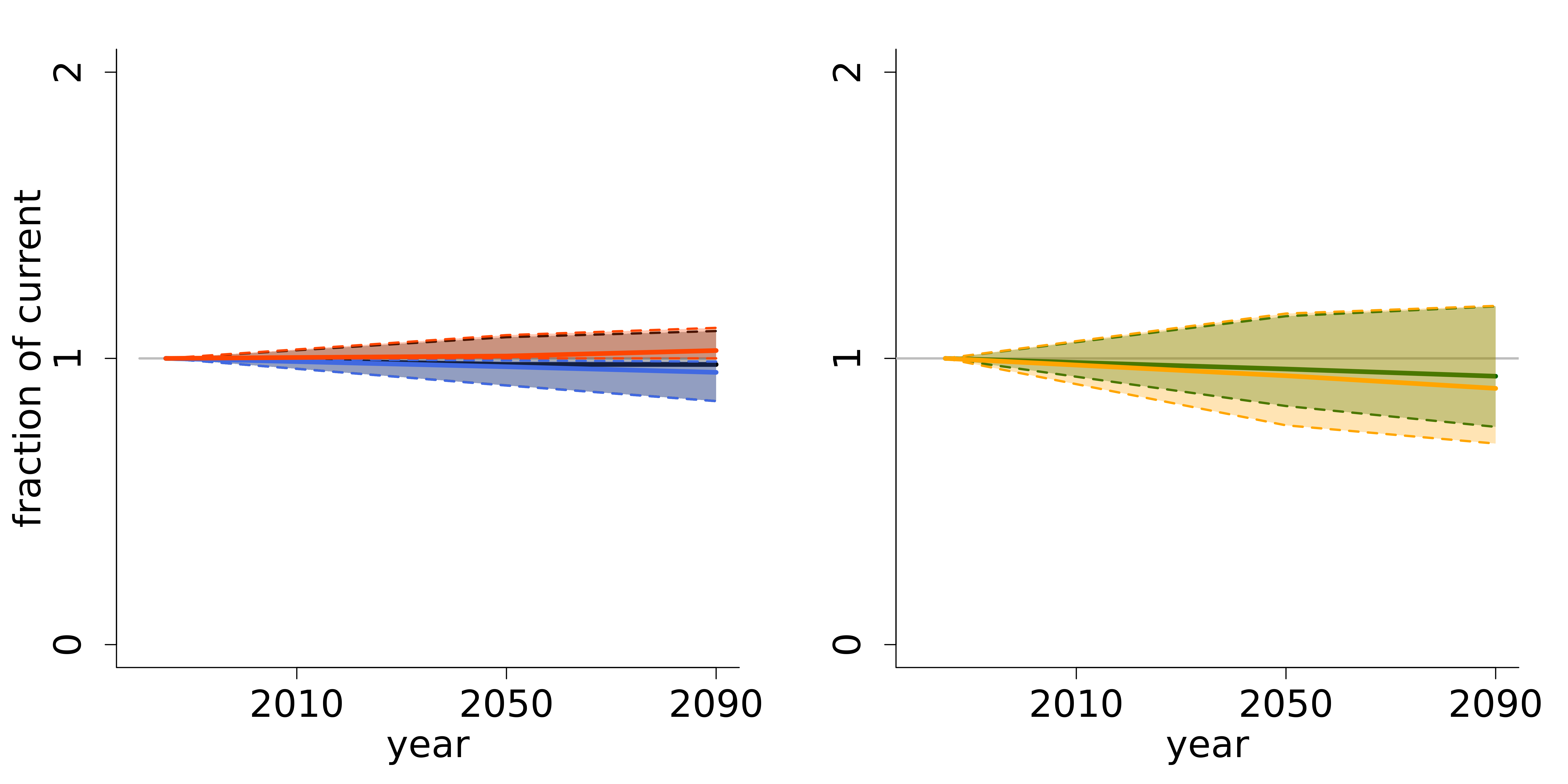

Supplement: S2 Appendix — (ZIP) [file pntd.0014030.s006.zip › Sup. Mat. 6-1 A-L - Species Trends/Bothrops_pictus_CCTrends.png]

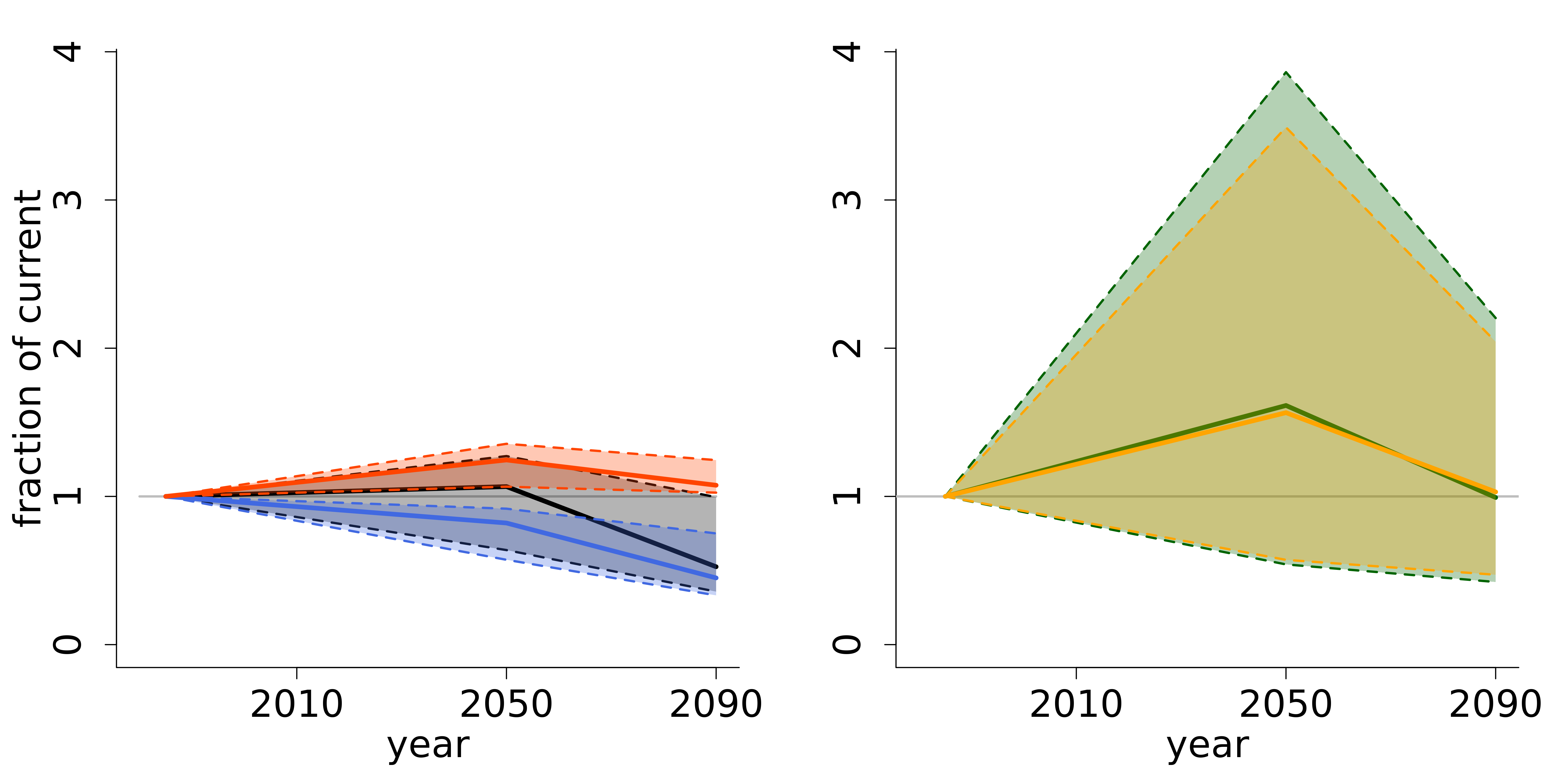

Supplement: S2 Appendix — (ZIP) [file pntd.0014030.s006.zip › Sup. Mat. 6-1 A-L - Species Trends/Bothrops_pirajai_CCTrends.png]

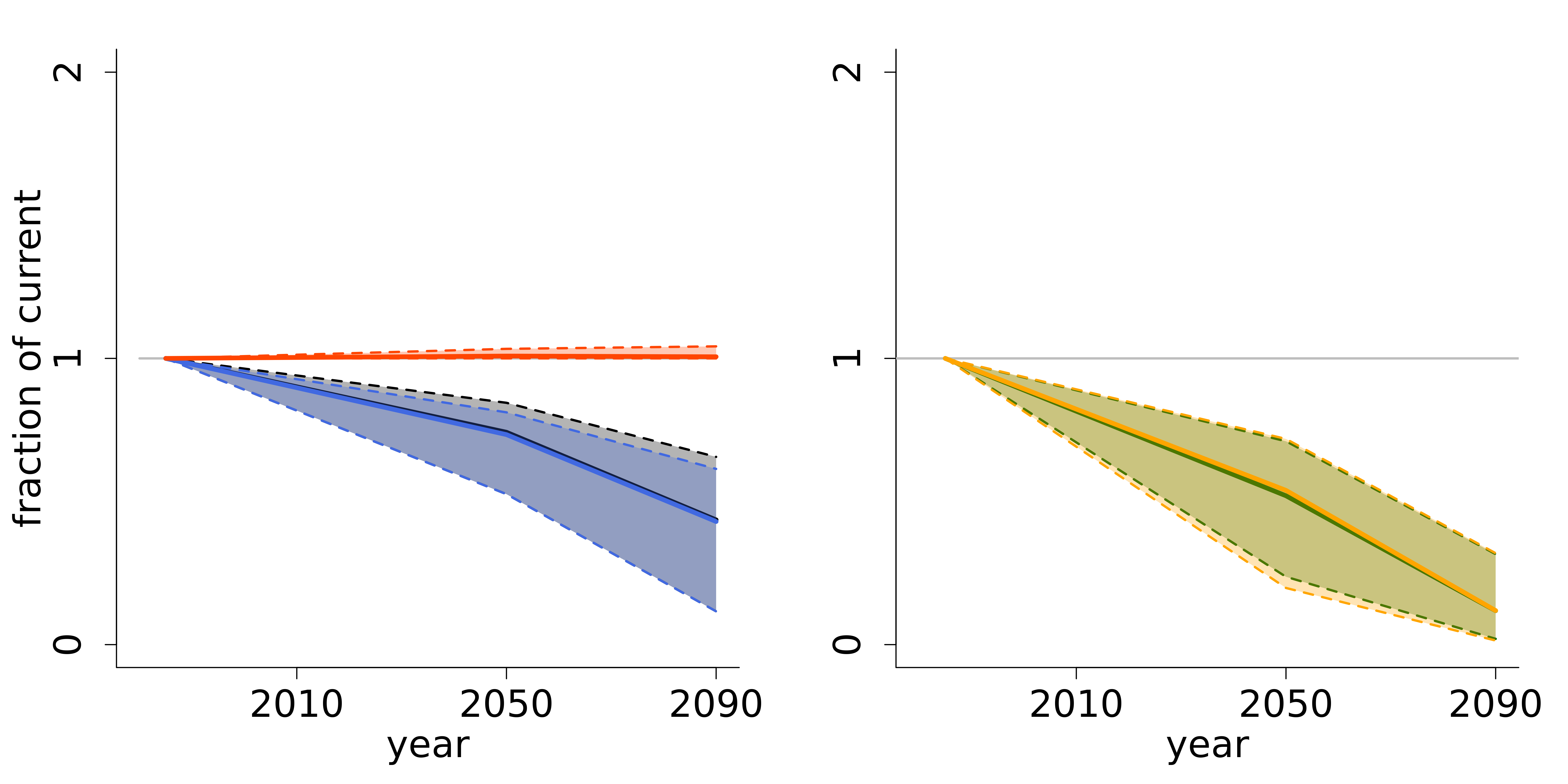

Supplement: S2 Appendix — (ZIP) [file pntd.0014030.s006.zip › Sup. Mat. 6-1 A-L - Species Trends/Bothrops_pubescens_CCTrends.png]

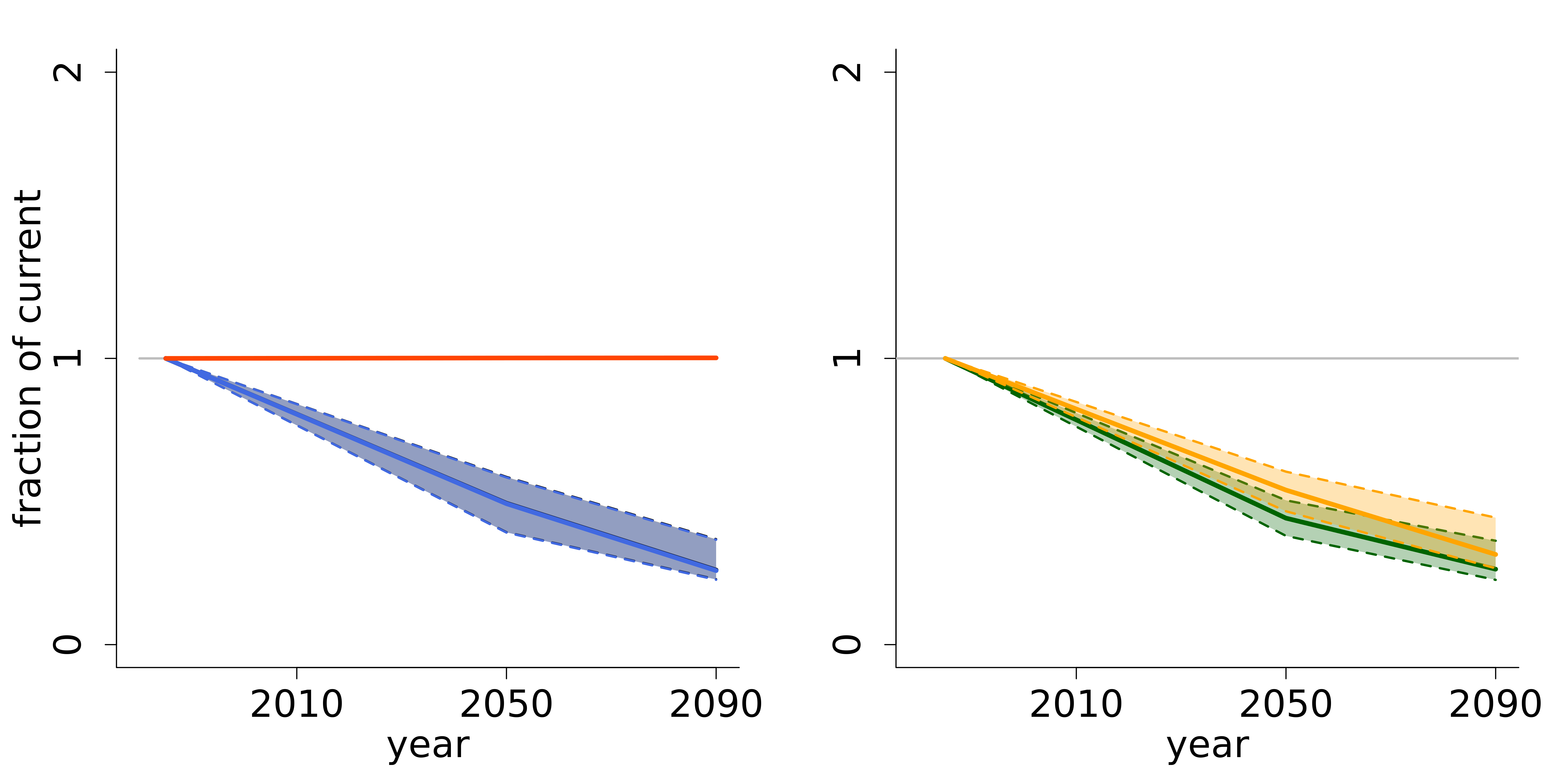

Supplement: S2 Appendix — (ZIP) [file pntd.0014030.s006.zip › Sup. Mat. 6-1 A-L - Species Trends/Bothrops_pulcher_CCTrends.png]

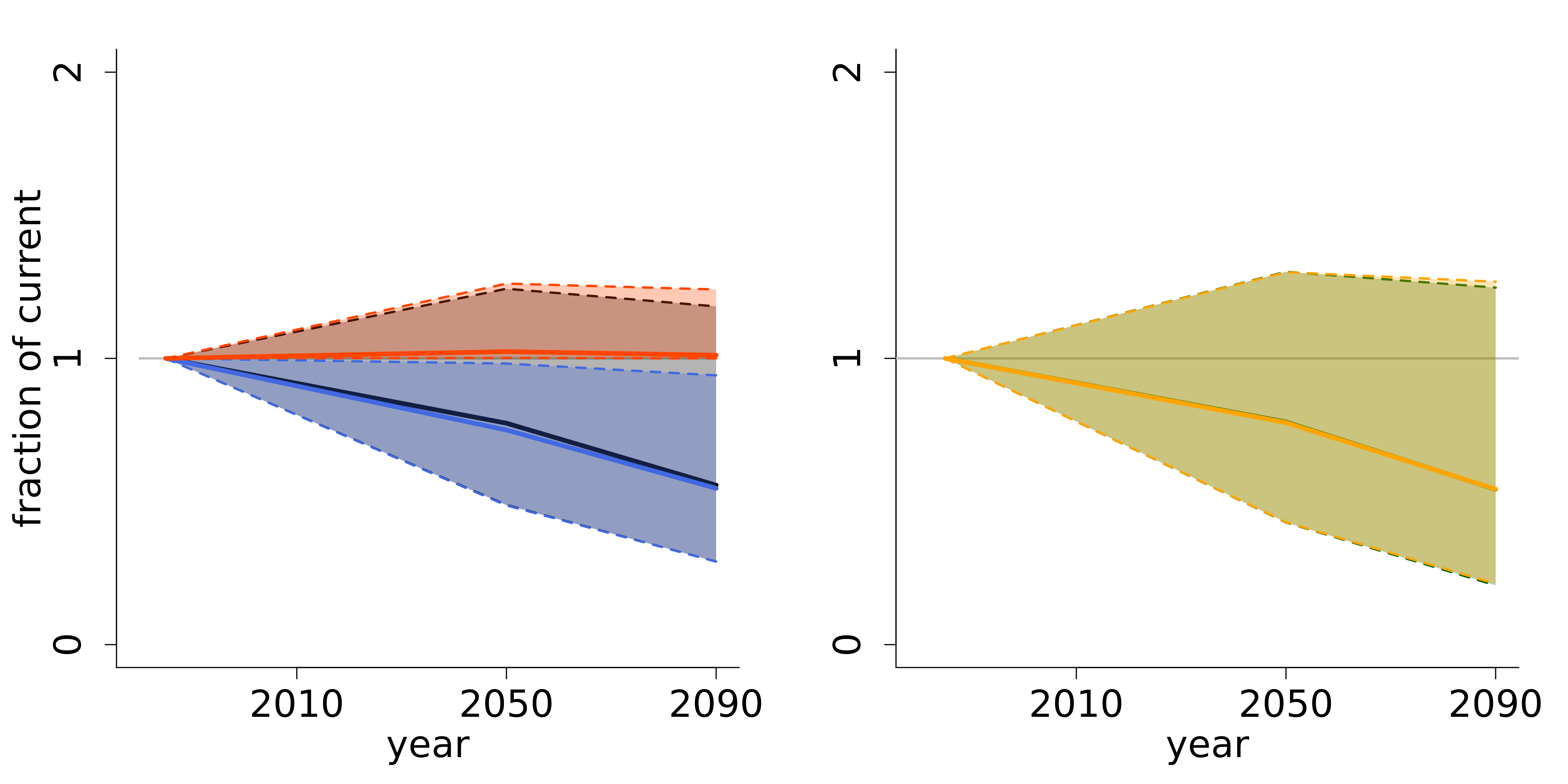

Supplement: S2 Appendix — (ZIP) [file pntd.0014030.s006.zip › Sup. Mat. 6-1 A-L - Species Trends/Bothrops_punctatus_CCTrends.png]

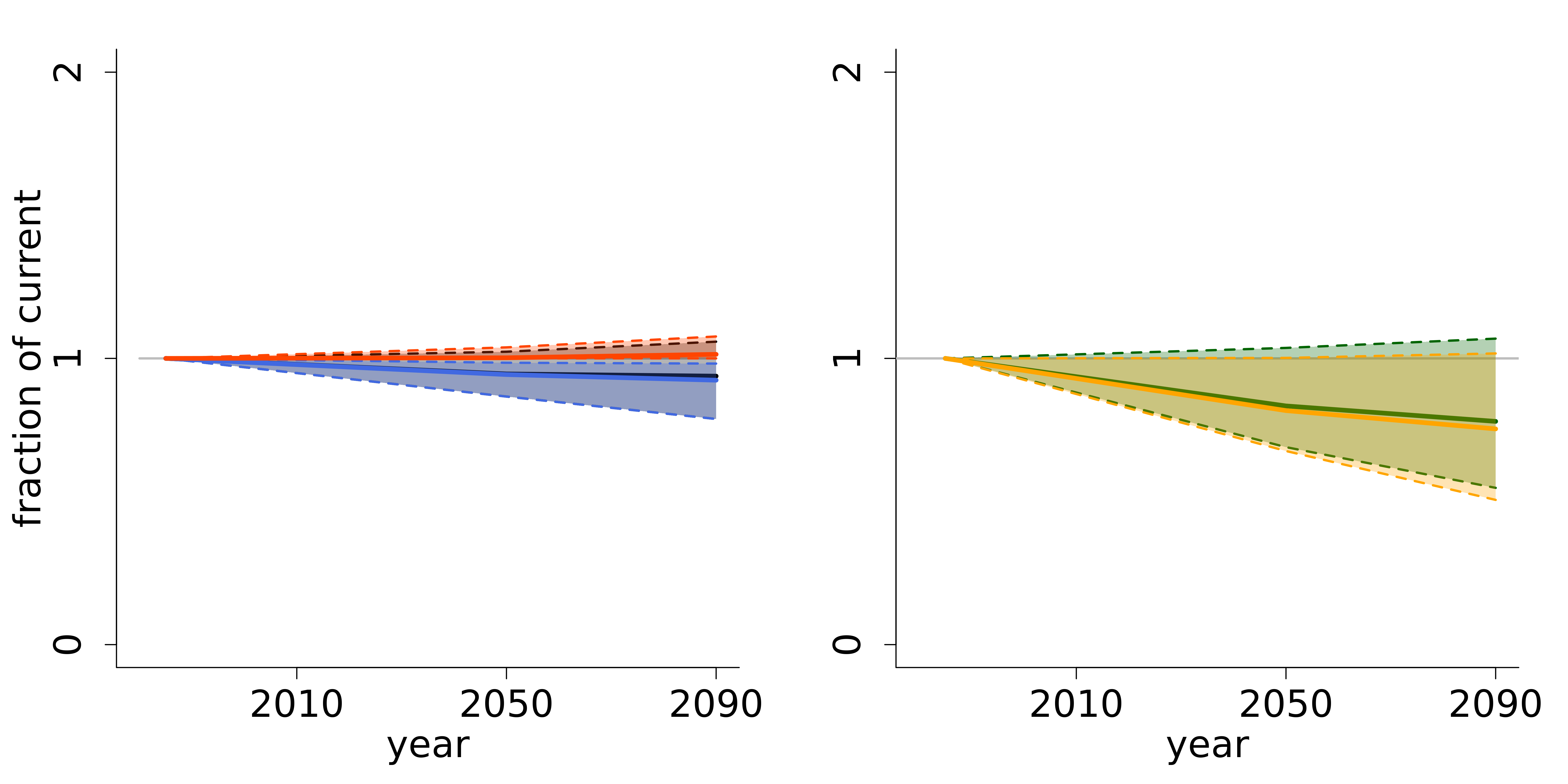

Supplement: S2 Appendix — (ZIP) [file pntd.0014030.s006.zip › Sup. Mat. 6-1 A-L - Species Trends/Bothrops_sanctaecrucis_CCTrends.png]

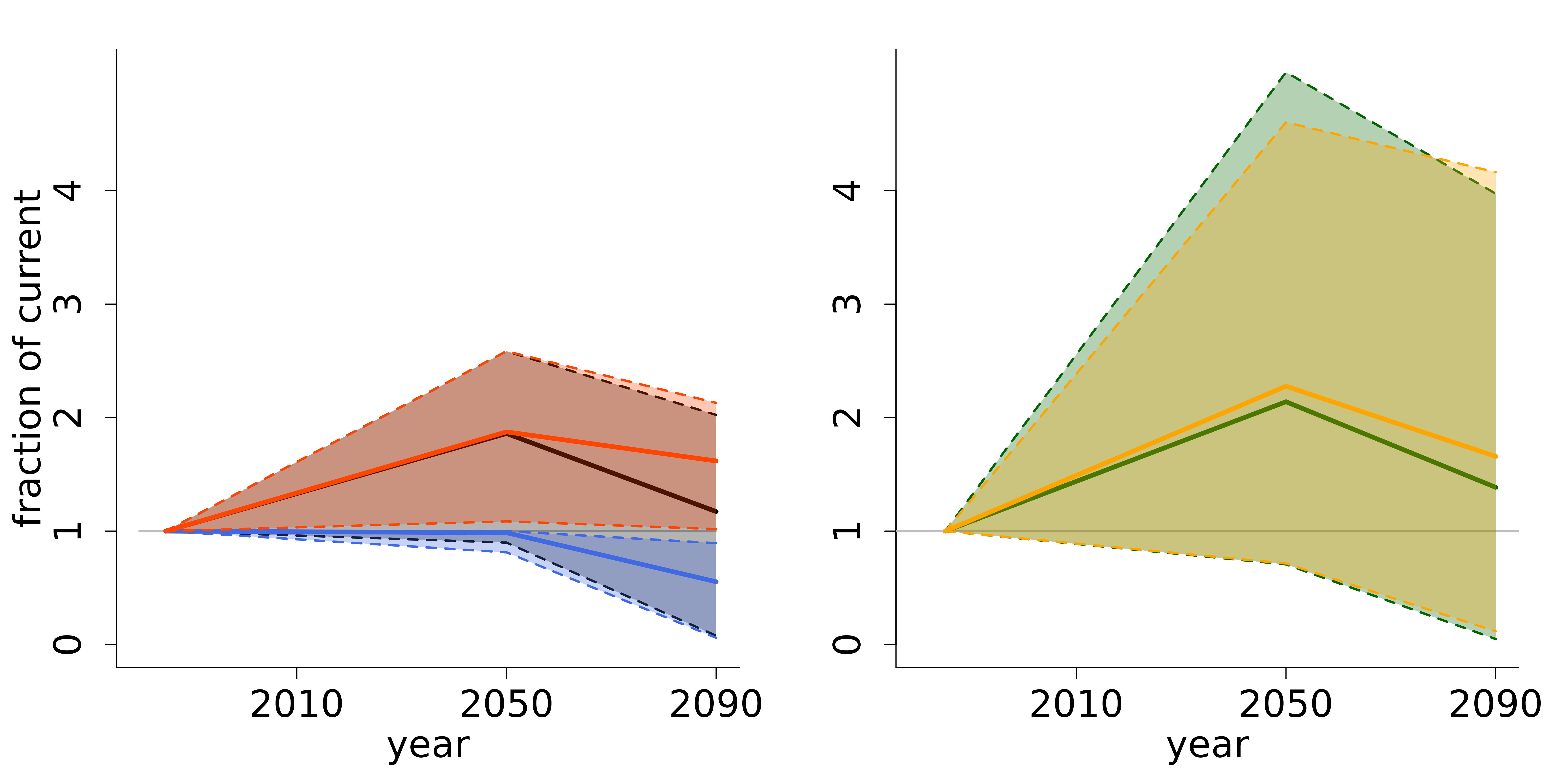

Supplement: S2 Appendix — (ZIP) [file pntd.0014030.s006.zip › Sup. Mat. 6-1 A-L - Species Trends/Bothrops_sonene_CCTrends.png]

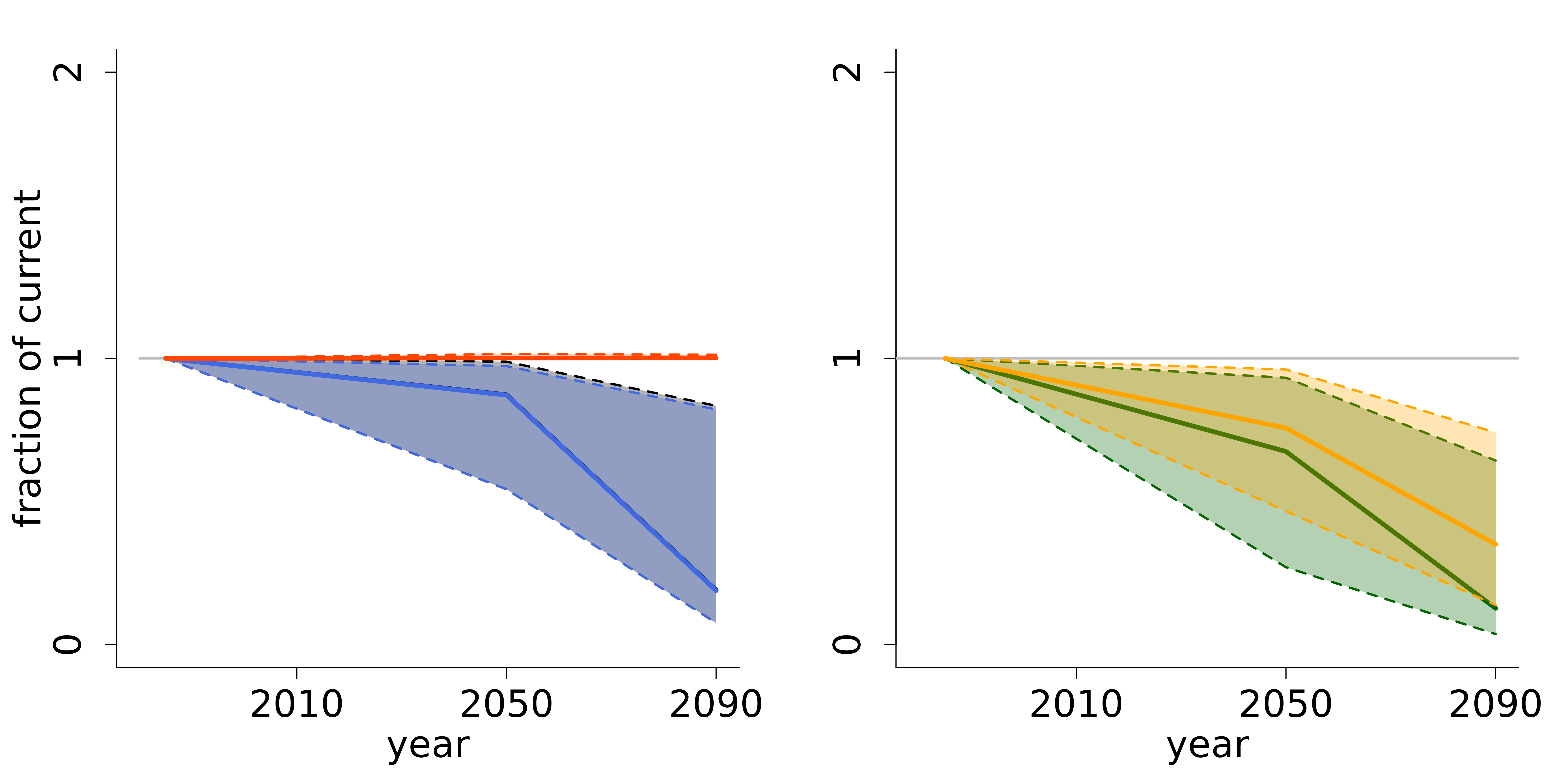

Supplement: S2 Appendix — (ZIP) [file pntd.0014030.s006.zip › Sup. Mat. 6-1 A-L - Species Trends/Bothrops_taeniatus_CCTrends.png]

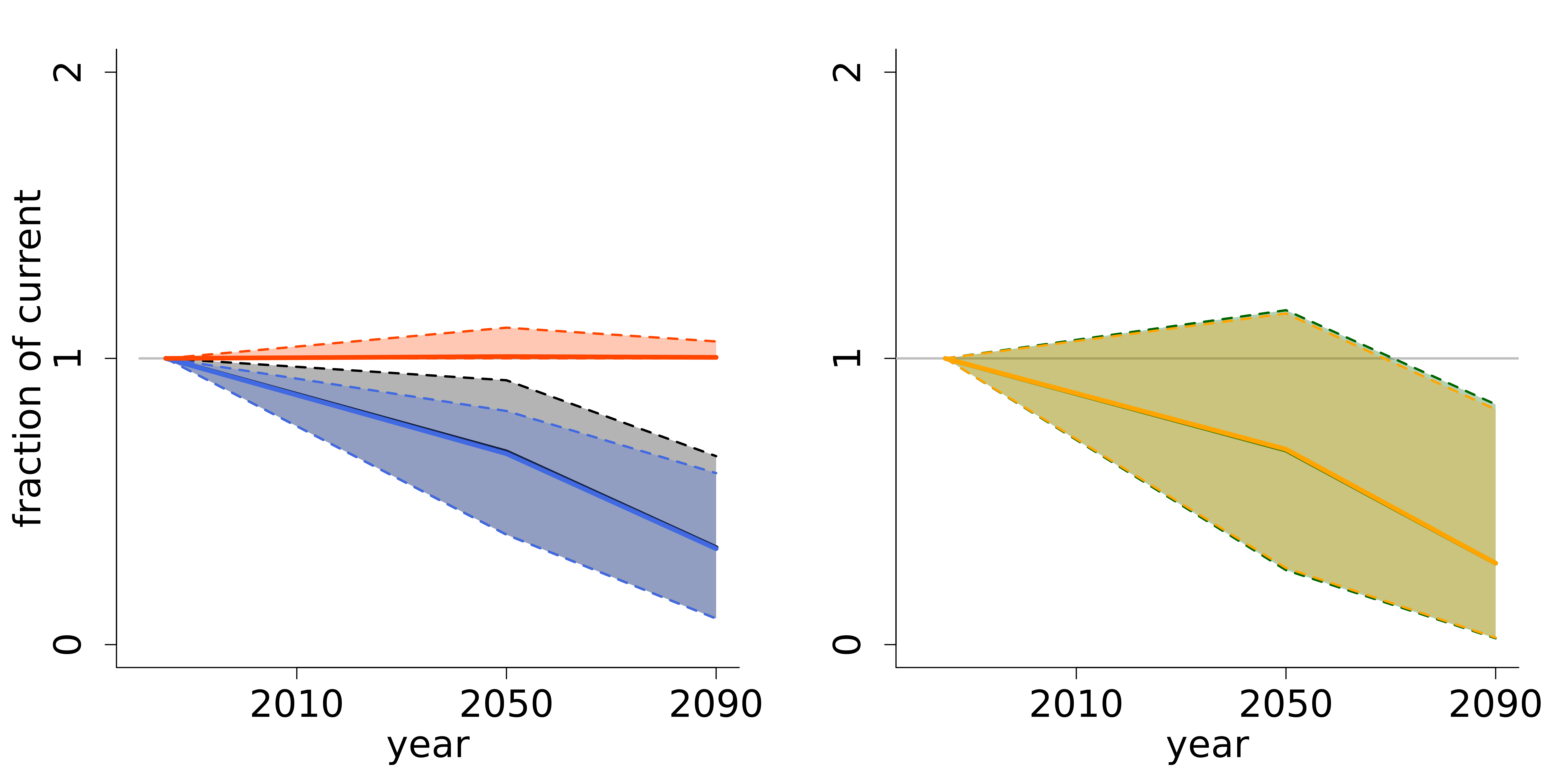

Supplement: S2 Appendix — (ZIP) [file pntd.0014030.s006.zip › Sup. Mat. 6-1 A-L - Species Trends/Bothrops_venezuelensis_CCTrends.png]

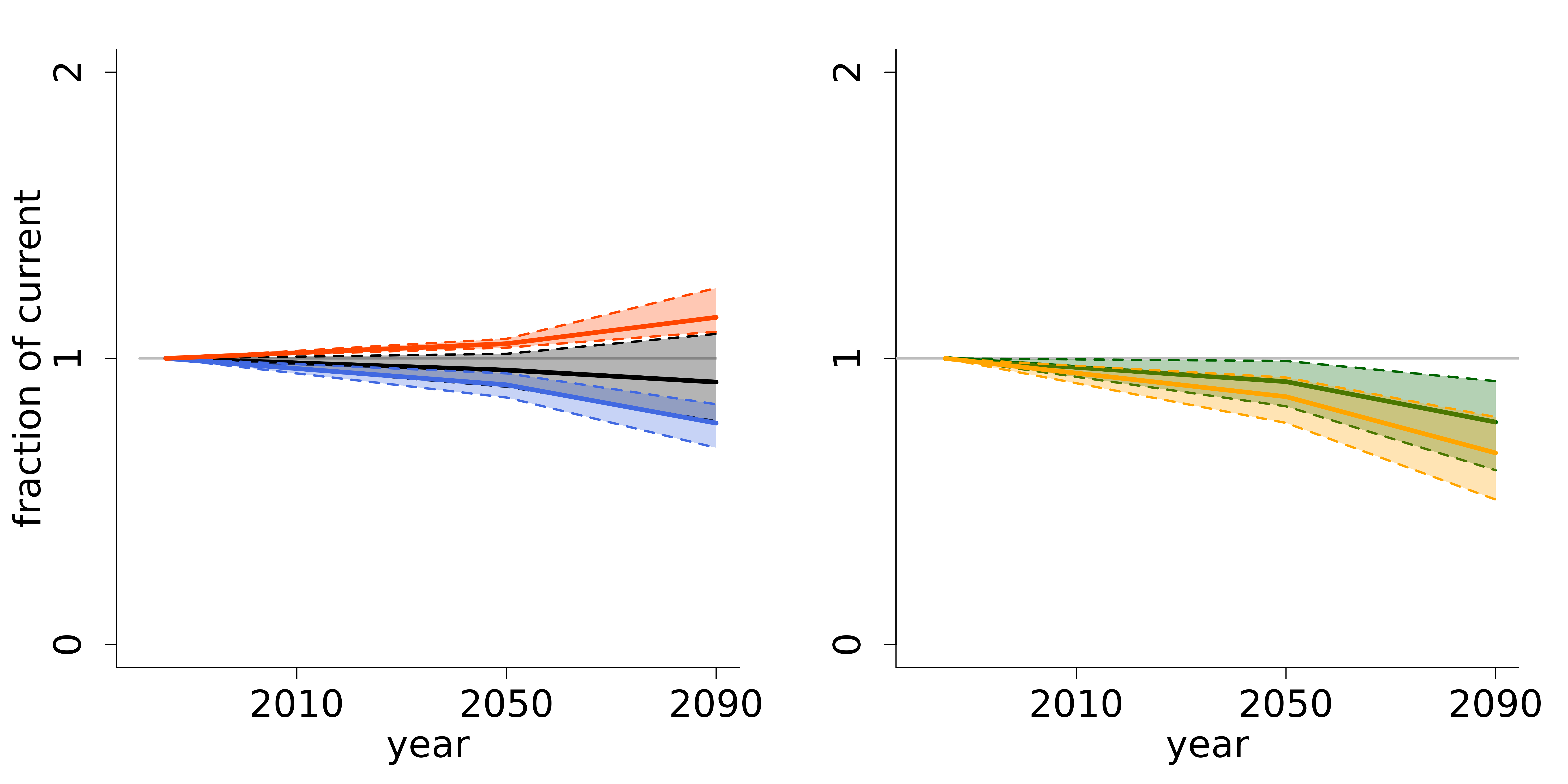

Supplement: S2 Appendix — (ZIP) [file pntd.0014030.s006.zip › Sup. Mat. 6-1 A-L - Species Trends/Bungarus_bungaroides_CCTrends.png]

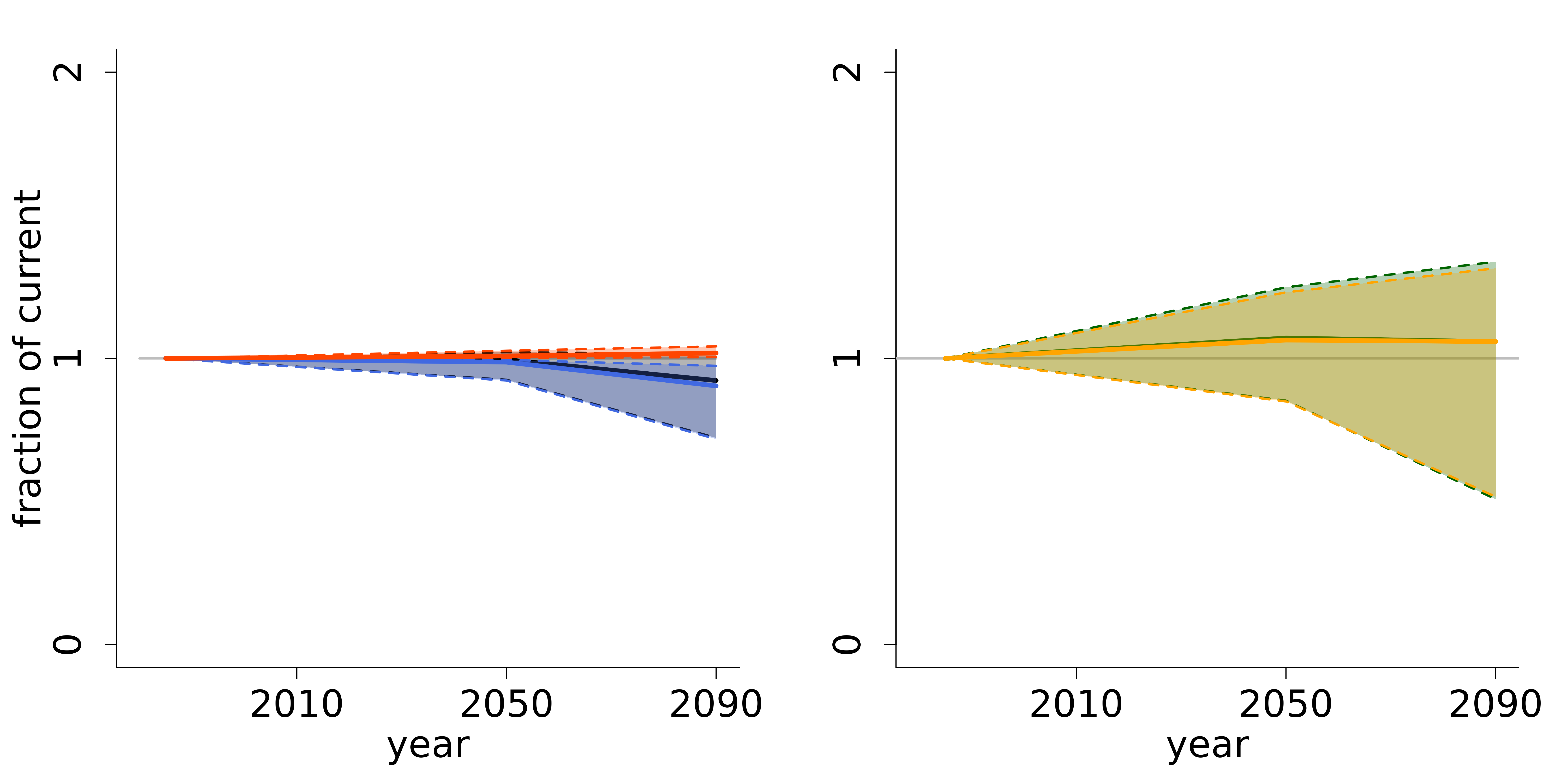

Supplement: S2 Appendix — (ZIP) [file pntd.0014030.s006.zip › Sup. Mat. 6-1 A-L - Species Trends/Bungarus_caeruleus_CCTrends.png]

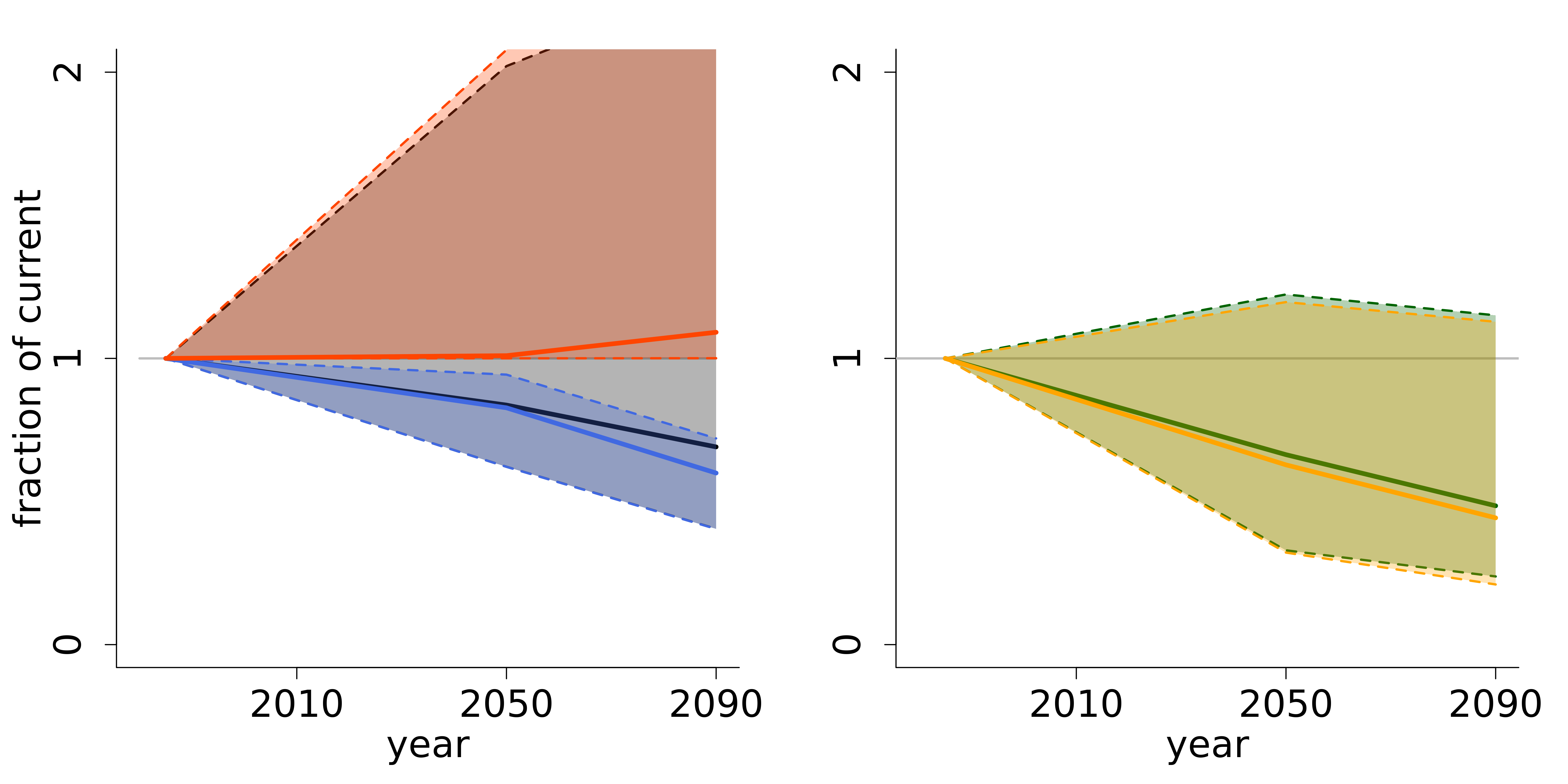

Supplement: S2 Appendix — (ZIP) [file pntd.0014030.s006.zip › Sup. Mat. 6-1 A-L - Species Trends/Bungarus_candidus_CCTrends.png]

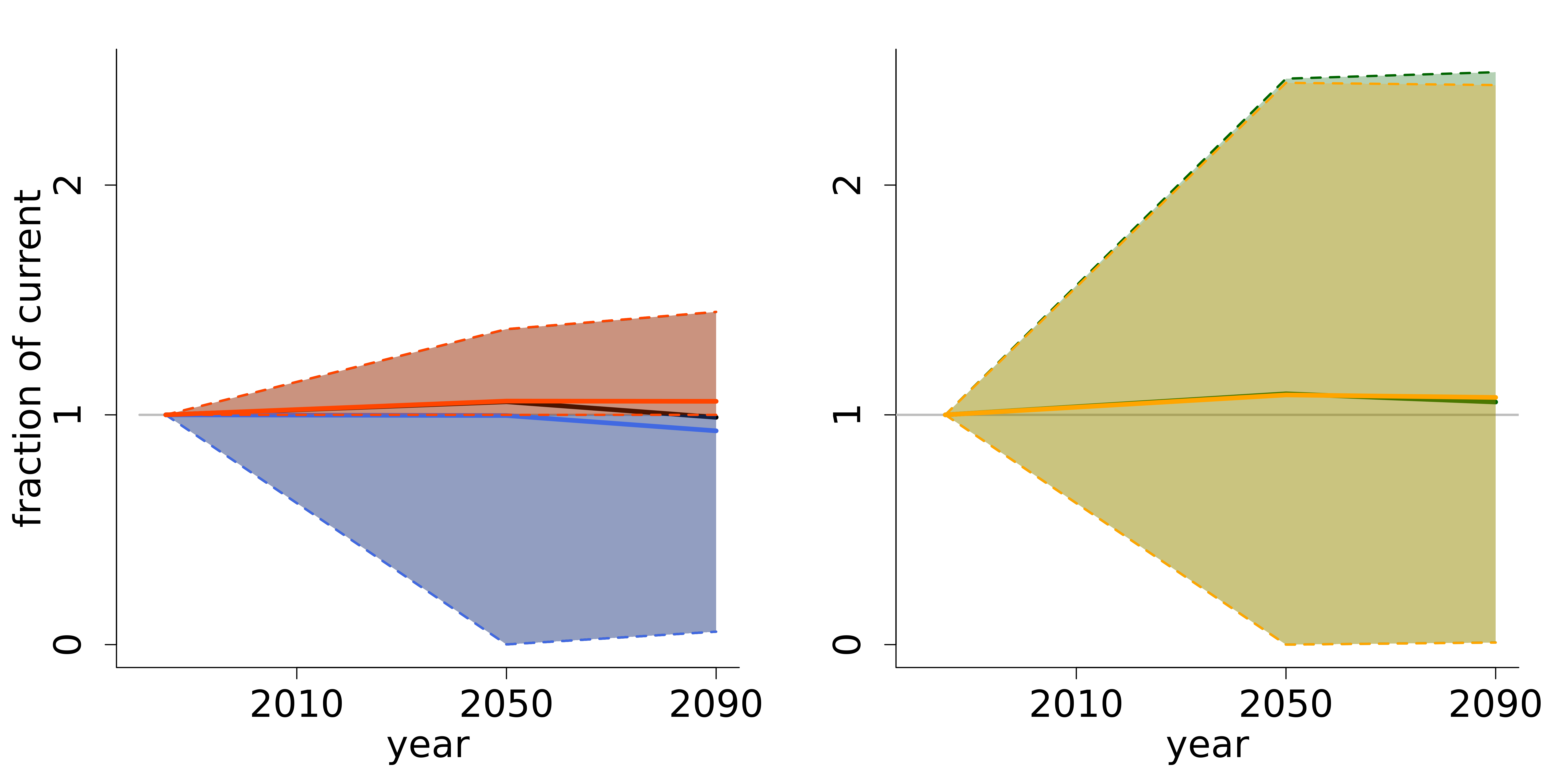

Supplement: S2 Appendix — (ZIP) [file pntd.0014030.s006.zip › Sup. Mat. 6-1 A-L - Species Trends/Bungarus_ceylonicus_CCTrends.png]

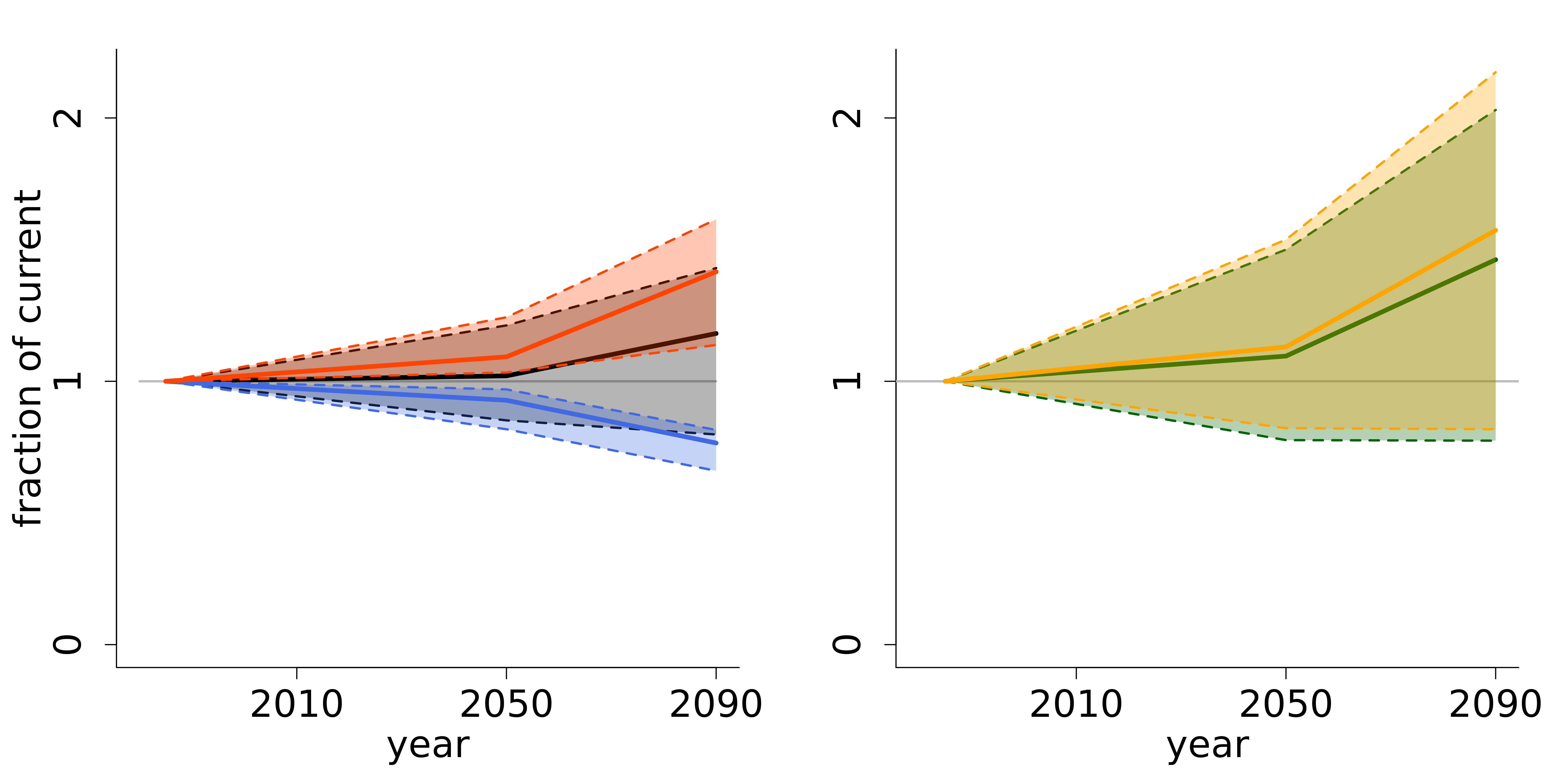

Supplement: S2 Appendix — (ZIP) [file pntd.0014030.s006.zip › Sup. Mat. 6-1 A-L - Species Trends/Bungarus_fasciatus_CCTrends.png]

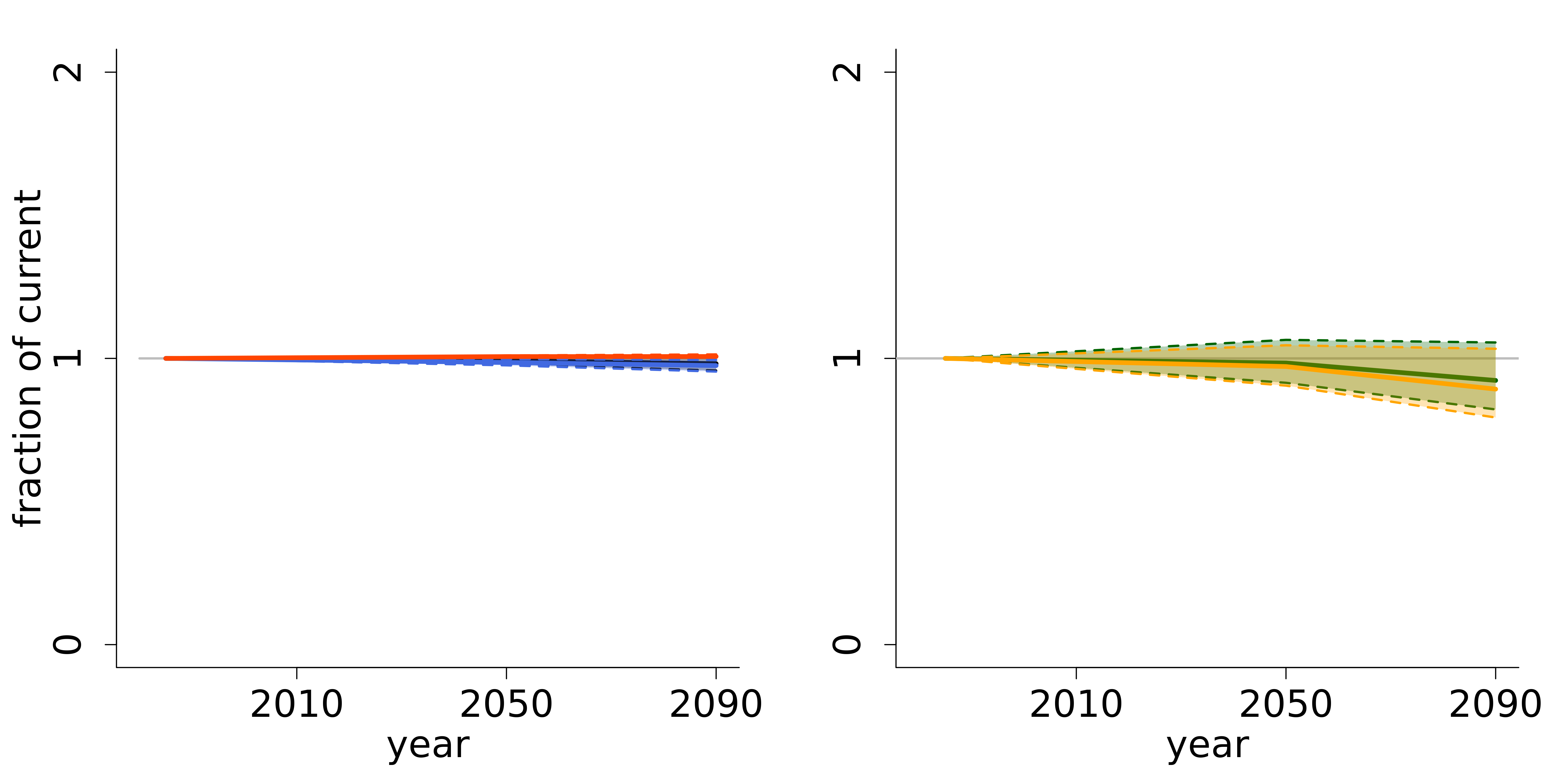

Supplement: S2 Appendix — (ZIP) [file pntd.0014030.s006.zip › Sup. Mat. 6-1 A-L - Species Trends/Bungarus_flaviceps_CCTrends.png]

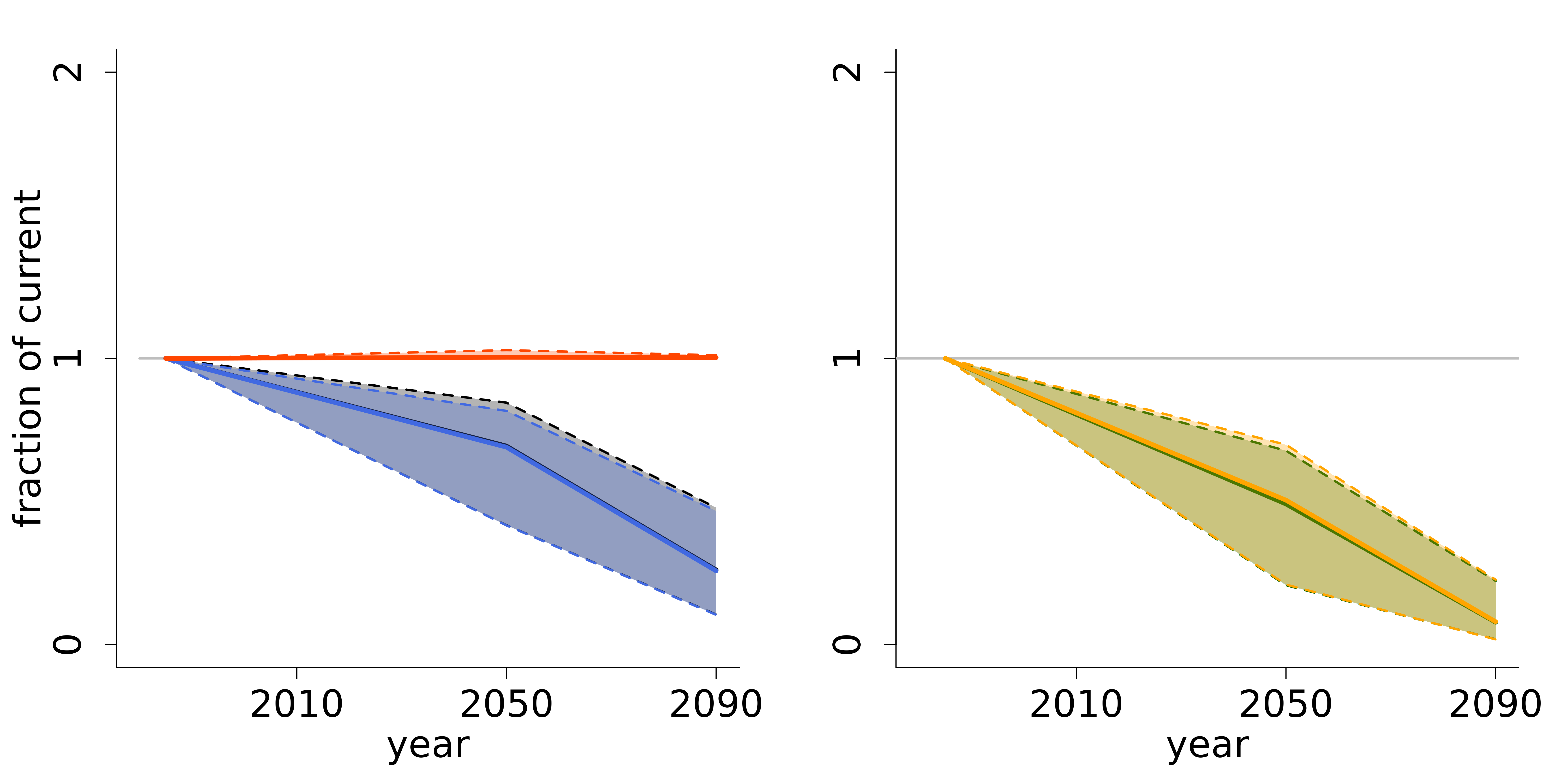

Supplement: S2 Appendix — (ZIP) [file pntd.0014030.s006.zip › Sup. Mat. 6-1 A-L - Species Trends/Bungarus_lividus_CCTrends.png]

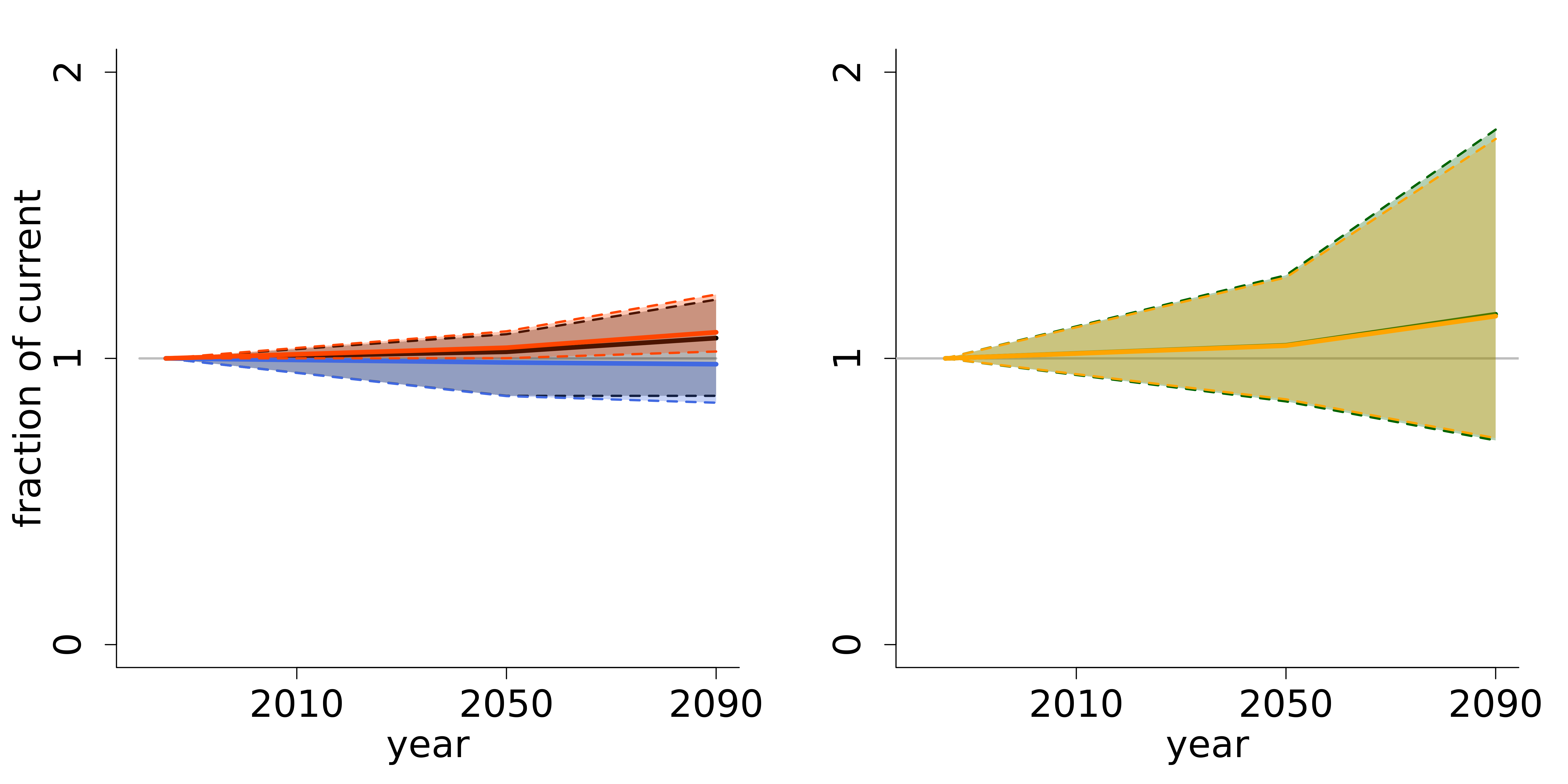

Supplement: S2 Appendix — (ZIP) [file pntd.0014030.s006.zip › Sup. Mat. 6-1 A-L - Species Trends/Bungarus_magnimaculatus_CCTrends.png]

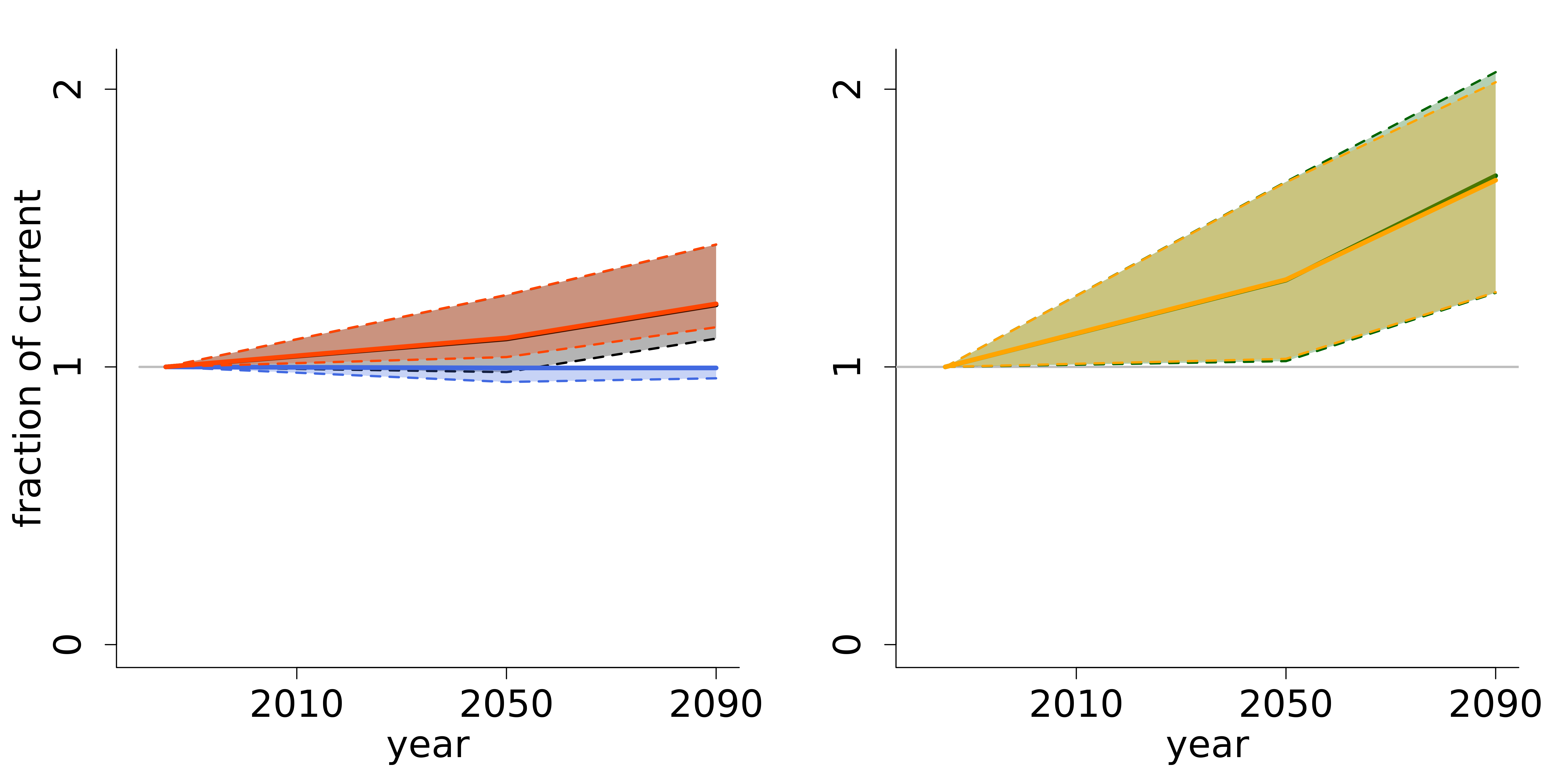

Supplement: S2 Appendix — (ZIP) [file pntd.0014030.s006.zip › Sup. Mat. 6-1 A-L - Species Trends/Bungarus_multicinctus_CCTrends.png]

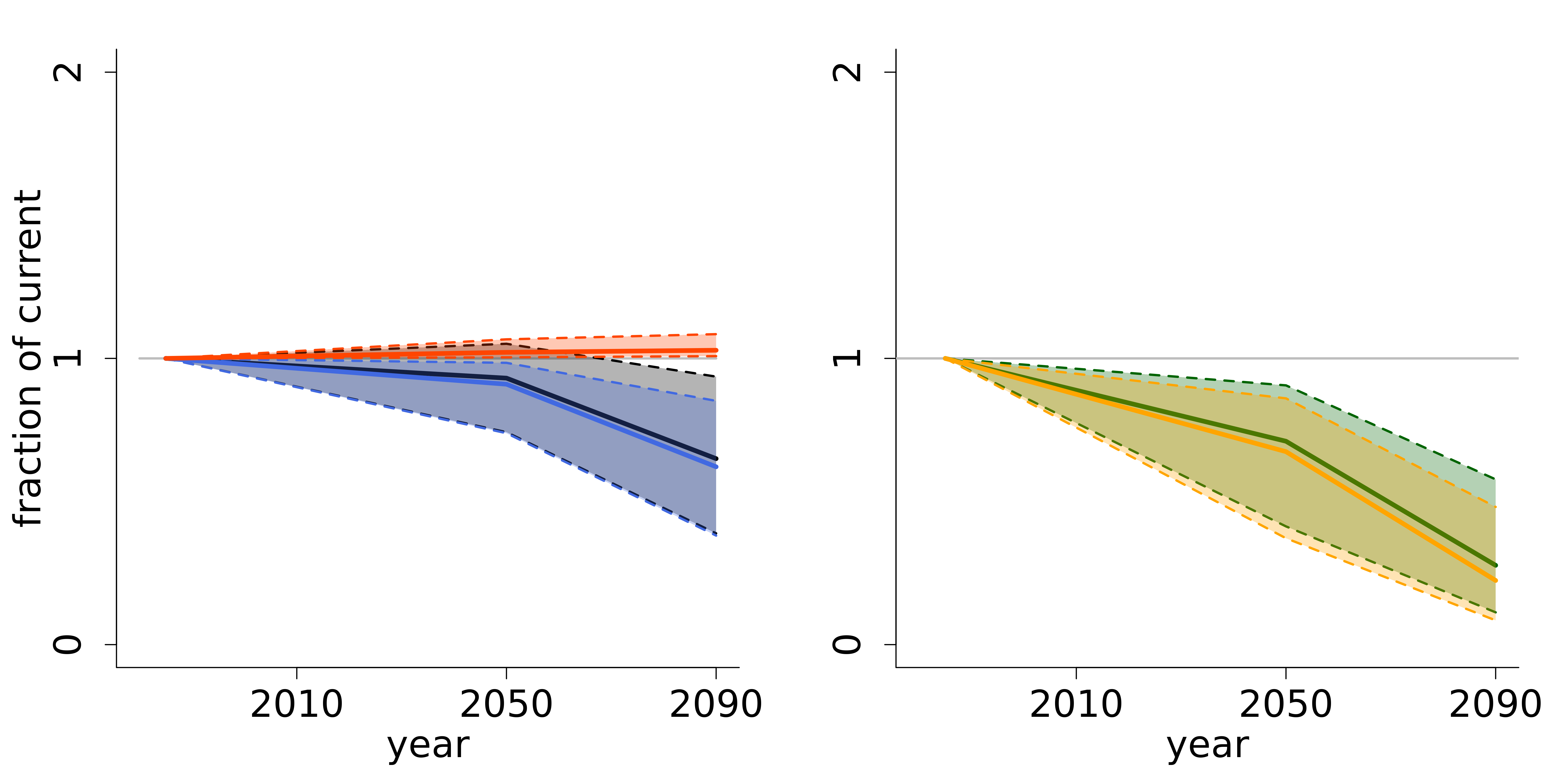

Supplement: S2 Appendix — (ZIP) [file pntd.0014030.s006.zip › Sup. Mat. 6-1 A-L - Species Trends/Bungarus_niger_CCTrends.png]

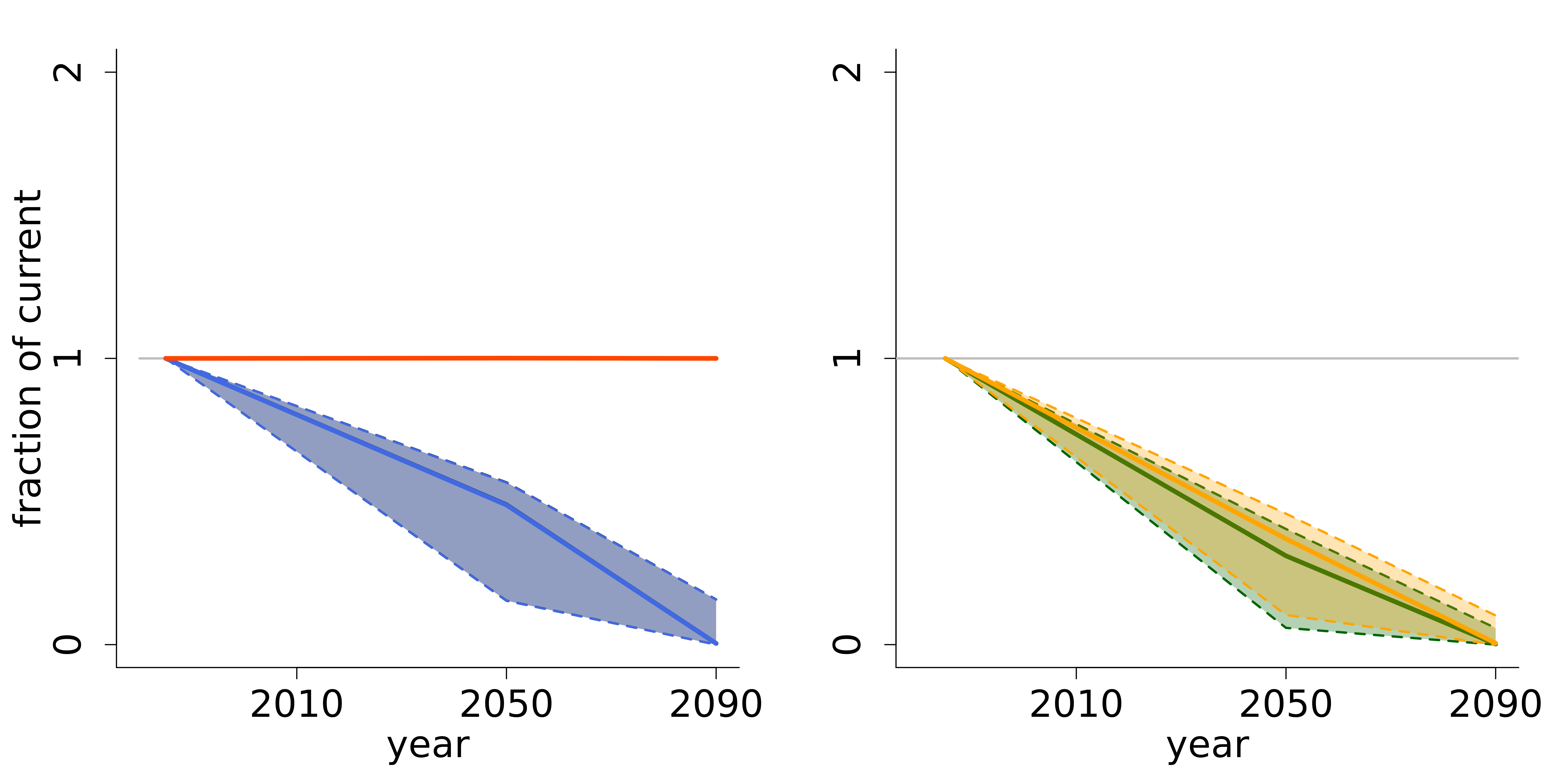

Supplement: S2 Appendix — (ZIP) [file pntd.0014030.s006.zip › Sup. Mat. 6-1 A-L - Species Trends/Bungarus_persicus_CCTrends.png]

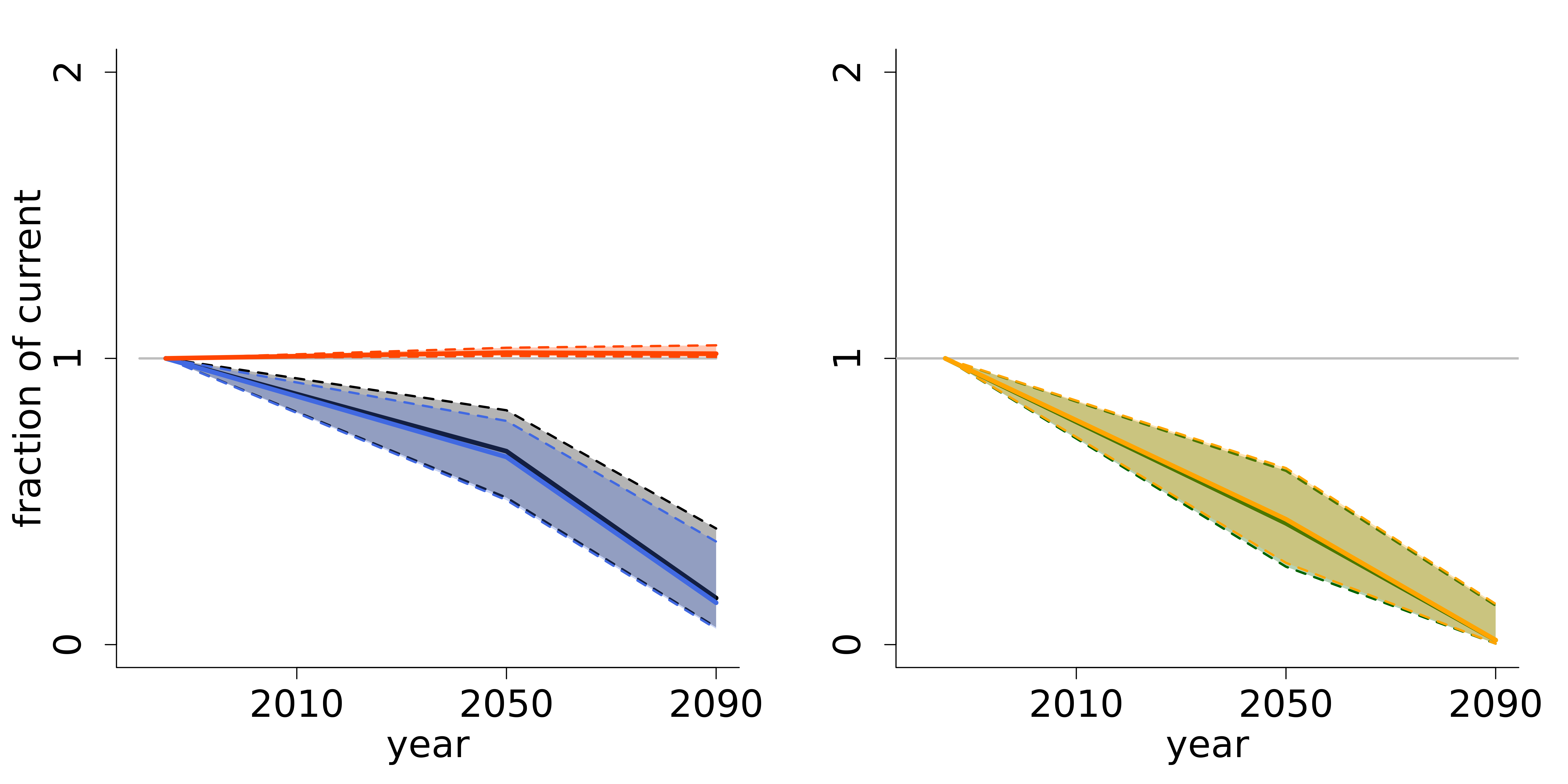

Supplement: S2 Appendix — (ZIP) [file pntd.0014030.s006.zip › Sup. Mat. 6-1 A-L - Species Trends/Bungarus_sindanus_CCTrends.png]

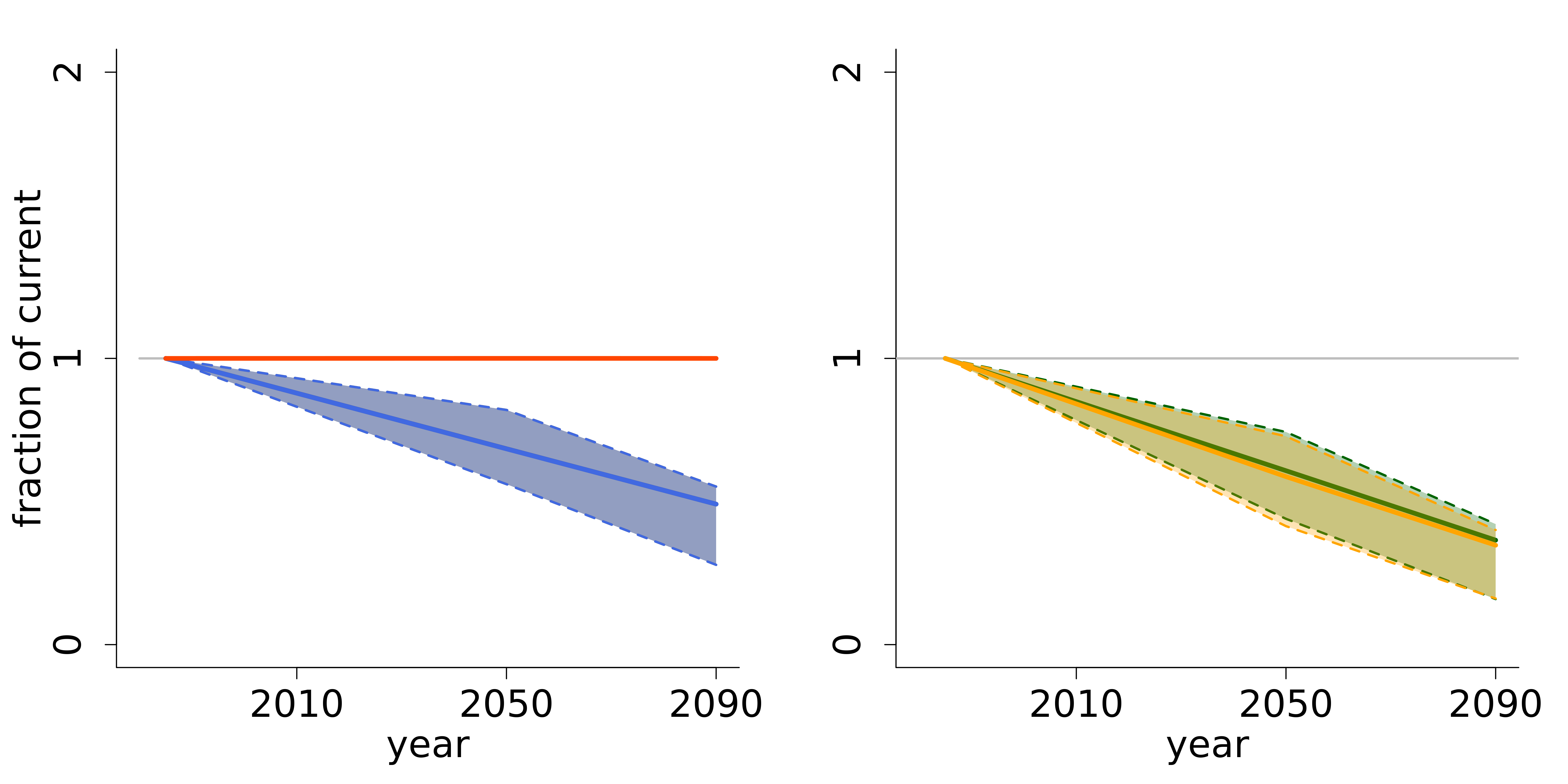

Supplement: S2 Appendix — (ZIP) [file pntd.0014030.s006.zip › Sup. Mat. 6-1 A-L - Species Trends/Bungarus_slowinskii_CCTrends.png]

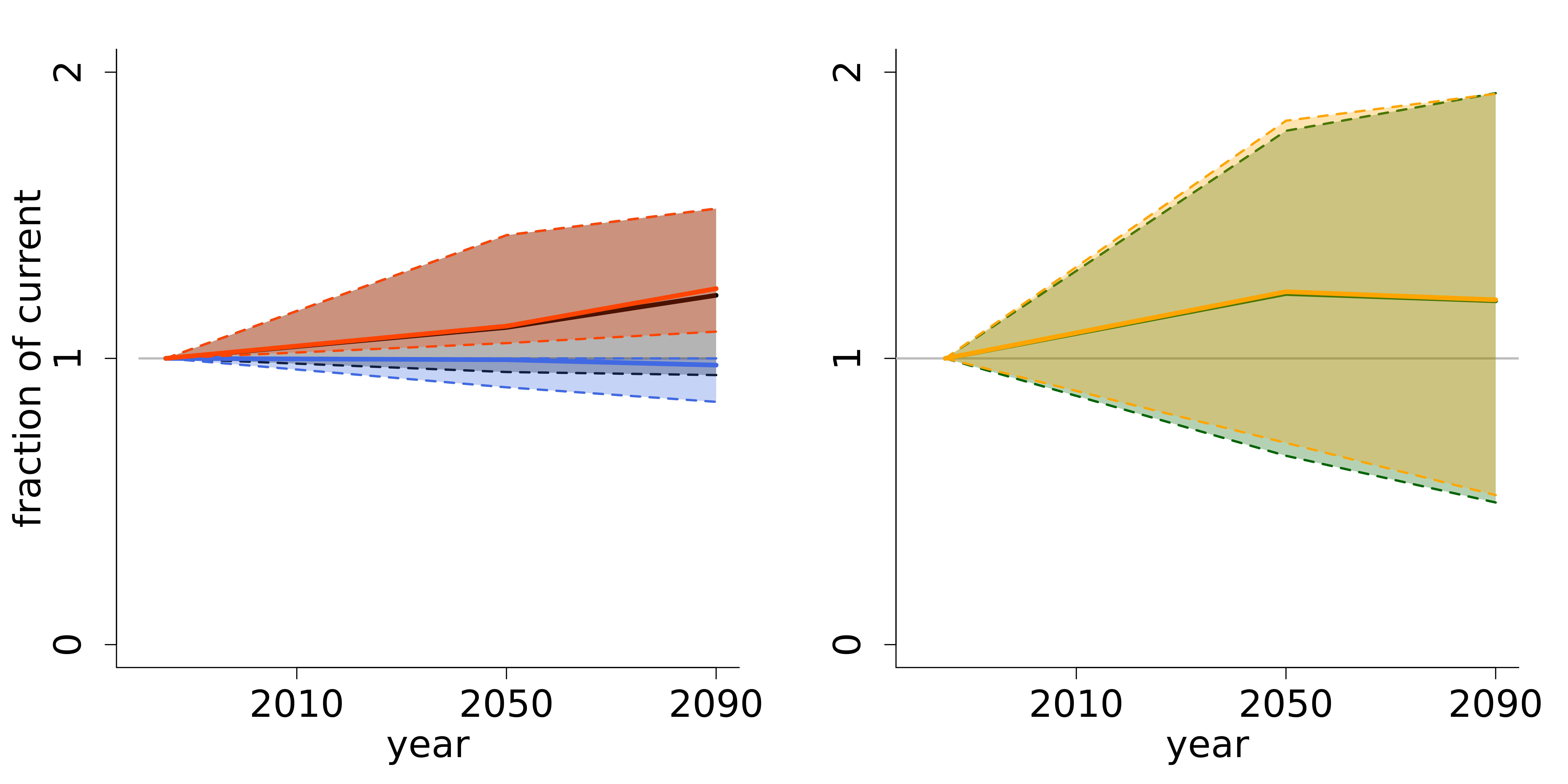

Supplement: S2 Appendix — (ZIP) [file pntd.0014030.s006.zip › Sup. Mat. 6-1 A-L - Species Trends/Bungarus_suzhenae_CCTrends.png]

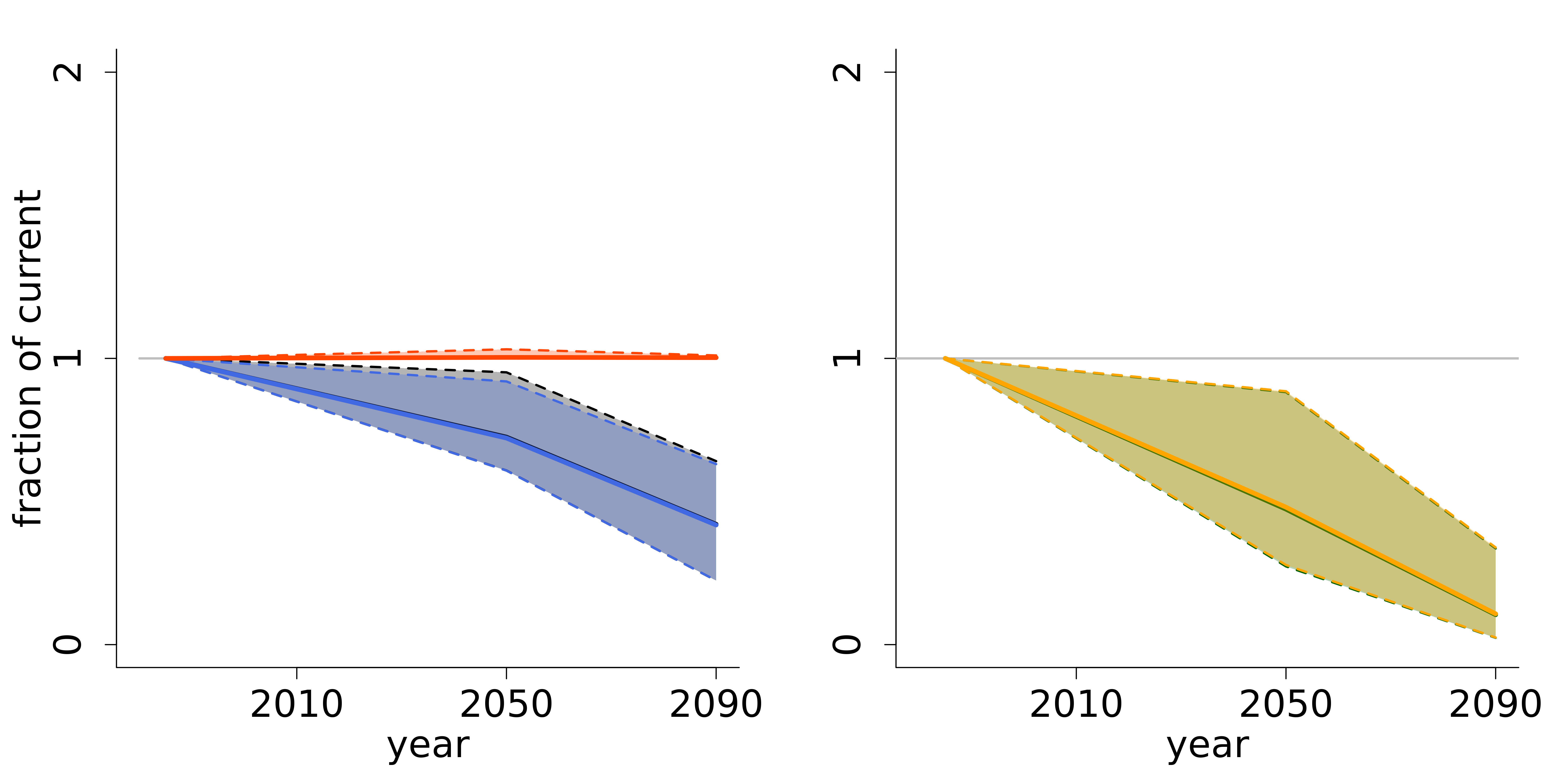

Supplement: S2 Appendix — (ZIP) [file pntd.0014030.s006.zip › Sup. Mat. 6-1 A-L - Species Trends/Bungarus_walli_CCTrends.png]

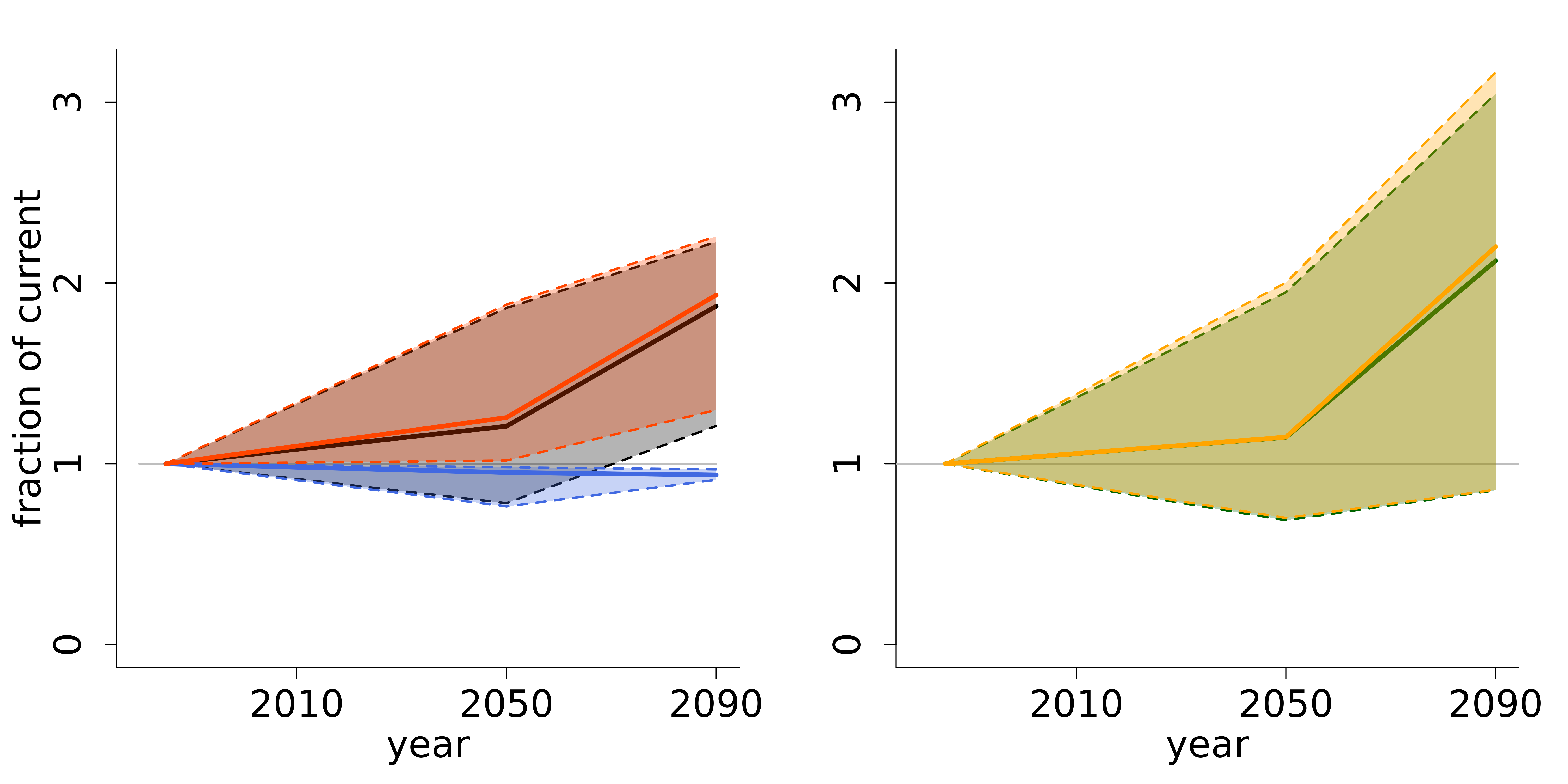

Supplement: S2 Appendix — (ZIP) [file pntd.0014030.s006.zip › Sup. Mat. 6-1 A-L - Species Trends/Bungarus_wanghaotingi_CCTrends.png]

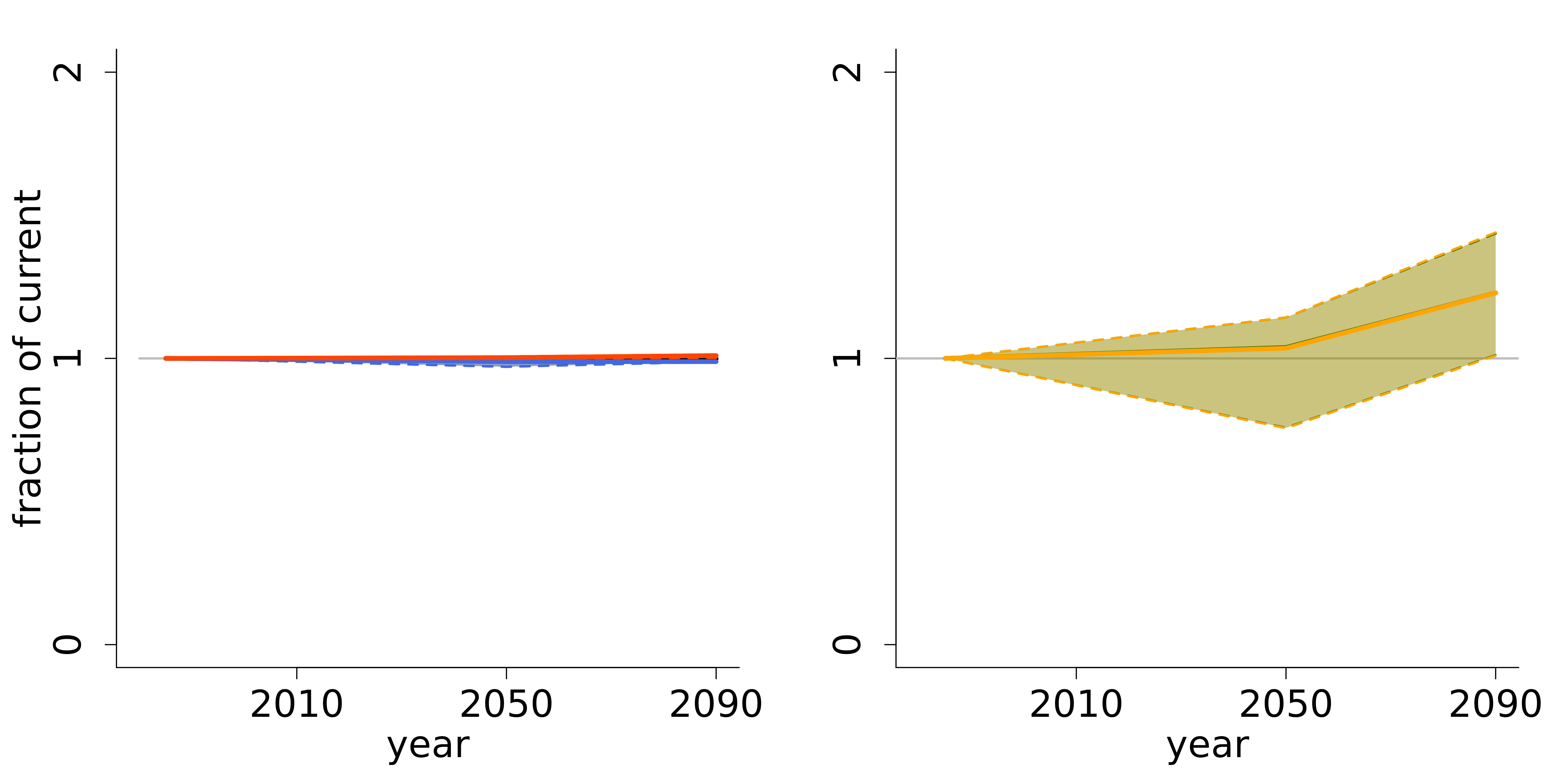

Supplement: S2 Appendix — (ZIP) [file pntd.0014030.s006.zip › Sup. Mat. 6-1 A-L - Species Trends/Calliophis_bilineata_CCTrends.png]

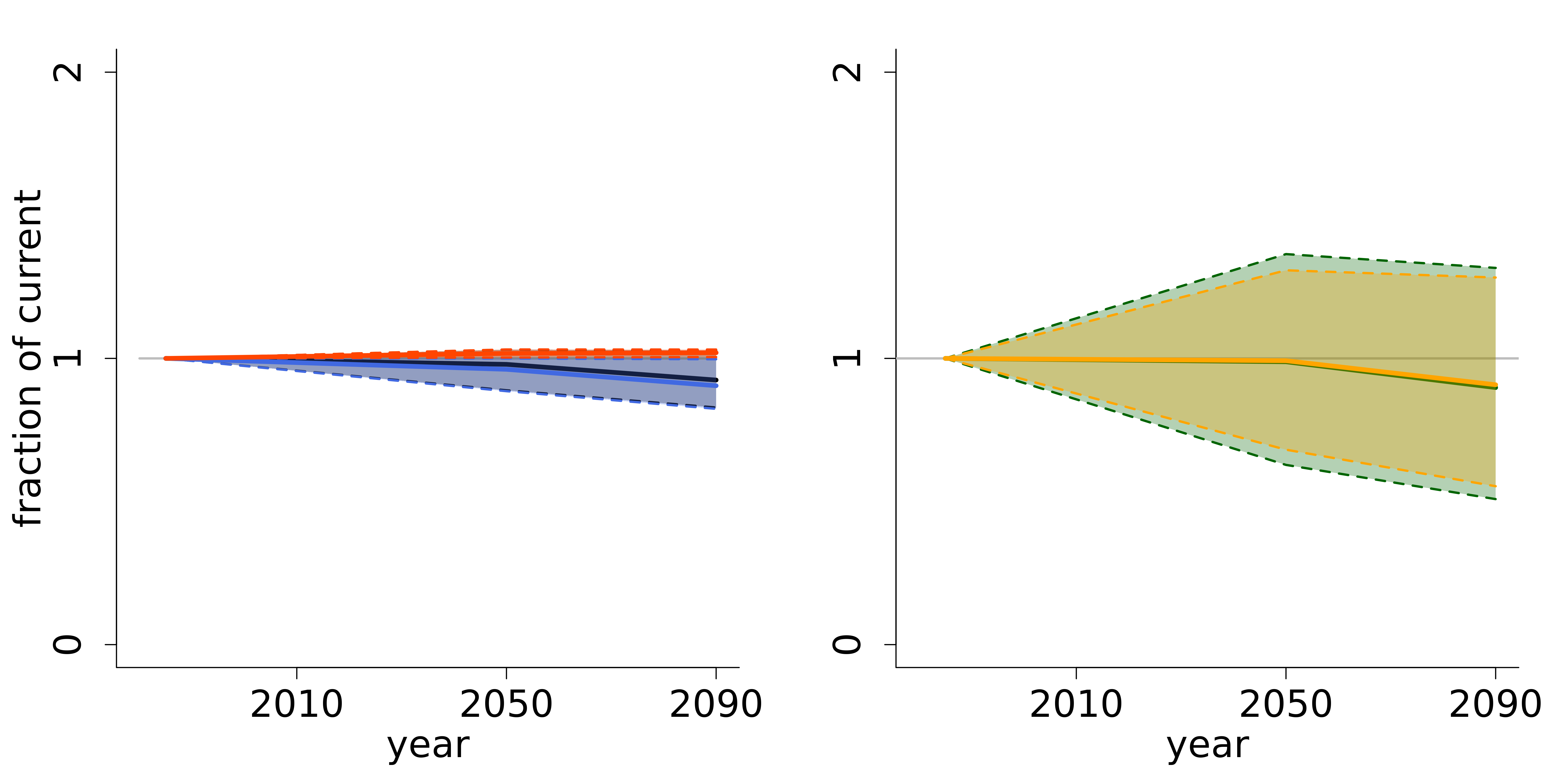

Supplement: S2 Appendix — (ZIP) [file pntd.0014030.s006.zip › Sup. Mat. 6-1 A-L - Species Trends/Calliophis_bivirgatus_CCTrends.png]

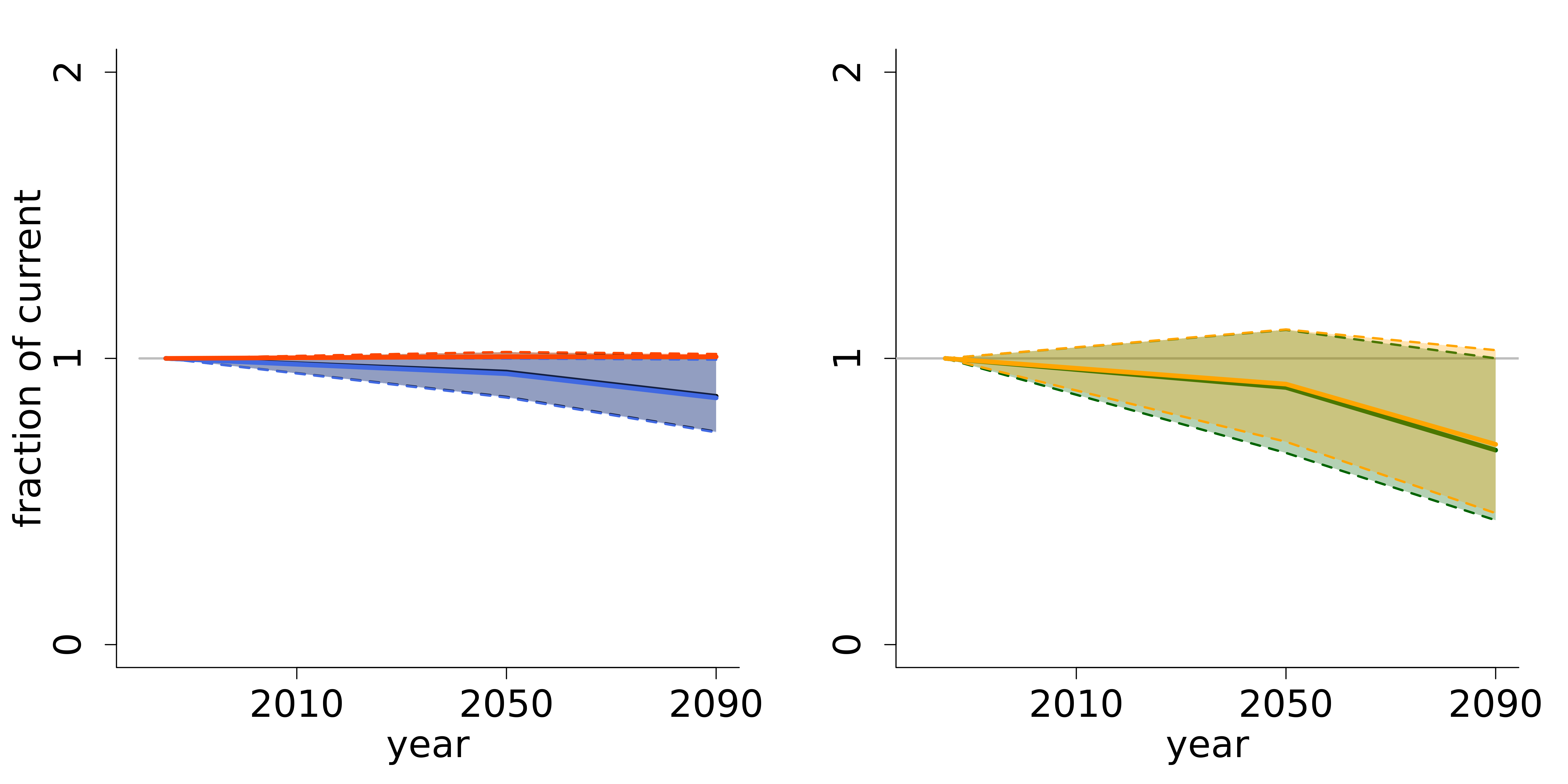

Supplement: S2 Appendix — (ZIP) [file pntd.0014030.s006.zip › Sup. Mat. 6-1 A-L - Species Trends/Calliophis_intestinalis_CCTrends.png]

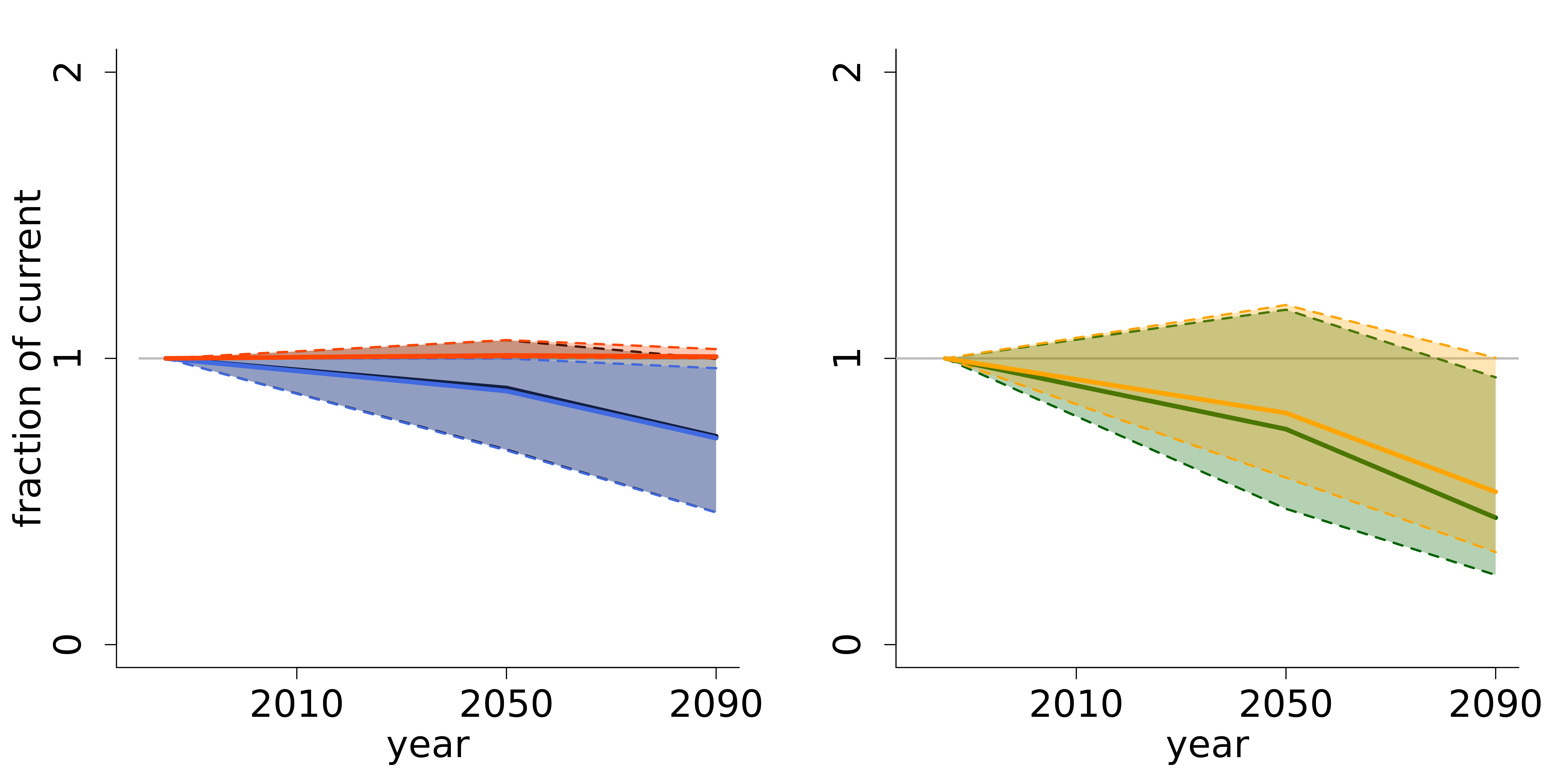

Supplement: S2 Appendix — (ZIP) [file pntd.0014030.s006.zip › Sup. Mat. 6-1 A-L - Species Trends/Calliophis_nigrotaeniatus_CCTrends.png]

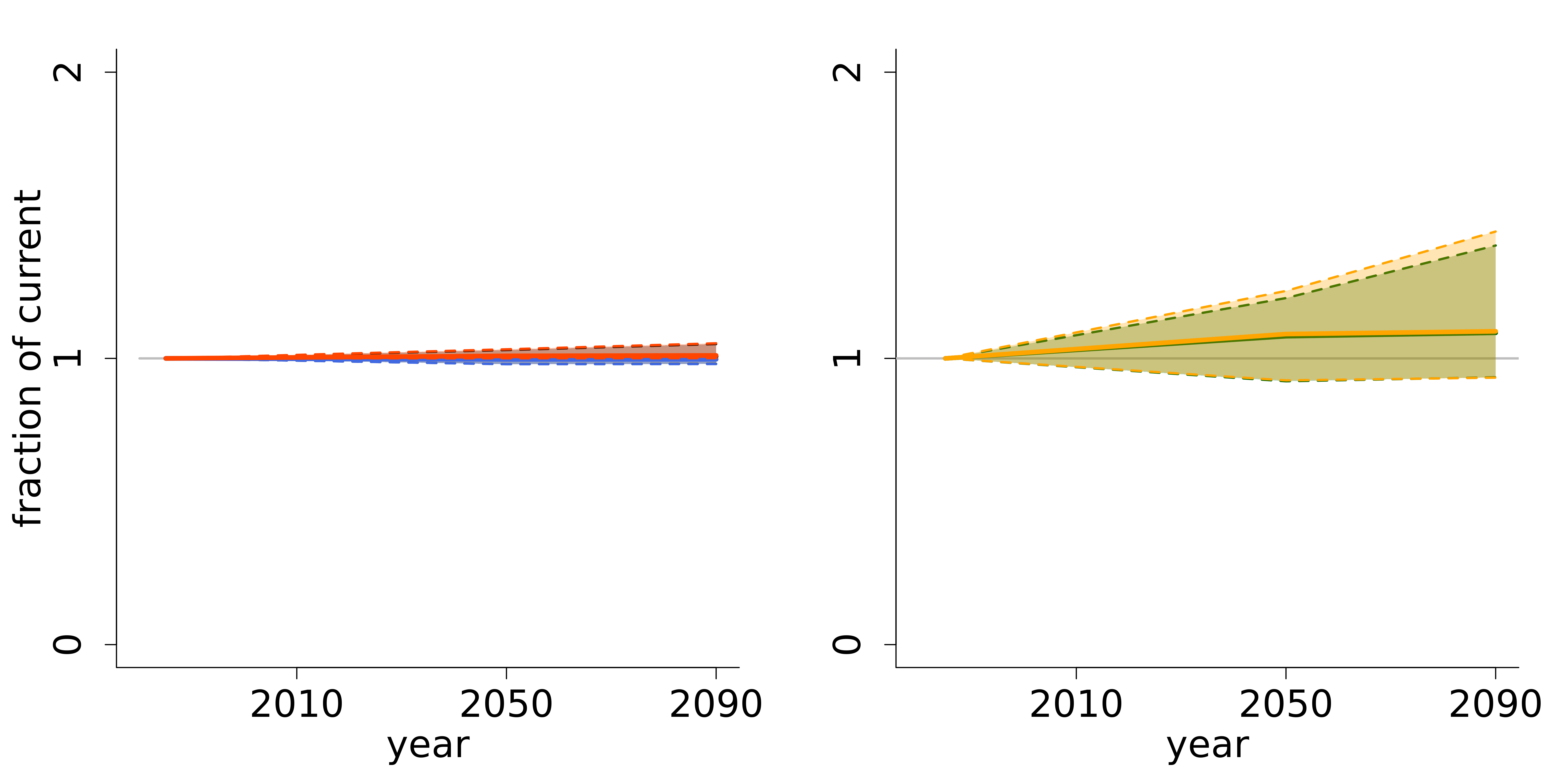

Supplement: S2 Appendix — (ZIP) [file pntd.0014030.s006.zip › Sup. Mat. 6-1 A-L - Species Trends/Calliophis_philippina_CCTrends.png]

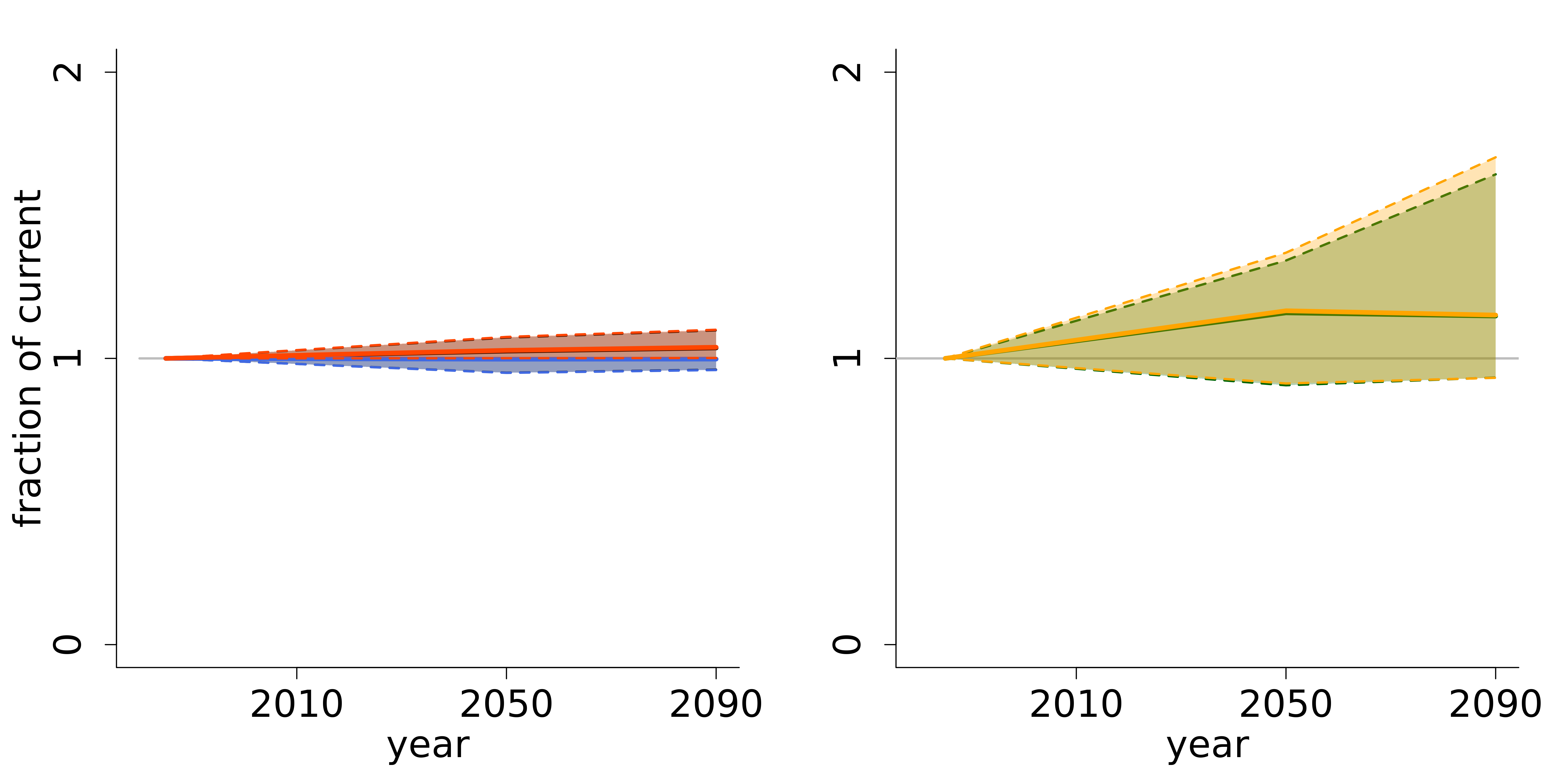

Supplement: S2 Appendix — (ZIP) [file pntd.0014030.s006.zip › Sup. Mat. 6-1 A-L - Species Trends/Calliophis_salitan_CCTrends.png]

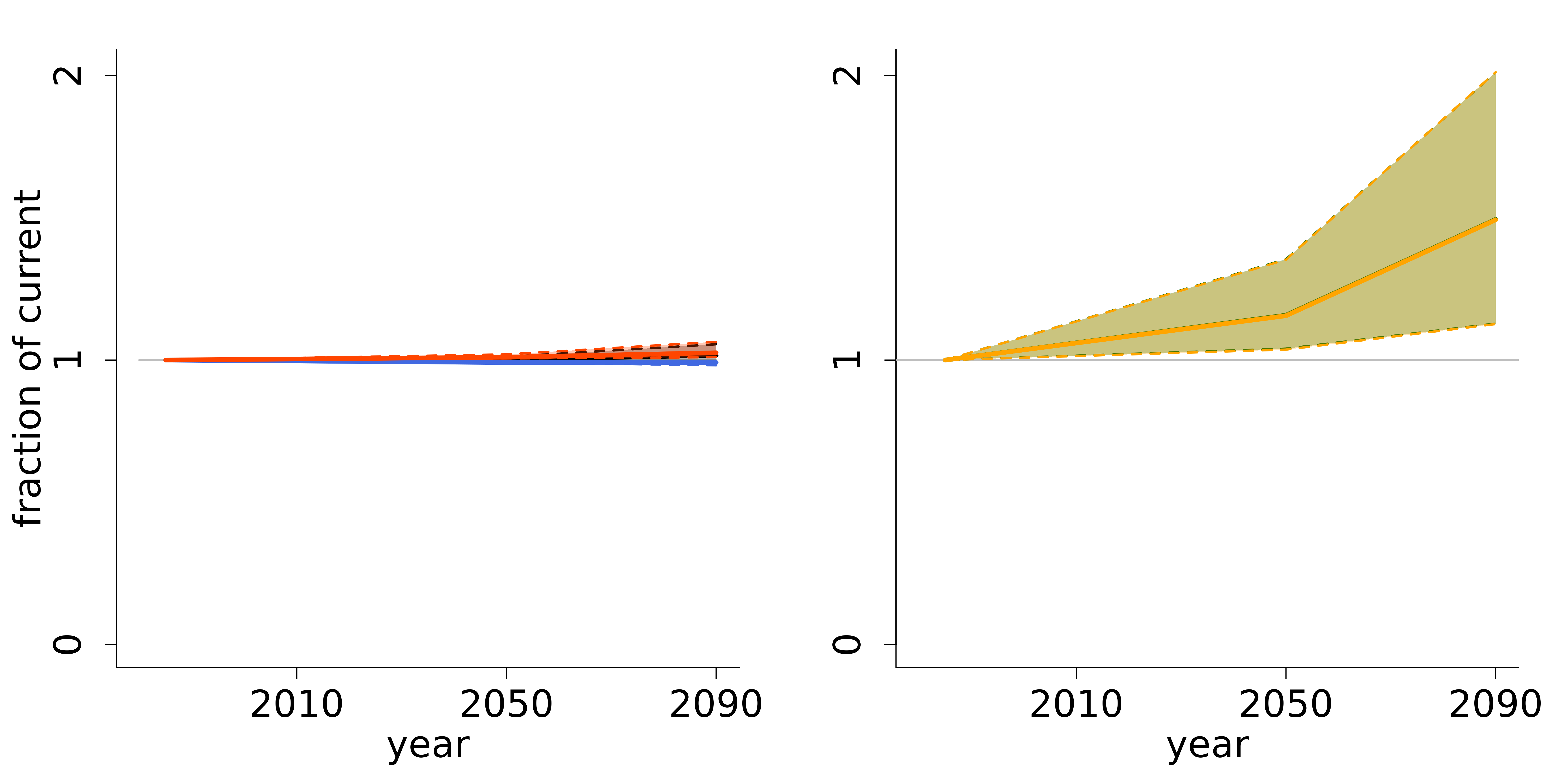

Supplement: S2 Appendix — (ZIP) [file pntd.0014030.s006.zip › Sup. Mat. 6-1 A-L - Species Trends/Calliophis_suluensis_CCTrends.png]

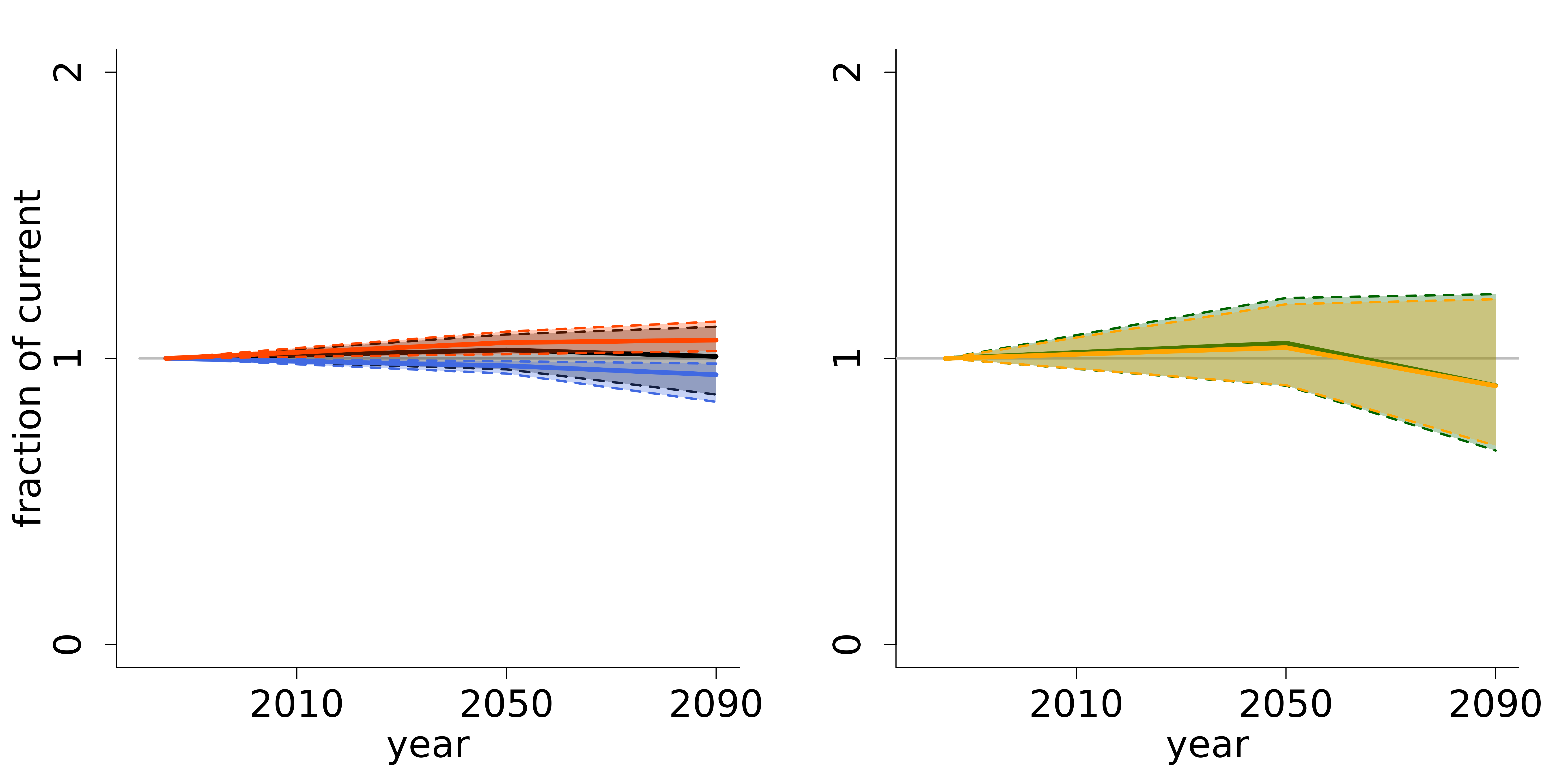

Supplement: S2 Appendix — (ZIP) [file pntd.0014030.s006.zip › Sup. Mat. 6-1 A-L - Species Trends/Calloselasma_rhodostoma_CCTrends.png]

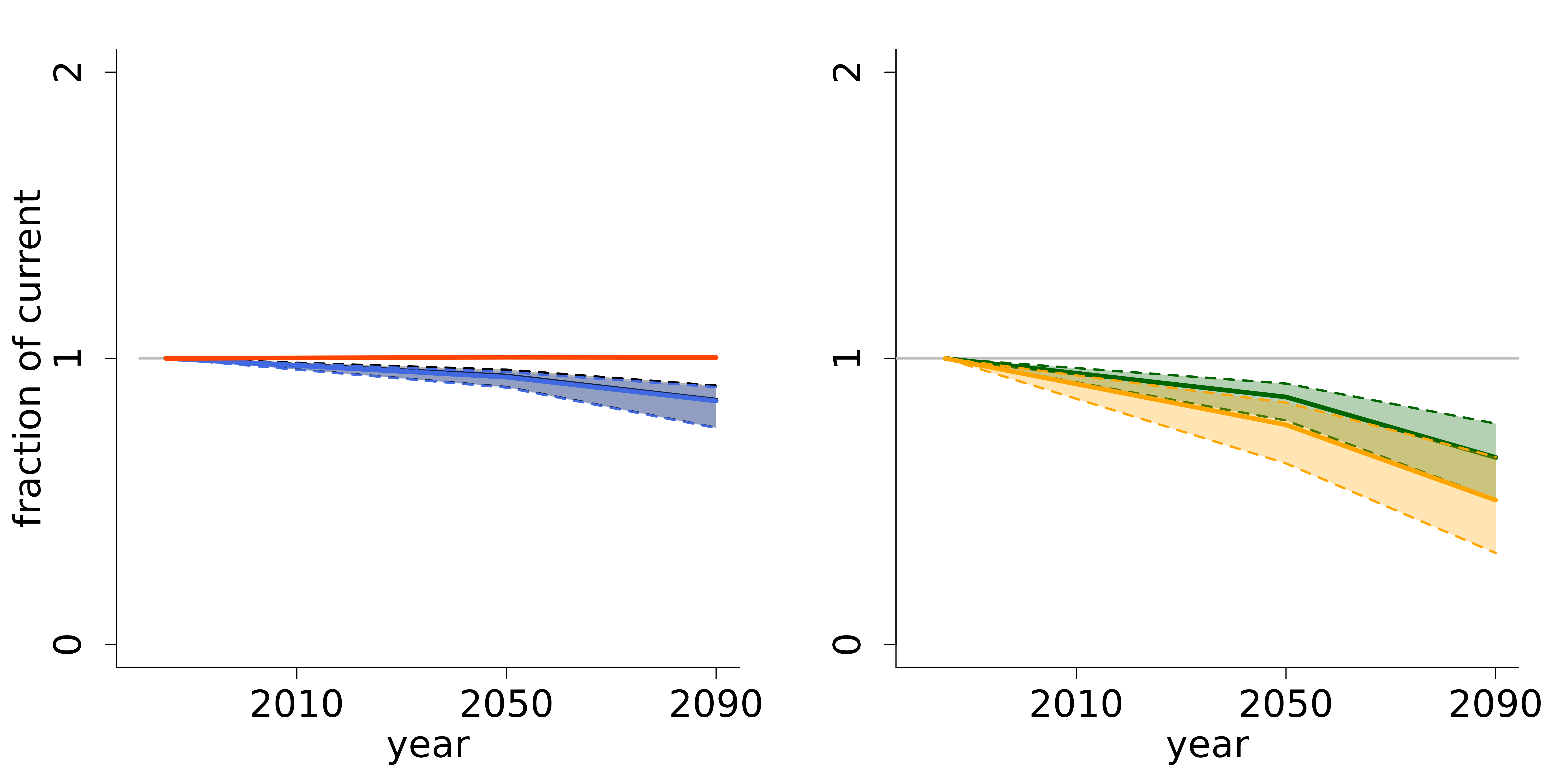

Supplement: S2 Appendix — (ZIP) [file pntd.0014030.s006.zip › Sup. Mat. 6-1 A-L - Species Trends/Cerastes_cerastes_CCTrends.png]

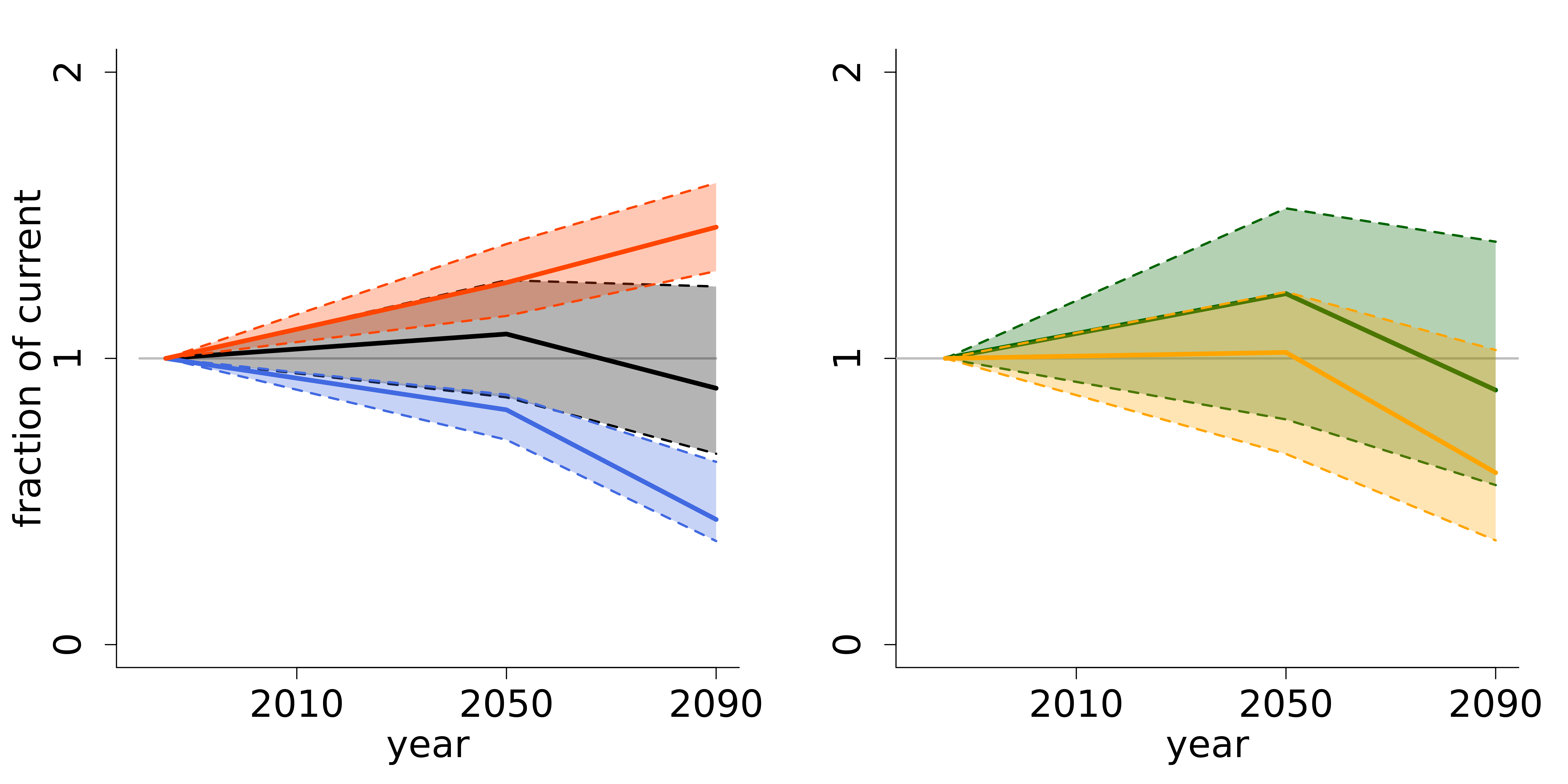

Supplement: S2 Appendix — (ZIP) [file pntd.0014030.s006.zip › Sup. Mat. 6-1 A-L - Species Trends/Cerastes_gasperettii_CCTrends.png]

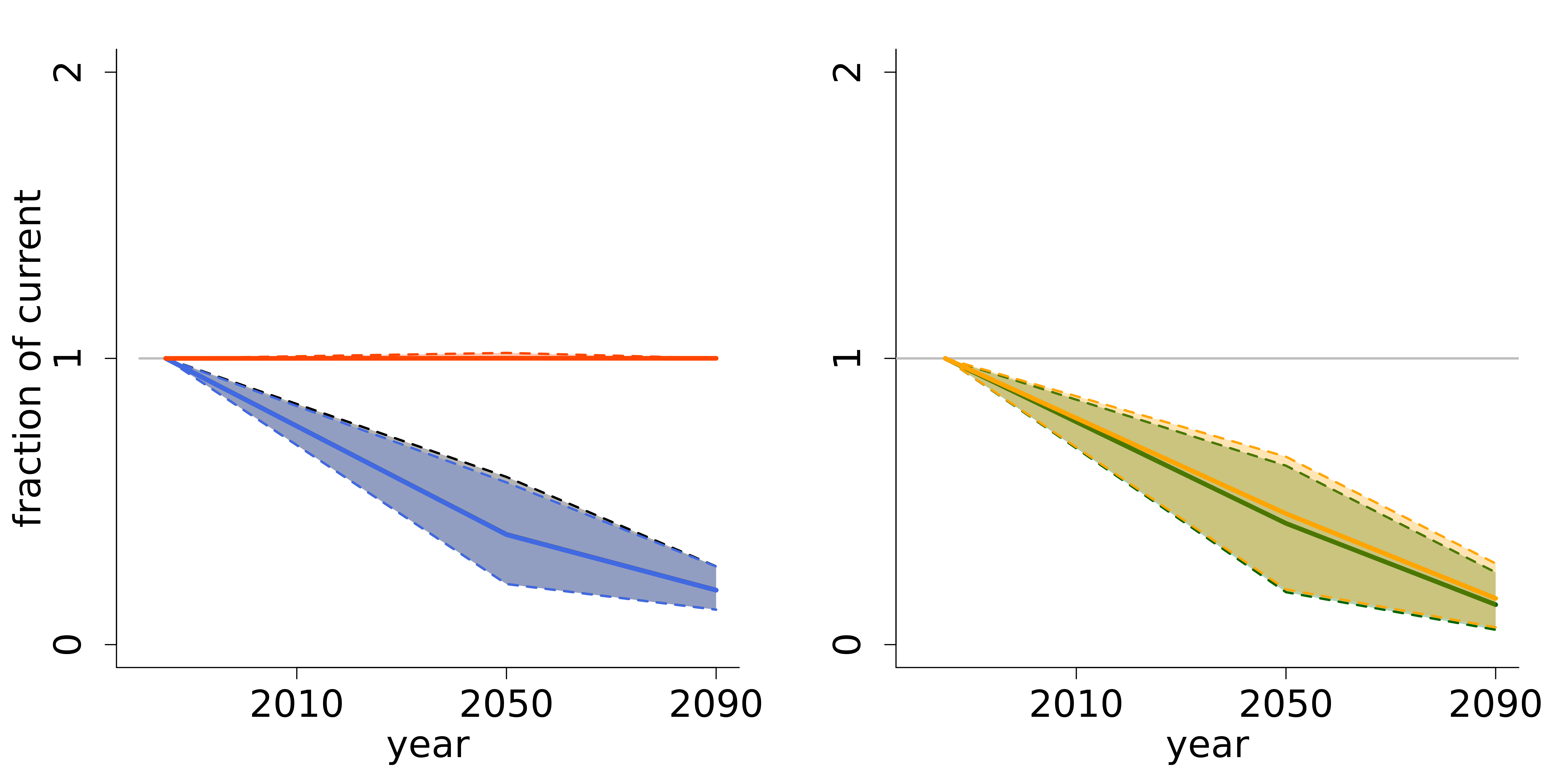

Supplement: S2 Appendix — (ZIP) [file pntd.0014030.s006.zip › Sup. Mat. 6-1 A-L - Species Trends/Cerrophidion_godmani_CCTrends.png]

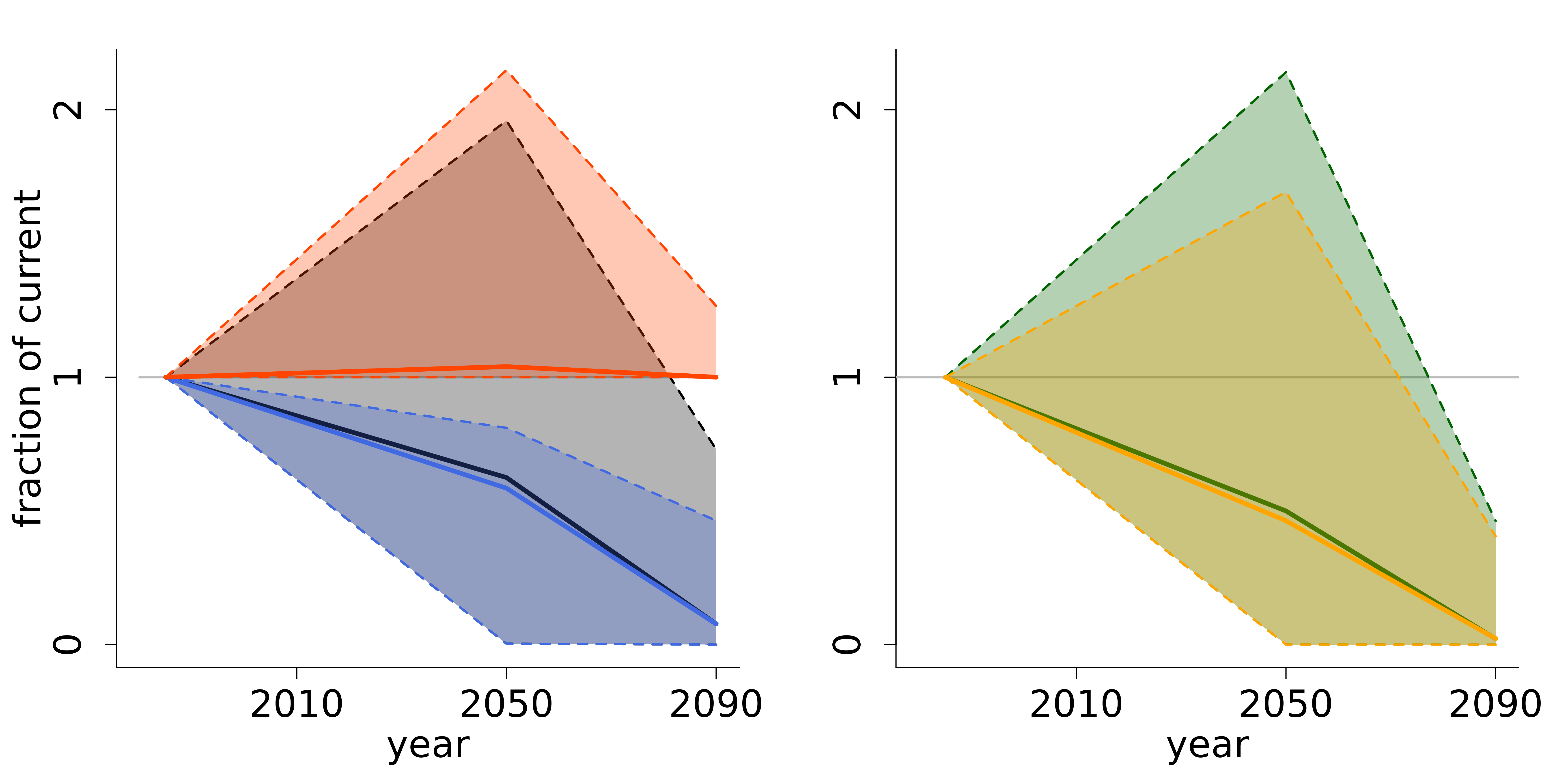

Supplement: S2 Appendix — (ZIP) [file pntd.0014030.s006.zip › Sup. Mat. 6-1 A-L - Species Trends/Cerrophidion_petlalcalensis_CCTrends.png]

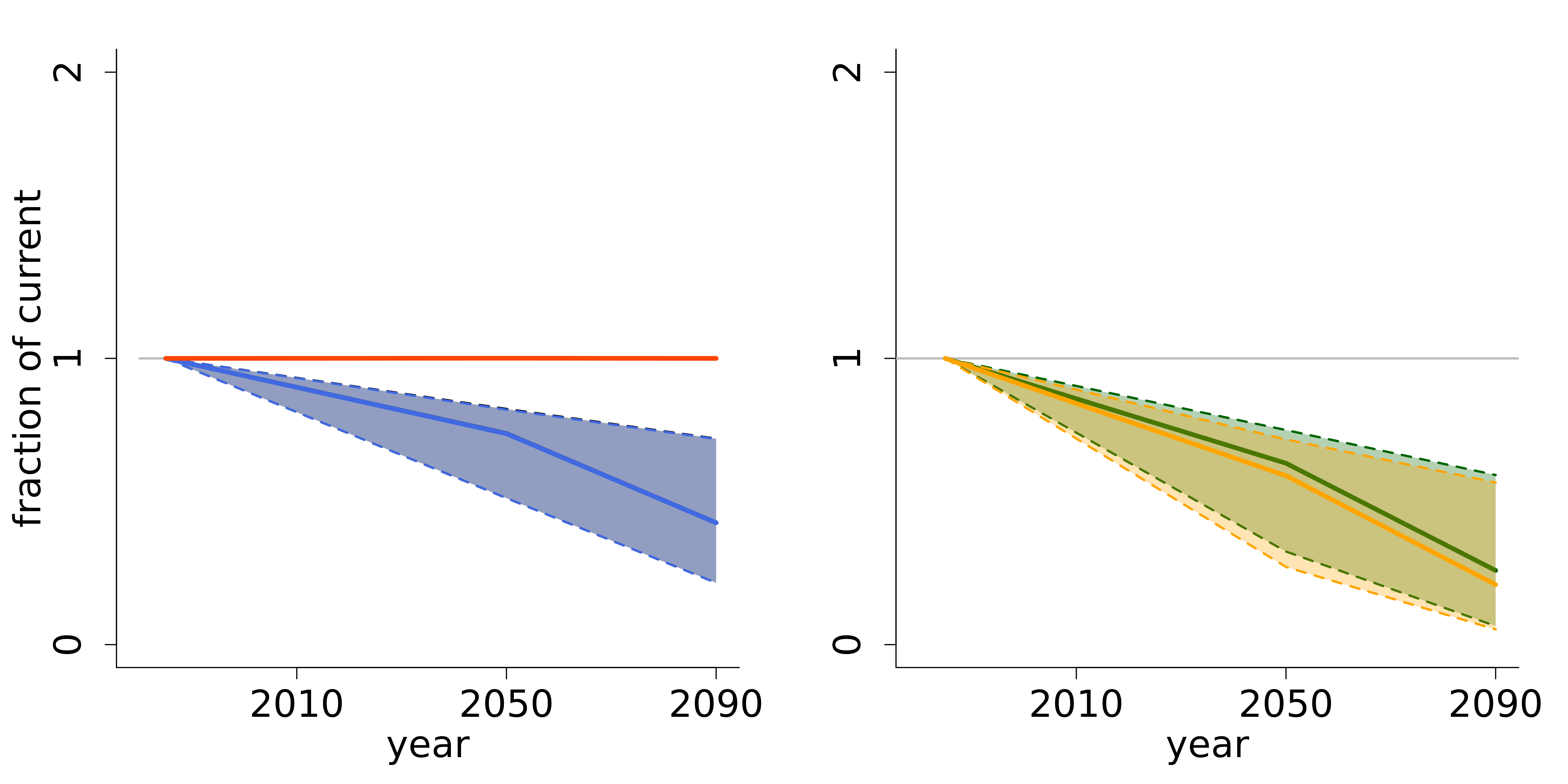

Supplement: S2 Appendix — (ZIP) [file pntd.0014030.s006.zip › Sup. Mat. 6-1 A-L - Species Trends/Cerrophidion_sasai_CCTrends.png]

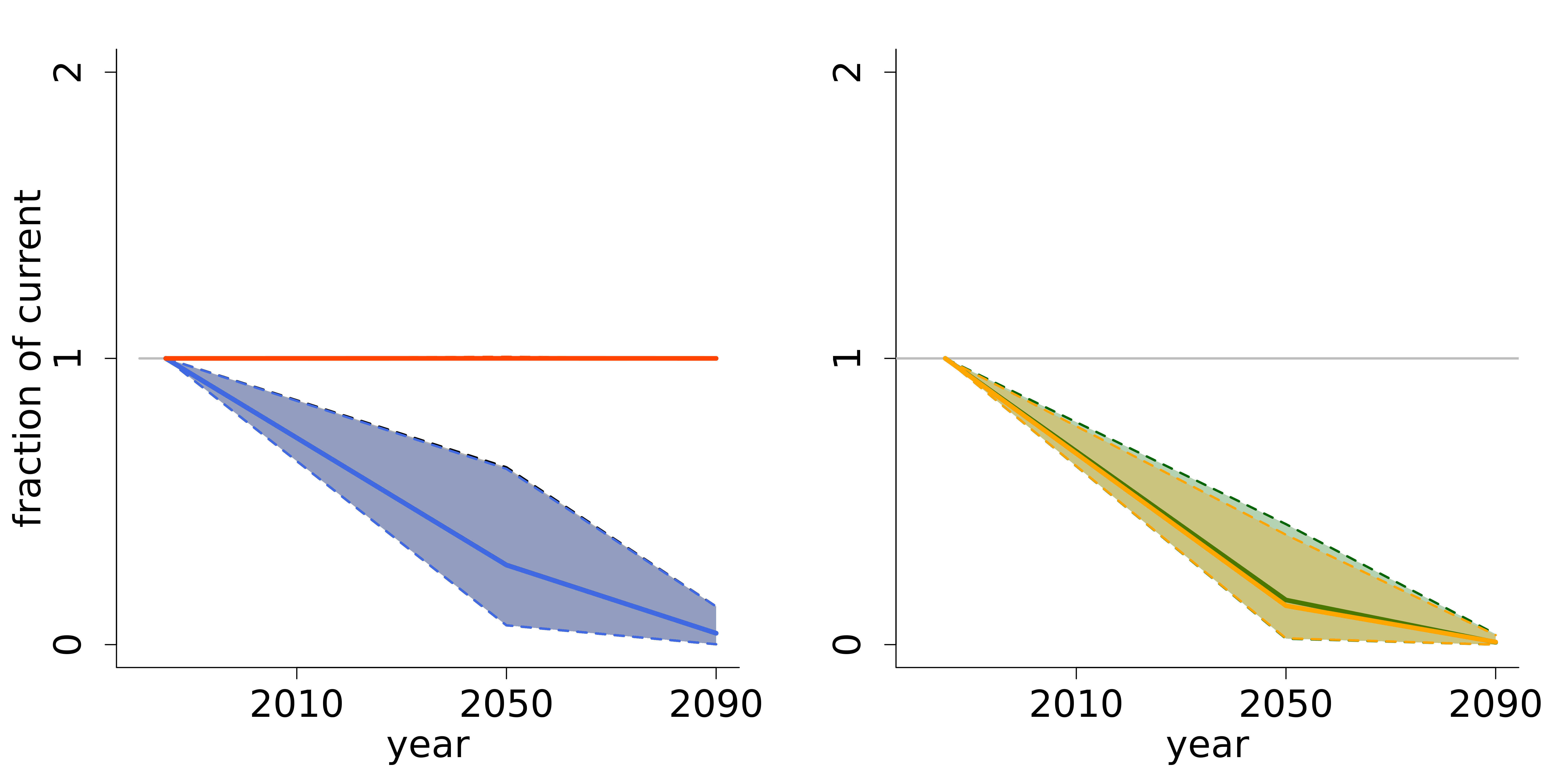

Supplement: S2 Appendix — (ZIP) [file pntd.0014030.s006.zip › Sup. Mat. 6-1 A-L - Species Trends/Cerrophidion_tzotzilorum_CCTrends.png]

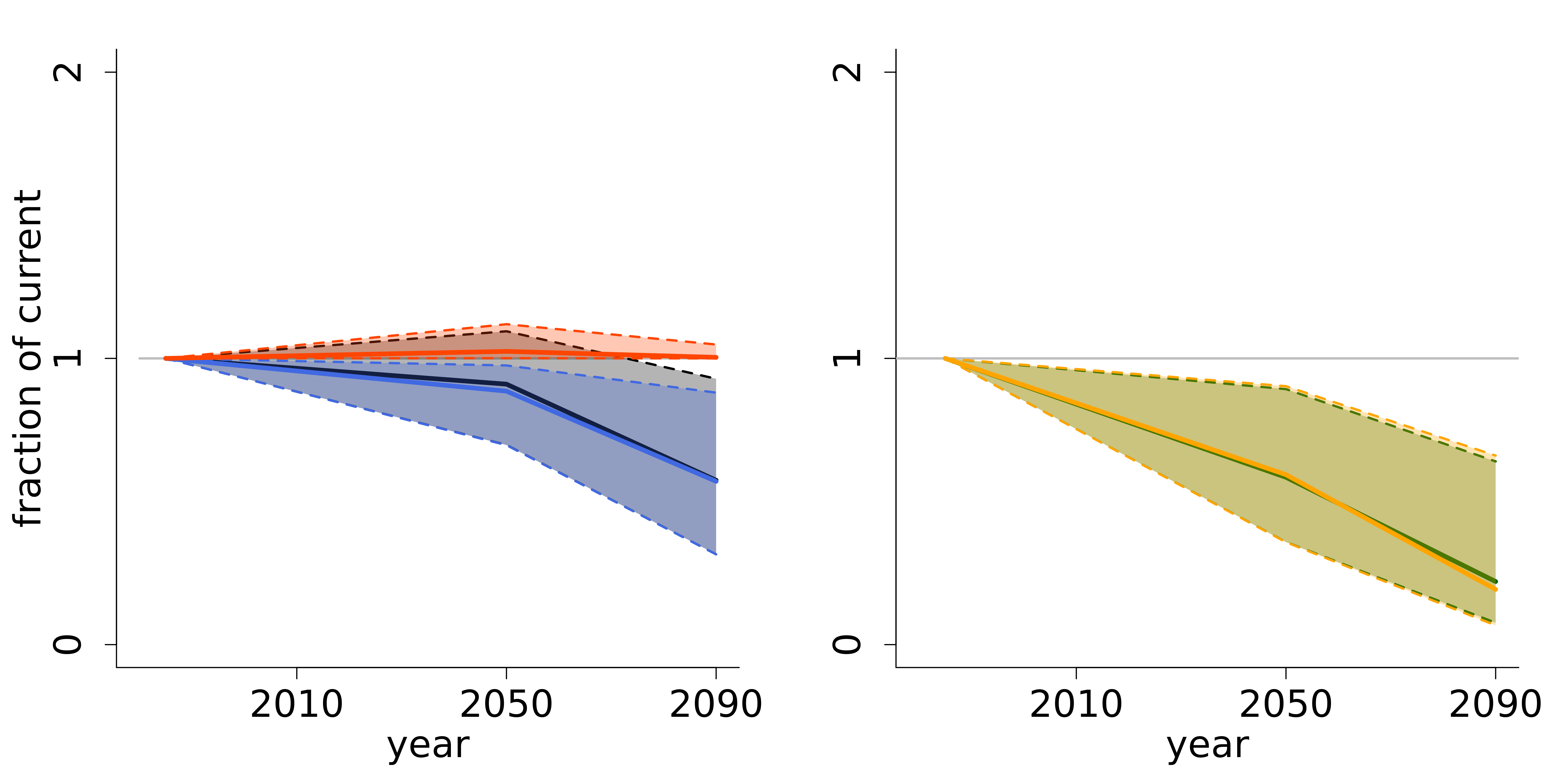

Supplement: S2 Appendix — (ZIP) [file pntd.0014030.s006.zip › Sup. Mat. 6-1 A-L - Species Trends/Cerrophidion_wilsoni_CCTrends.png]

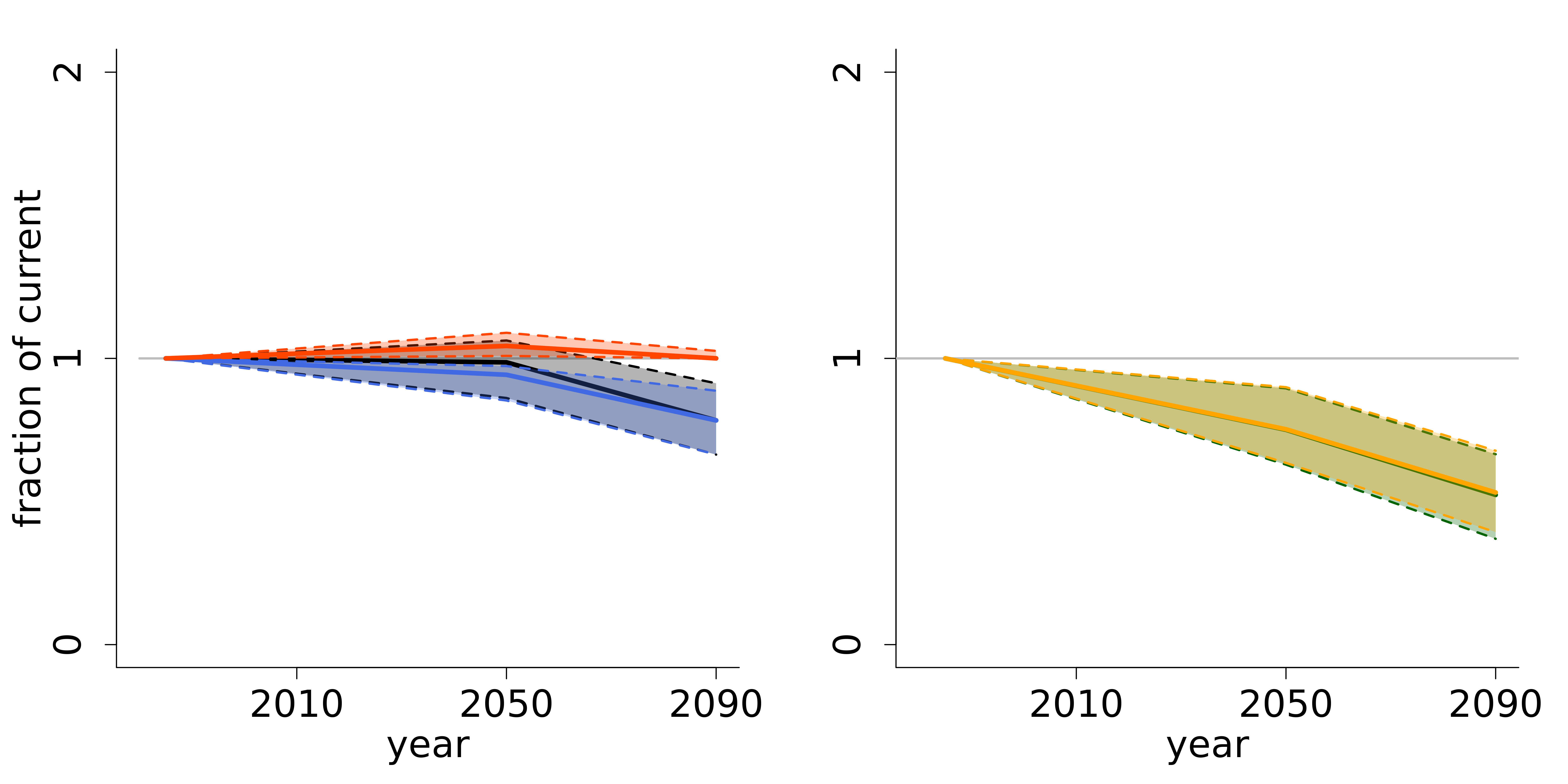

Supplement: S2 Appendix — (ZIP) [file pntd.0014030.s006.zip › Sup. Mat. 6-1 A-L - Species Trends/Crotalus_adamanteus_CCTrends.png]

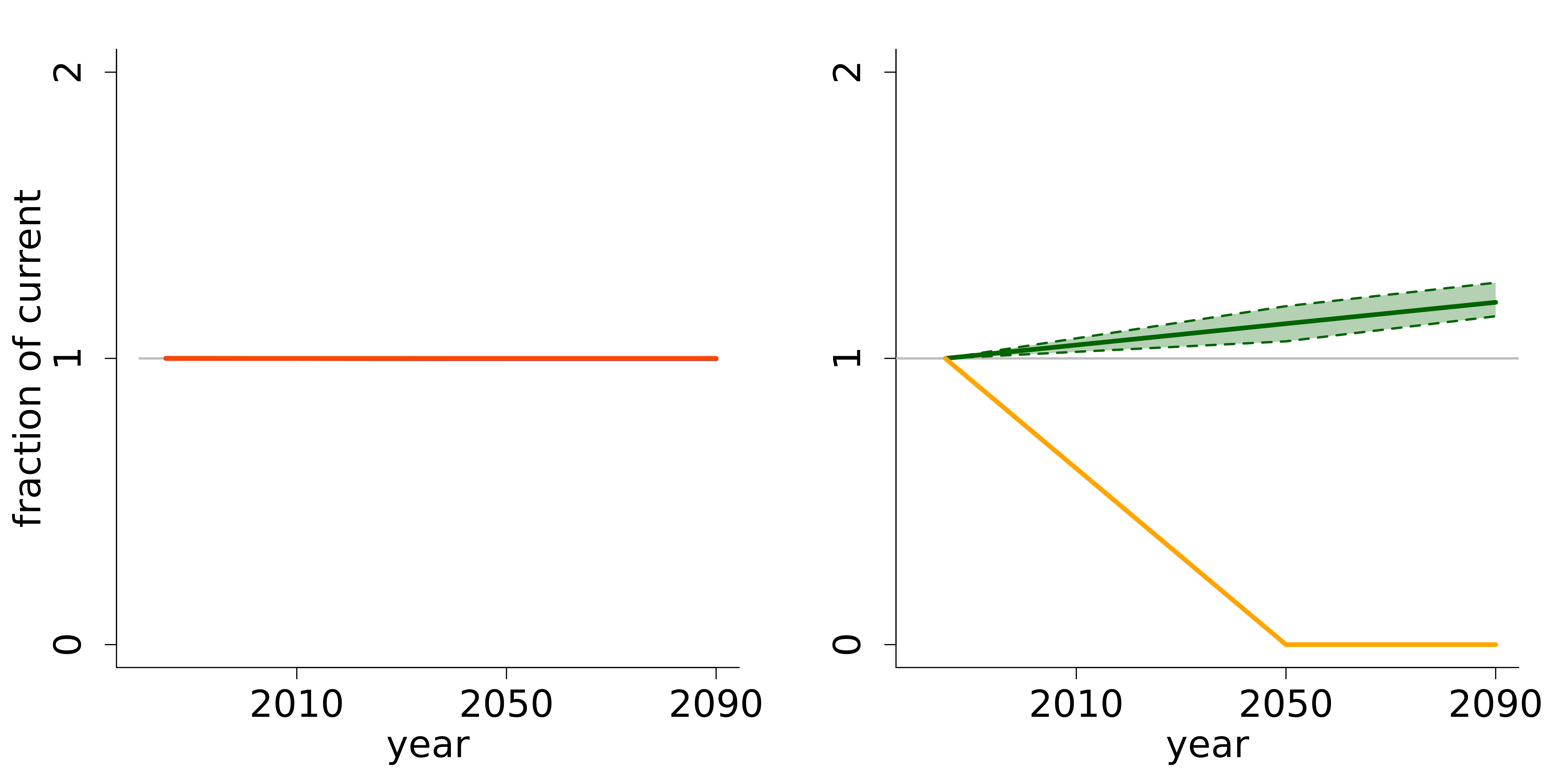

Supplement: S2 Appendix — (ZIP) [file pntd.0014030.s006.zip › Sup. Mat. 6-1 A-L - Species Trends/Crotalus_angelensis_CCTrends.png]

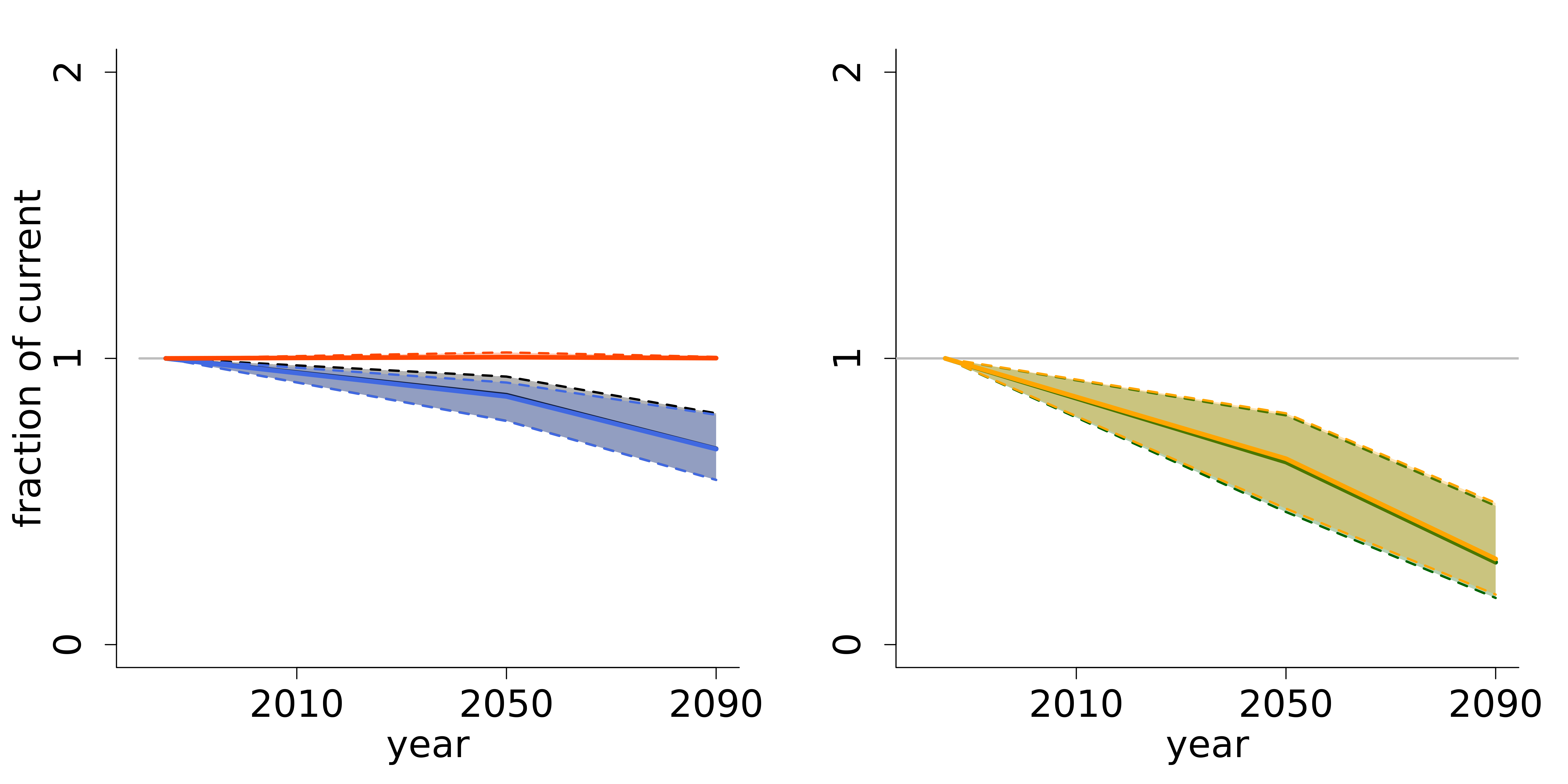

Supplement: S2 Appendix — (ZIP) [file pntd.0014030.s006.zip › Sup. Mat. 6-1 A-L - Species Trends/Crotalus_aquilus_CCTrends.png]

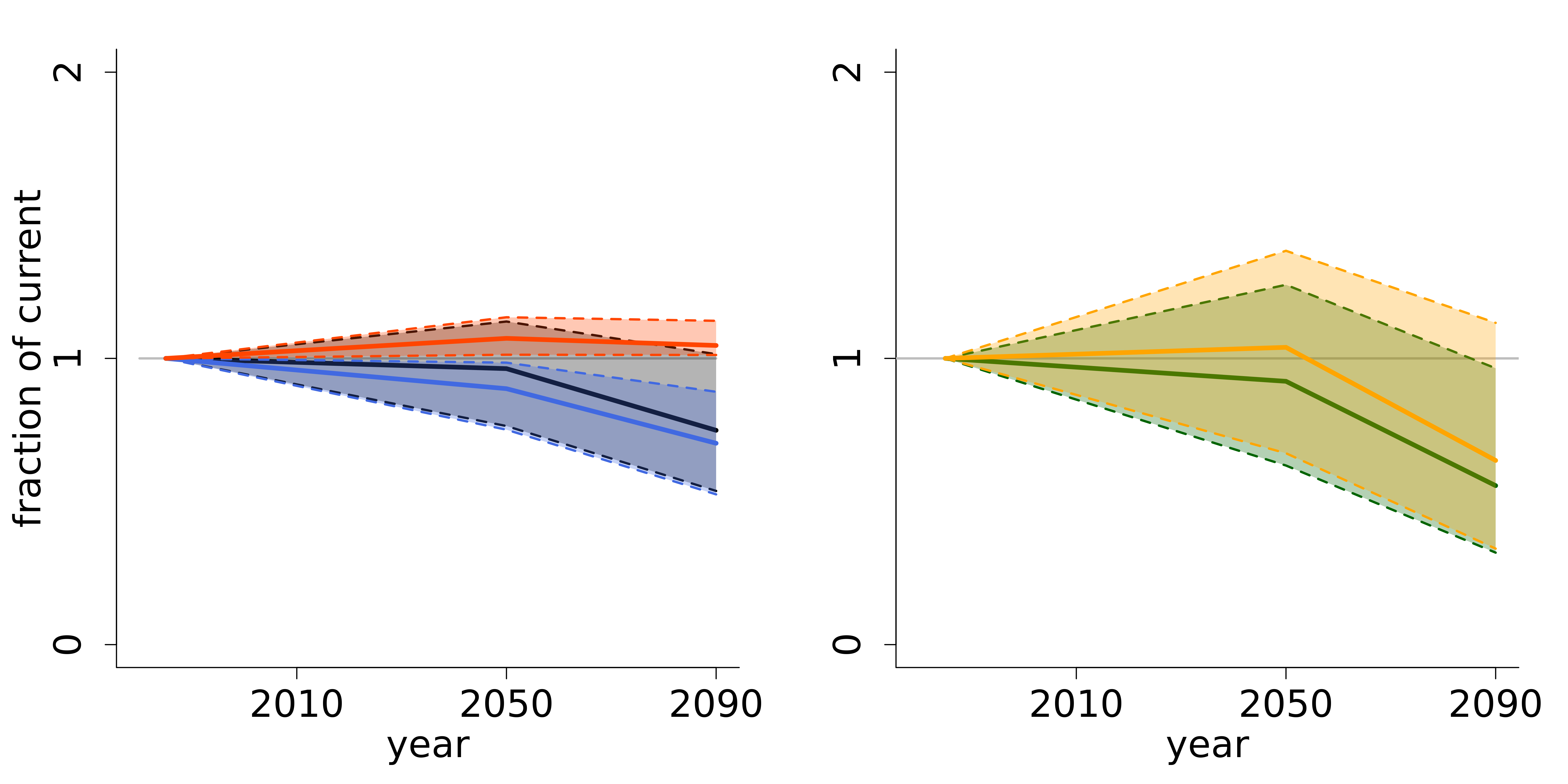

Supplement: S2 Appendix — (ZIP) [file pntd.0014030.s006.zip › Sup. Mat. 6-1 A-L - Species Trends/Crotalus_armstrongi_CCTrends.png]

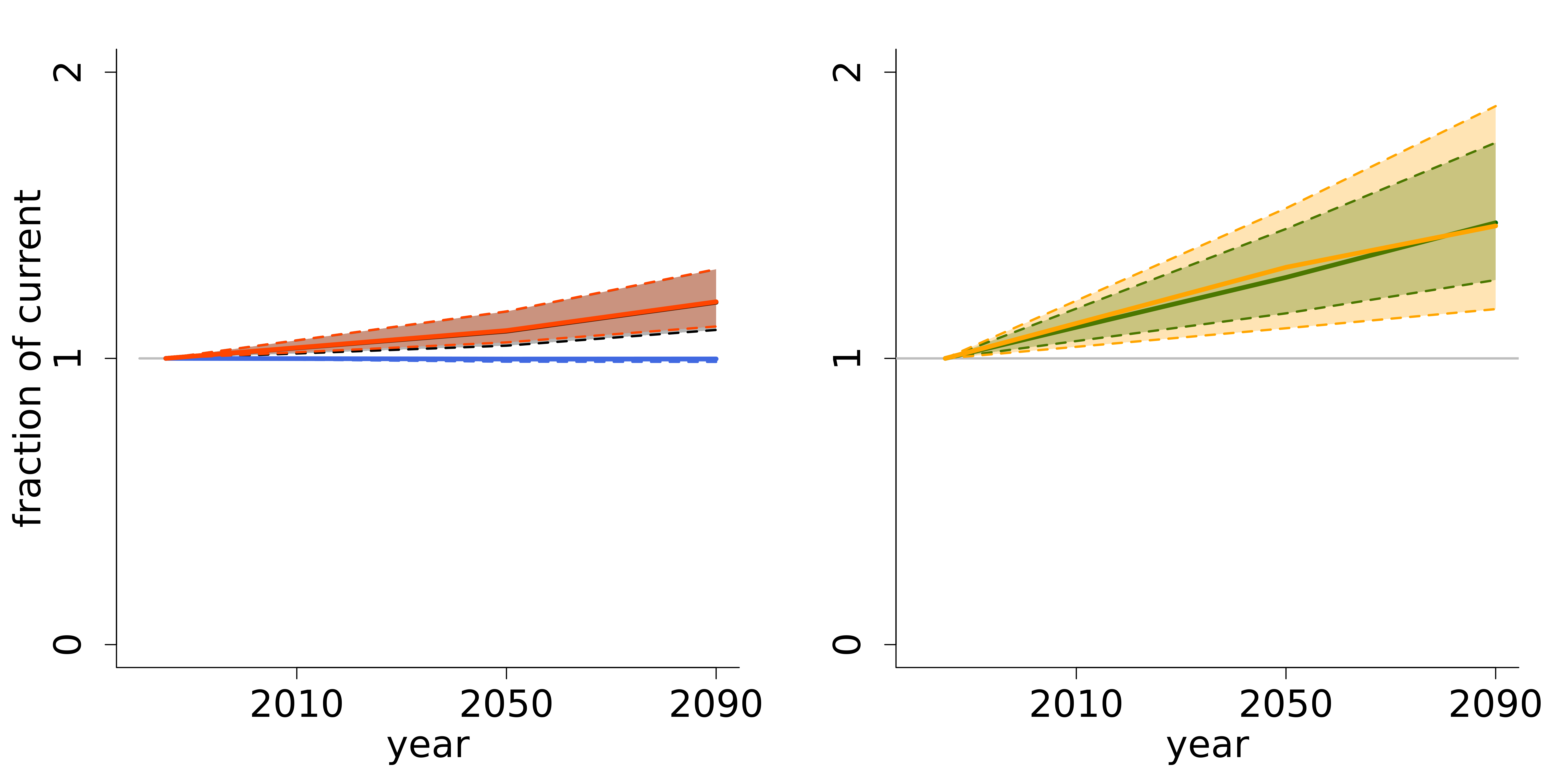

Supplement: S2 Appendix — (ZIP) [file pntd.0014030.s006.zip › Sup. Mat. 6-1 A-L - Species Trends/Crotalus_atrox_CCTrends.png]

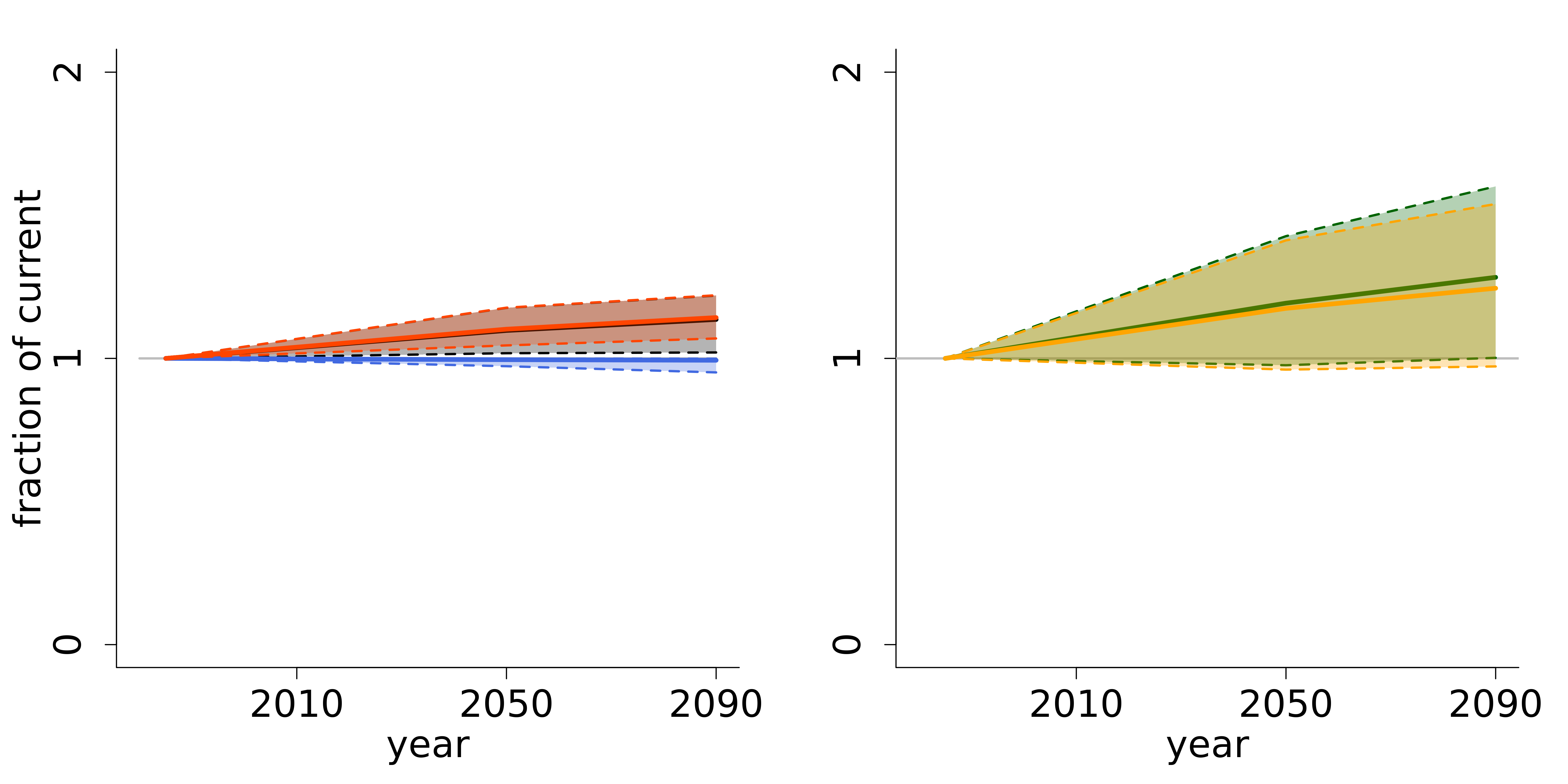

Supplement: S2 Appendix — (ZIP) [file pntd.0014030.s006.zip › Sup. Mat. 6-1 A-L - Species Trends/Crotalus_basiliscus_CCTrends.png]

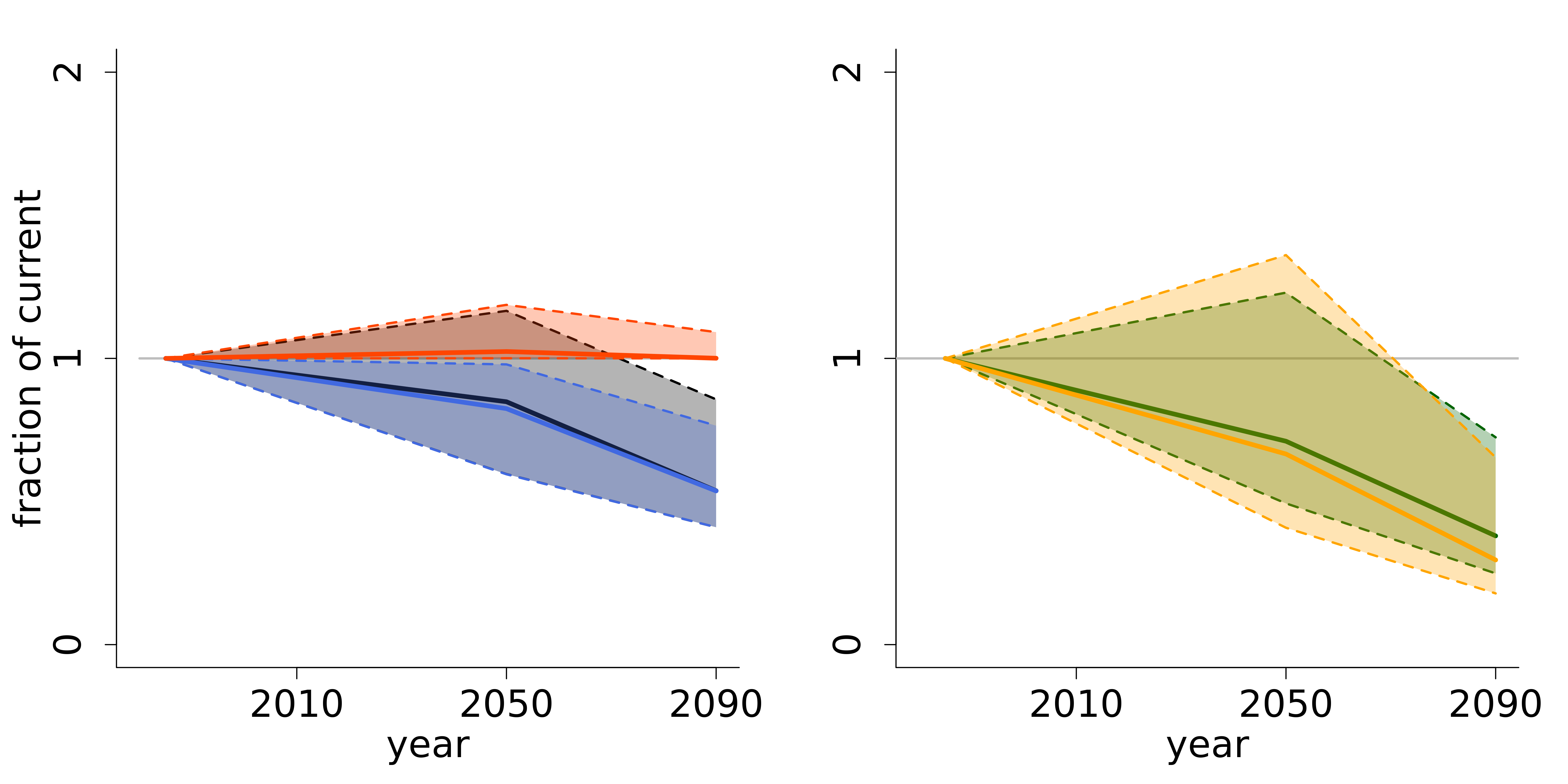

Supplement: S2 Appendix — (ZIP) [file pntd.0014030.s006.zip › Sup. Mat. 6-1 A-L - Species Trends/Crotalus_campbelli_CCTrends.png]

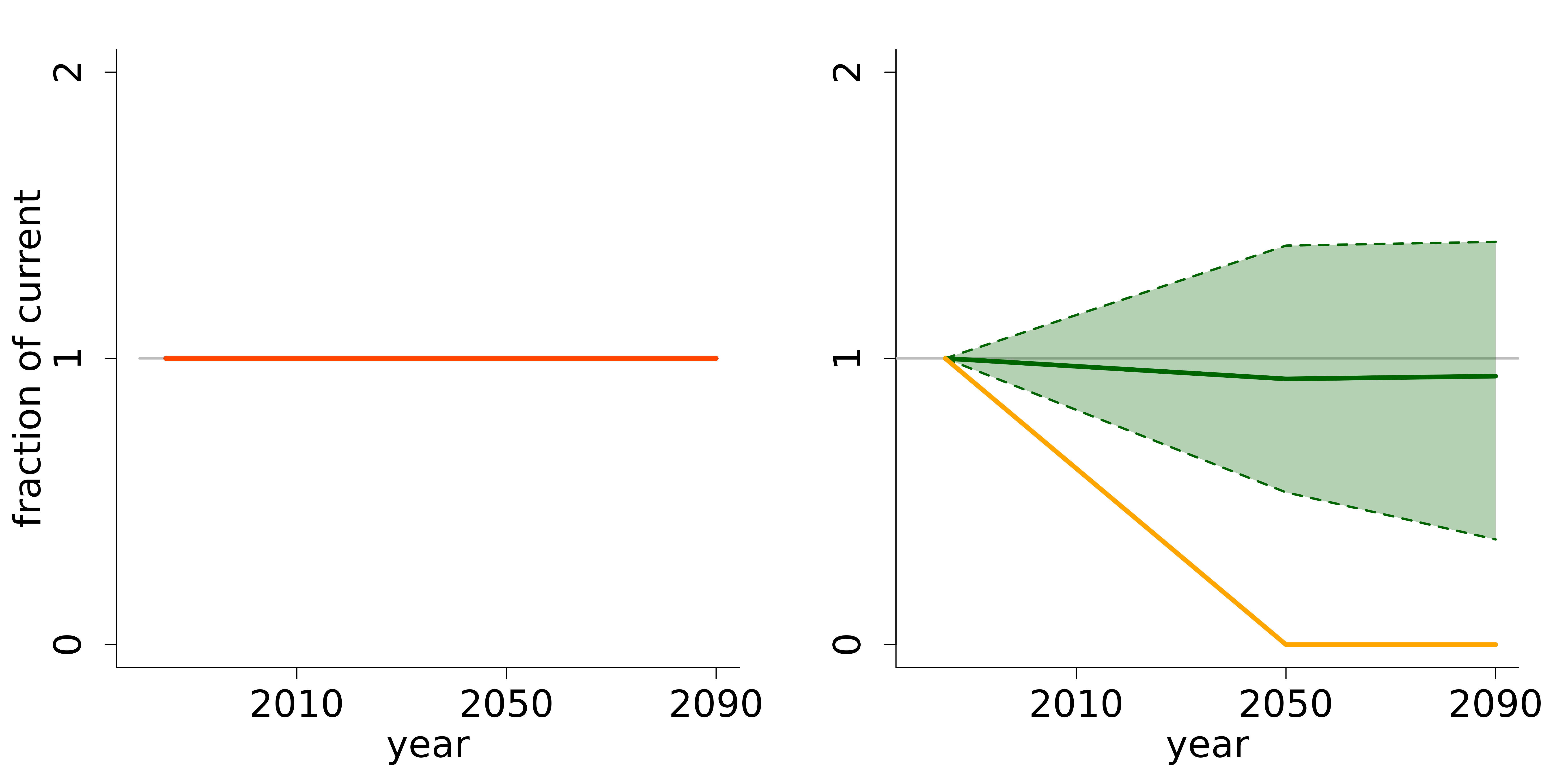

Supplement: S2 Appendix — (ZIP) [file pntd.0014030.s006.zip › Sup. Mat. 6-1 A-L - Species Trends/Crotalus_catalinensis_CCTrends.png]

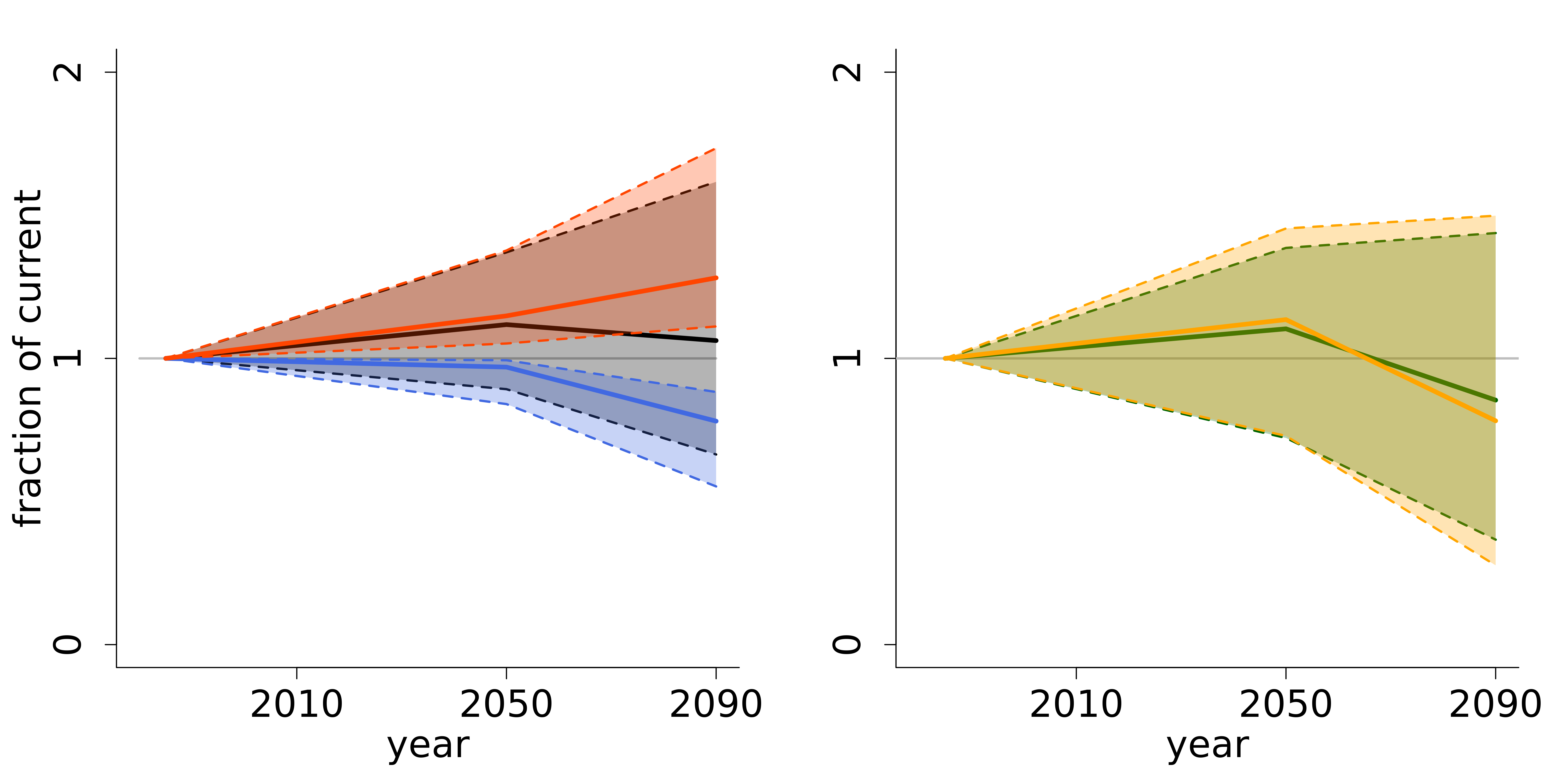

Supplement: S2 Appendix — (ZIP) [file pntd.0014030.s006.zip › Sup. Mat. 6-1 A-L - Species Trends/Crotalus_cerastes_CCTrends.png]

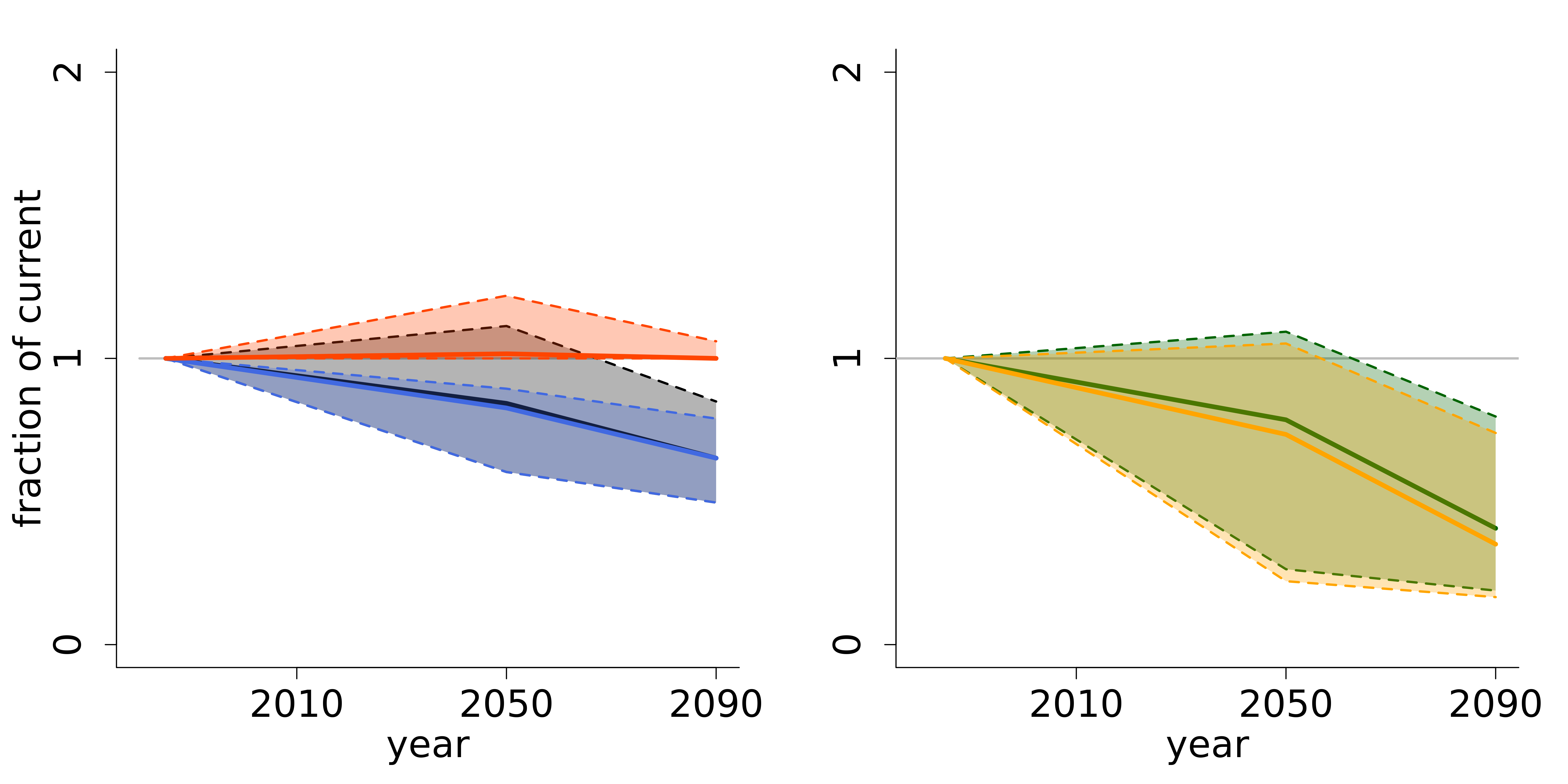

Supplement: S2 Appendix — (ZIP) [file pntd.0014030.s006.zip › Sup. Mat. 6-1 A-L - Species Trends/Crotalus_cerberus_CCTrends.png]

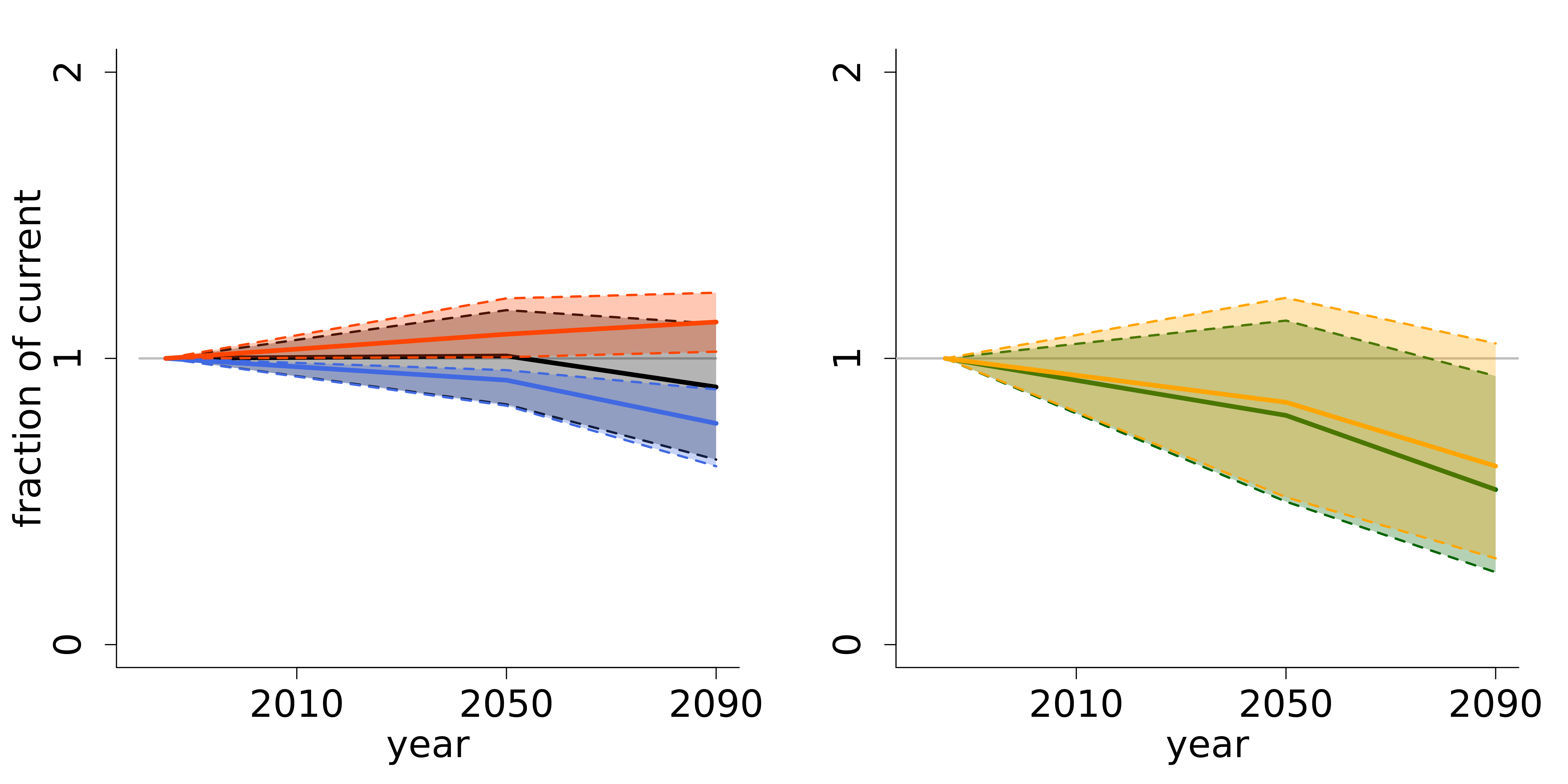

Supplement: S2 Appendix — (ZIP) [file pntd.0014030.s006.zip › Sup. Mat. 6-1 A-L - Species Trends/Crotalus_culminatus_CCTrends.png]

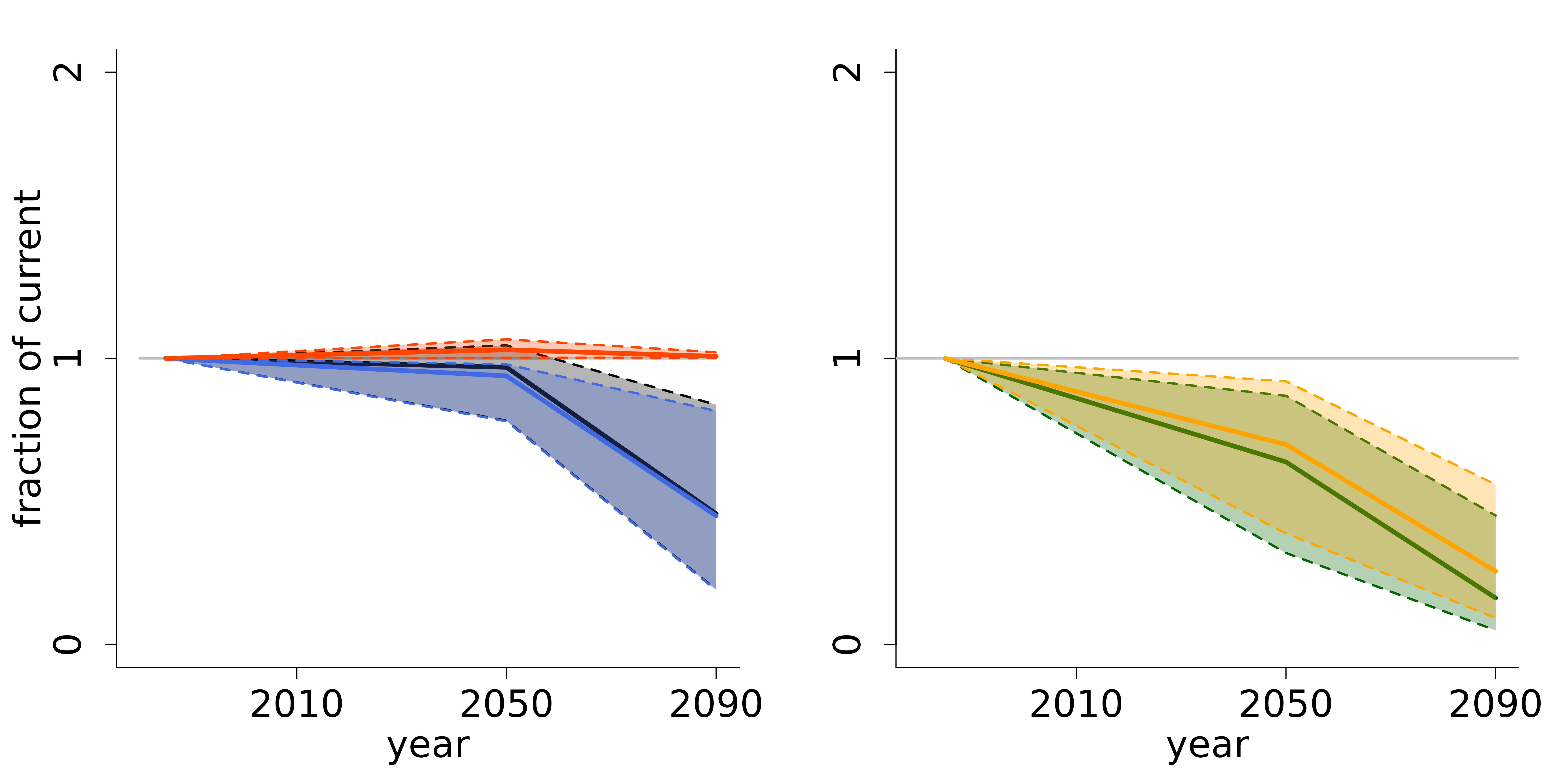

Supplement: S2 Appendix — (ZIP) [file pntd.0014030.s006.zip › Sup. Mat. 6-1 A-L - Species Trends/Crotalus_durissus_CCTrends.png]

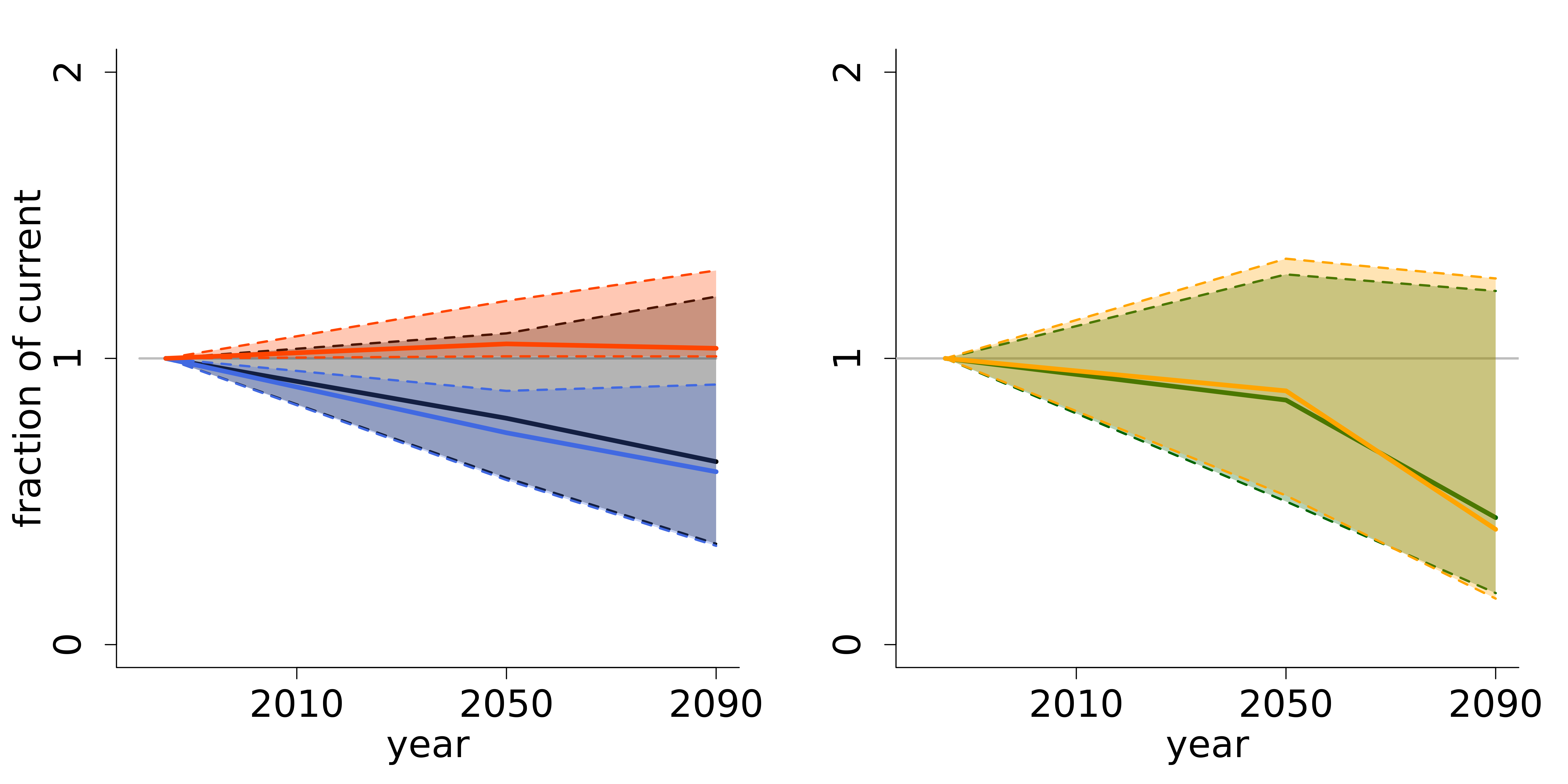

Supplement: S2 Appendix — (ZIP) [file pntd.0014030.s006.zip › Sup. Mat. 6-1 A-L - Species Trends/Crotalus_ehecatl_CCTrends.png]

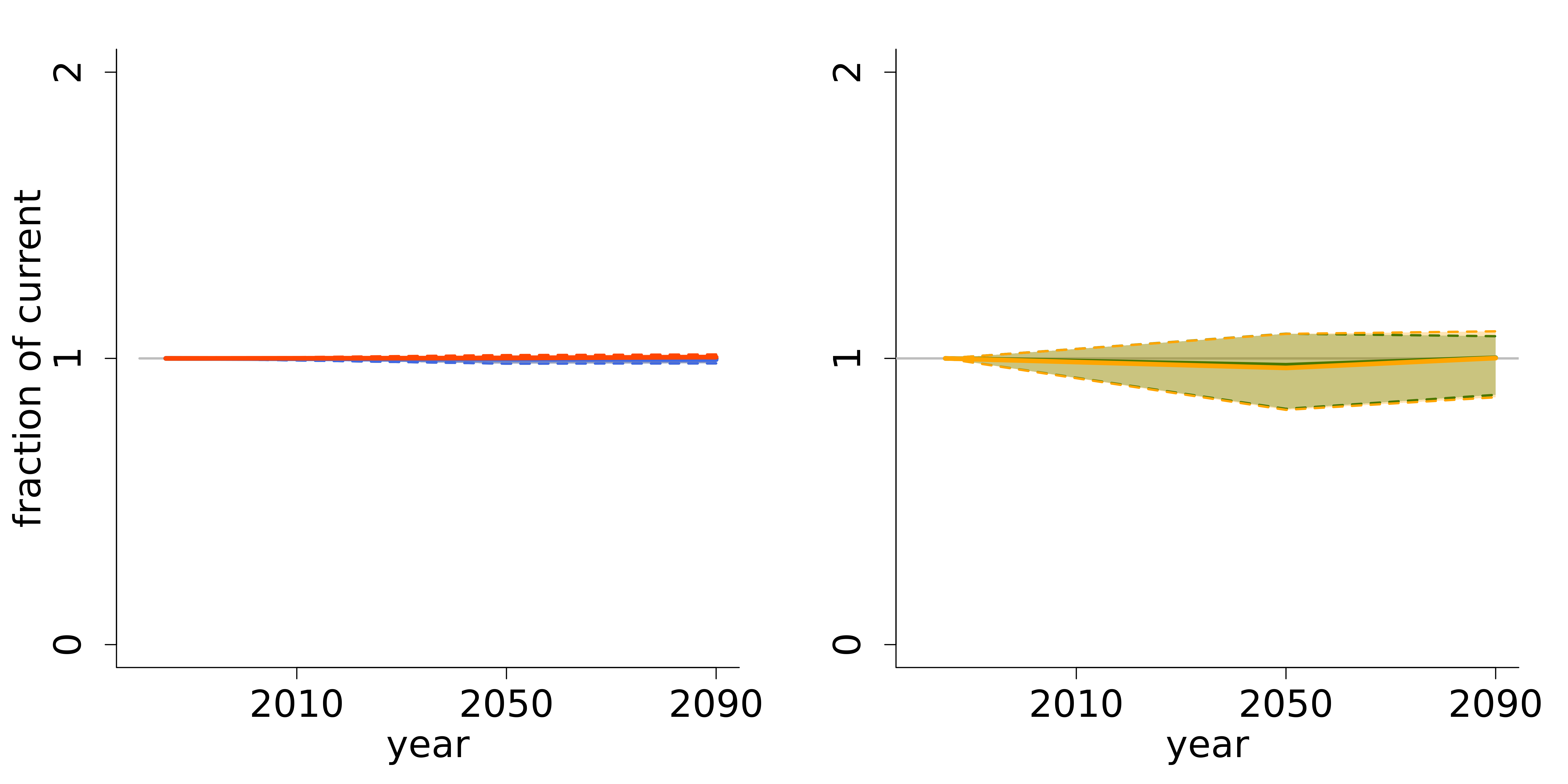

Supplement: S2 Appendix — (ZIP) [file pntd.0014030.s006.zip › Sup. Mat. 6-1 A-L - Species Trends/Crotalus_enyo_CCTrends.png]

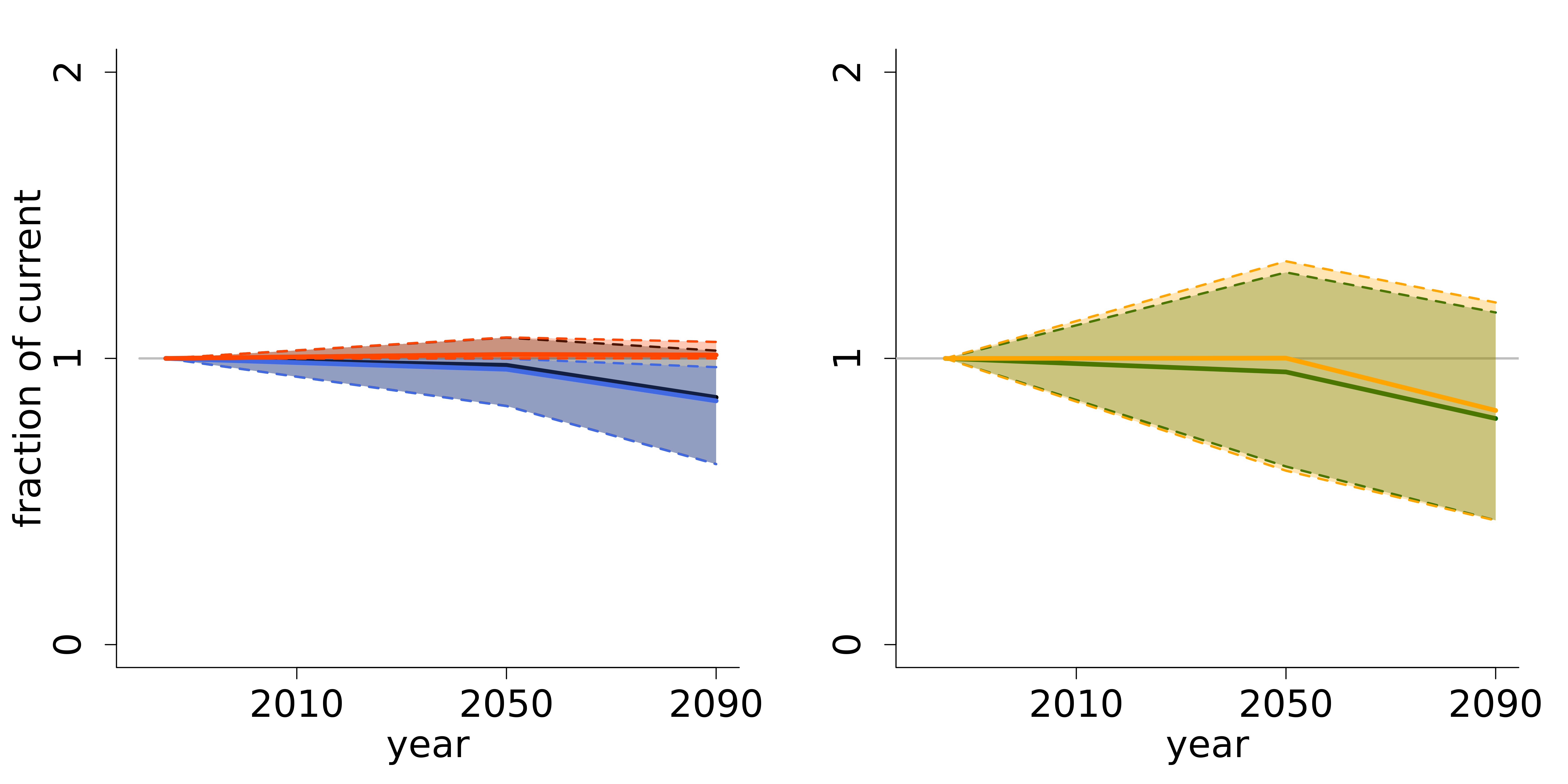

Supplement: S2 Appendix — (ZIP) [file pntd.0014030.s006.zip › Sup. Mat. 6-1 A-L - Species Trends/Crotalus_ericsmithi_CCTrends.png]

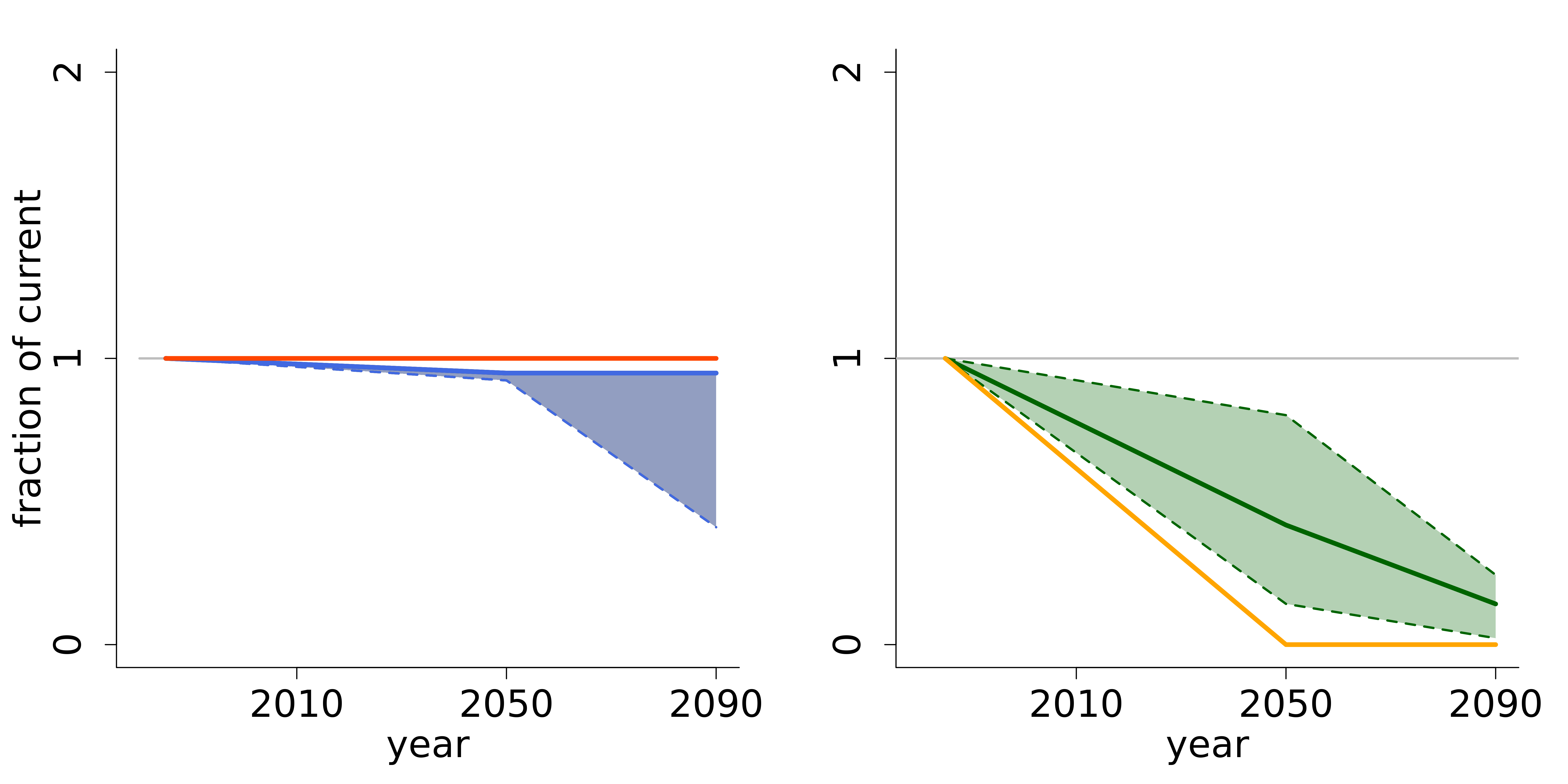

Supplement: S2 Appendix — (ZIP) [file pntd.0014030.s006.zip › Sup. Mat. 6-1 A-L - Species Trends/Crotalus_estebanensis_CCTrends.png]

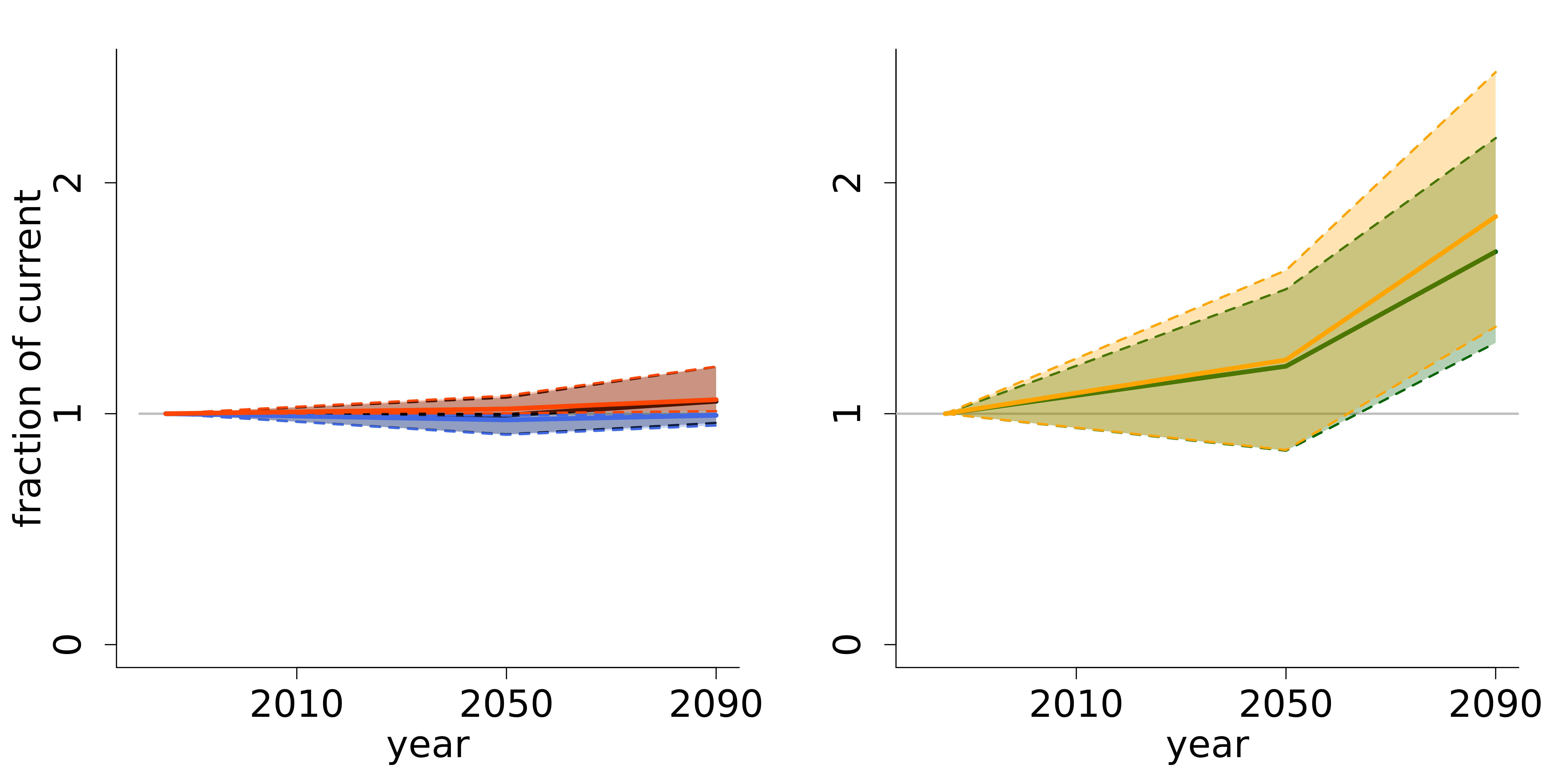

Supplement: S2 Appendix — (ZIP) [file pntd.0014030.s006.zip › Sup. Mat. 6-1 A-L - Species Trends/Crotalus_horridus_CCTrends.png]

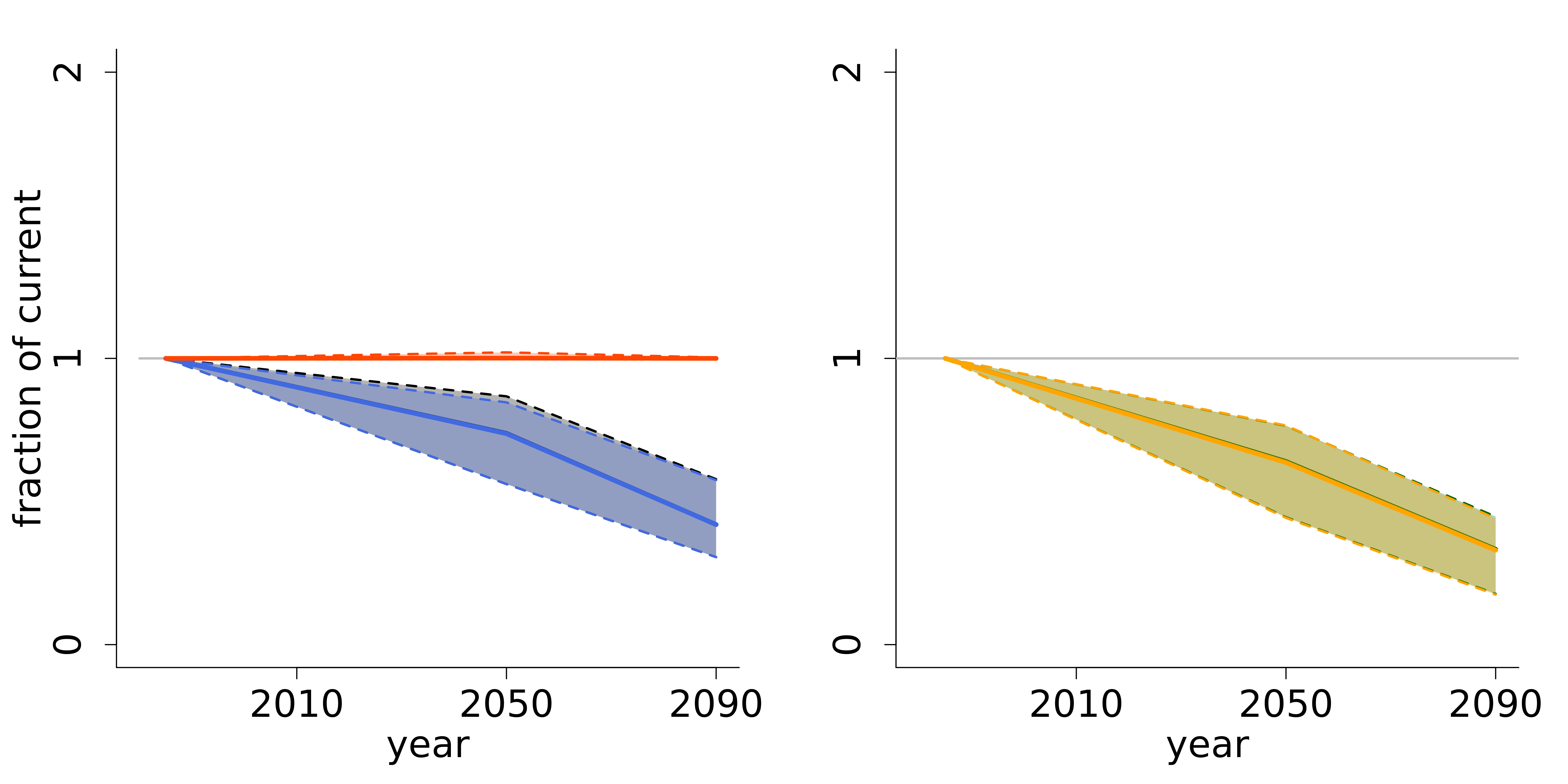

Supplement: S2 Appendix — (ZIP) [file pntd.0014030.s006.zip › Sup. Mat. 6-1 A-L - Species Trends/Crotalus_intermedius_CCTrends.png]

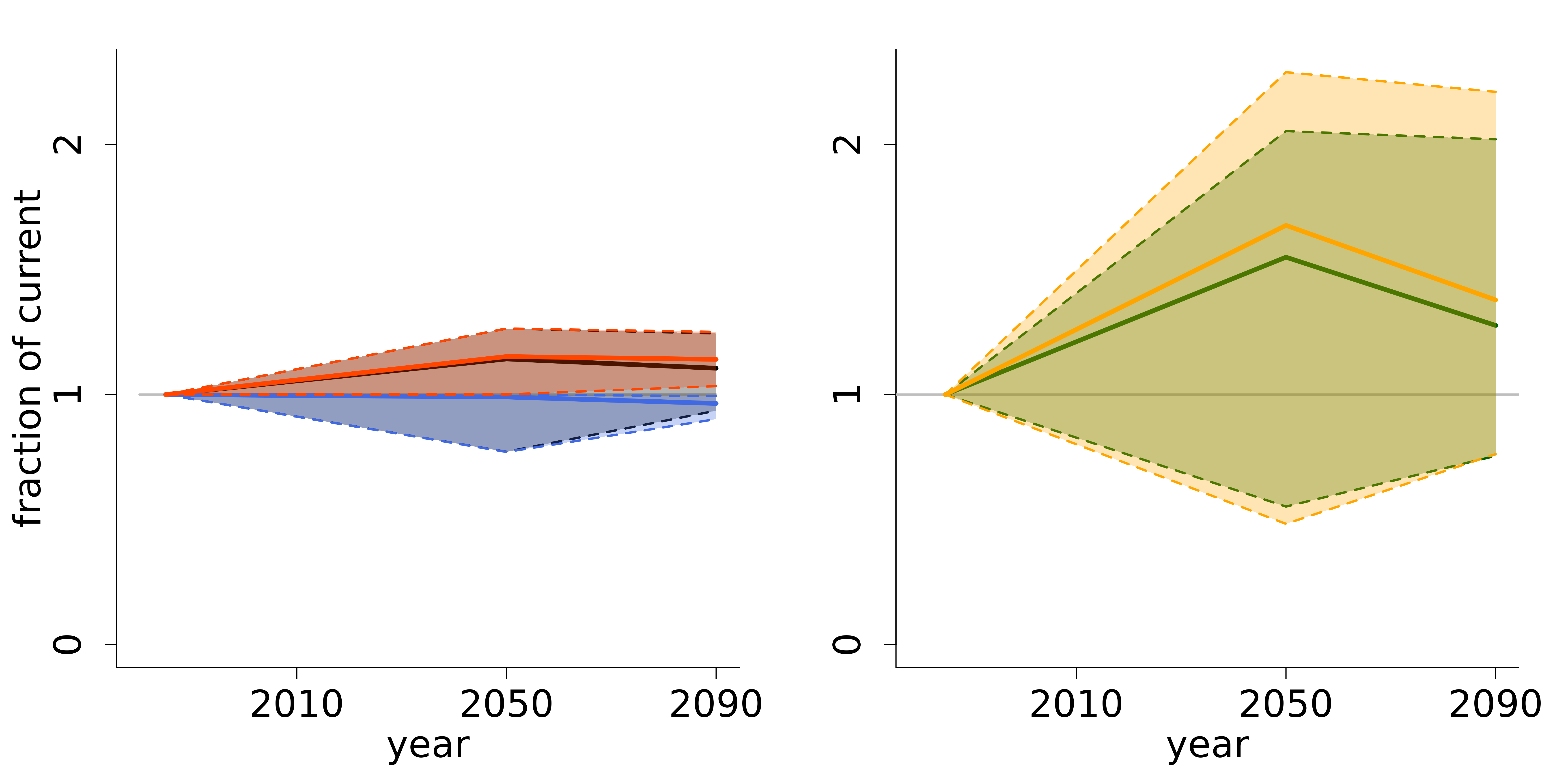

Supplement: S2 Appendix — (ZIP) [file pntd.0014030.s006.zip › Sup. Mat. 6-1 A-L - Species Trends/Crotalus_lannomi_CCTrends.png]

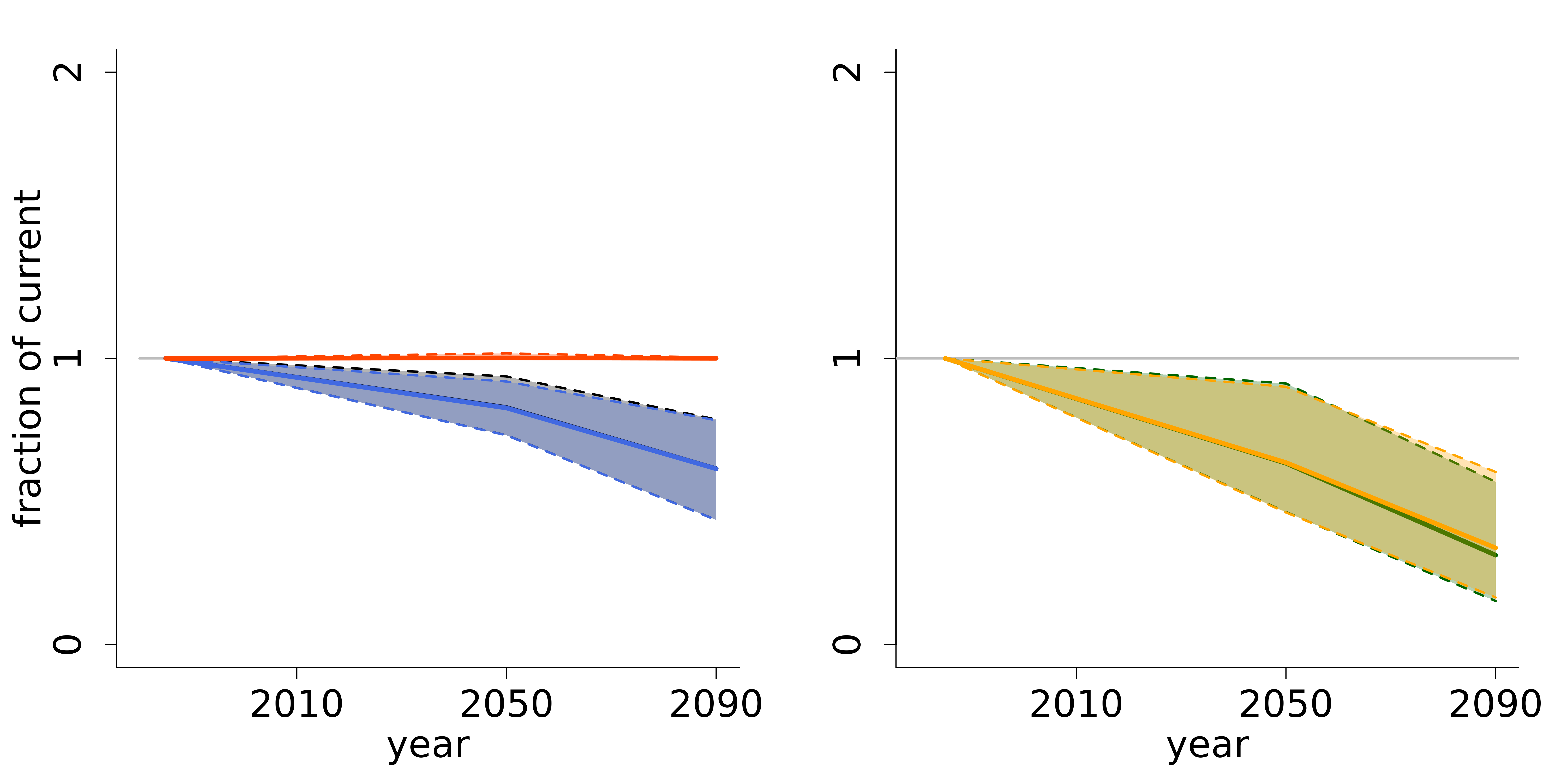

Supplement: S2 Appendix — (ZIP) [file pntd.0014030.s006.zip › Sup. Mat. 6-1 A-L - Species Trends/Crotalus_lepidus_CCTrends.png]

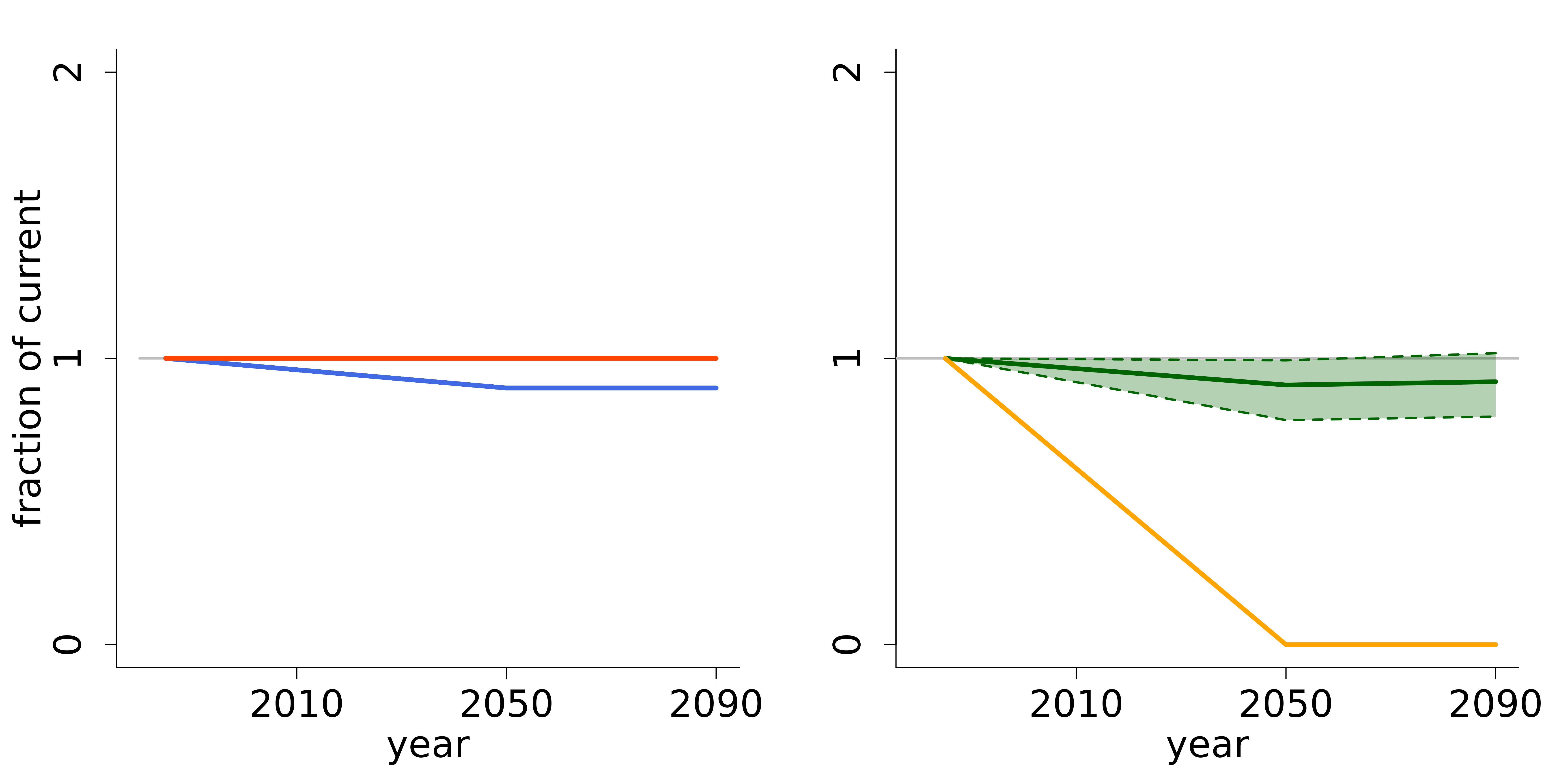

Supplement: S2 Appendix — (ZIP) [file pntd.0014030.s006.zip › Sup. Mat. 6-1 A-L - Species Trends/Crotalus_lorenzoensis_CCTrends.png]

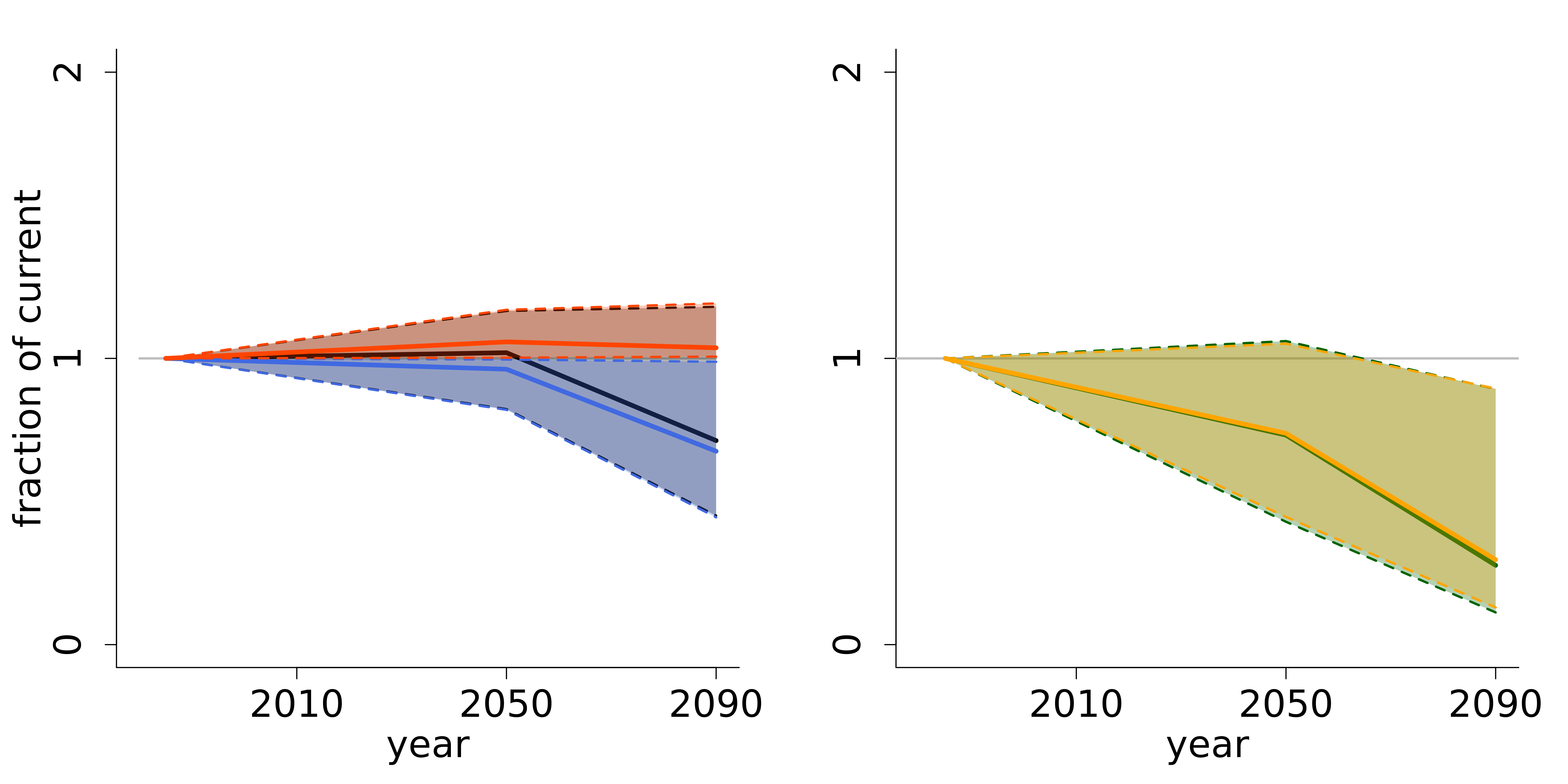

Supplement: S2 Appendix — (ZIP) [file pntd.0014030.s006.zip › Sup. Mat. 6-1 A-L - Species Trends/Crotalus_mictlantecuhtli_CCTrends.png]

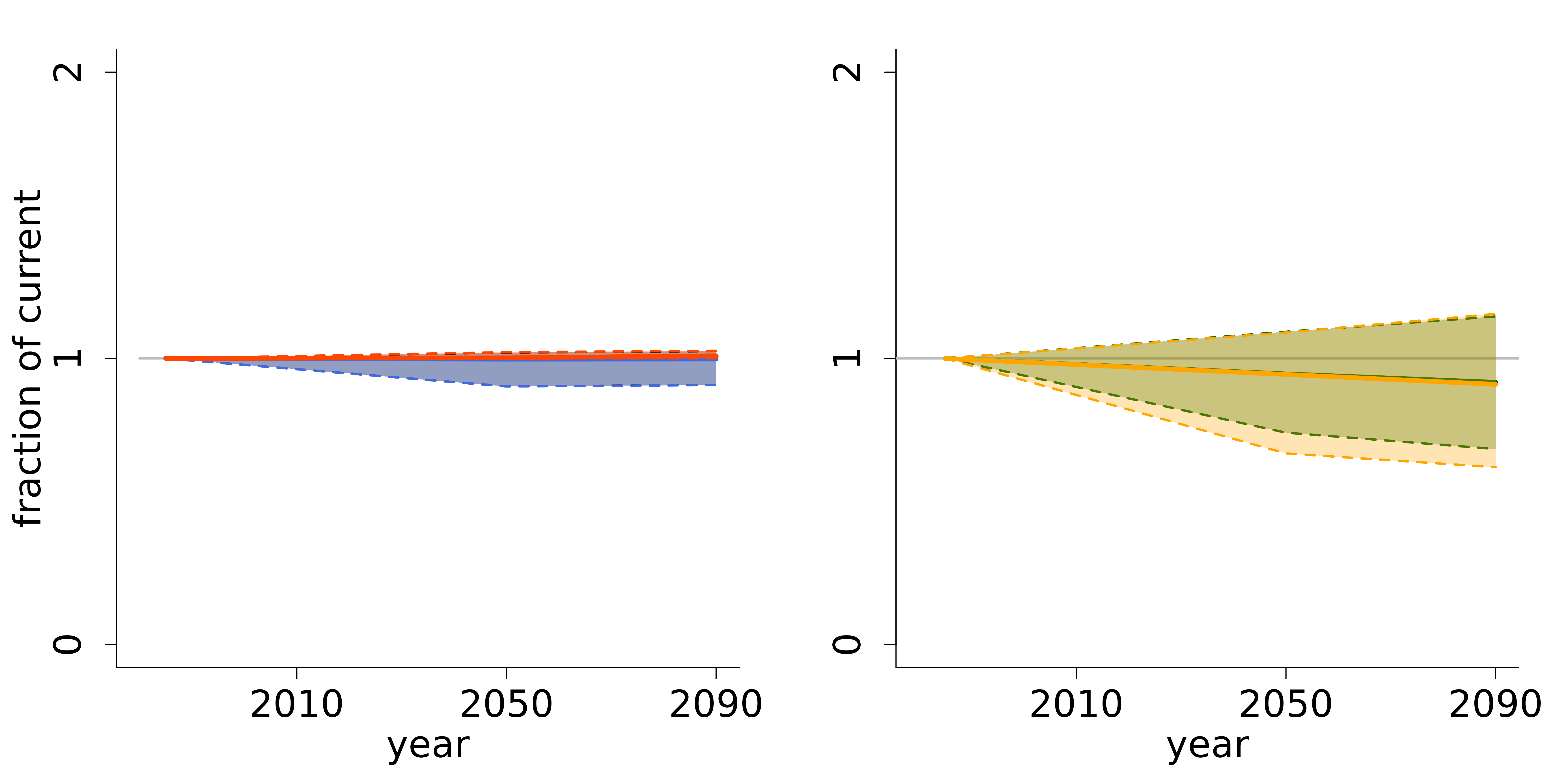

Supplement: S2 Appendix — (ZIP) [file pntd.0014030.s006.zip › Sup. Mat. 6-1 A-L - Species Trends/Crotalus_mitchellii_CCTrends.png]

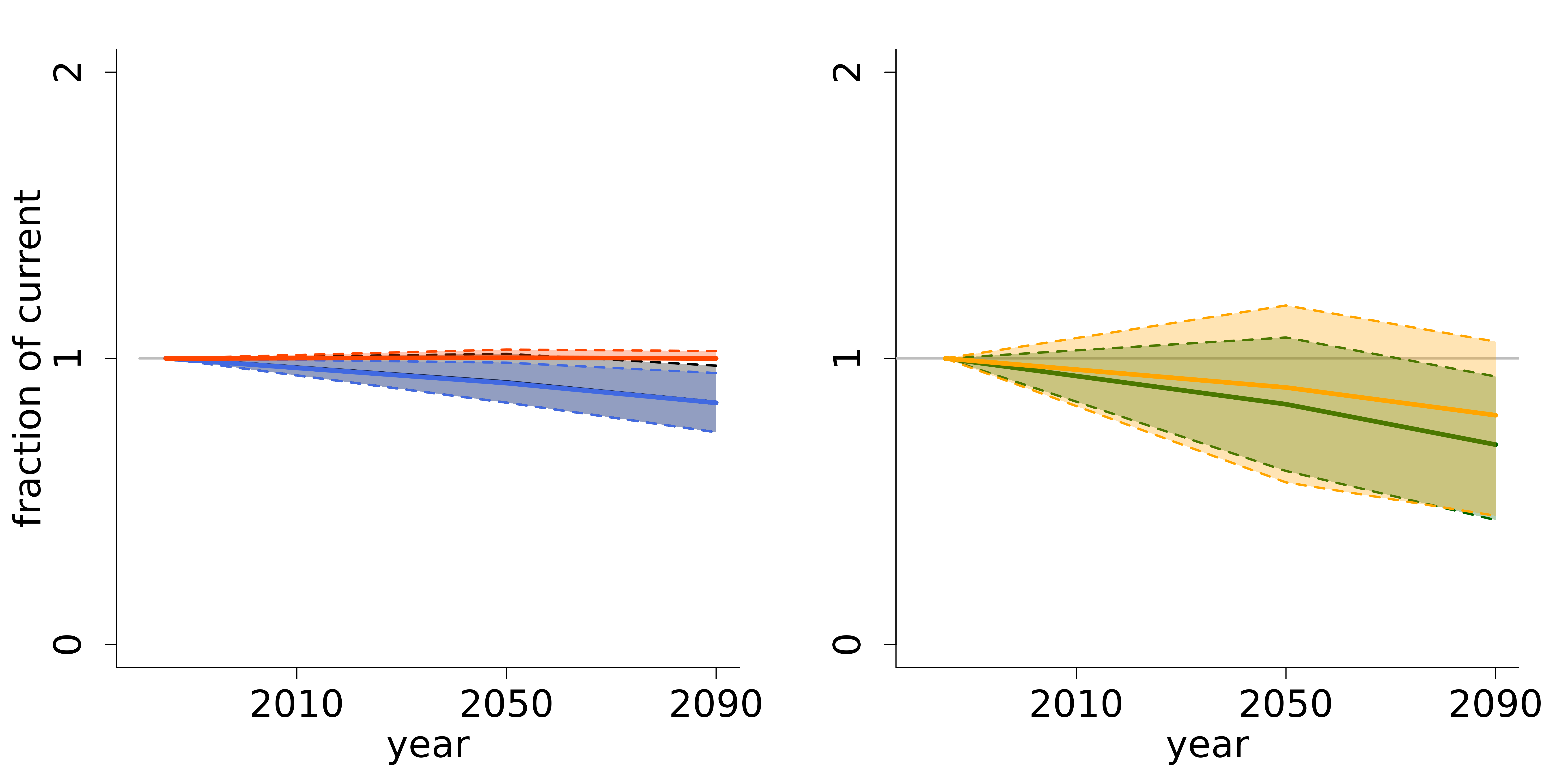

Supplement: S2 Appendix — (ZIP) [file pntd.0014030.s006.zip › Sup. Mat. 6-1 A-L - Species Trends/Crotalus_molossus_CCTrends.png]

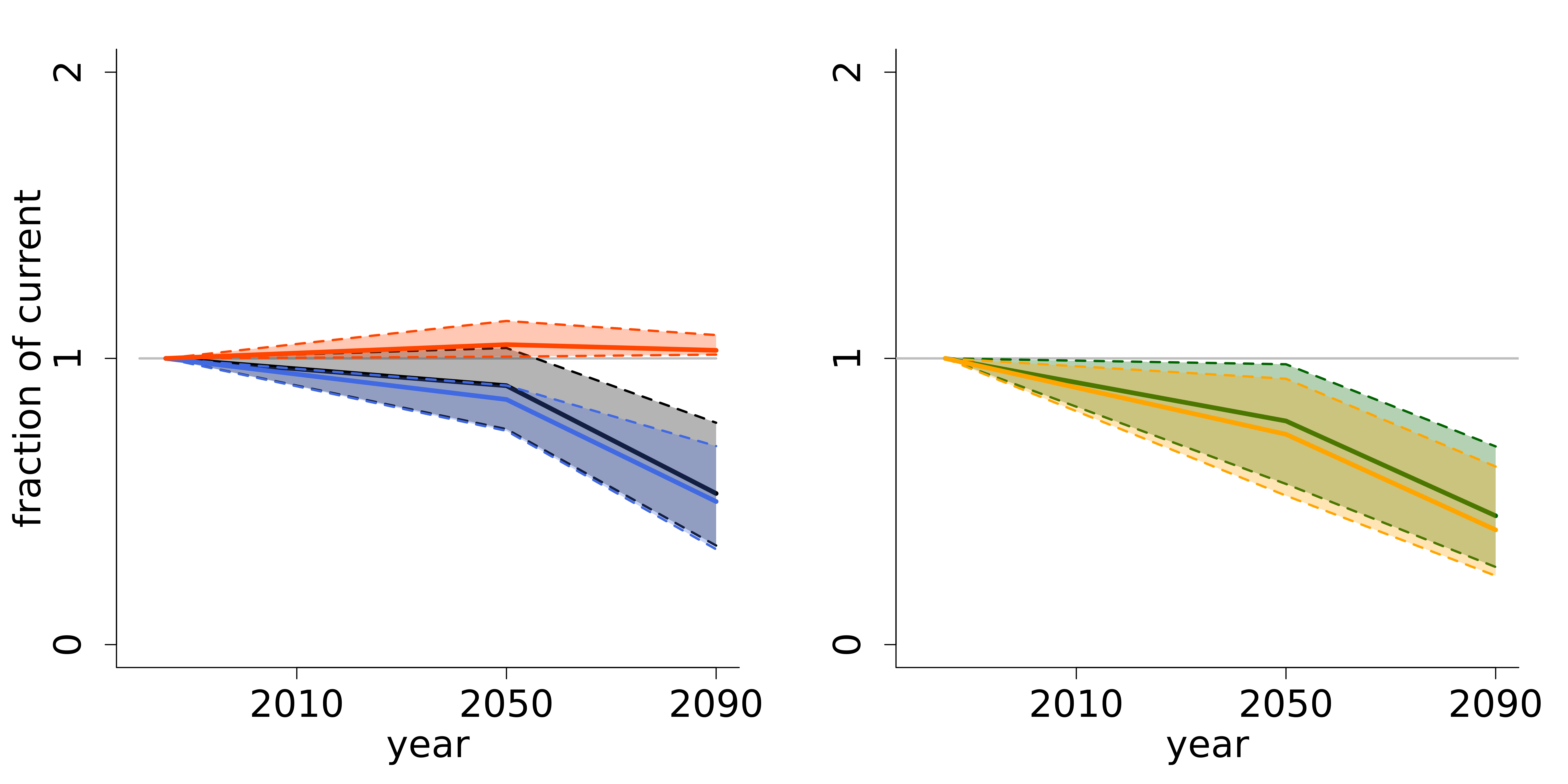

Supplement: S2 Appendix — (ZIP) [file pntd.0014030.s006.zip › Sup. Mat. 6-1 A-L - Species Trends/Crotalus_morulus_CCTrends.png]

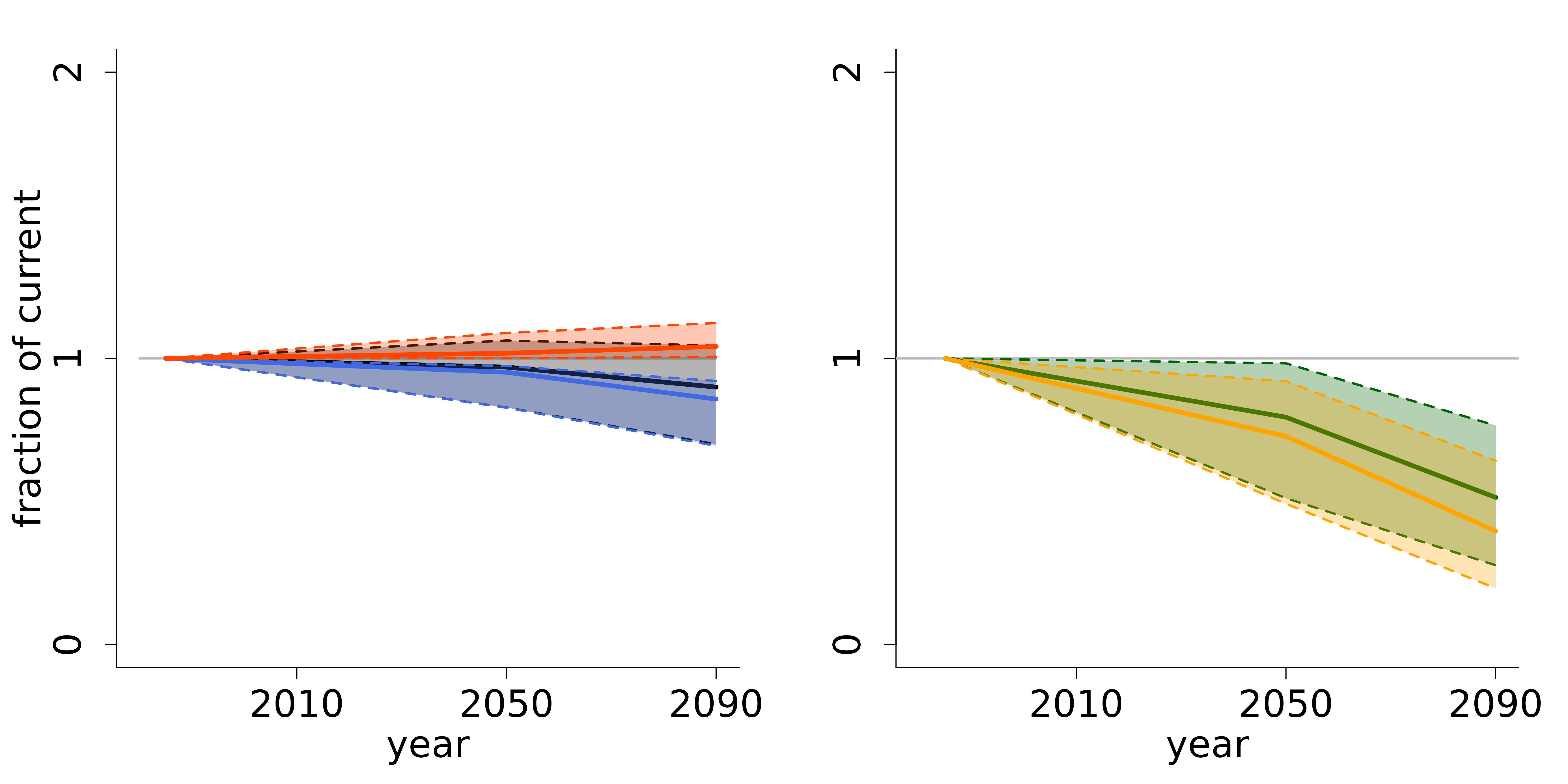

Supplement: S2 Appendix — (ZIP) [file pntd.0014030.s006.zip › Sup. Mat. 6-1 A-L - Species Trends/Crotalus_oreganus_CCTrends.png]

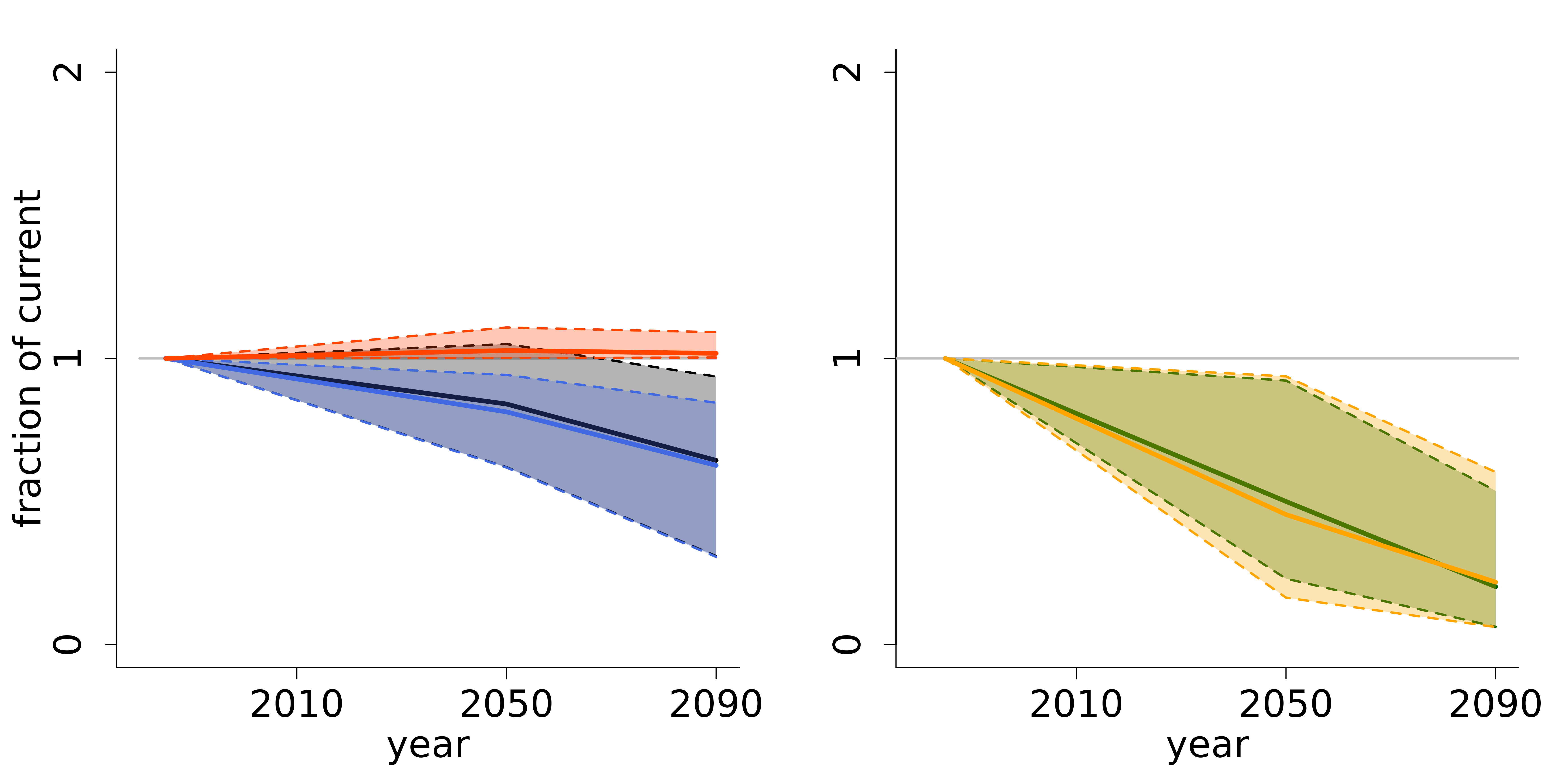

Supplement: S2 Appendix — (ZIP) [file pntd.0014030.s006.zip › Sup. Mat. 6-1 A-L - Species Trends/Crotalus_ornatus_CCTrends.png]

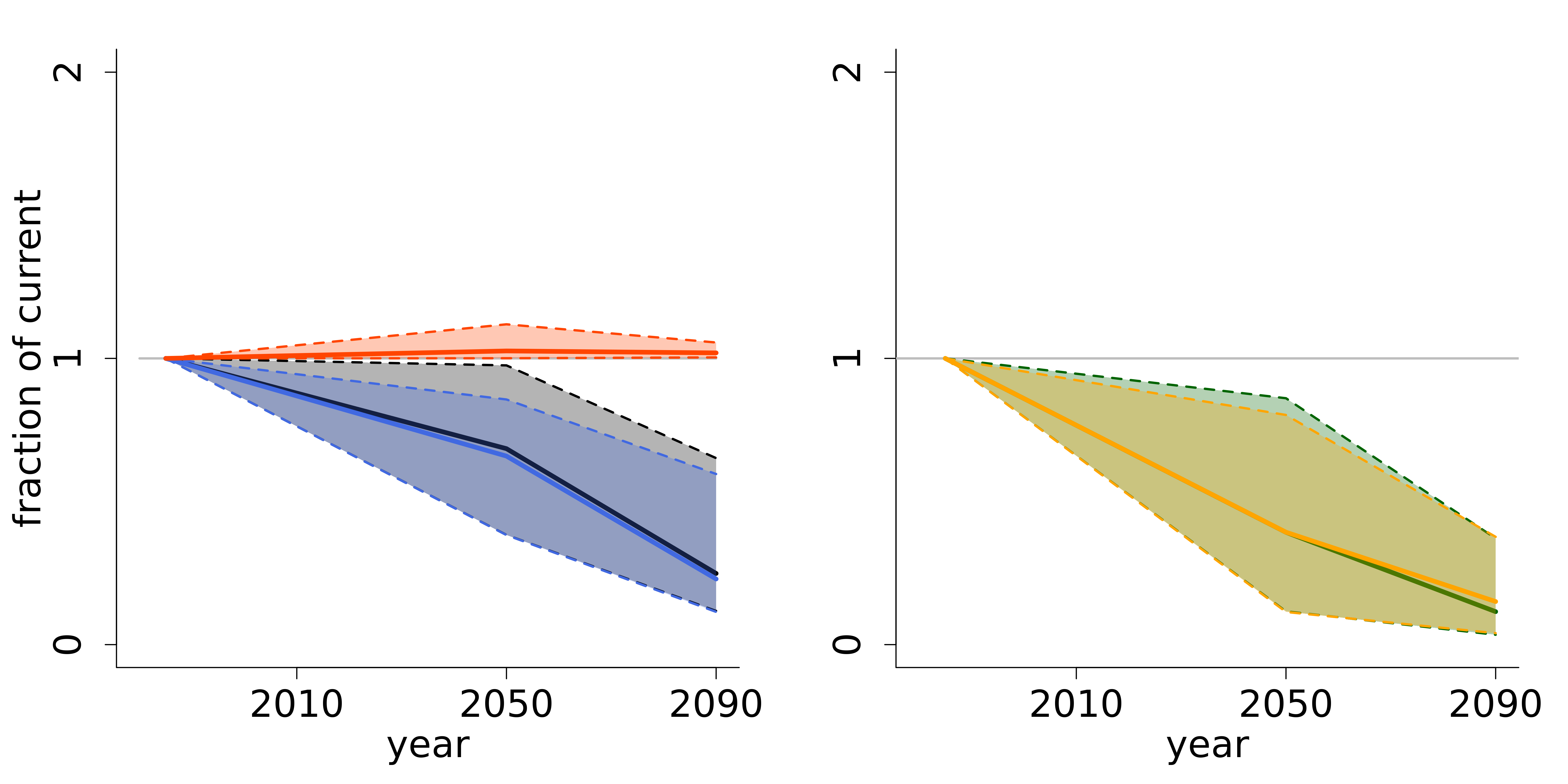

Supplement: S2 Appendix — (ZIP) [file pntd.0014030.s006.zip › Sup. Mat. 6-1 A-L - Species Trends/Crotalus_polystictus_CCTrends.png]

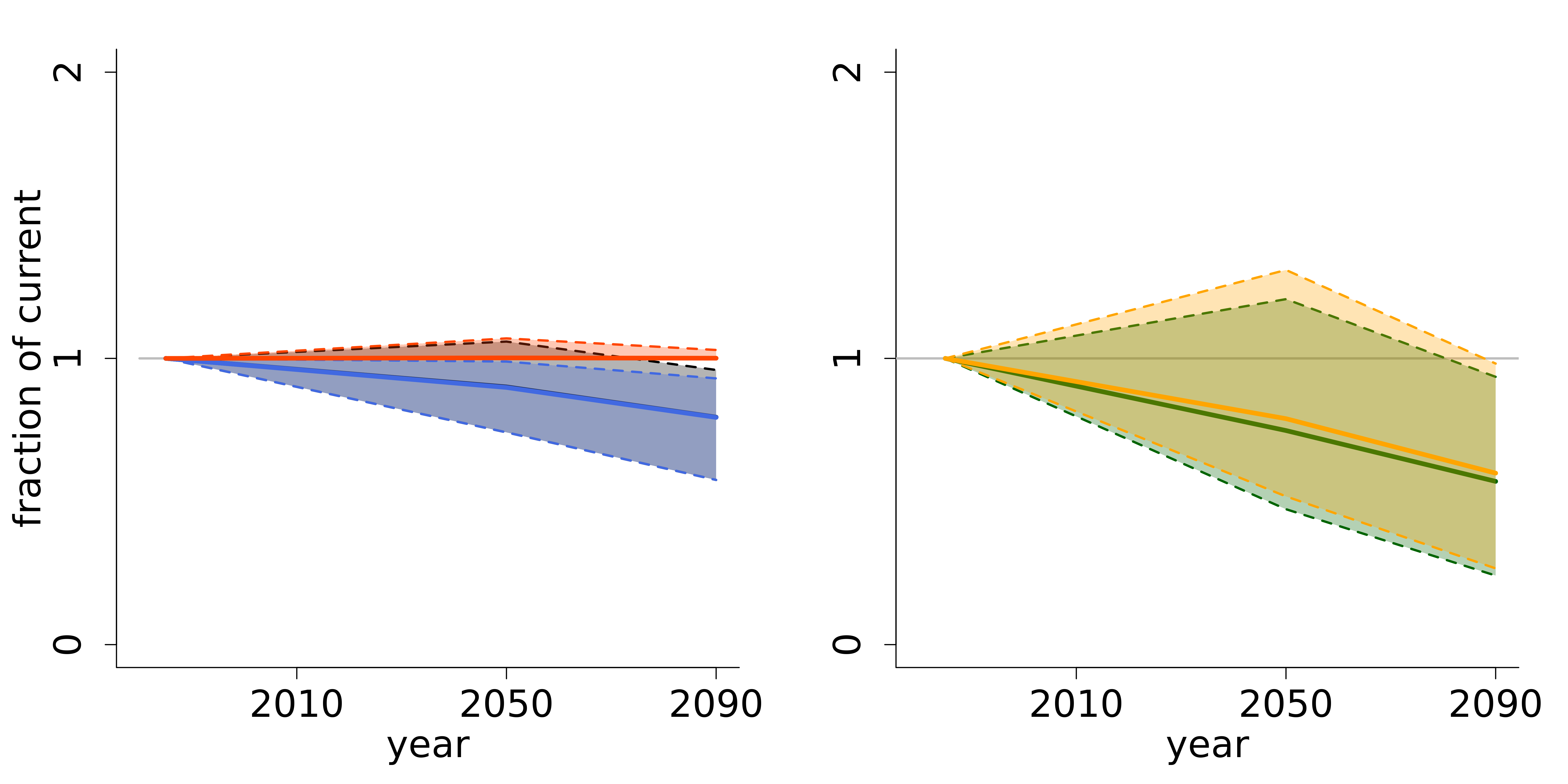

Supplement: S2 Appendix — (ZIP) [file pntd.0014030.s006.zip › Sup. Mat. 6-1 A-L - Species Trends/Crotalus_pricei_CCTrends.png]

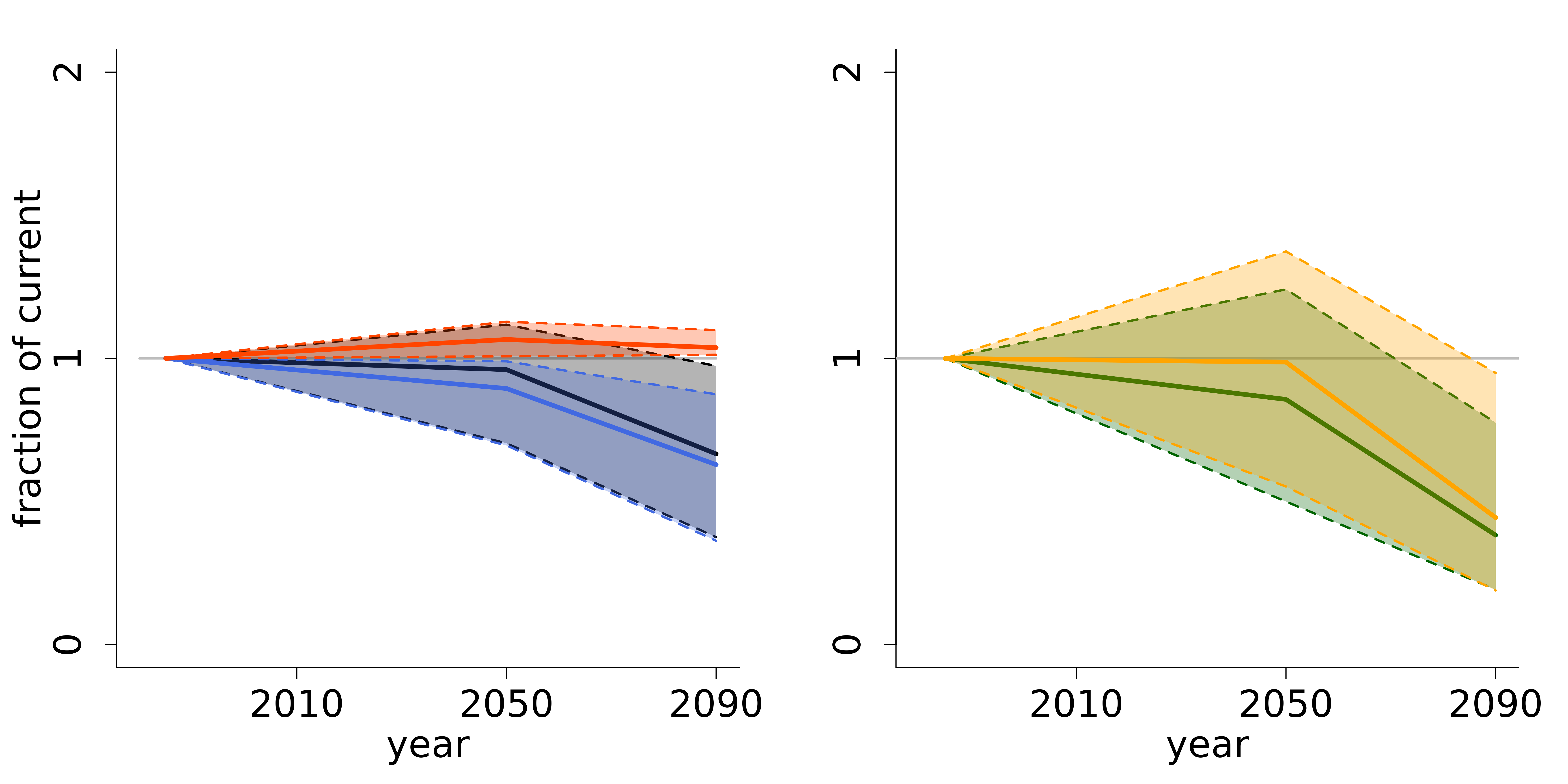

Supplement: S2 Appendix — (ZIP) [file pntd.0014030.s006.zip › Sup. Mat. 6-1 A-L - Species Trends/Crotalus_pusillus_CCTrends.png]

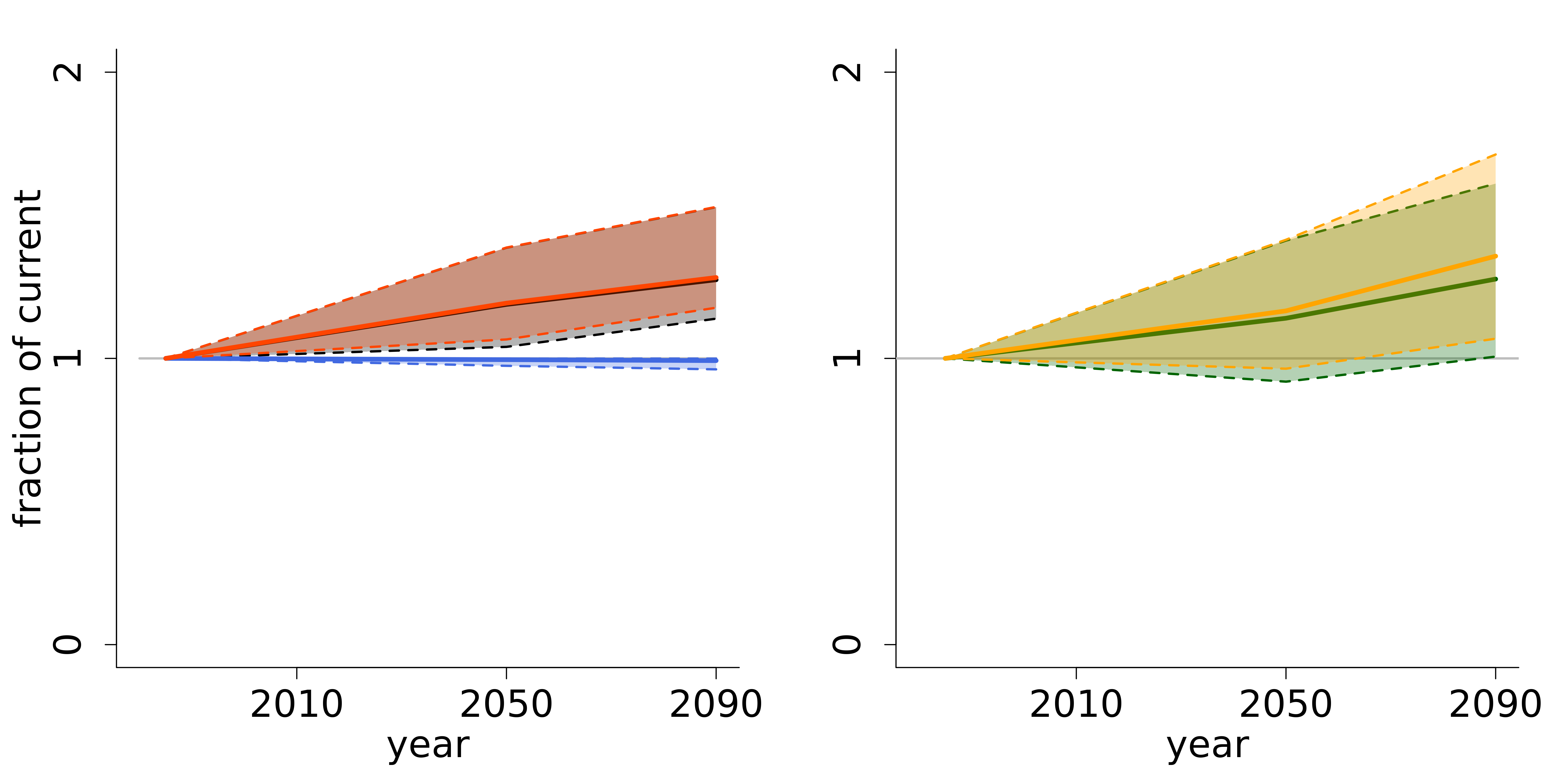

Supplement: S2 Appendix — (ZIP) [file pntd.0014030.s006.zip › Sup. Mat. 6-1 A-L - Species Trends/Crotalus_pyrrhus_CCTrends.png]

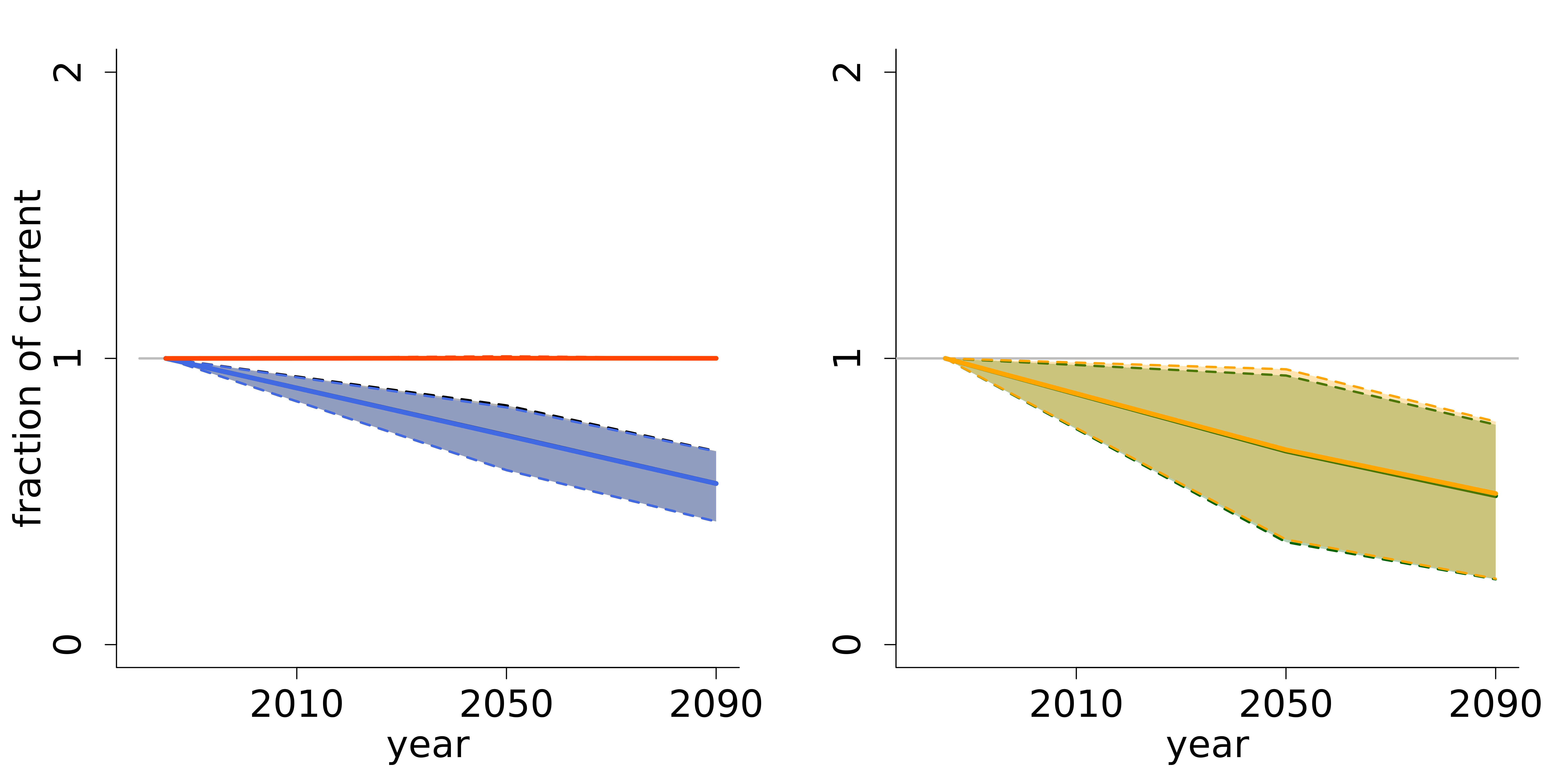

Supplement: S2 Appendix — (ZIP) [file pntd.0014030.s006.zip › Sup. Mat. 6-1 A-L - Species Trends/Crotalus_ravus_CCTrends.png]

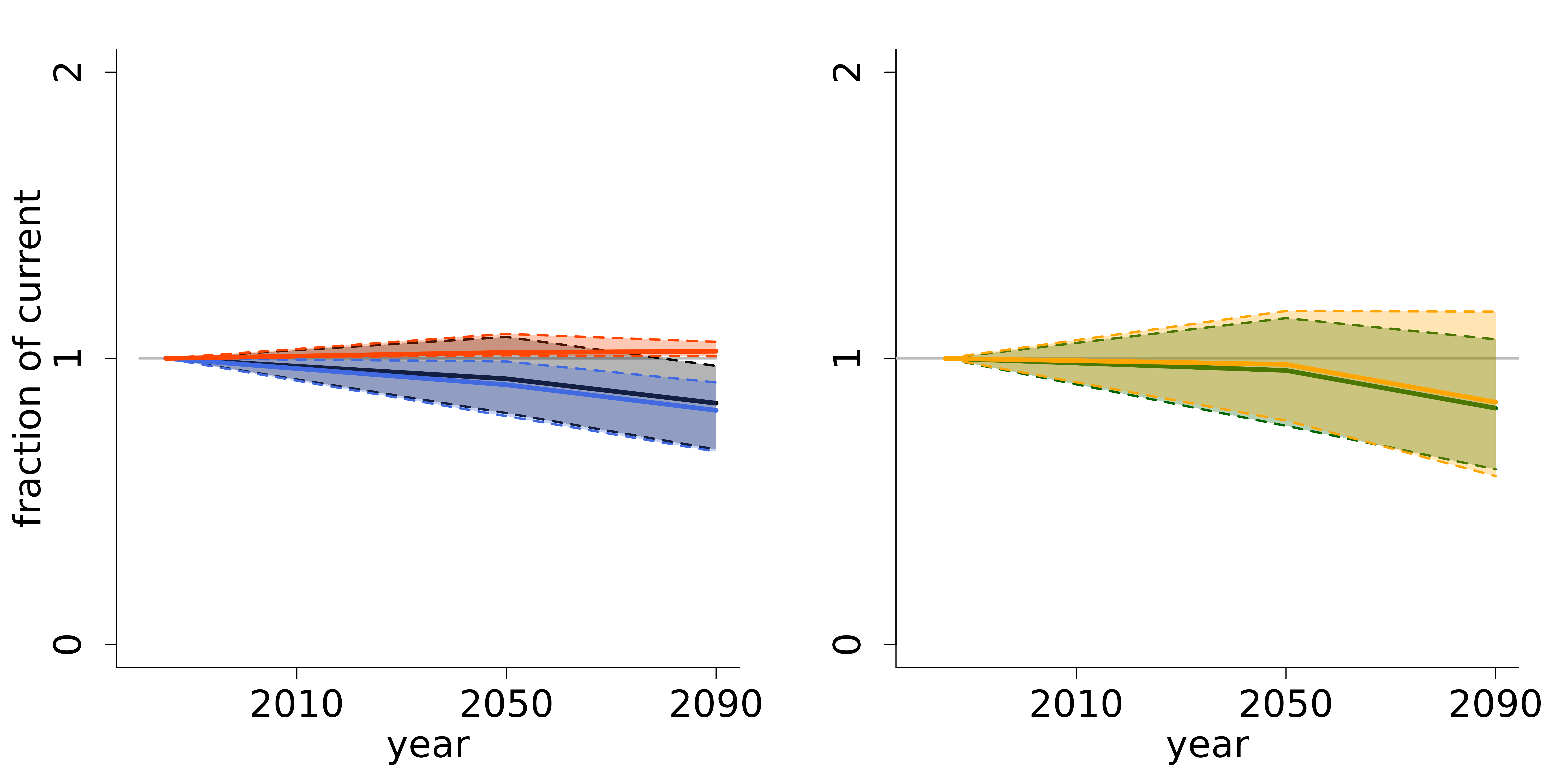

Supplement: S2 Appendix — (ZIP) [file pntd.0014030.s006.zip › Sup. Mat. 6-1 A-L - Species Trends/Crotalus_ruber_CCTrends.png]

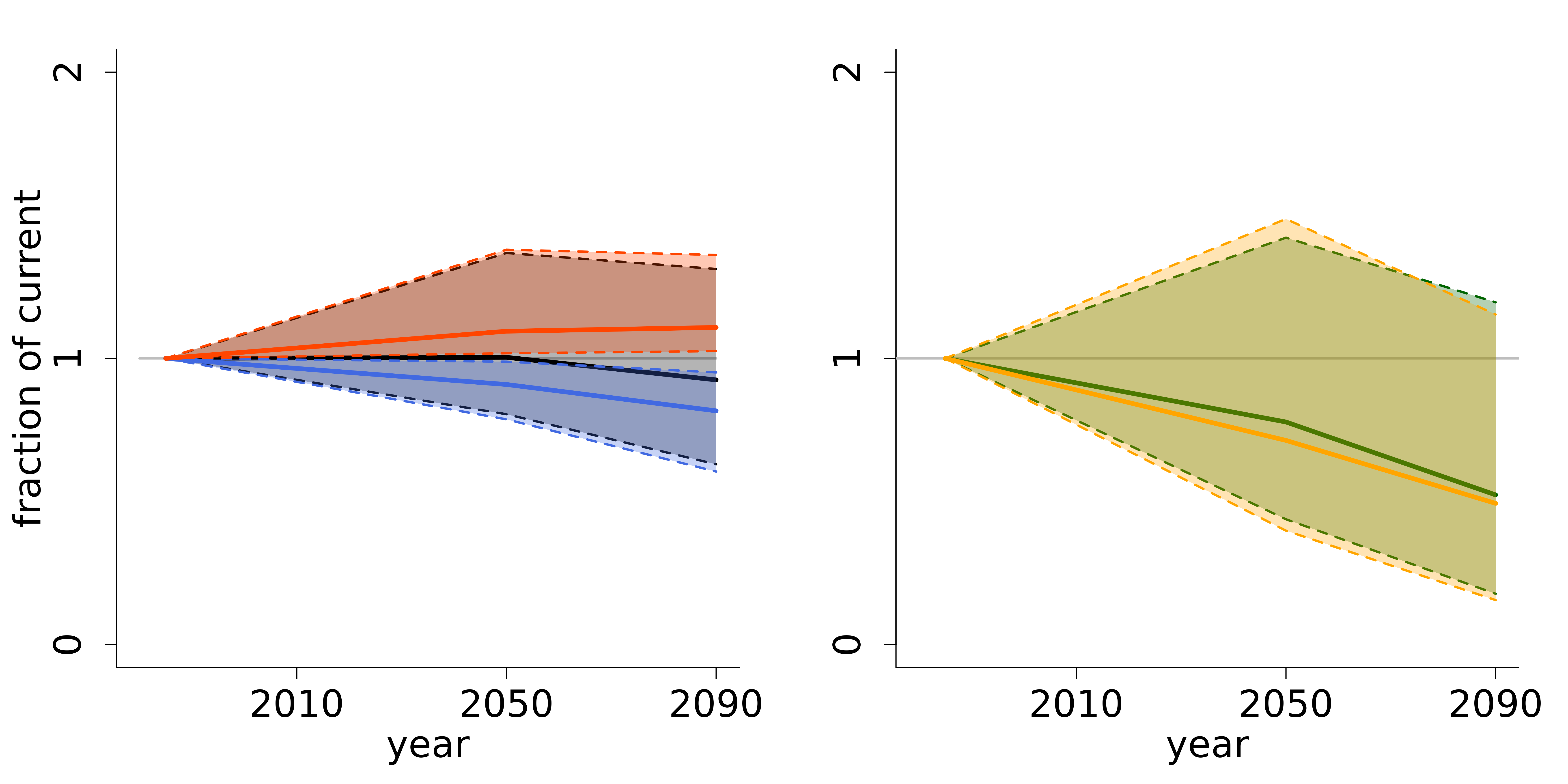

Supplement: S2 Appendix — (ZIP) [file pntd.0014030.s006.zip › Sup. Mat. 6-1 A-L - Species Trends/Crotalus_scutulatus_CCTrends.png]

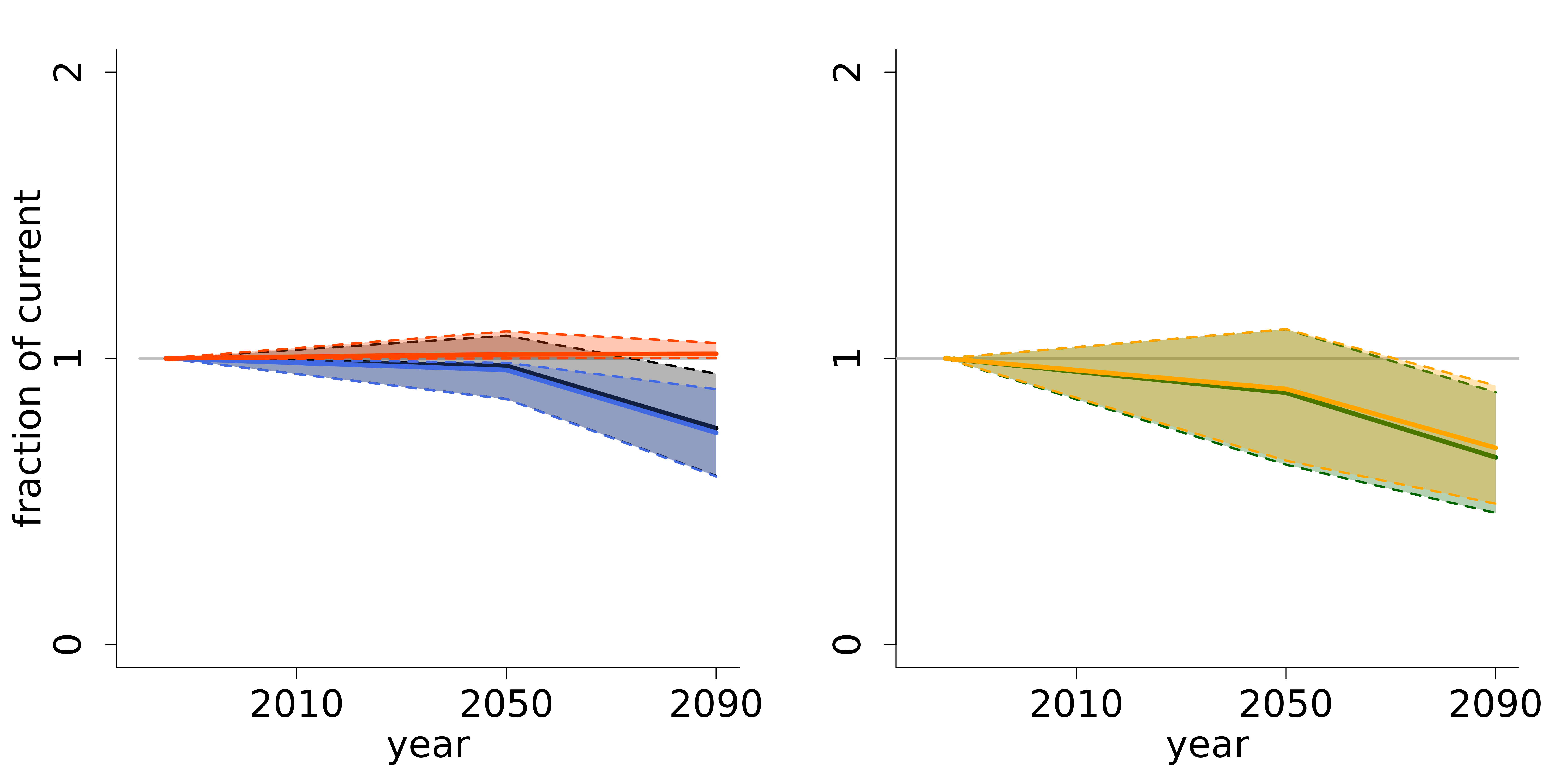

Supplement: S2 Appendix — (ZIP) [file pntd.0014030.s006.zip › Sup. Mat. 6-1 A-L - Species Trends/Crotalus_simus_CCTrends.png]

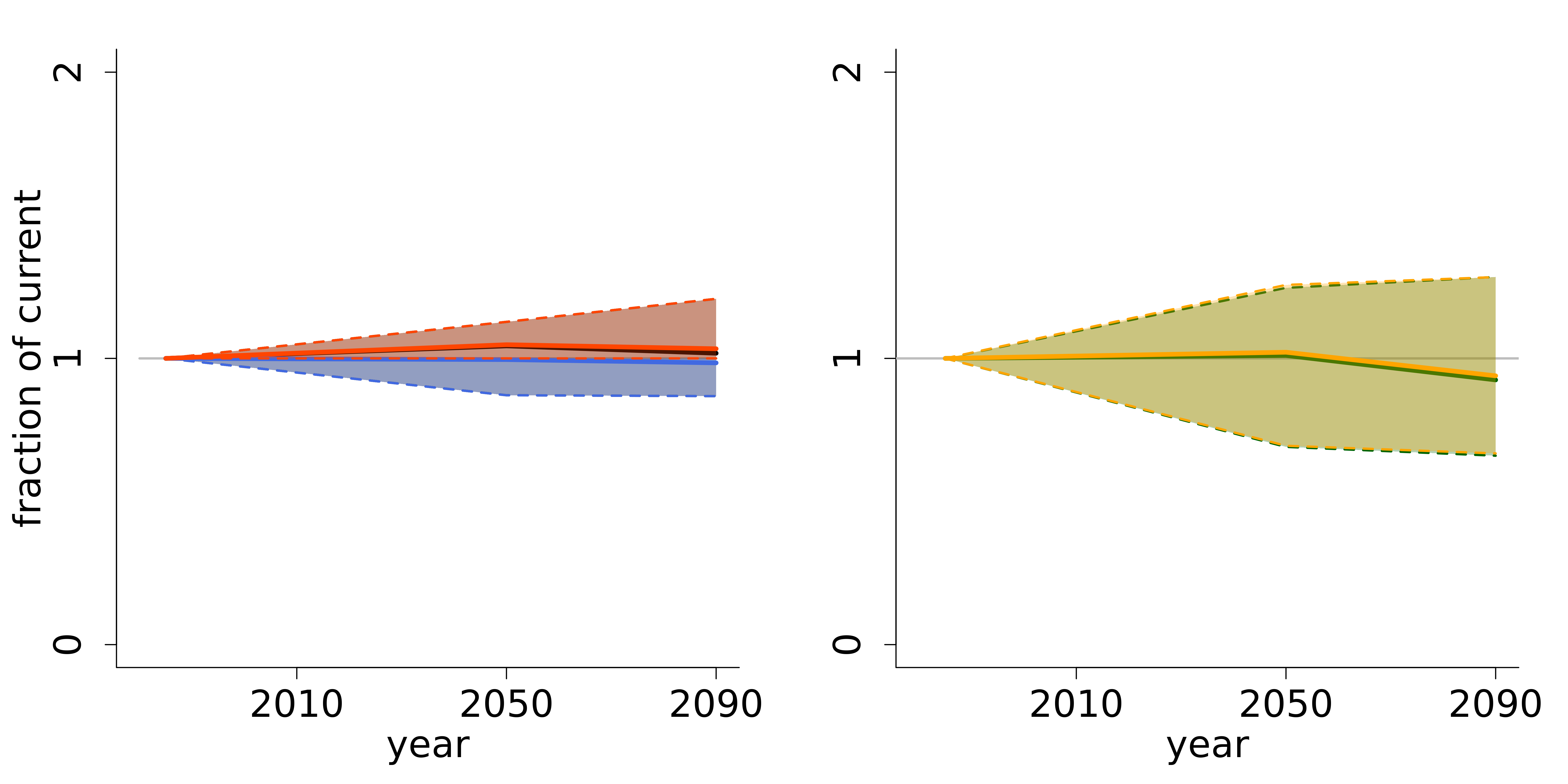

Supplement: S2 Appendix — (ZIP) [file pntd.0014030.s006.zip › Sup. Mat. 6-1 A-L - Species Trends/Crotalus_stejnegeri_CCTrends.png]

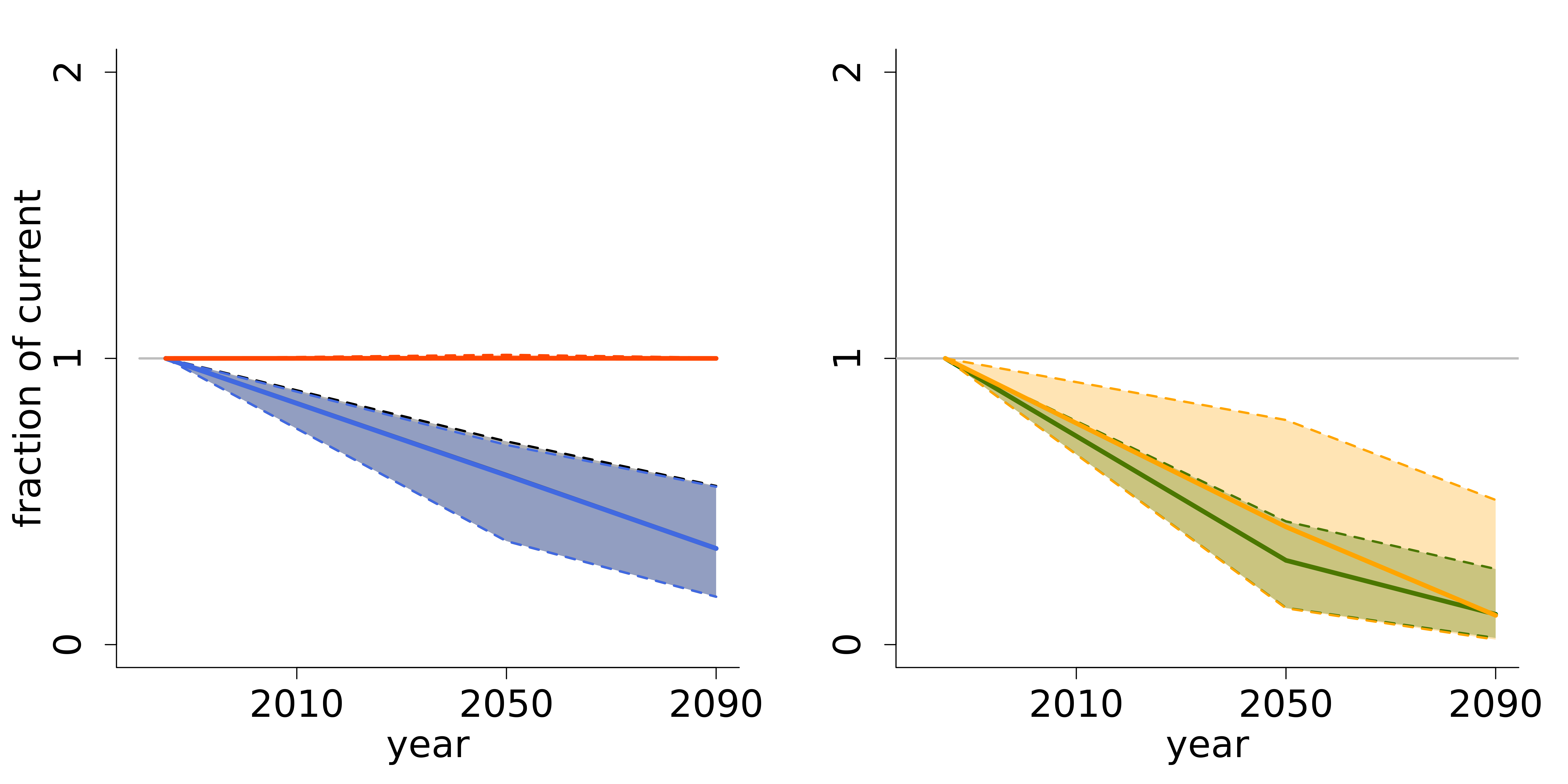

Supplement: S2 Appendix — (ZIP) [file pntd.0014030.s006.zip › Sup. Mat. 6-1 A-L - Species Trends/Crotalus_stephensi_CCTrends.png]

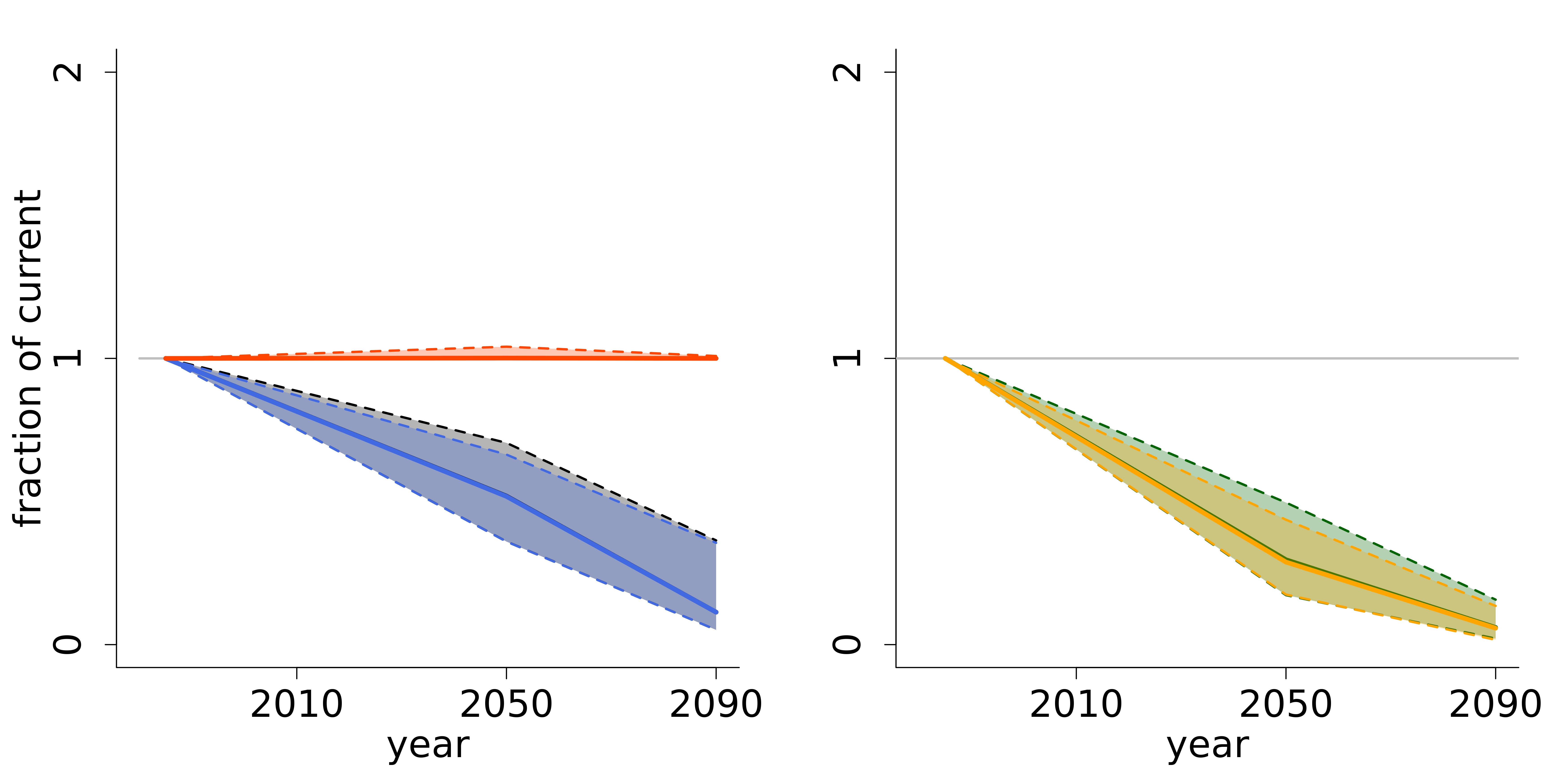

Supplement: S2 Appendix — (ZIP) [file pntd.0014030.s006.zip › Sup. Mat. 6-1 A-L - Species Trends/Crotalus_tancitarensis_CCTrends.png]

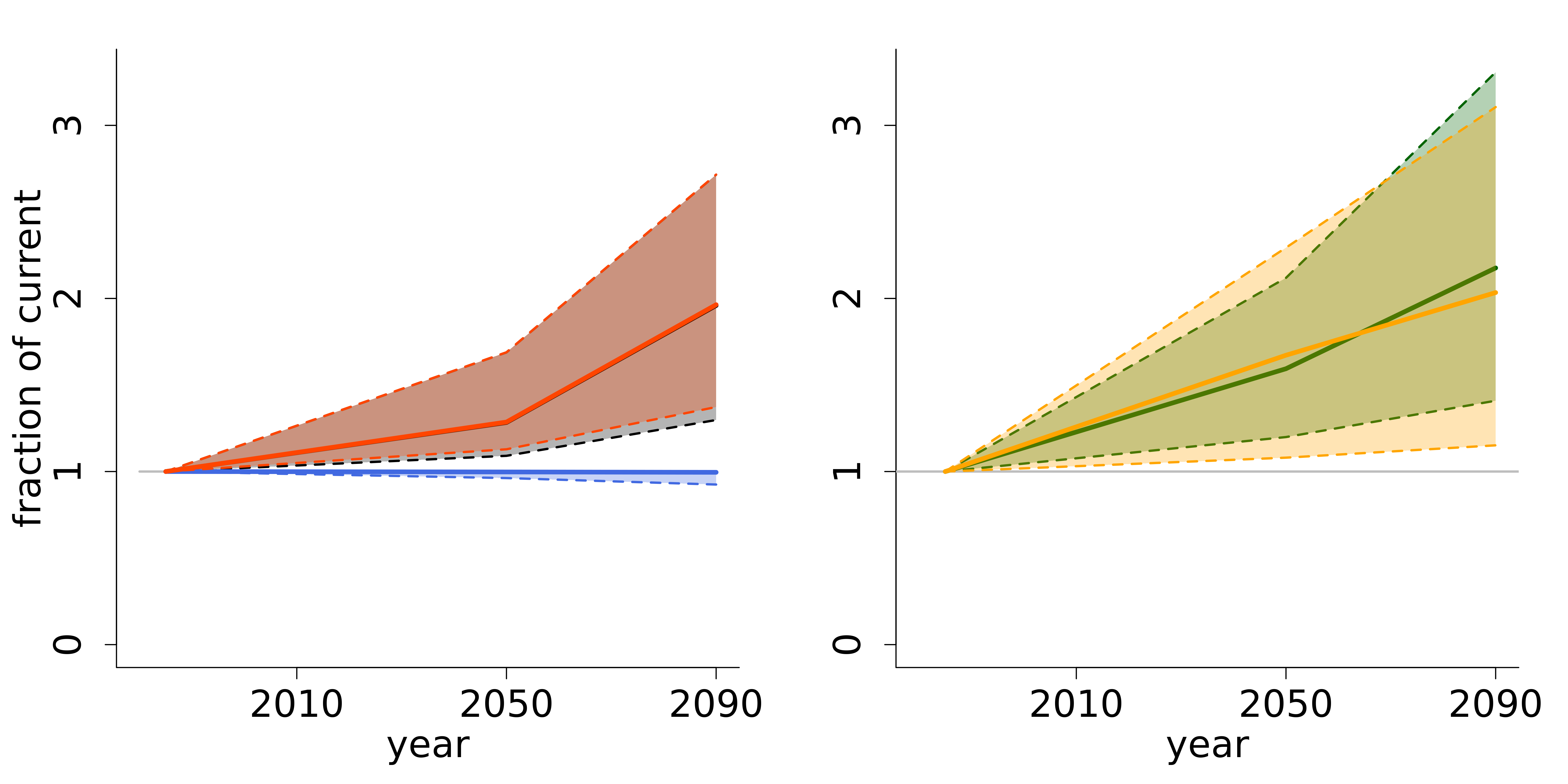

Supplement: S2 Appendix — (ZIP) [file pntd.0014030.s006.zip › Sup. Mat. 6-1 A-L - Species Trends/Crotalus_tigris_CCTrends.png]

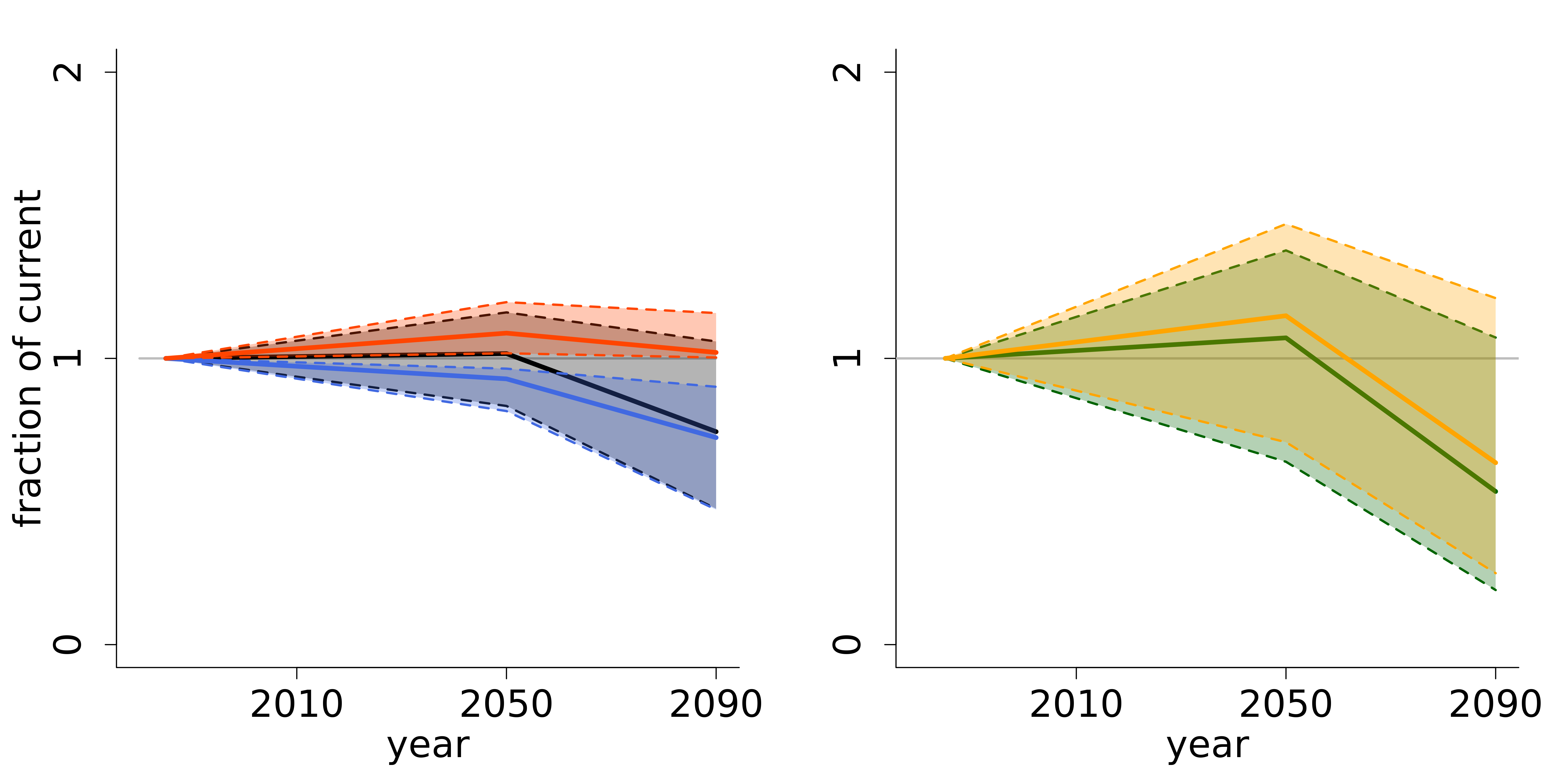

Supplement: S2 Appendix — (ZIP) [file pntd.0014030.s006.zip › Sup. Mat. 6-1 A-L - Species Trends/Crotalus_tlaloci_CCTrends.png]

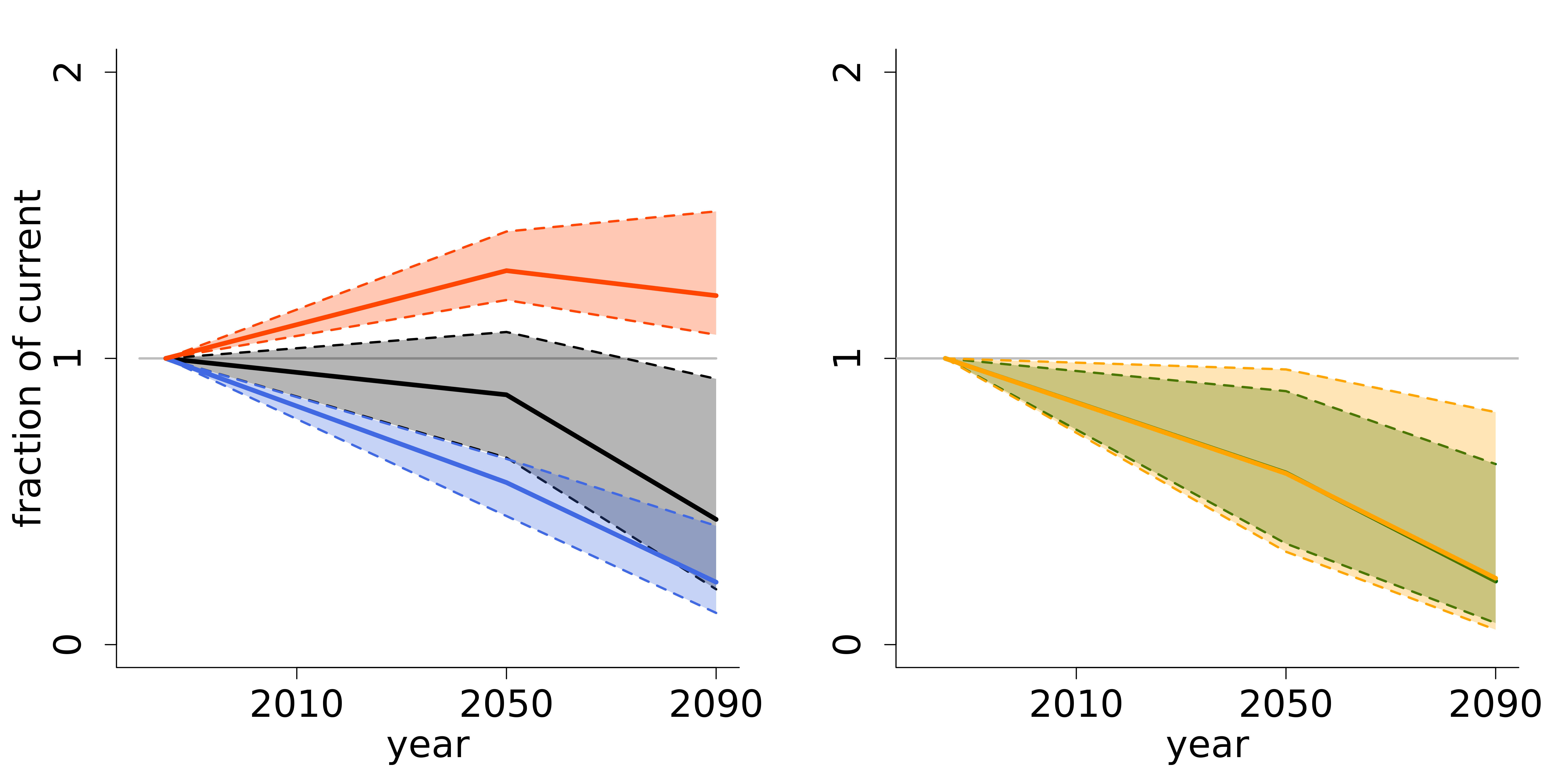

Supplement: S2 Appendix — (ZIP) [file pntd.0014030.s006.zip › Sup. Mat. 6-1 A-L - Species Trends/Crotalus_totonacus_CCTrends.png]

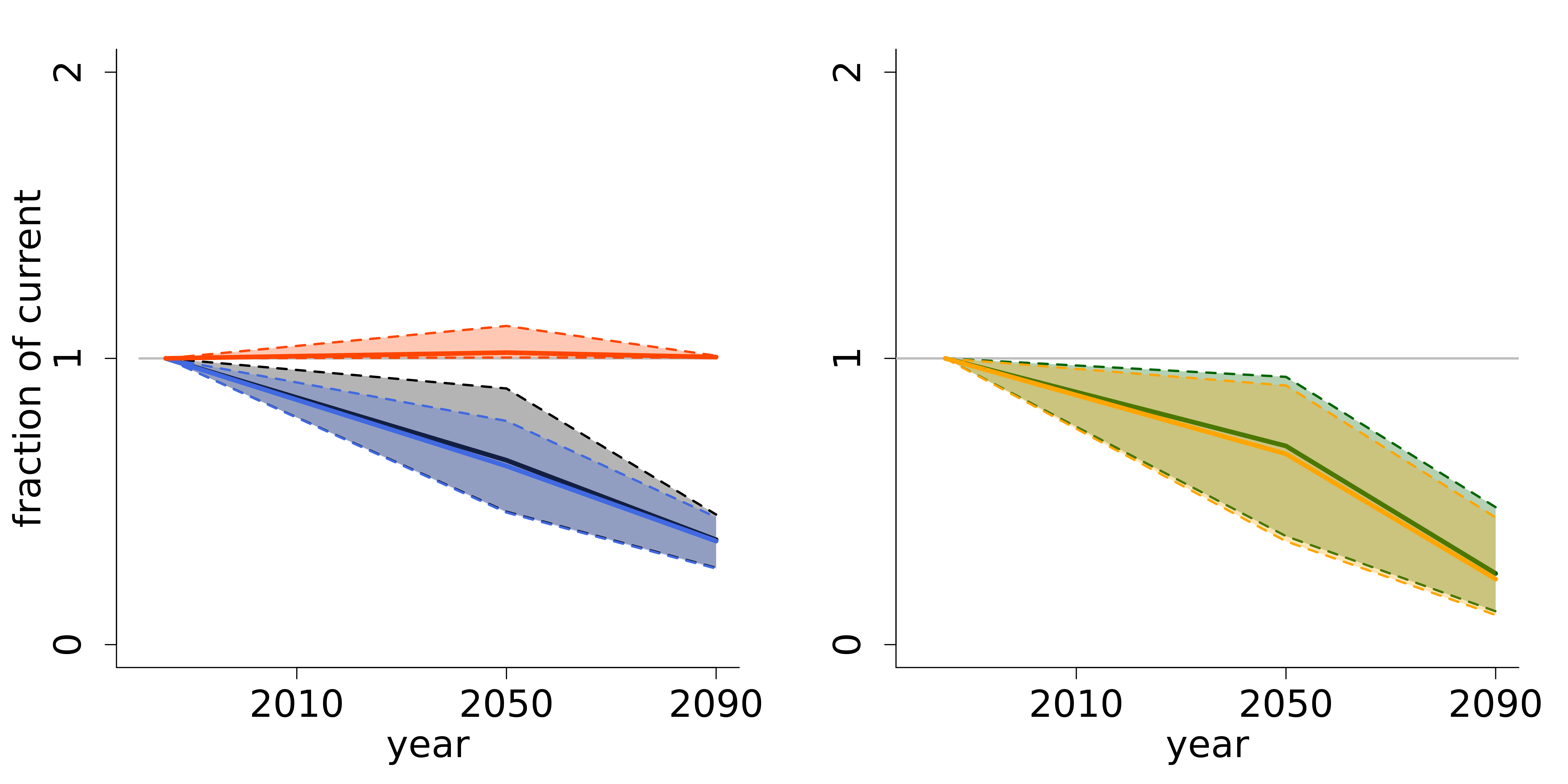

Supplement: S2 Appendix — (ZIP) [file pntd.0014030.s006.zip › Sup. Mat. 6-1 A-L - Species Trends/Crotalus_transversus_CCTrends.png]

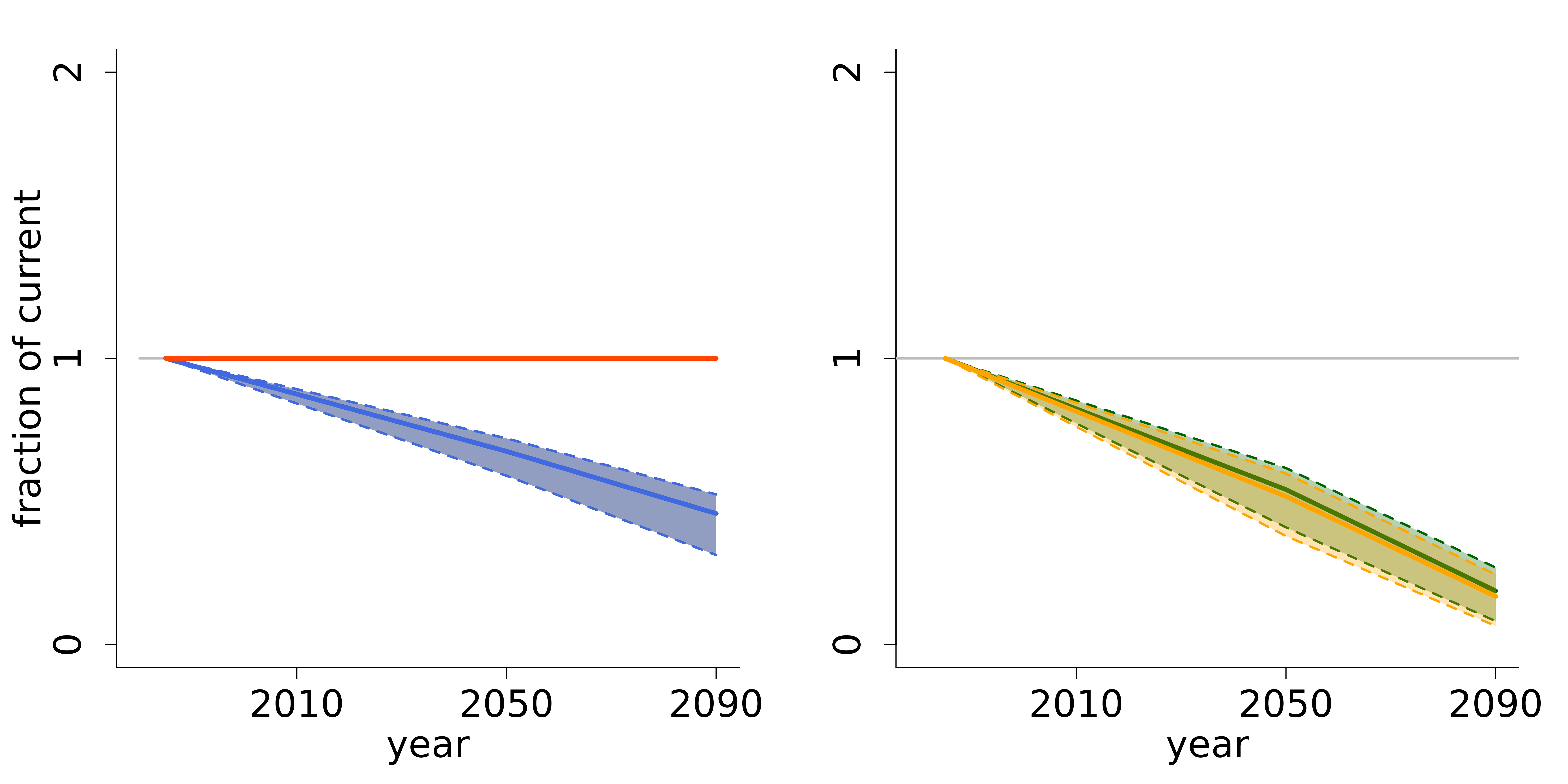

Supplement: S2 Appendix — (ZIP) [file pntd.0014030.s006.zip › Sup. Mat. 6-1 A-L - Species Trends/Crotalus_triseriatus_CCTrends.png]

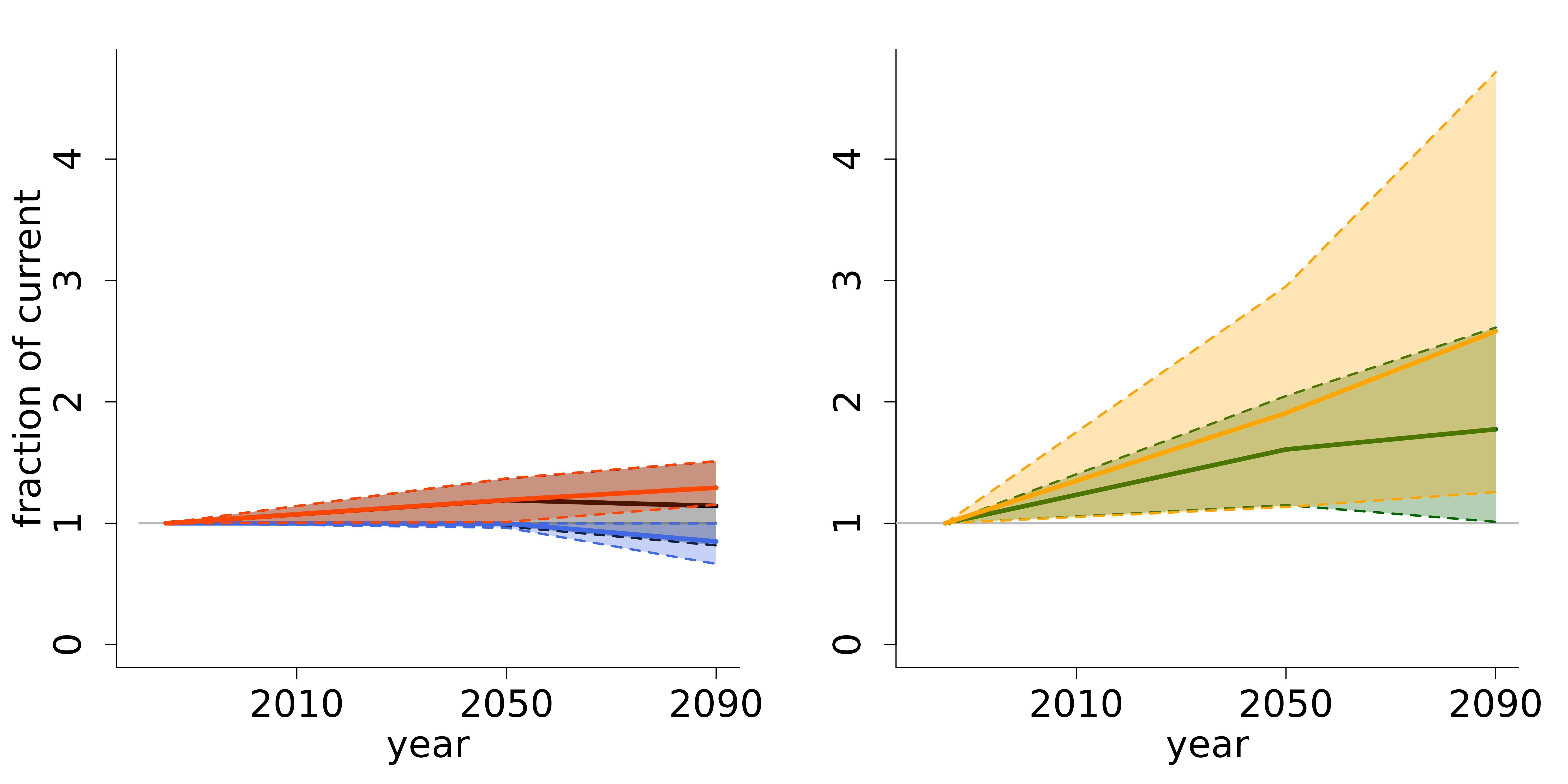

Supplement: S2 Appendix — (ZIP) [file pntd.0014030.s006.zip › Sup. Mat. 6-1 A-L - Species Trends/Crotalus_tzabcan_CCTrends.png]

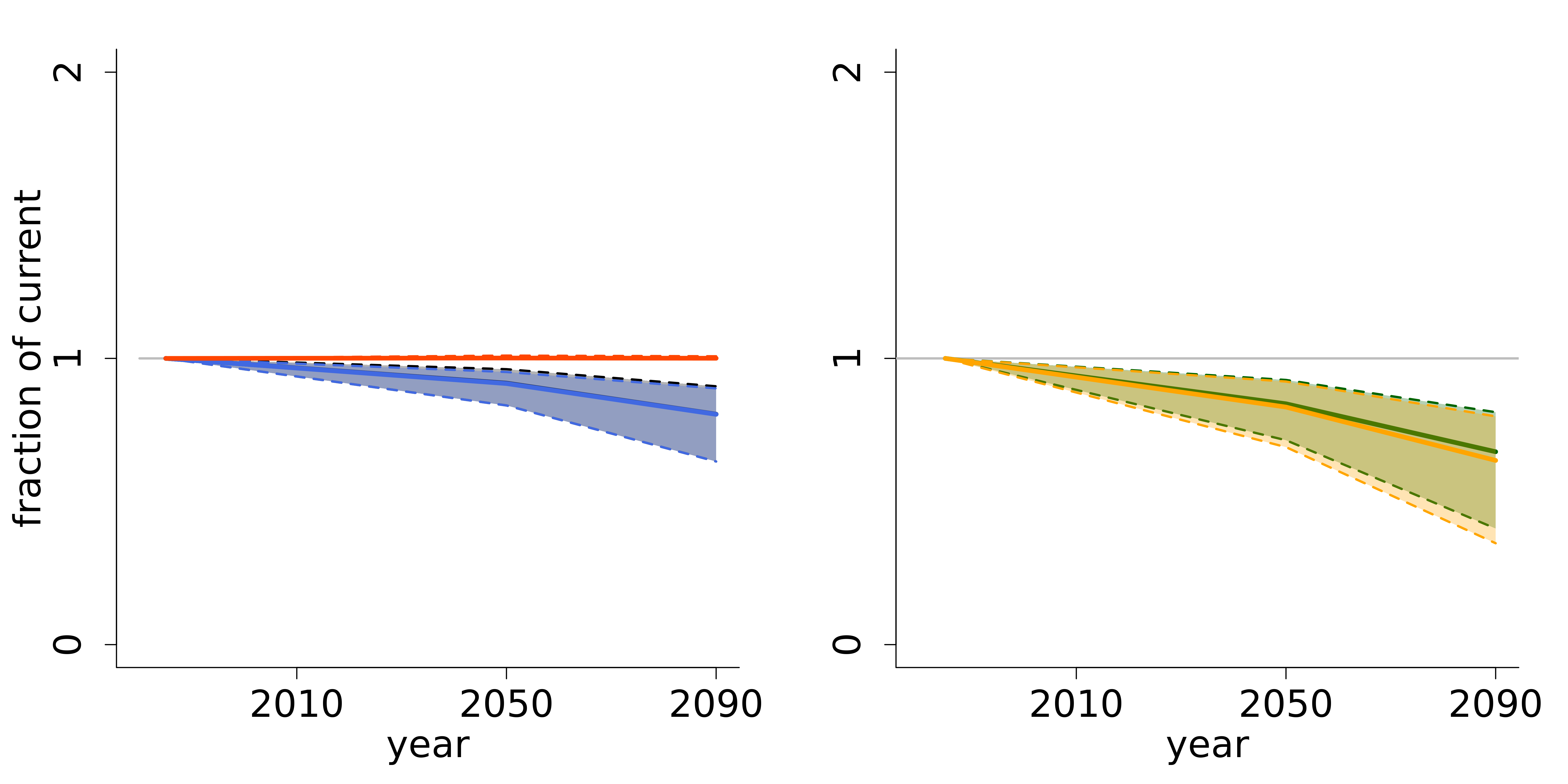

Supplement: S2 Appendix — (ZIP) [file pntd.0014030.s006.zip › Sup. Mat. 6-1 A-L - Species Trends/Crotalus_viridis_CCTrends.png]

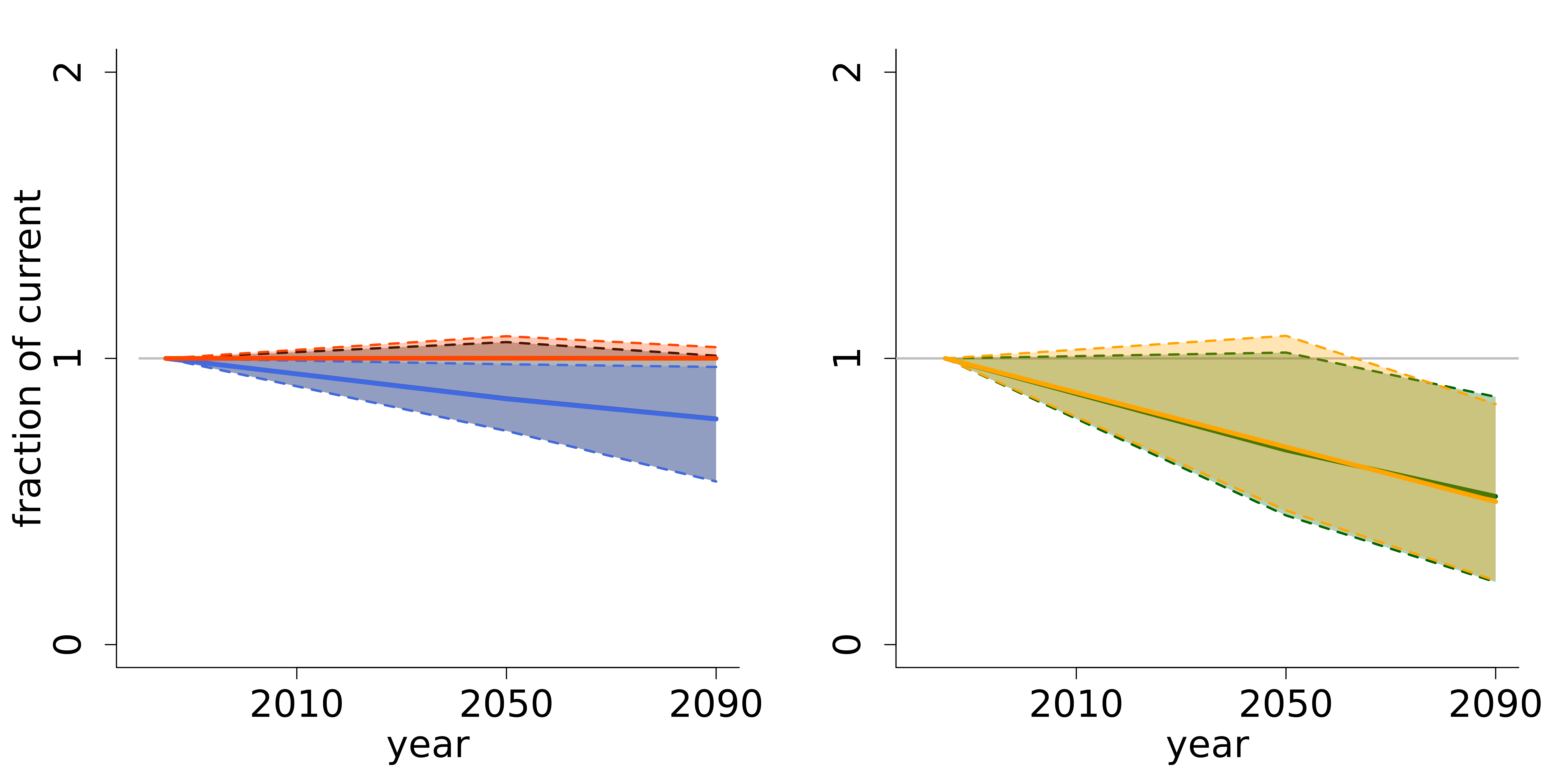

Supplement: S2 Appendix — (ZIP) [file pntd.0014030.s006.zip › Sup. Mat. 6-1 A-L - Species Trends/Crotalus_willardi_CCTrends.png]

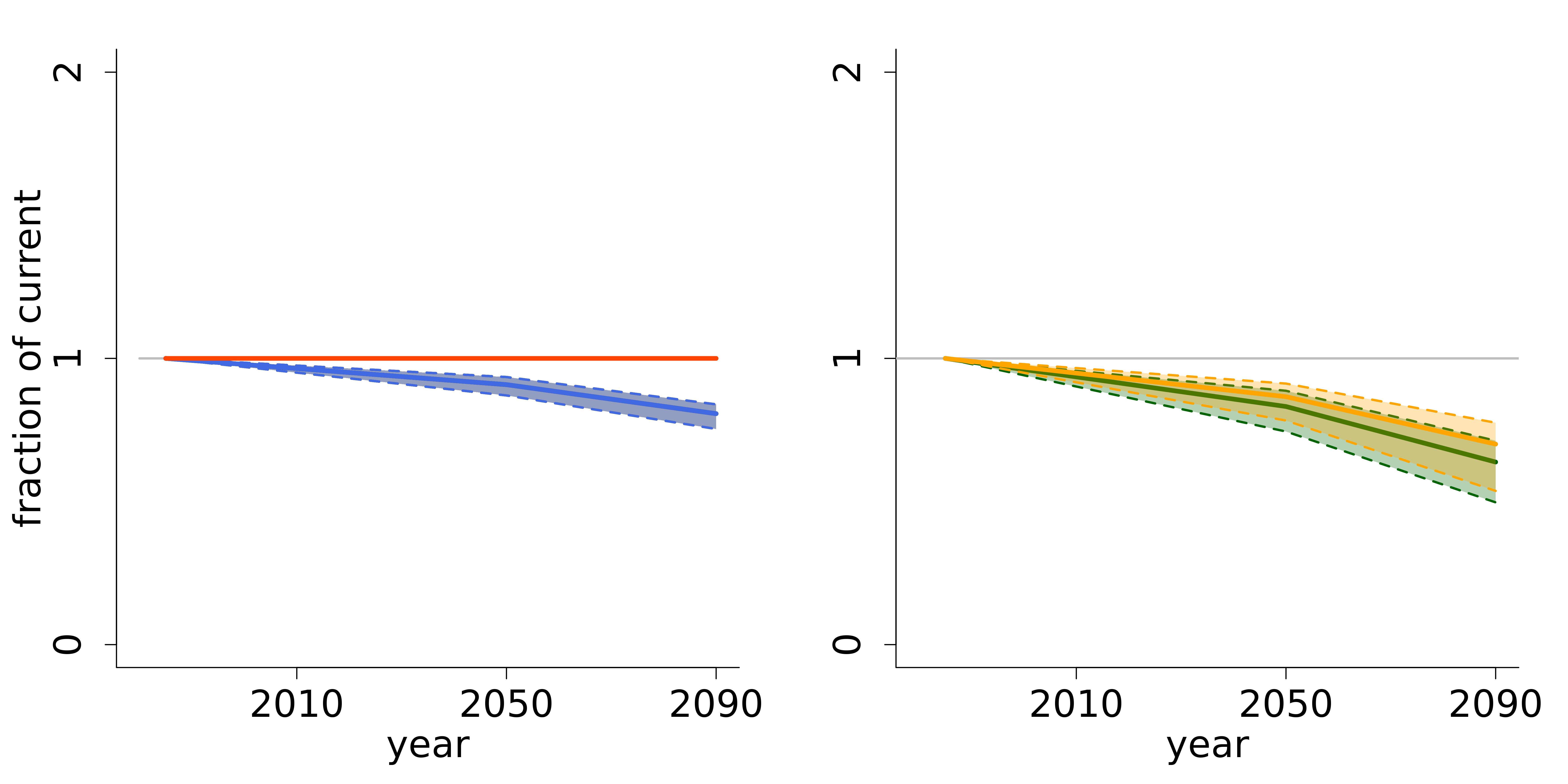

Supplement: S2 Appendix — (ZIP) [file pntd.0014030.s006.zip › Sup. Mat. 6-1 A-L - Species Trends/Daboia_mauritanica_CCTrends.png]

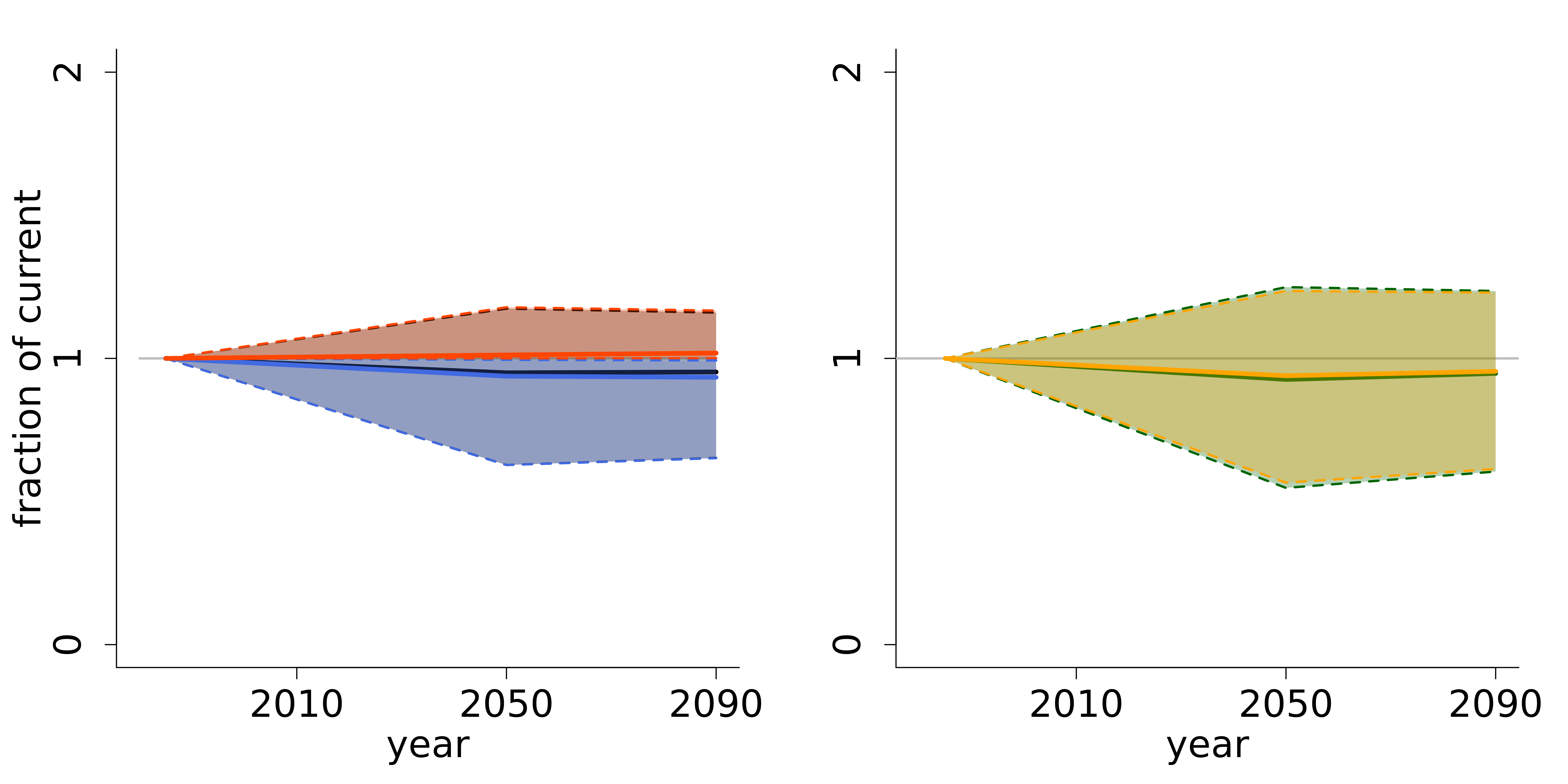

Supplement: S2 Appendix — (ZIP) [file pntd.0014030.s006.zip › Sup. Mat. 6-1 A-L - Species Trends/Daboia_palaestinae_CCTrends.png]

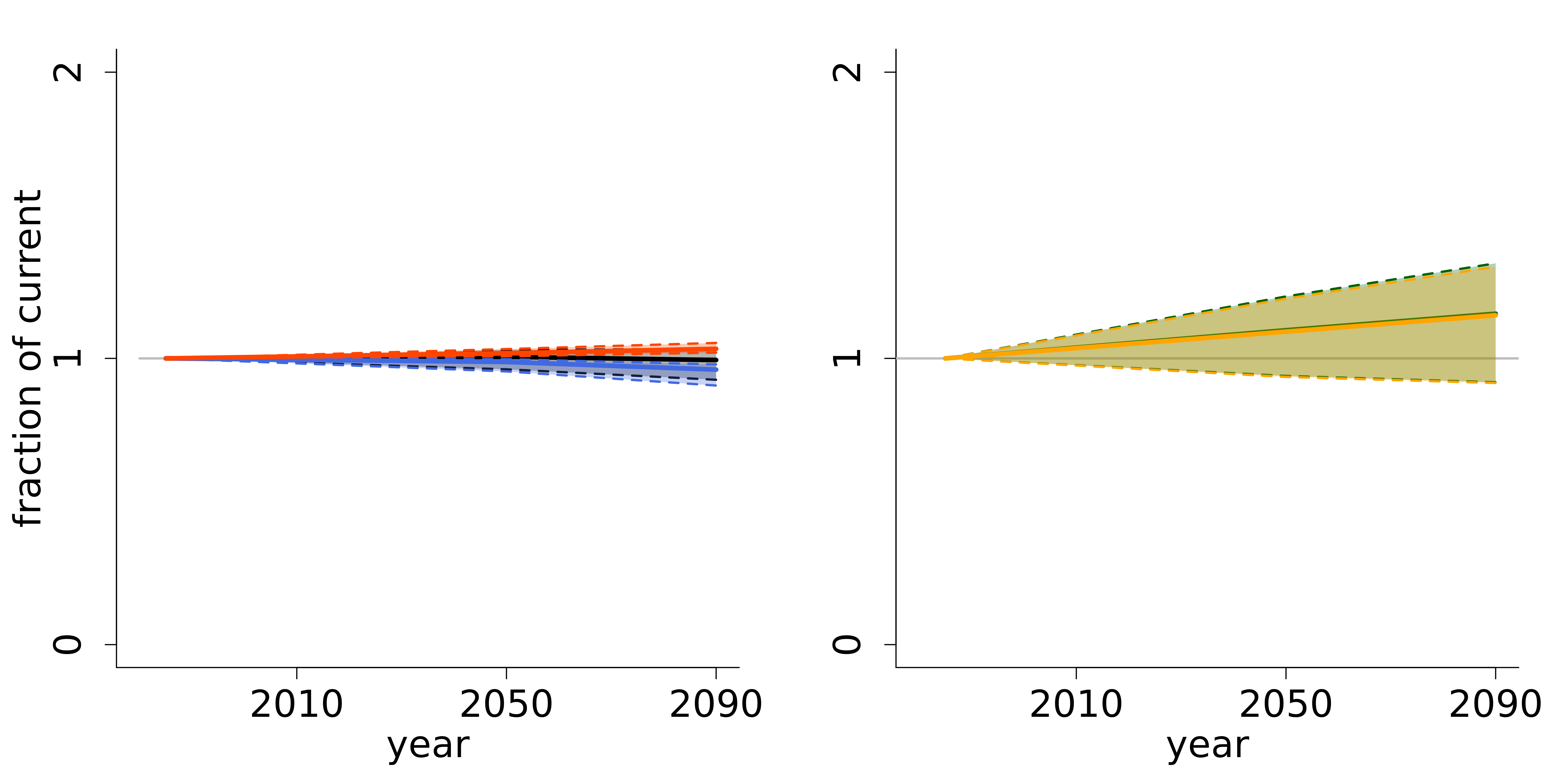

Supplement: S2 Appendix — (ZIP) [file pntd.0014030.s006.zip › Sup. Mat. 6-1 A-L - Species Trends/Daboia_russelii_CCTrends.png]

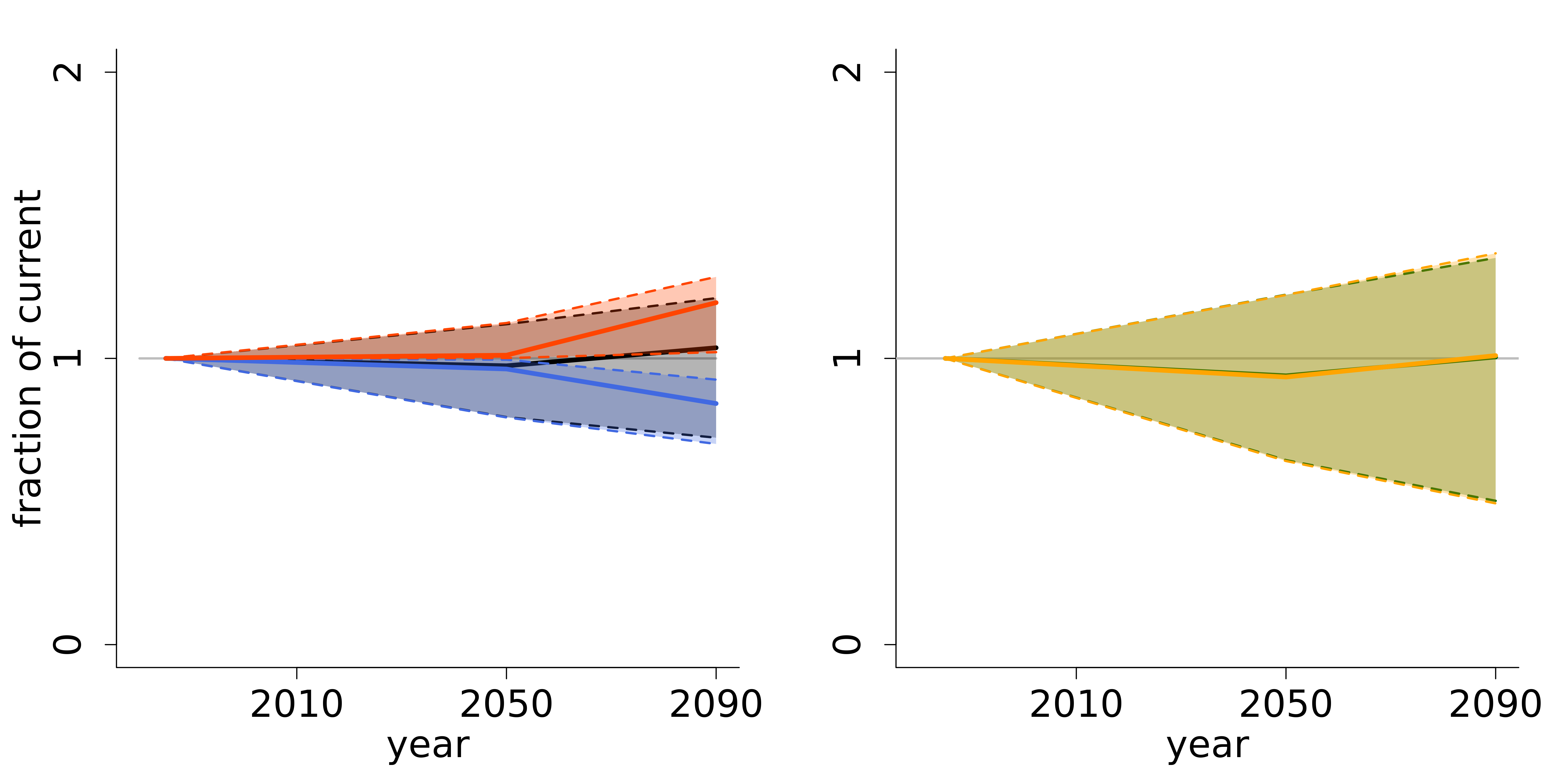

Supplement: S2 Appendix — (ZIP) [file pntd.0014030.s006.zip › Sup. Mat. 6-1 A-L - Species Trends/Daboia_siamensis_CCTrends.png]

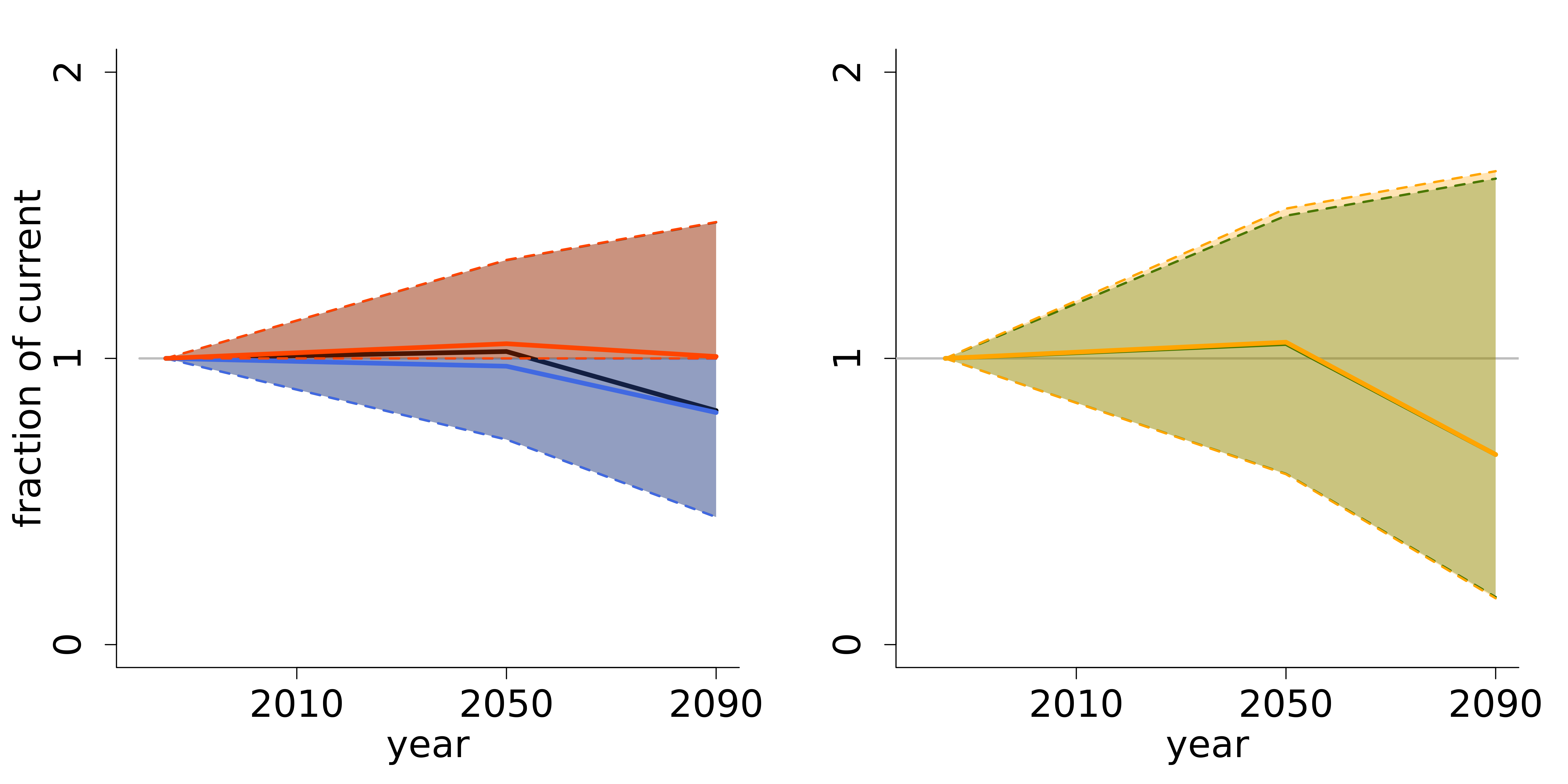

Supplement: S2 Appendix — (ZIP) [file pntd.0014030.s006.zip › Sup. Mat. 6-1 A-L - Species Trends/Deinagkistrodon_acutus_CCTrends.png]

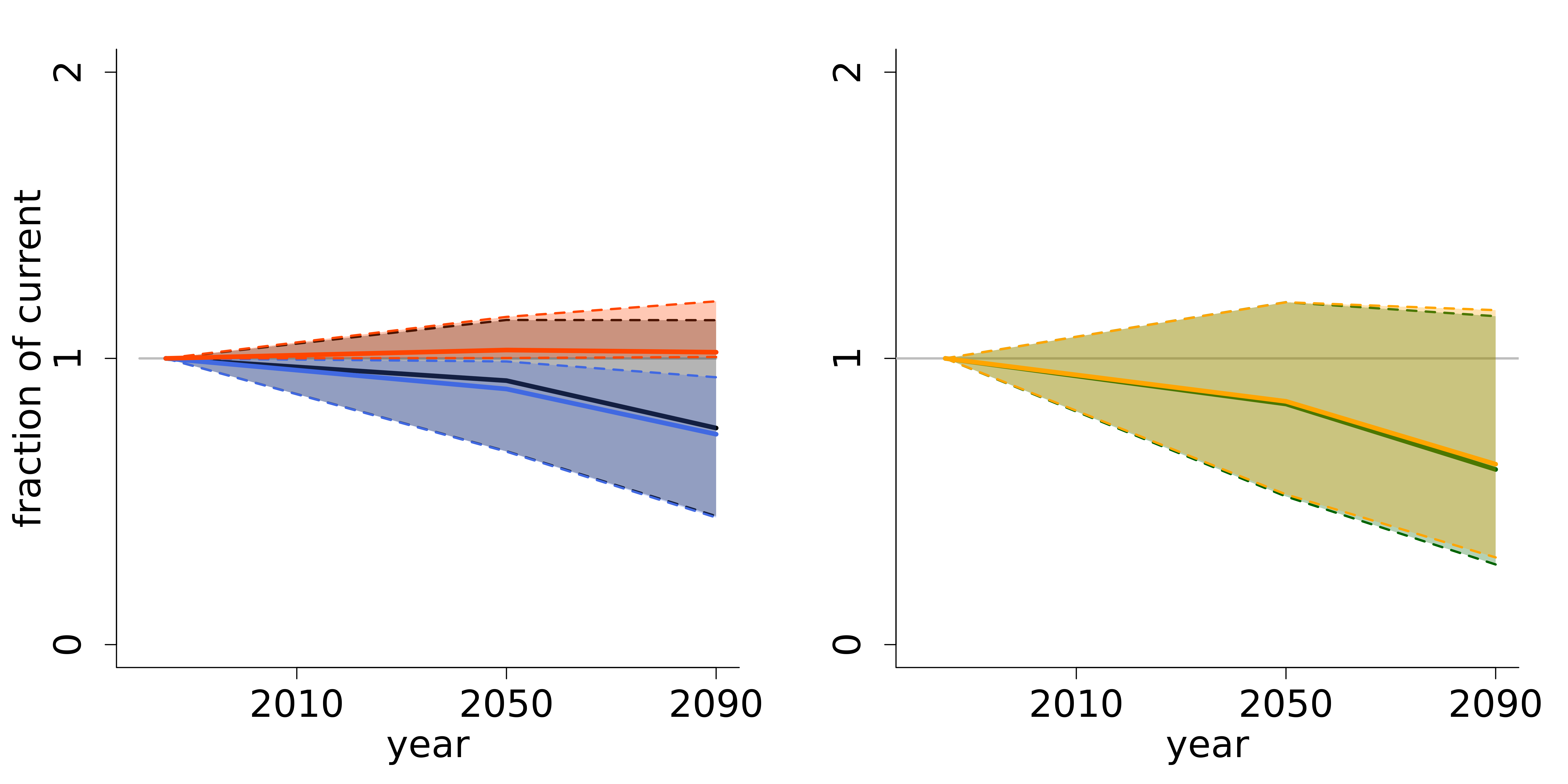

Supplement: S2 Appendix — (ZIP) [file pntd.0014030.s006.zip › Sup. Mat. 6-1 A-L - Species Trends/Dendroaspis_angusticeps_CCTrends.png]

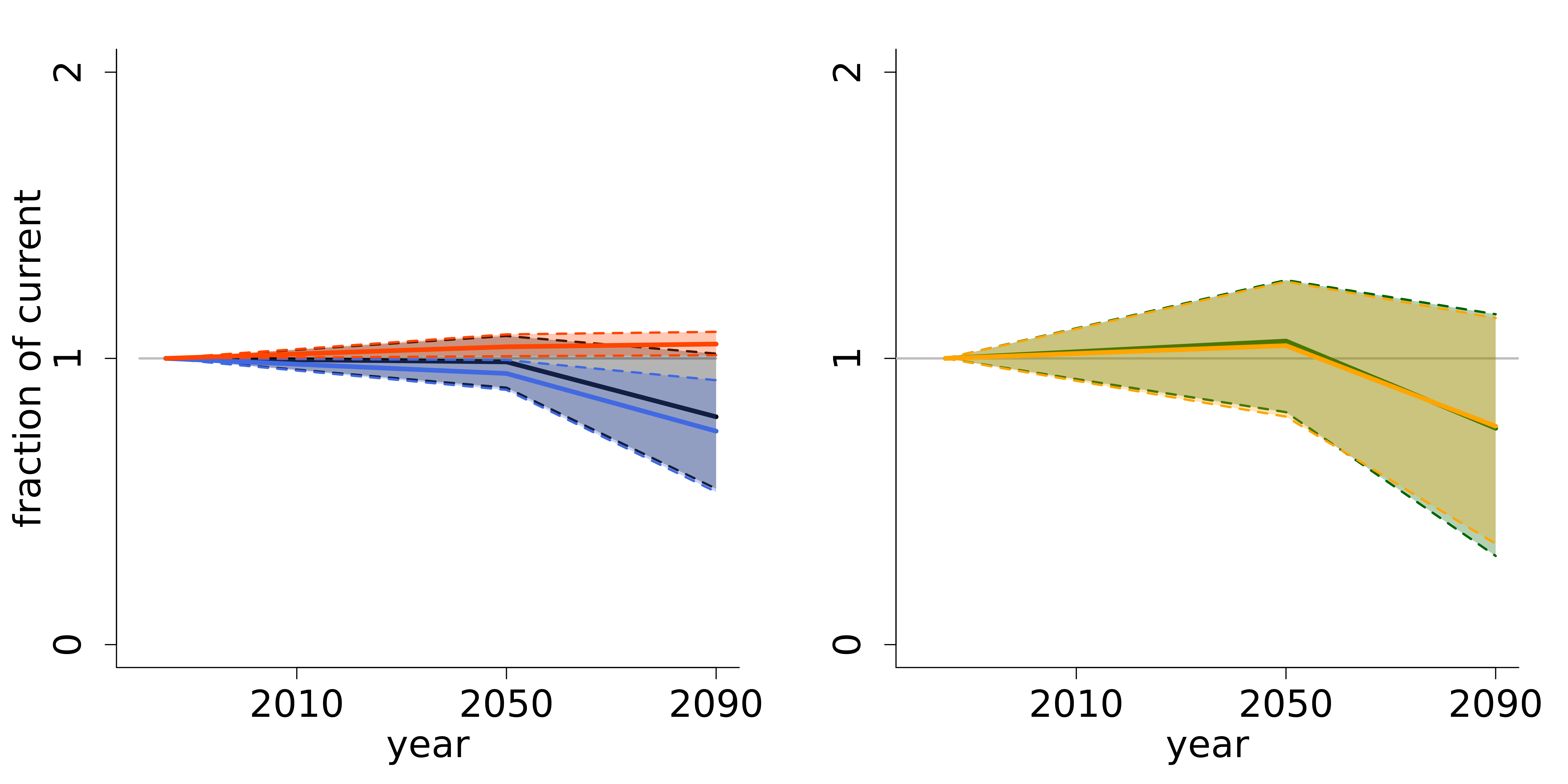

Supplement: S2 Appendix — (ZIP) [file pntd.0014030.s006.zip › Sup. Mat. 6-1 A-L - Species Trends/Dendroaspis_jamesoni_CCTrends.png]

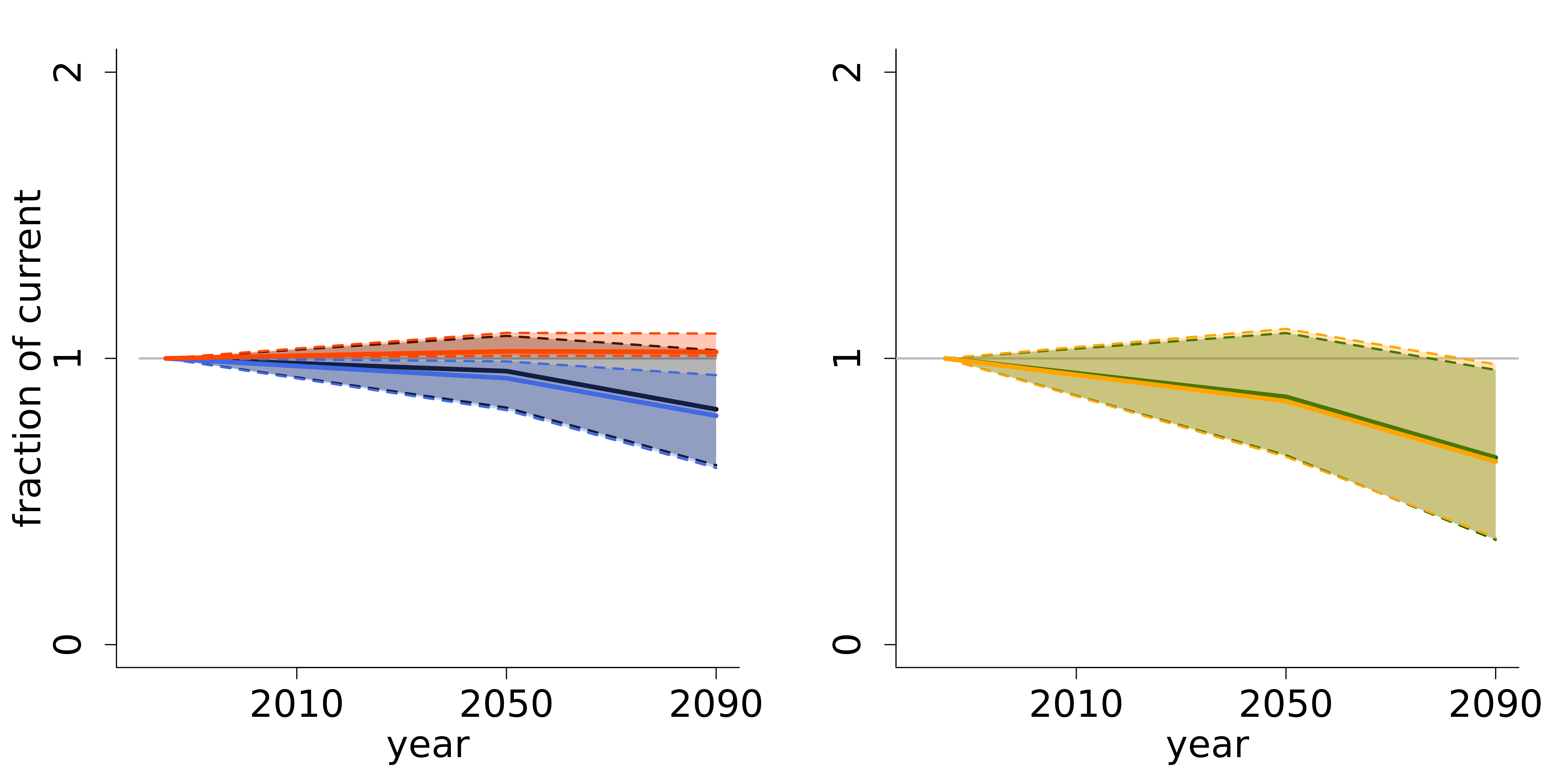

Supplement: S2 Appendix — (ZIP) [file pntd.0014030.s006.zip › Sup. Mat. 6-1 A-L - Species Trends/Dendroaspis_polylepis_CCTrends.png]

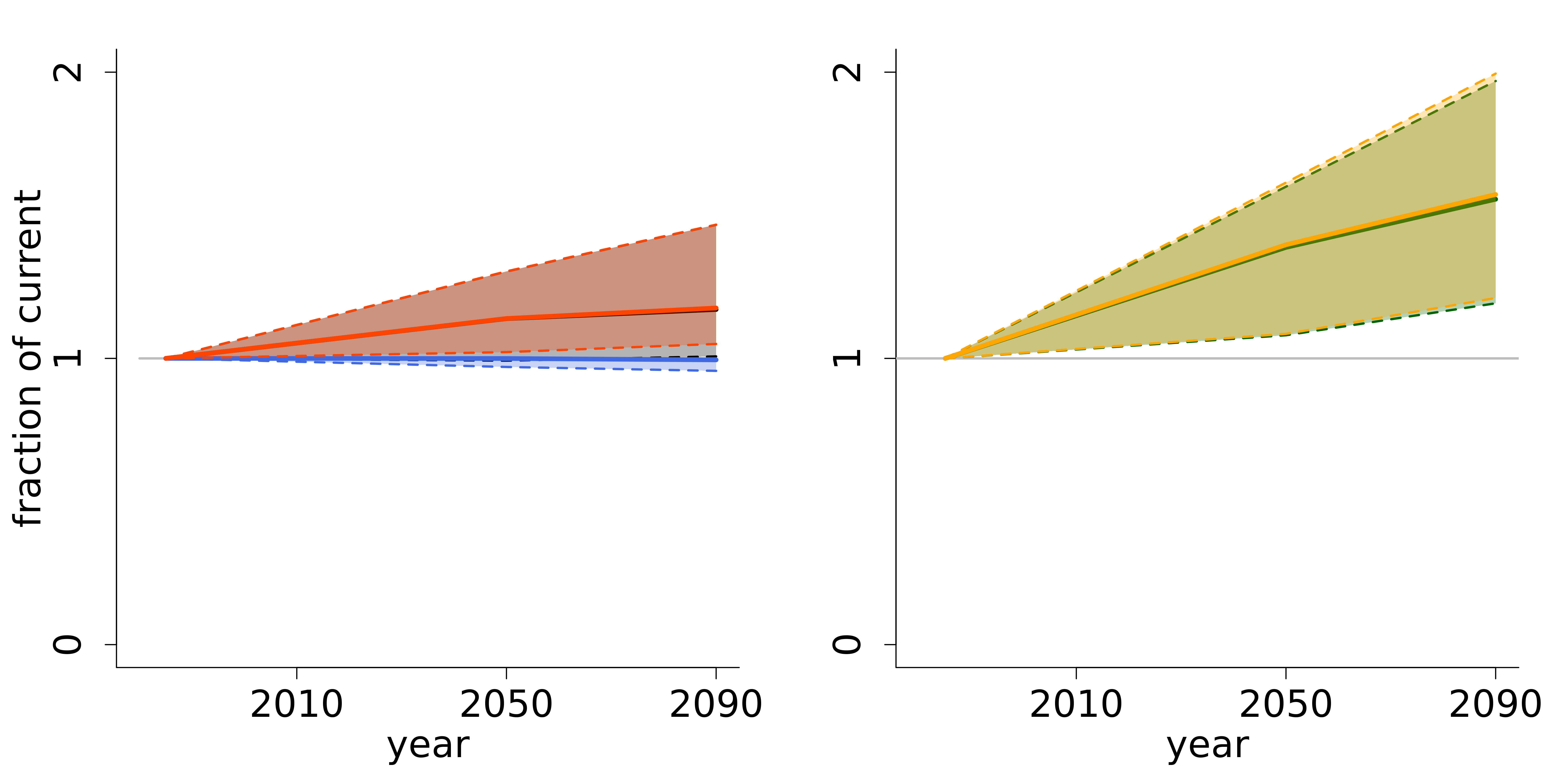

Supplement: S2 Appendix — (ZIP) [file pntd.0014030.s006.zip › Sup. Mat. 6-1 A-L - Species Trends/Dendroaspis_viridis_CCTrends.png]

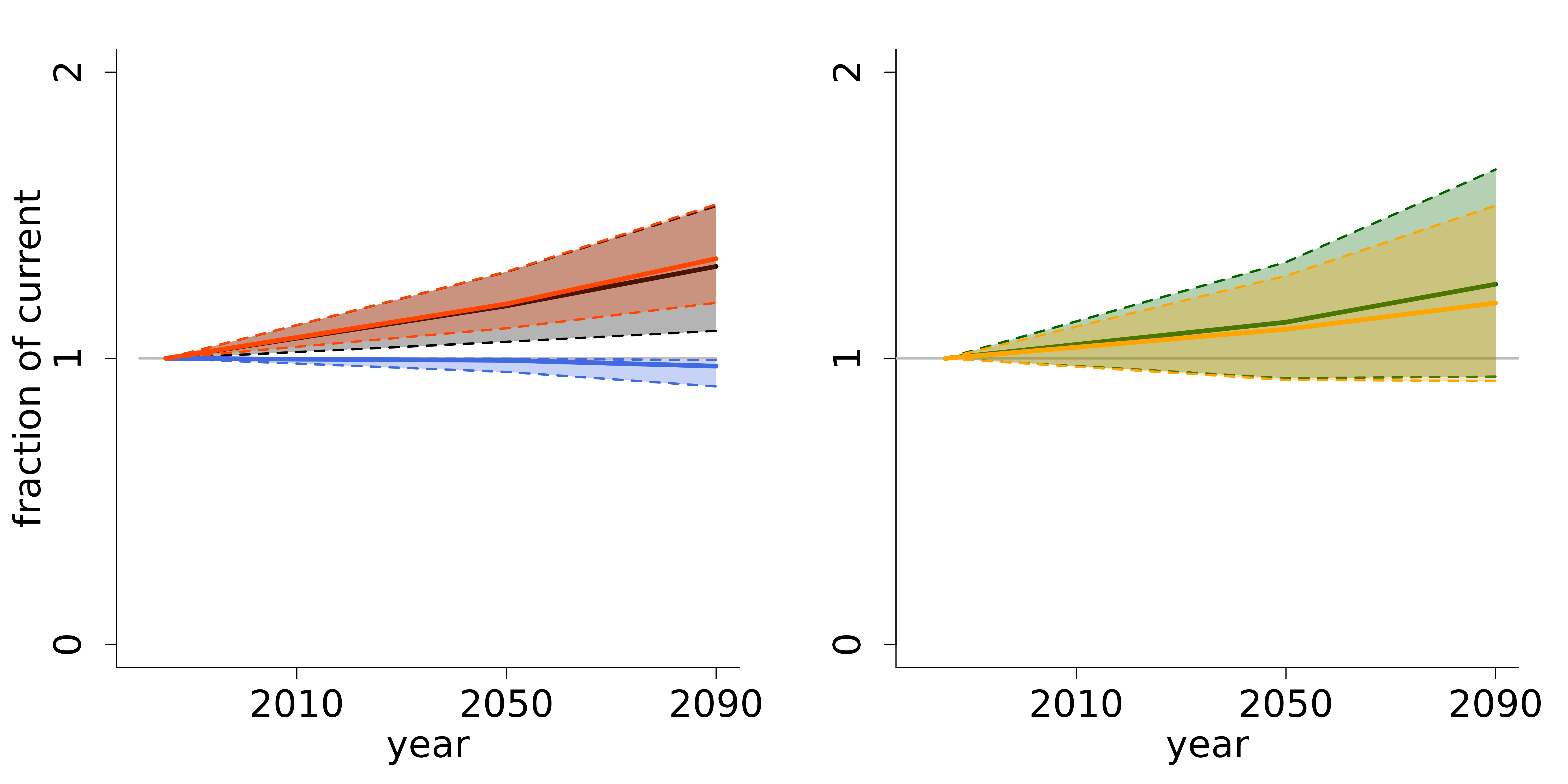

Supplement: S3 Appendix — (ZIP) [file pntd.0014030.s007.zip › Sup. Mat. 6-2 M-Z - Species Trends/Macrovipera_lebetina_CCTrends.png]

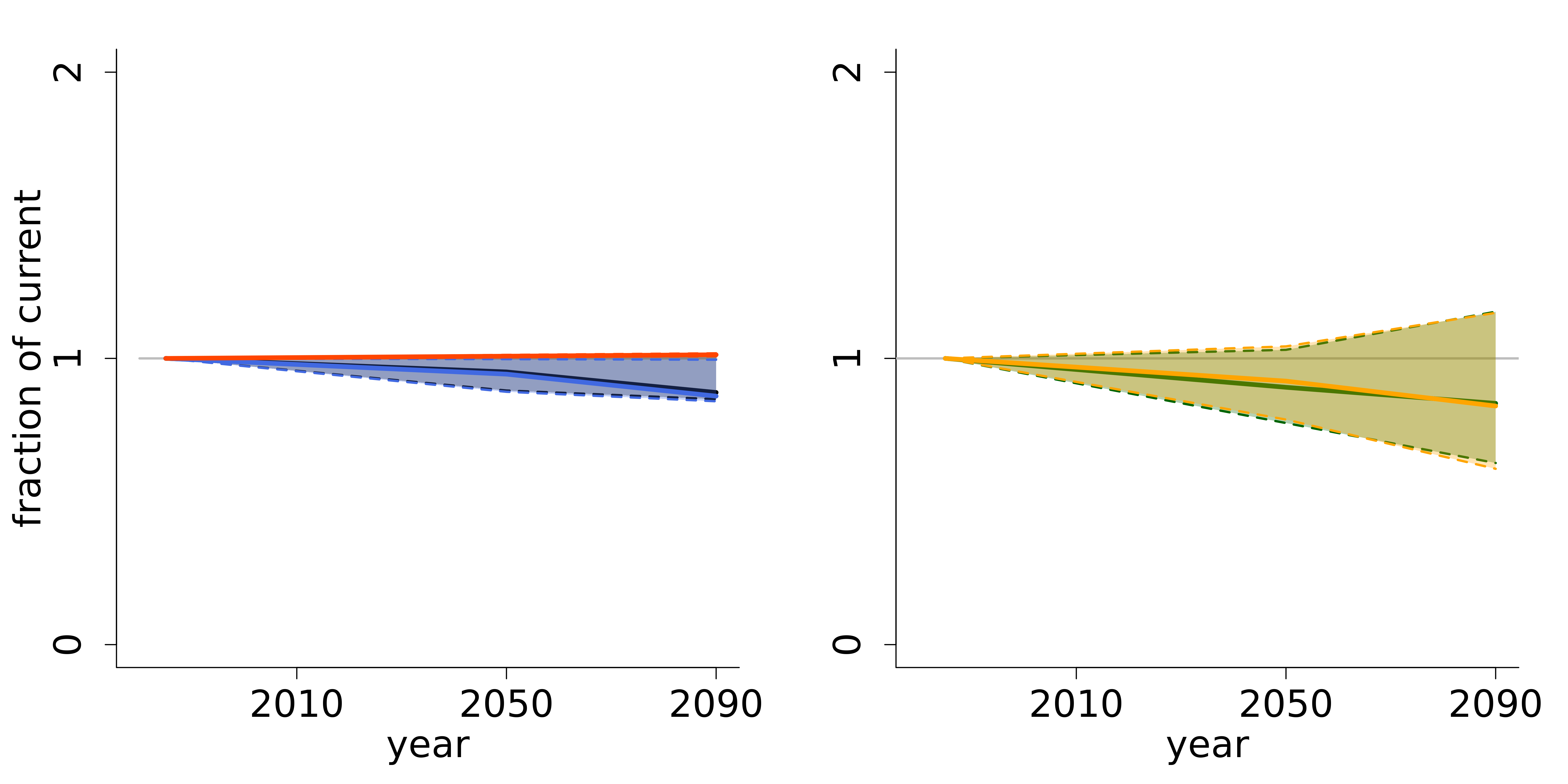

Supplement: S3 Appendix — (ZIP) [file pntd.0014030.s007.zip › Sup. Mat. 6-2 M-Z - Species Trends/Macrovipera_razii_CCTrends.png]

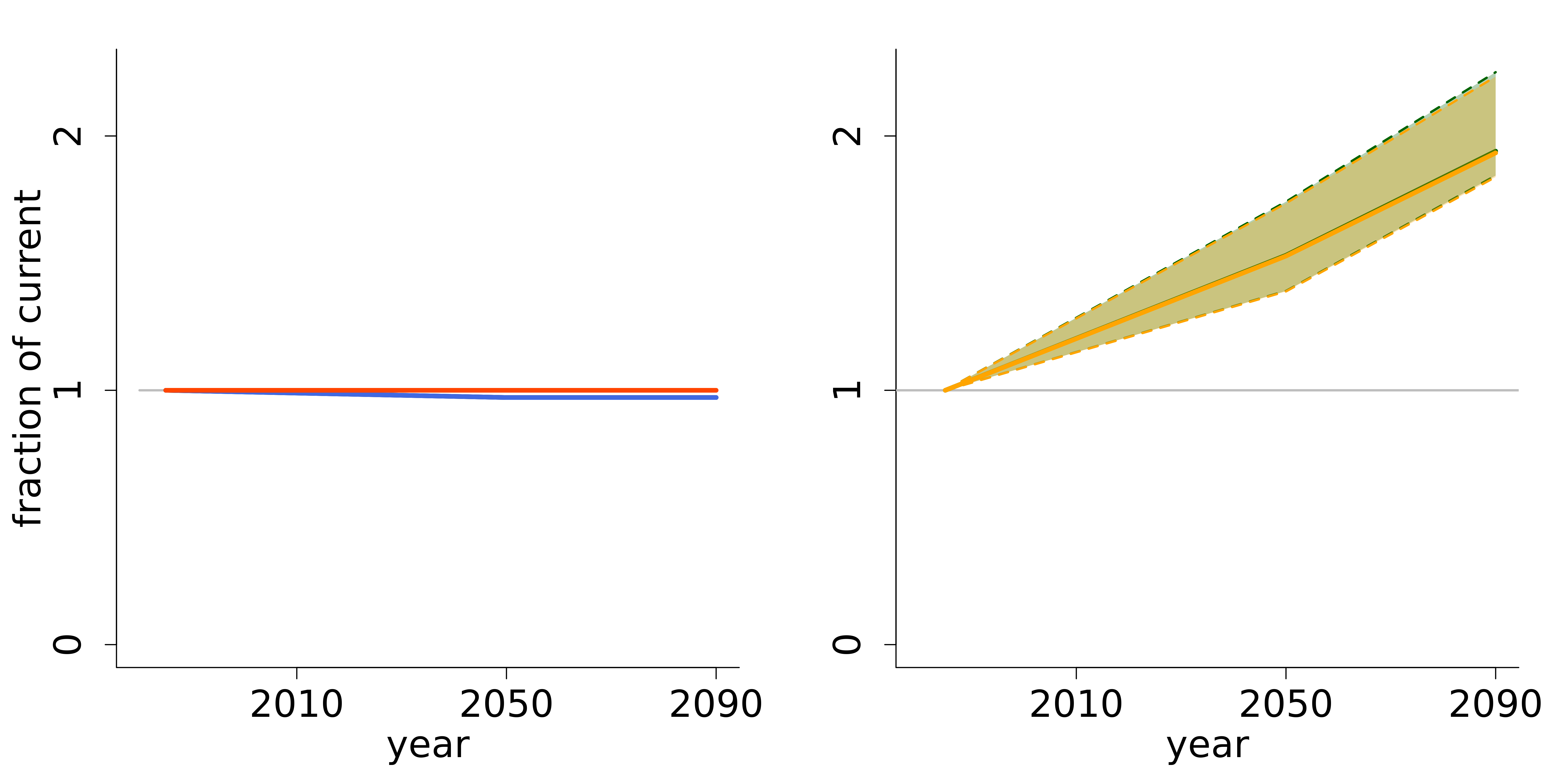

Supplement: S3 Appendix — (ZIP) [file pntd.0014030.s007.zip › Sup. Mat. 6-2 M-Z - Species Trends/Macrovipera_schweizeri_CCTrends.png]

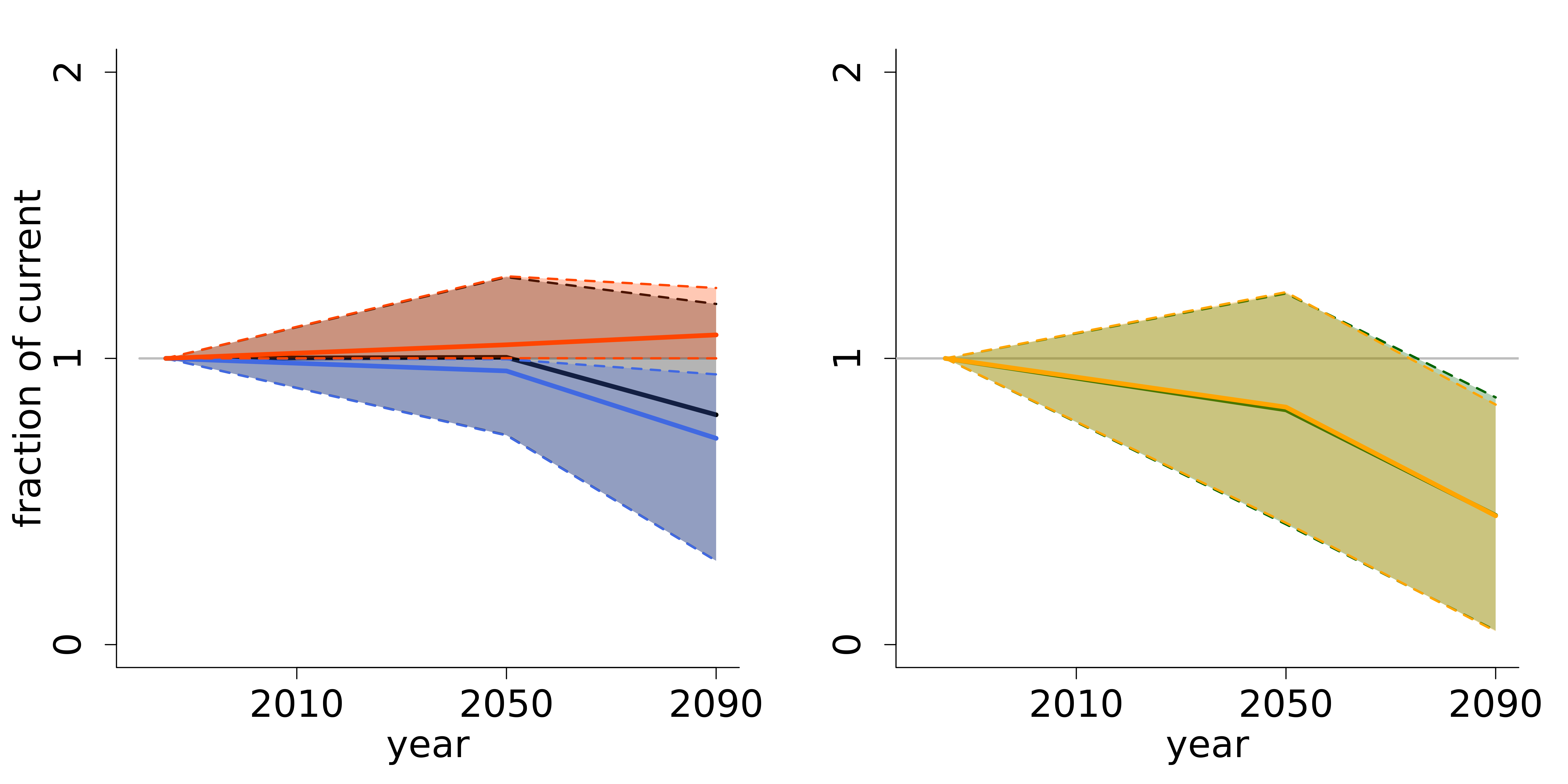

Supplement: S3 Appendix — (ZIP) [file pntd.0014030.s007.zip › Sup. Mat. 6-2 M-Z - Species Trends/Metlapilcoatlus_indomitus_CCTrends.png]
