# Supplementary figures and images for: Climate change induced complex shifts in snake distributions expose people to snakebite and threaten biodiversity (part 1 of 4)
Source: PLoS Negl Trop Dis. 2026 May 21;20(5):e0014030. doi: 10.1371/journal.pntd.0014030 (PMC13193456; doi:10.1371/journal.pntd.0014030)

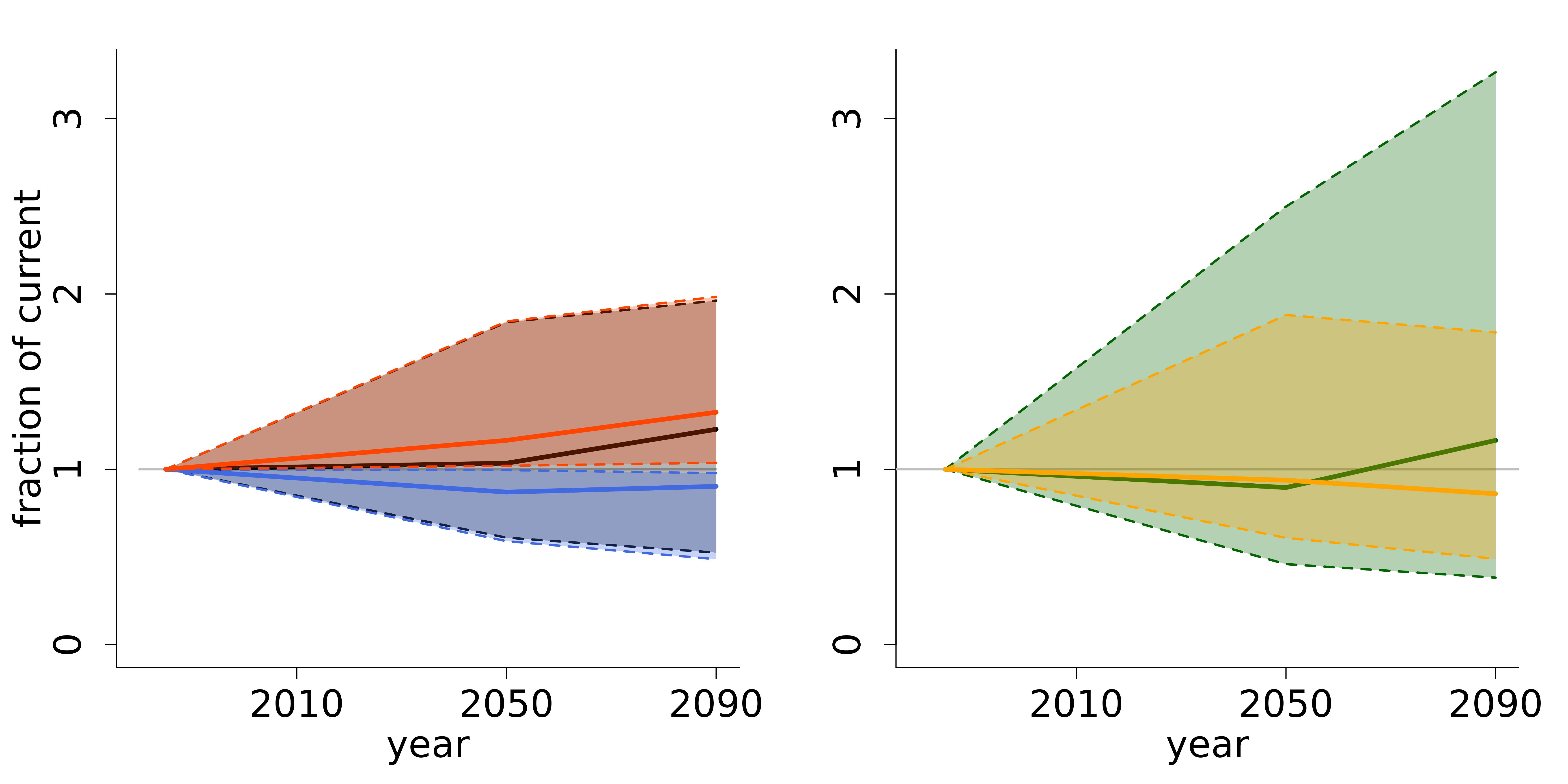

Supplement: S2 Appendix — (ZIP) [file pntd.0014030.s006.zip › Sup. Mat. 6-1 A-L - Species Trends/Acanthophis_antarcticus_CCTrends.png]

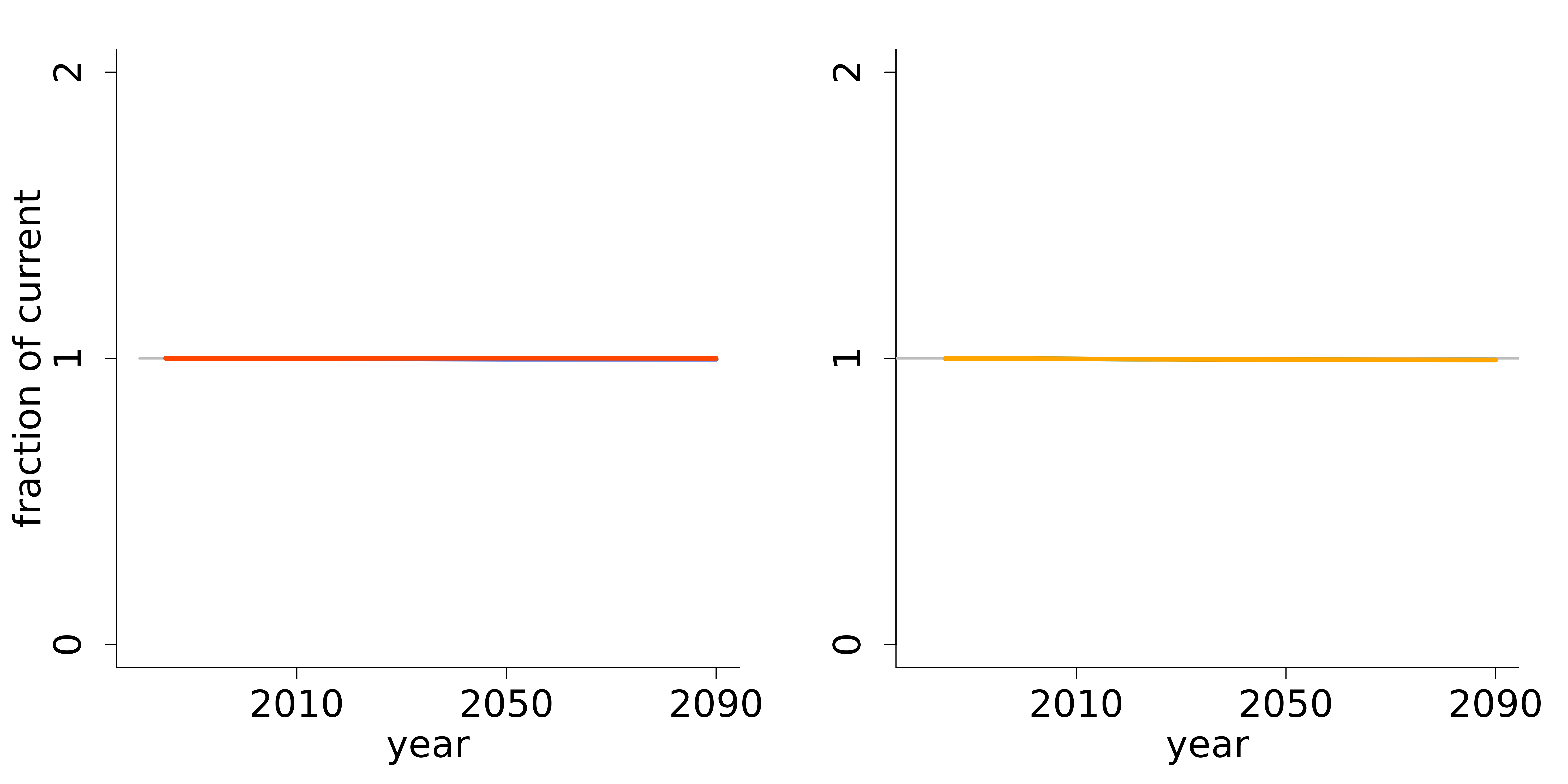

Supplement: S2 Appendix — (ZIP) [file pntd.0014030.s006.zip › Sup. Mat. 6-1 A-L - Species Trends/Acanthophis_cryptamydros_CCTrends.png]

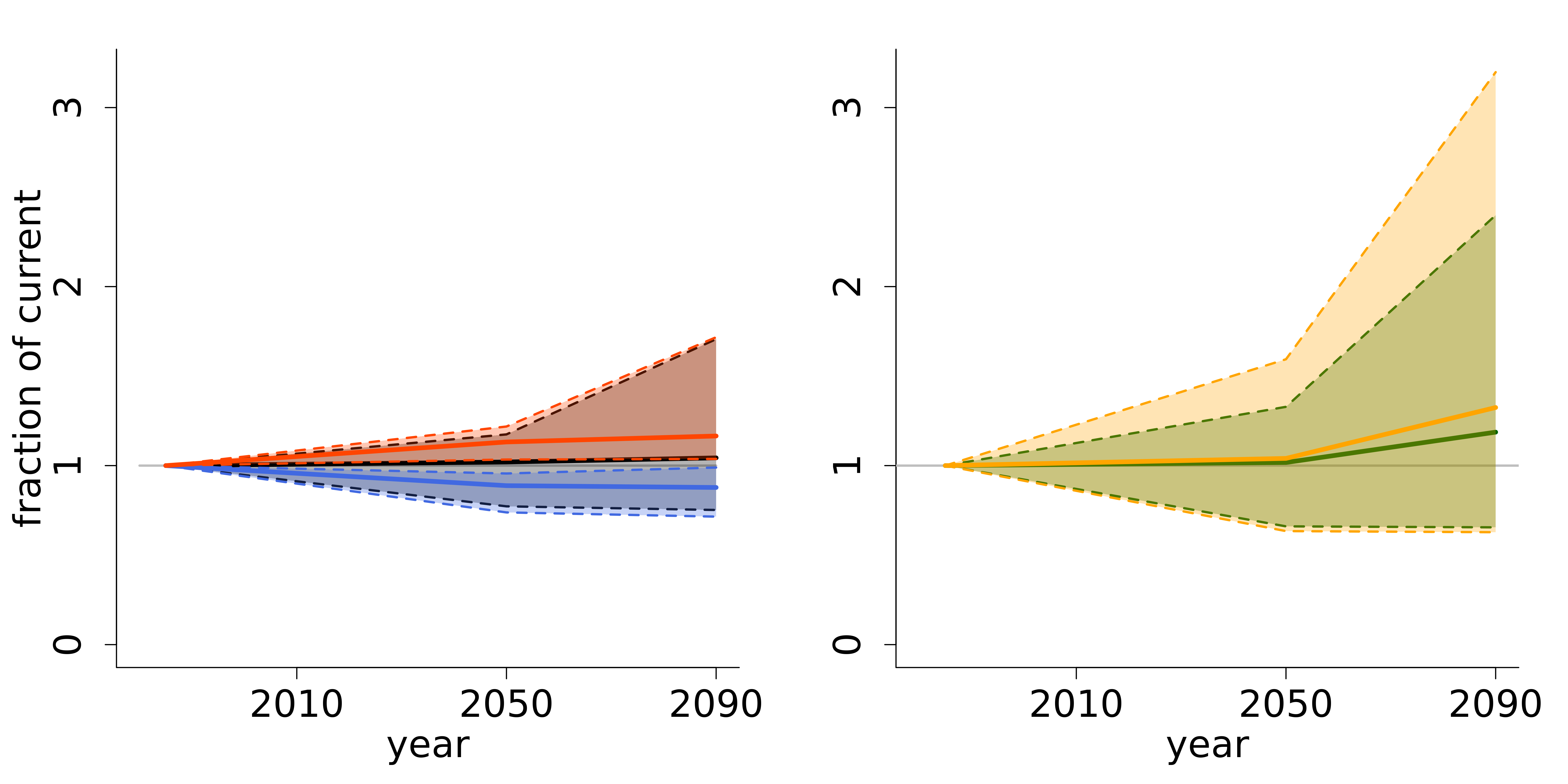

Supplement: S2 Appendix — (ZIP) [file pntd.0014030.s006.zip › Sup. Mat. 6-1 A-L - Species Trends/Acanthophis_hawkei_CCTrends.png]

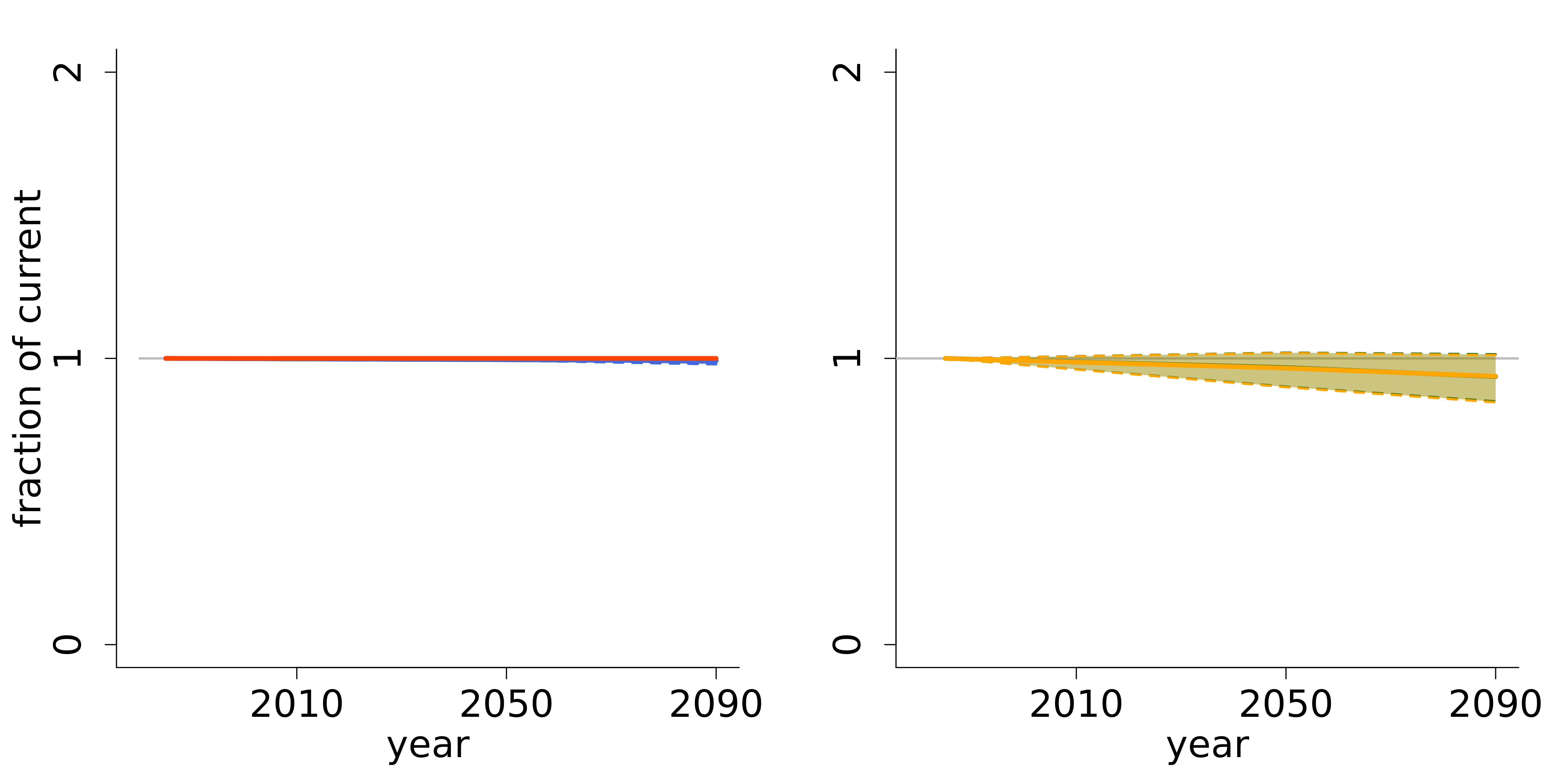

Supplement: S2 Appendix — (ZIP) [file pntd.0014030.s006.zip › Sup. Mat. 6-1 A-L - Species Trends/Acanthophis_laevis_CCTrends.png]

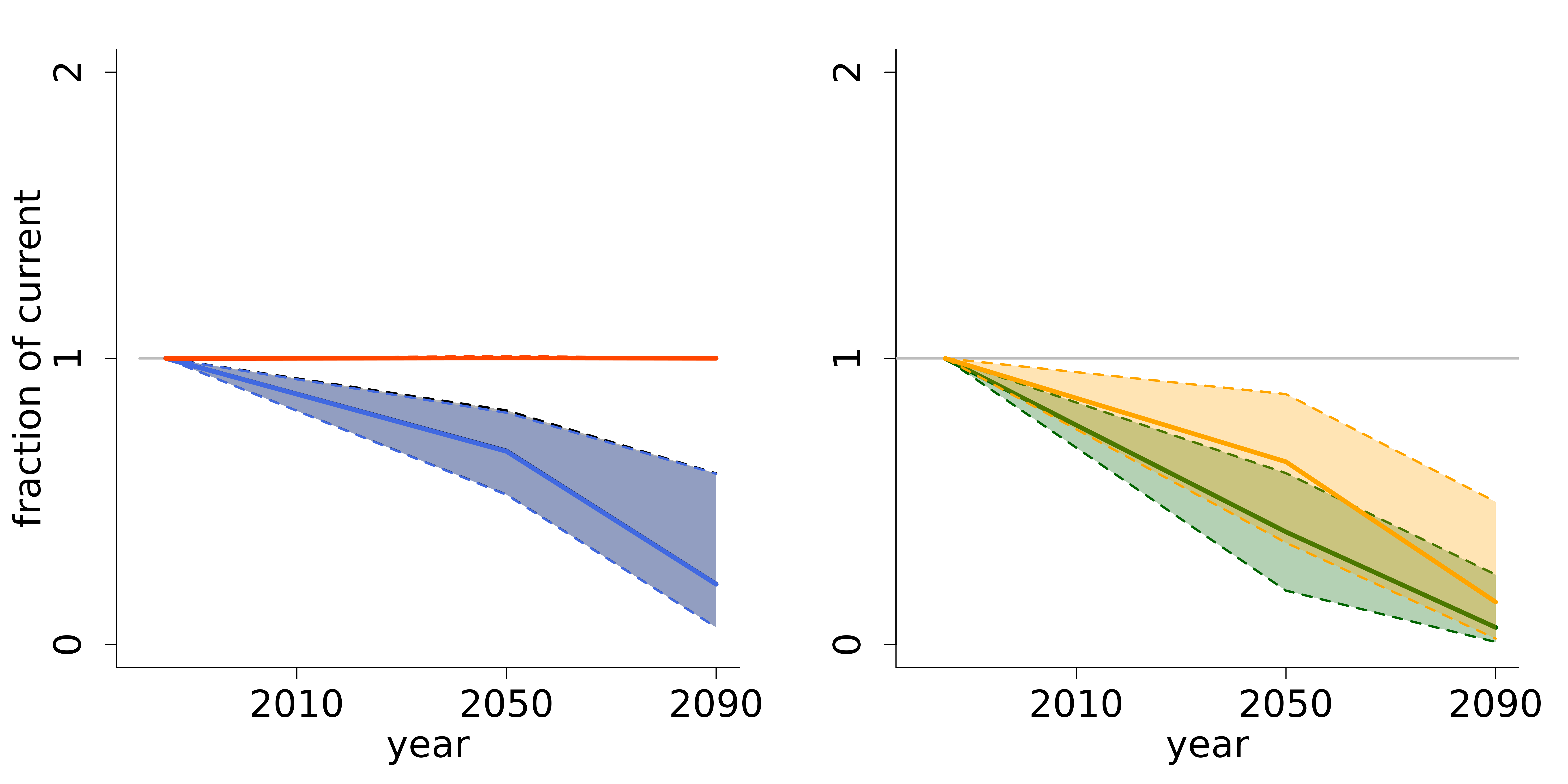

Supplement: S2 Appendix — (ZIP) [file pntd.0014030.s006.zip › Sup. Mat. 6-1 A-L - Species Trends/Acanthophis_praelongus_CCTrends.png]

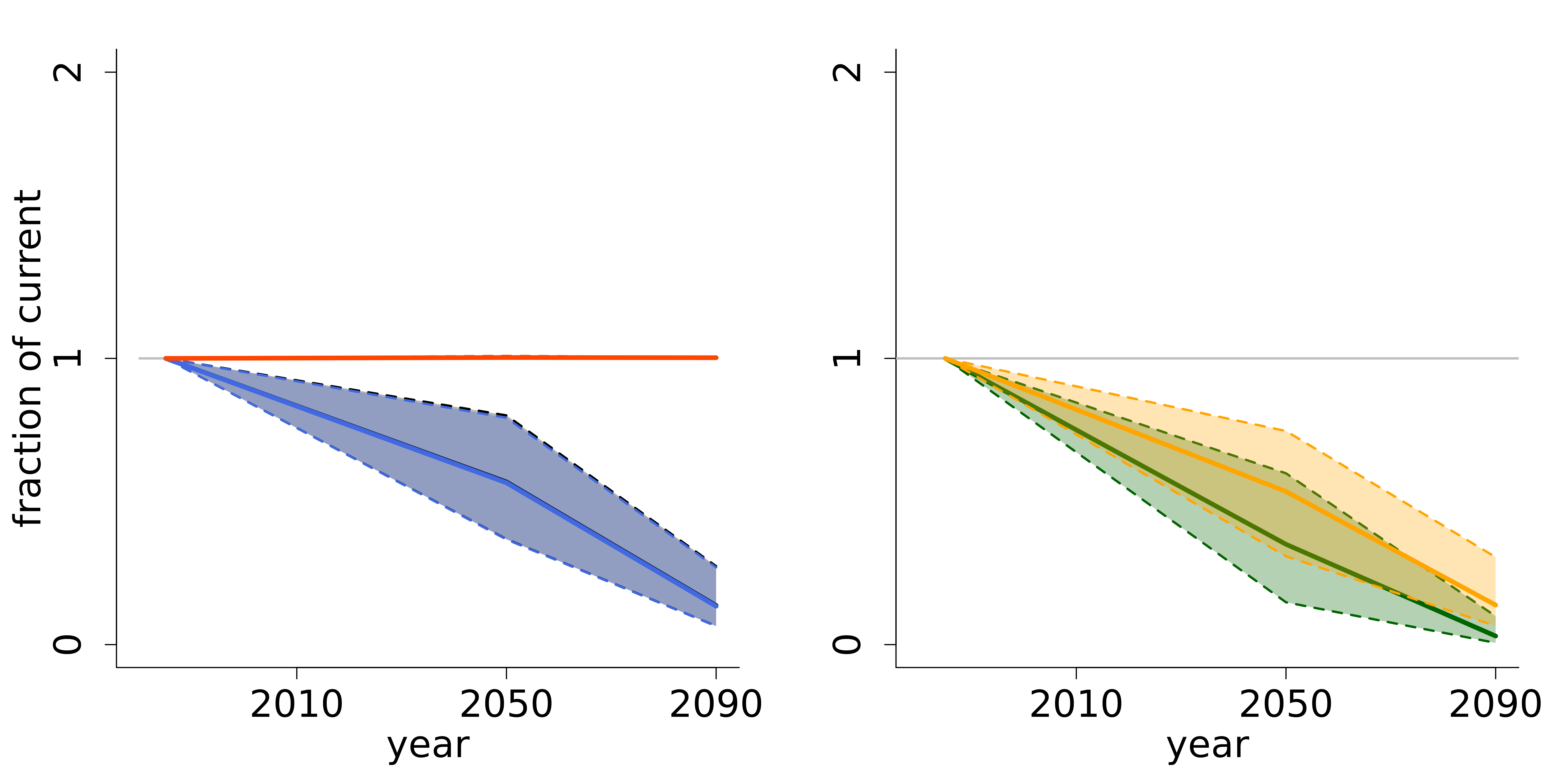

Supplement: S2 Appendix — (ZIP) [file pntd.0014030.s006.zip › Sup. Mat. 6-1 A-L - Species Trends/Acanthophis_pyrrhus_CCTrends.png]

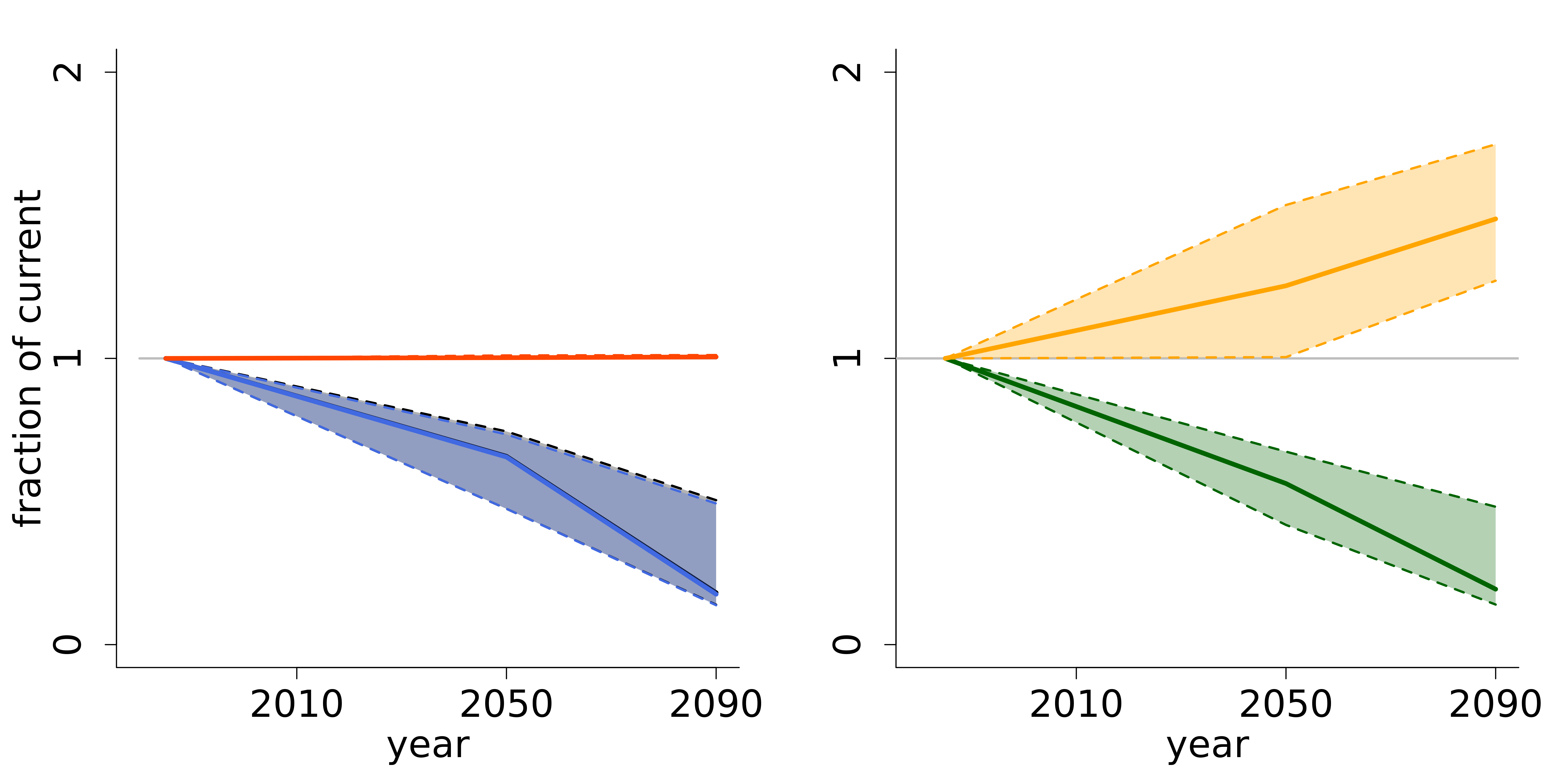

Supplement: S2 Appendix — (ZIP) [file pntd.0014030.s006.zip › Sup. Mat. 6-1 A-L - Species Trends/Acanthophis_rugosus_CCTrends.png]

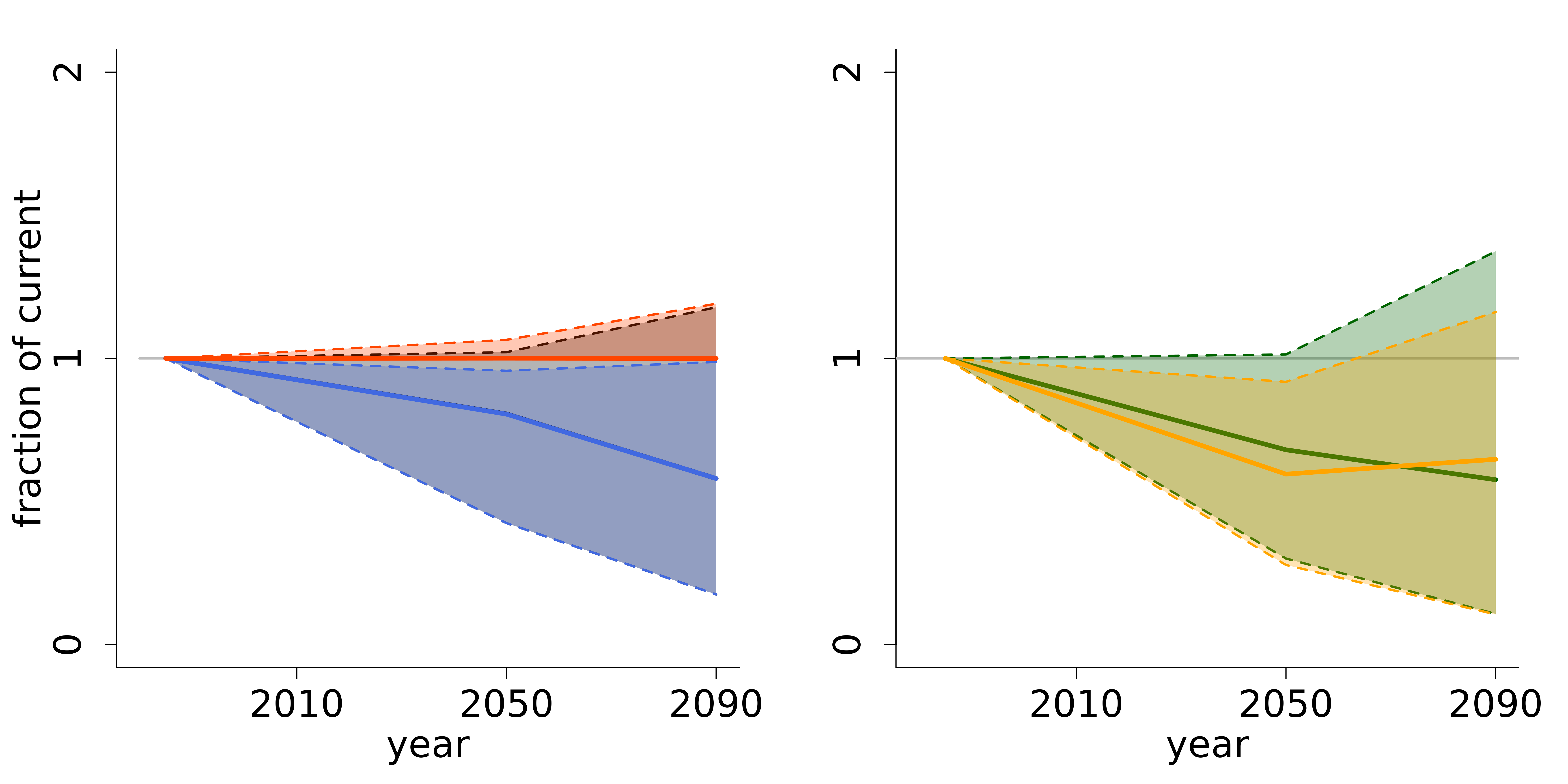

Supplement: S2 Appendix — (ZIP) [file pntd.0014030.s006.zip › Sup. Mat. 6-1 A-L - Species Trends/Acanthophis_wellsi_CCTrends.png]

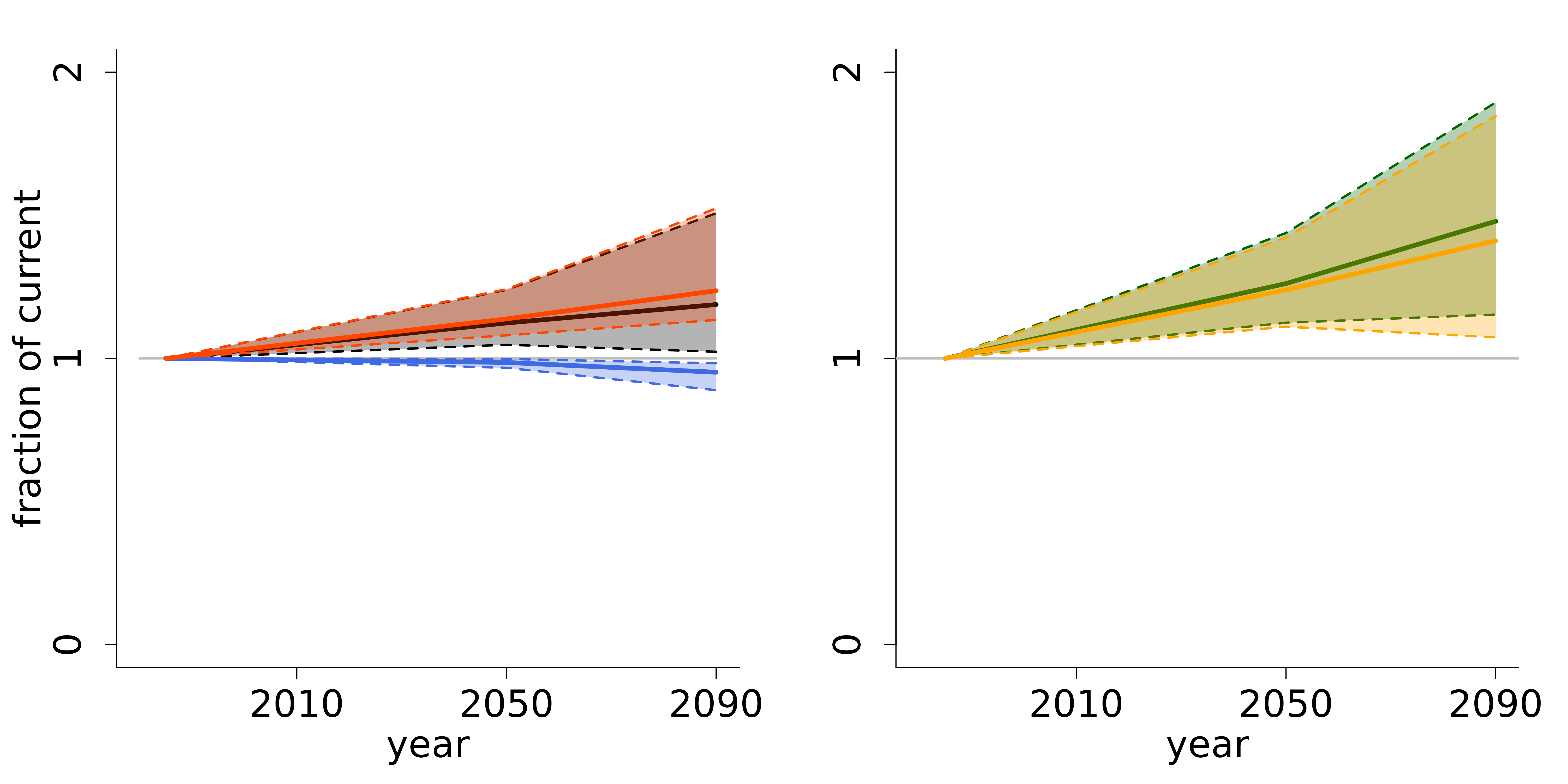

Supplement: S2 Appendix — (ZIP) [file pntd.0014030.s006.zip › Sup. Mat. 6-1 A-L - Species Trends/Agkistrodon_bilineatus_CCTrends.png]

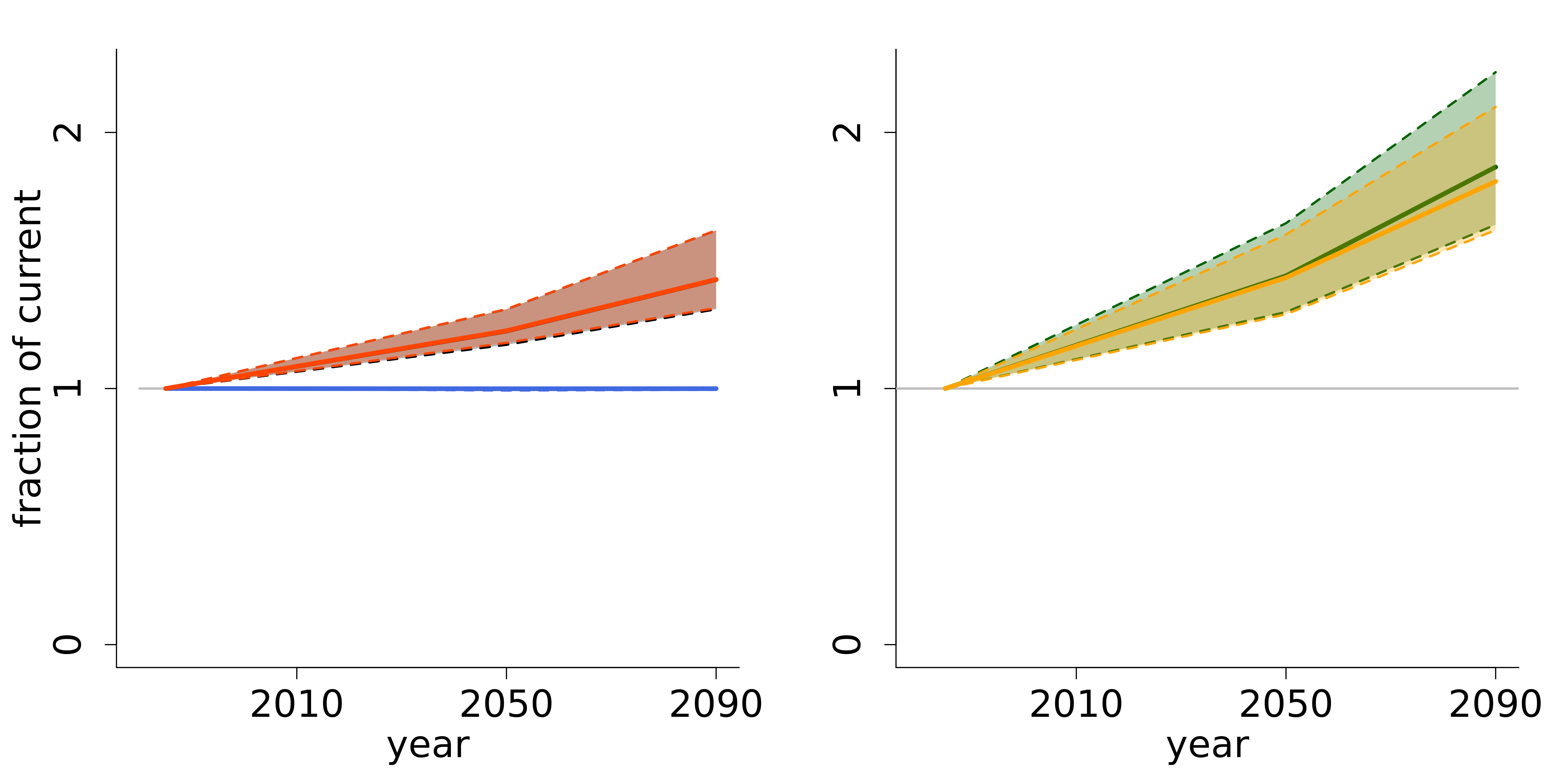

Supplement: S2 Appendix — (ZIP) [file pntd.0014030.s006.zip › Sup. Mat. 6-1 A-L - Species Trends/Agkistrodon_contortrix_CCTrends.png]

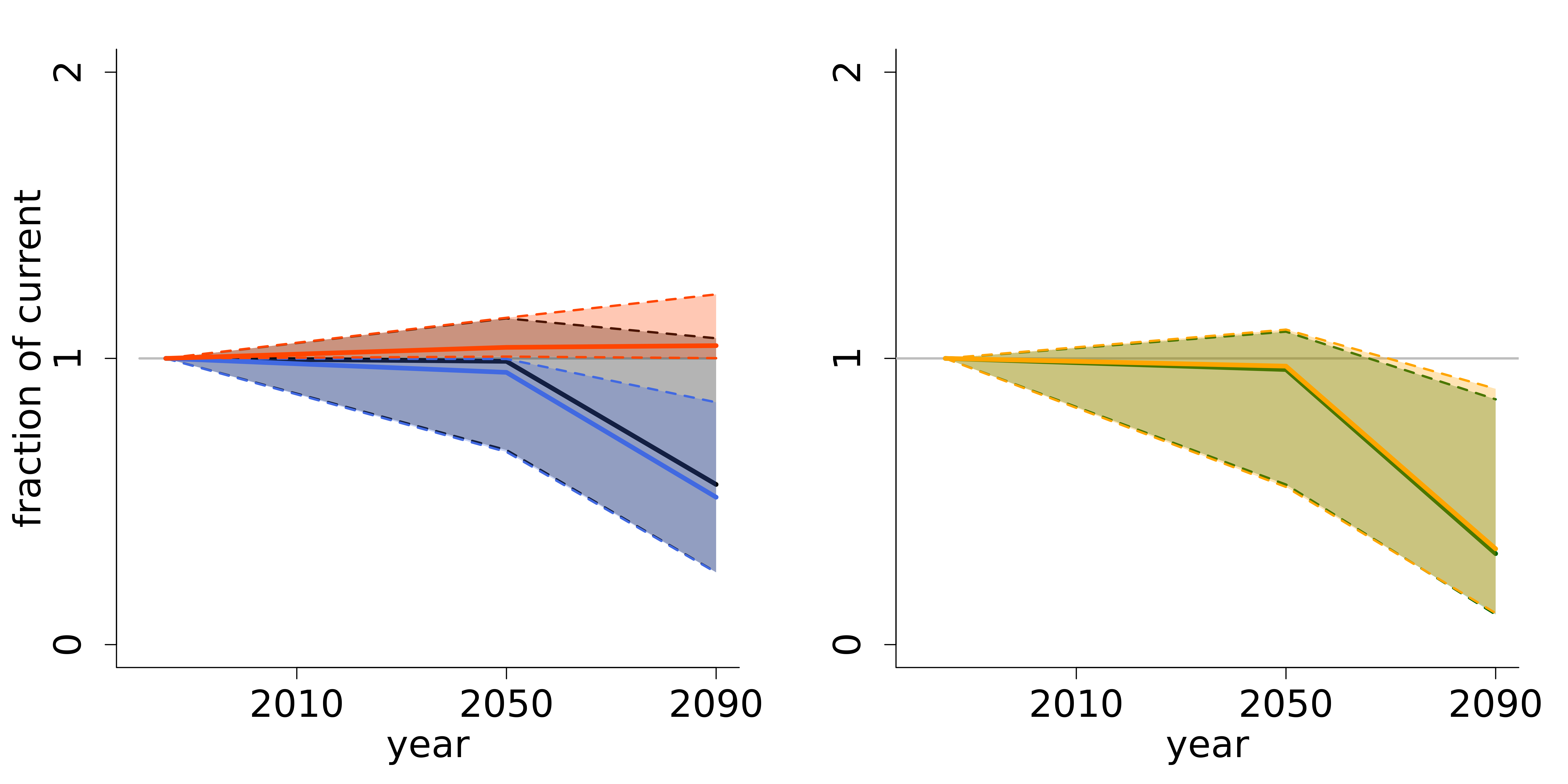

Supplement: S2 Appendix — (ZIP) [file pntd.0014030.s006.zip › Sup. Mat. 6-1 A-L - Species Trends/Agkistrodon_howardgloydi_CCTrends.png]

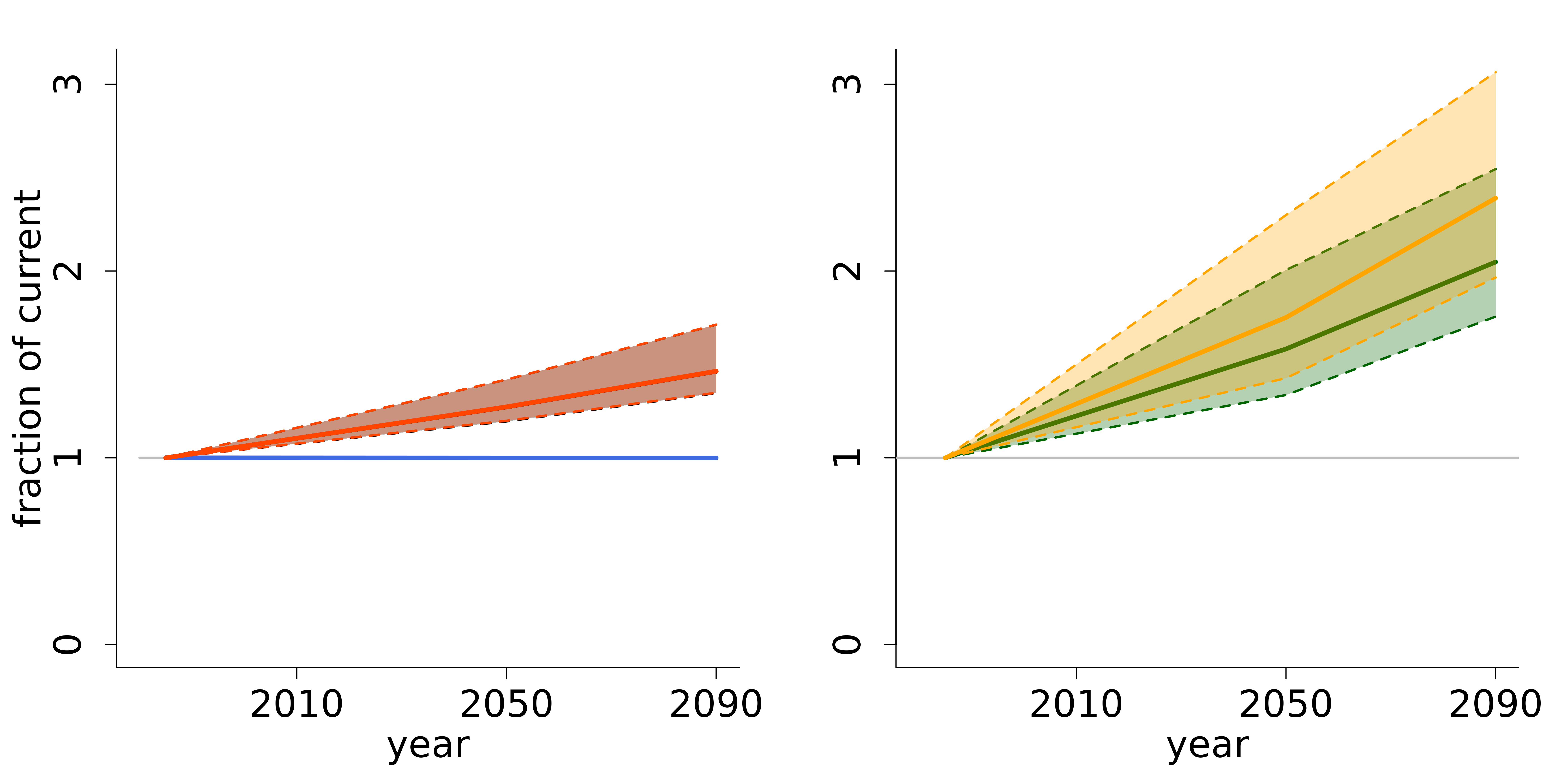

Supplement: S2 Appendix — (ZIP) [file pntd.0014030.s006.zip › Sup. Mat. 6-1 A-L - Species Trends/Agkistrodon_piscivorus_CCTrends.png]

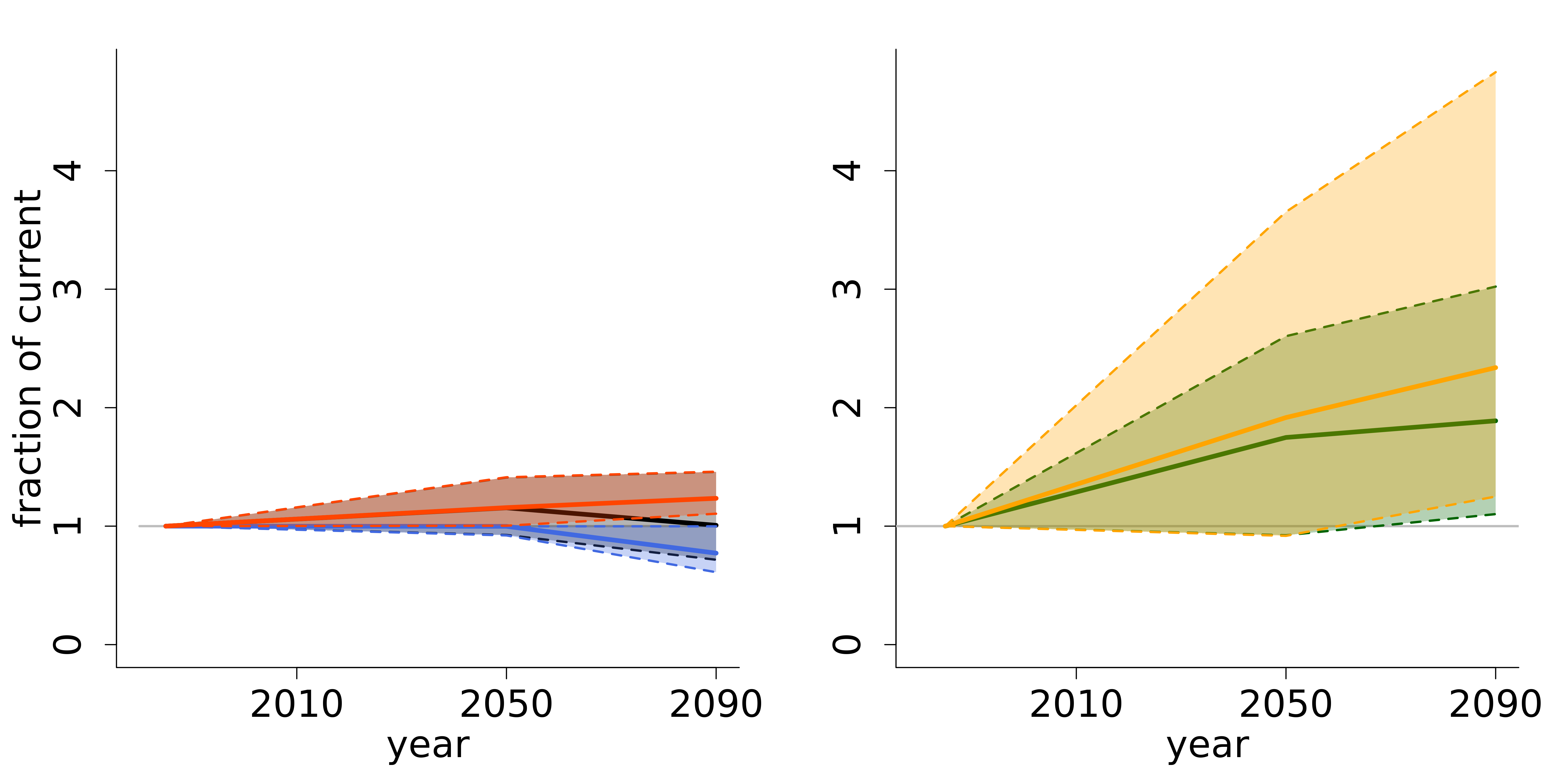

Supplement: S2 Appendix — (ZIP) [file pntd.0014030.s006.zip › Sup. Mat. 6-1 A-L - Species Trends/Agkistrodon_russeolus_CCTrends.png]

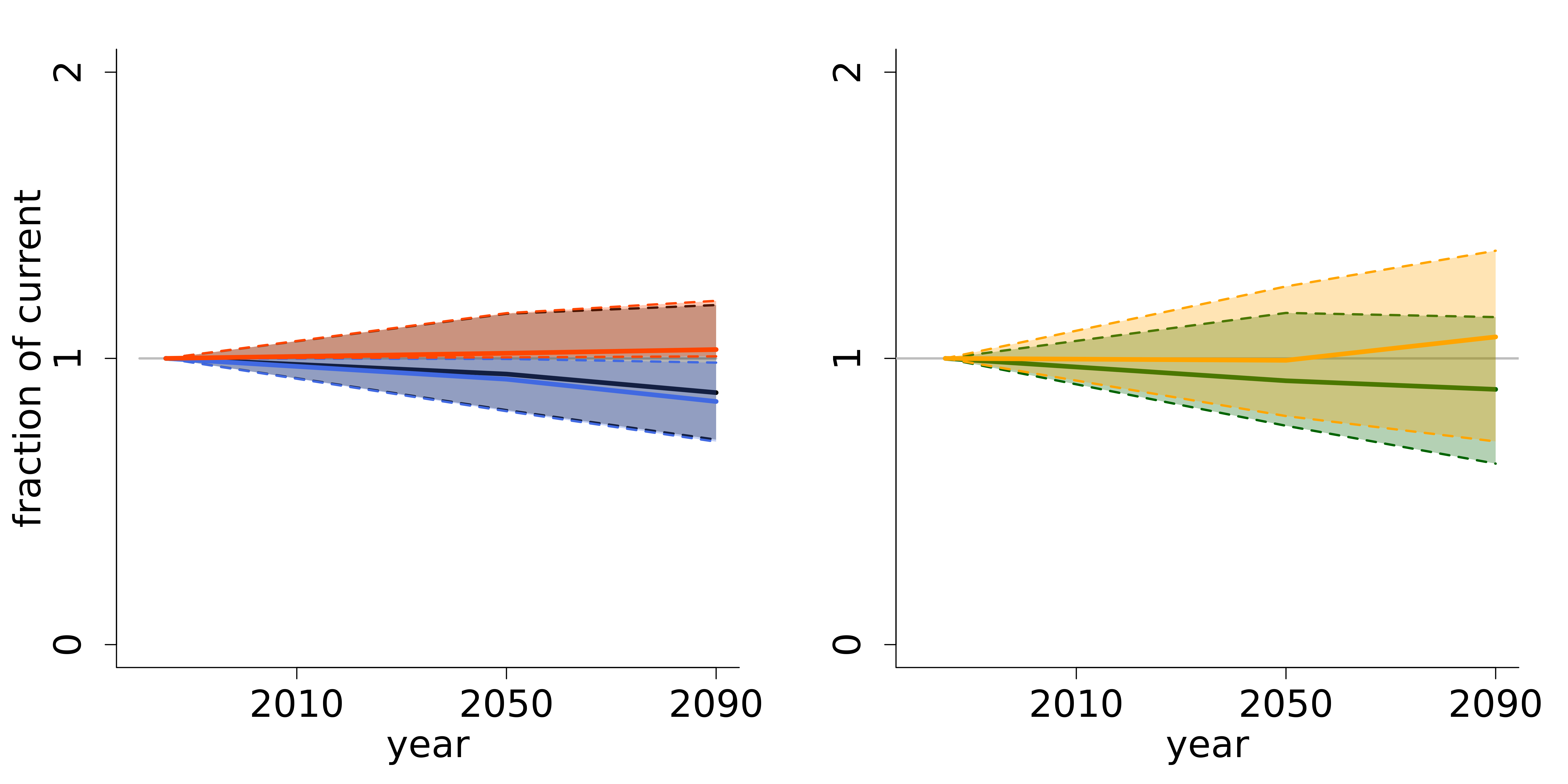

Supplement: S2 Appendix — (ZIP) [file pntd.0014030.s006.zip › Sup. Mat. 6-1 A-L - Species Trends/Agkistrodon_taylori_CCTrends.png]

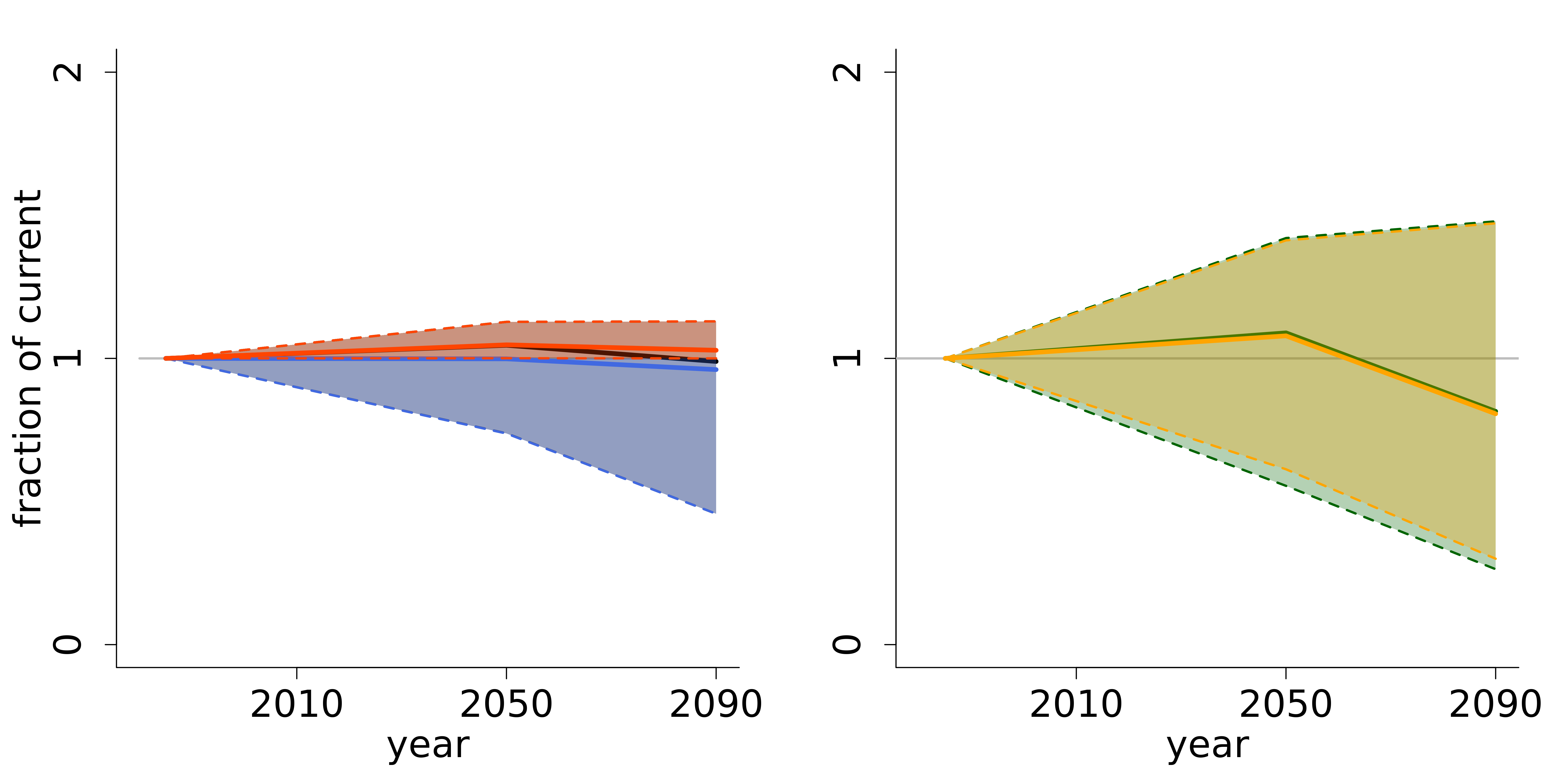

Supplement: S2 Appendix — (ZIP) [file pntd.0014030.s006.zip › Sup. Mat. 6-1 A-L - Species Trends/Atheris_acuminata_CCTrends.png]

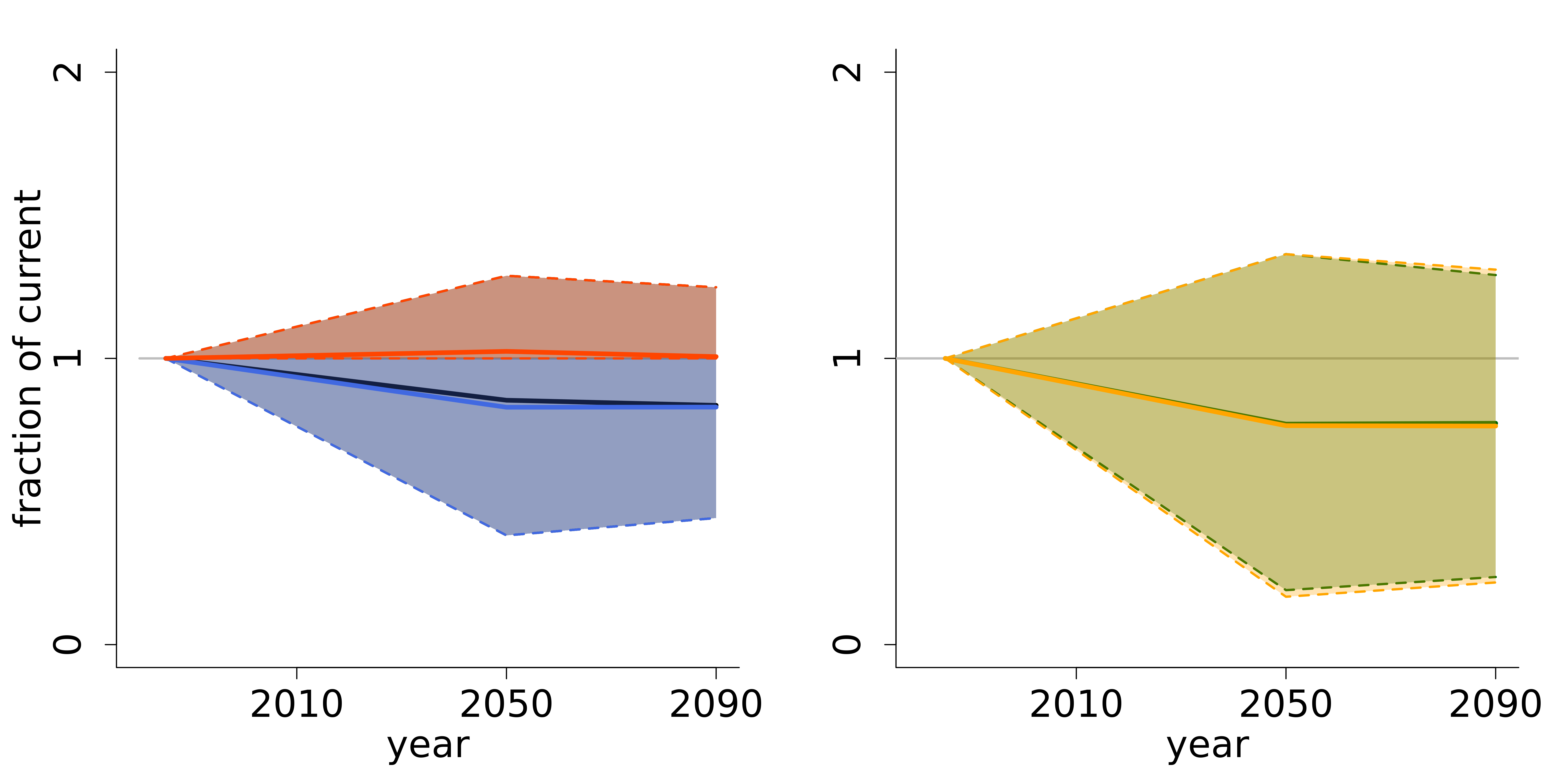

Supplement: S2 Appendix — (ZIP) [file pntd.0014030.s006.zip › Sup. Mat. 6-1 A-L - Species Trends/Atheris_barbouri_CCTrends.png]

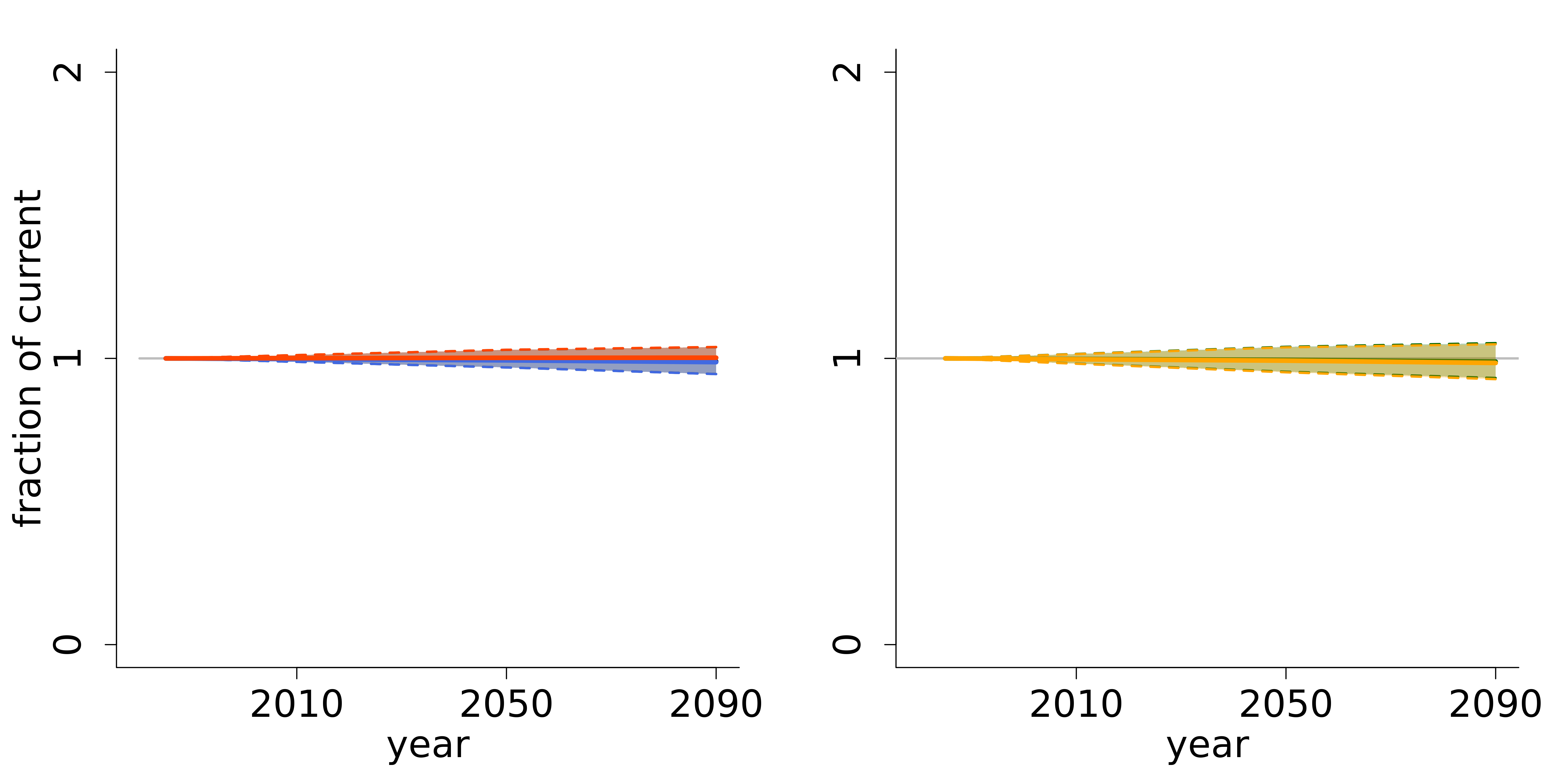

Supplement: S2 Appendix — (ZIP) [file pntd.0014030.s006.zip › Sup. Mat. 6-1 A-L - Species Trends/Atheris_broadleyi_CCTrends.png]

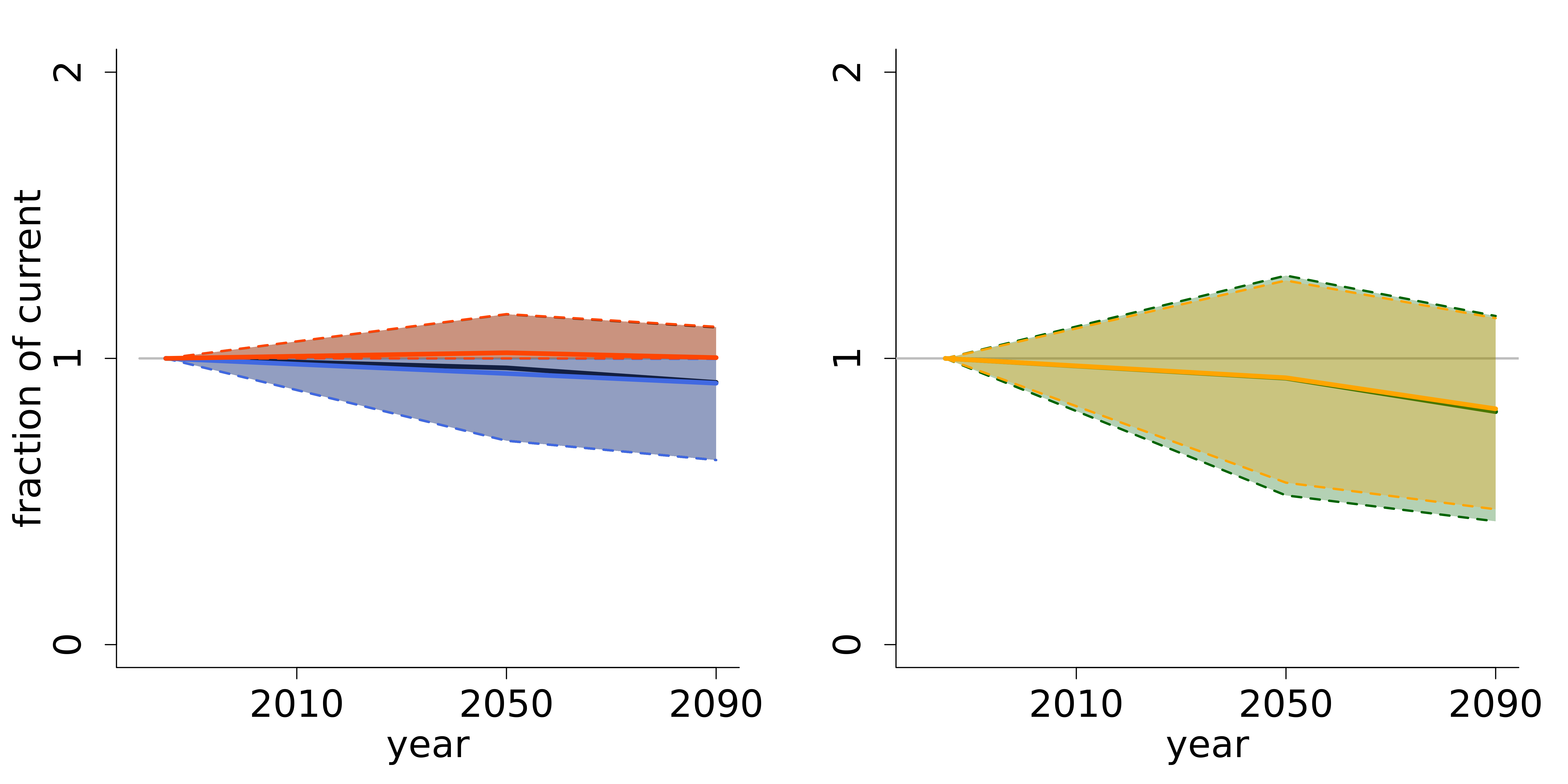

Supplement: S2 Appendix — (ZIP) [file pntd.0014030.s006.zip › Sup. Mat. 6-1 A-L - Species Trends/Atheris_ceratophora_CCTrends.png]

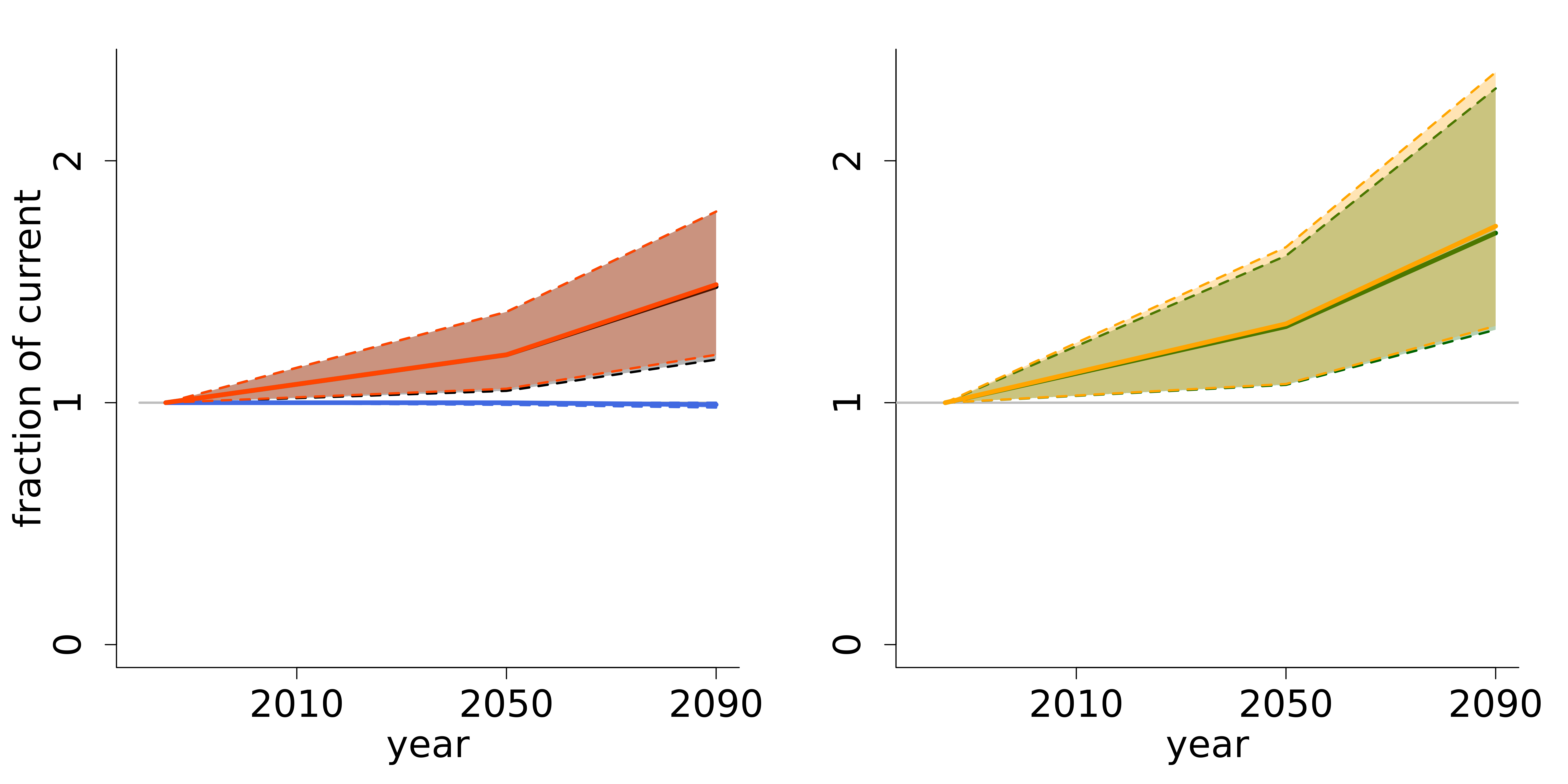

Supplement: S2 Appendix — (ZIP) [file pntd.0014030.s006.zip › Sup. Mat. 6-1 A-L - Species Trends/Atheris_chlorechis_CCTrends.png]

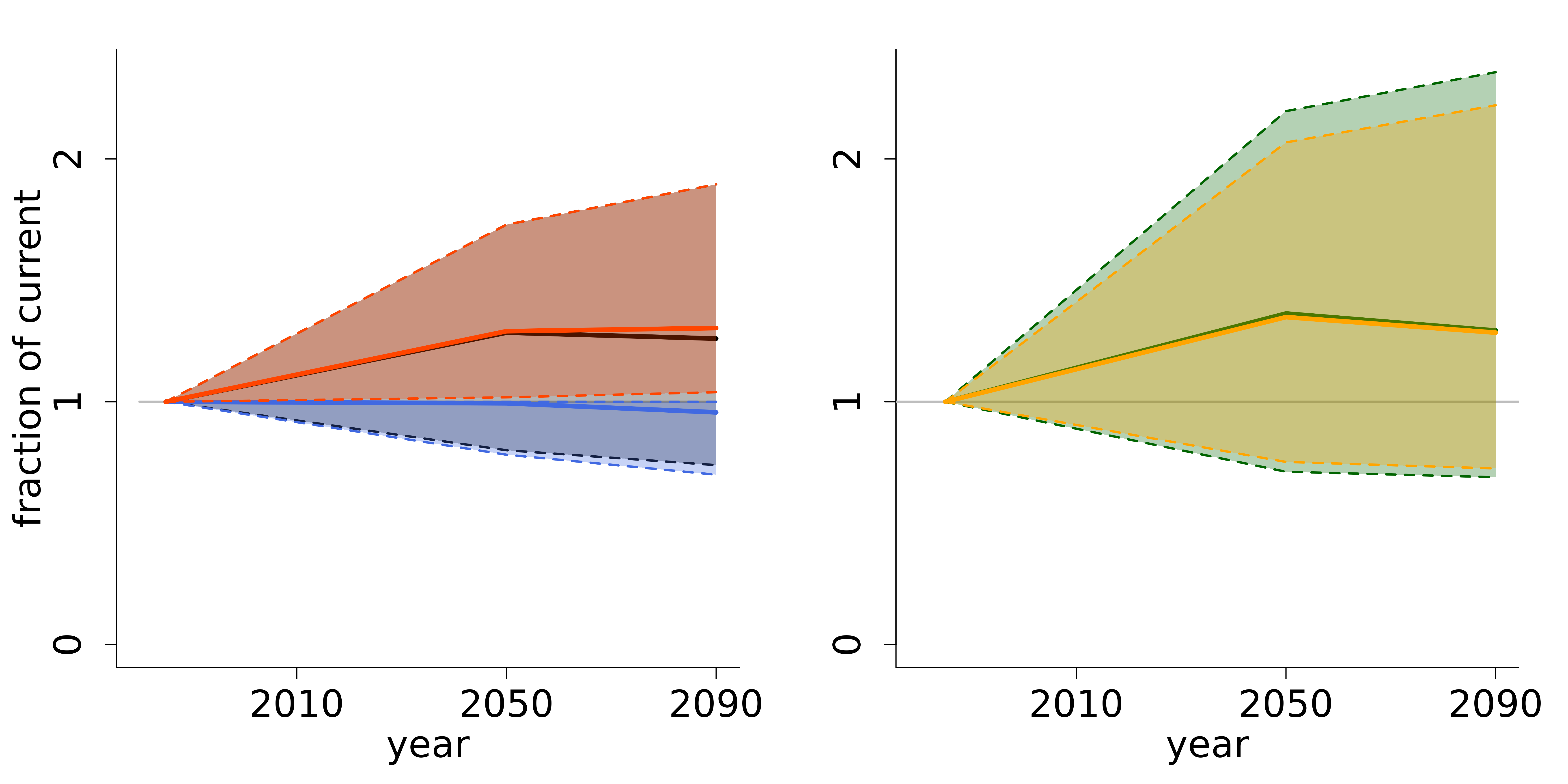

Supplement: S2 Appendix — (ZIP) [file pntd.0014030.s006.zip › Sup. Mat. 6-1 A-L - Species Trends/Atheris_desaixi_CCTrends.png]

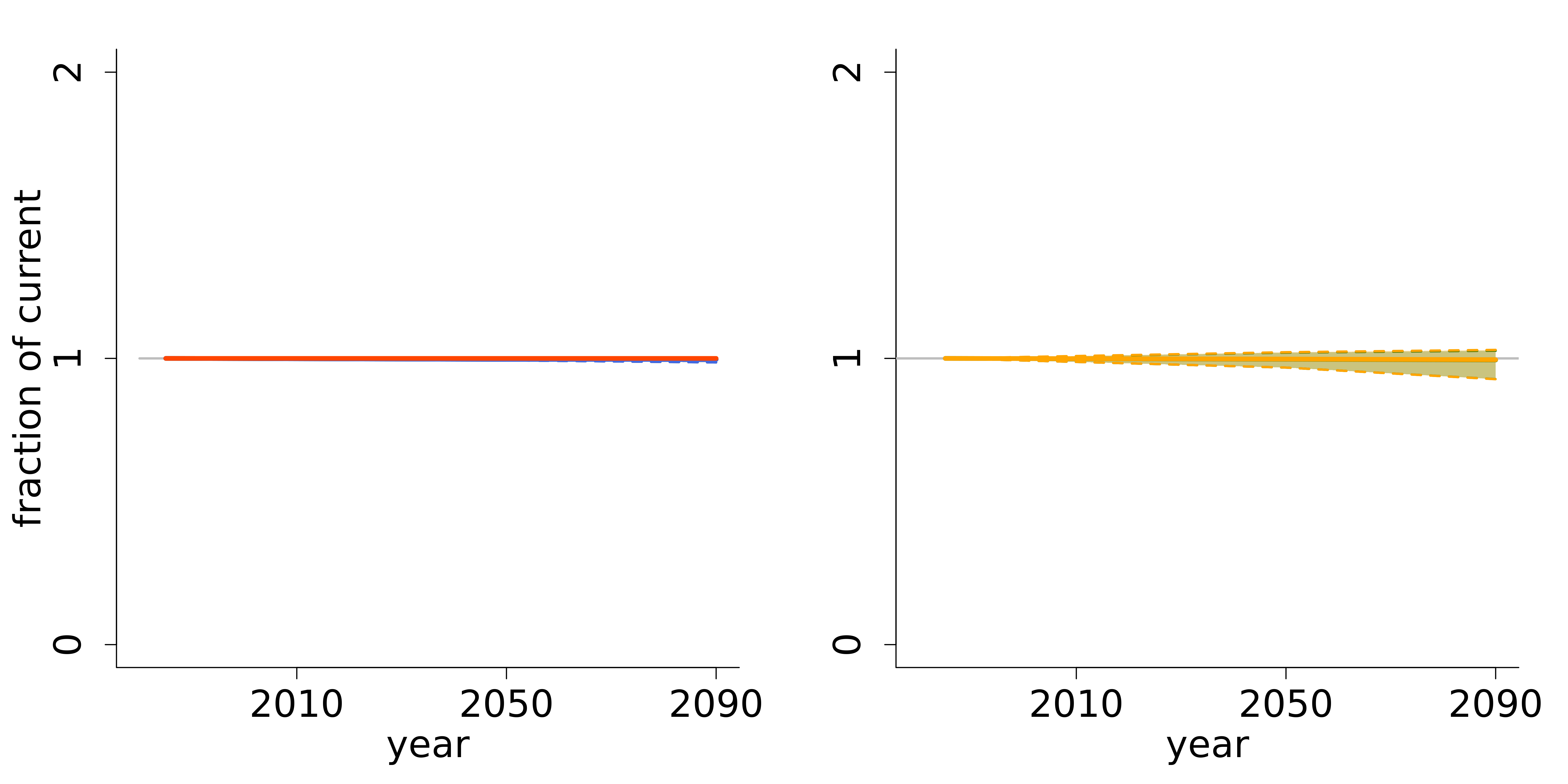

Supplement: S2 Appendix — (ZIP) [file pntd.0014030.s006.zip › Sup. Mat. 6-1 A-L - Species Trends/Atheris_hetfieldi_CCTrends.png]

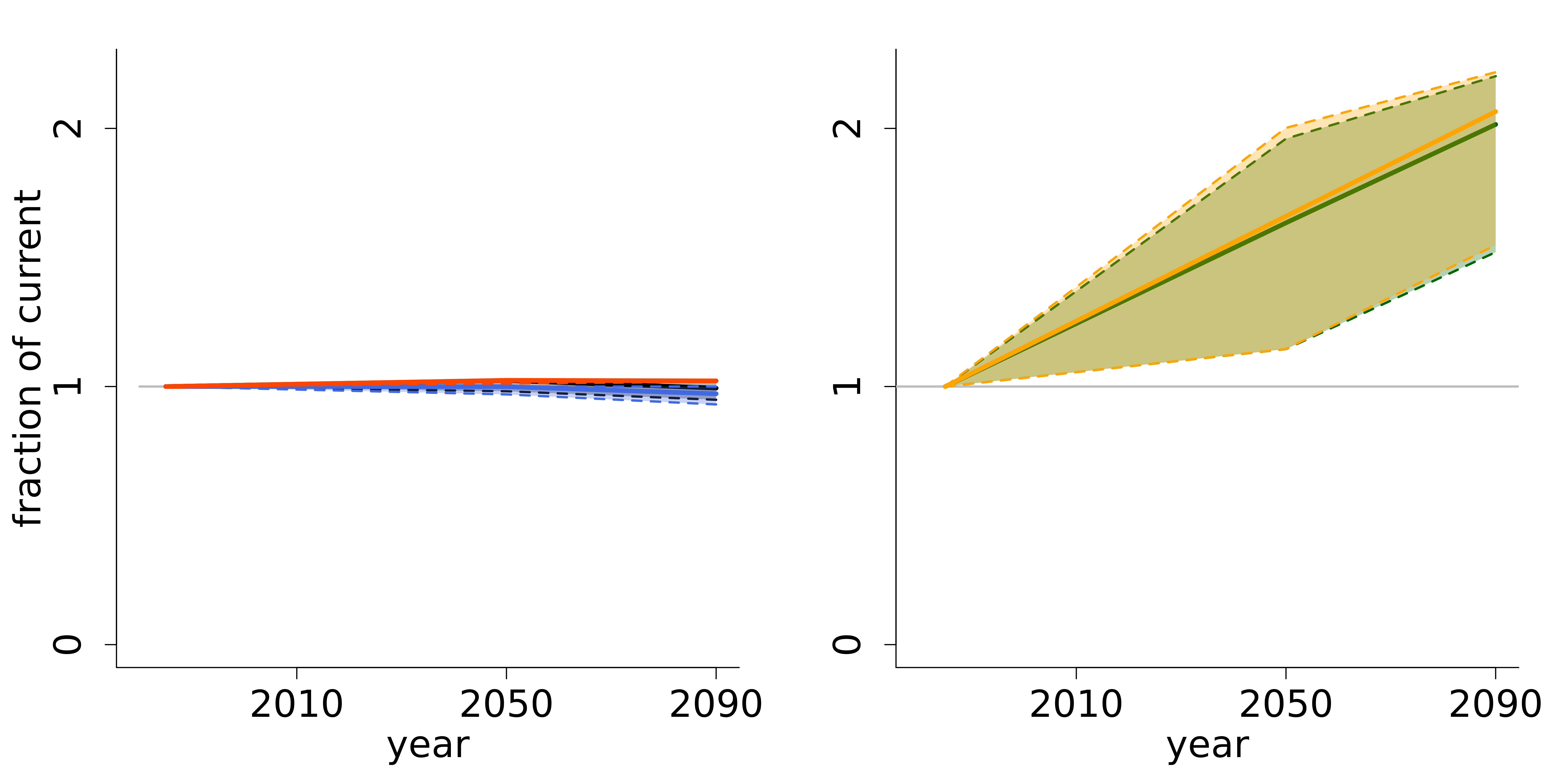

Supplement: S2 Appendix — (ZIP) [file pntd.0014030.s006.zip › Sup. Mat. 6-1 A-L - Species Trends/Atheris_hirsuta_CCTrends.png]

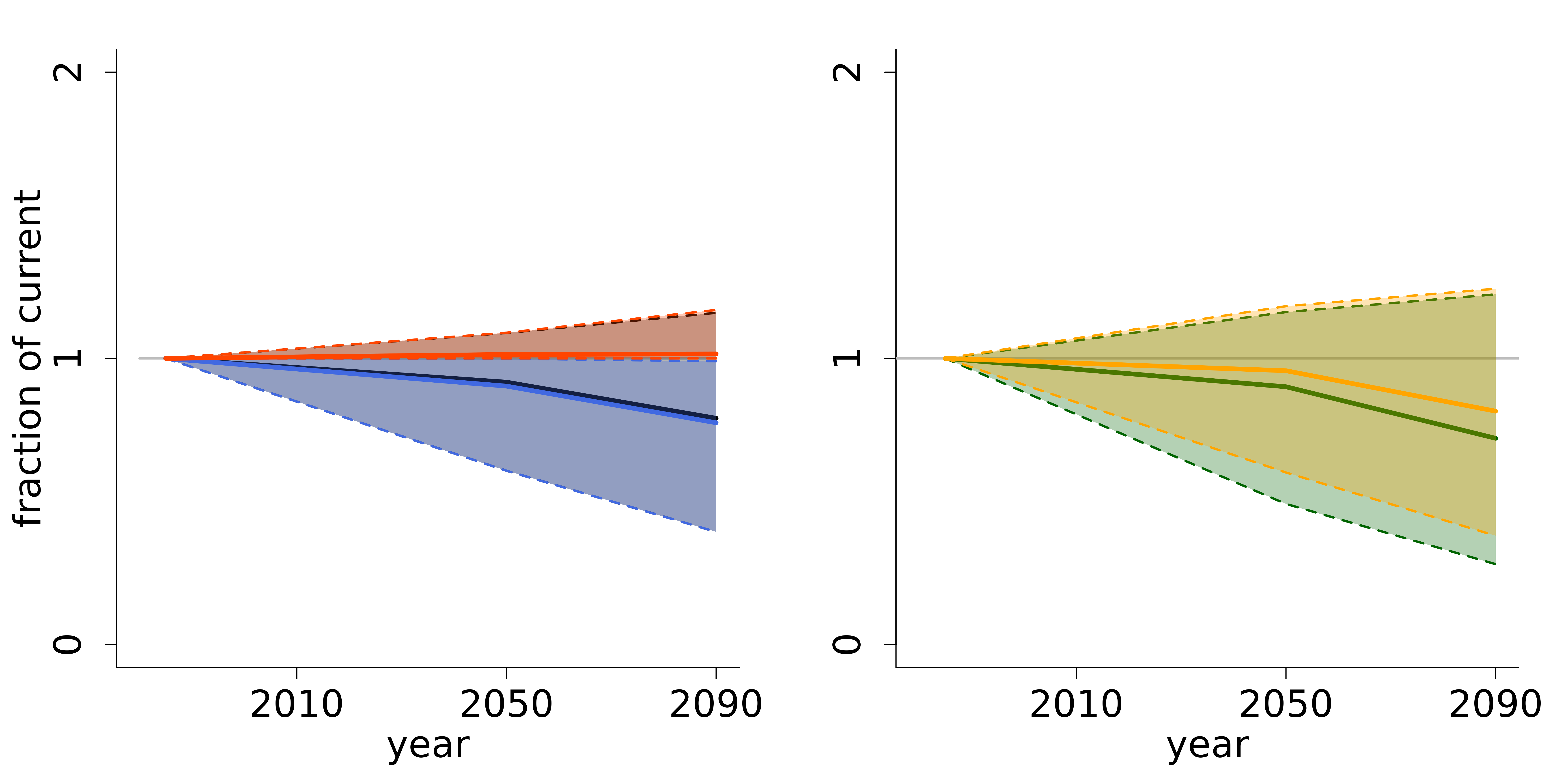

Supplement: S2 Appendix — (ZIP) [file pntd.0014030.s006.zip › Sup. Mat. 6-1 A-L - Species Trends/Atheris_hispida_CCTrends.png]

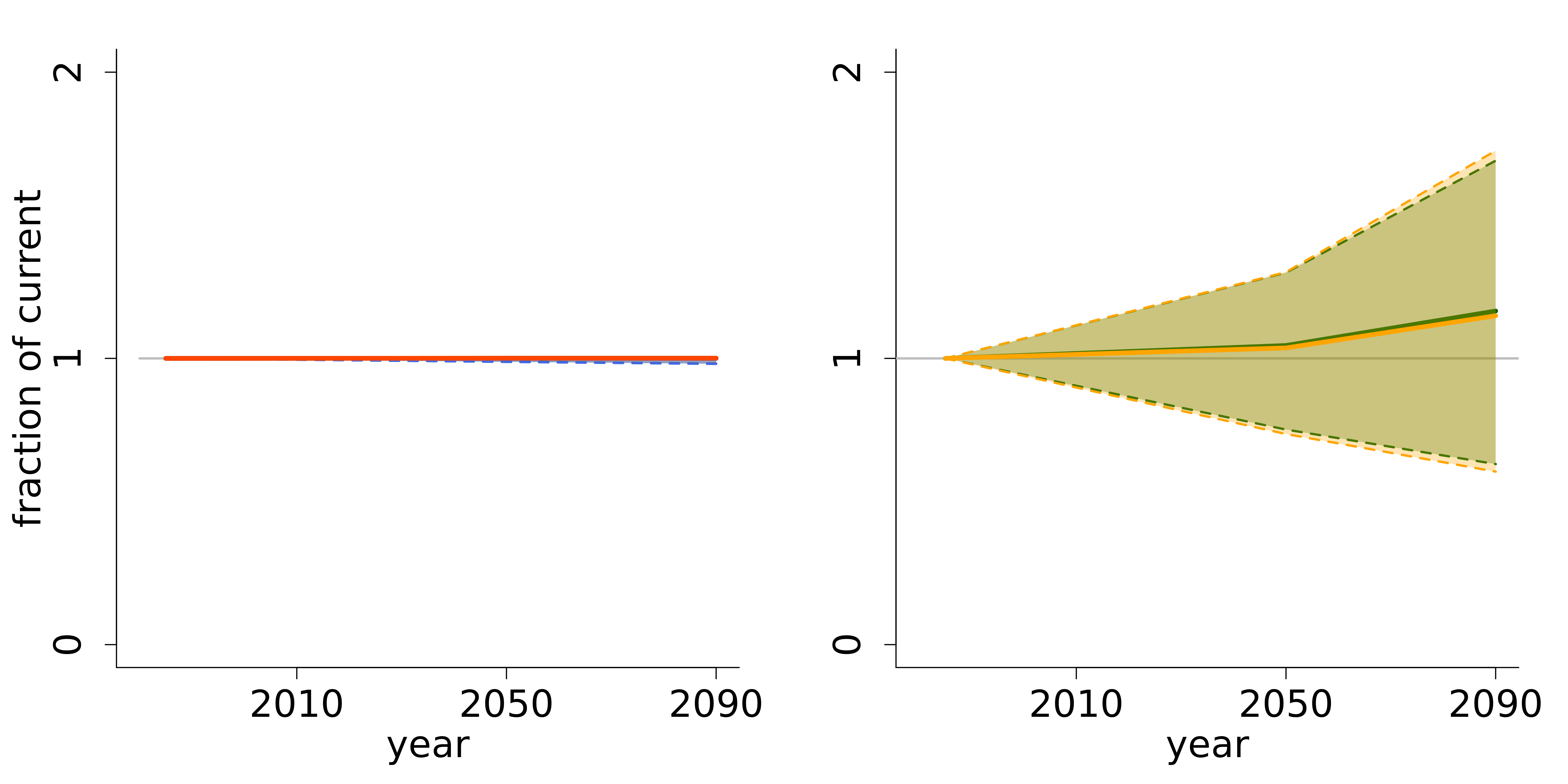

Supplement: S2 Appendix — (ZIP) [file pntd.0014030.s006.zip › Sup. Mat. 6-1 A-L - Species Trends/Atheris_katangensis_CCTrends.png]

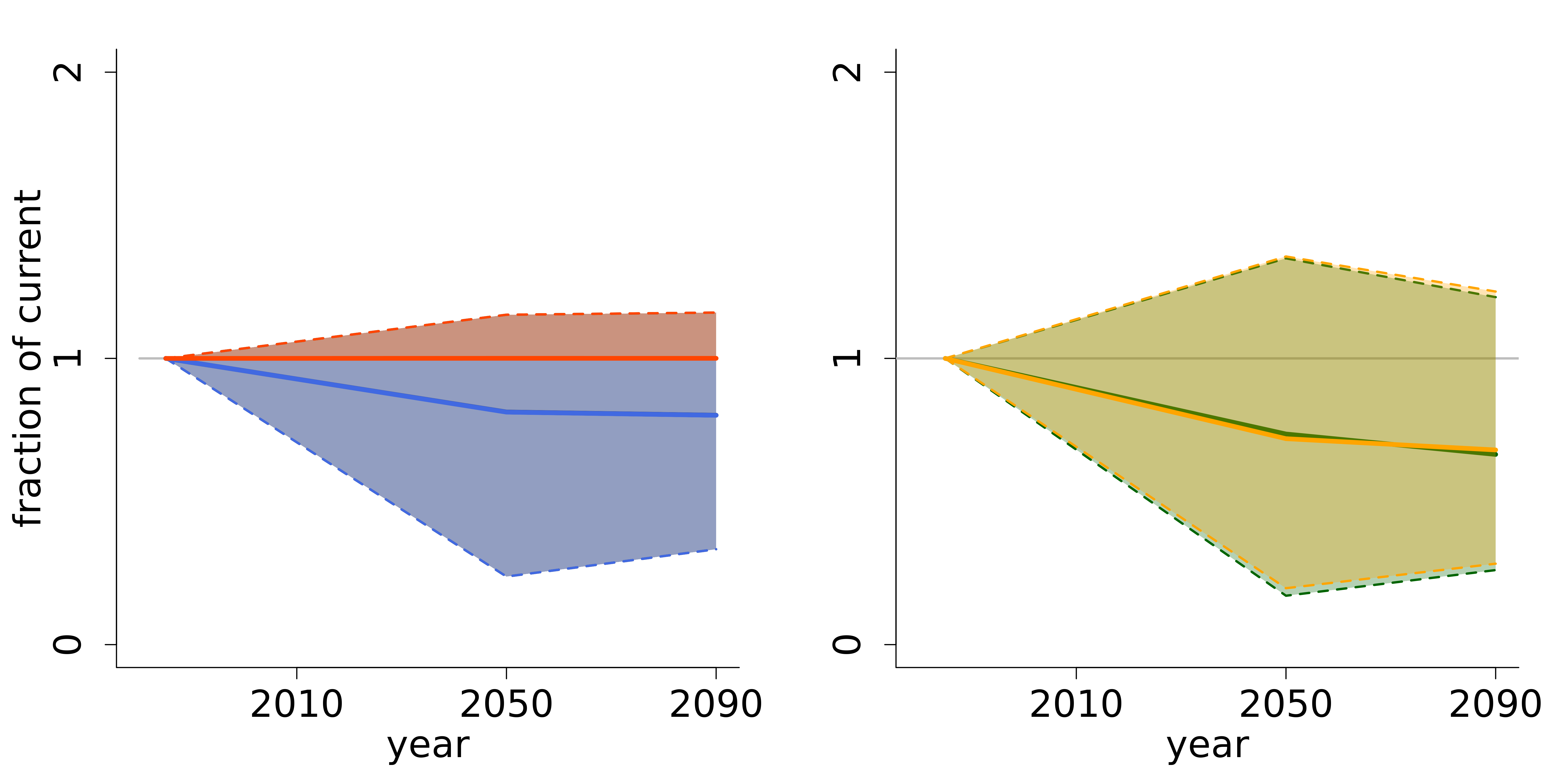

Supplement: S2 Appendix — (ZIP) [file pntd.0014030.s006.zip › Sup. Mat. 6-1 A-L - Species Trends/Atheris_matildae_CCTrends.png]

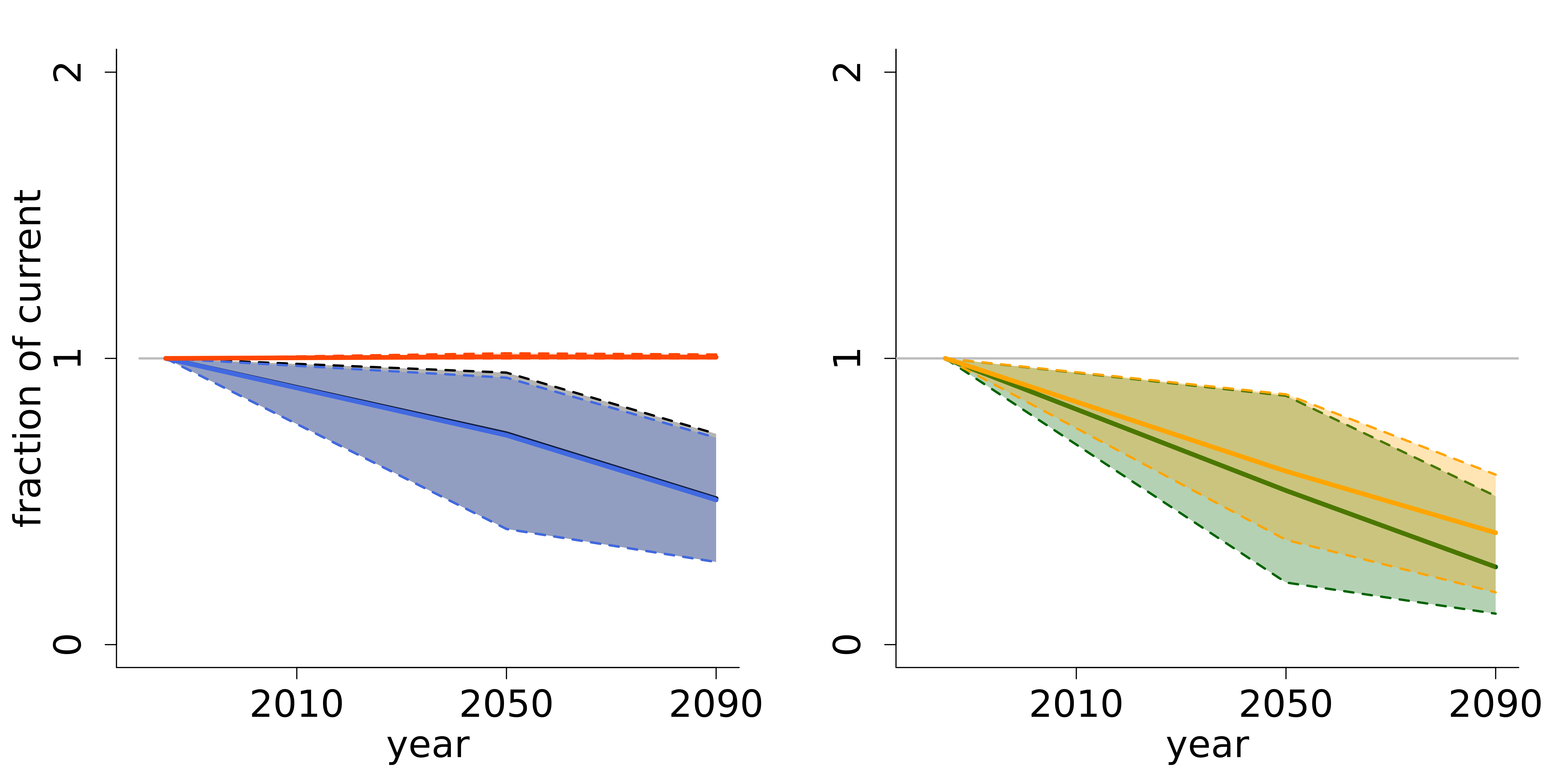

Supplement: S2 Appendix — (ZIP) [file pntd.0014030.s006.zip › Sup. Mat. 6-1 A-L - Species Trends/Atheris_nitschei_CCTrends.png]

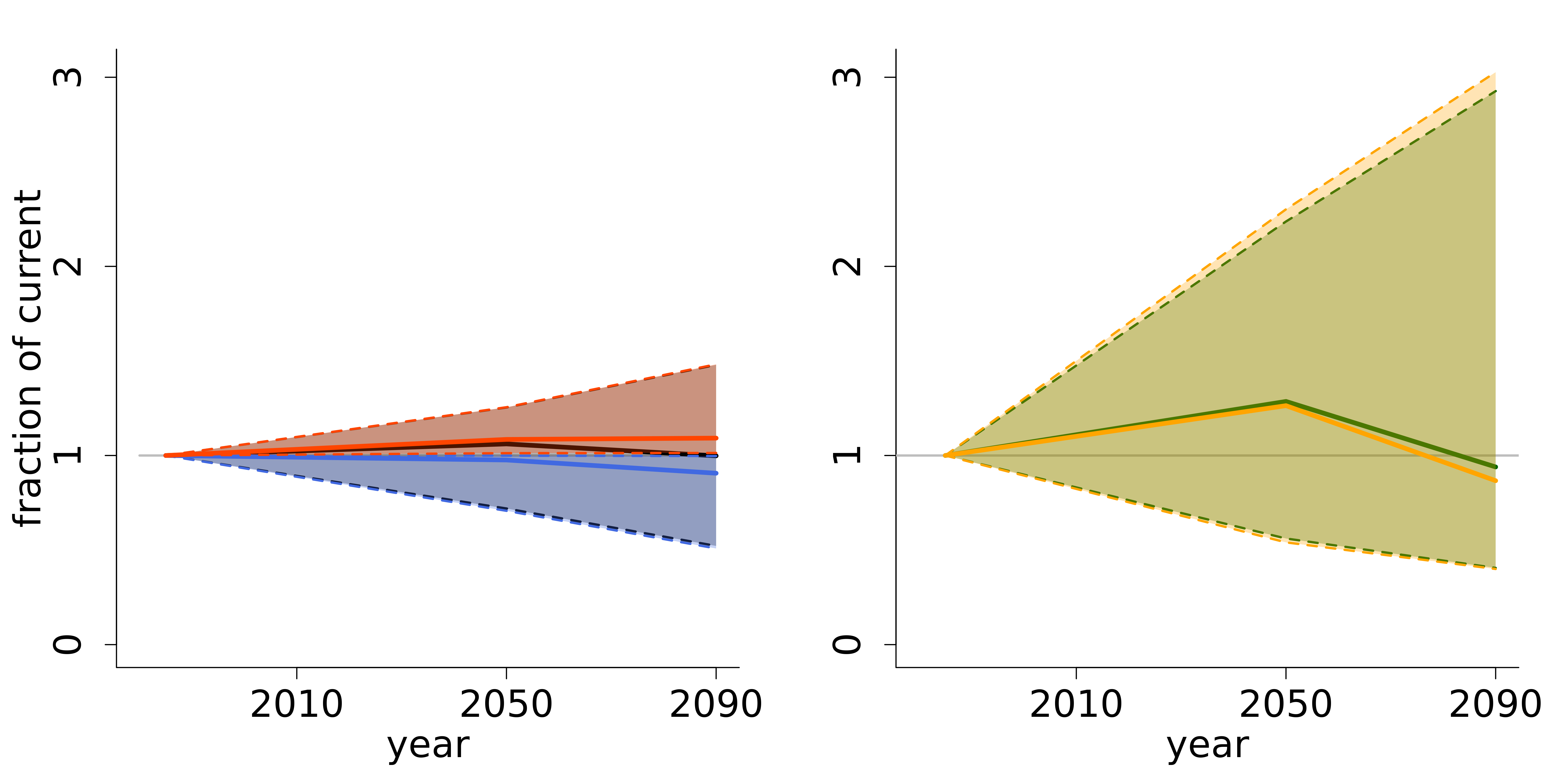

Supplement: S2 Appendix — (ZIP) [file pntd.0014030.s006.zip › Sup. Mat. 6-1 A-L - Species Trends/Atheris_rungweensis_CCTrends.png]

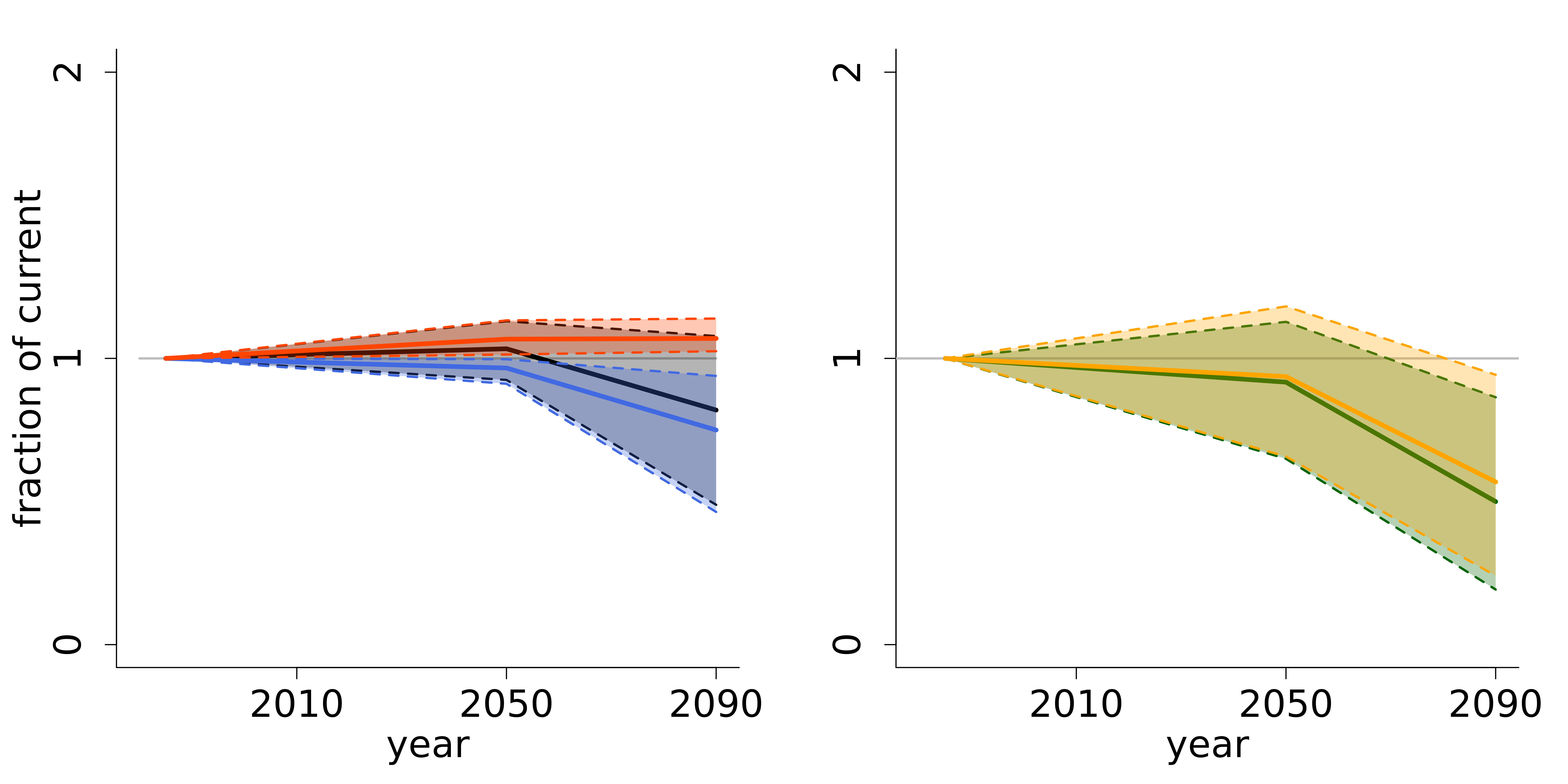

Supplement: S2 Appendix — (ZIP) [file pntd.0014030.s006.zip › Sup. Mat. 6-1 A-L - Species Trends/Atheris_squamigera_CCTrends.png]

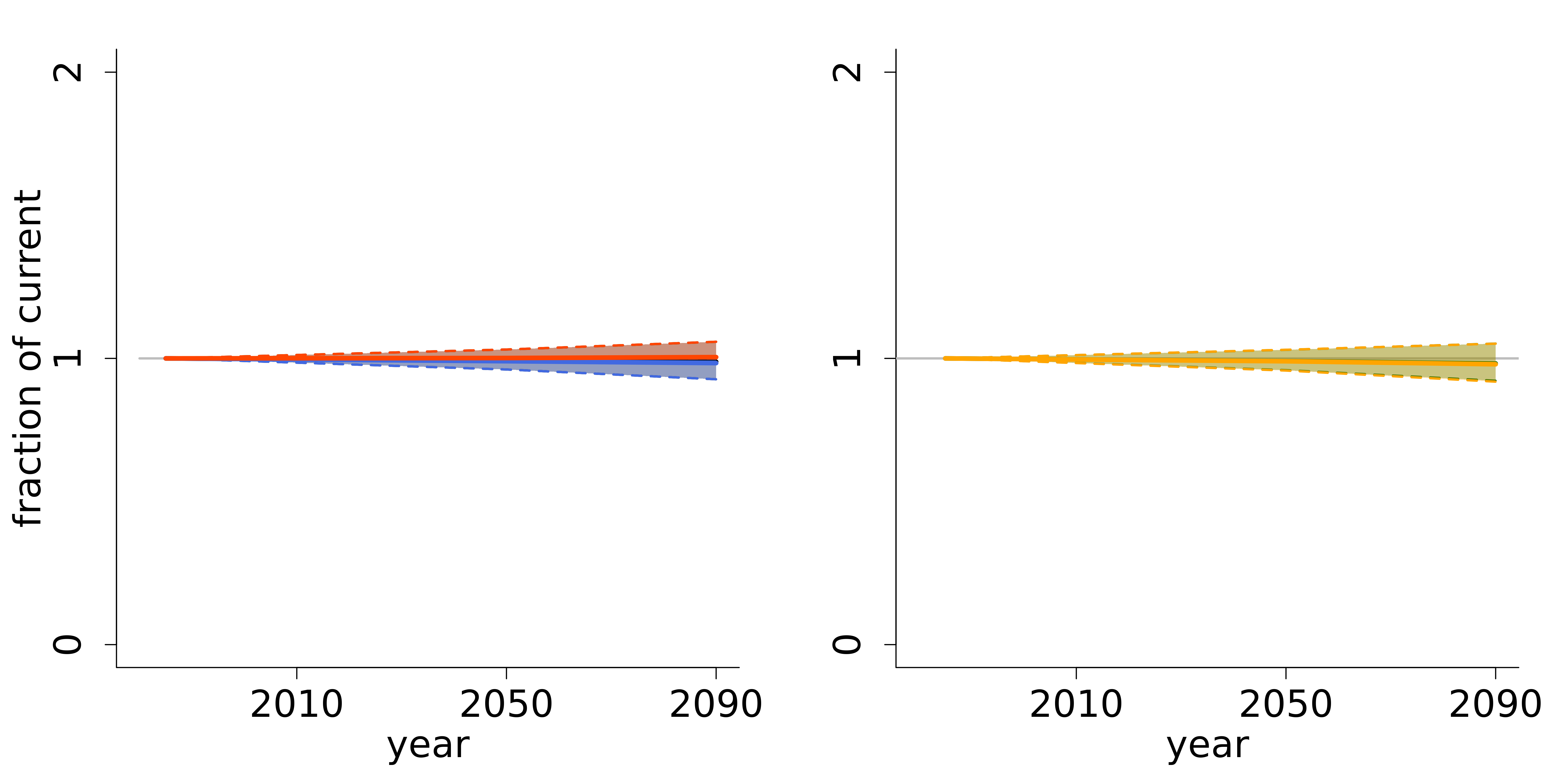

Supplement: S2 Appendix — (ZIP) [file pntd.0014030.s006.zip › Sup. Mat. 6-1 A-L - Species Trends/Atheris_subocularis_CCTrends.png]

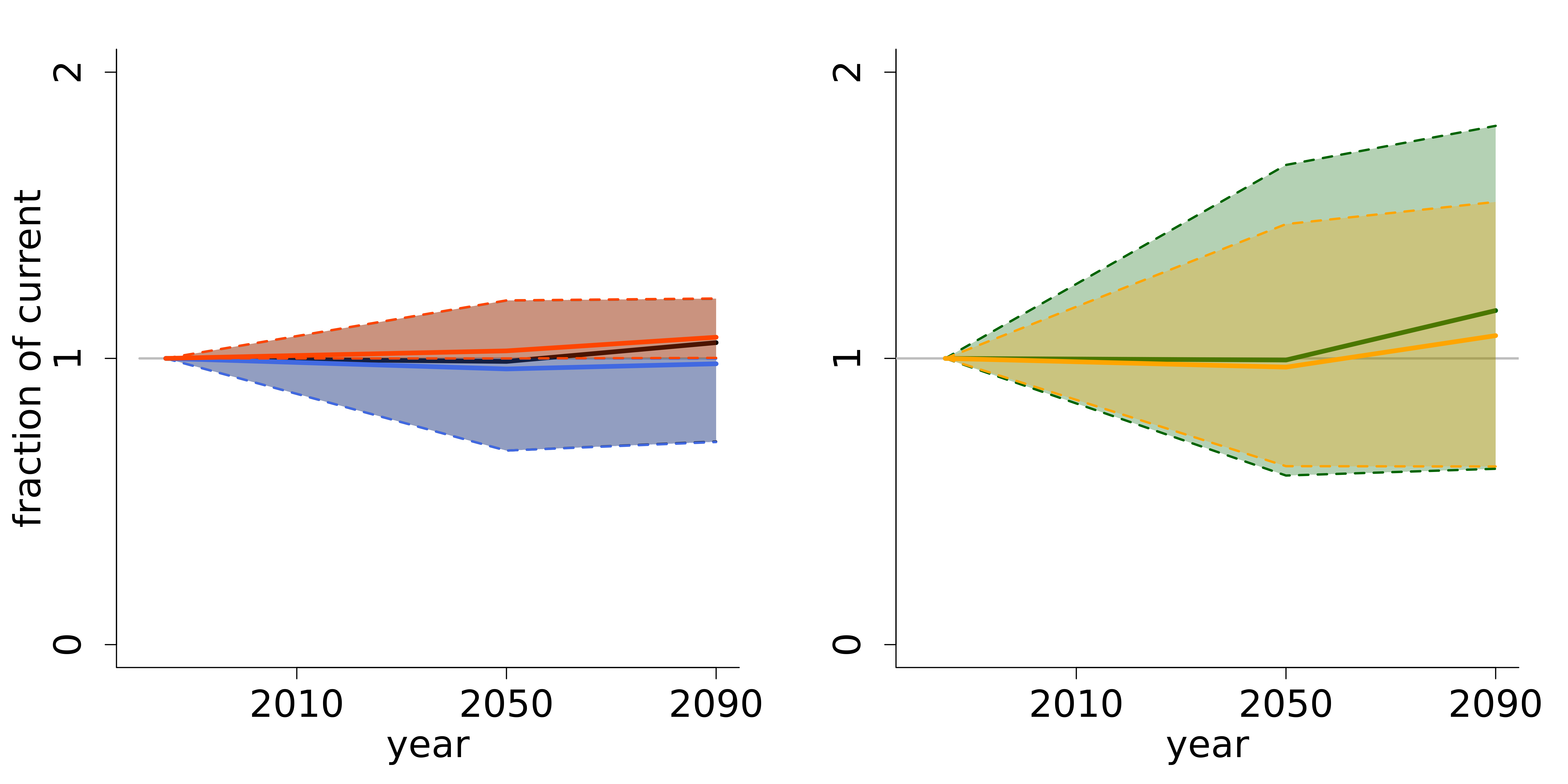

Supplement: S2 Appendix — (ZIP) [file pntd.0014030.s006.zip › Sup. Mat. 6-1 A-L - Species Trends/Atractaspis_andersonii_CCTrends.png]

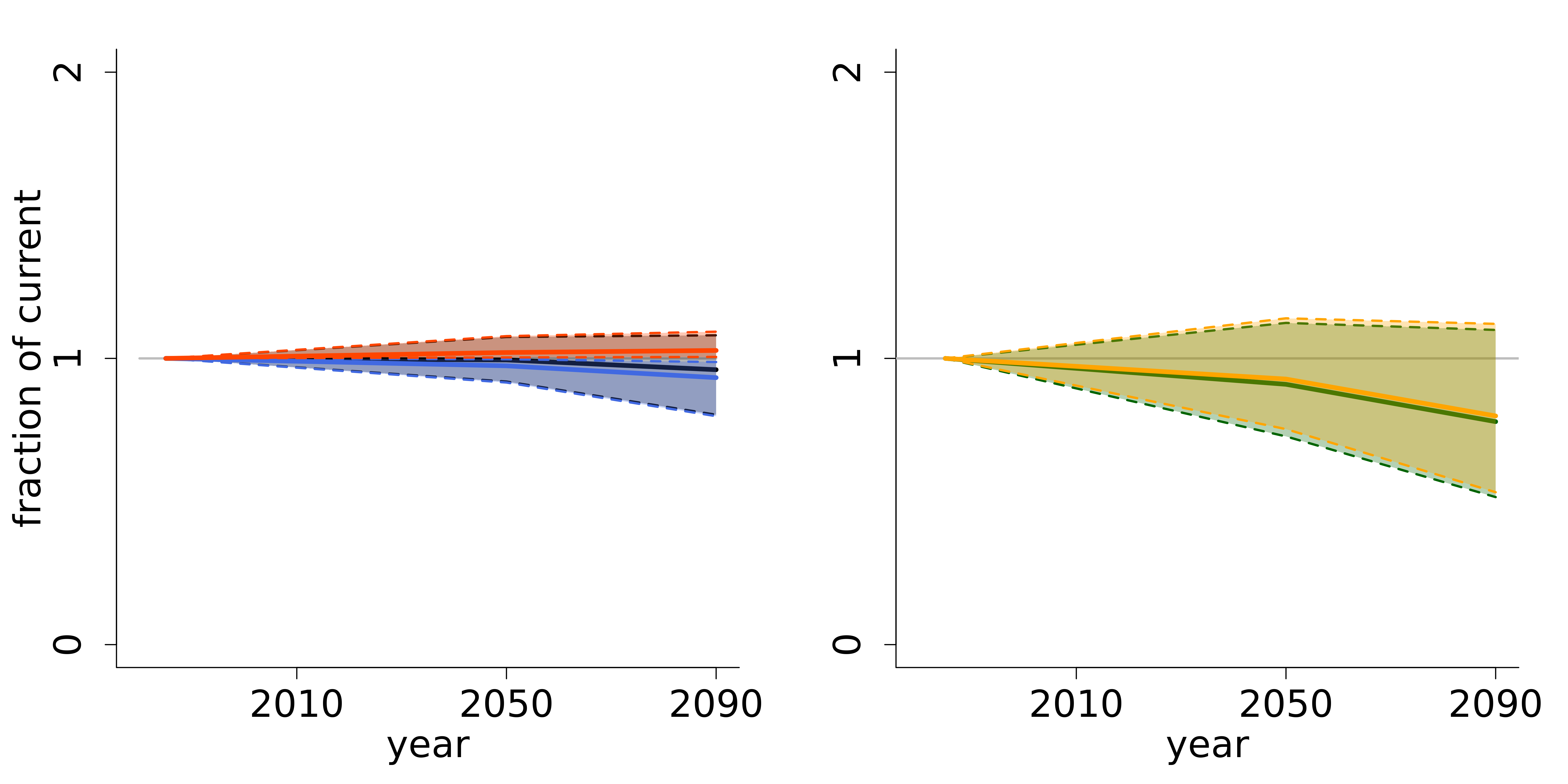

Supplement: S2 Appendix — (ZIP) [file pntd.0014030.s006.zip › Sup. Mat. 6-1 A-L - Species Trends/Atractaspis_bibronii_CCTrends.png]

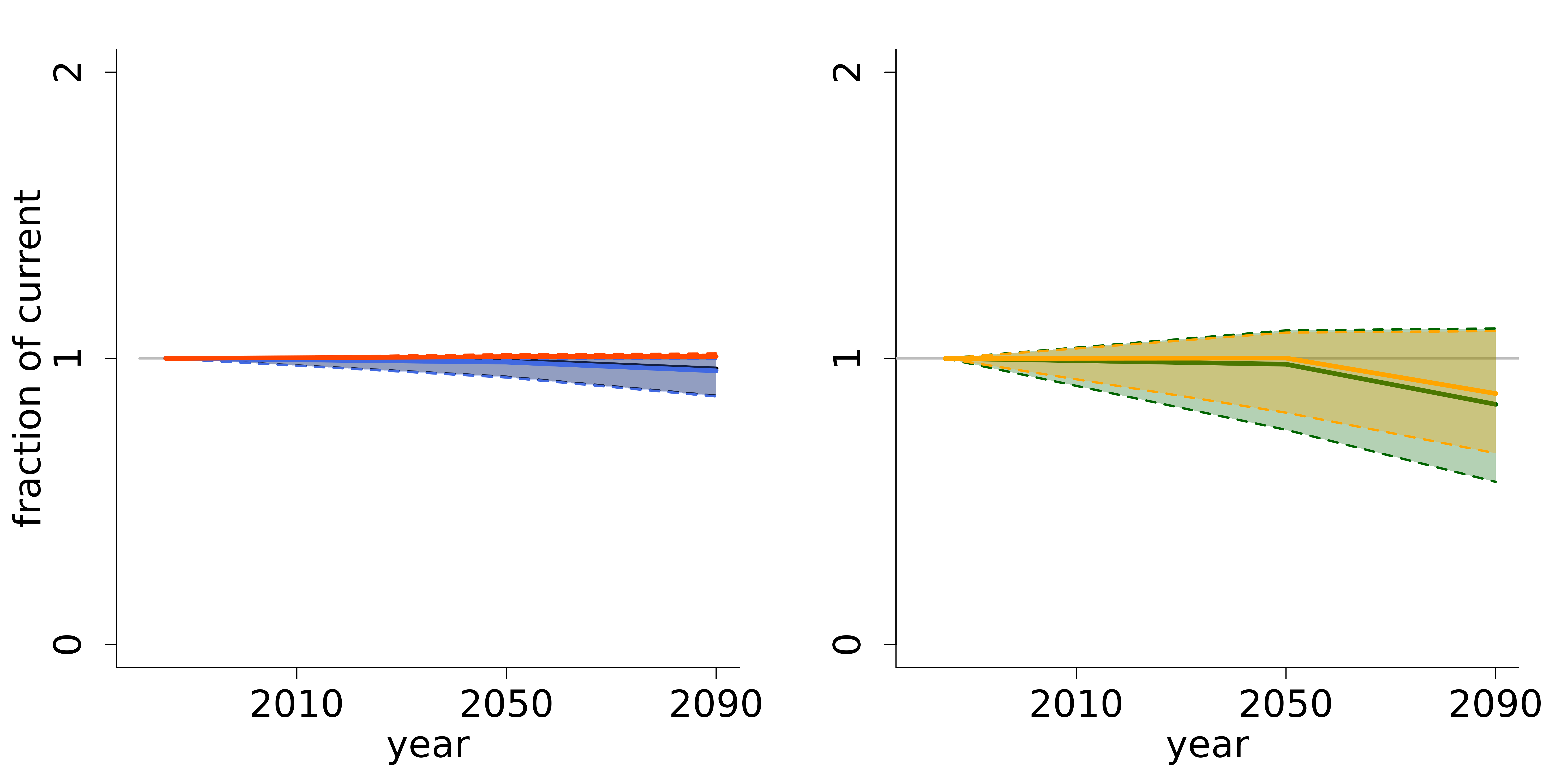

Supplement: S2 Appendix — (ZIP) [file pntd.0014030.s006.zip › Sup. Mat. 6-1 A-L - Species Trends/Atractaspis_duerdeni_CCTrends.png]

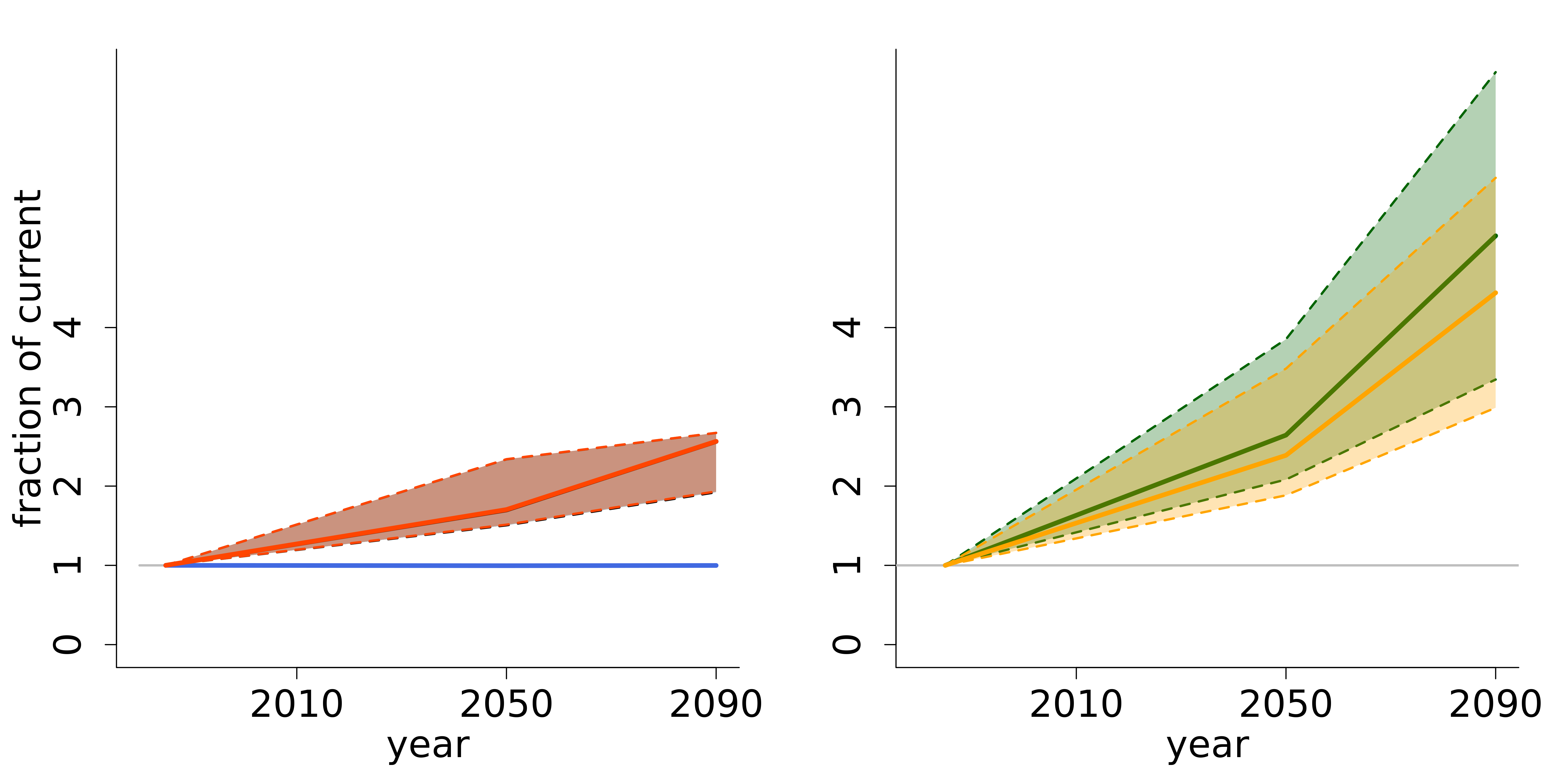

Supplement: S2 Appendix — (ZIP) [file pntd.0014030.s006.zip › Sup. Mat. 6-1 A-L - Species Trends/Atractaspis_engaddensis_CCTrends.png]

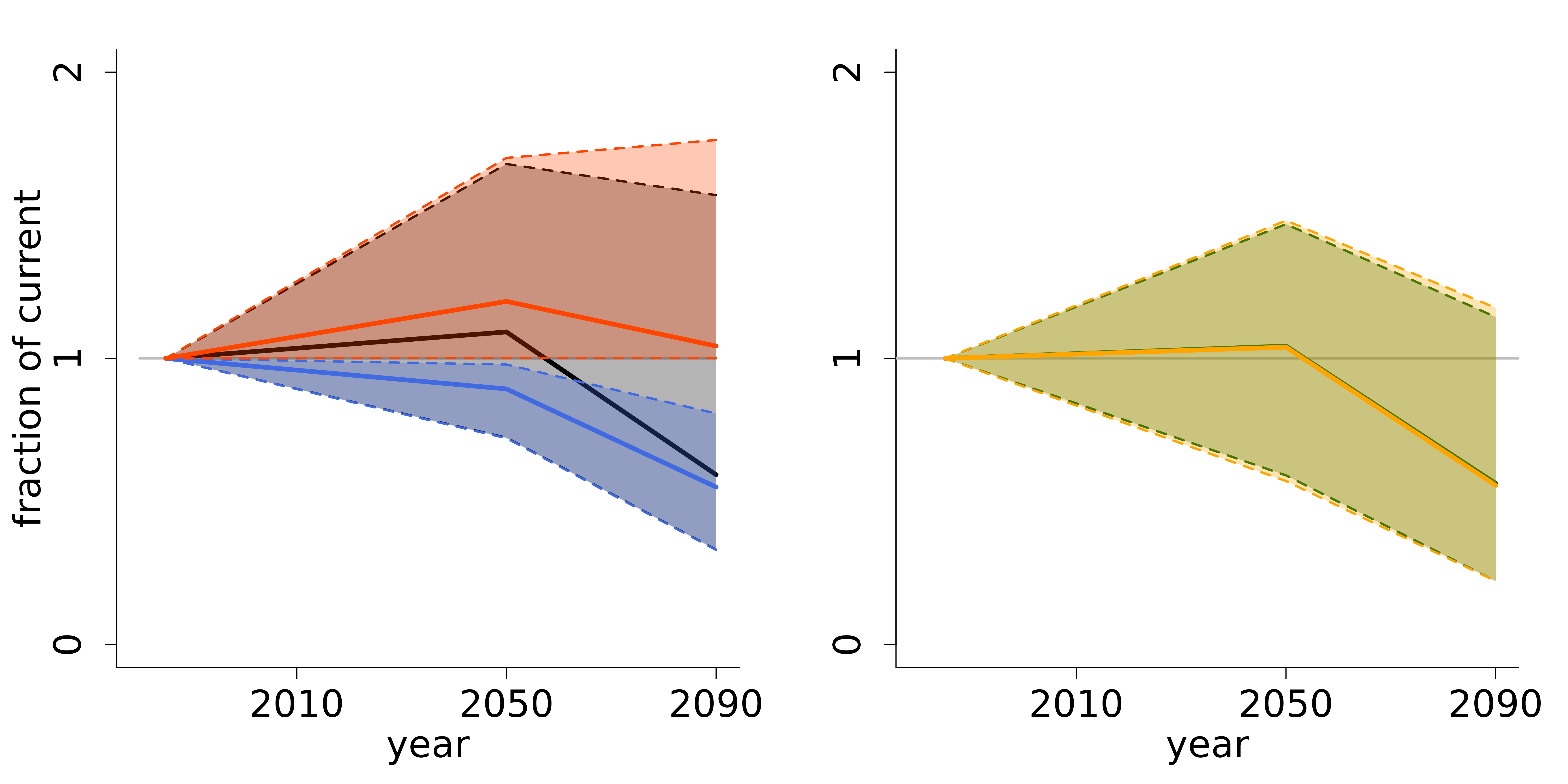

Supplement: S2 Appendix — (ZIP) [file pntd.0014030.s006.zip › Sup. Mat. 6-1 A-L - Species Trends/Atractaspis_fallax_CCTrends.png]

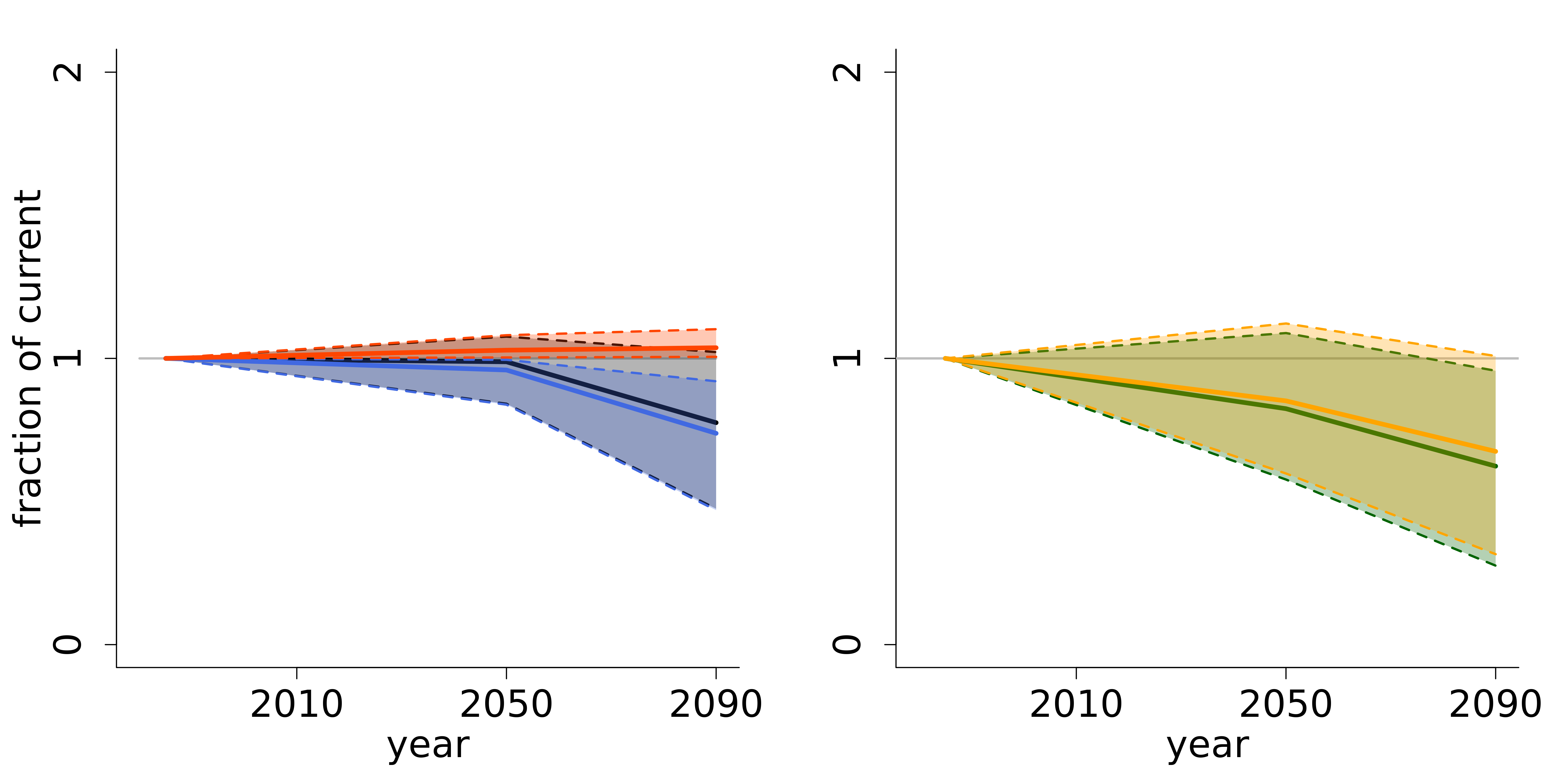

Supplement: S2 Appendix — (ZIP) [file pntd.0014030.s006.zip › Sup. Mat. 6-1 A-L - Species Trends/Atractaspis_irregularis_CCTrends.png]

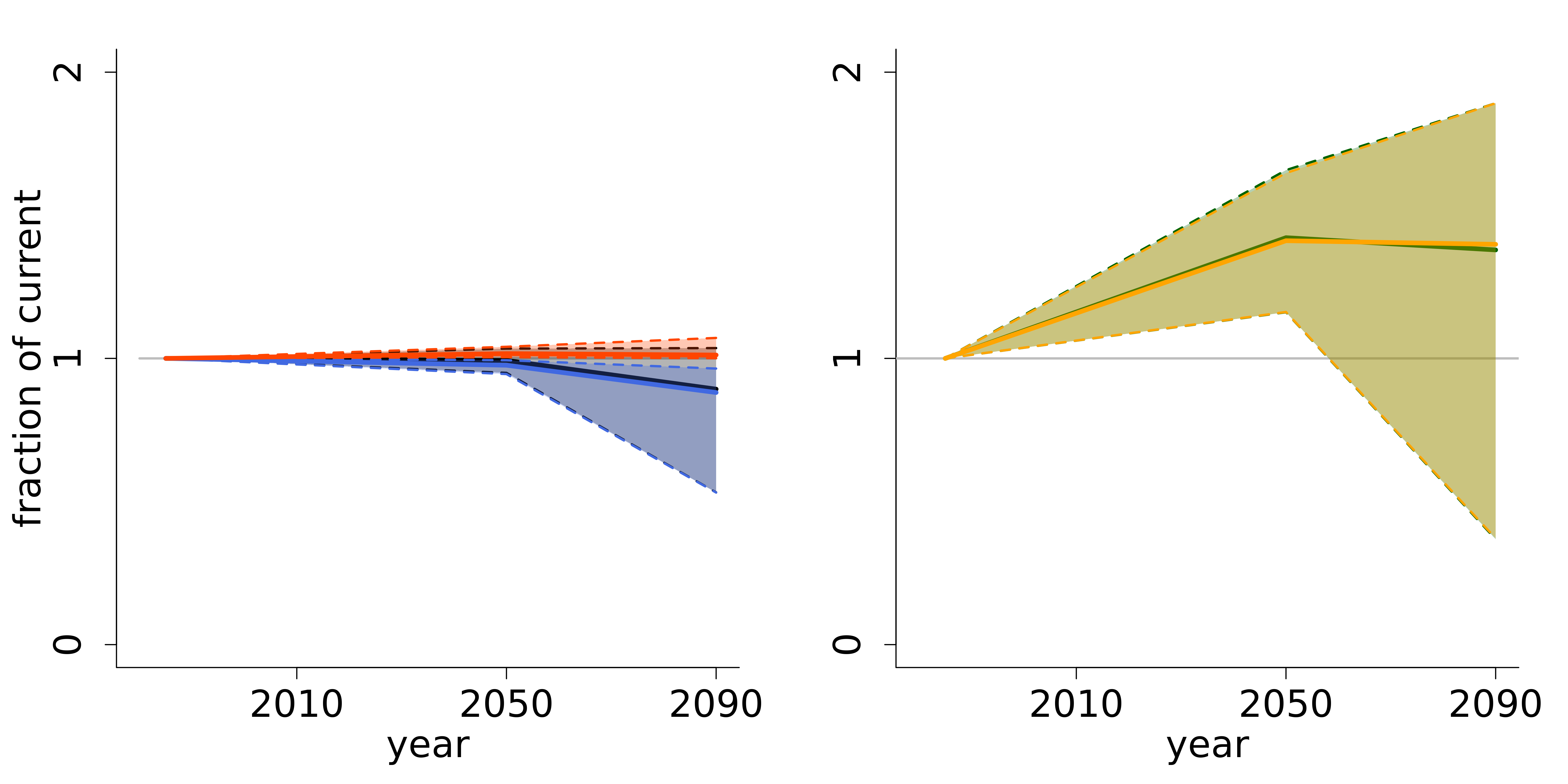

Supplement: S2 Appendix — (ZIP) [file pntd.0014030.s006.zip › Sup. Mat. 6-1 A-L - Species Trends/Atractaspis_magrettii_CCTrends.png]

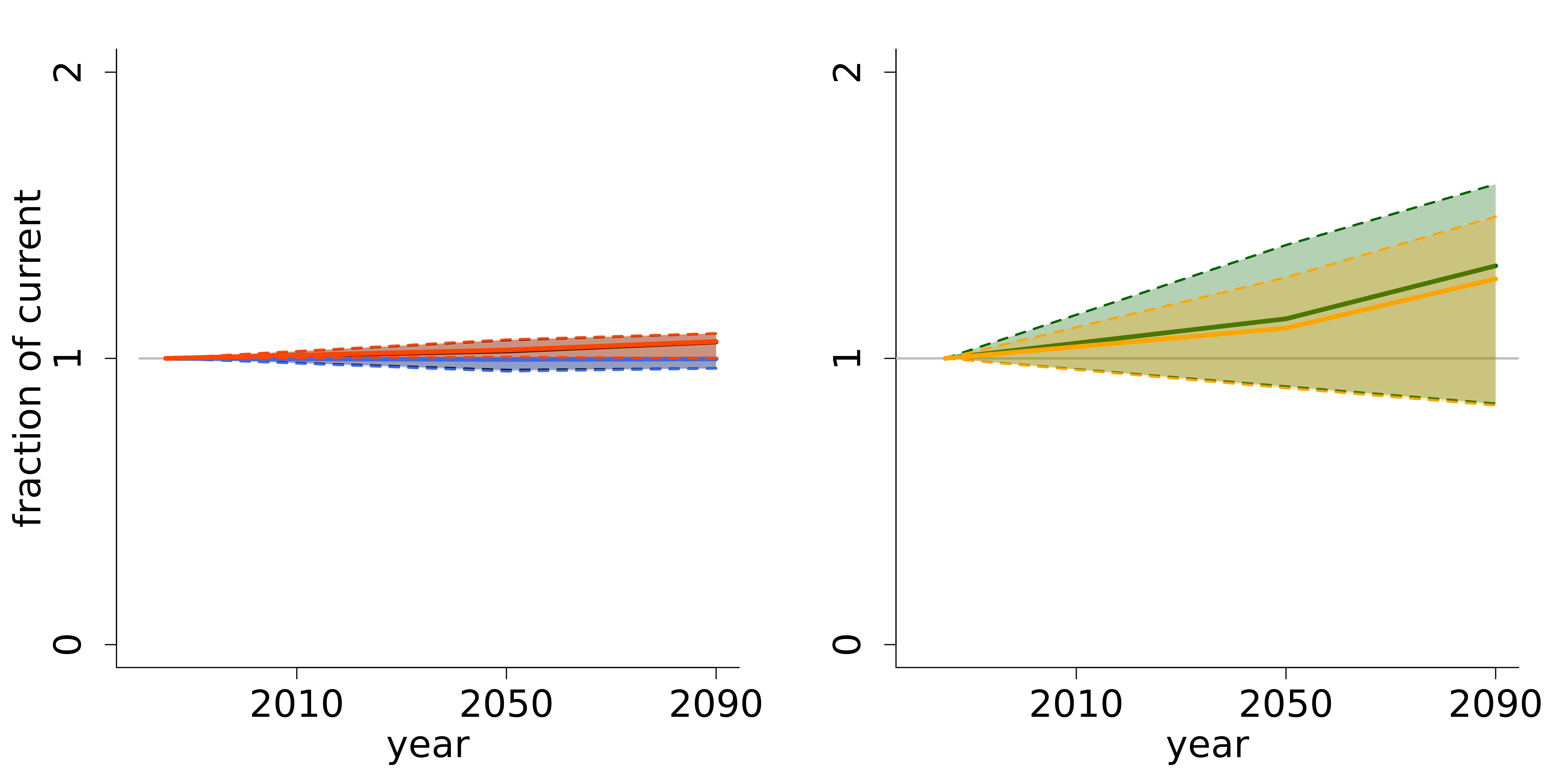

Supplement: S2 Appendix — (ZIP) [file pntd.0014030.s006.zip › Sup. Mat. 6-1 A-L - Species Trends/Atractaspis_microlepidota_CCTrends.png]

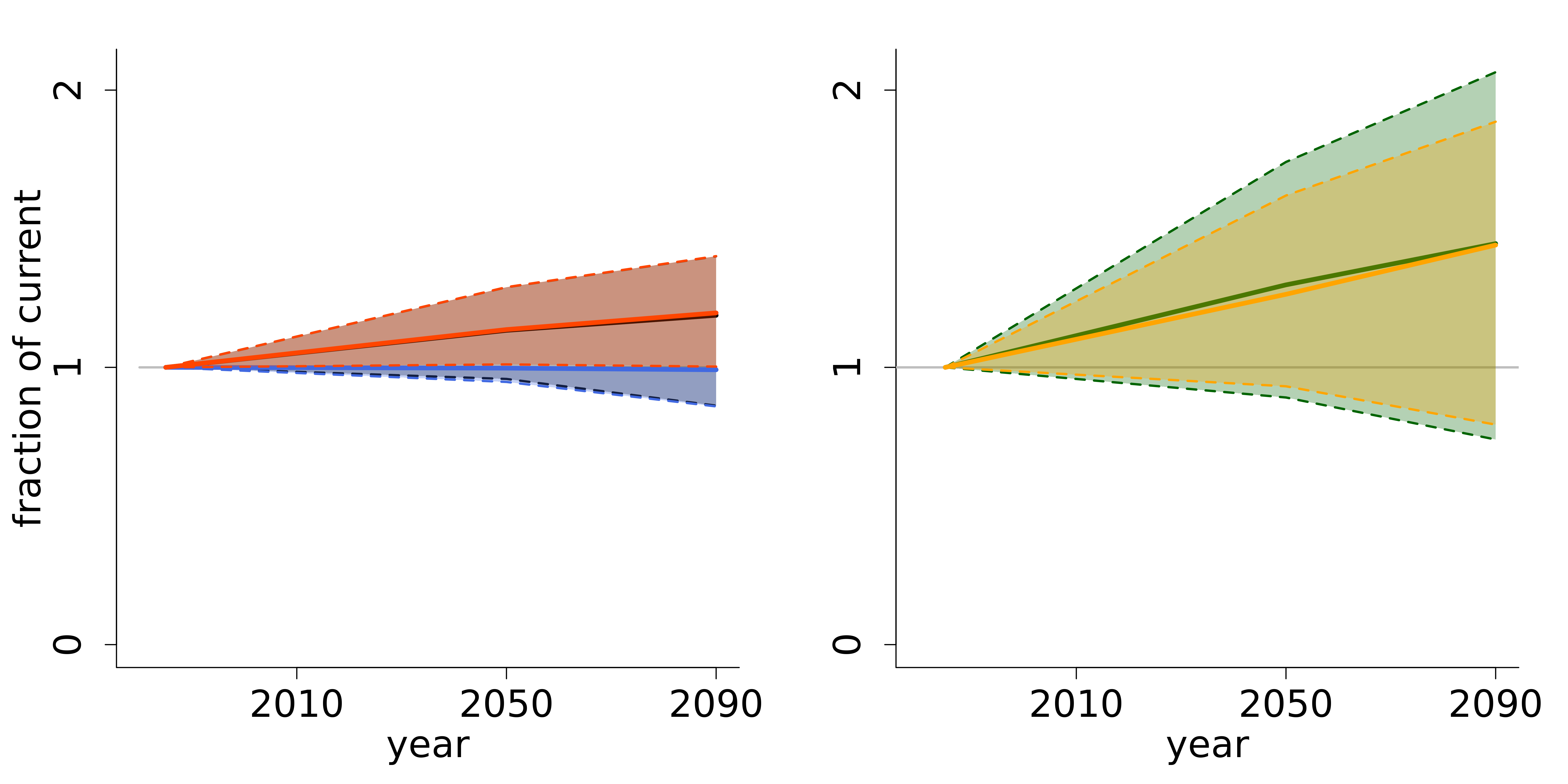

Supplement: S2 Appendix — (ZIP) [file pntd.0014030.s006.zip › Sup. Mat. 6-1 A-L - Species Trends/Atractaspis_micropholis_CCTrends.png]

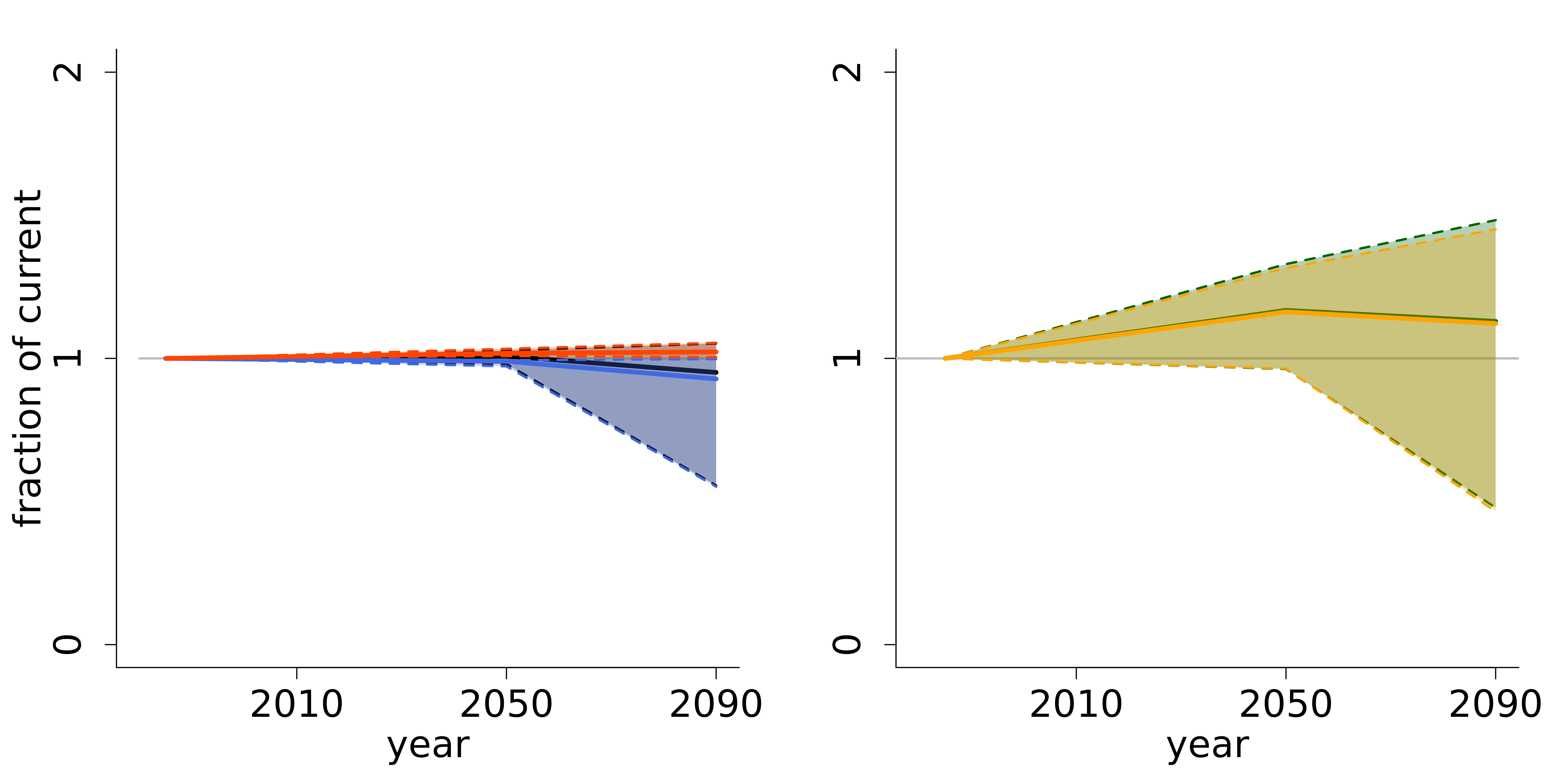

Supplement: S2 Appendix — (ZIP) [file pntd.0014030.s006.zip › Sup. Mat. 6-1 A-L - Species Trends/Atractaspis_phillipsi_CCTrends.png]

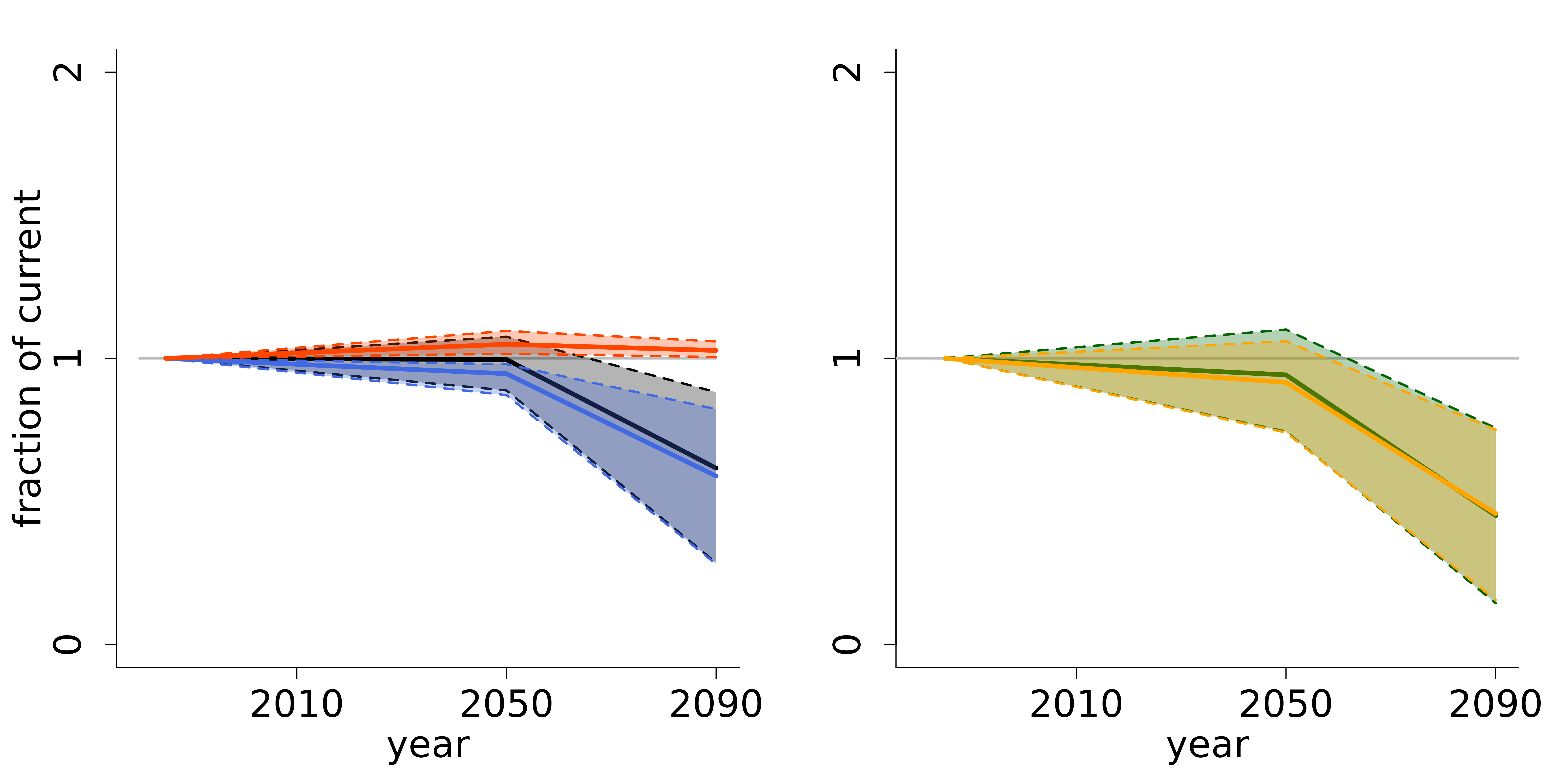

Supplement: S2 Appendix — (ZIP) [file pntd.0014030.s006.zip › Sup. Mat. 6-1 A-L - Species Trends/Atractaspis_watsoni_CCTrends.png]

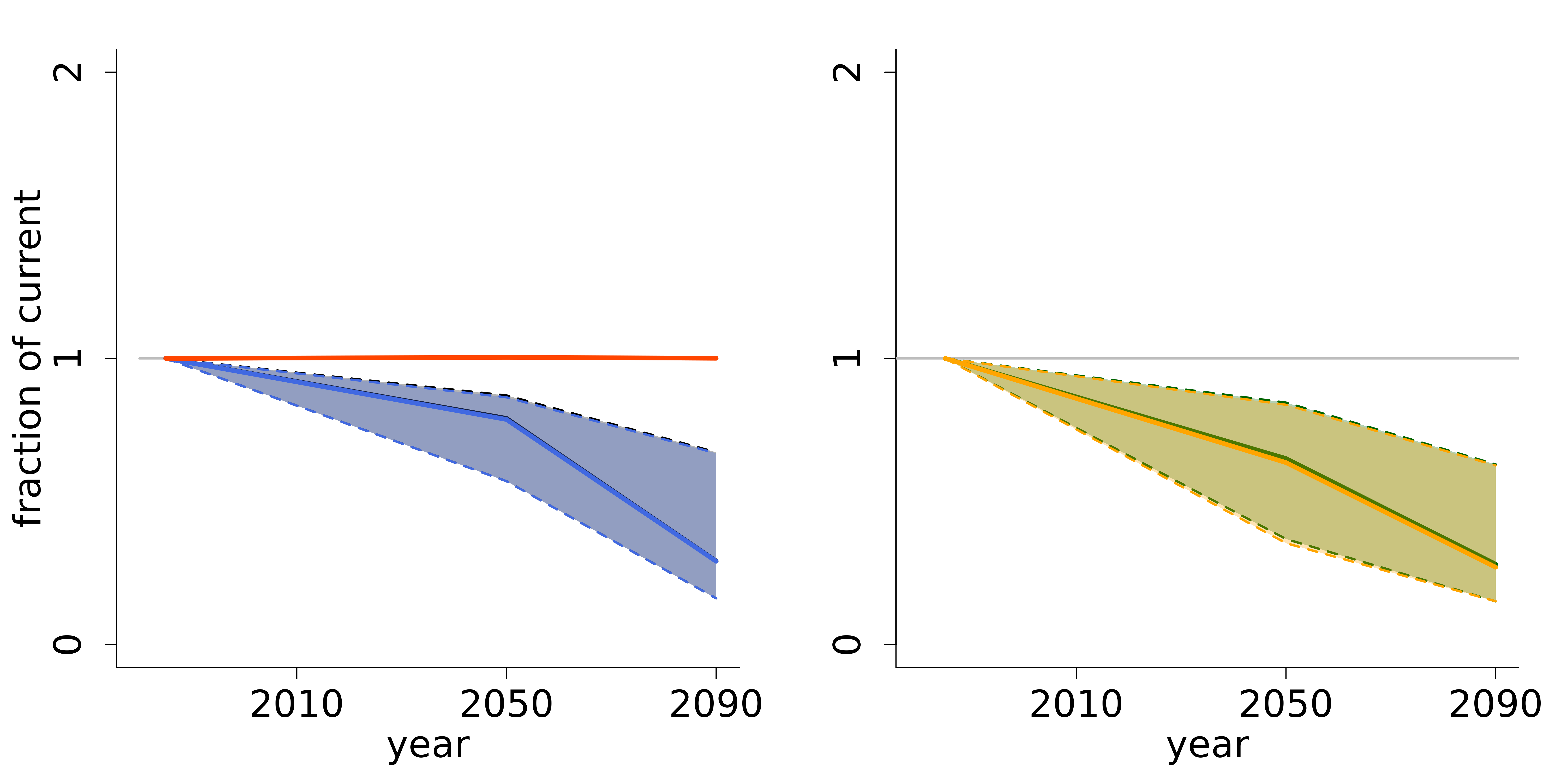

Supplement: S2 Appendix — (ZIP) [file pntd.0014030.s006.zip › Sup. Mat. 6-1 A-L - Species Trends/Atropoides_picadoi_CCTrends.png]

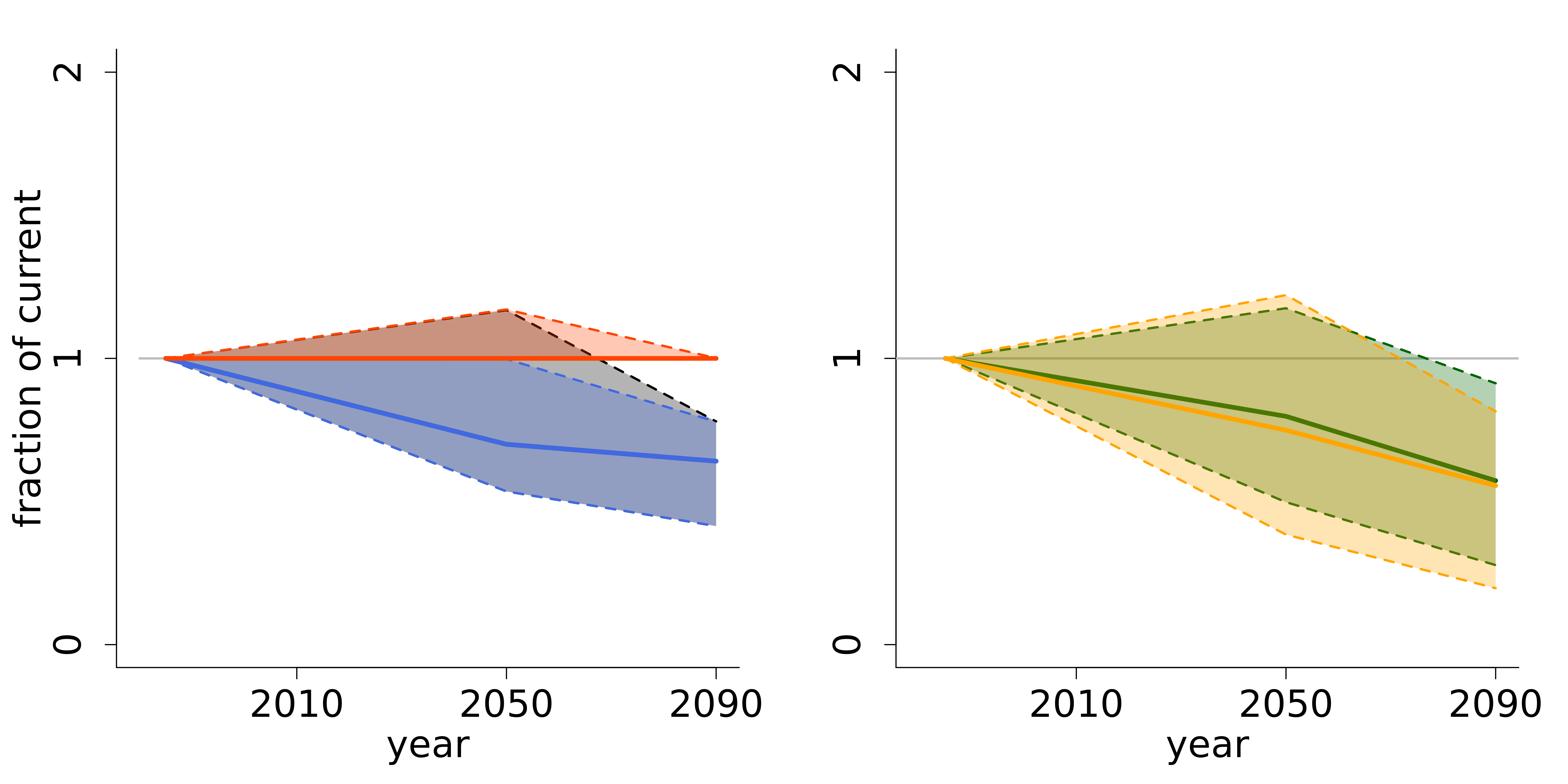

Supplement: S2 Appendix — (ZIP) [file pntd.0014030.s006.zip › Sup. Mat. 6-1 A-L - Species Trends/Austrelaps_labialis_CCTrends.png]

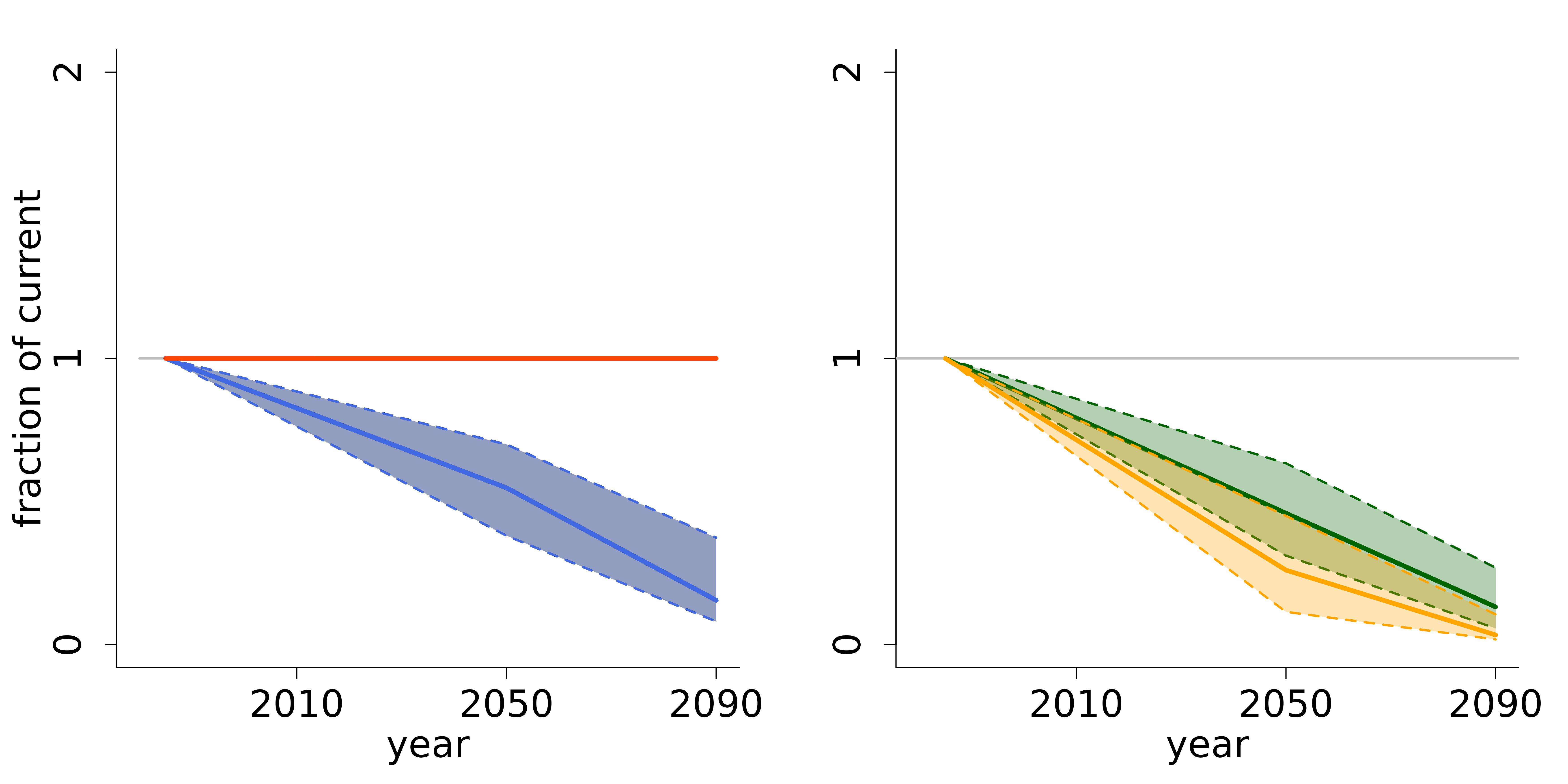

Supplement: S2 Appendix — (ZIP) [file pntd.0014030.s006.zip › Sup. Mat. 6-1 A-L - Species Trends/Austrelaps_ramsayi_CCTrends.png]

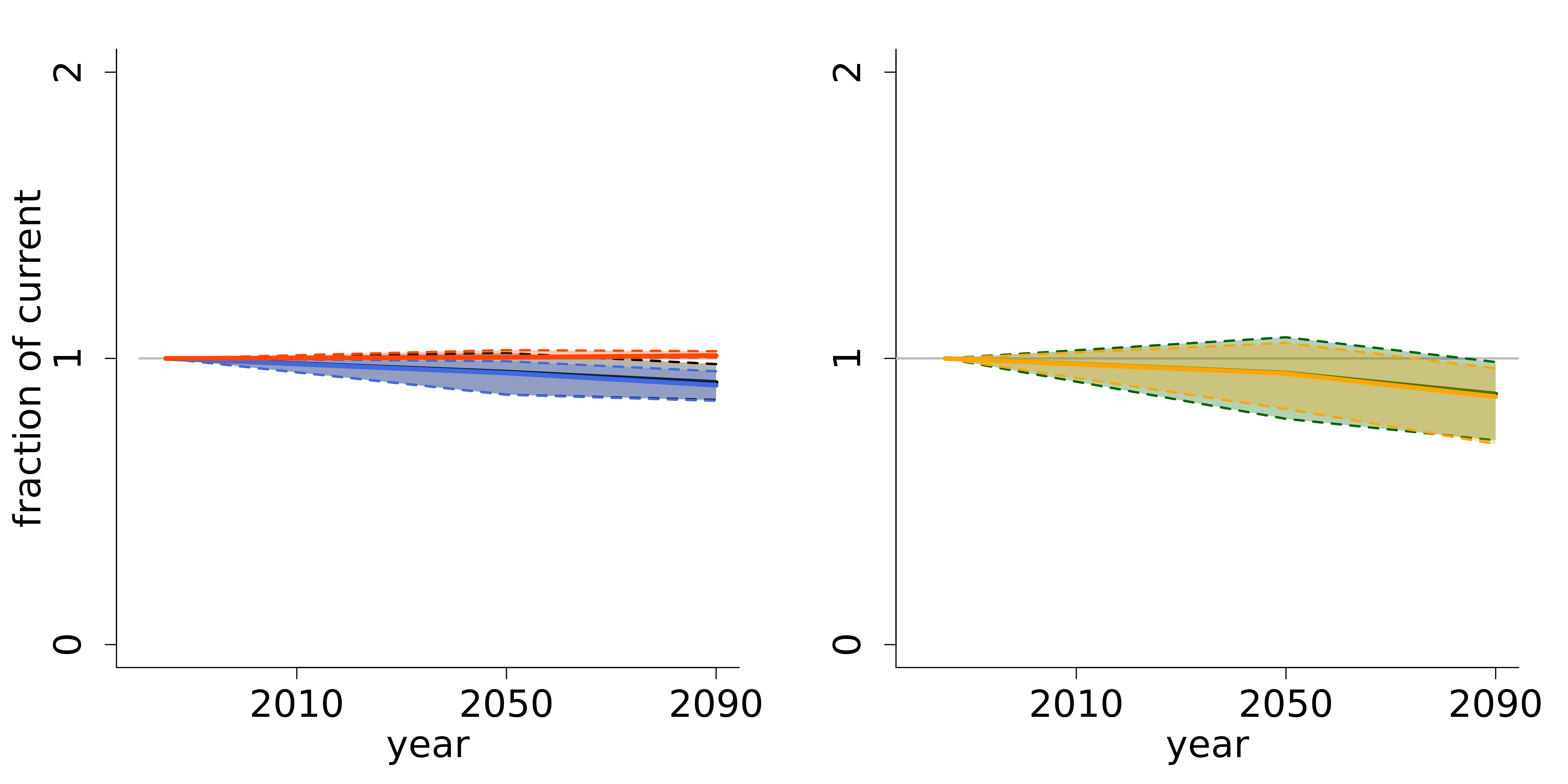

Supplement: S2 Appendix — (ZIP) [file pntd.0014030.s006.zip › Sup. Mat. 6-1 A-L - Species Trends/Austrelaps_superbus_CCTrends.png]

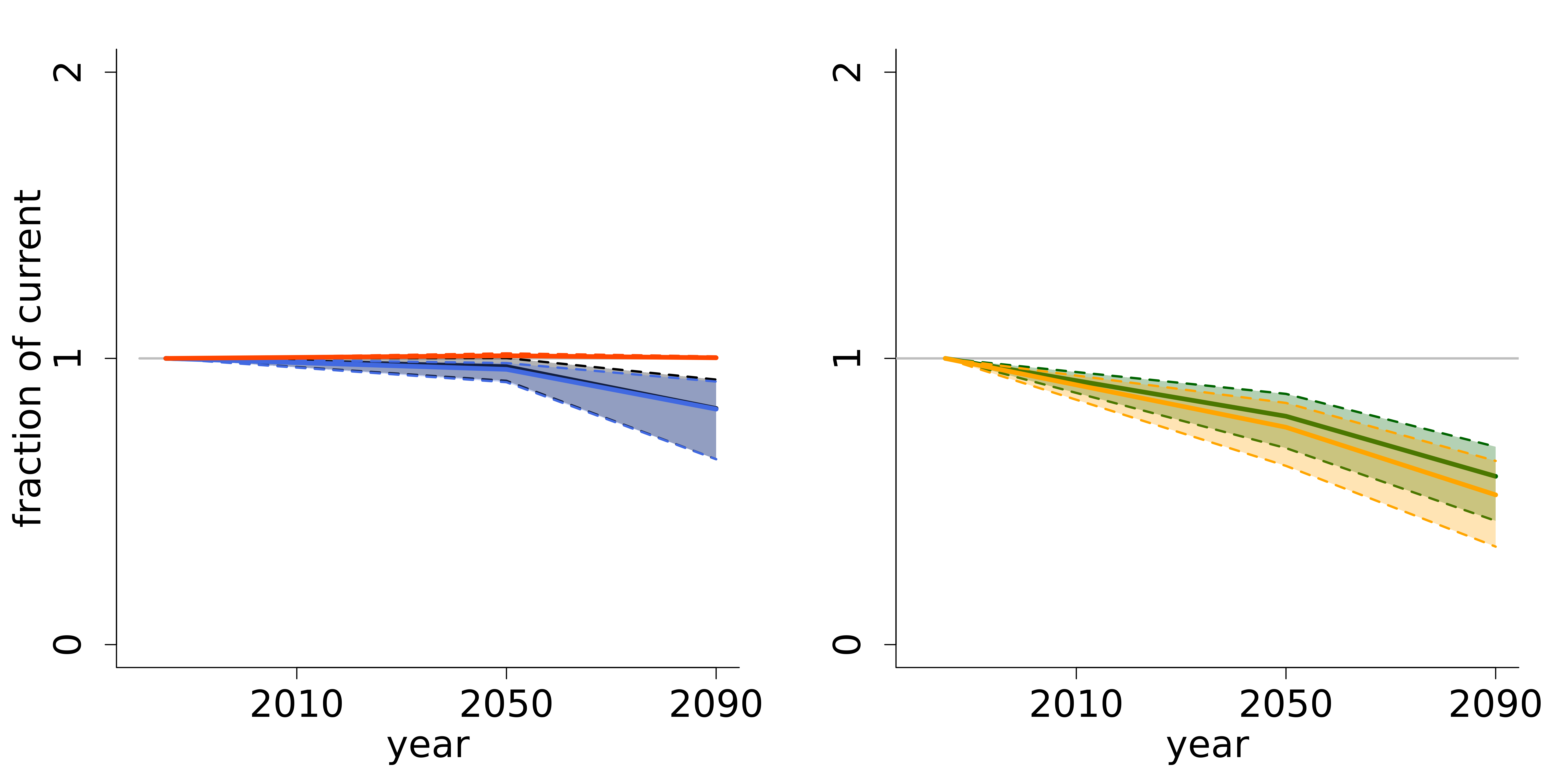

Supplement: S2 Appendix — (ZIP) [file pntd.0014030.s006.zip › Sup. Mat. 6-1 A-L - Species Trends/Bitis_arietans_CCTrends.png]

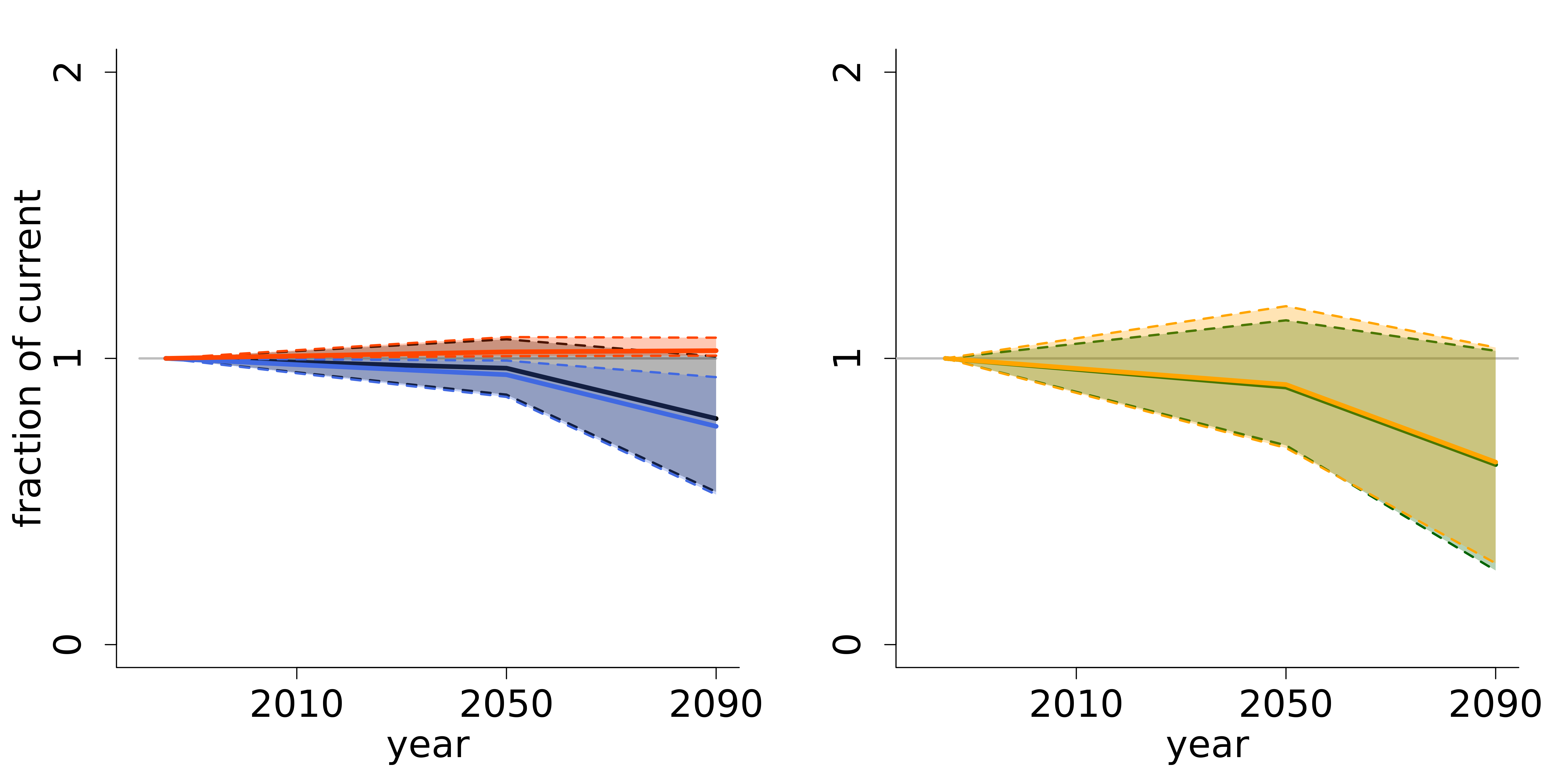

Supplement: S2 Appendix — (ZIP) [file pntd.0014030.s006.zip › Sup. Mat. 6-1 A-L - Species Trends/Bitis_gabonica_CCTrends.png]

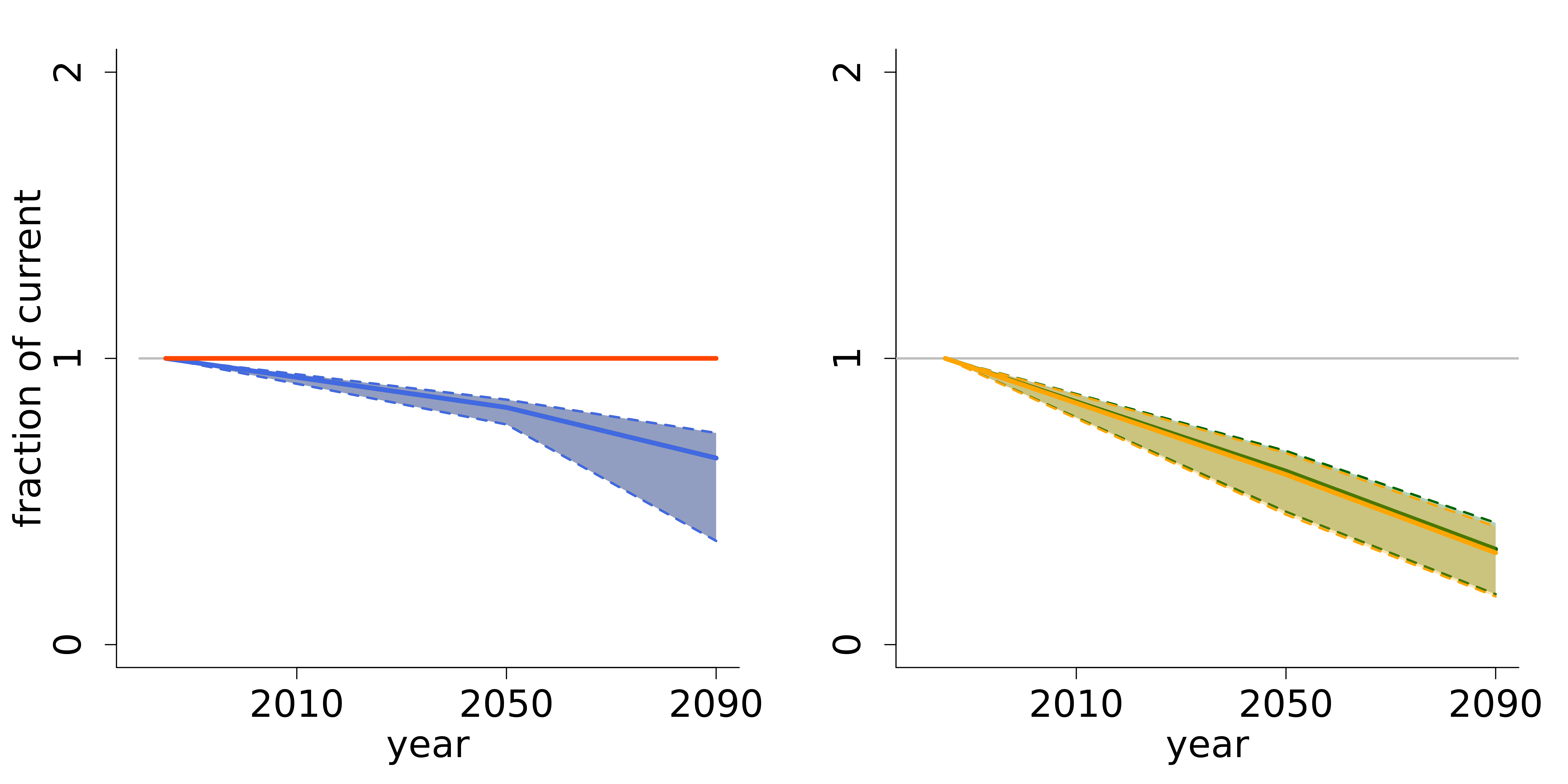

Supplement: S2 Appendix — (ZIP) [file pntd.0014030.s006.zip › Sup. Mat. 6-1 A-L - Species Trends/Bitis_harenna_CCTrends.png]

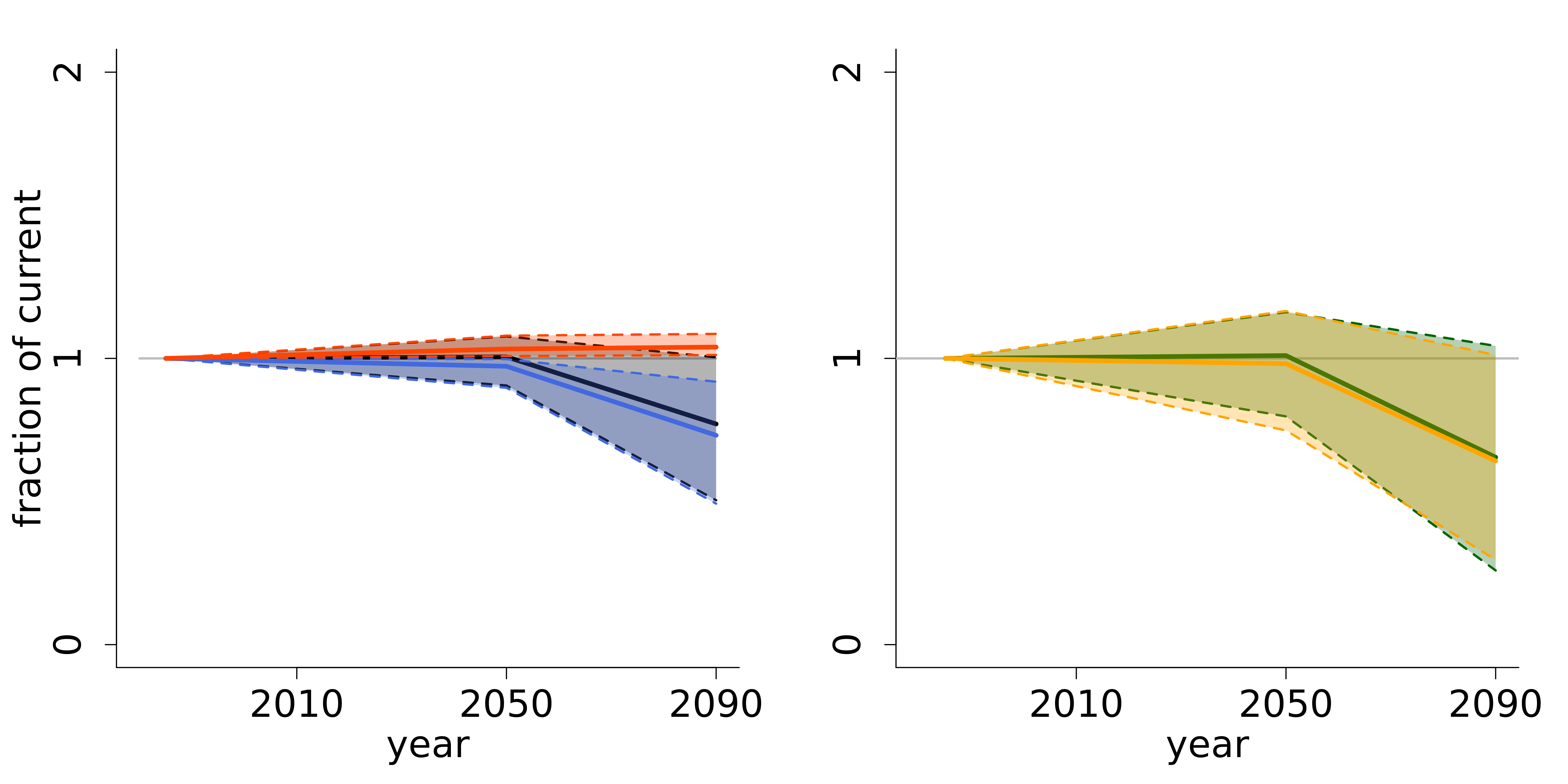

Supplement: S2 Appendix — (ZIP) [file pntd.0014030.s006.zip › Sup. Mat. 6-1 A-L - Species Trends/Bitis_nasicornis_CCTrends.png]

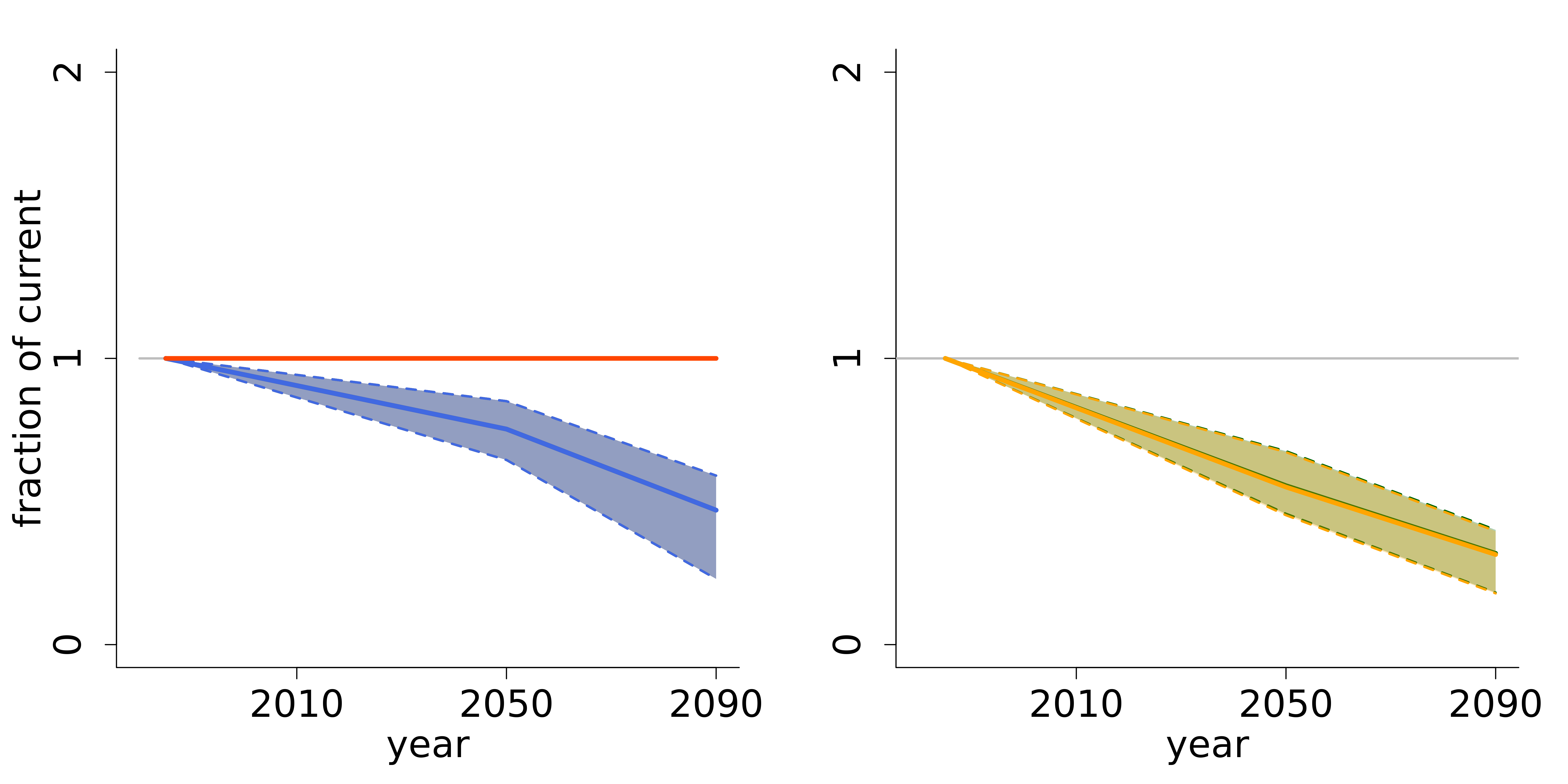

Supplement: S2 Appendix — (ZIP) [file pntd.0014030.s006.zip › Sup. Mat. 6-1 A-L - Species Trends/Bitis_parviocula_CCTrends.png]

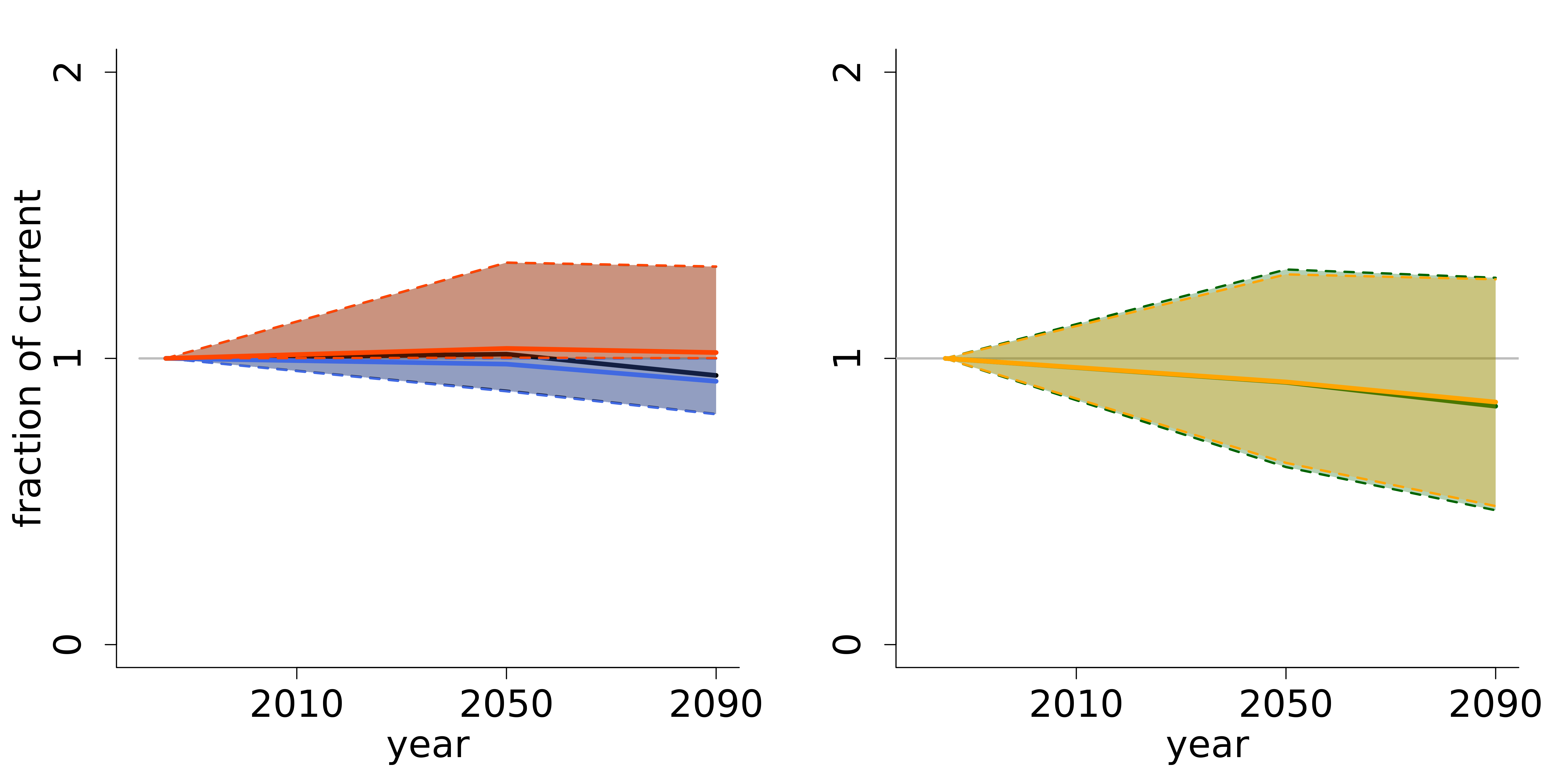

Supplement: S2 Appendix — (ZIP) [file pntd.0014030.s006.zip › Sup. Mat. 6-1 A-L - Species Trends/Bitis_rhinoceros_CCTrends.png]

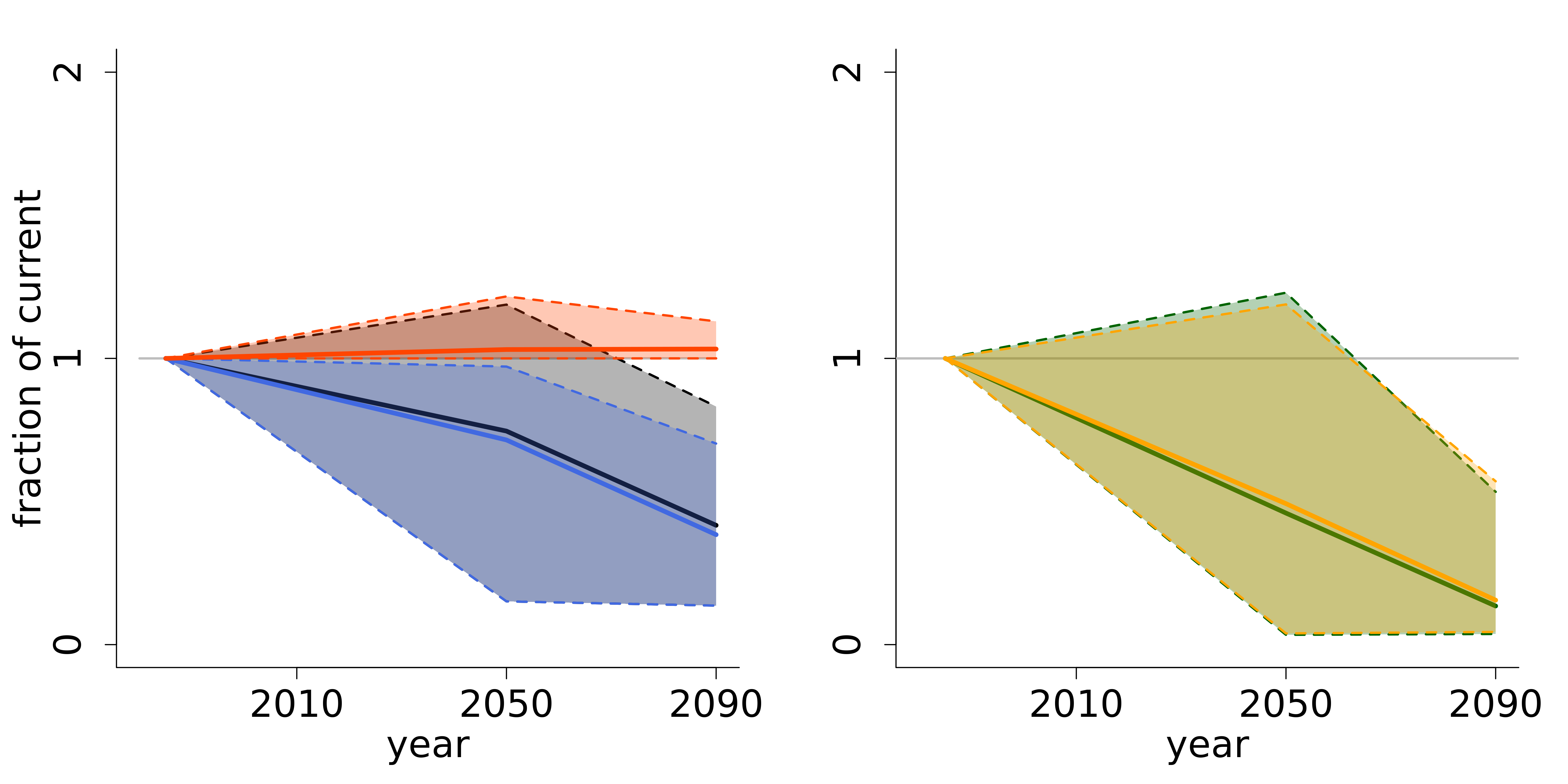

Supplement: S2 Appendix — (ZIP) [file pntd.0014030.s006.zip › Sup. Mat. 6-1 A-L - Species Trends/Bothriechis_aurifer_CCTrends.png]

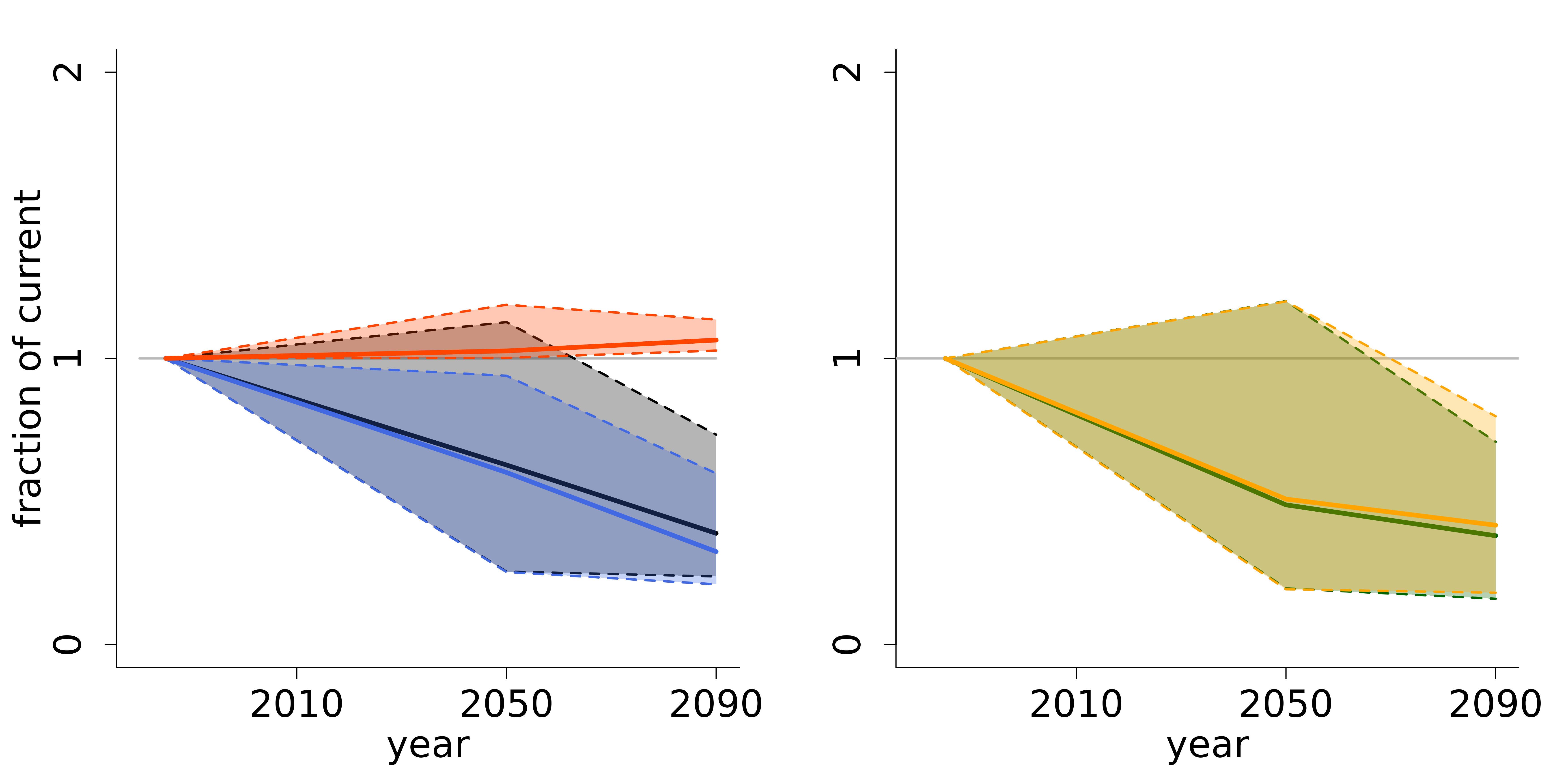

Supplement: S2 Appendix — (ZIP) [file pntd.0014030.s006.zip › Sup. Mat. 6-1 A-L - Species Trends/Bothriechis_bicolor_CCTrends.png]

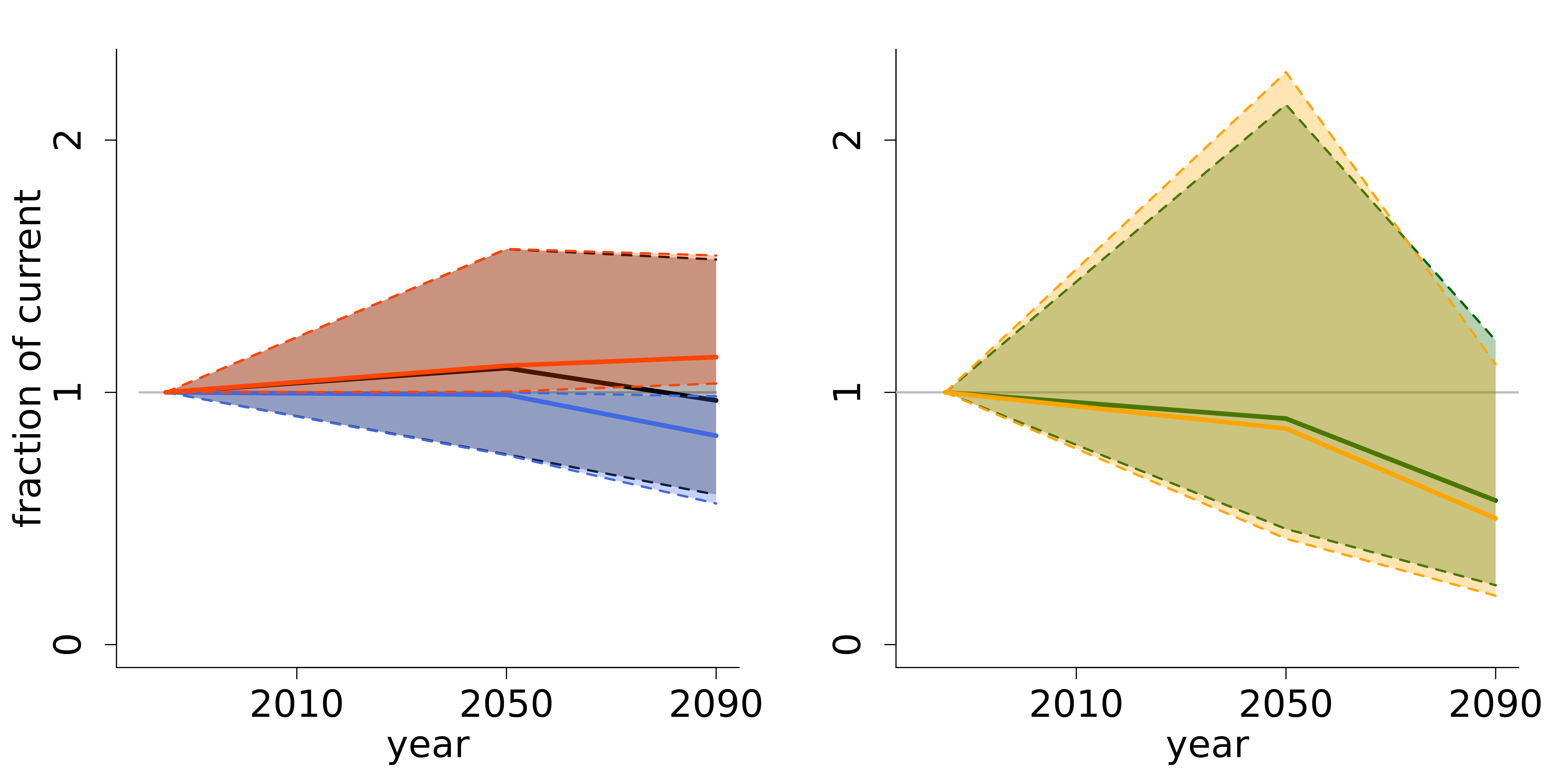

Supplement: S2 Appendix — (ZIP) [file pntd.0014030.s006.zip › Sup. Mat. 6-1 A-L - Species Trends/Bothriechis_guifarroi_CCTrends.png]

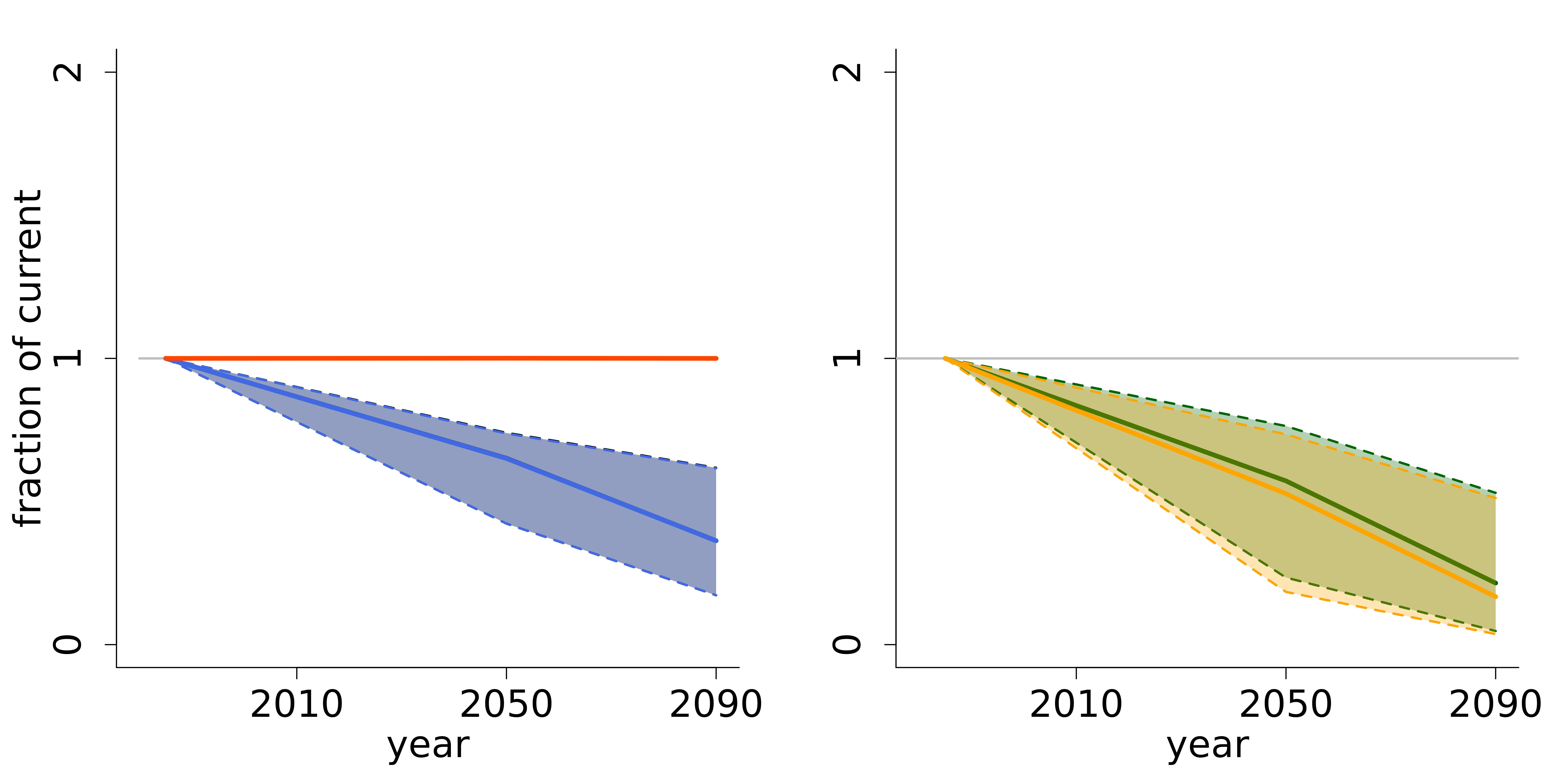

Supplement: S2 Appendix — (ZIP) [file pntd.0014030.s006.zip › Sup. Mat. 6-1 A-L - Species Trends/Bothriechis_lateralis_CCTrends.png]

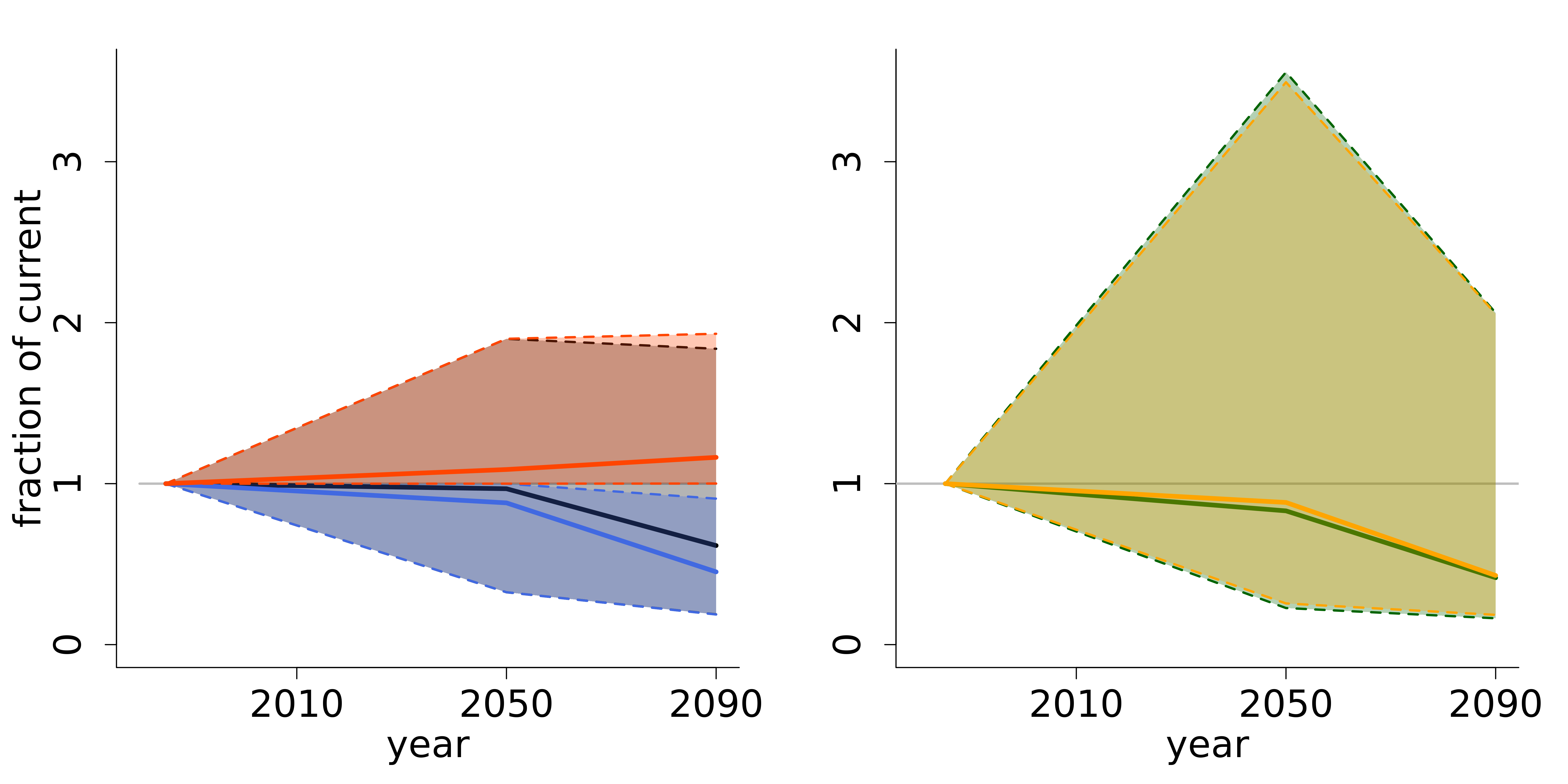

Supplement: S2 Appendix — (ZIP) [file pntd.0014030.s006.zip › Sup. Mat. 6-1 A-L - Species Trends/Bothriechis_marchi_CCTrends.png]

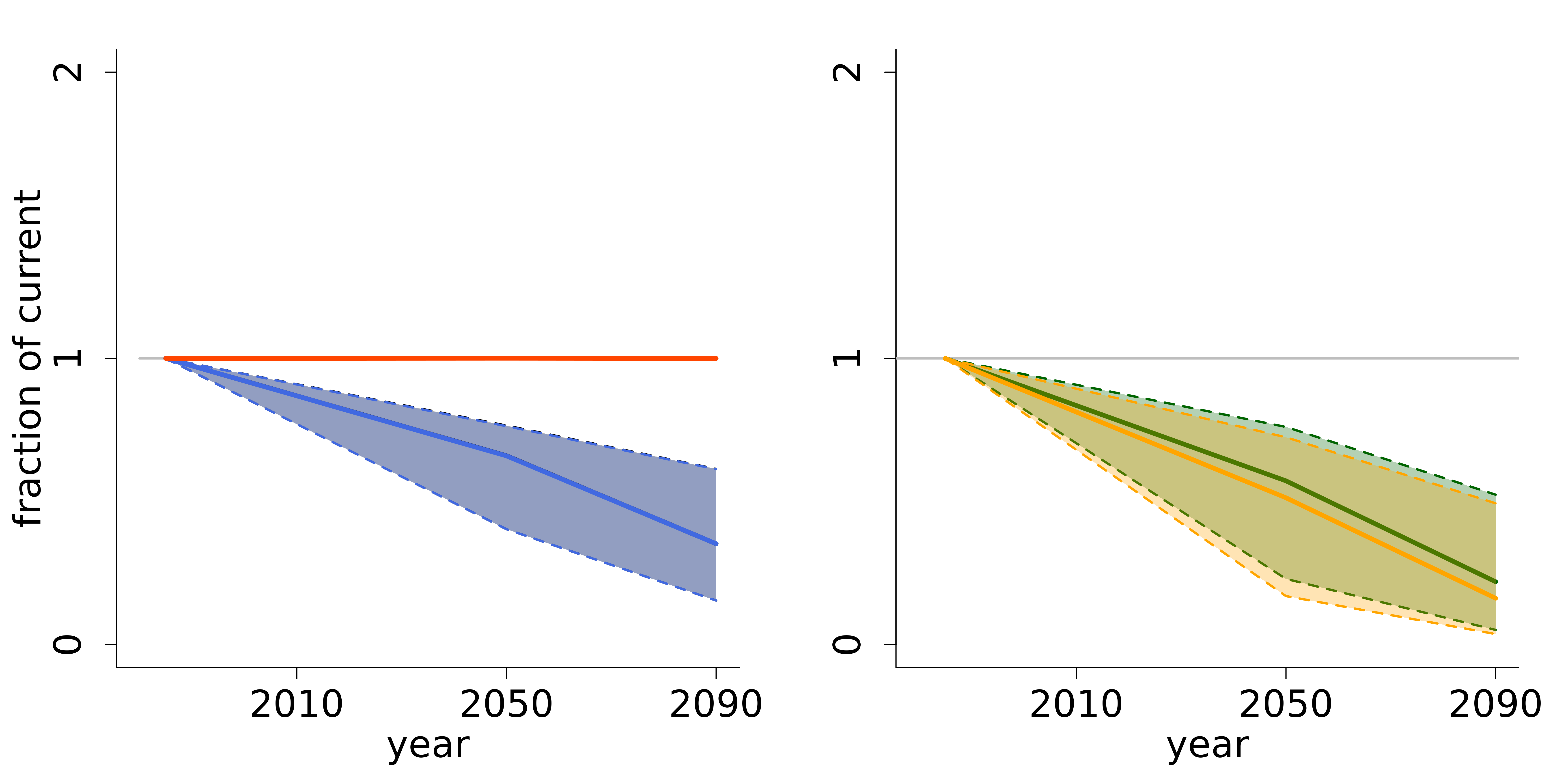

Supplement: S2 Appendix — (ZIP) [file pntd.0014030.s006.zip › Sup. Mat. 6-1 A-L - Species Trends/Bothriechis_nigroviridis_CCTrends.png]

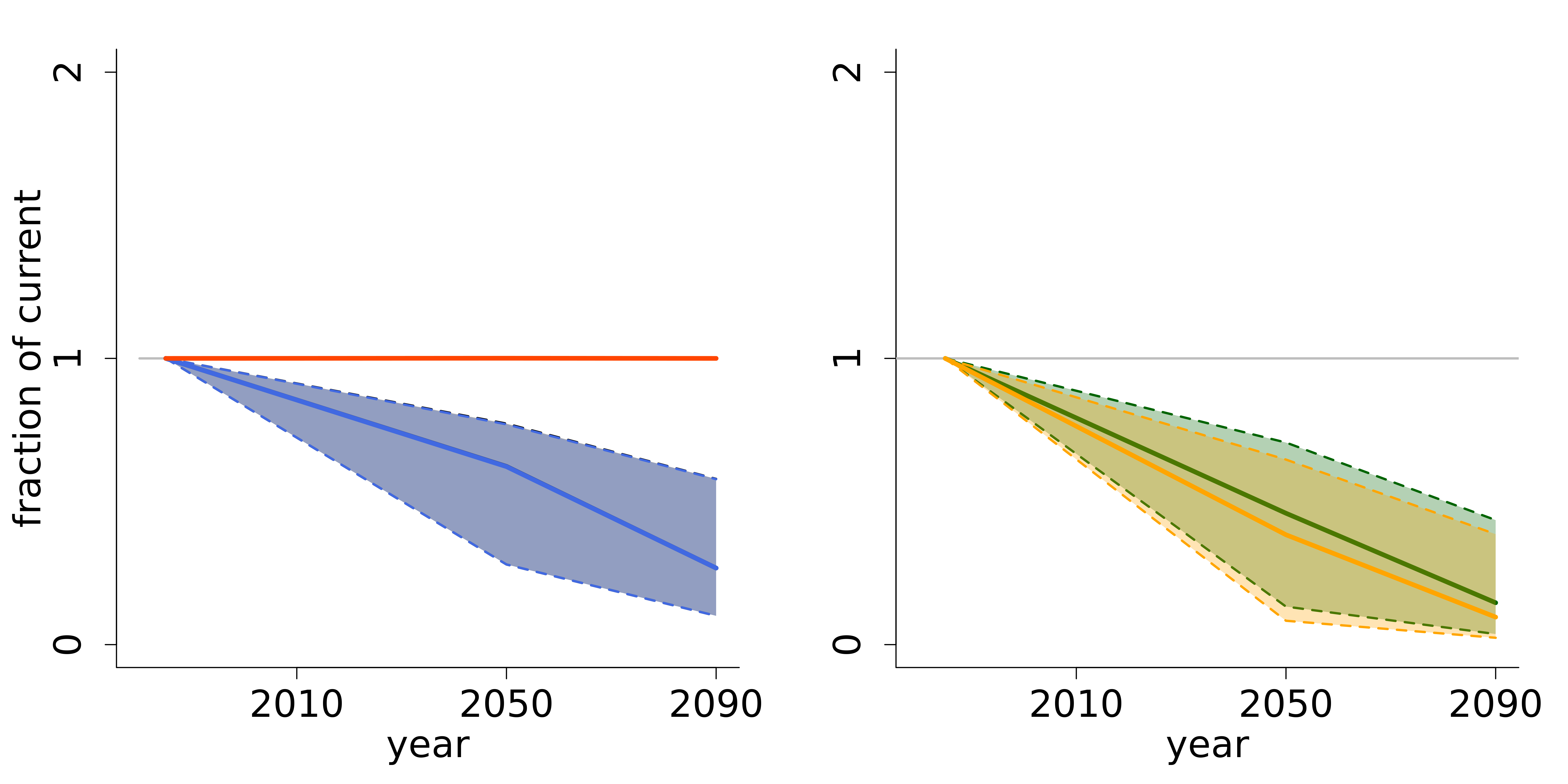

Supplement: S2 Appendix — (ZIP) [file pntd.0014030.s006.zip › Sup. Mat. 6-1 A-L - Species Trends/Bothriechis_nubestris_CCTrends.png]

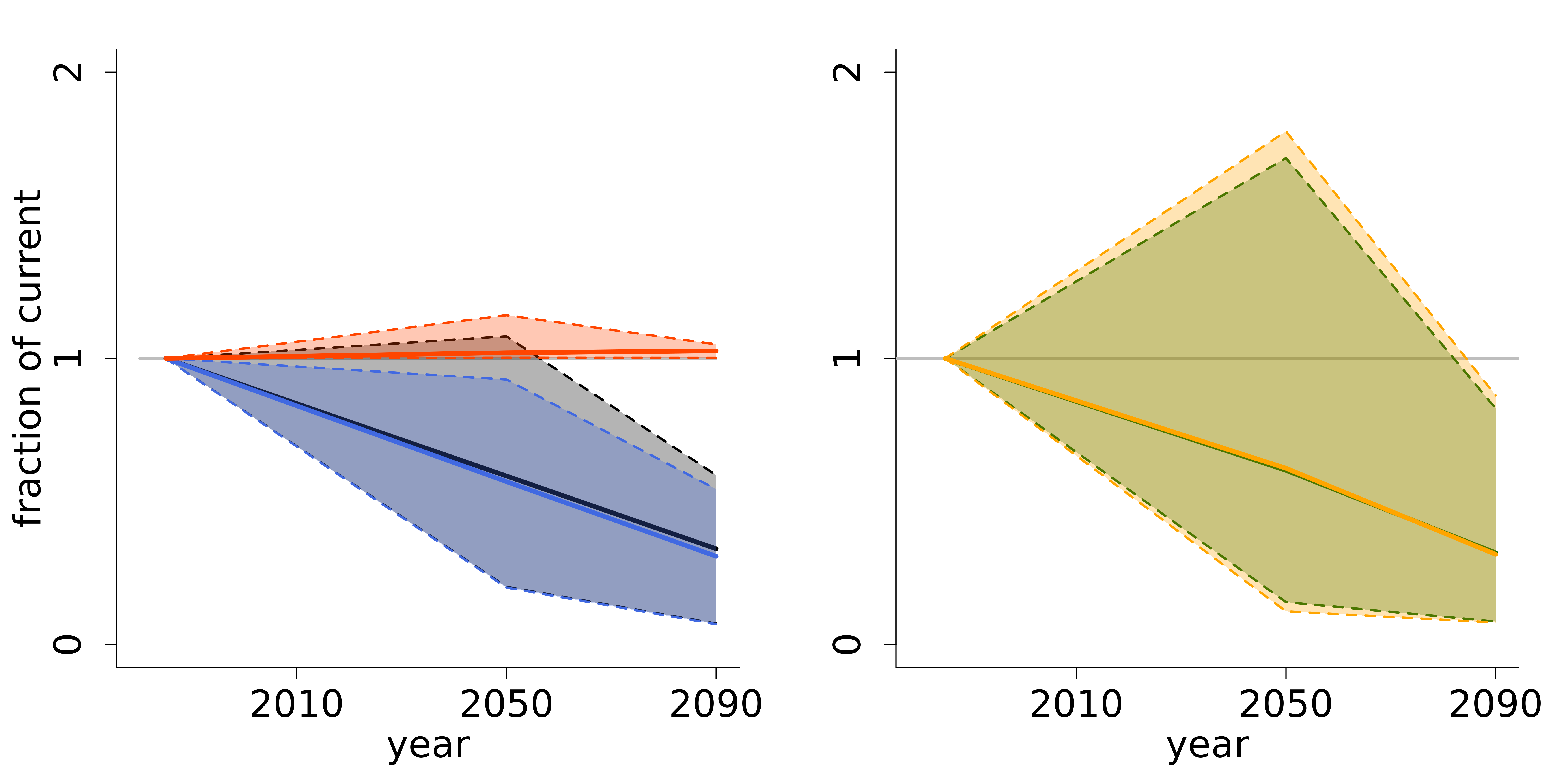

Supplement: S2 Appendix — (ZIP) [file pntd.0014030.s006.zip › Sup. Mat. 6-1 A-L - Species Trends/Bothriechis_rowleyi_CCTrends.png]

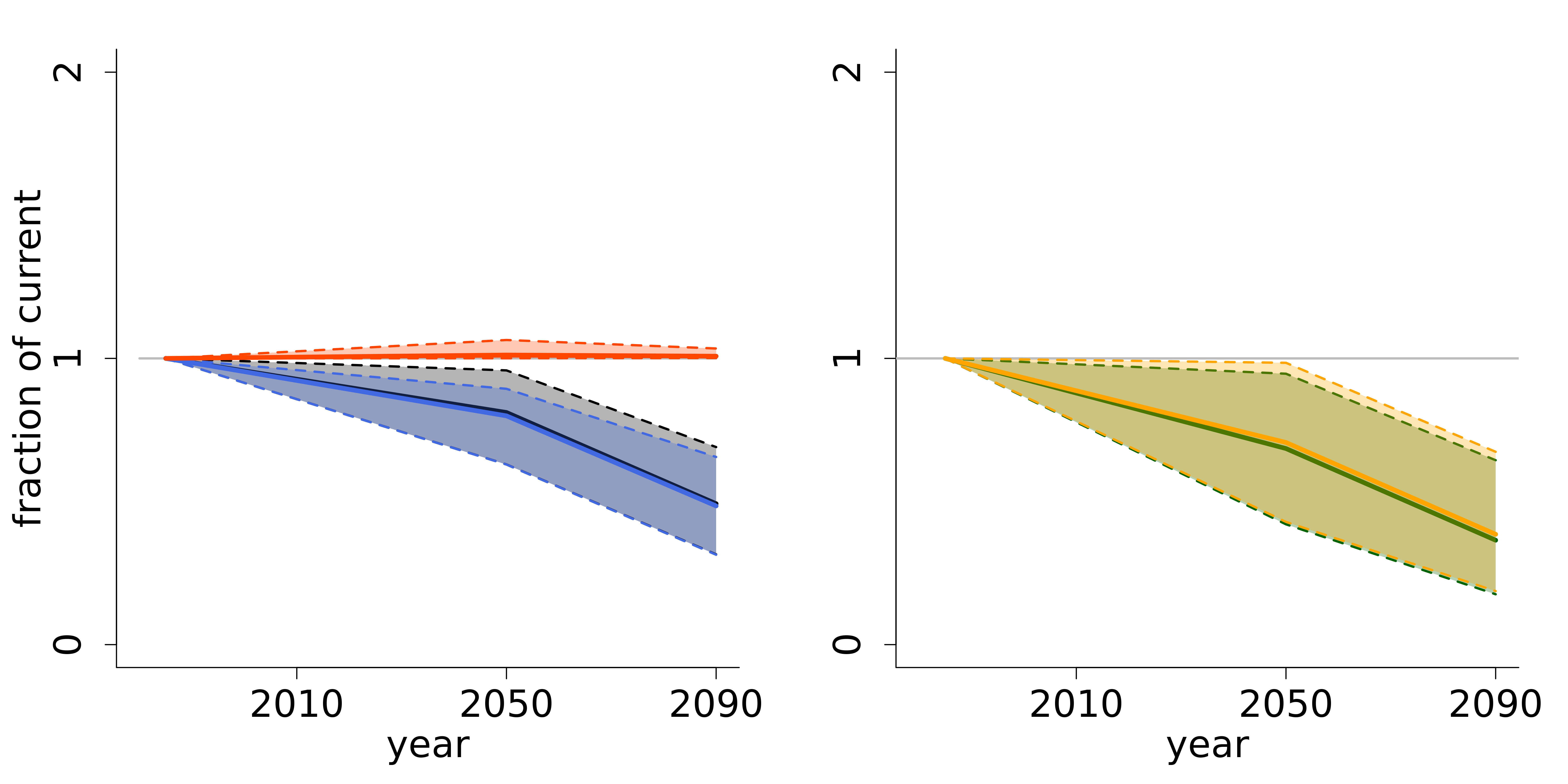

Supplement: S2 Appendix — (ZIP) [file pntd.0014030.s006.zip › Sup. Mat. 6-1 A-L - Species Trends/Bothriechis_schlegelii_CCTrends.png]

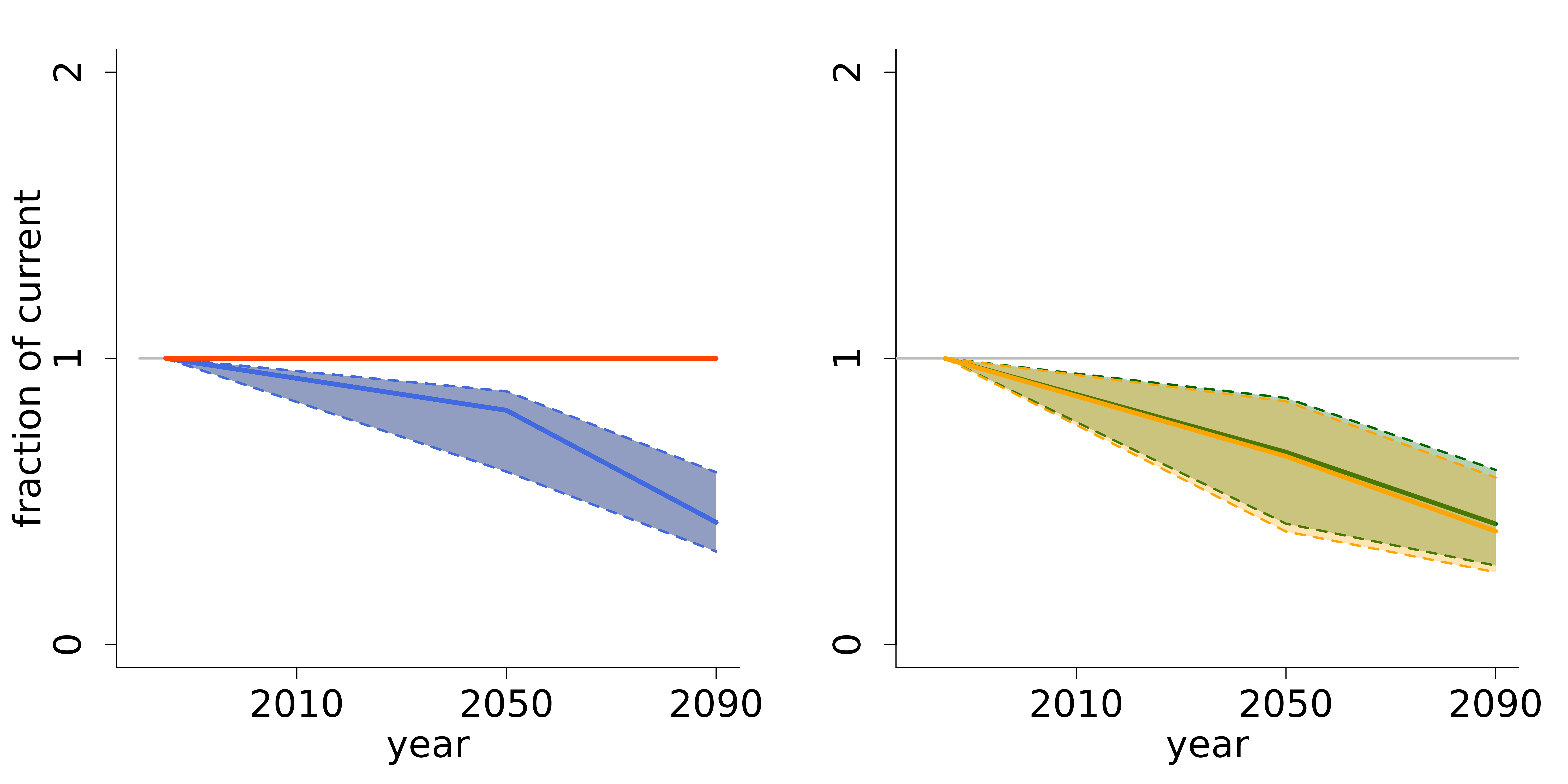

Supplement: S2 Appendix — (ZIP) [file pntd.0014030.s006.zip › Sup. Mat. 6-1 A-L - Species Trends/Bothriechis_supraciliaris_CCTrends.png]

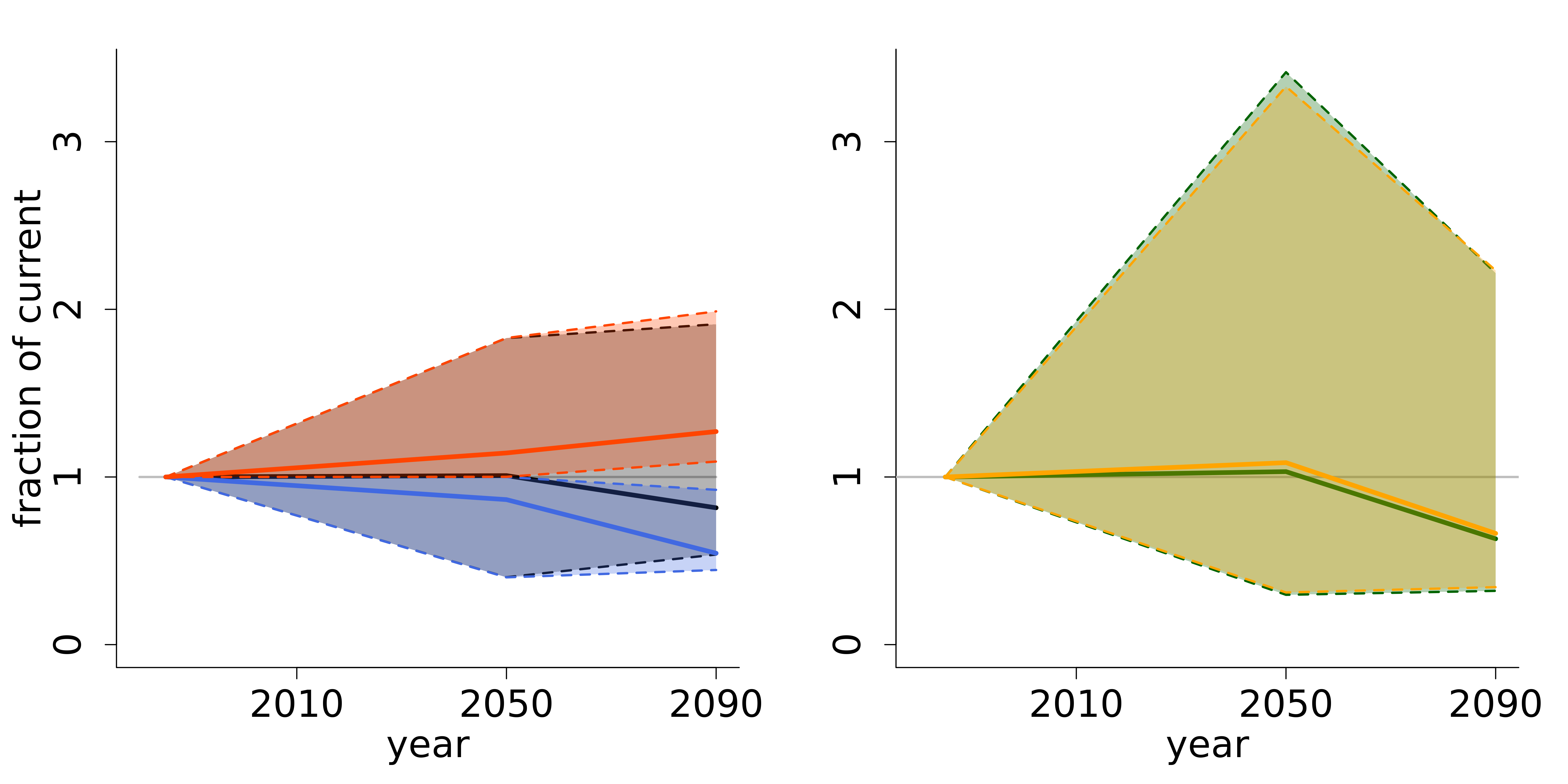

Supplement: S2 Appendix — (ZIP) [file pntd.0014030.s006.zip › Sup. Mat. 6-1 A-L - Species Trends/Bothriechis_thalassinus_CCTrends.png]

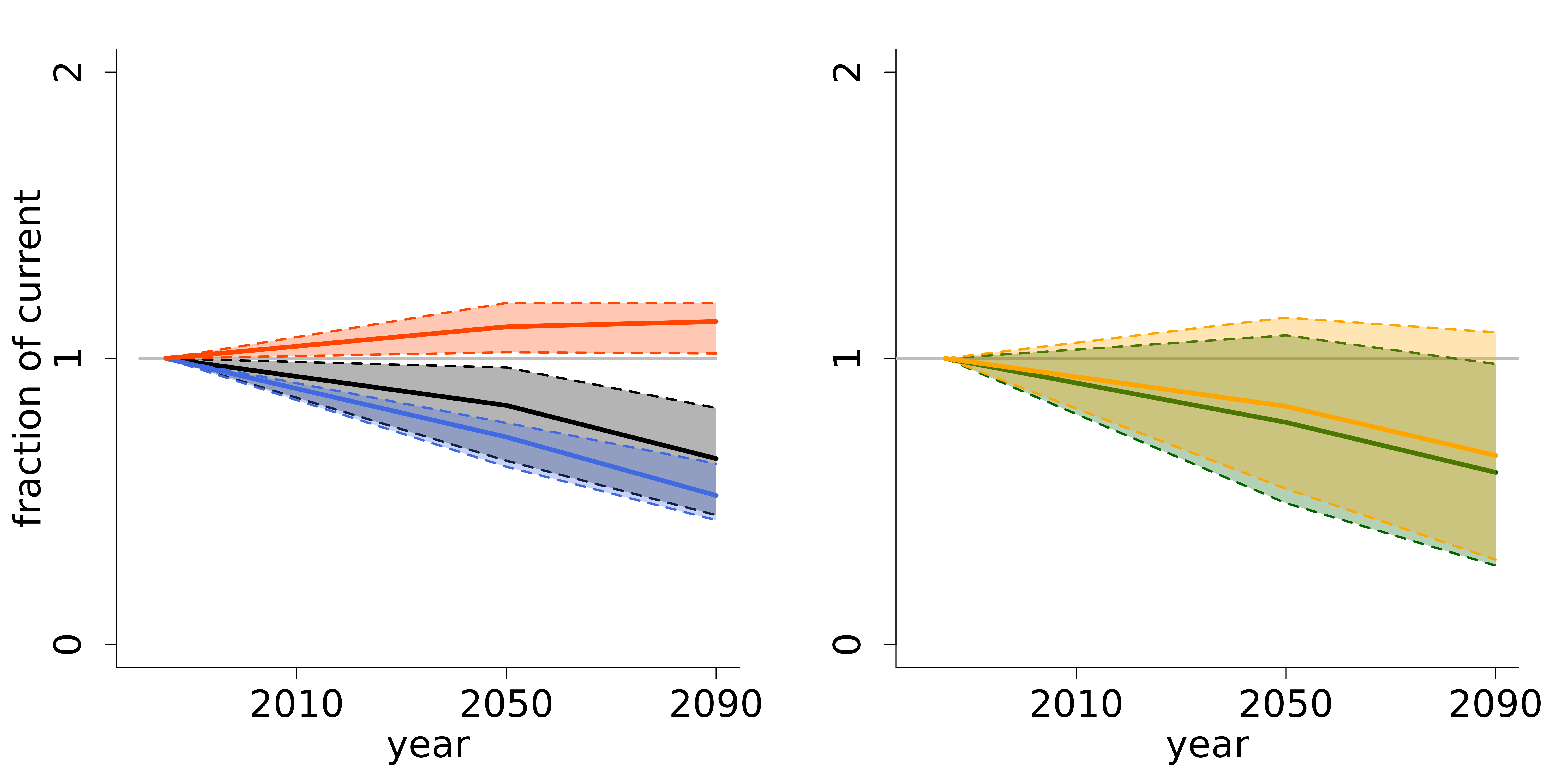

Supplement: S2 Appendix — (ZIP) [file pntd.0014030.s006.zip › Sup. Mat. 6-1 A-L - Species Trends/Bothrocophias_andianus_CCTrends.png]

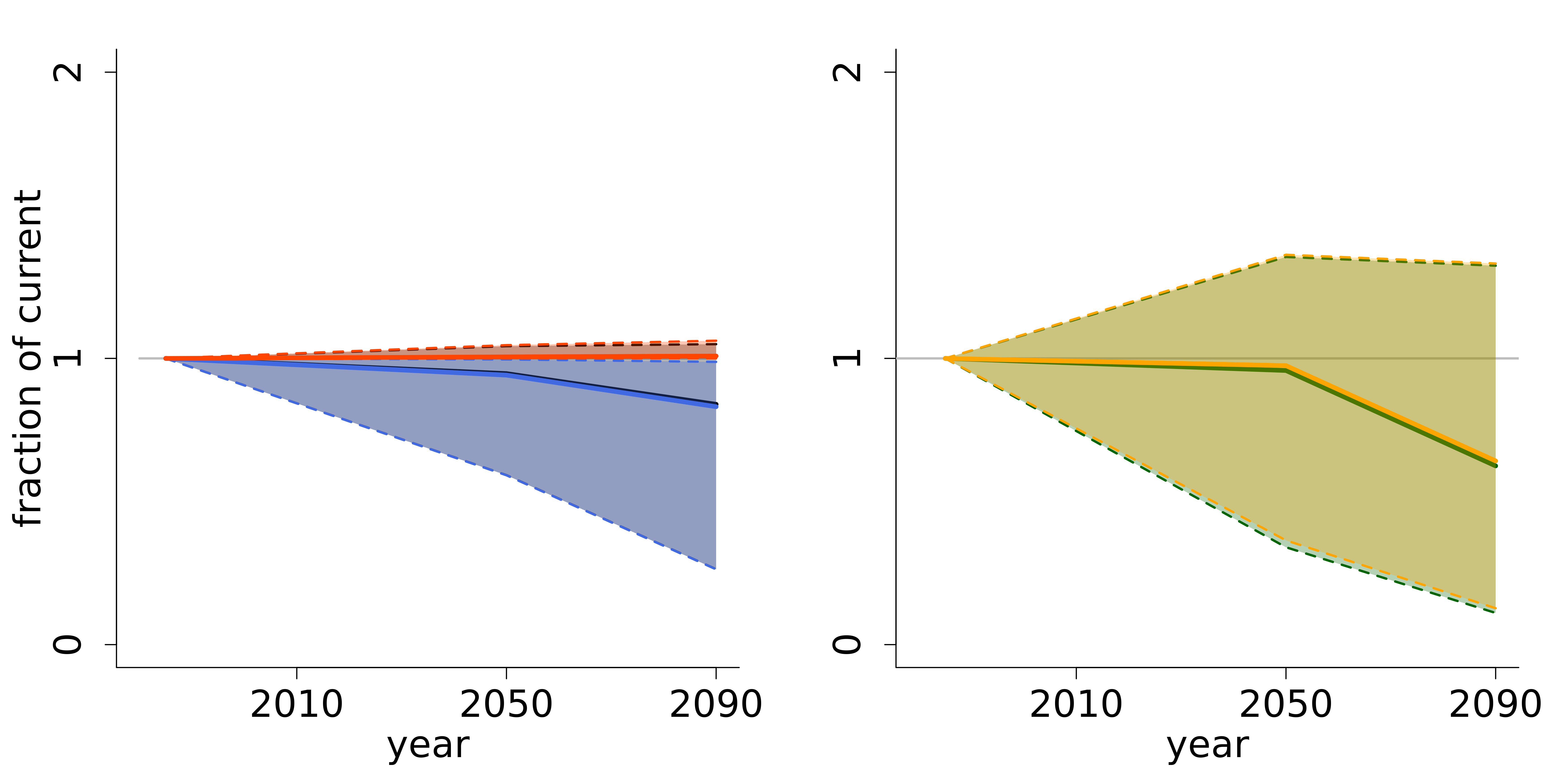

Supplement: S2 Appendix — (ZIP) [file pntd.0014030.s006.zip › Sup. Mat. 6-1 A-L - Species Trends/Bothrocophias_campbelli_CCTrends.png]

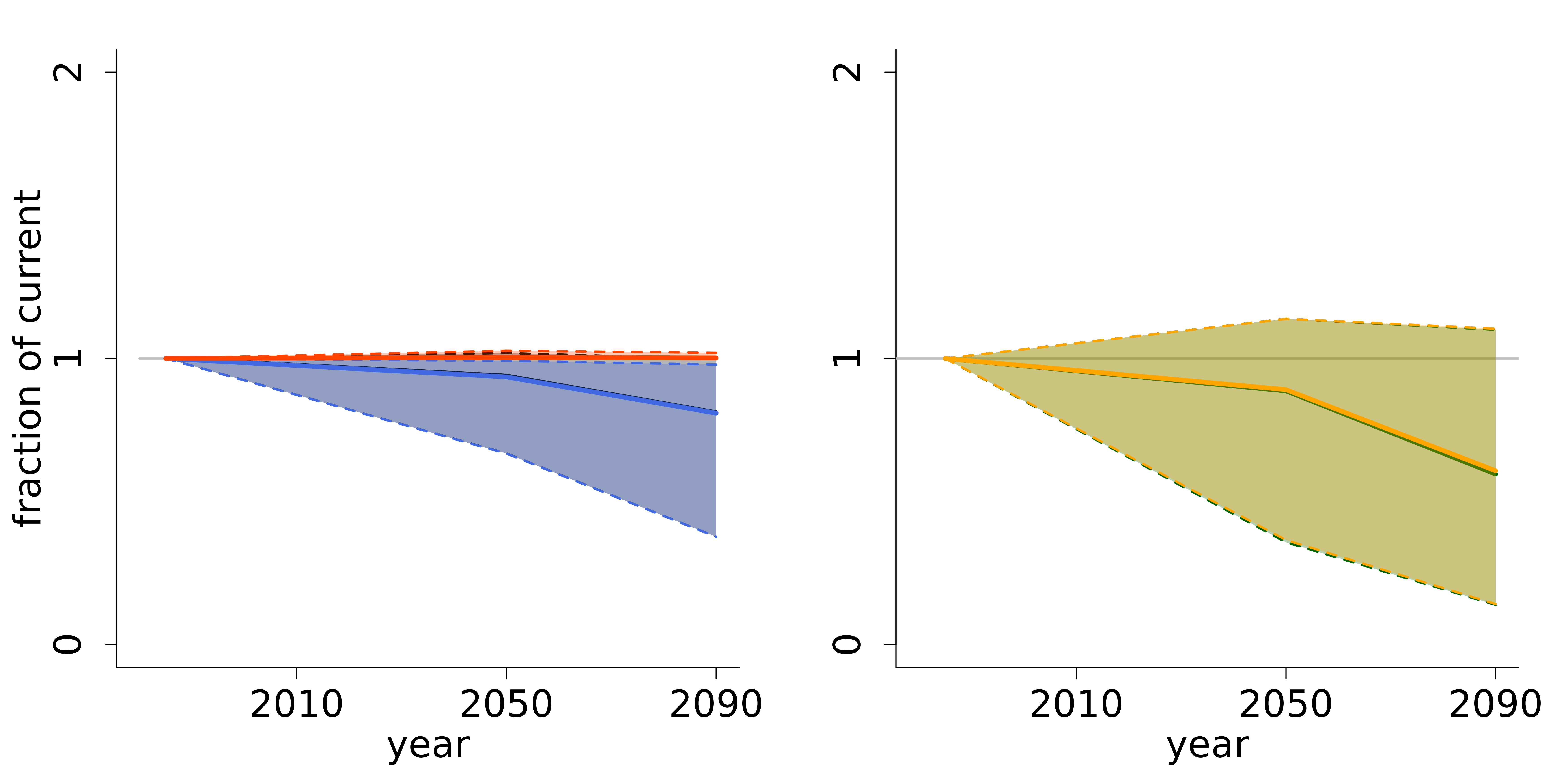

Supplement: S2 Appendix — (ZIP) [file pntd.0014030.s006.zip › Sup. Mat. 6-1 A-L - Species Trends/Bothrocophias_colombianus_CCTrends.png]

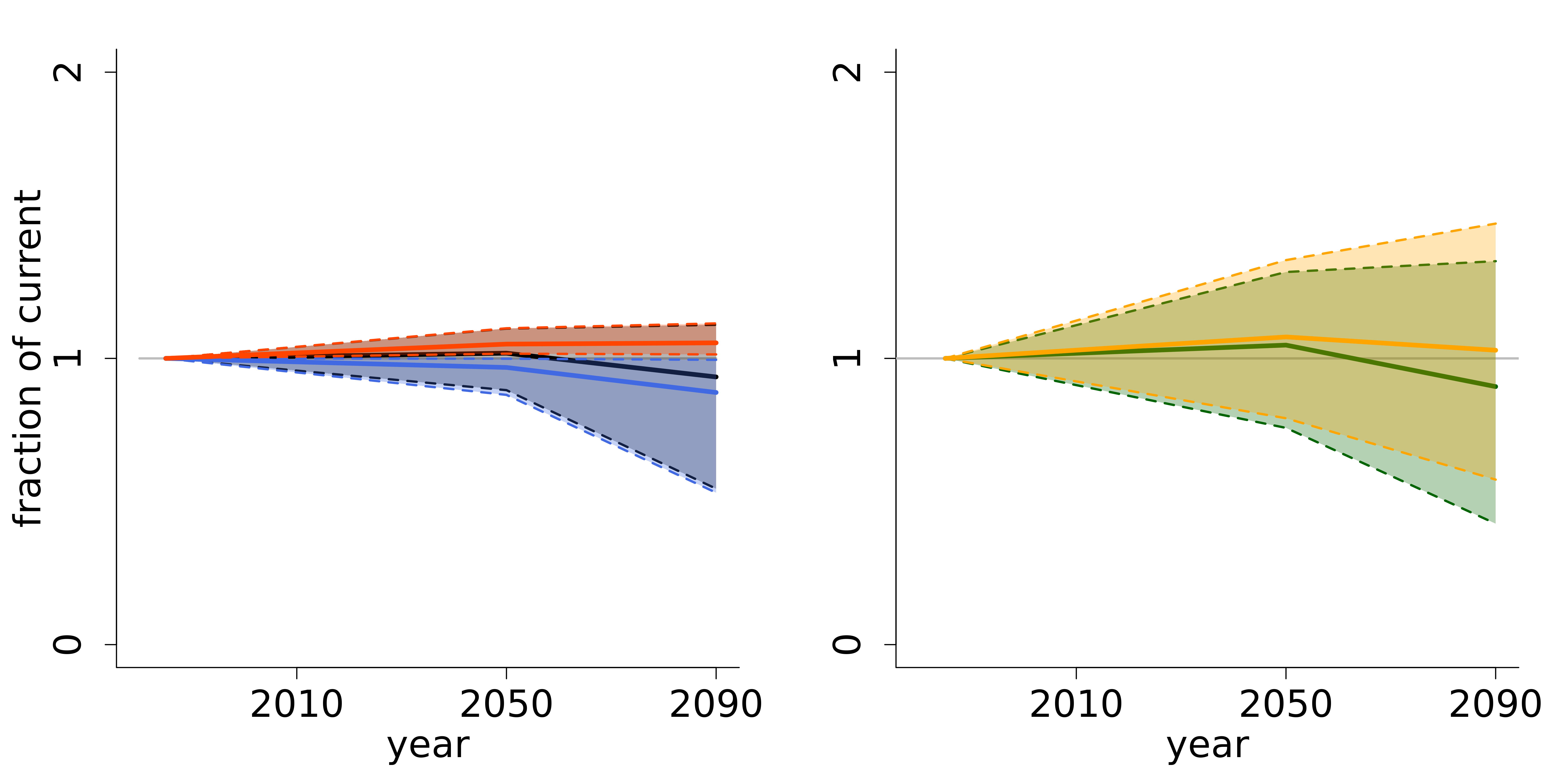

Supplement: S2 Appendix — (ZIP) [file pntd.0014030.s006.zip › Sup. Mat. 6-1 A-L - Species Trends/Bothrocophias_hyoprora_CCTrends.png]

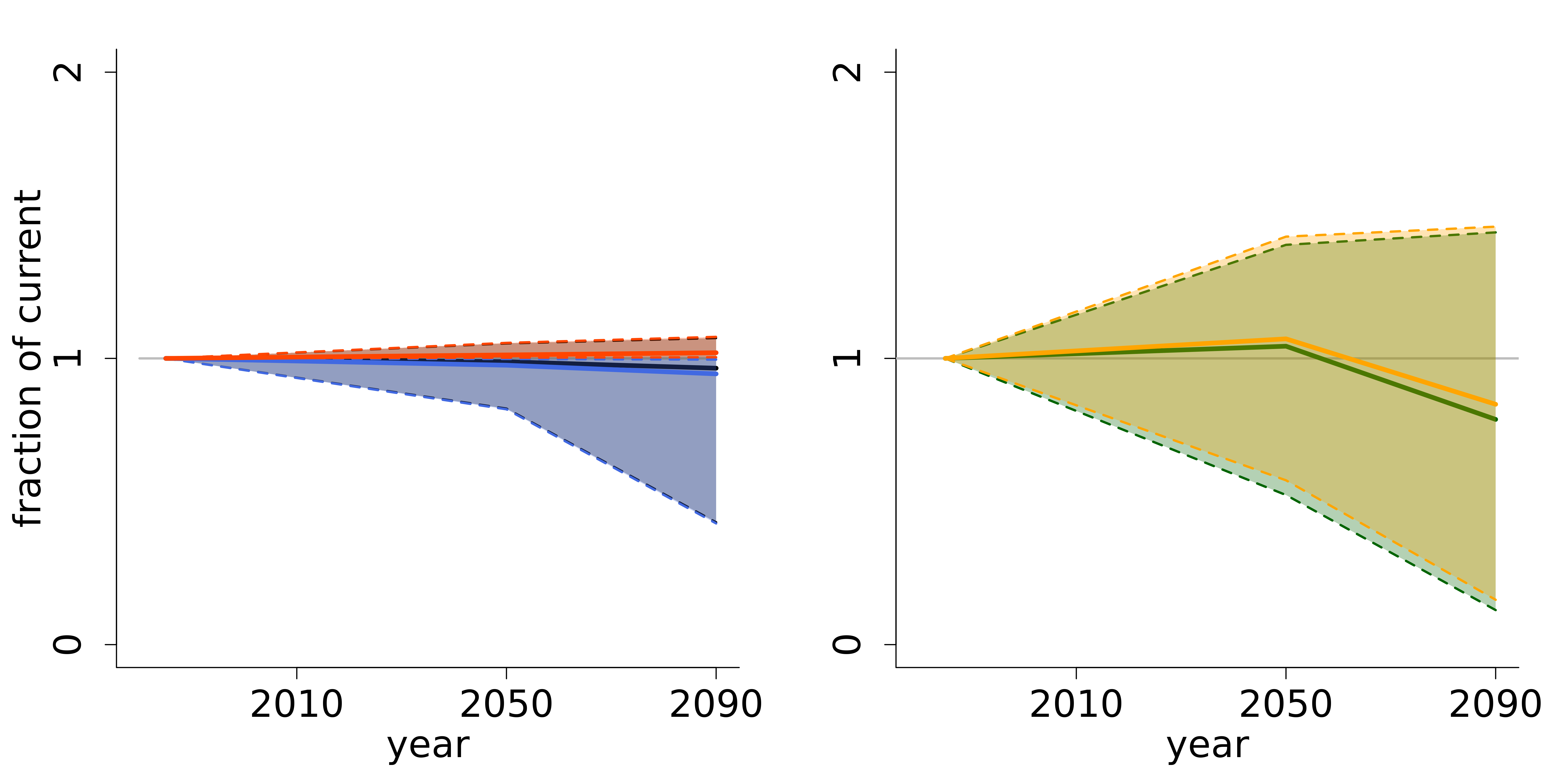

Supplement: S2 Appendix — (ZIP) [file pntd.0014030.s006.zip › Sup. Mat. 6-1 A-L - Species Trends/Bothrocophias_lojanus_CCTrends.png]

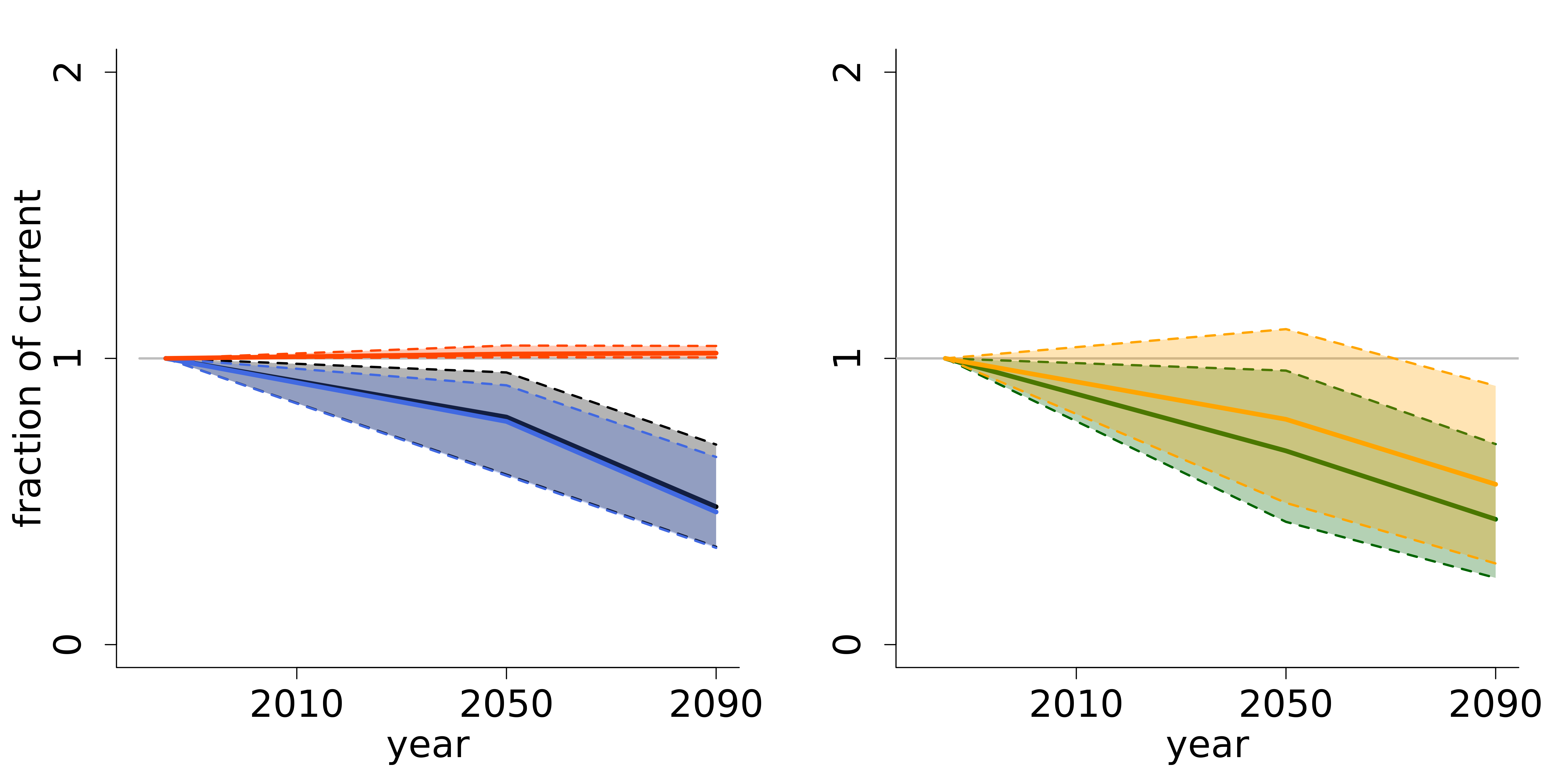

Supplement: S2 Appendix — (ZIP) [file pntd.0014030.s006.zip › Sup. Mat. 6-1 A-L - Species Trends/Bothrocophias_microphthalmus_CCTrends.png]

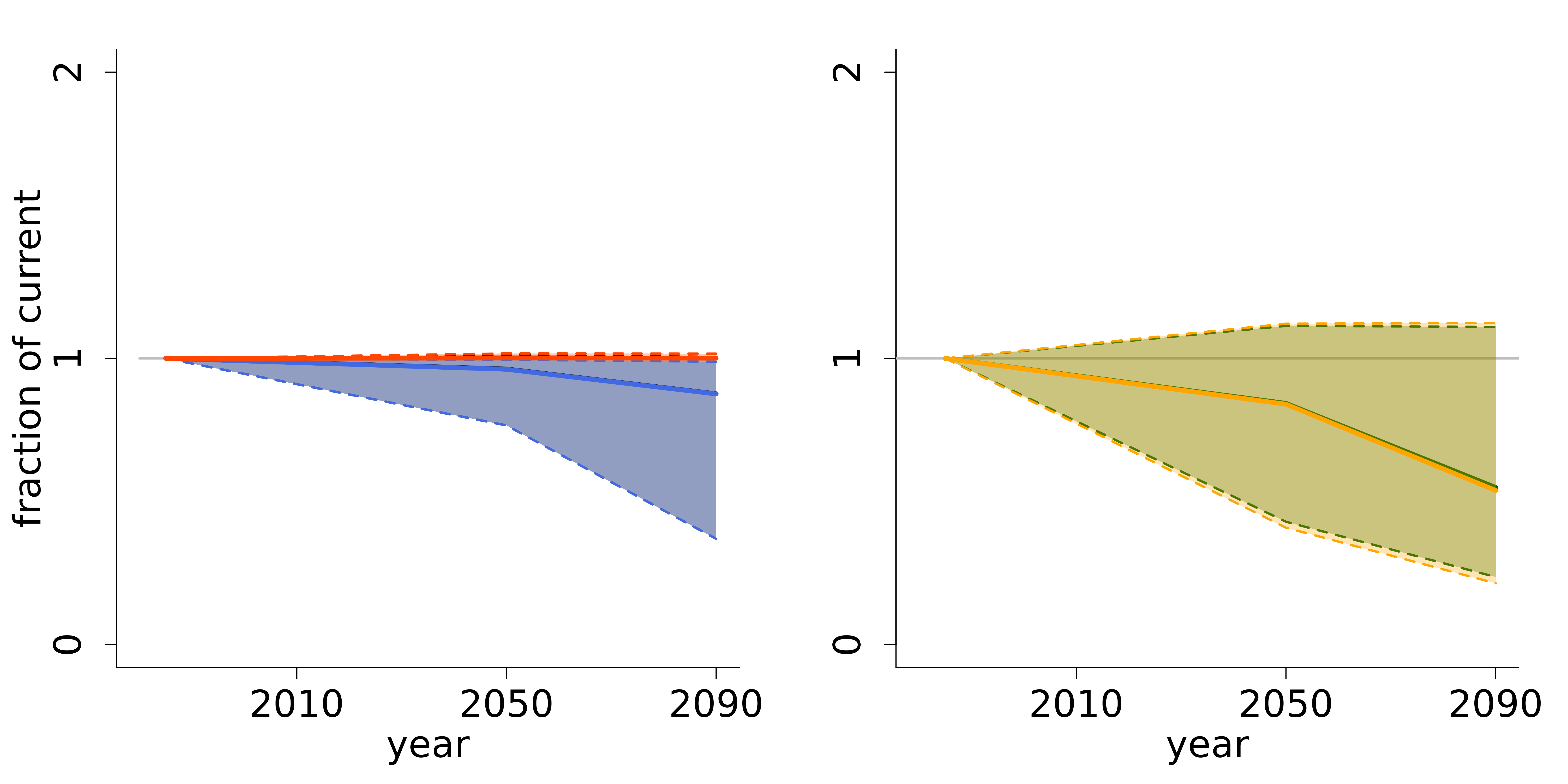

Supplement: S2 Appendix — (ZIP) [file pntd.0014030.s006.zip › Sup. Mat. 6-1 A-L - Species Trends/Bothrocophias_myersi_CCTrends.png]

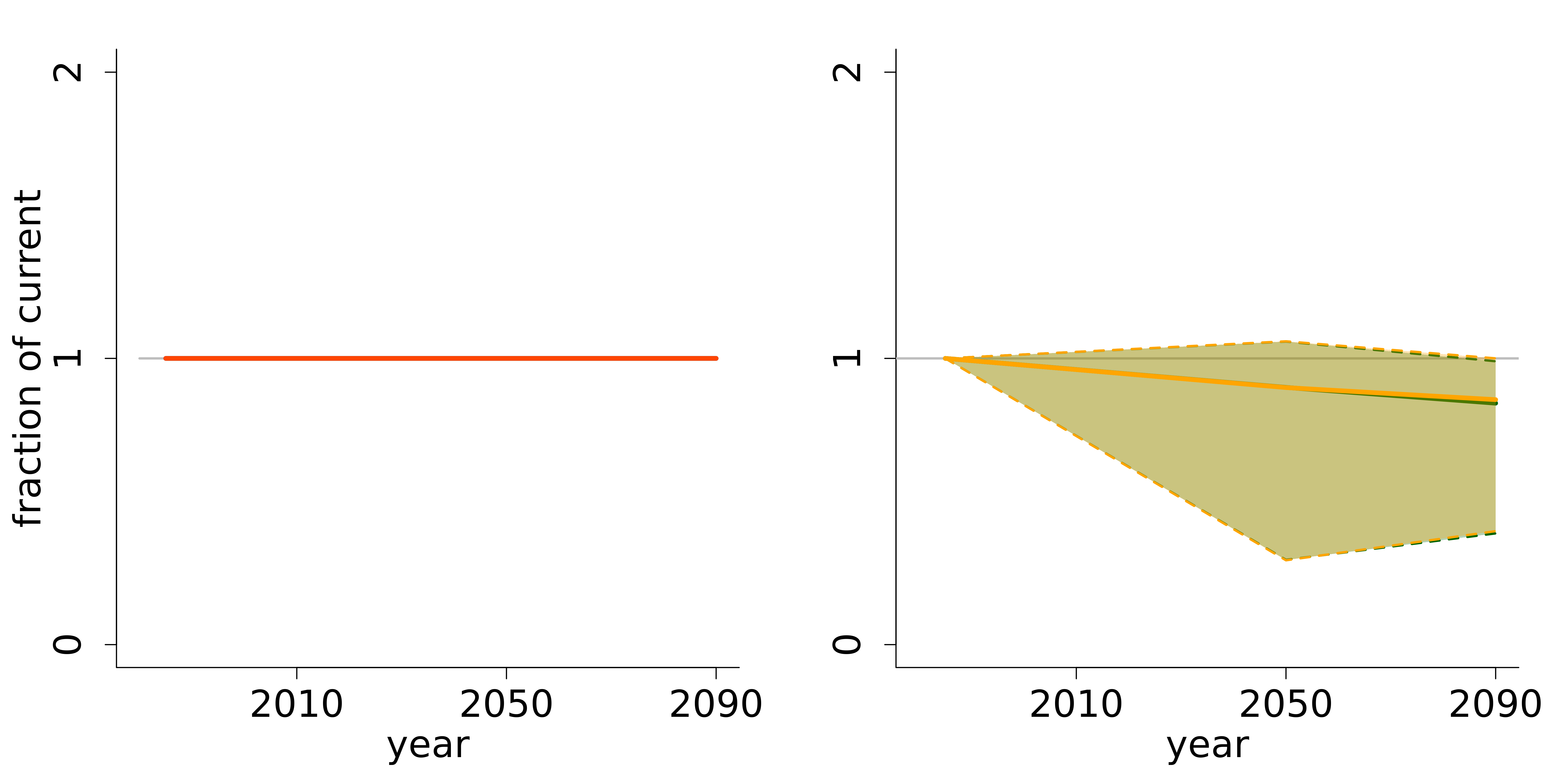

Supplement: S2 Appendix — (ZIP) [file pntd.0014030.s006.zip › Sup. Mat. 6-1 A-L - Species Trends/Bothrops_alcatraz_CCTrends.png]

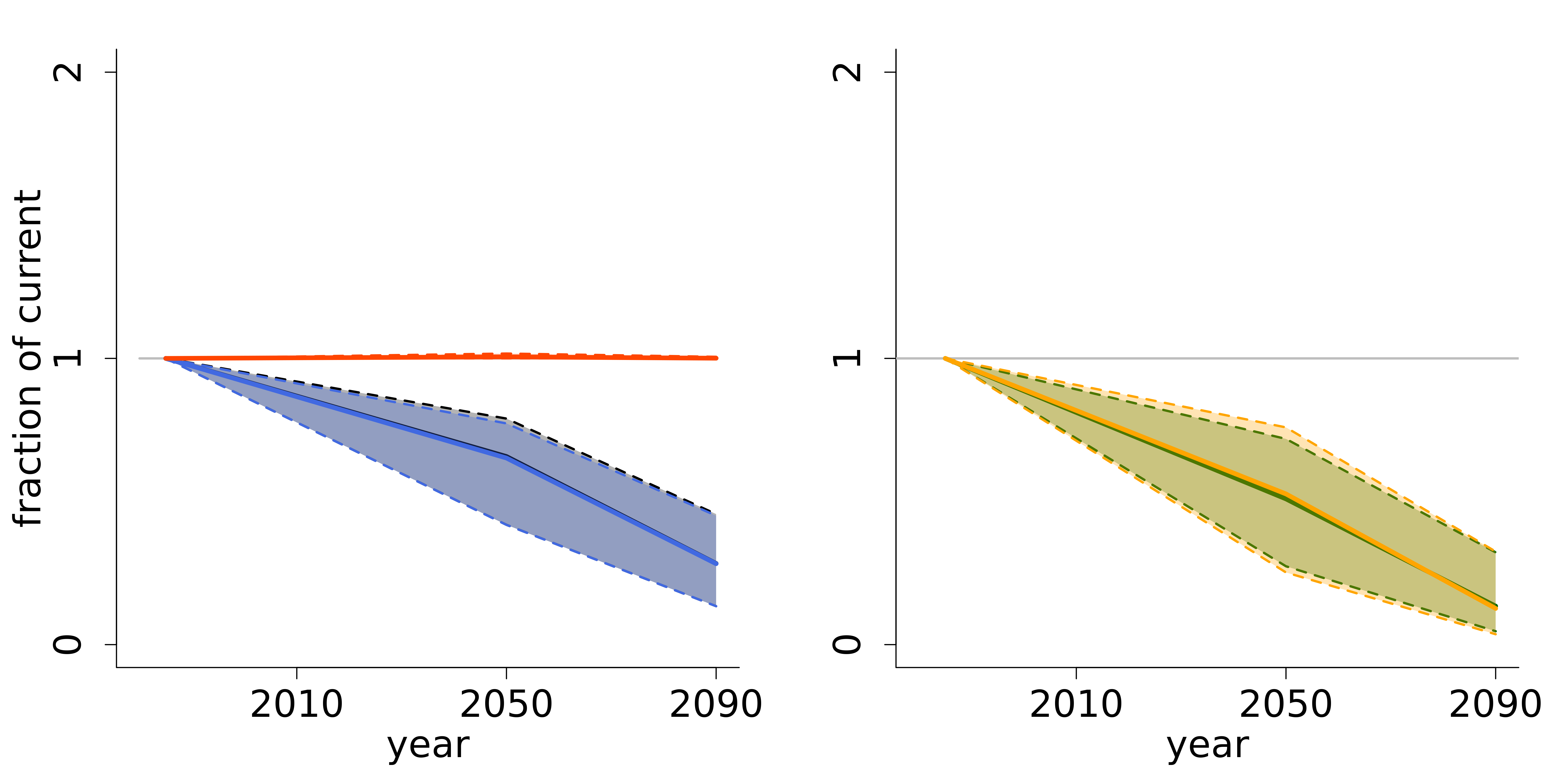

Supplement: S2 Appendix — (ZIP) [file pntd.0014030.s006.zip › Sup. Mat. 6-1 A-L - Species Trends/Bothrops_alternatus_CCTrends.png]

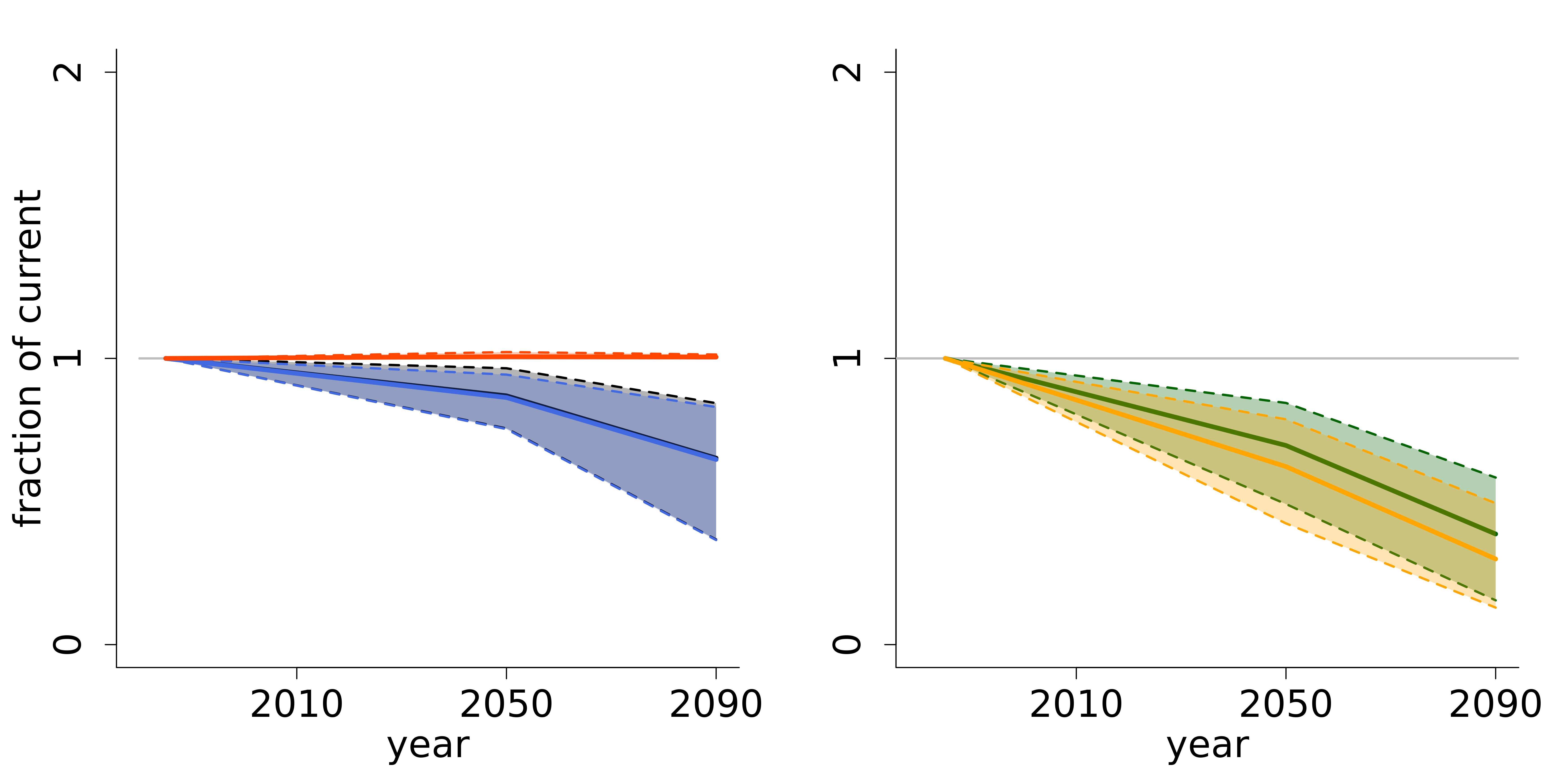

Supplement: S2 Appendix — (ZIP) [file pntd.0014030.s006.zip › Sup. Mat. 6-1 A-L - Species Trends/Bothrops_ammodytoides_CCTrends.png]

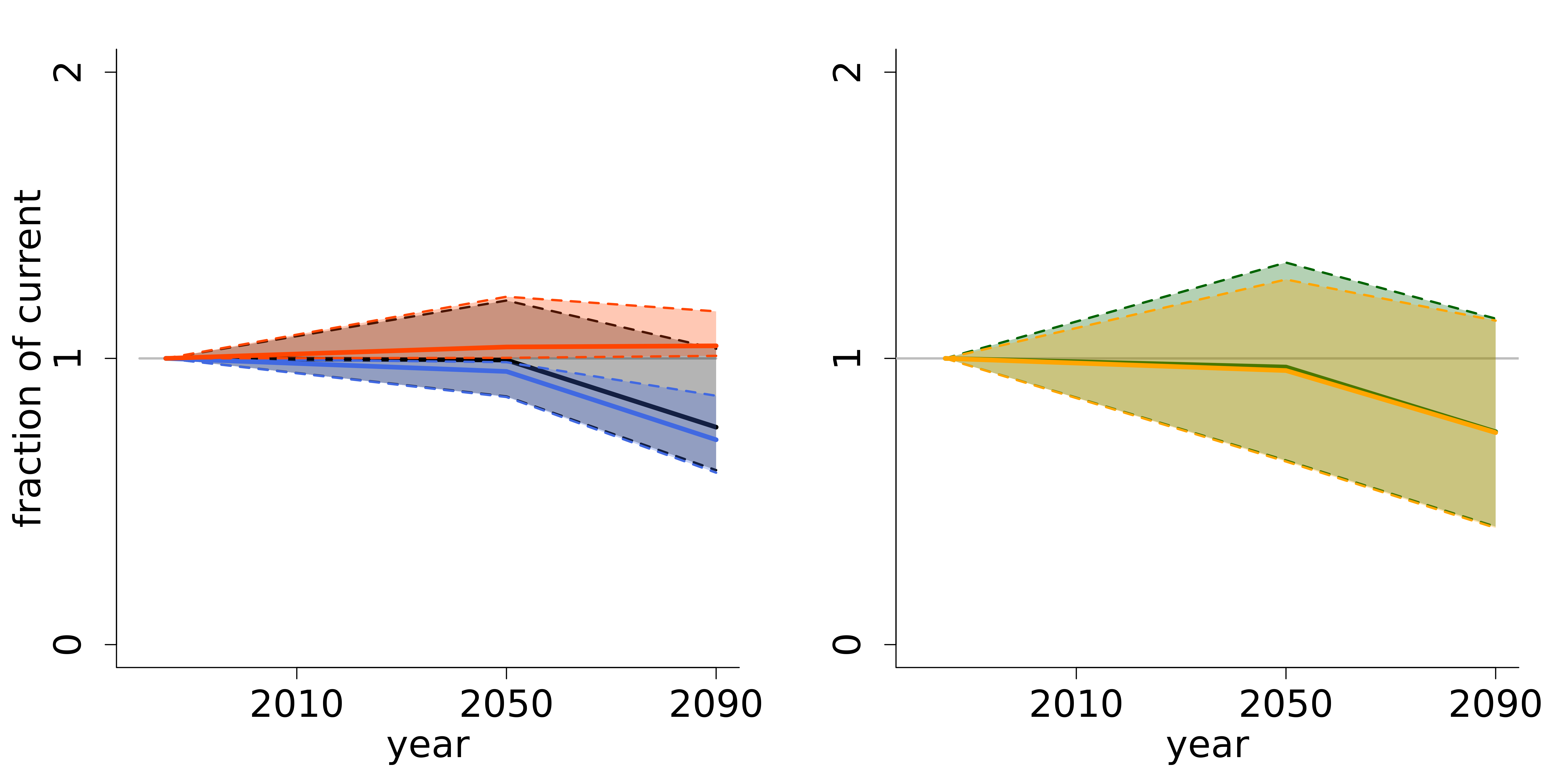

Supplement: S2 Appendix — (ZIP) [file pntd.0014030.s006.zip › Sup. Mat. 6-1 A-L - Species Trends/Bothrops_asper_CCTrends.png]

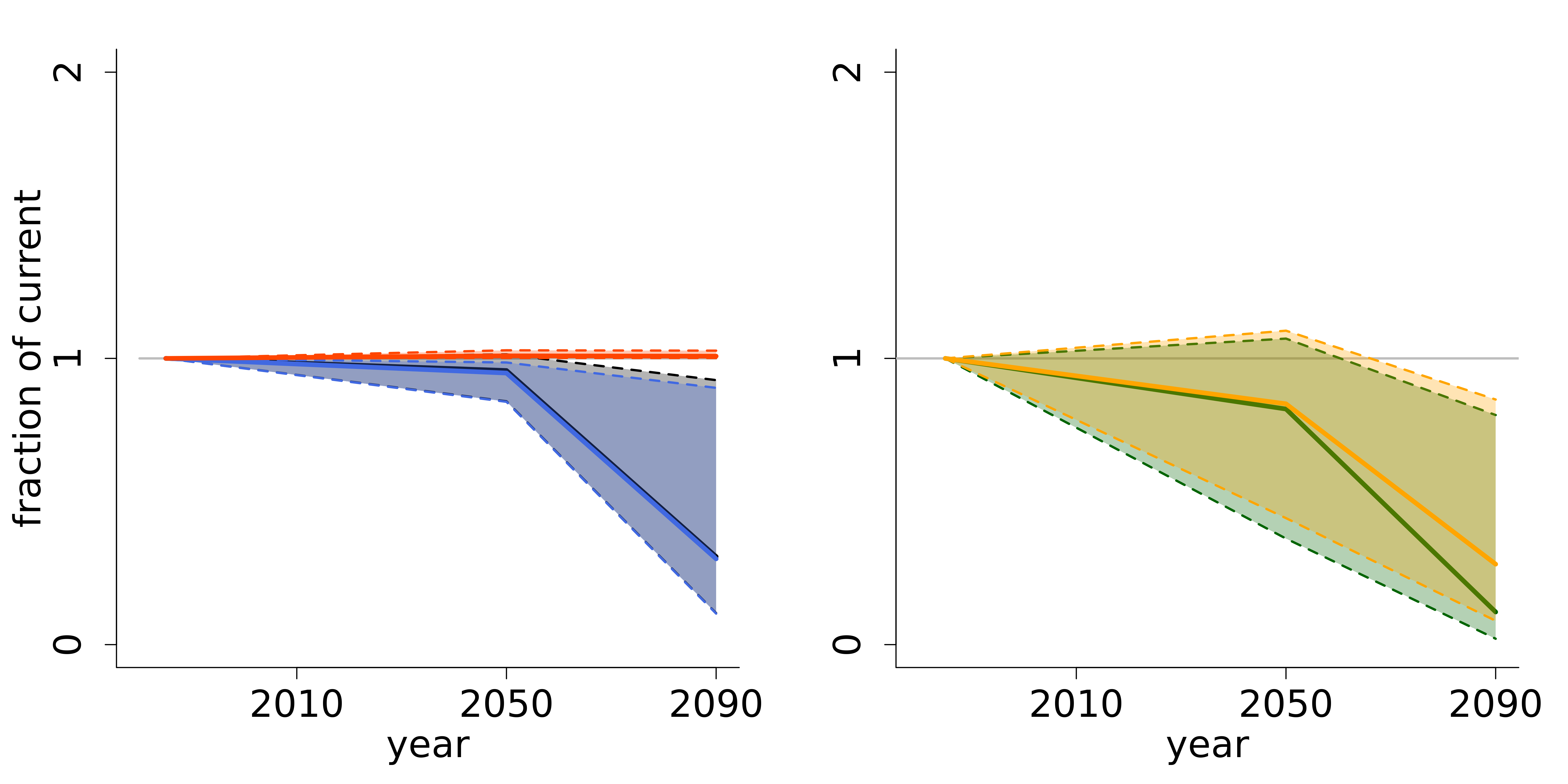

Supplement: S2 Appendix — (ZIP) [file pntd.0014030.s006.zip › Sup. Mat. 6-1 A-L - Species Trends/Bothrops_atrox_CCTrends.png]

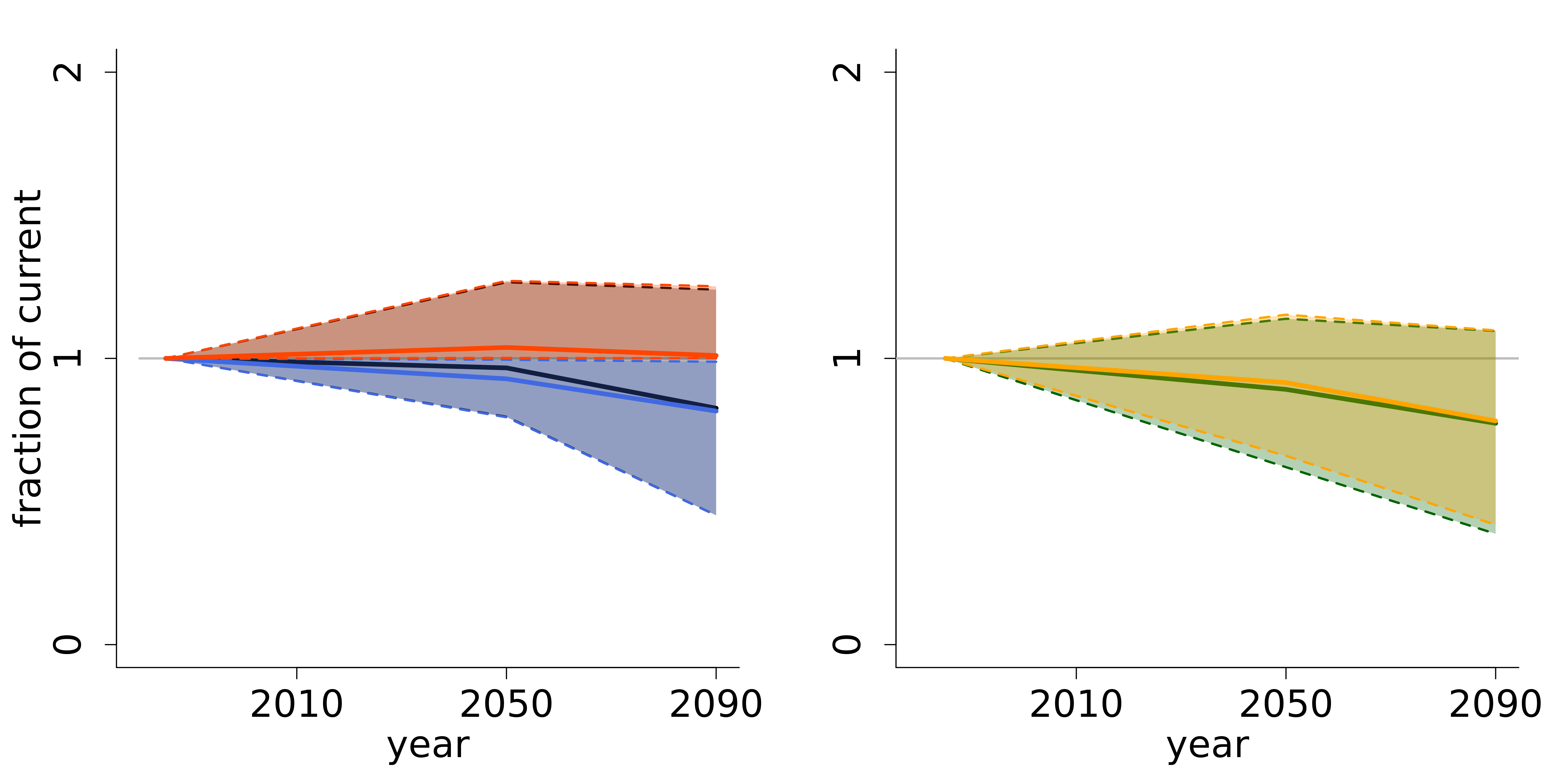

Supplement: S2 Appendix — (ZIP) [file pntd.0014030.s006.zip › Sup. Mat. 6-1 A-L - Species Trends/Bothrops_barnetti_CCTrends.png]

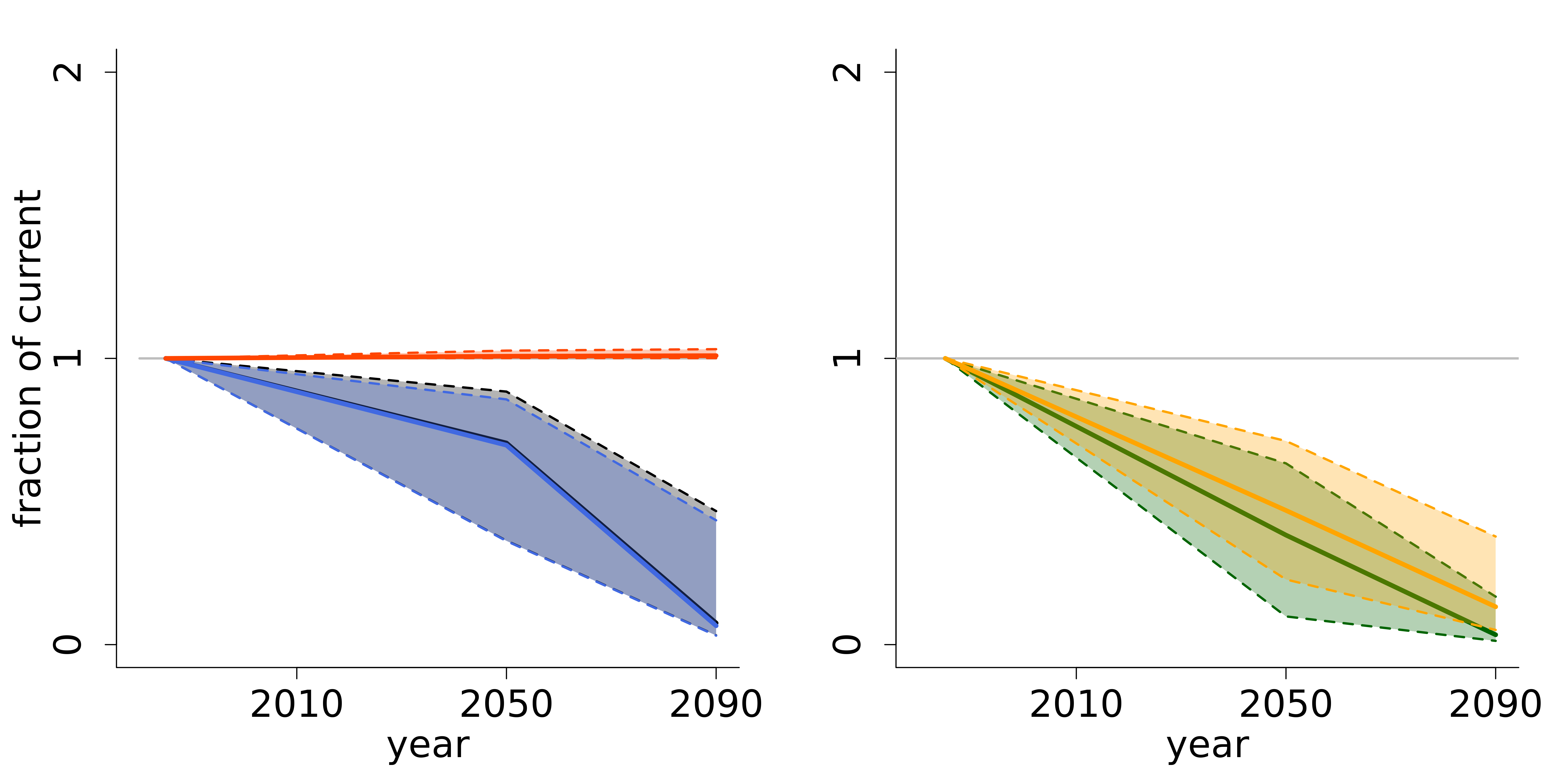

Supplement: S2 Appendix — (ZIP) [file pntd.0014030.s006.zip › Sup. Mat. 6-1 A-L - Species Trends/Bothrops_bilineatus_CCTrends.png]

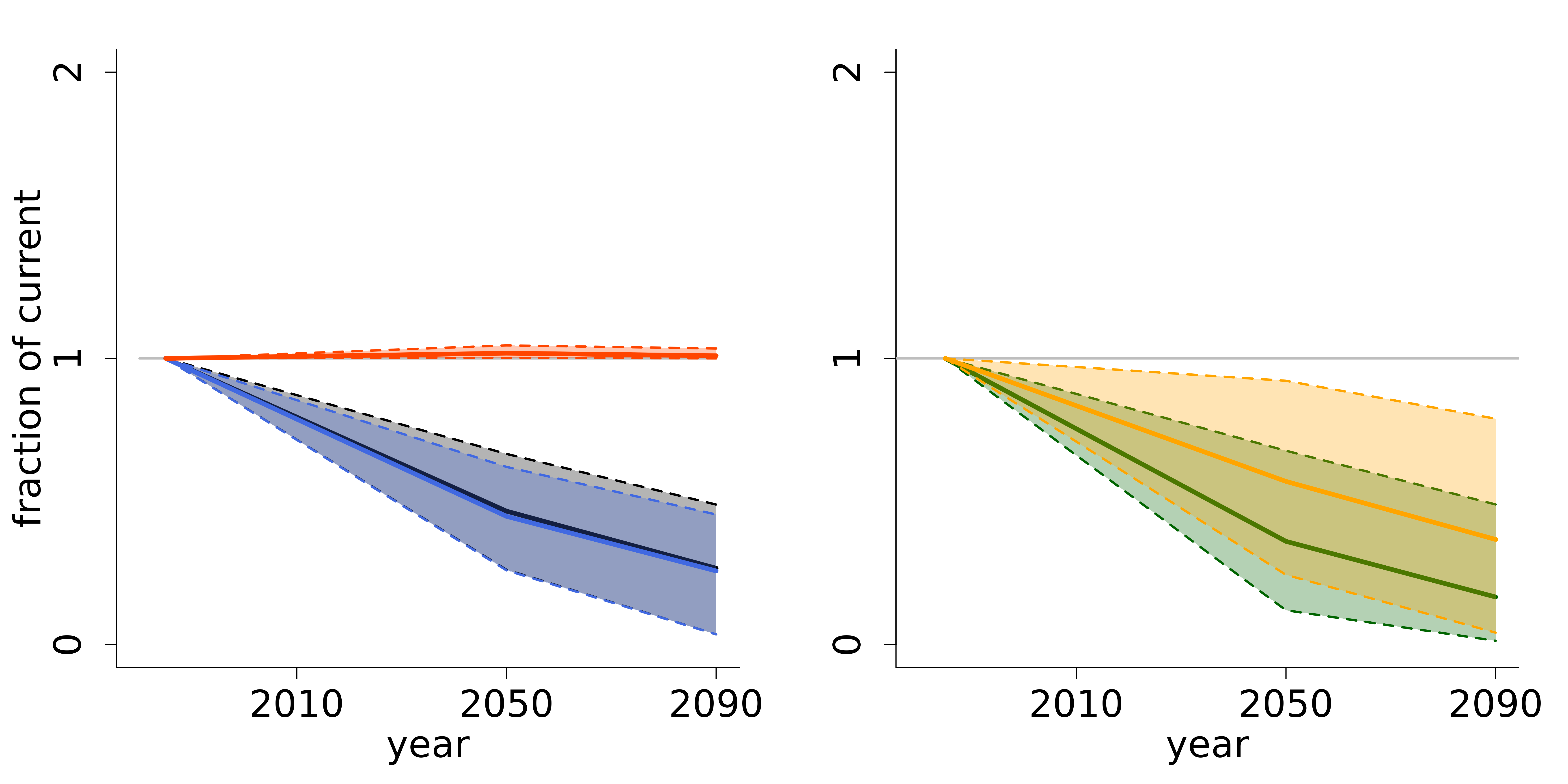

Supplement: S2 Appendix — (ZIP) [file pntd.0014030.s006.zip › Sup. Mat. 6-1 A-L - Species Trends/Bothrops_brazili_CCTrends.png]

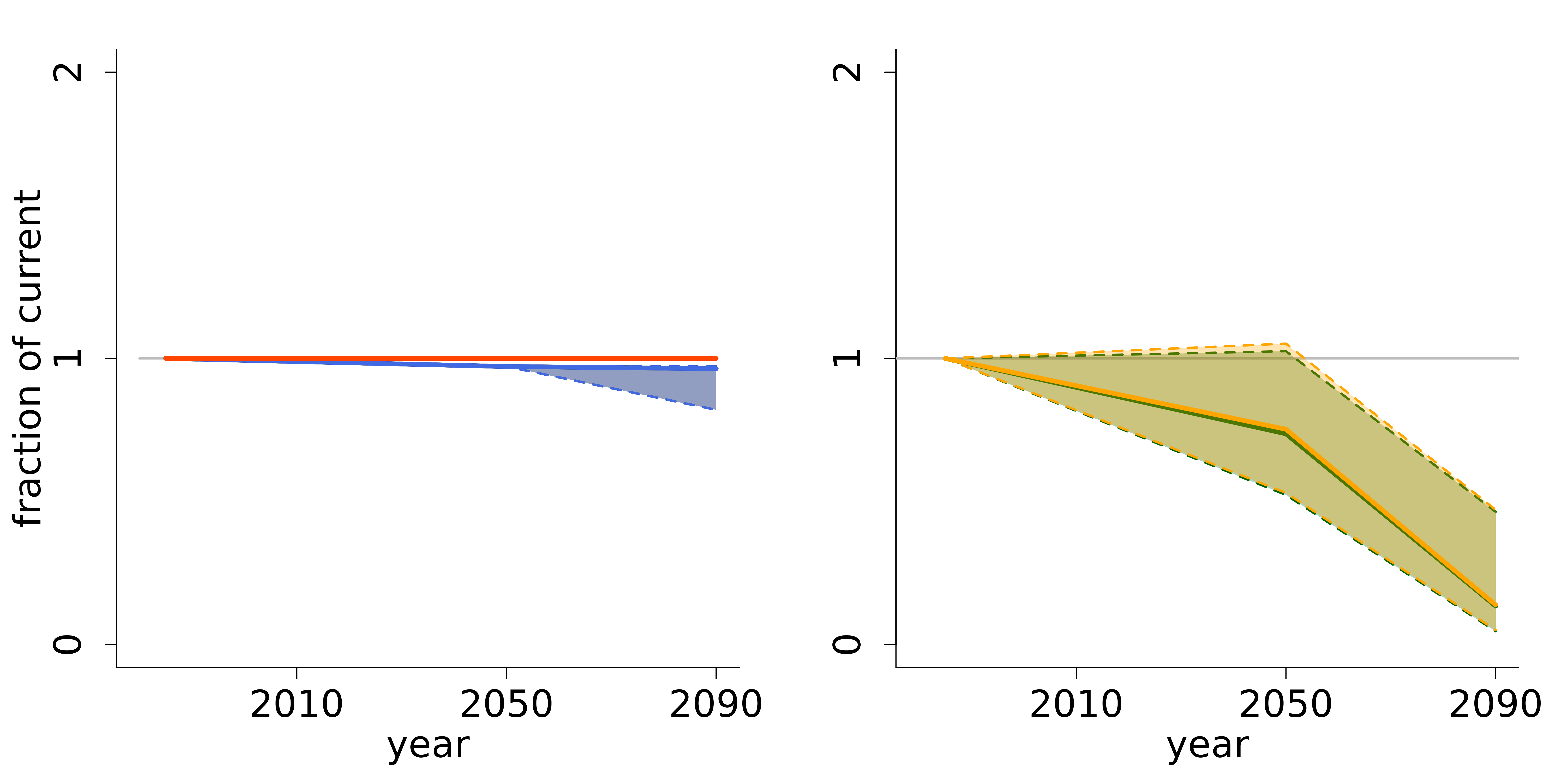

Supplement: S2 Appendix — (ZIP) [file pntd.0014030.s006.zip › Sup. Mat. 6-1 A-L - Species Trends/Bothrops_caribbaeus_CCTrends.png]

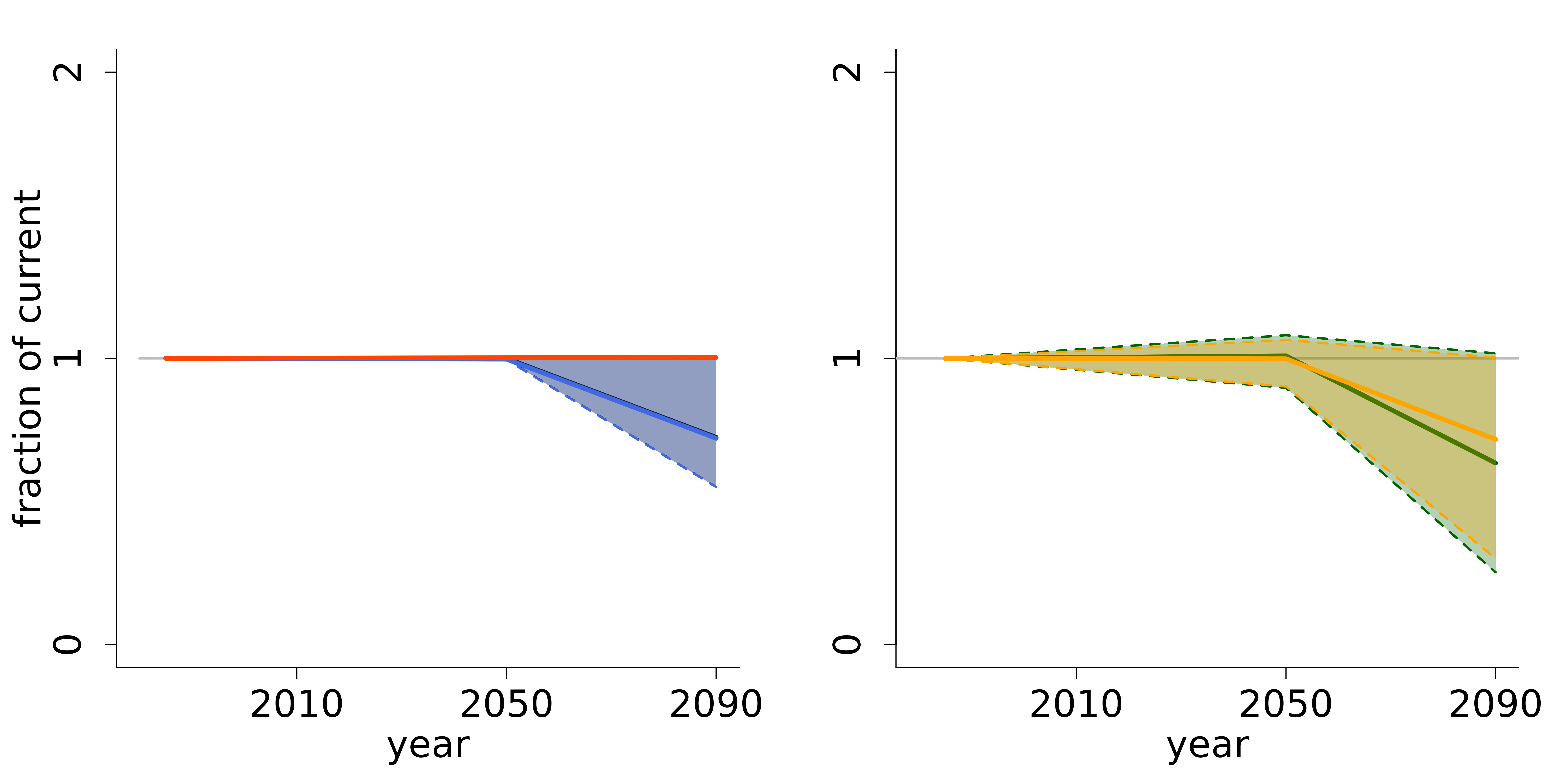

Supplement: S2 Appendix — (ZIP) [file pntd.0014030.s006.zip › Sup. Mat. 6-1 A-L - Species Trends/Bothrops_chloromelas_CCTrends.png]

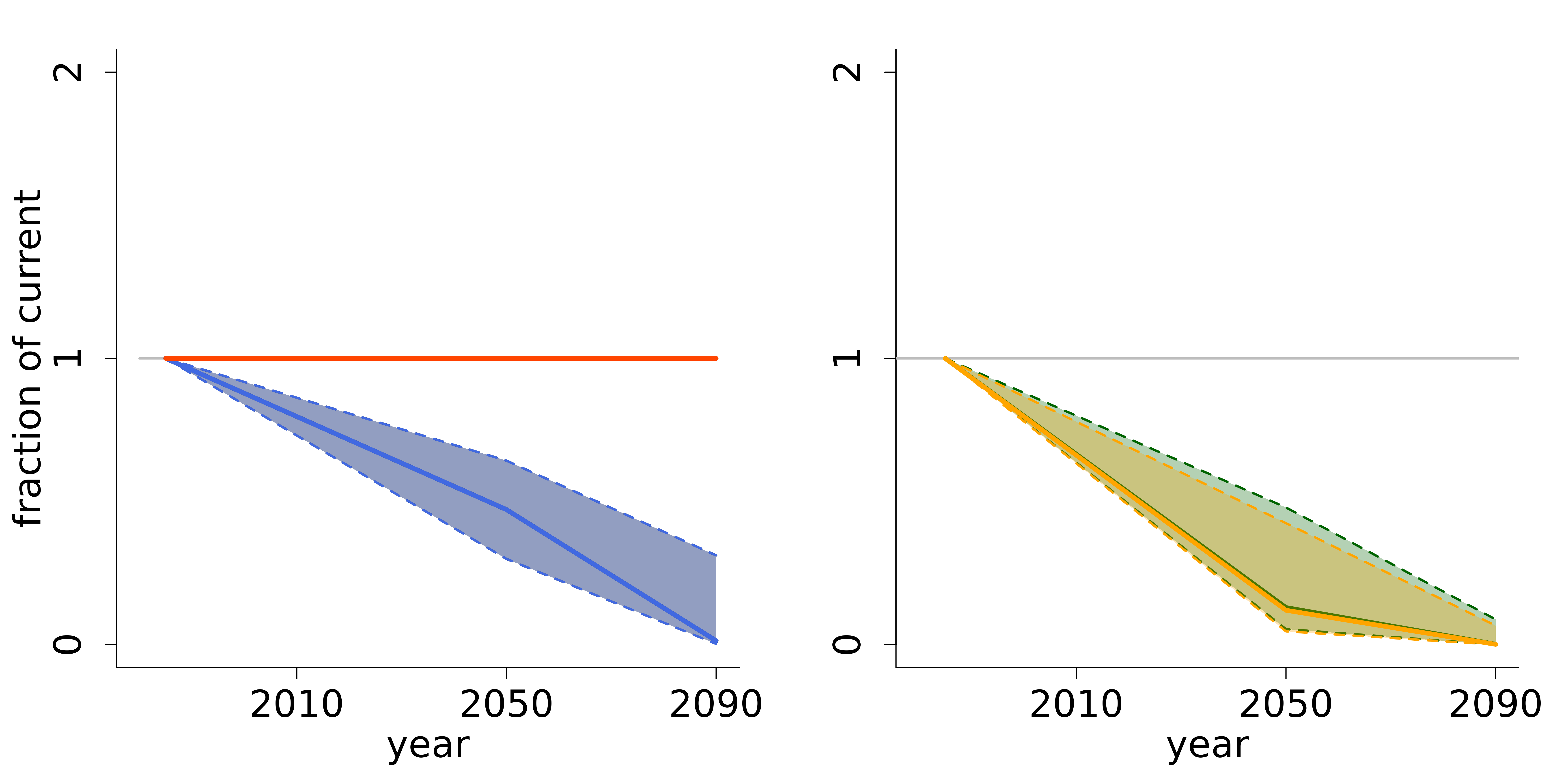

Supplement: S2 Appendix — (ZIP) [file pntd.0014030.s006.zip › Sup. Mat. 6-1 A-L - Species Trends/Bothrops_cotiara_CCTrends.png]

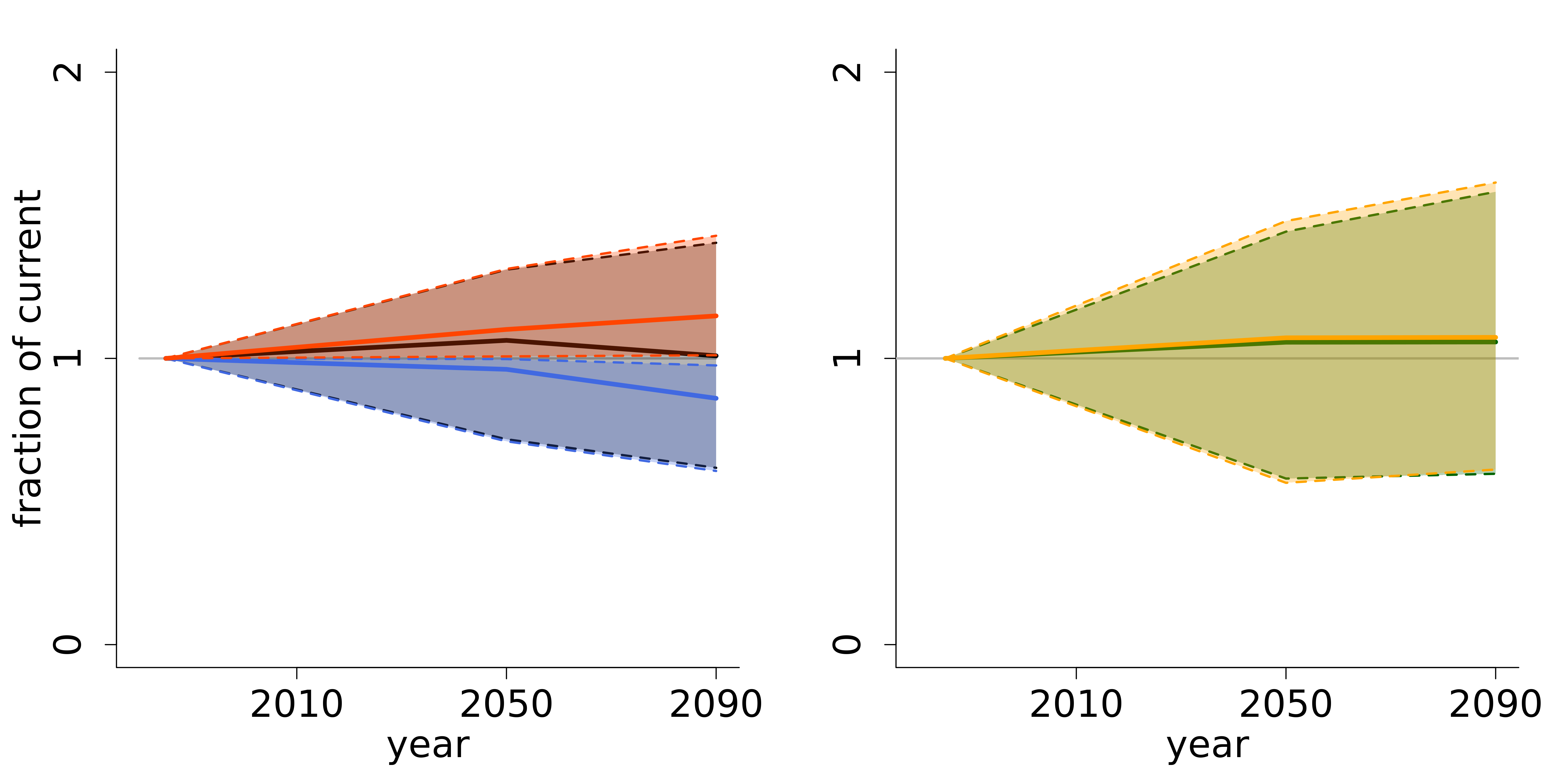

Supplement: S2 Appendix — (ZIP) [file pntd.0014030.s006.zip › Sup. Mat. 6-1 A-L - Species Trends/Bothrops_diporus_CCTrends.png]

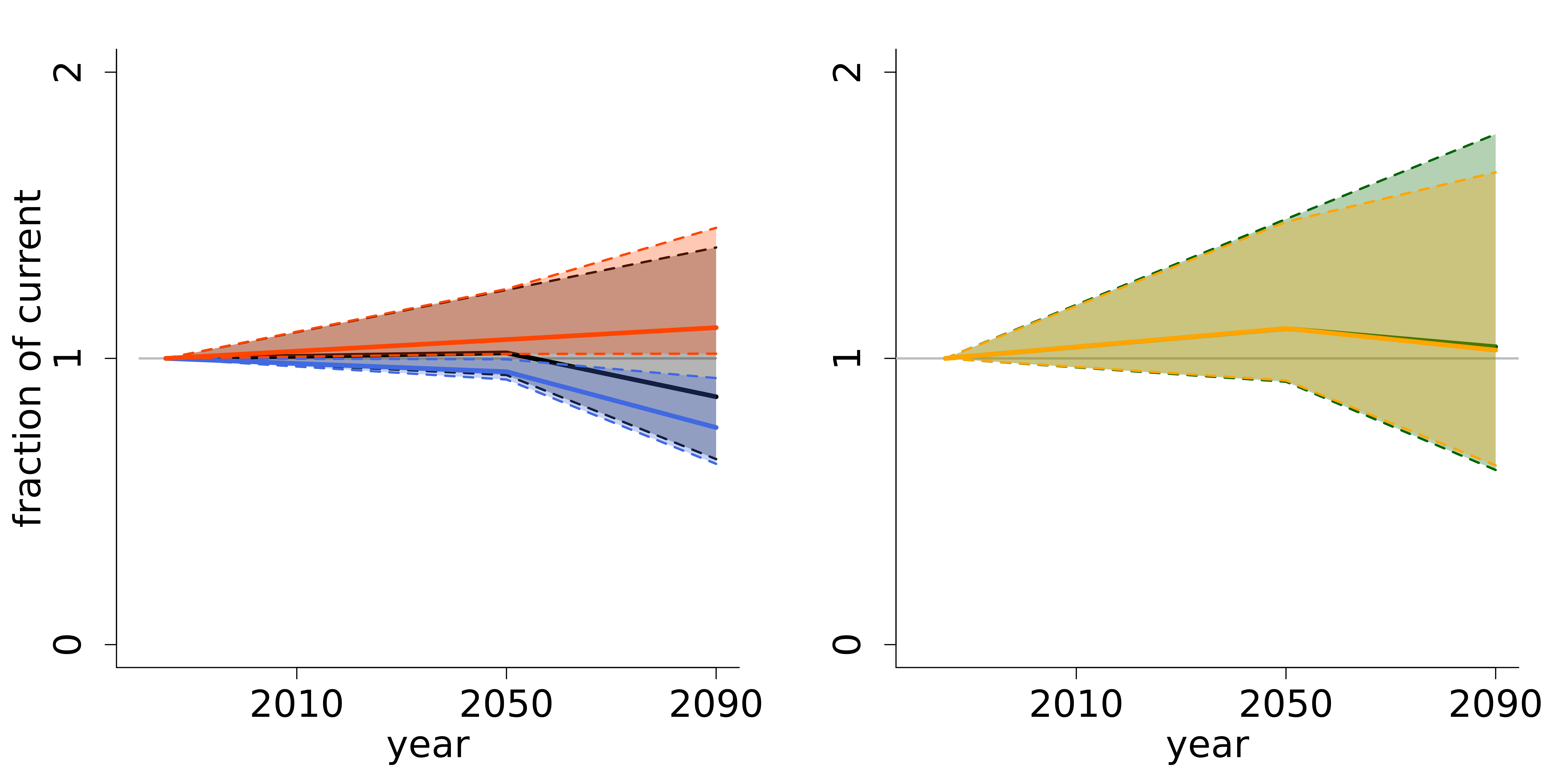

Supplement: S2 Appendix — (ZIP) [file pntd.0014030.s006.zip › Sup. Mat. 6-1 A-L - Species Trends/Bothrops_erythromelas_CCTrends.png]

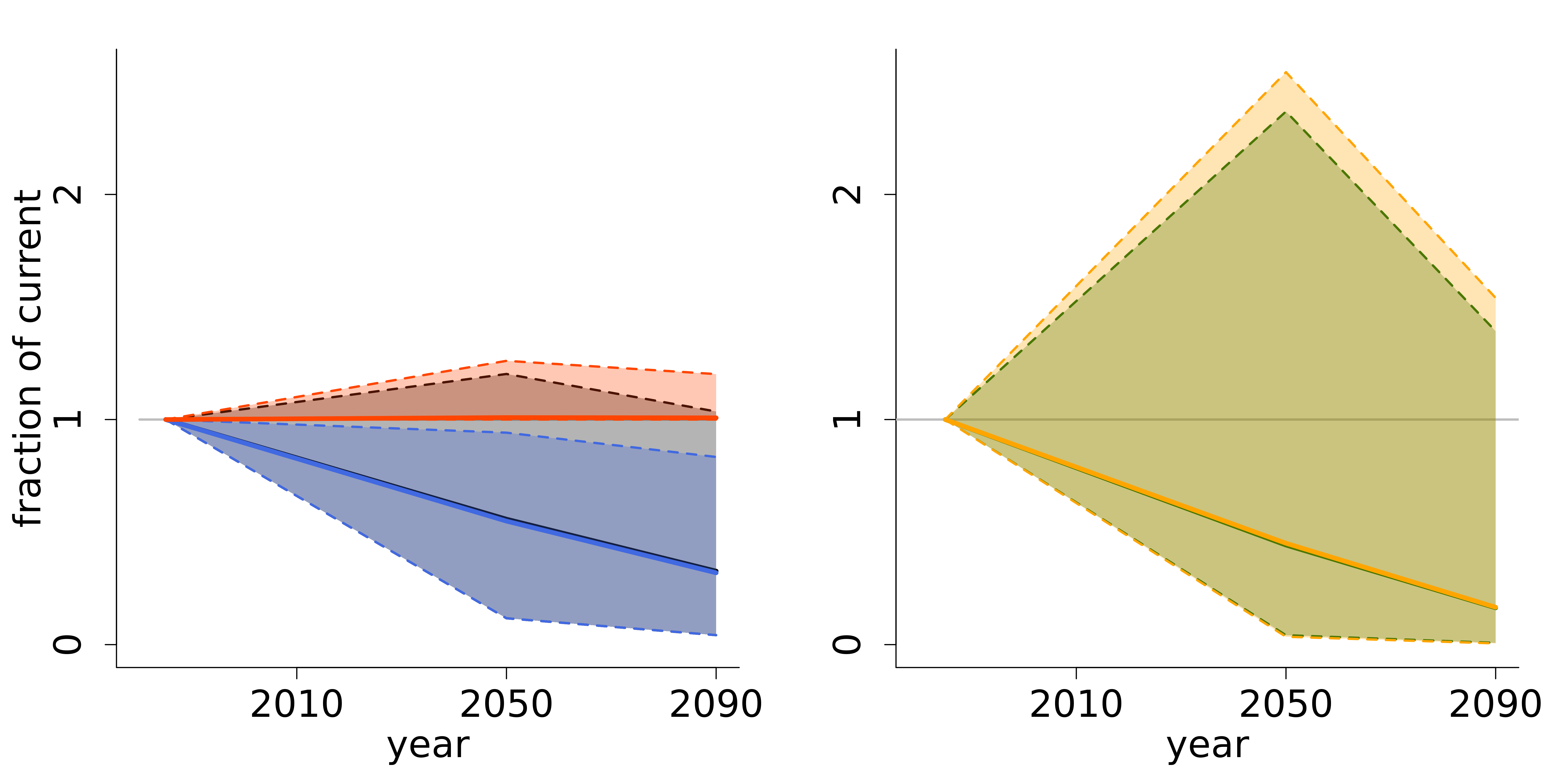

Supplement: S2 Appendix — (ZIP) [file pntd.0014030.s006.zip › Sup. Mat. 6-1 A-L - Species Trends/Bothrops_fonsecai_CCTrends.png]

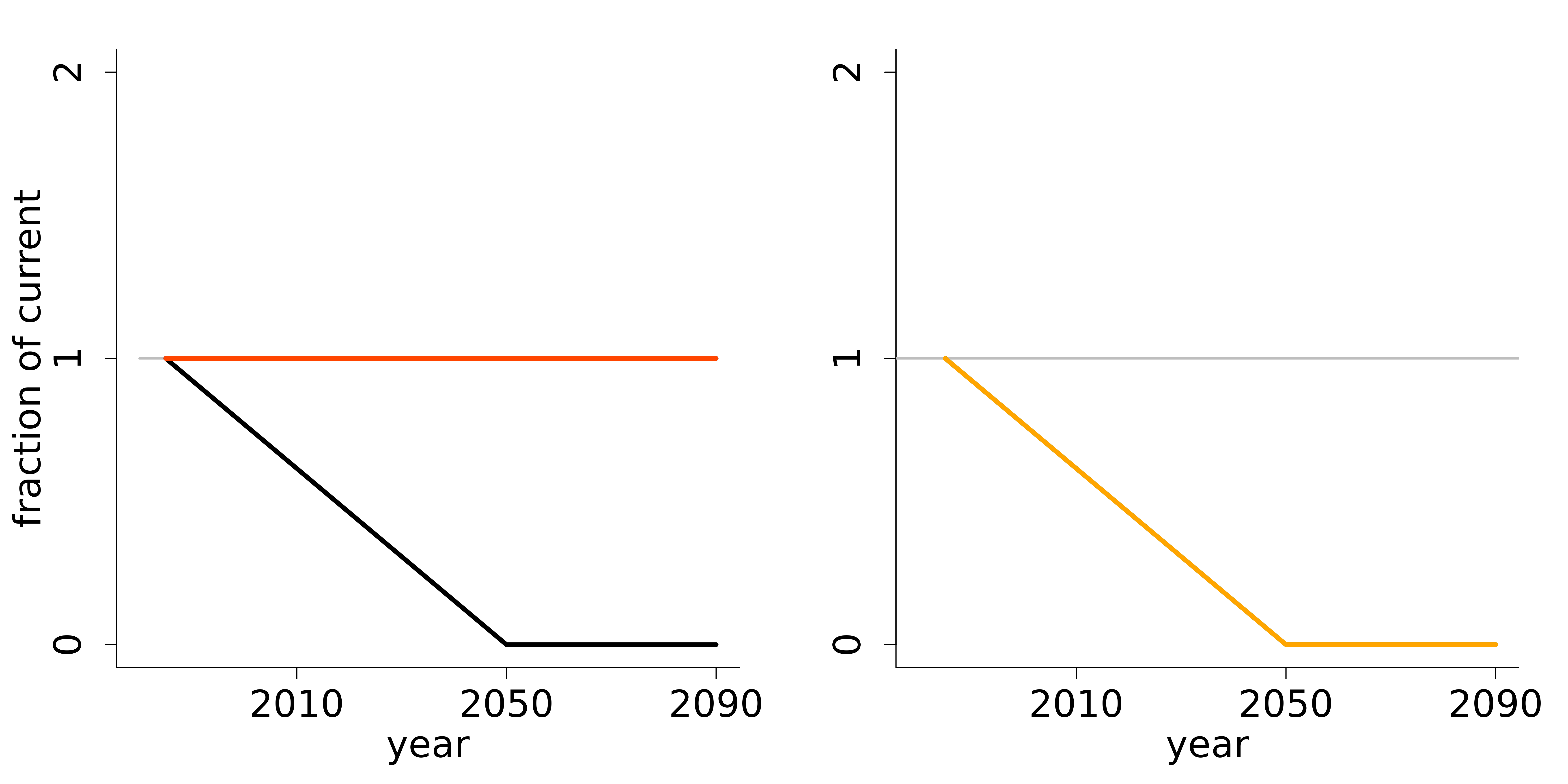

Supplement: S2 Appendix — (ZIP) [file pntd.0014030.s006.zip › Sup. Mat. 6-1 A-L - Species Trends/Bothrops_insularis_CCTrends.png]

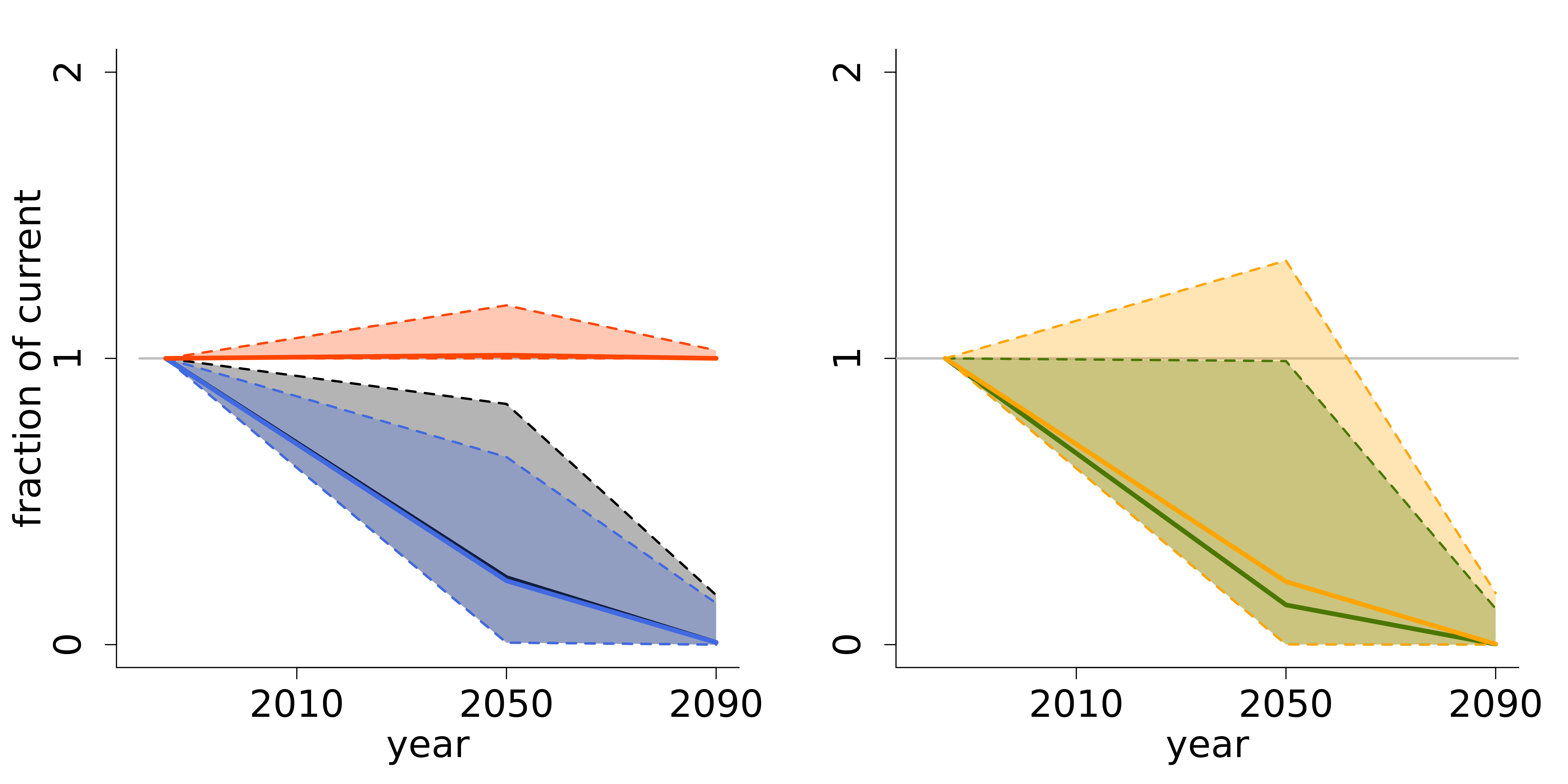

Supplement: S2 Appendix — (ZIP) [file pntd.0014030.s006.zip › Sup. Mat. 6-1 A-L - Species Trends/Bothrops_itapetiningae_CCTrends.png]

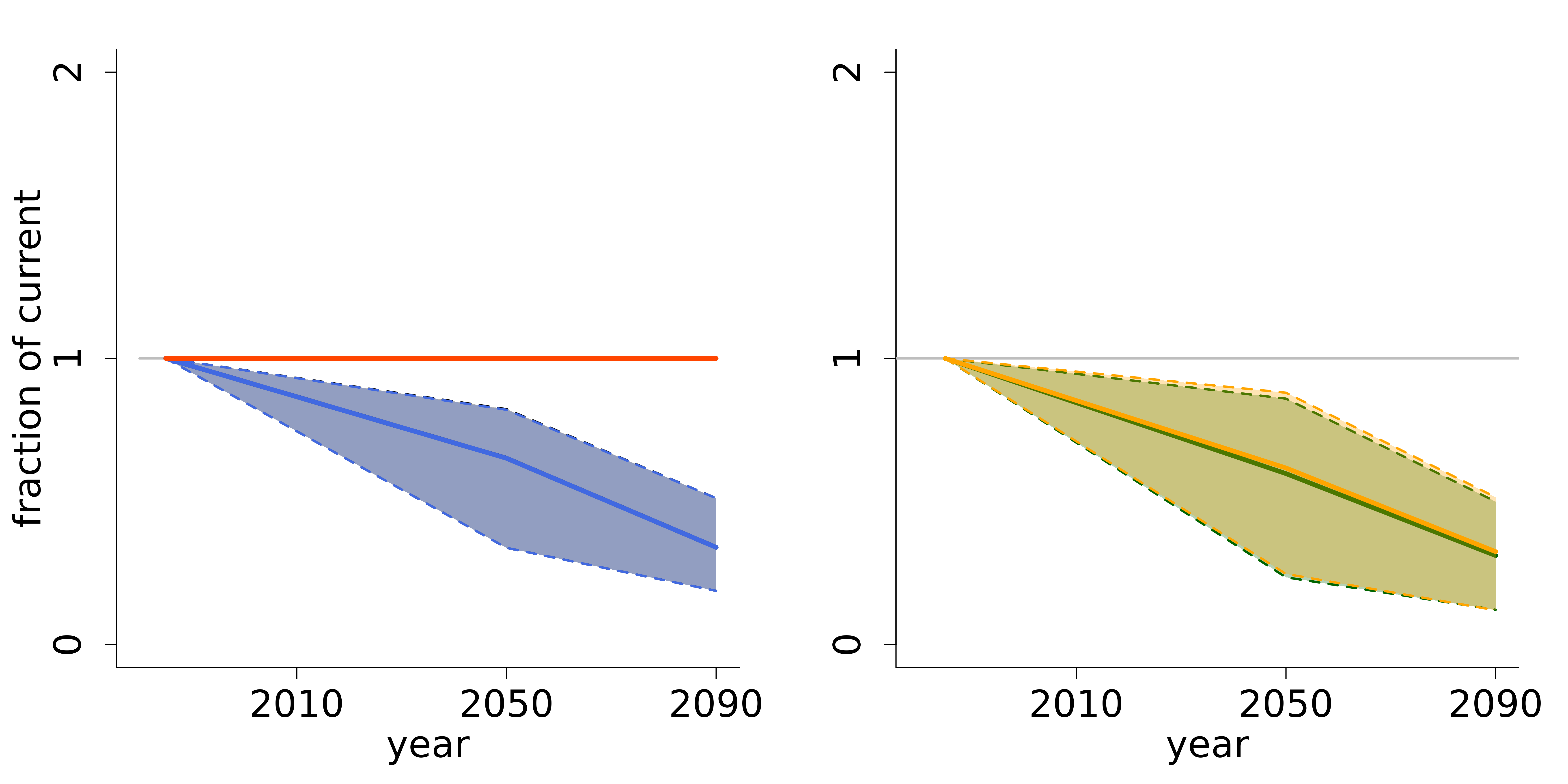

Supplement: S2 Appendix — (ZIP) [file pntd.0014030.s006.zip › Sup. Mat. 6-1 A-L - Species Trends/Bothrops_jararaca_CCTrends.png]

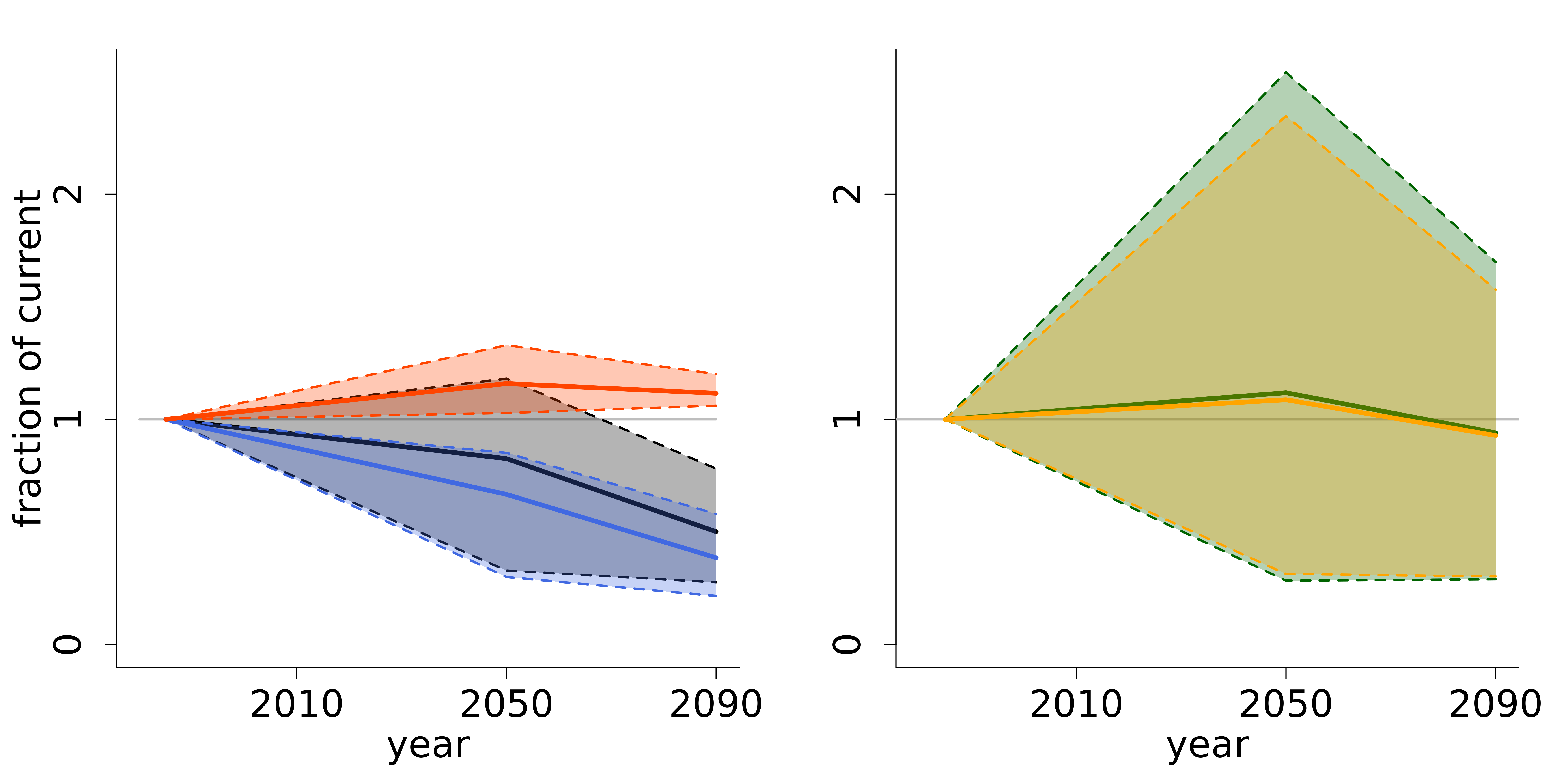

Supplement: S2 Appendix — (ZIP) [file pntd.0014030.s006.zip › Sup. Mat. 6-1 A-L - Species Trends/Bothrops_jararacussu_CCTrends.png]

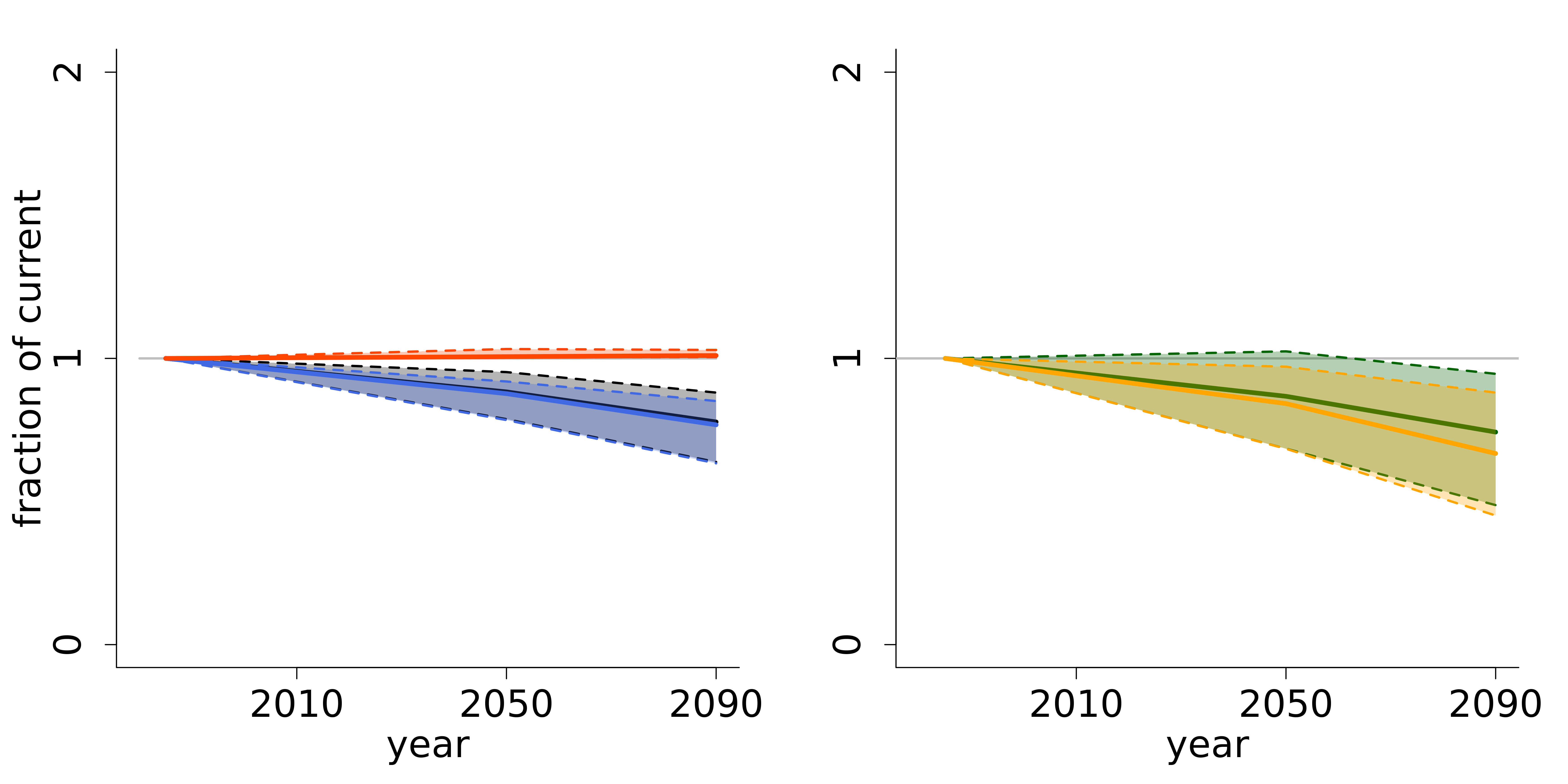

Supplement: S2 Appendix — (ZIP) [file pntd.0014030.s006.zip › Sup. Mat. 6-1 A-L - Species Trends/Bothrops_jonathani_CCTrends.png]

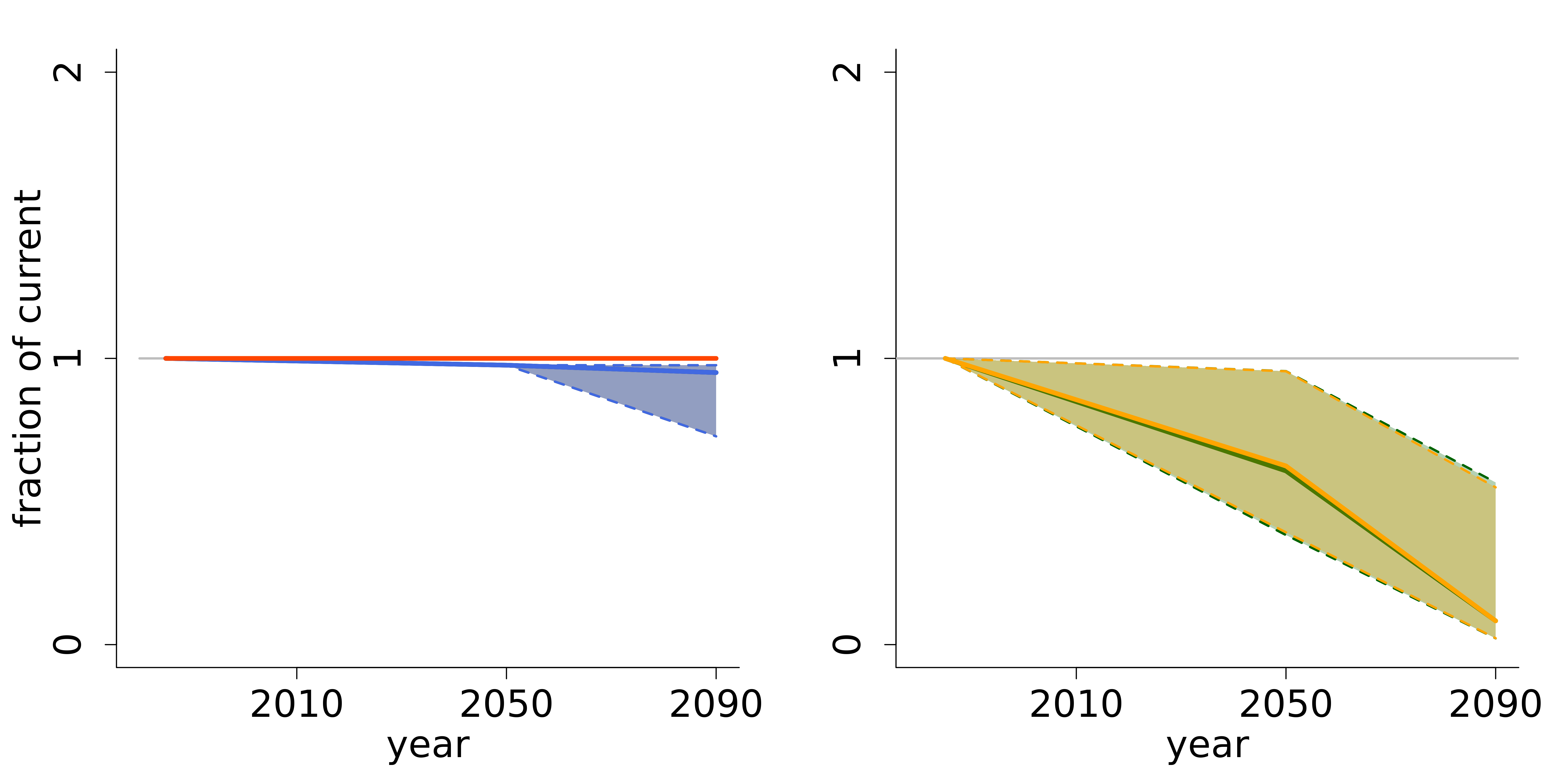

Supplement: S2 Appendix — (ZIP) [file pntd.0014030.s006.zip › Sup. Mat. 6-1 A-L - Species Trends/Bothrops_lanceolatus_CCTrends.png]

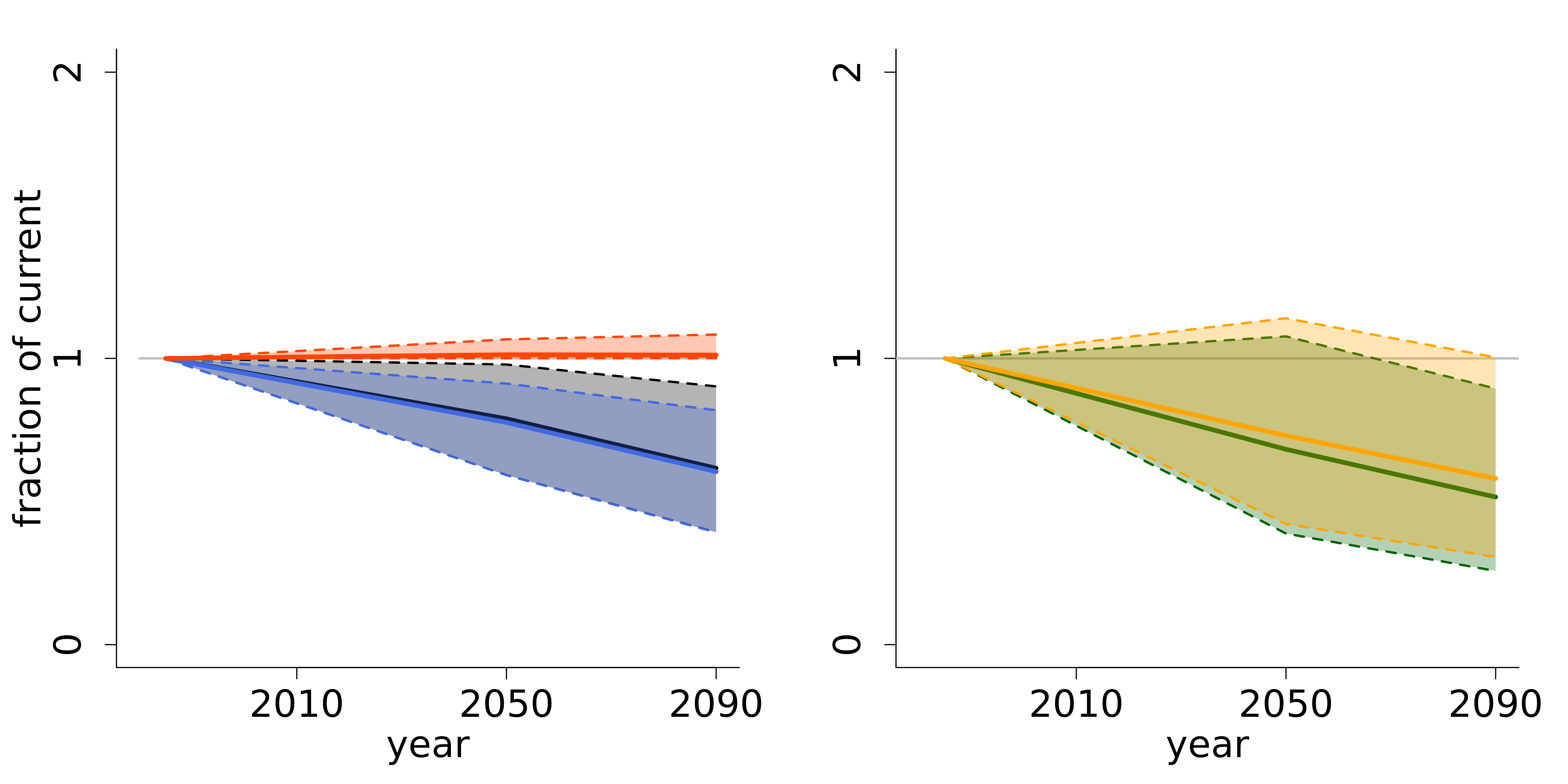

Supplement: S2 Appendix — (ZIP) [file pntd.0014030.s006.zip › Sup. Mat. 6-1 A-L - Species Trends/Bothrops_leucurus_CCTrends.png]

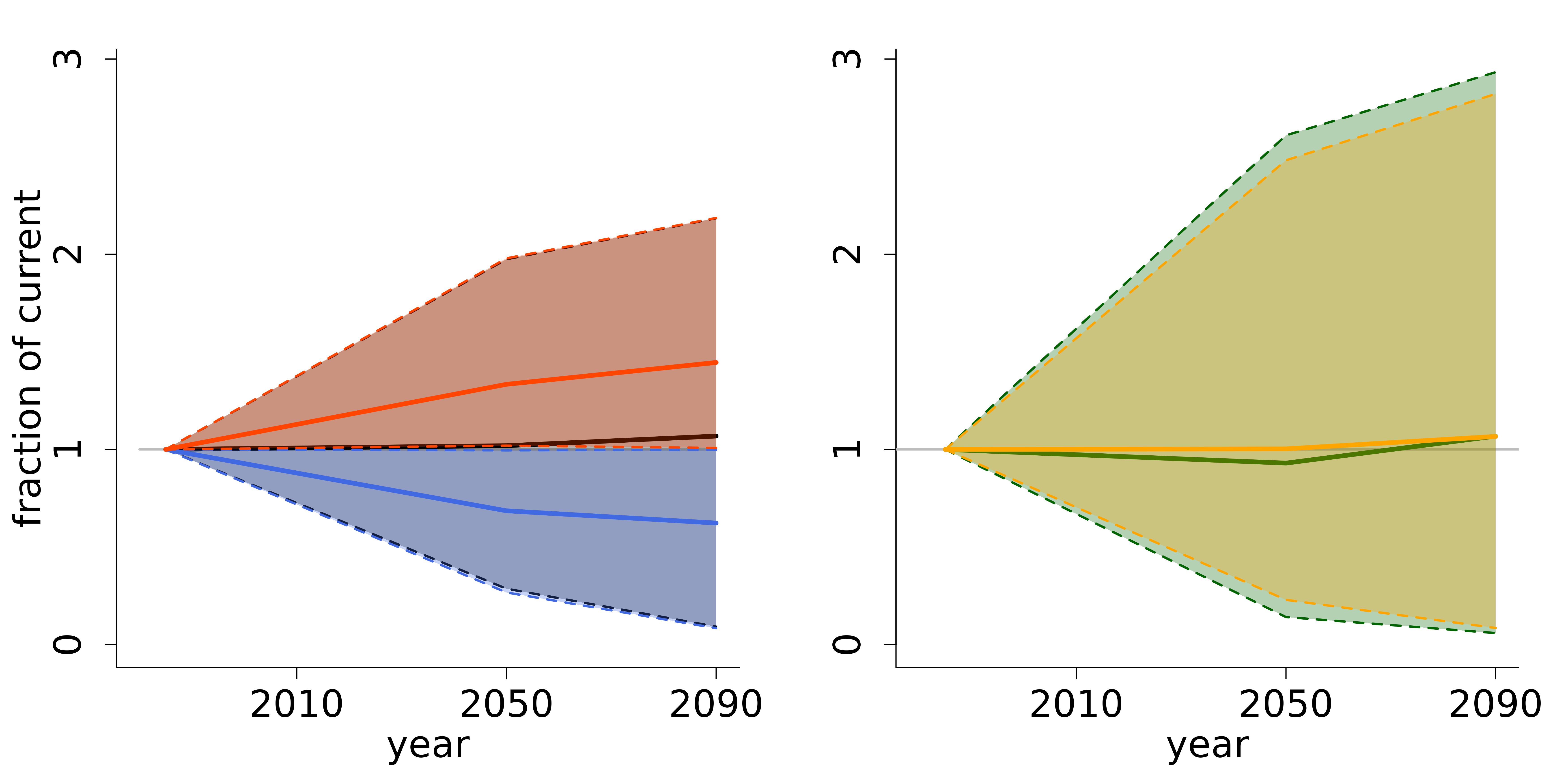

Supplement: S2 Appendix — (ZIP) [file pntd.0014030.s006.zip › Sup. Mat. 6-1 A-L - Species Trends/Bothrops_lutzi_CCTrends.png]

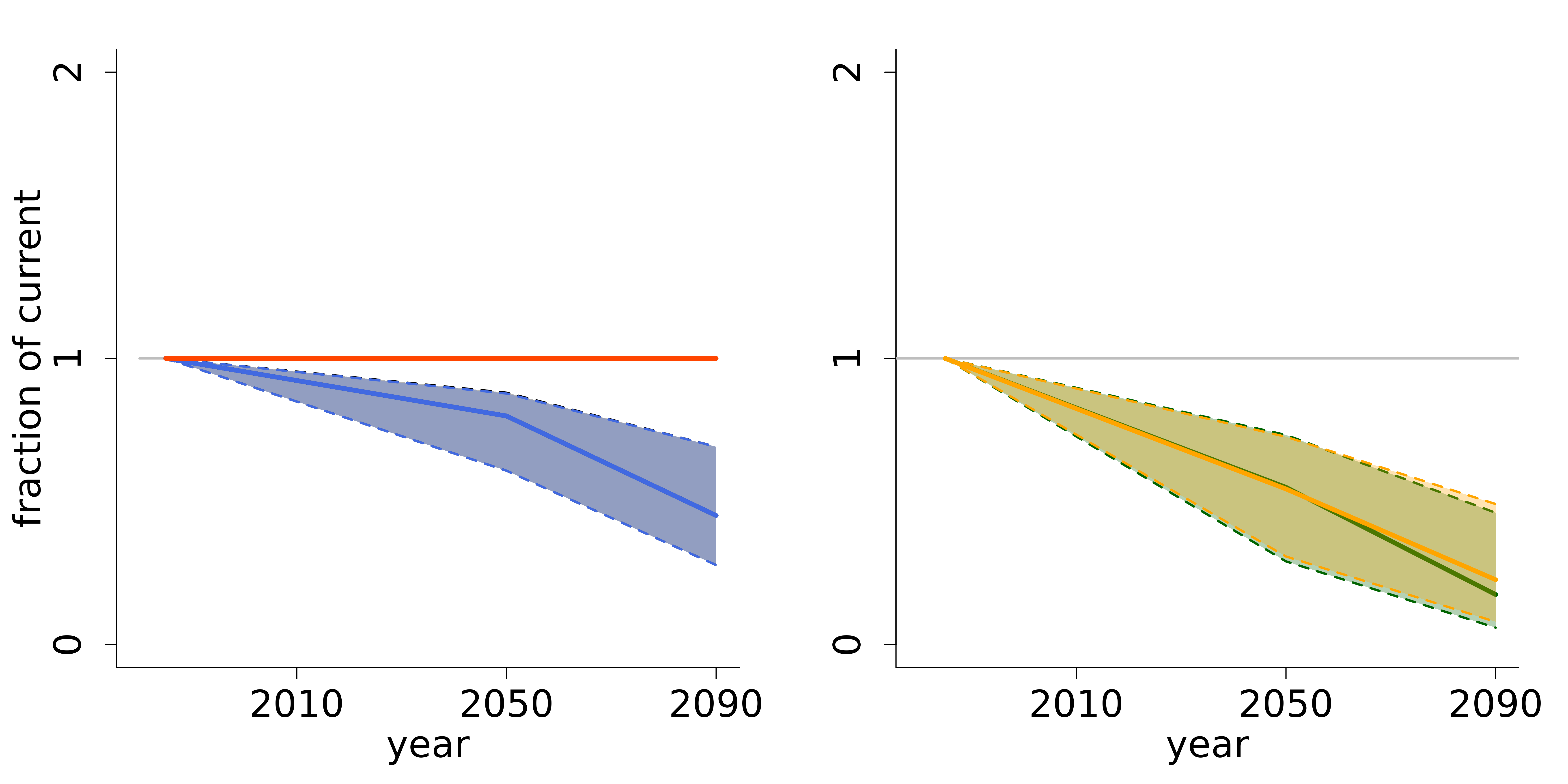

Supplement: S2 Appendix — (ZIP) [file pntd.0014030.s006.zip › Sup. Mat. 6-1 A-L - Species Trends/Bothrops_marmoratus_CCTrends.png]

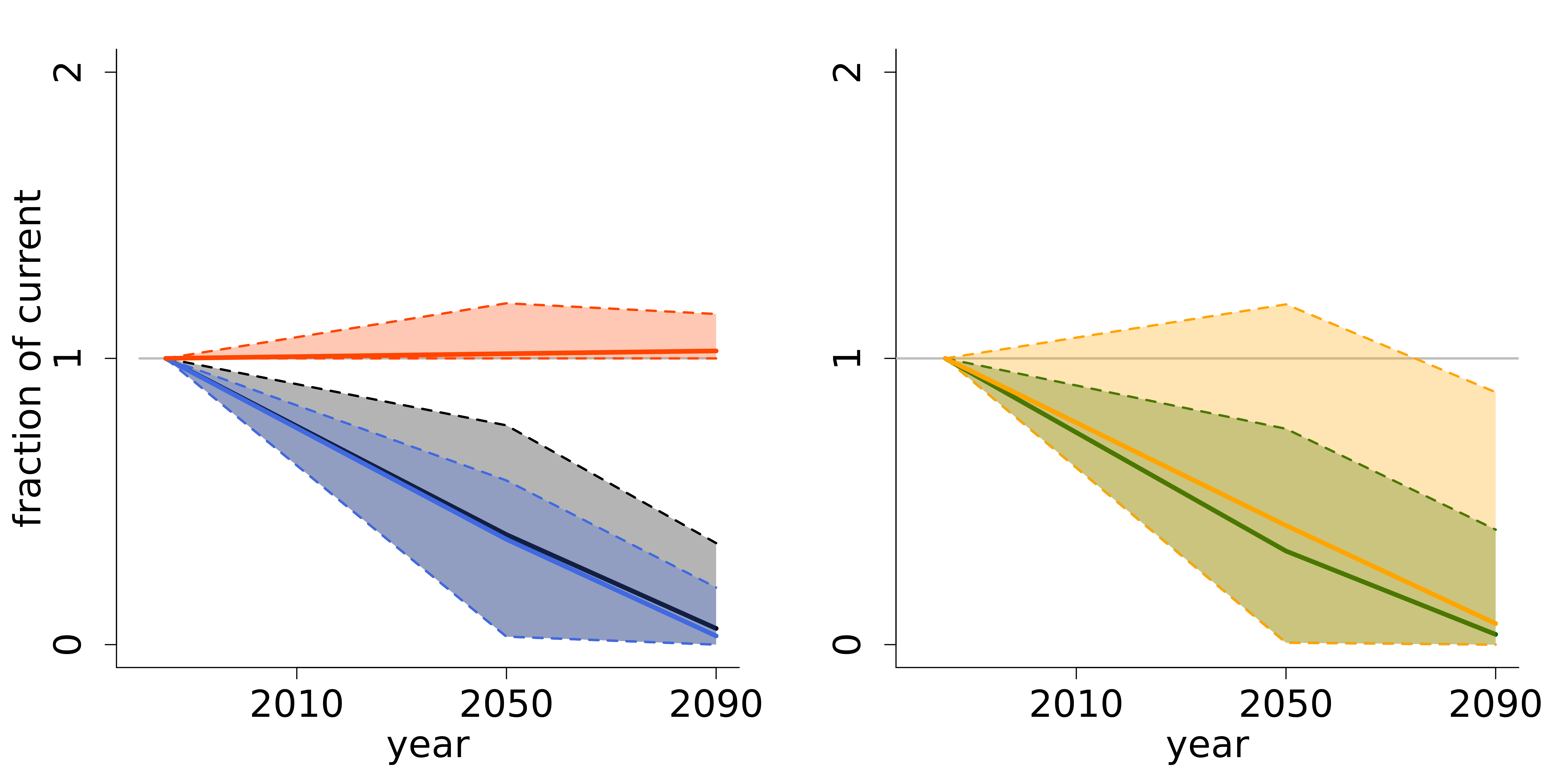

Supplement: S2 Appendix — (ZIP) [file pntd.0014030.s006.zip › Sup. Mat. 6-1 A-L - Species Trends/Bothrops_mattogrossensis_CCTrends.png]

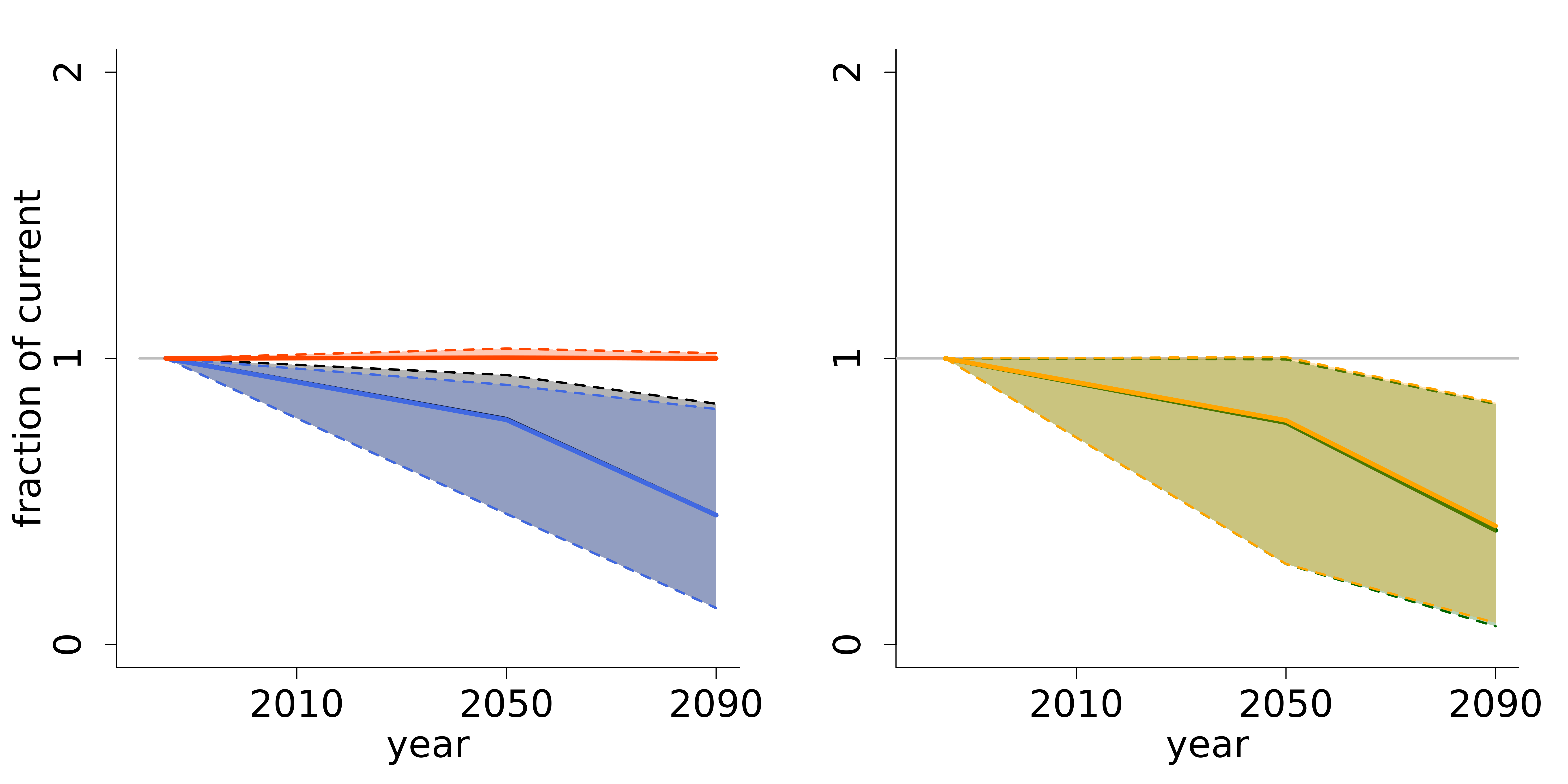

Supplement: S2 Appendix — (ZIP) [file pntd.0014030.s006.zip › Sup. Mat. 6-1 A-L - Species Trends/Bothrops_medusa_CCTrends.png]

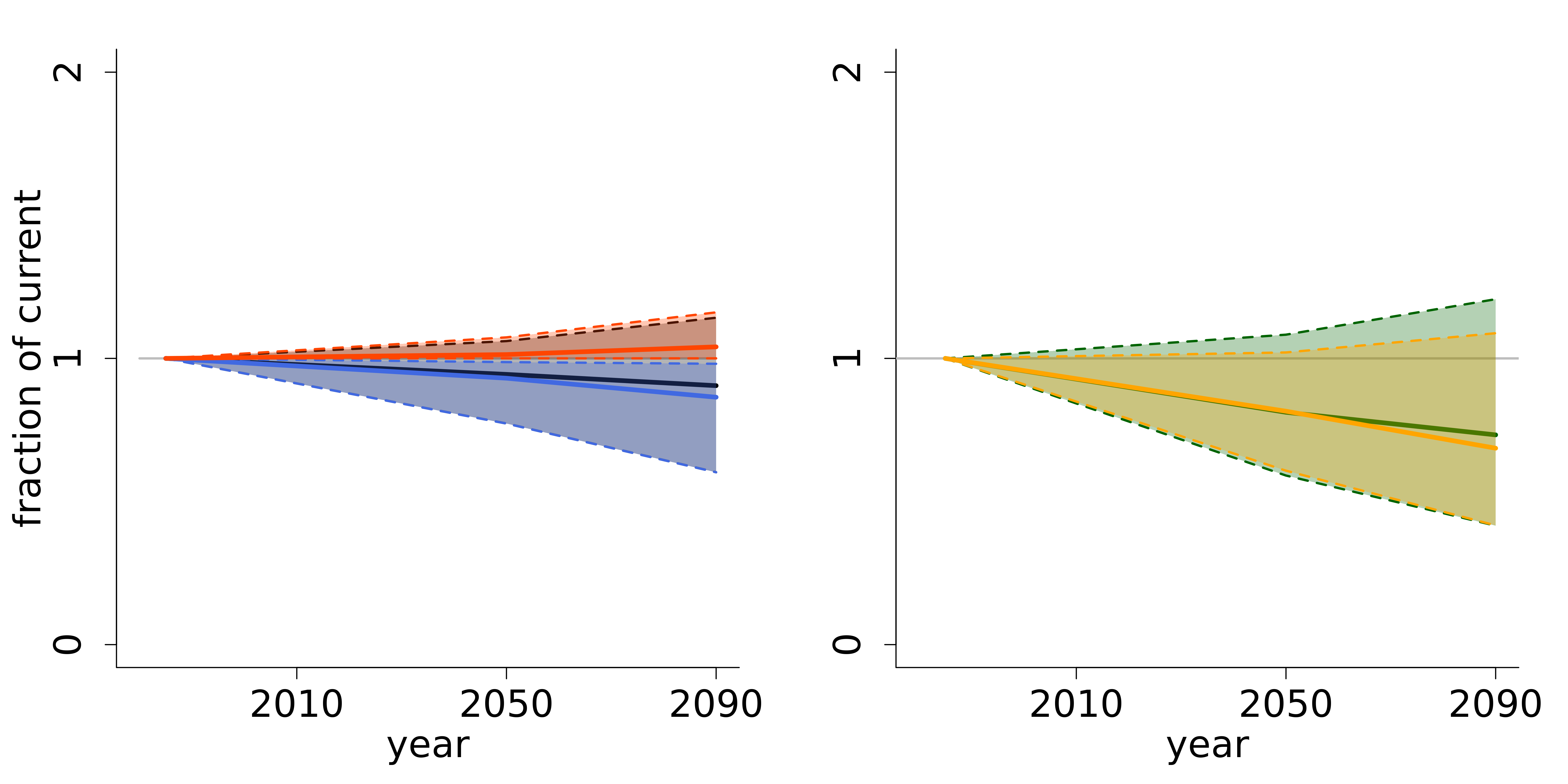

Supplement: S2 Appendix — (ZIP) [file pntd.0014030.s006.zip › Sup. Mat. 6-1 A-L - Species Trends/Bothrops_monsignifer_CCTrends.png]

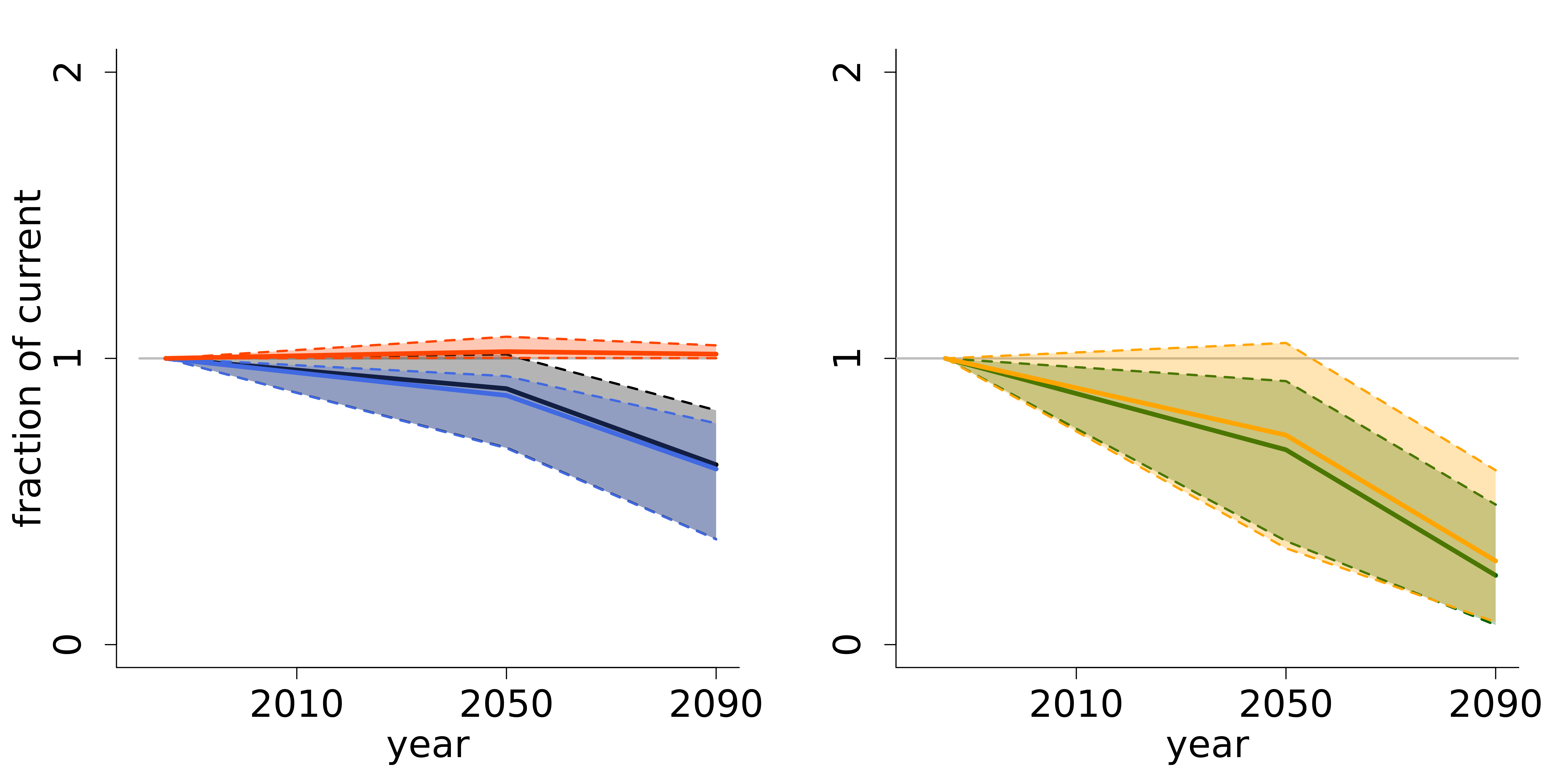

Supplement: S2 Appendix — (ZIP) [file pntd.0014030.s006.zip › Sup. Mat. 6-1 A-L - Species Trends/Bothrops_moojeni_CCTrends.png]

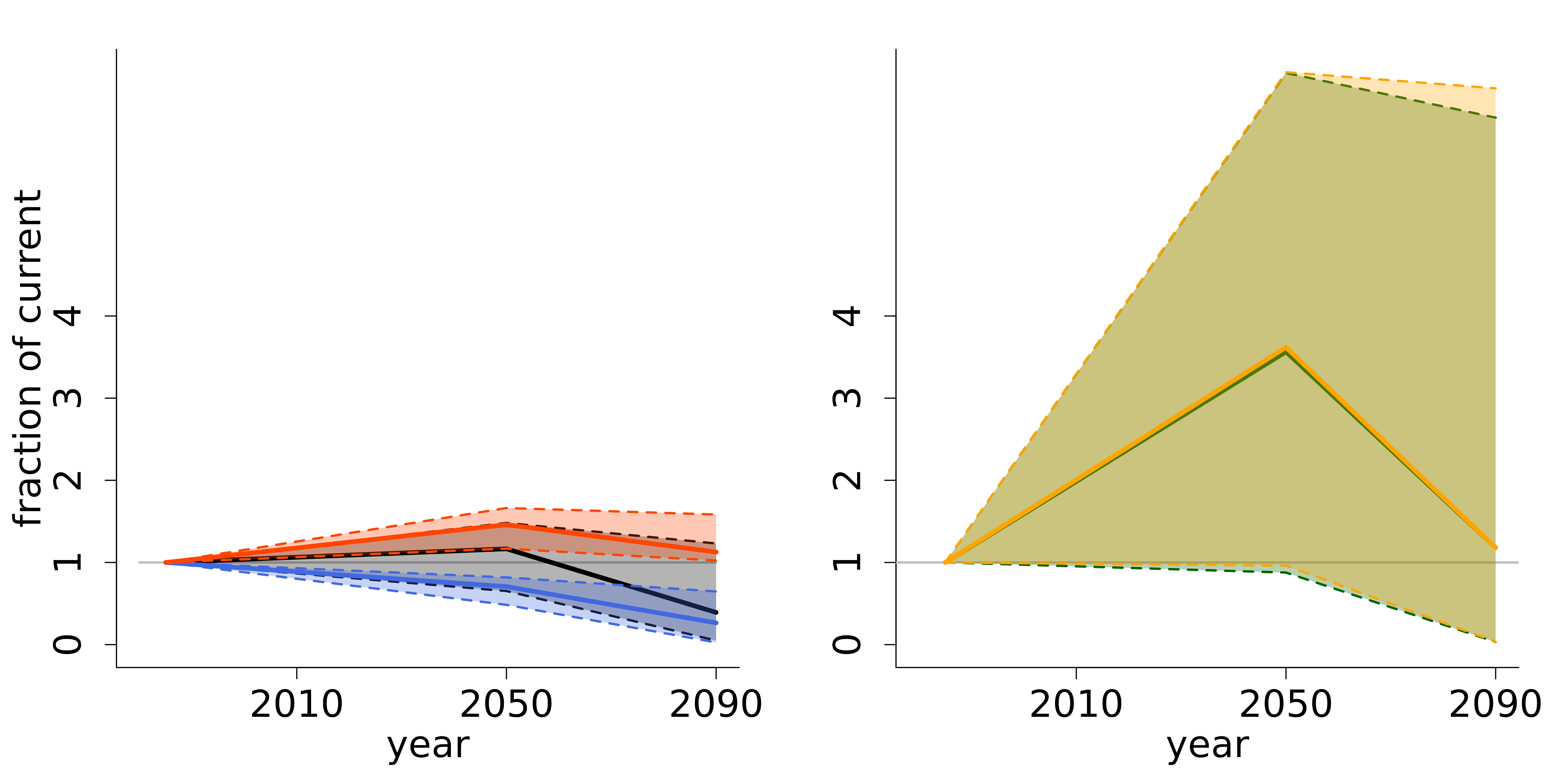

Supplement: S2 Appendix — (ZIP) [file pntd.0014030.s006.zip › Sup. Mat. 6-1 A-L - Species Trends/Bothrops_muriciensis_CCTrends.png]

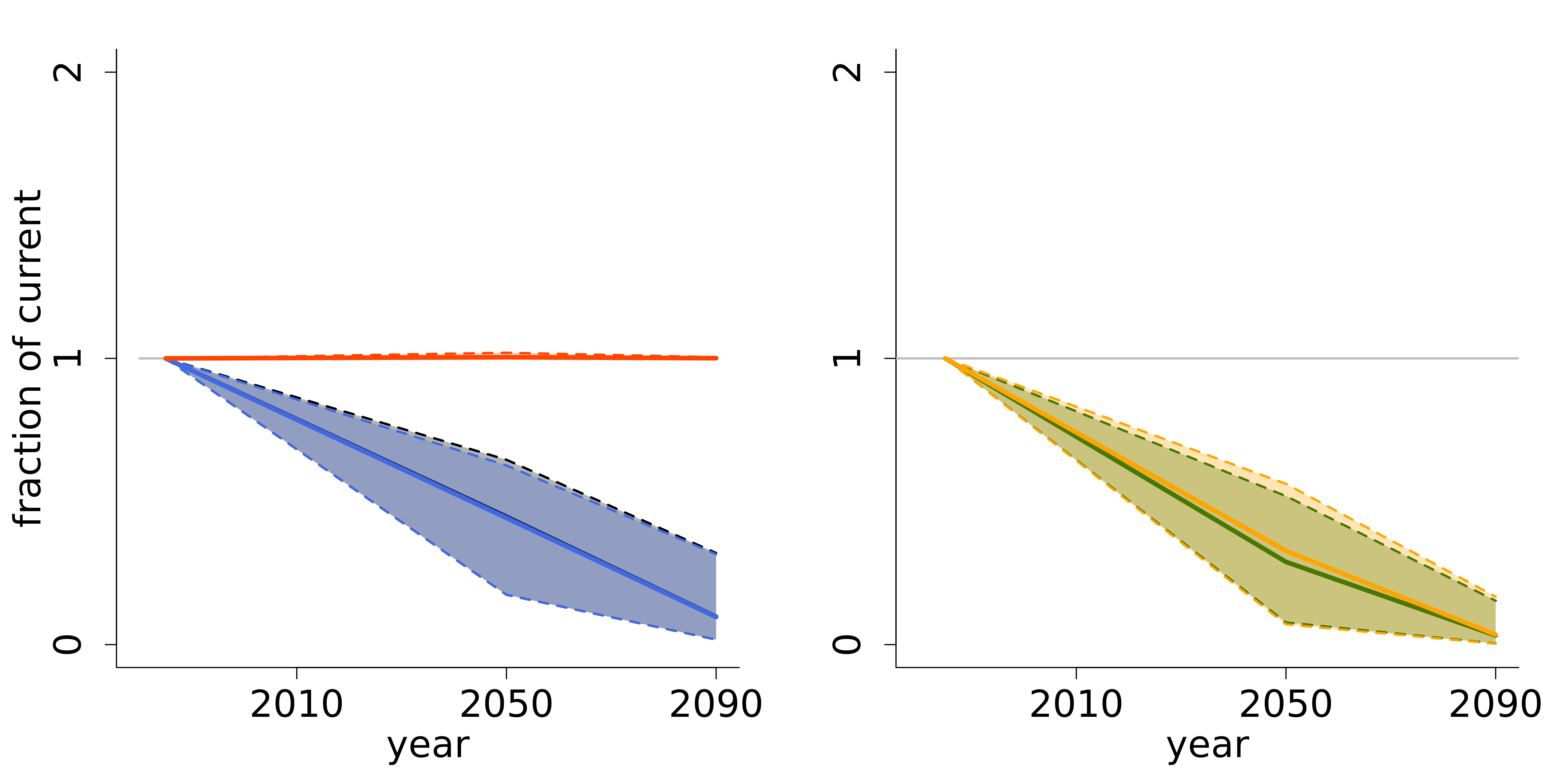

Supplement: S2 Appendix — (ZIP) [file pntd.0014030.s006.zip › Sup. Mat. 6-1 A-L - Species Trends/Bothrops_neuwiedi_CCTrends.png]

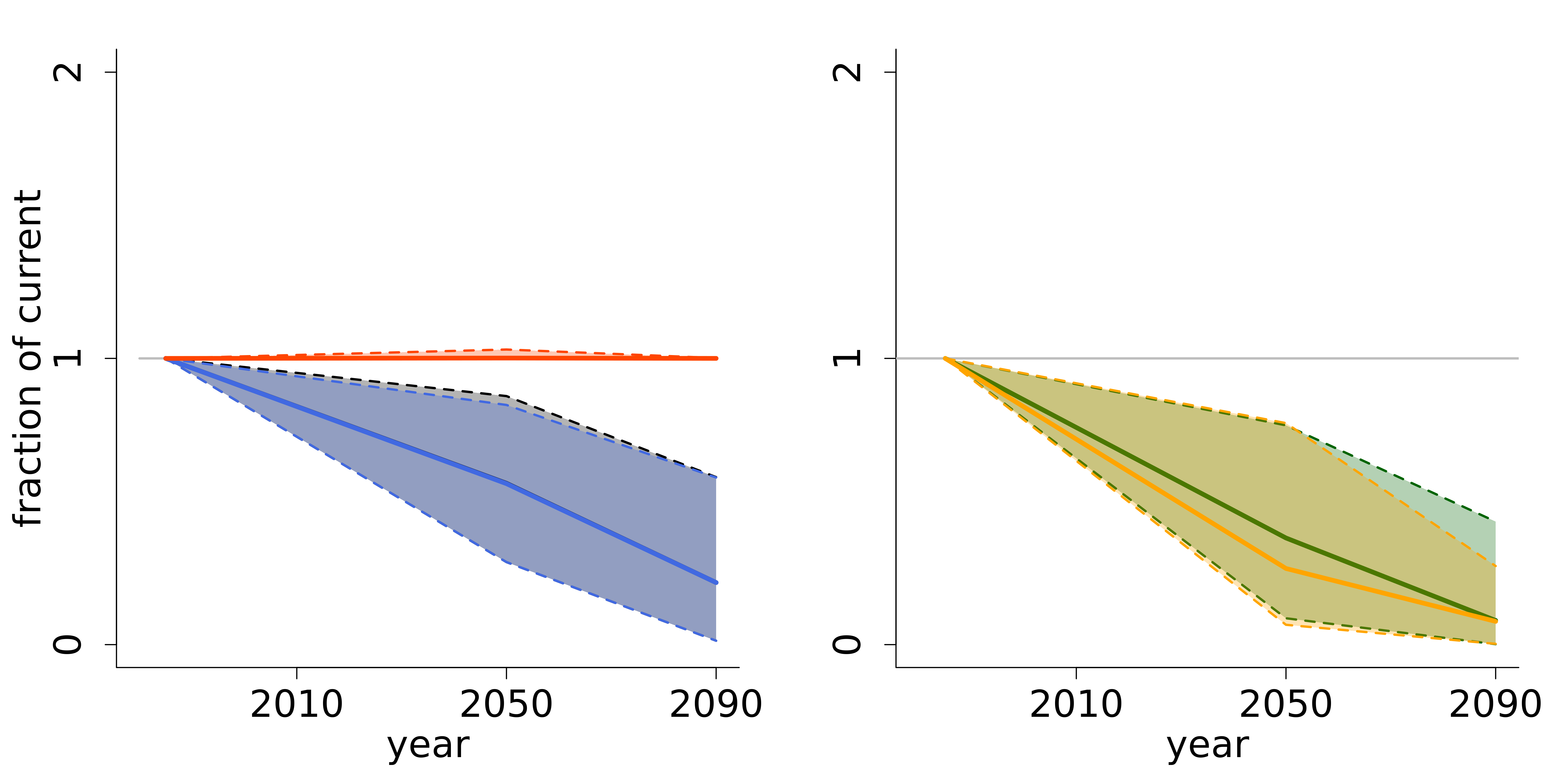

Supplement: S2 Appendix — (ZIP) [file pntd.0014030.s006.zip › Sup. Mat. 6-1 A-L - Species Trends/Bothrops_oligobalius_CCTrends.png]

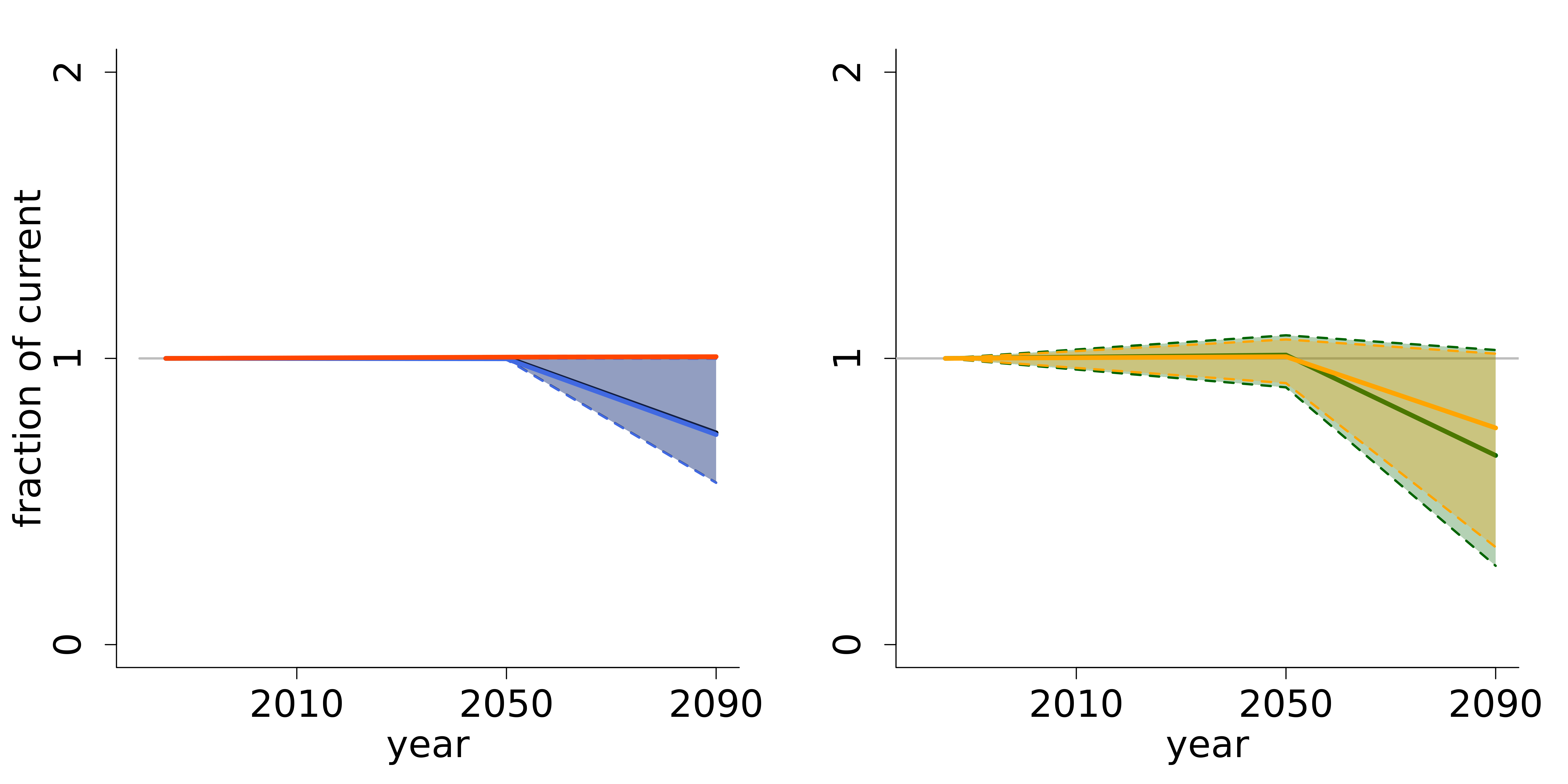

Supplement: S2 Appendix — (ZIP) [file pntd.0014030.s006.zip › Sup. Mat. 6-1 A-L - Species Trends/Bothrops_oligolepis_CCTrends.png]

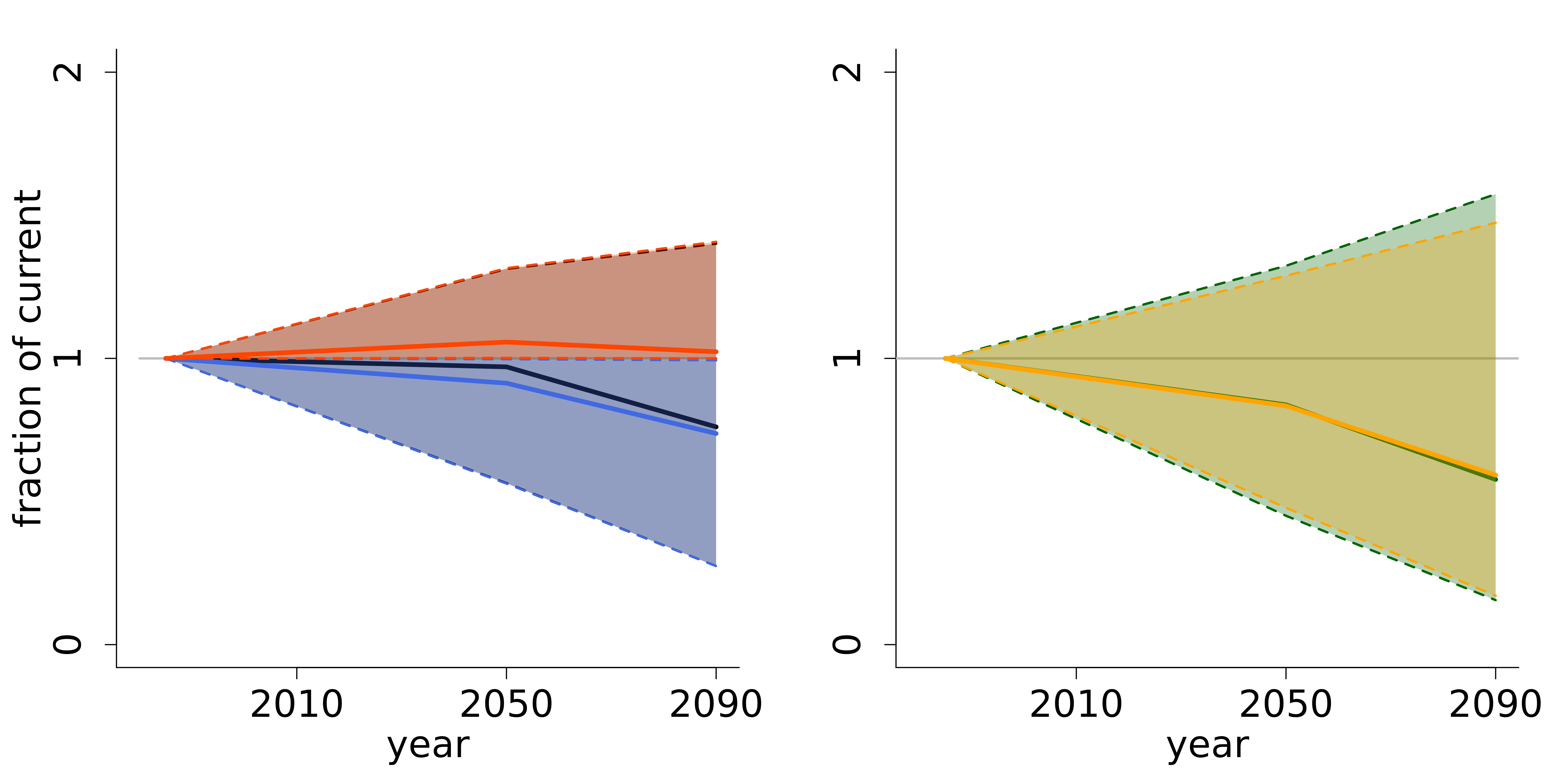

Supplement: S2 Appendix — (ZIP) [file pntd.0014030.s006.zip › Sup. Mat. 6-1 A-L - Species Trends/Bothrops_osbornei_CCTrends.png]
