# Supplementary figures and images for: Climate change induced complex shifts in snake distributions expose people to snakebite and threaten biodiversity (part 4 of 4)
Source: PLoS Negl Trop Dis. 2026 May 21;20(5):e0014030. doi: 10.1371/journal.pntd.0014030 (PMC13193456; doi:10.1371/journal.pntd.0014030)

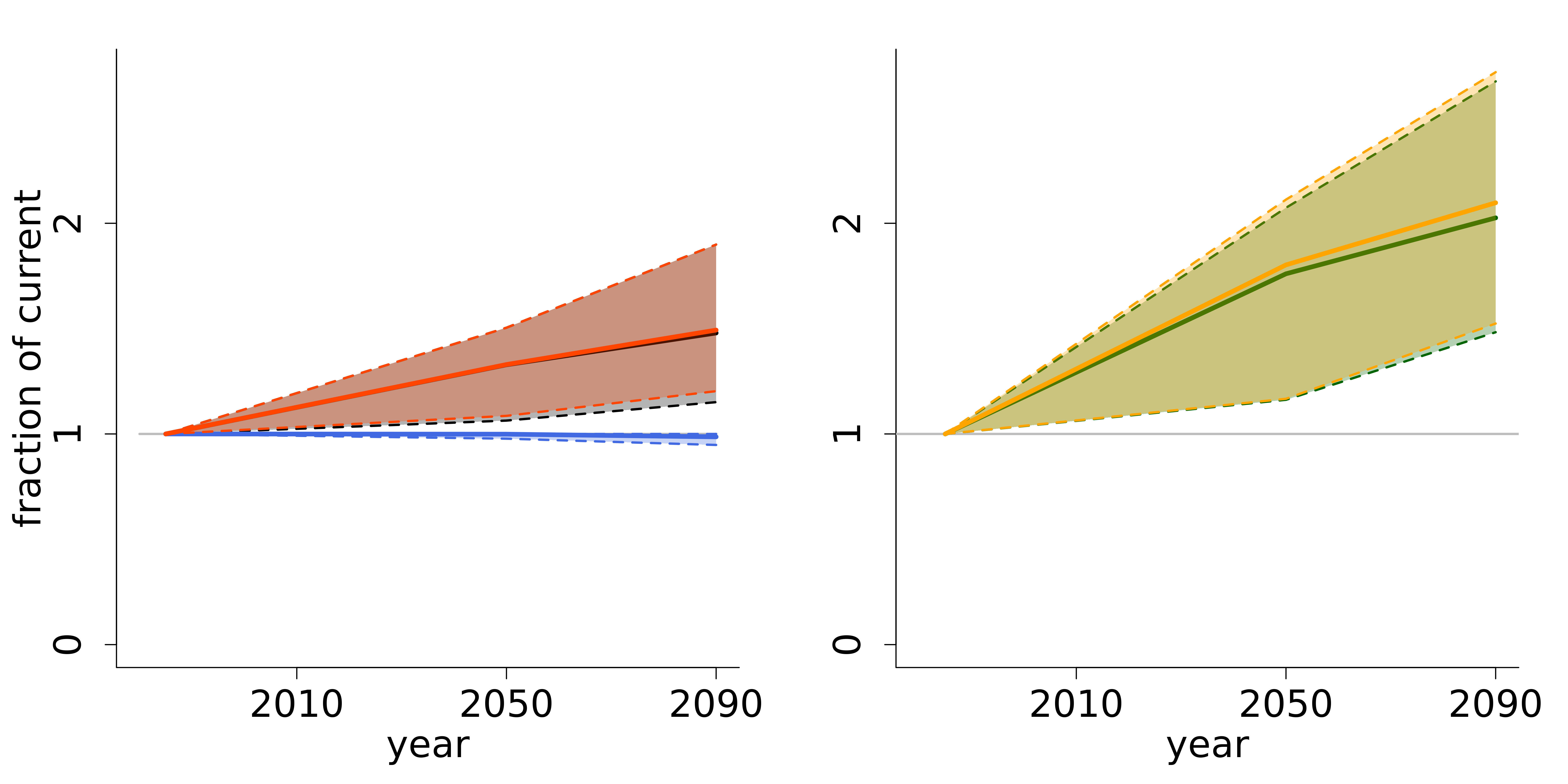

Supplement: S3 Appendix — (ZIP) [file pntd.0014030.s007.zip › Sup. Mat. 6-2 M-Z - Species Trends/Naja_guineensis_CCTrends.png]

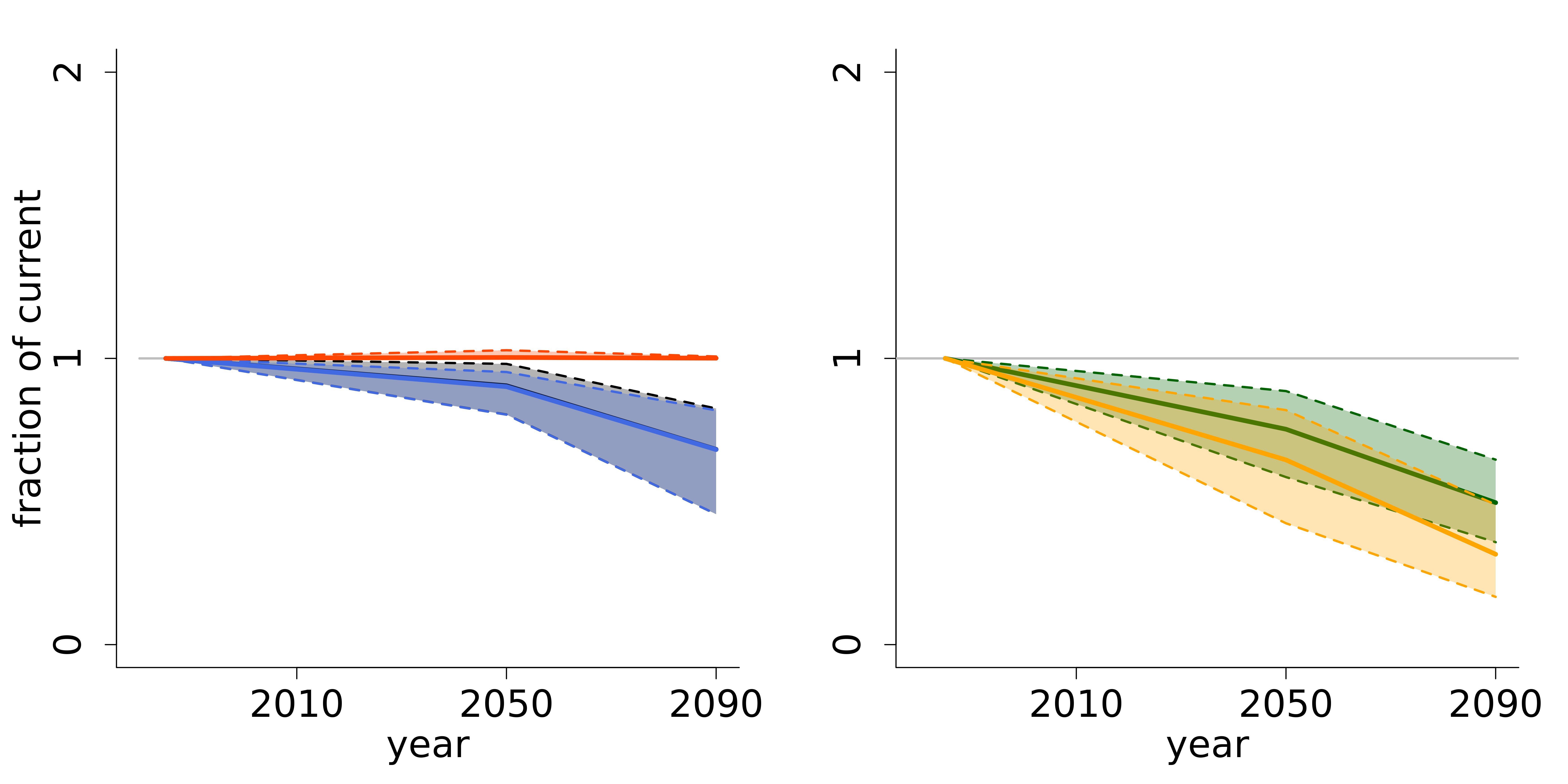

Supplement: S3 Appendix — (ZIP) [file pntd.0014030.s007.zip › Sup. Mat. 6-2 M-Z - Species Trends/Naja_haje_CCTrends.png]

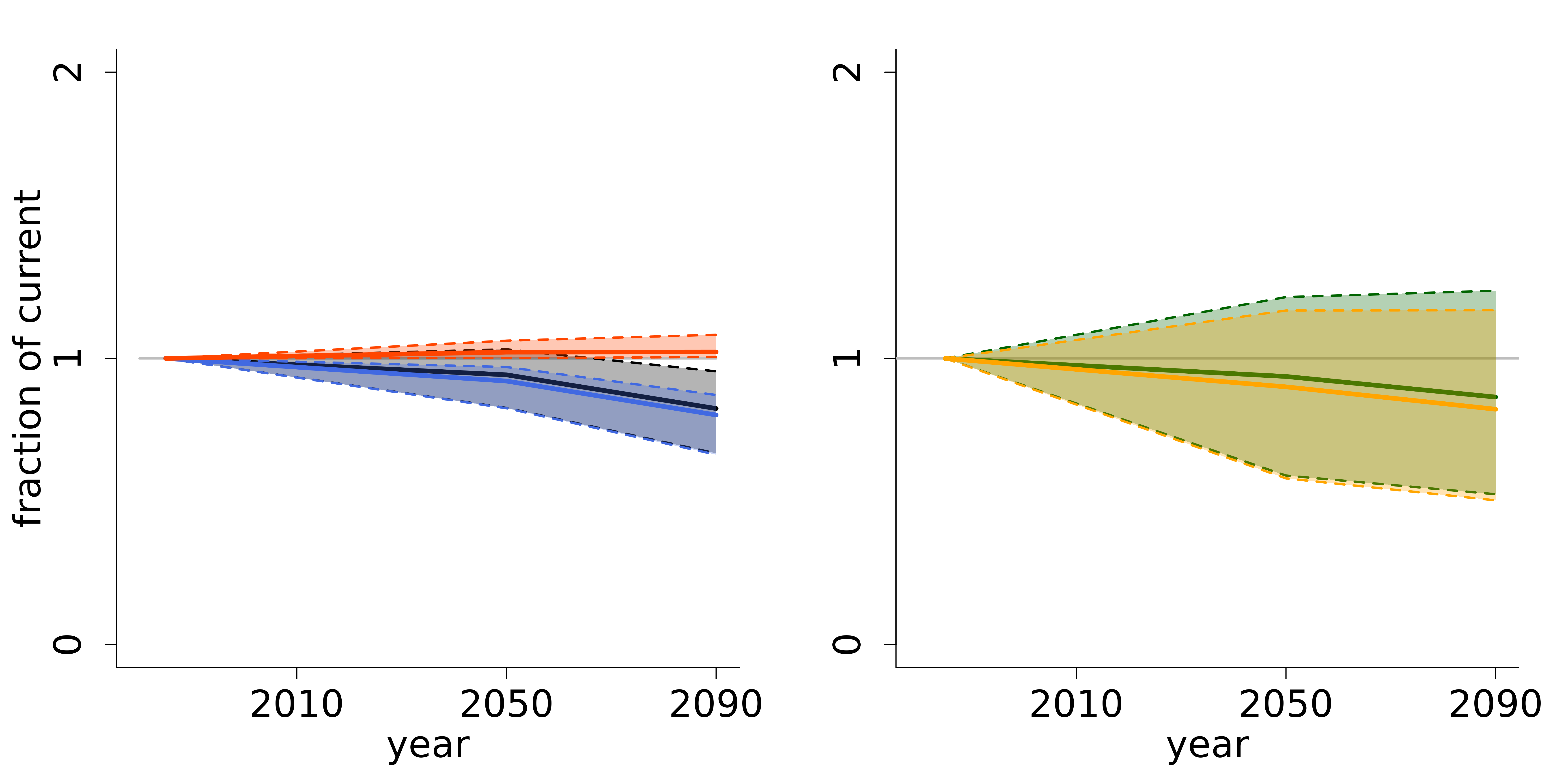

Supplement: S3 Appendix — (ZIP) [file pntd.0014030.s007.zip › Sup. Mat. 6-2 M-Z - Species Trends/Naja_kaouthia_CCTrends.png]

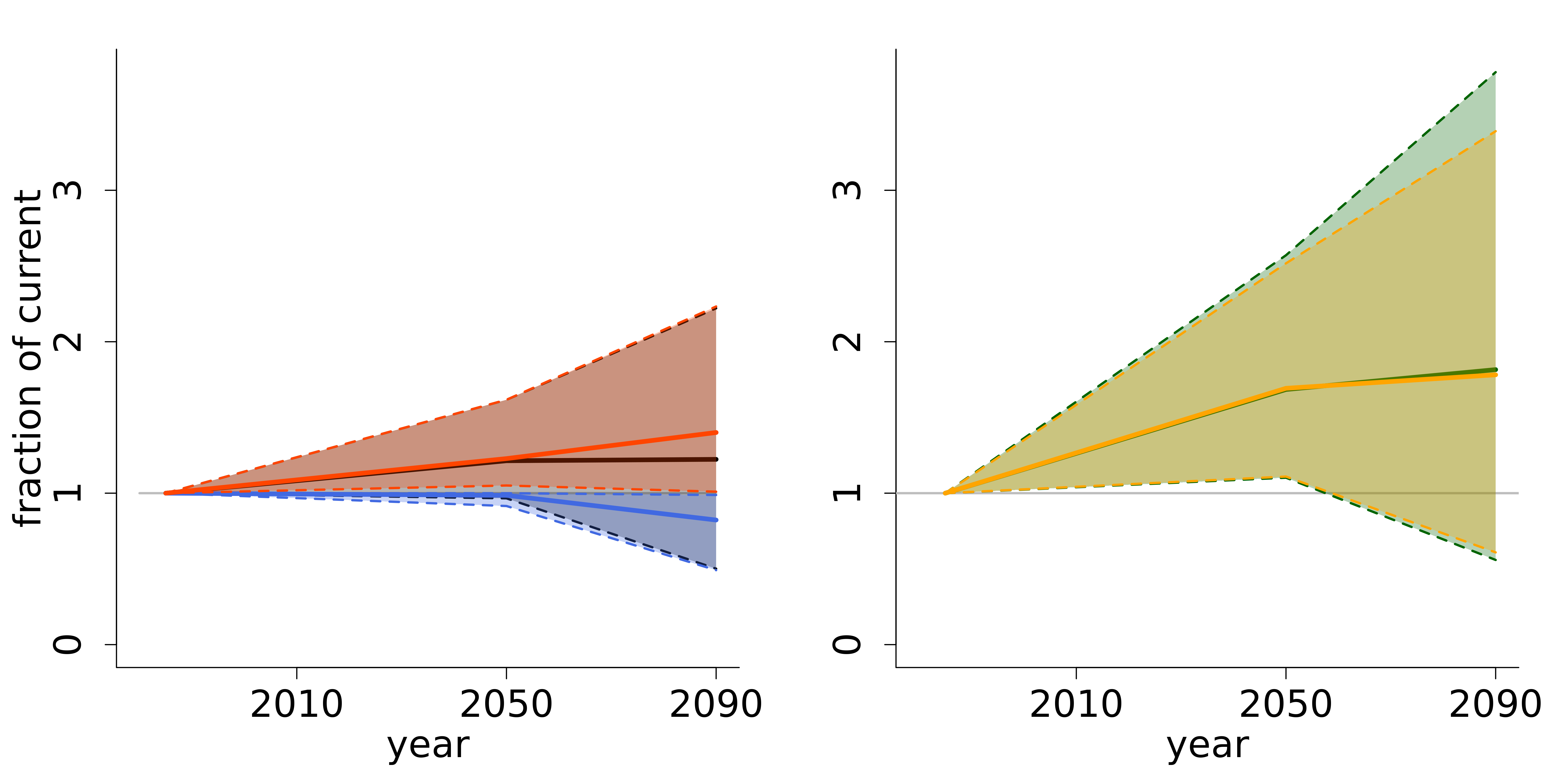

Supplement: S3 Appendix — (ZIP) [file pntd.0014030.s007.zip › Sup. Mat. 6-2 M-Z - Species Trends/Naja_katiensis_CCTrends.png]

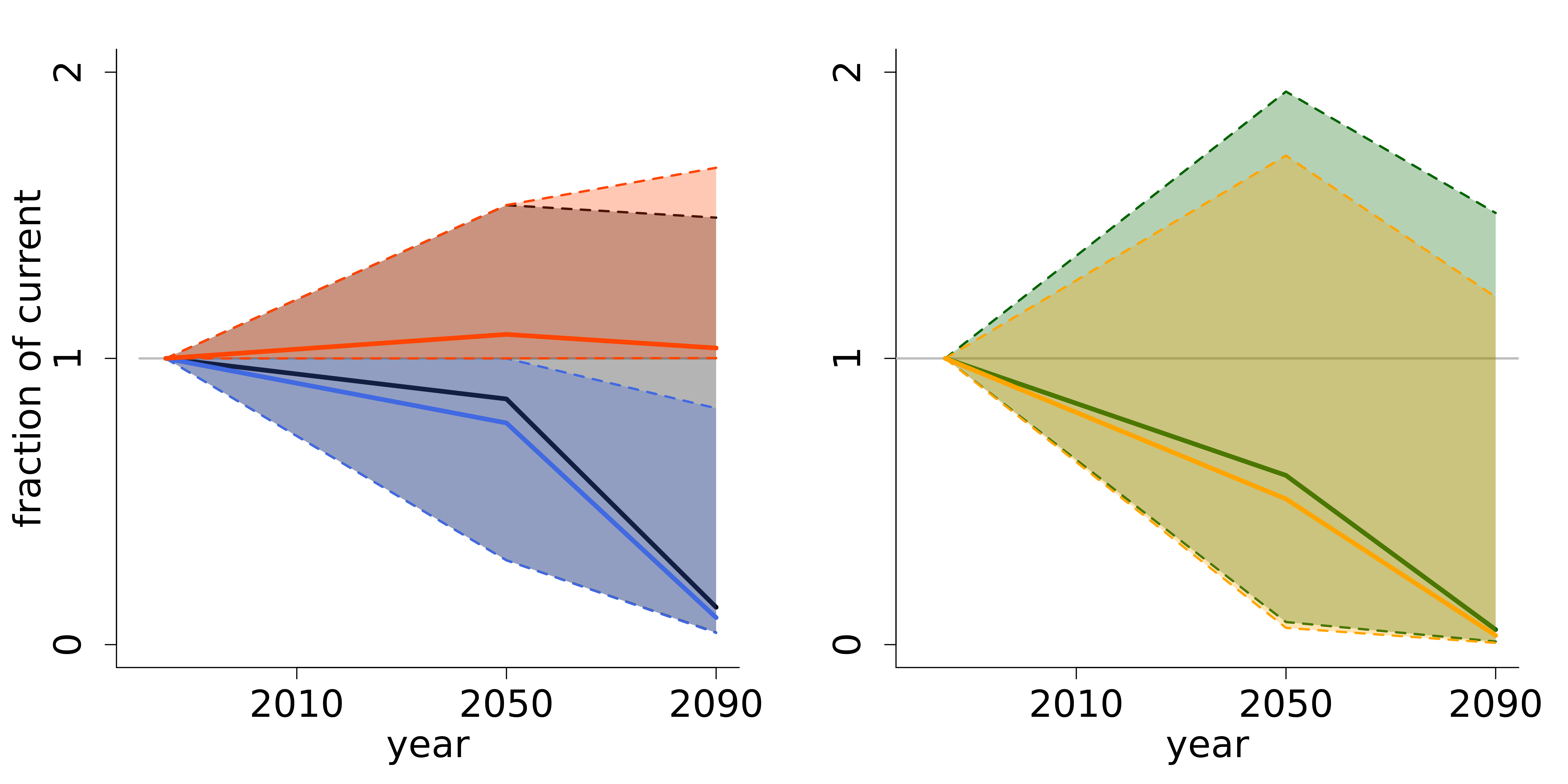

Supplement: S3 Appendix — (ZIP) [file pntd.0014030.s007.zip › Sup. Mat. 6-2 M-Z - Species Trends/Naja_mandalayensis_CCTrends.png]

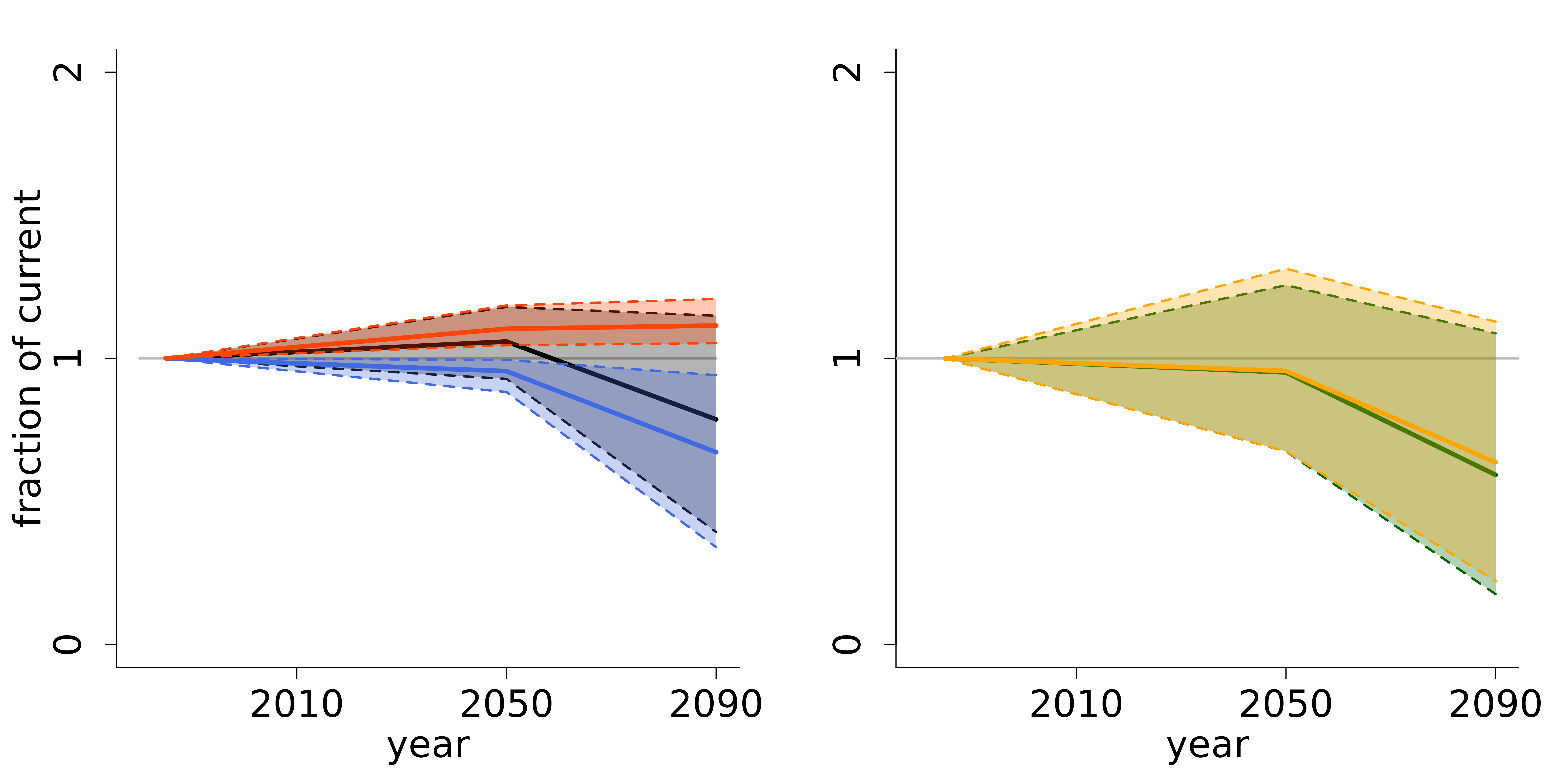

Supplement: S3 Appendix — (ZIP) [file pntd.0014030.s007.zip › Sup. Mat. 6-2 M-Z - Species Trends/Naja_melanoleuca_CCTrends.png]

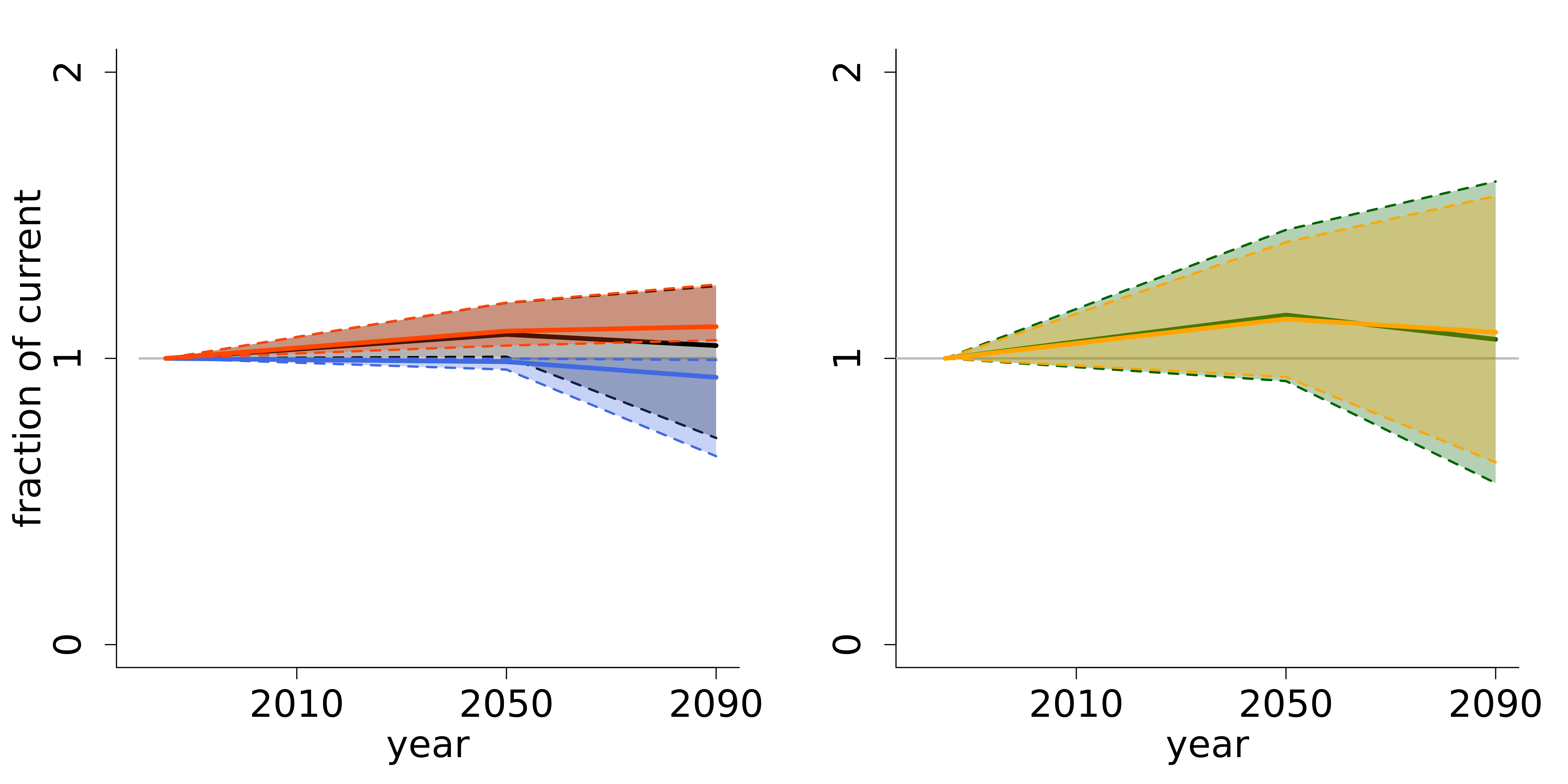

Supplement: S3 Appendix — (ZIP) [file pntd.0014030.s007.zip › Sup. Mat. 6-2 M-Z - Species Trends/Naja_mossambica_CCTrends.png]

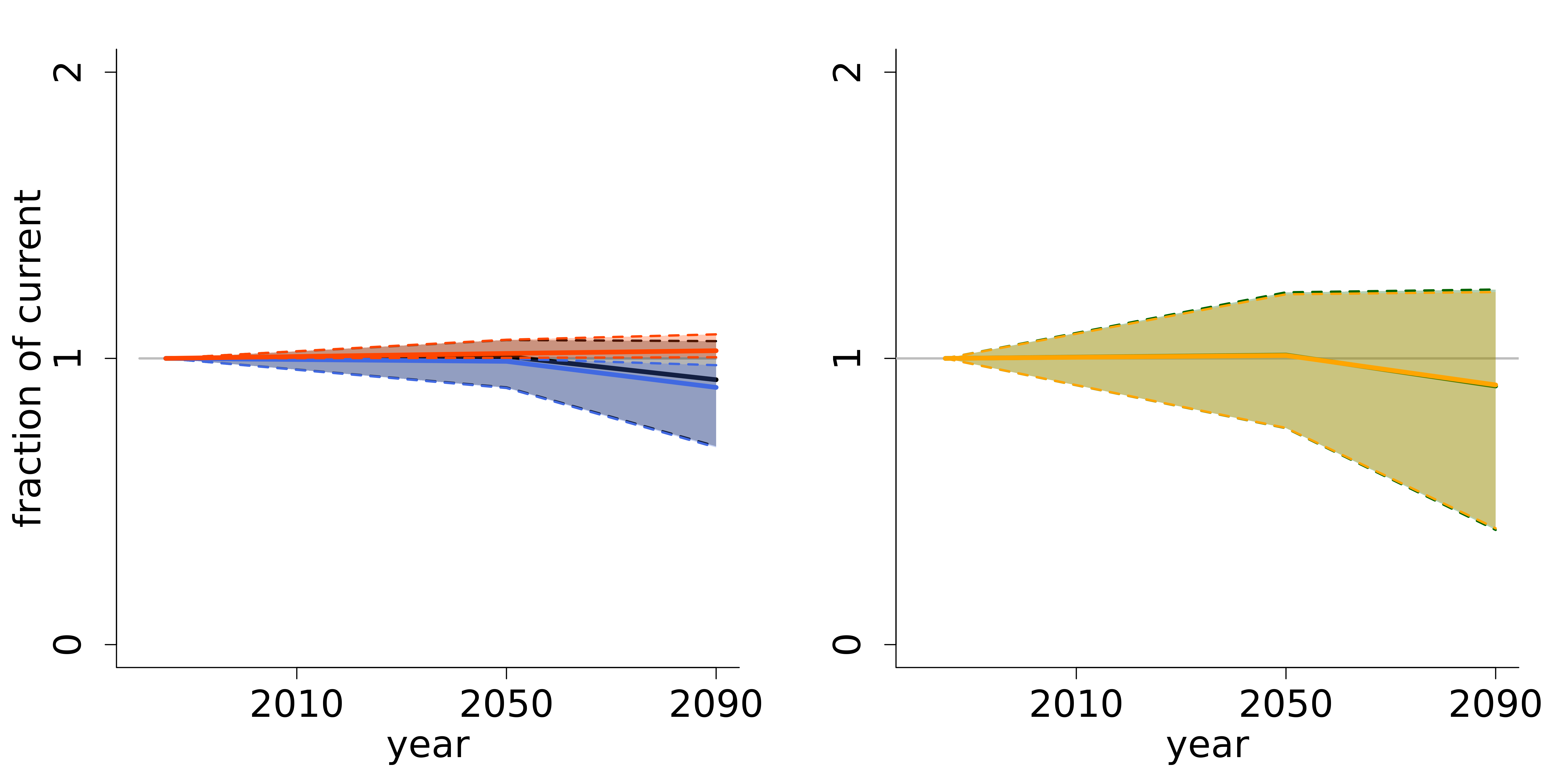

Supplement: S3 Appendix — (ZIP) [file pntd.0014030.s007.zip › Sup. Mat. 6-2 M-Z - Species Trends/Naja_naja_CCTrends.png]

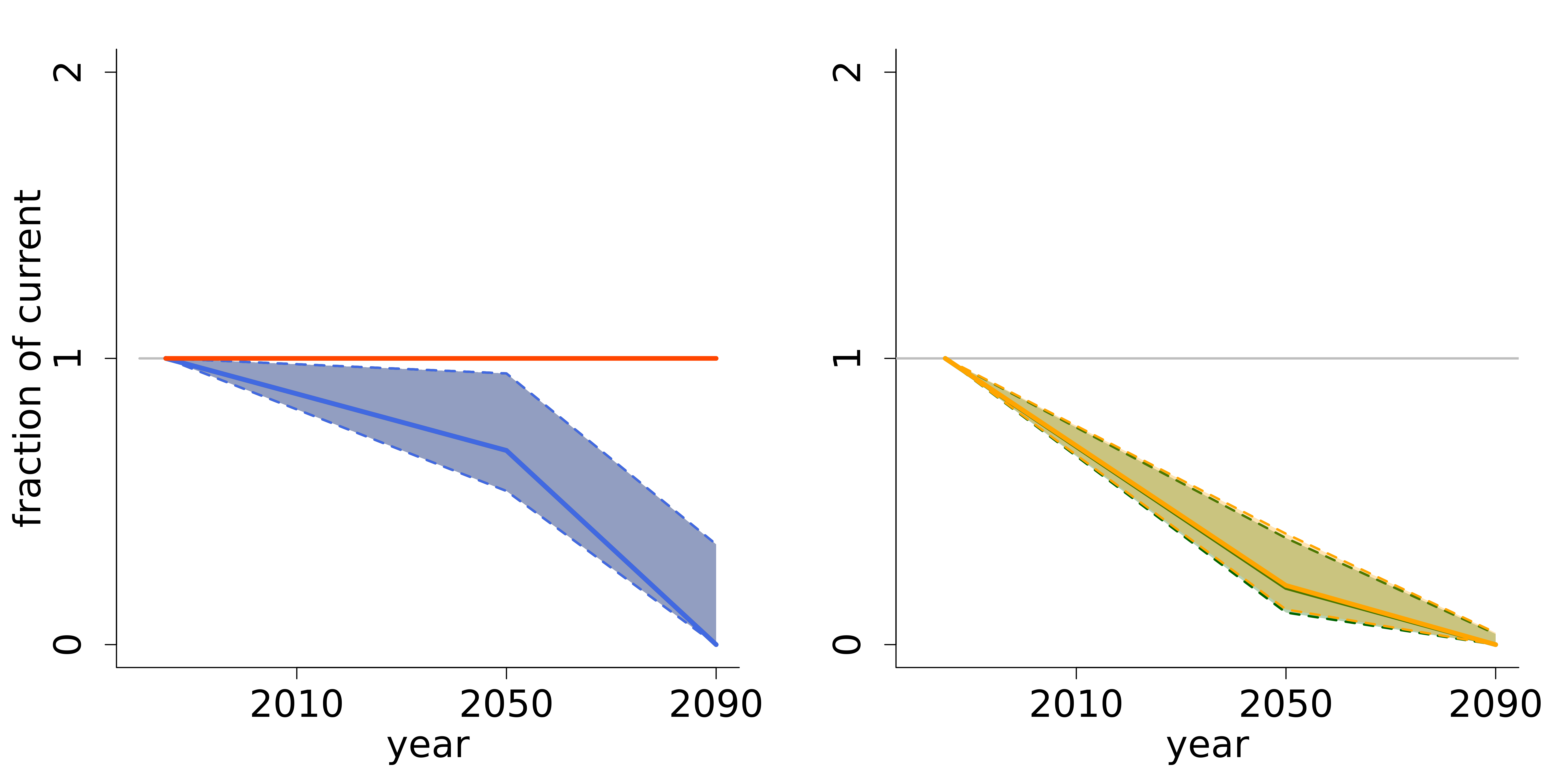

Supplement: S3 Appendix — (ZIP) [file pntd.0014030.s007.zip › Sup. Mat. 6-2 M-Z - Species Trends/Naja_nana_CCTrends.png]

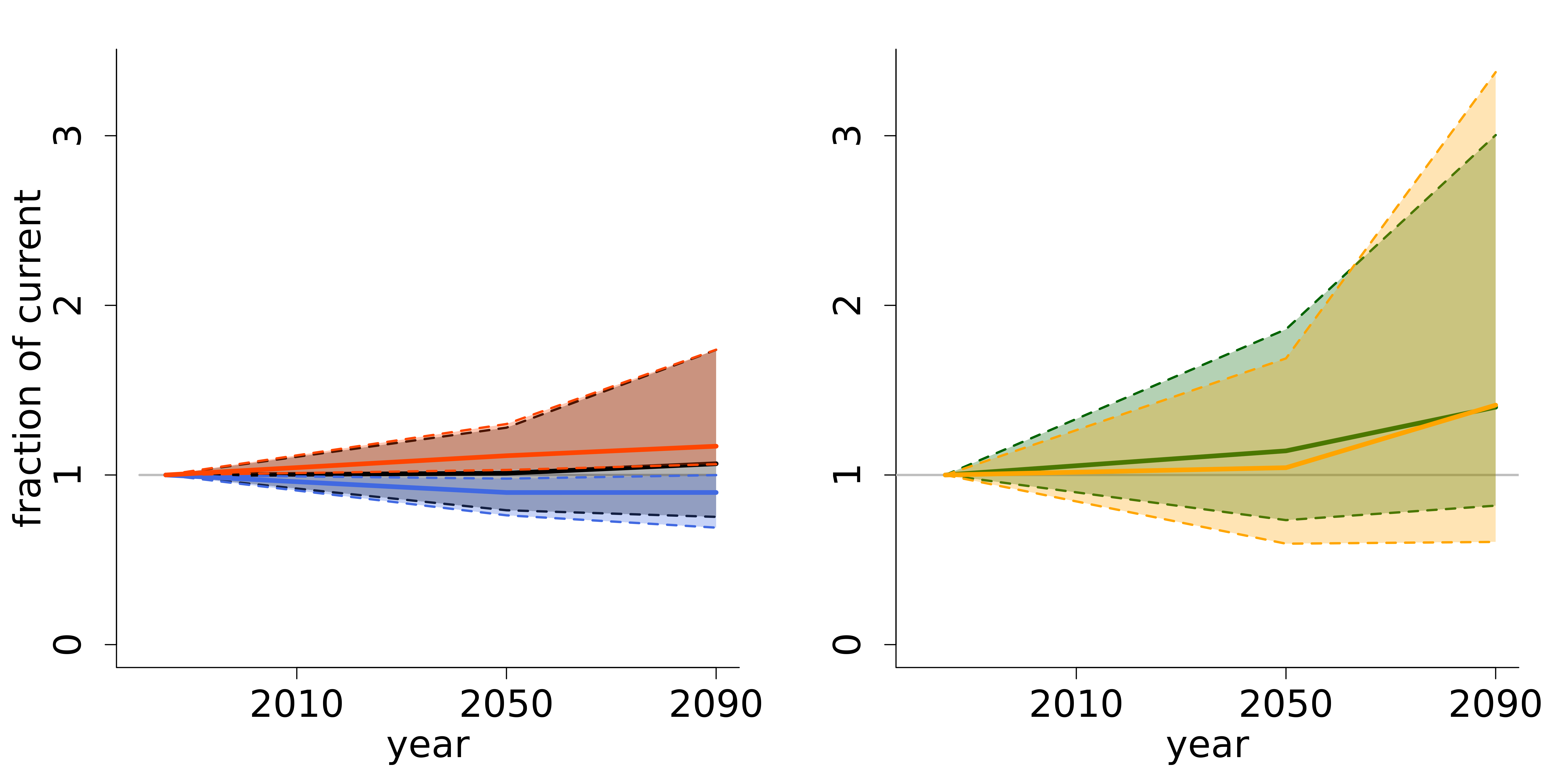

Supplement: S3 Appendix — (ZIP) [file pntd.0014030.s007.zip › Sup. Mat. 6-2 M-Z - Species Trends/Naja_nigricincta_CCTrends.png]

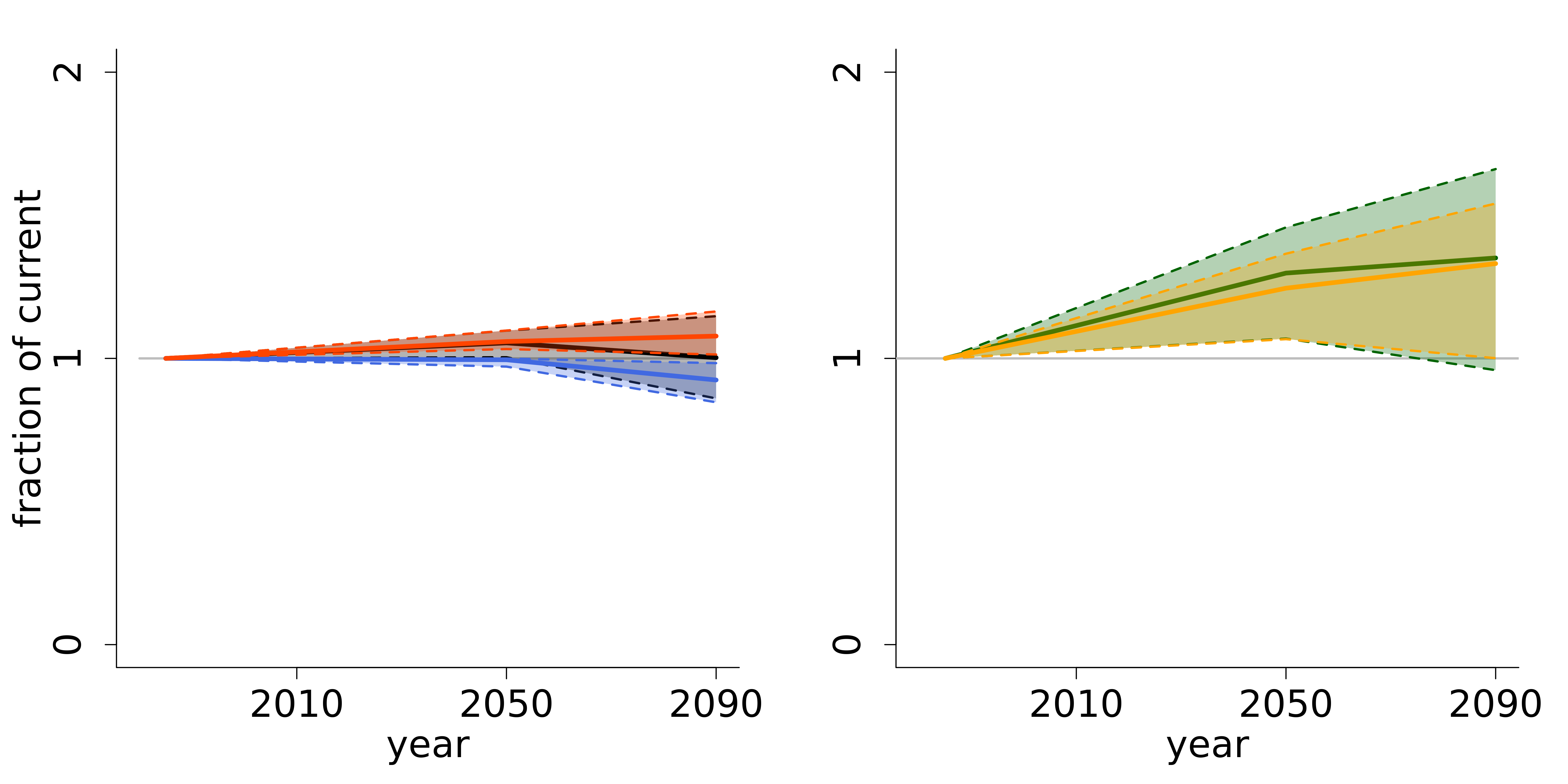

Supplement: S3 Appendix — (ZIP) [file pntd.0014030.s007.zip › Sup. Mat. 6-2 M-Z - Species Trends/Naja_nigricollis_CCTrends.png]

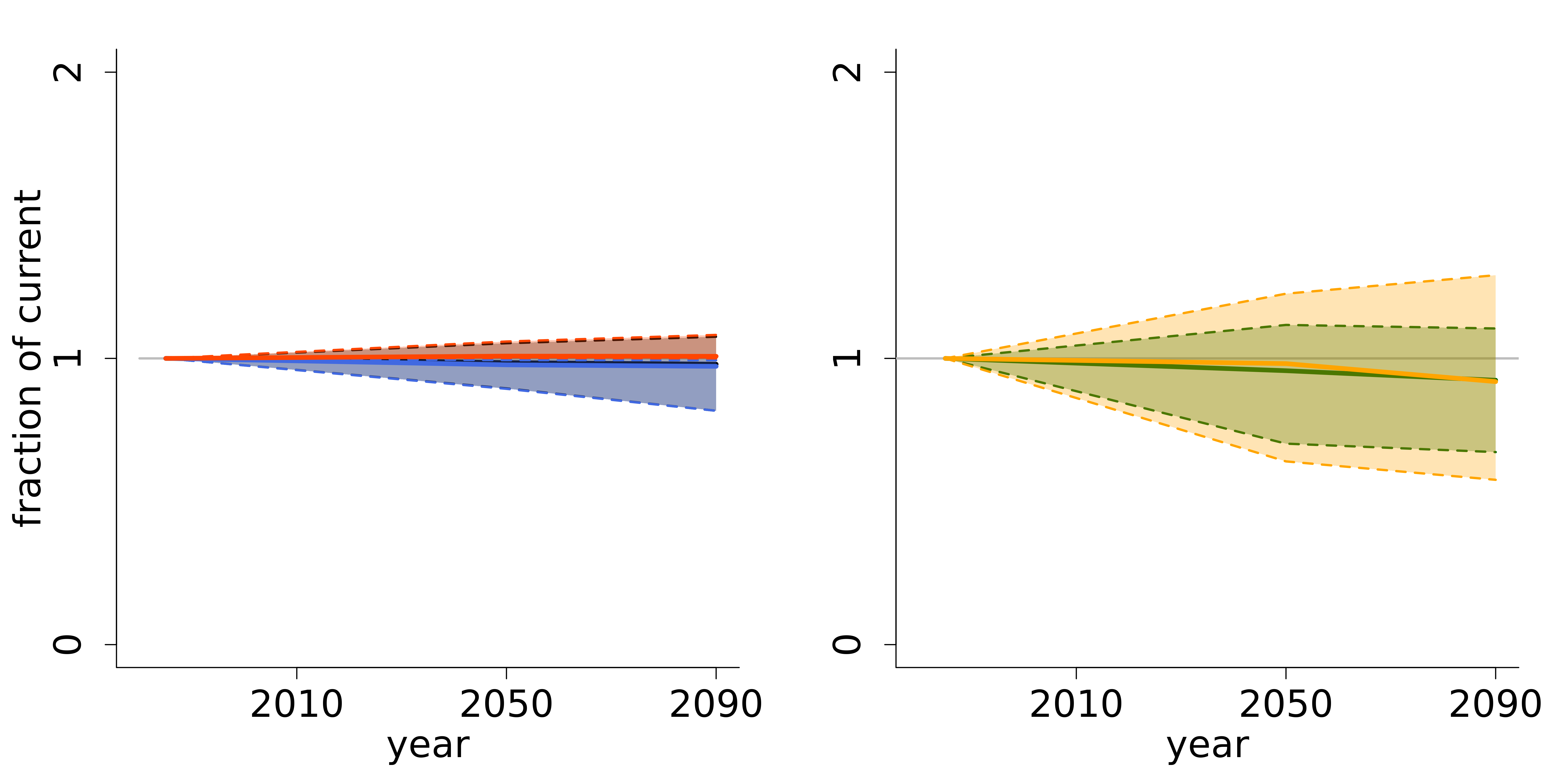

Supplement: S3 Appendix — (ZIP) [file pntd.0014030.s007.zip › Sup. Mat. 6-2 M-Z - Species Trends/Naja_nivea_CCTrends.png]

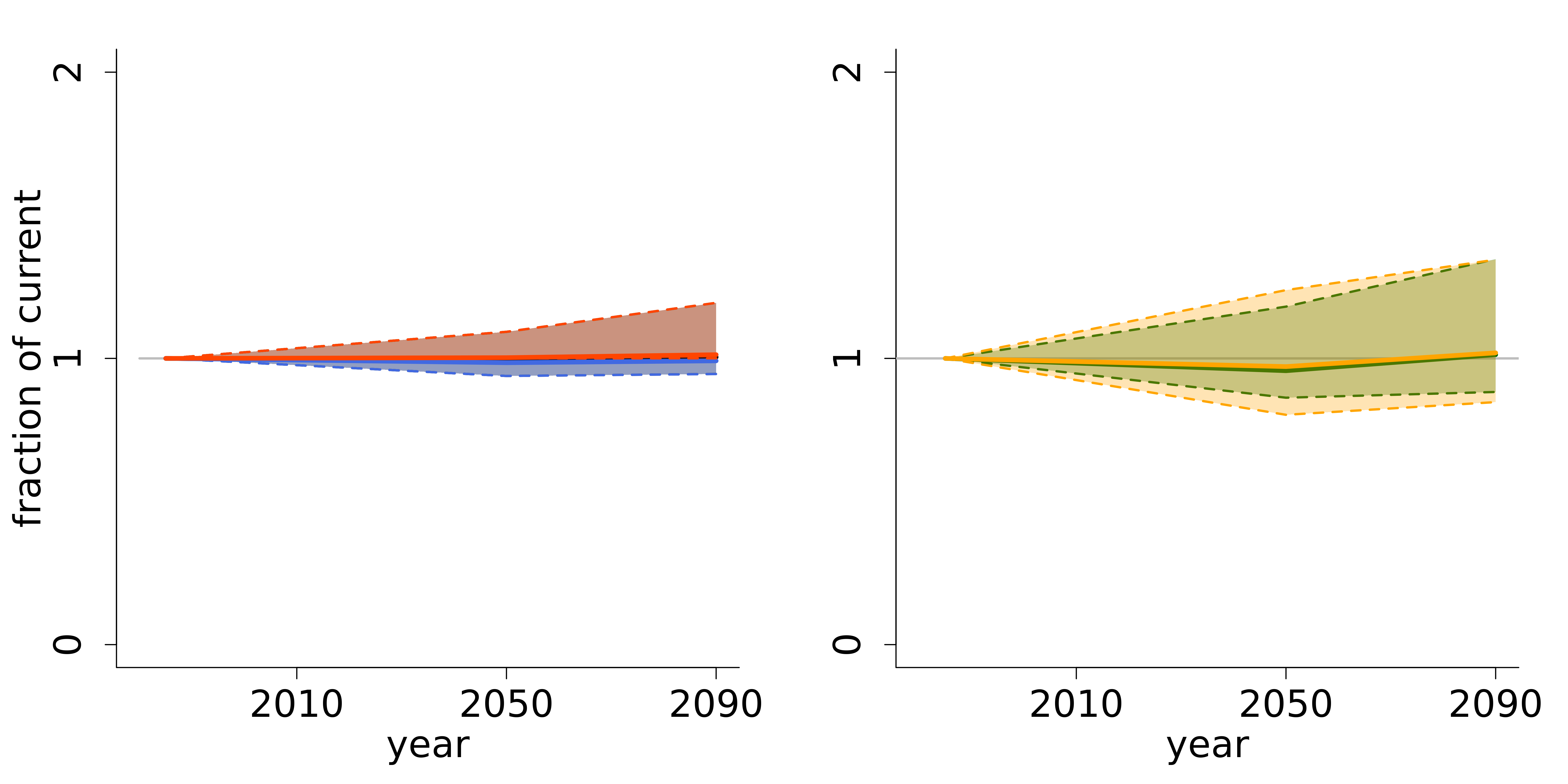

Supplement: S3 Appendix — (ZIP) [file pntd.0014030.s007.zip › Sup. Mat. 6-2 M-Z - Species Trends/Naja_nubiae_CCTrends.png]

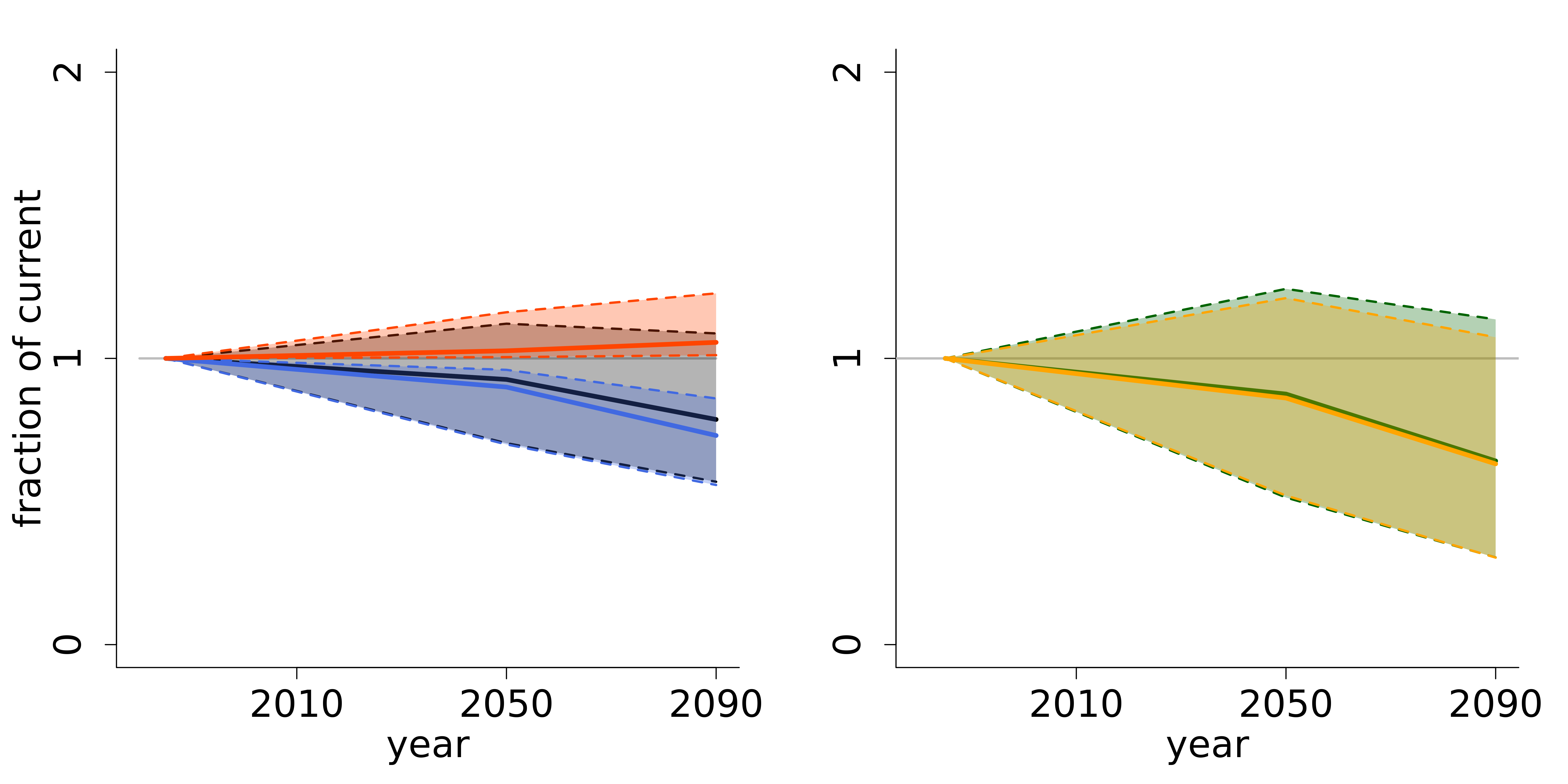

Supplement: S3 Appendix — (ZIP) [file pntd.0014030.s007.zip › Sup. Mat. 6-2 M-Z - Species Trends/Naja_oxiana_CCTrends.png]

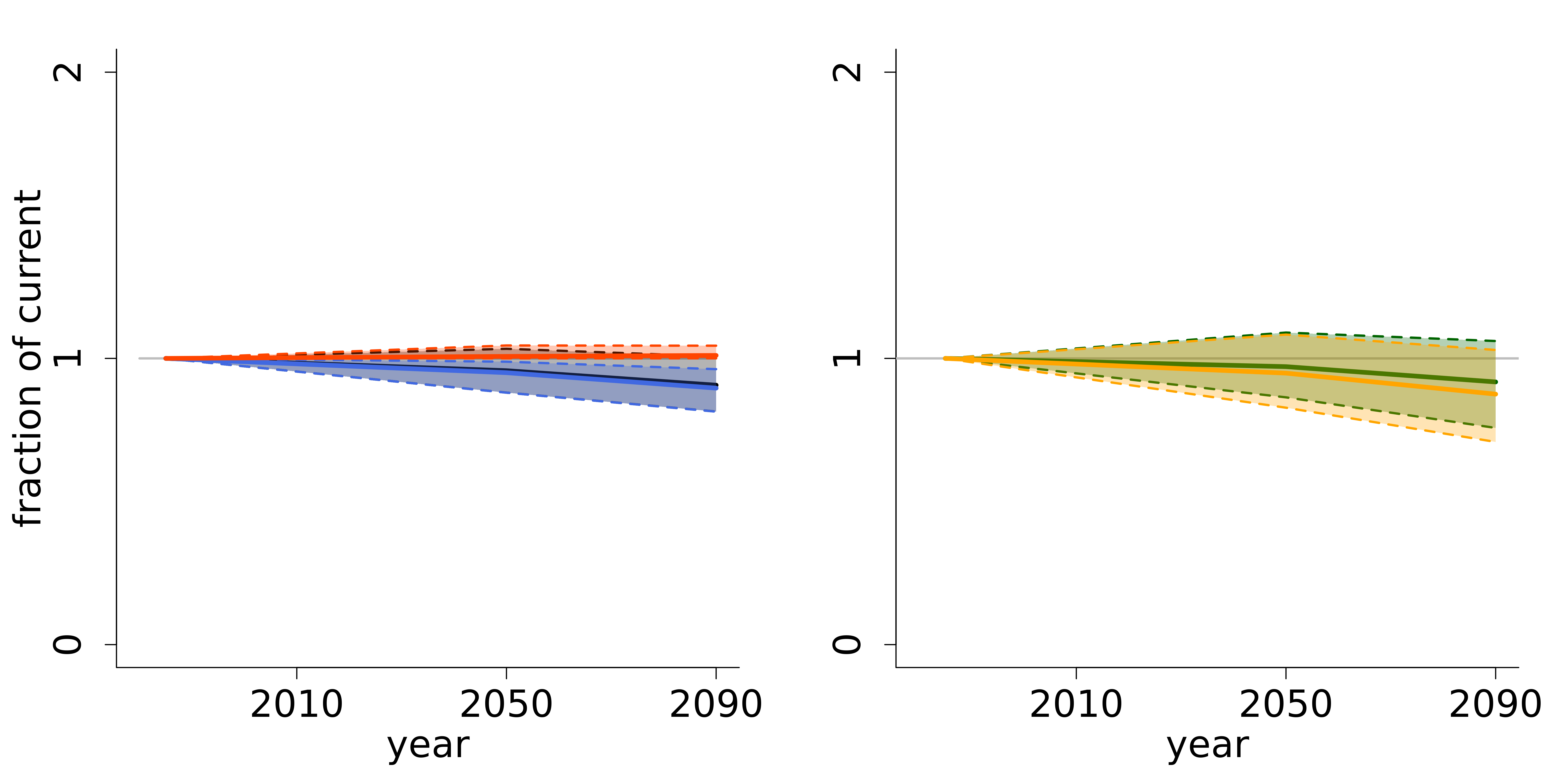

Supplement: S3 Appendix — (ZIP) [file pntd.0014030.s007.zip › Sup. Mat. 6-2 M-Z - Species Trends/Naja_pallida_CCTrends.png]

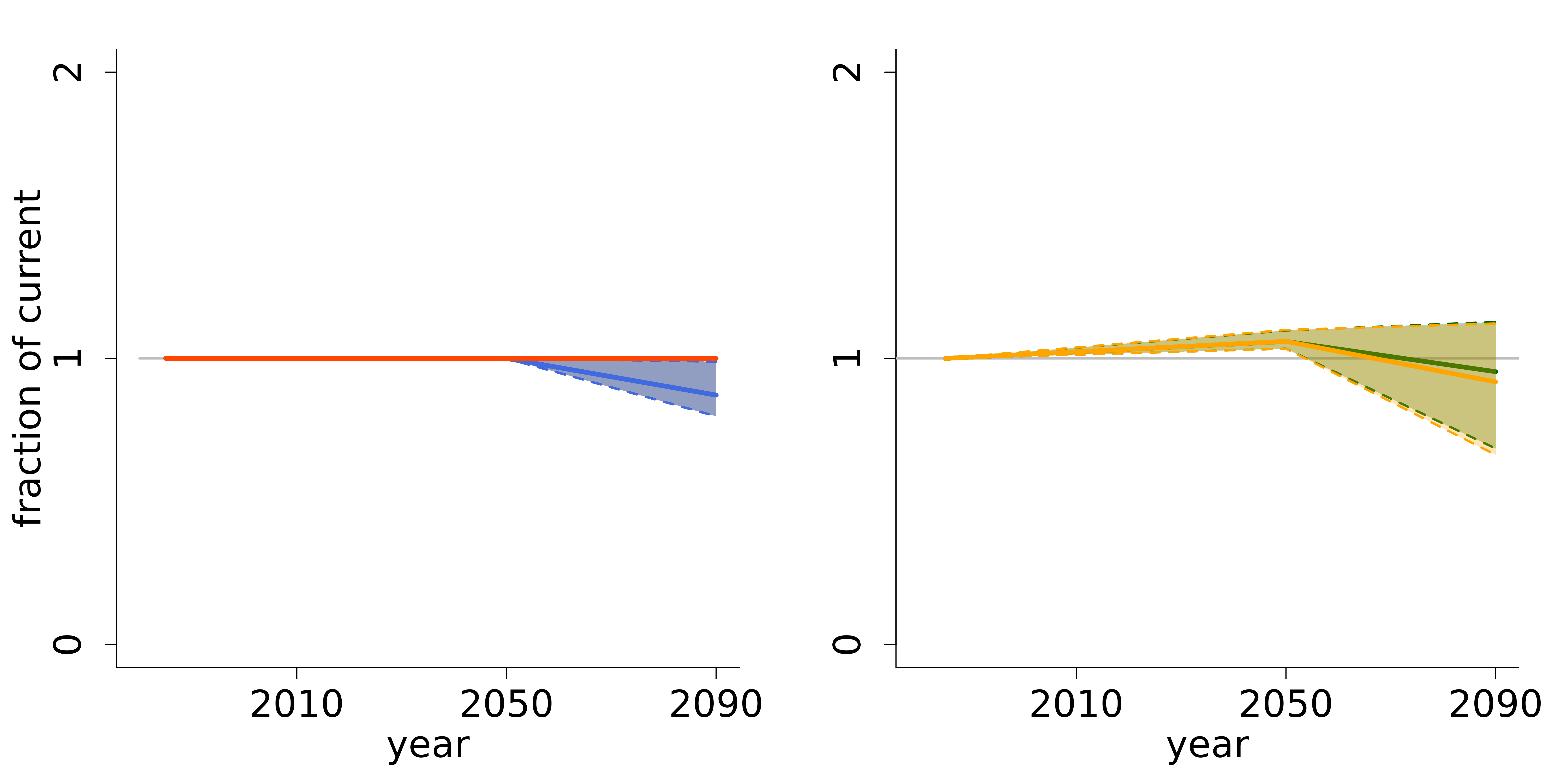

Supplement: S3 Appendix — (ZIP) [file pntd.0014030.s007.zip › Sup. Mat. 6-2 M-Z - Species Trends/Naja_peroescobari_CCTrends.png]

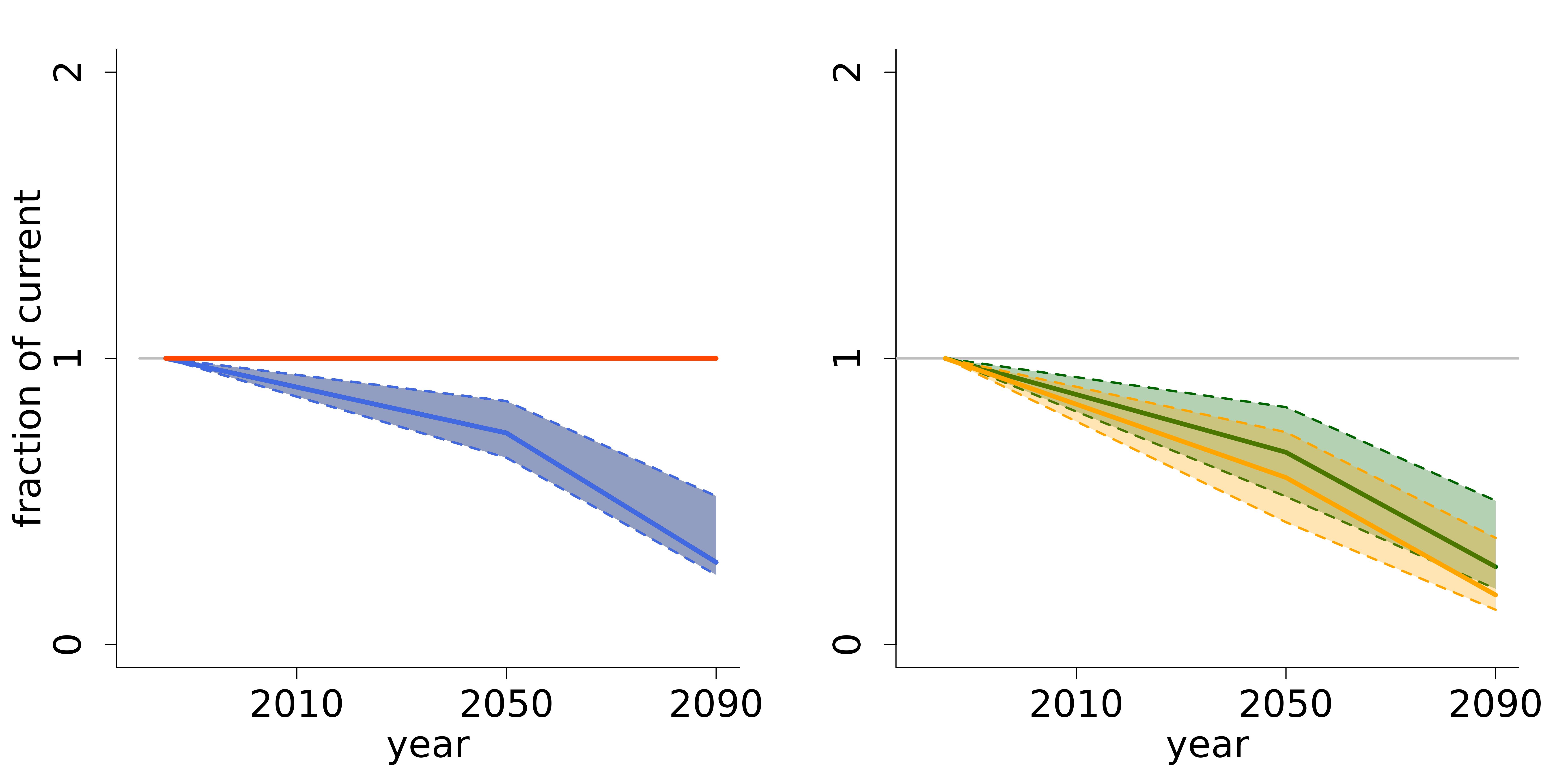

Supplement: S3 Appendix — (ZIP) [file pntd.0014030.s007.zip › Sup. Mat. 6-2 M-Z - Species Trends/Naja_philippinensis_CCTrends.png]

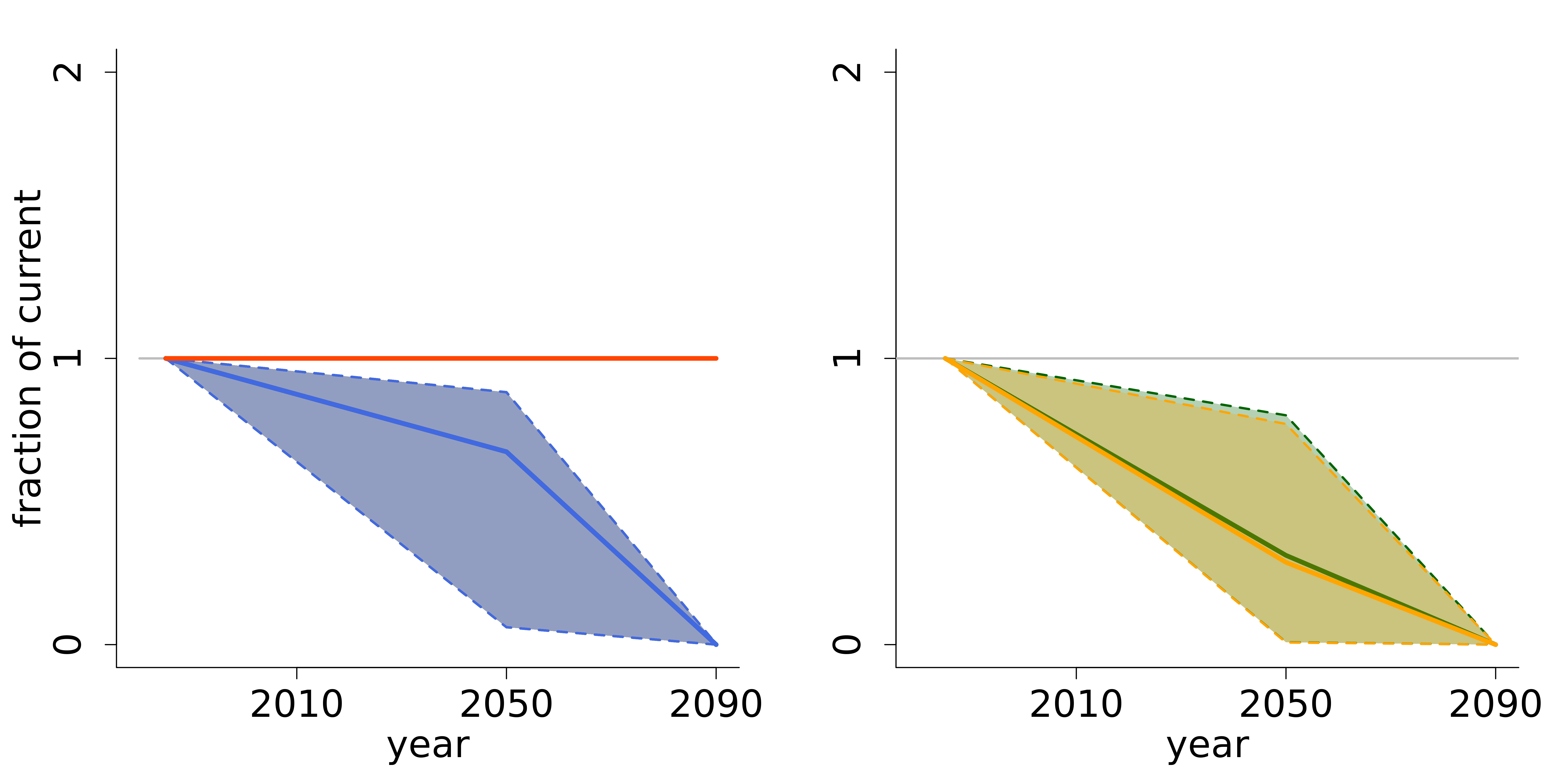

Supplement: S3 Appendix — (ZIP) [file pntd.0014030.s007.zip › Sup. Mat. 6-2 M-Z - Species Trends/Naja_sagittifera_CCTrends.png]

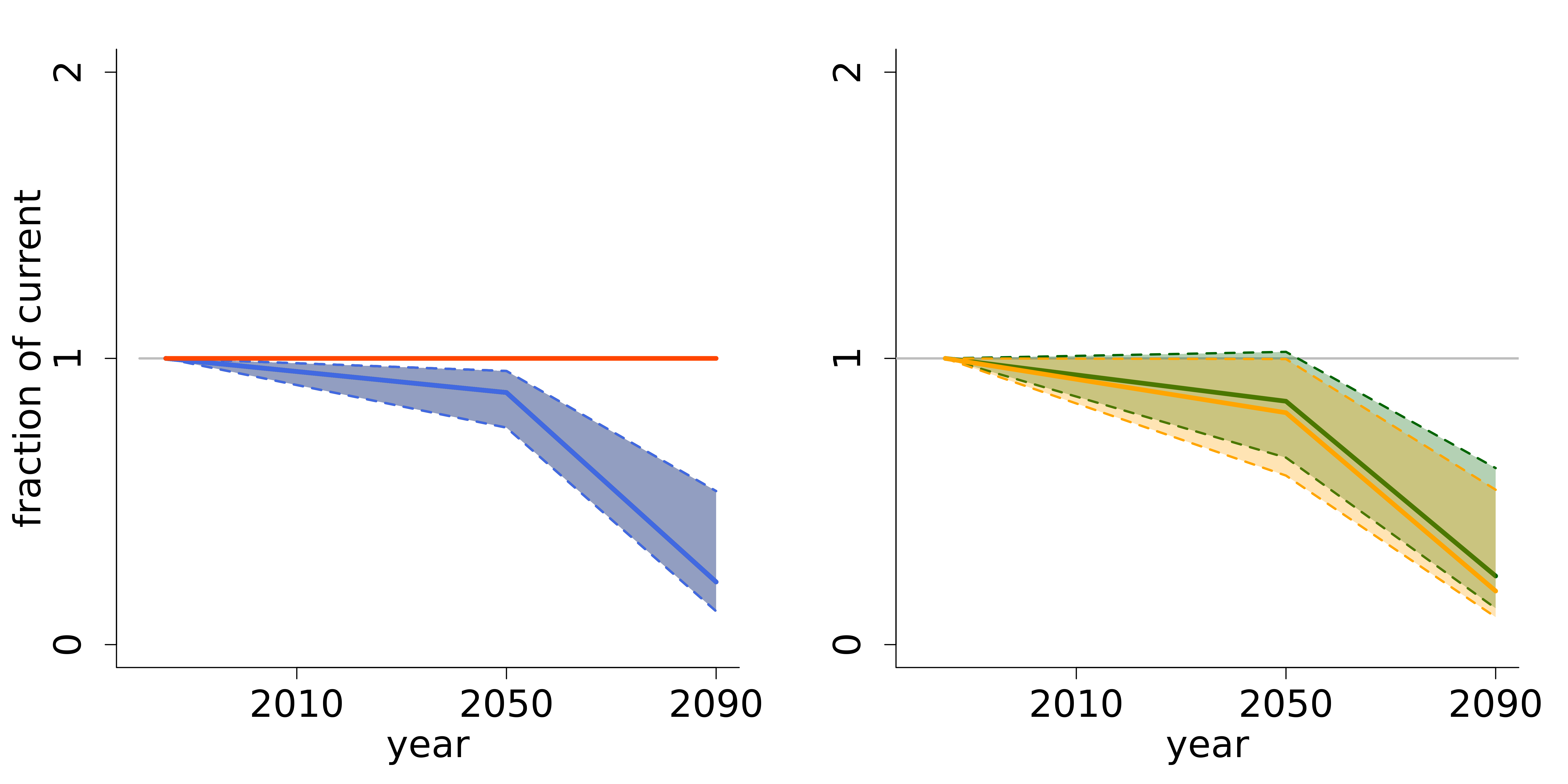

Supplement: S3 Appendix — (ZIP) [file pntd.0014030.s007.zip › Sup. Mat. 6-2 M-Z - Species Trends/Naja_samarensis_CCTrends.png]

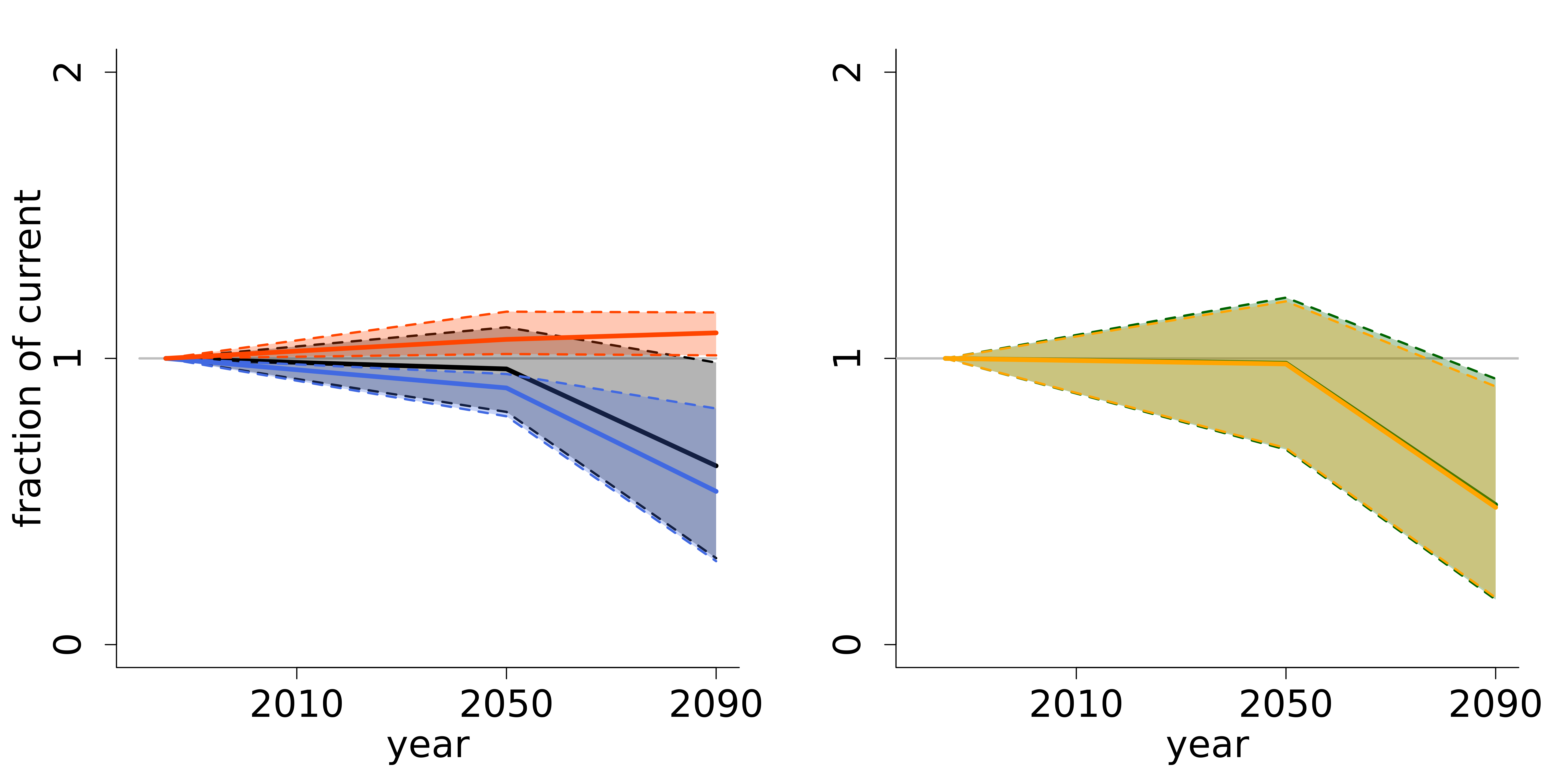

Supplement: S3 Appendix — (ZIP) [file pntd.0014030.s007.zip › Sup. Mat. 6-2 M-Z - Species Trends/Naja_savannula_CCTrends.png]

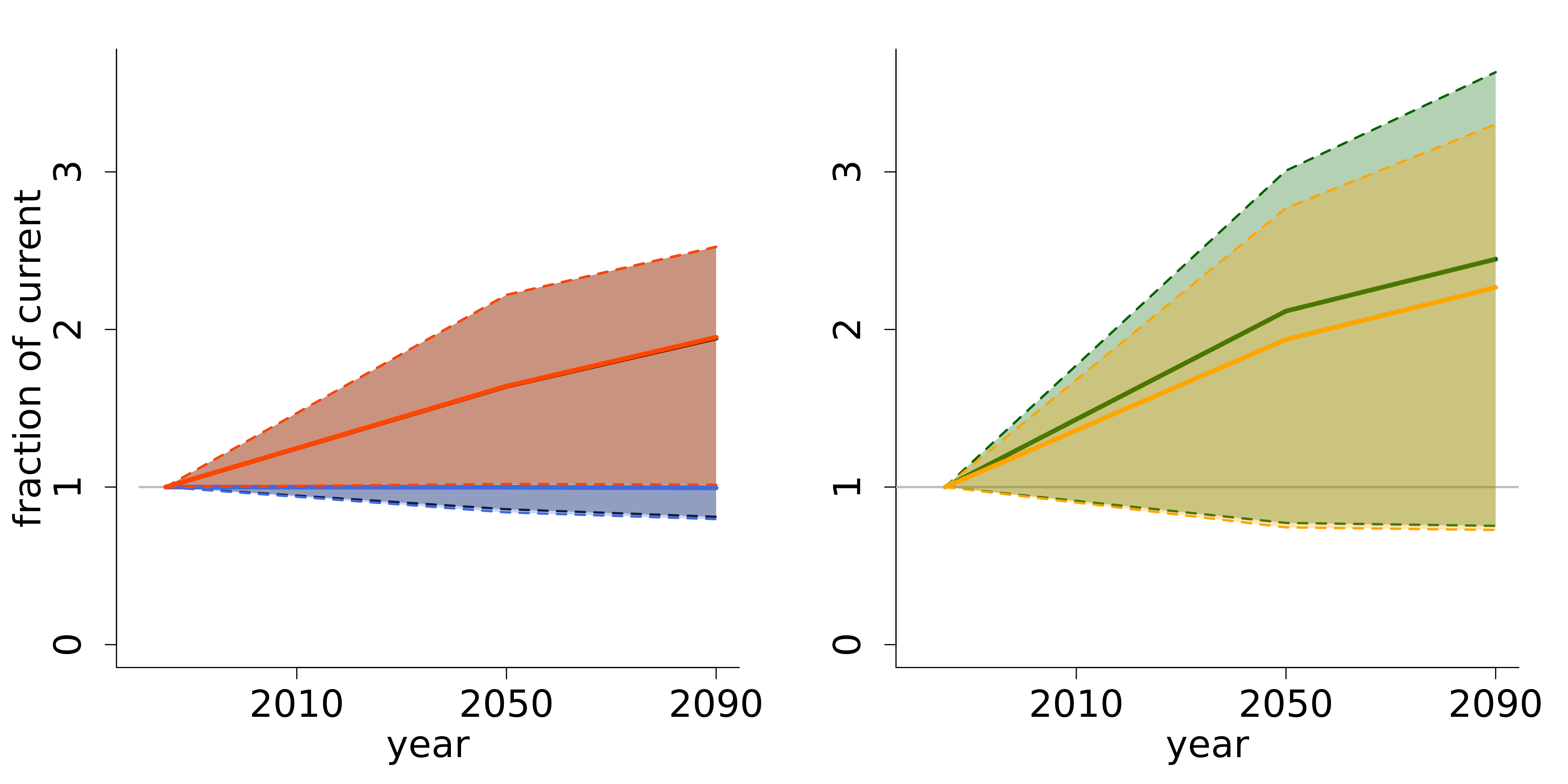

Supplement: S3 Appendix — (ZIP) [file pntd.0014030.s007.zip › Sup. Mat. 6-2 M-Z - Species Trends/Naja_senegalensis_CCTrends.png]

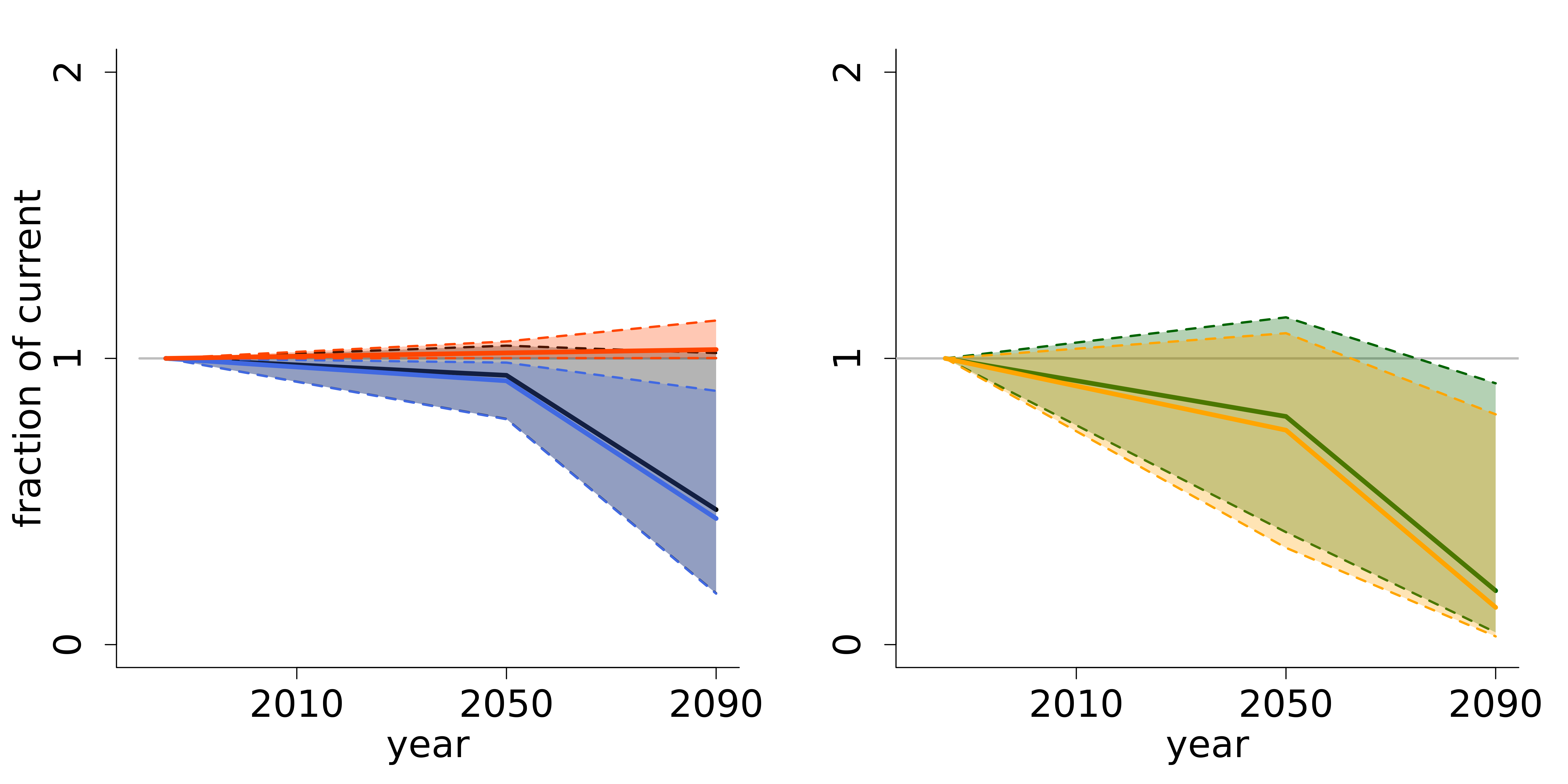

Supplement: S3 Appendix — (ZIP) [file pntd.0014030.s007.zip › Sup. Mat. 6-2 M-Z - Species Trends/Naja_siamensis_CCTrends.png]

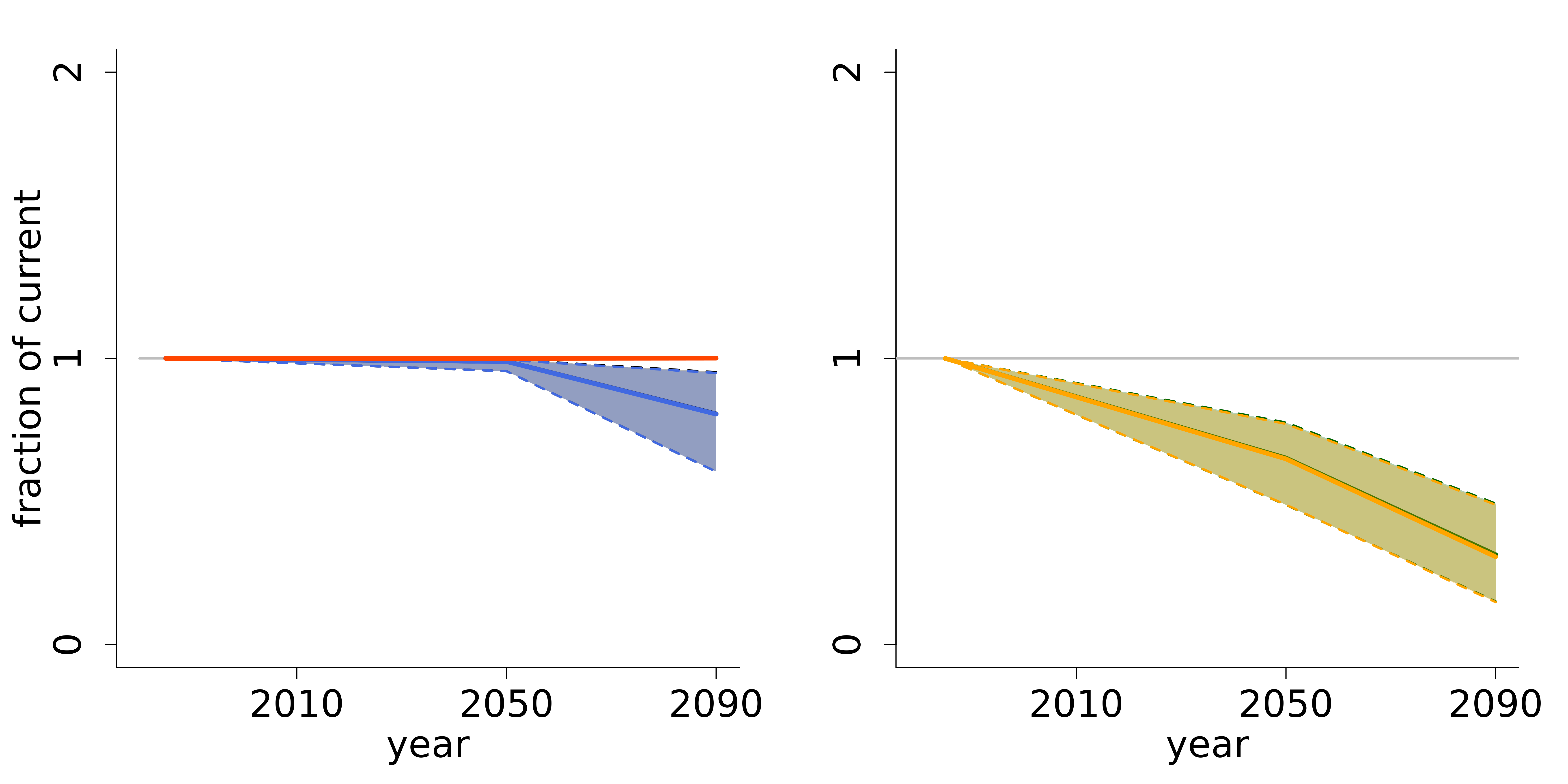

Supplement: S3 Appendix — (ZIP) [file pntd.0014030.s007.zip › Sup. Mat. 6-2 M-Z - Species Trends/Naja_sputatrix_CCTrends.png]

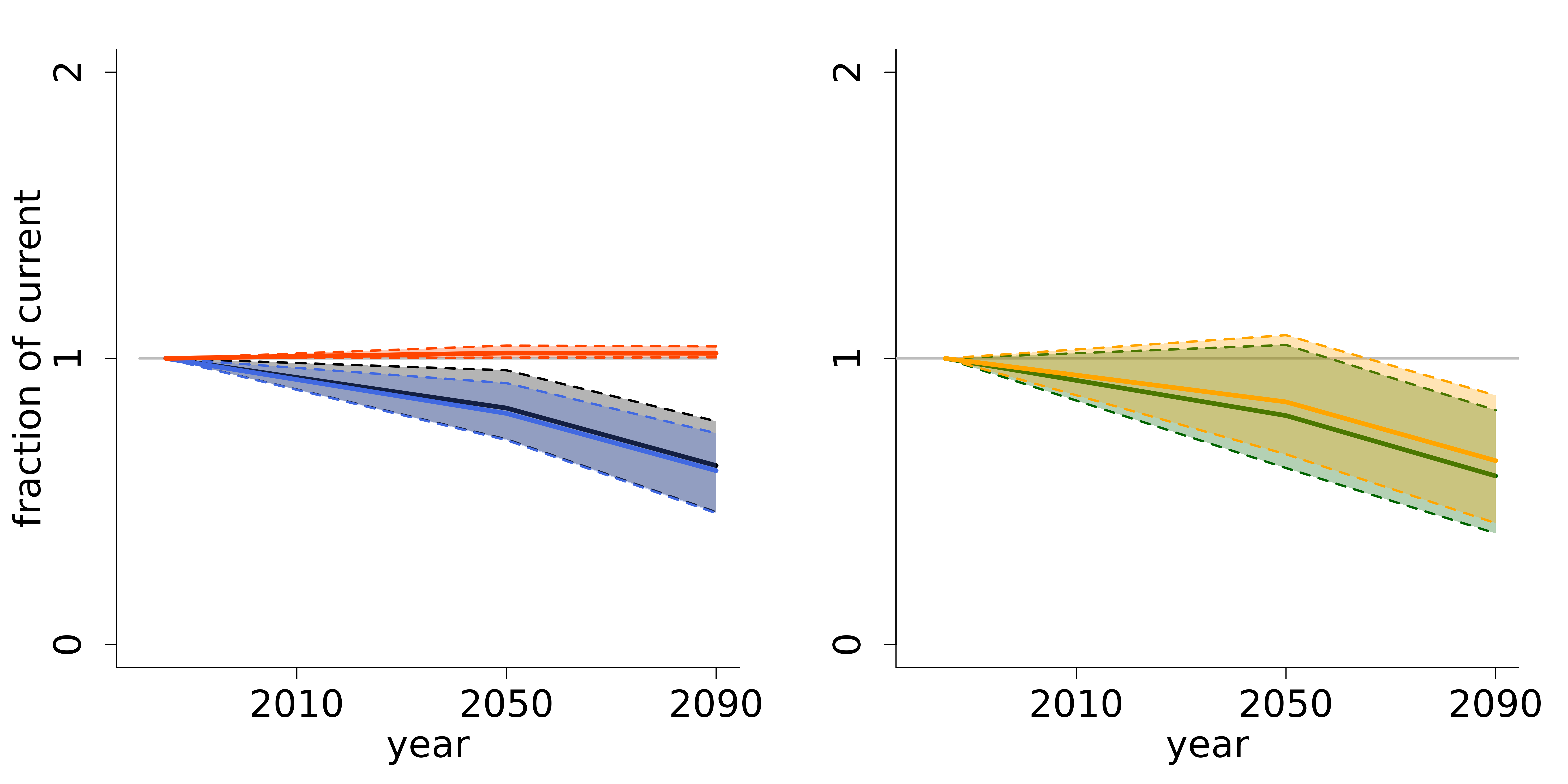

Supplement: S3 Appendix — (ZIP) [file pntd.0014030.s007.zip › Sup. Mat. 6-2 M-Z - Species Trends/Naja_subfulva_CCTrends.png]

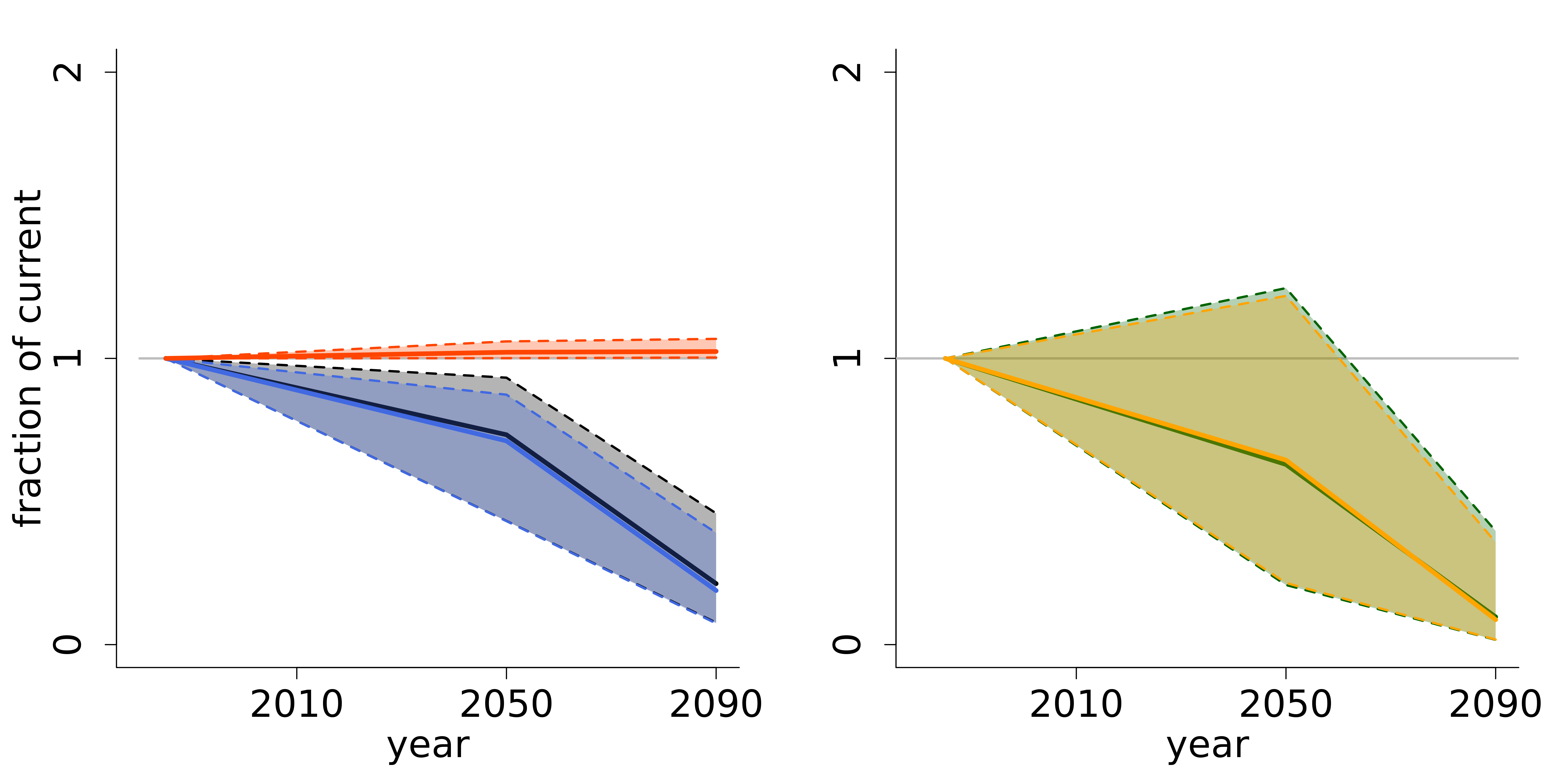

Supplement: S3 Appendix — (ZIP) [file pntd.0014030.s007.zip › Sup. Mat. 6-2 M-Z - Species Trends/Naja_sumatrana_CCTrends.png]

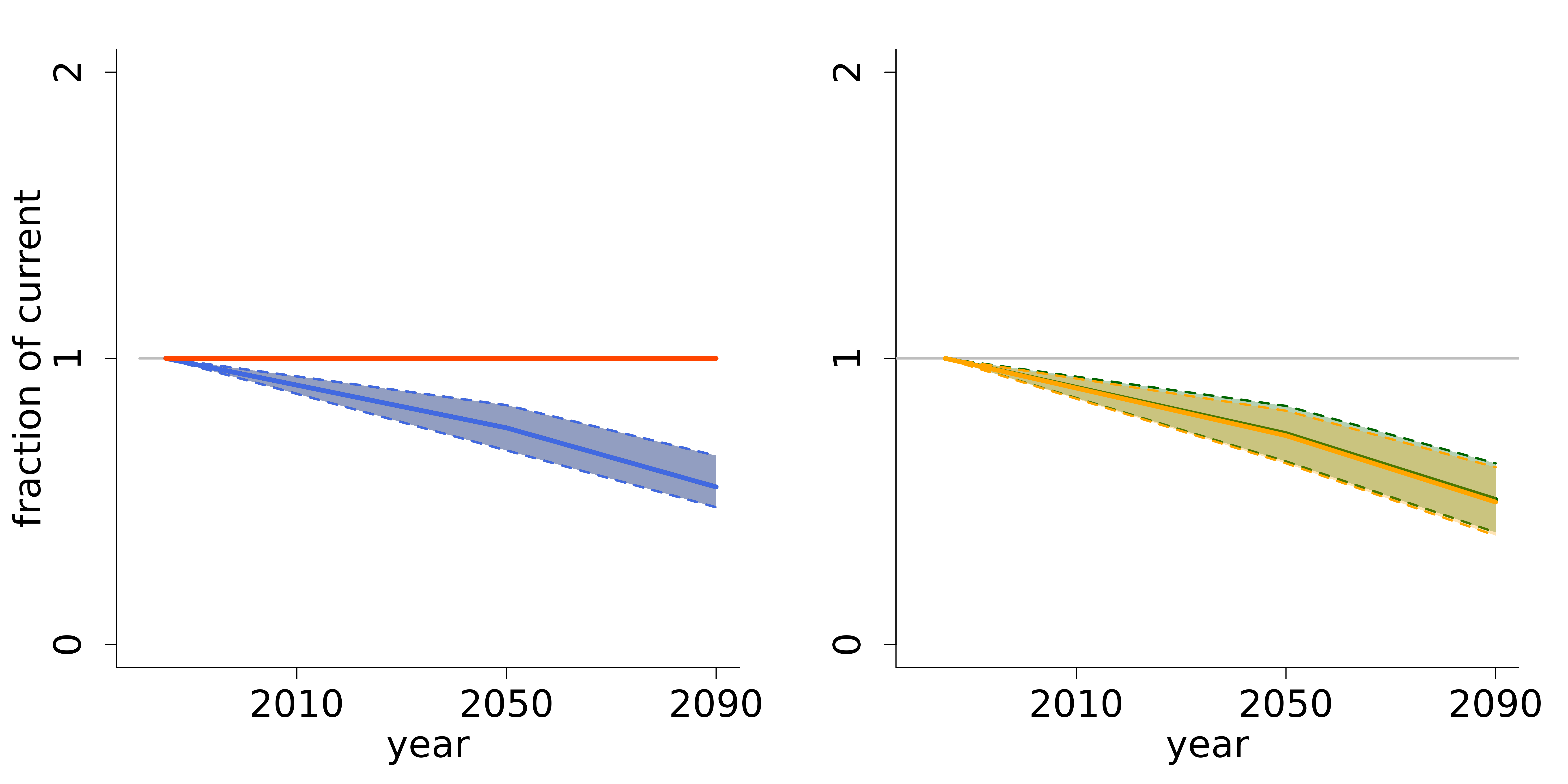

Supplement: S3 Appendix — (ZIP) [file pntd.0014030.s007.zip › Sup. Mat. 6-2 M-Z - Species Trends/Notechis_scutatus_CCTrends.png]

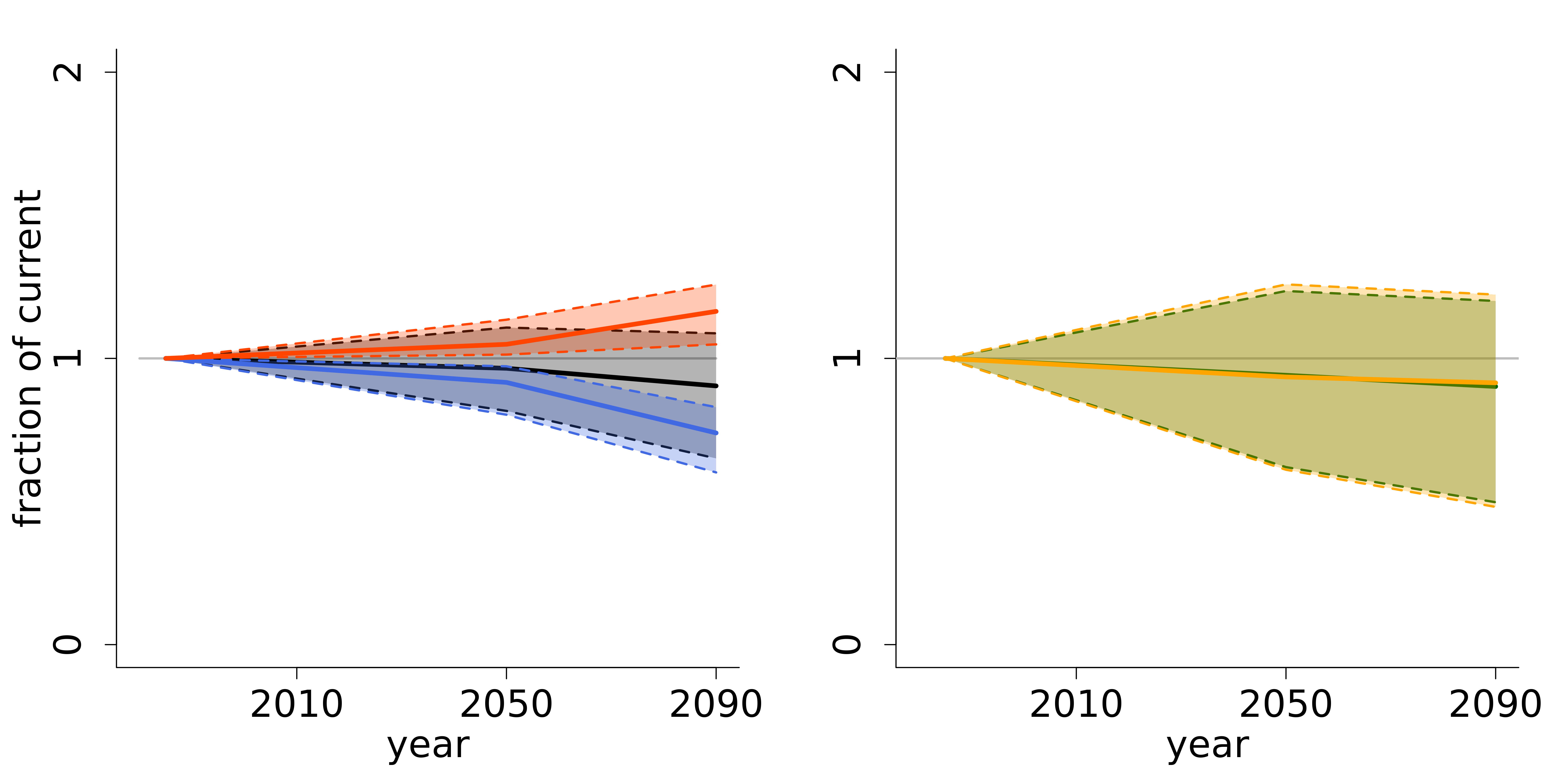

Supplement: S3 Appendix — (ZIP) [file pntd.0014030.s007.zip › Sup. Mat. 6-2 M-Z - Species Trends/Ophiophagus_hannah_CCTrends.png]

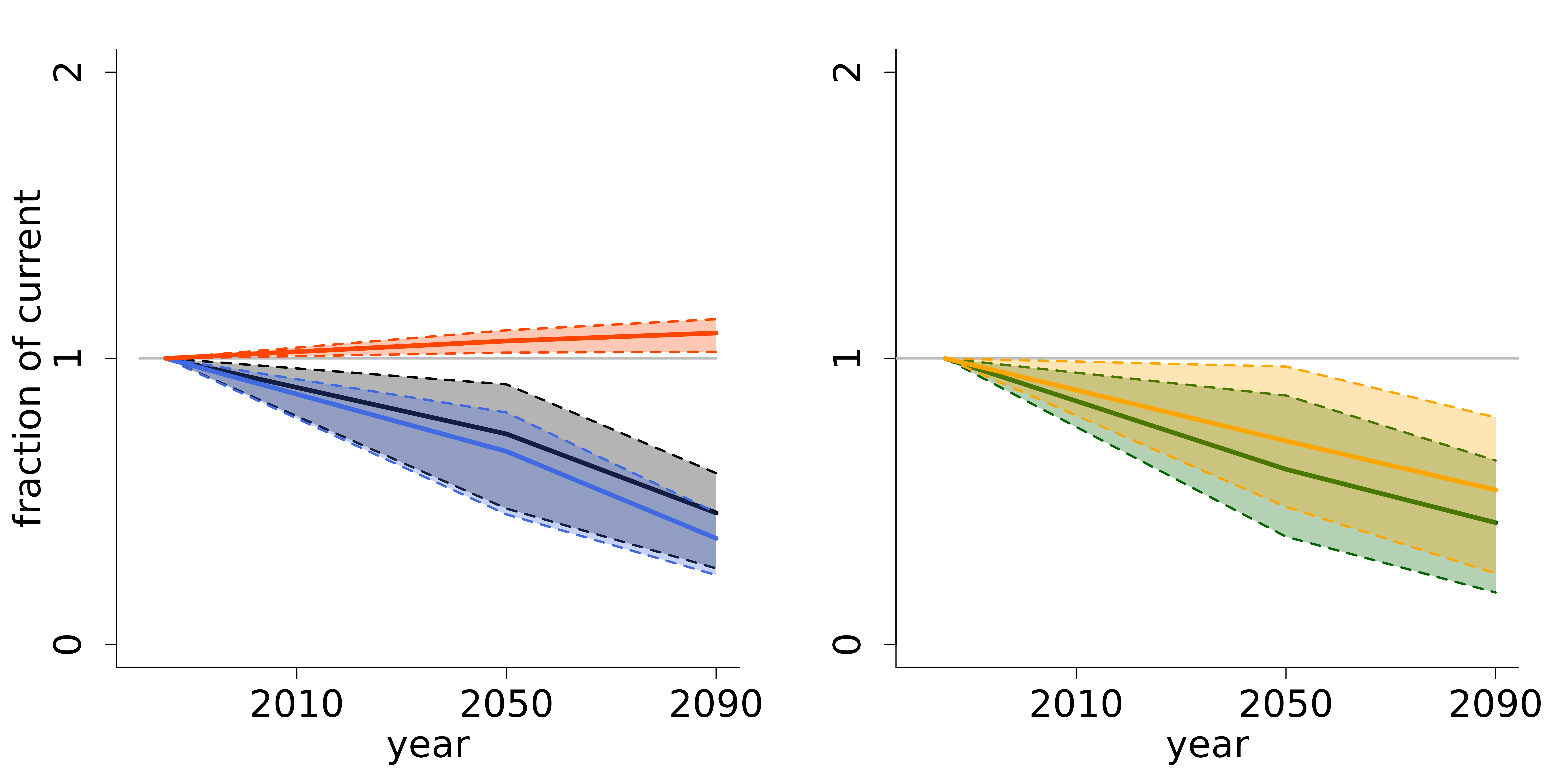

Supplement: S3 Appendix — (ZIP) [file pntd.0014030.s007.zip › Sup. Mat. 6-2 M-Z - Species Trends/Ophryacus_smaragdinus_CCTrends.png]

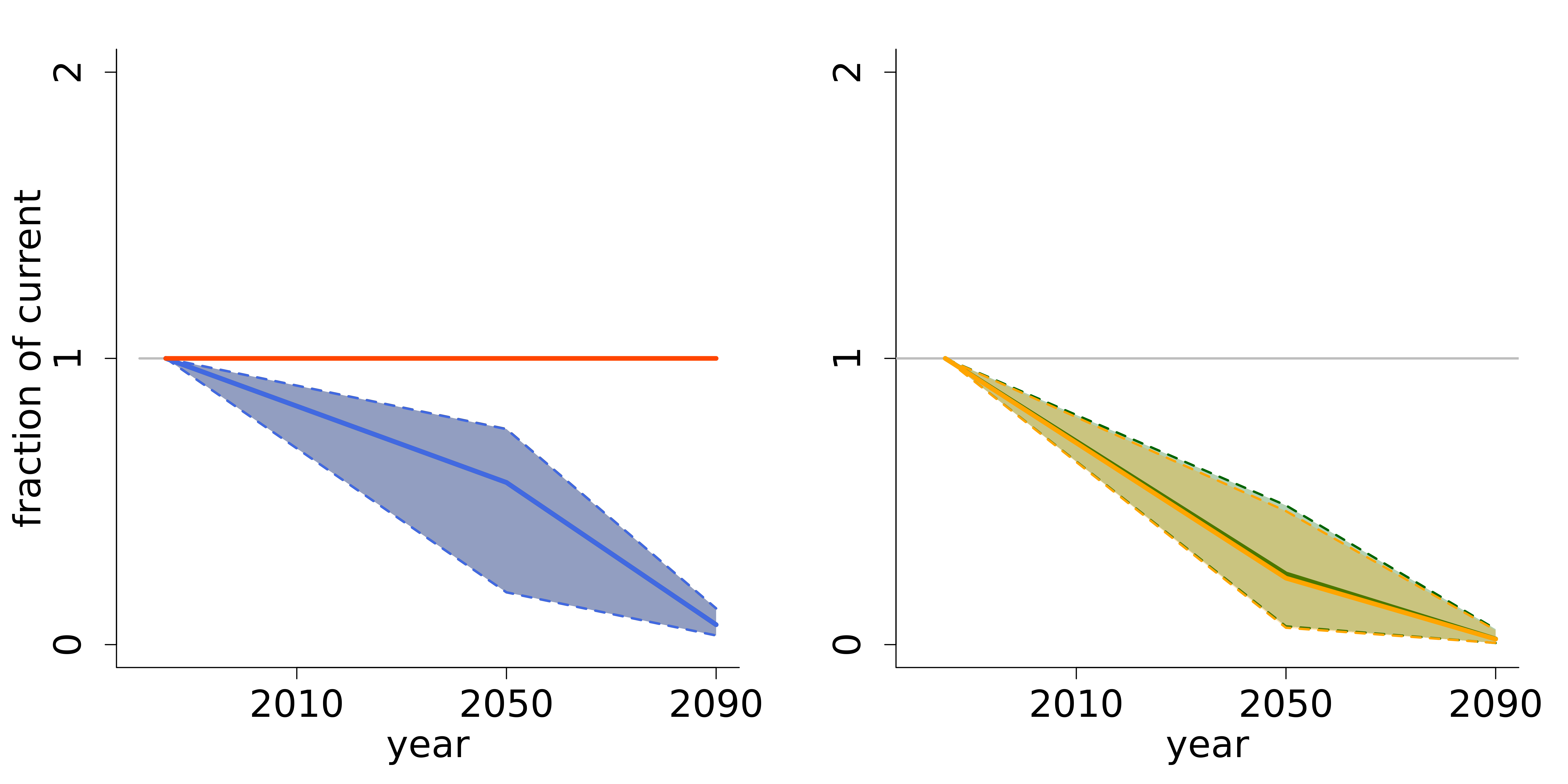

Supplement: S3 Appendix — (ZIP) [file pntd.0014030.s007.zip › Sup. Mat. 6-2 M-Z - Species Trends/Ophryacus_sphenophrys_CCTrends.png]

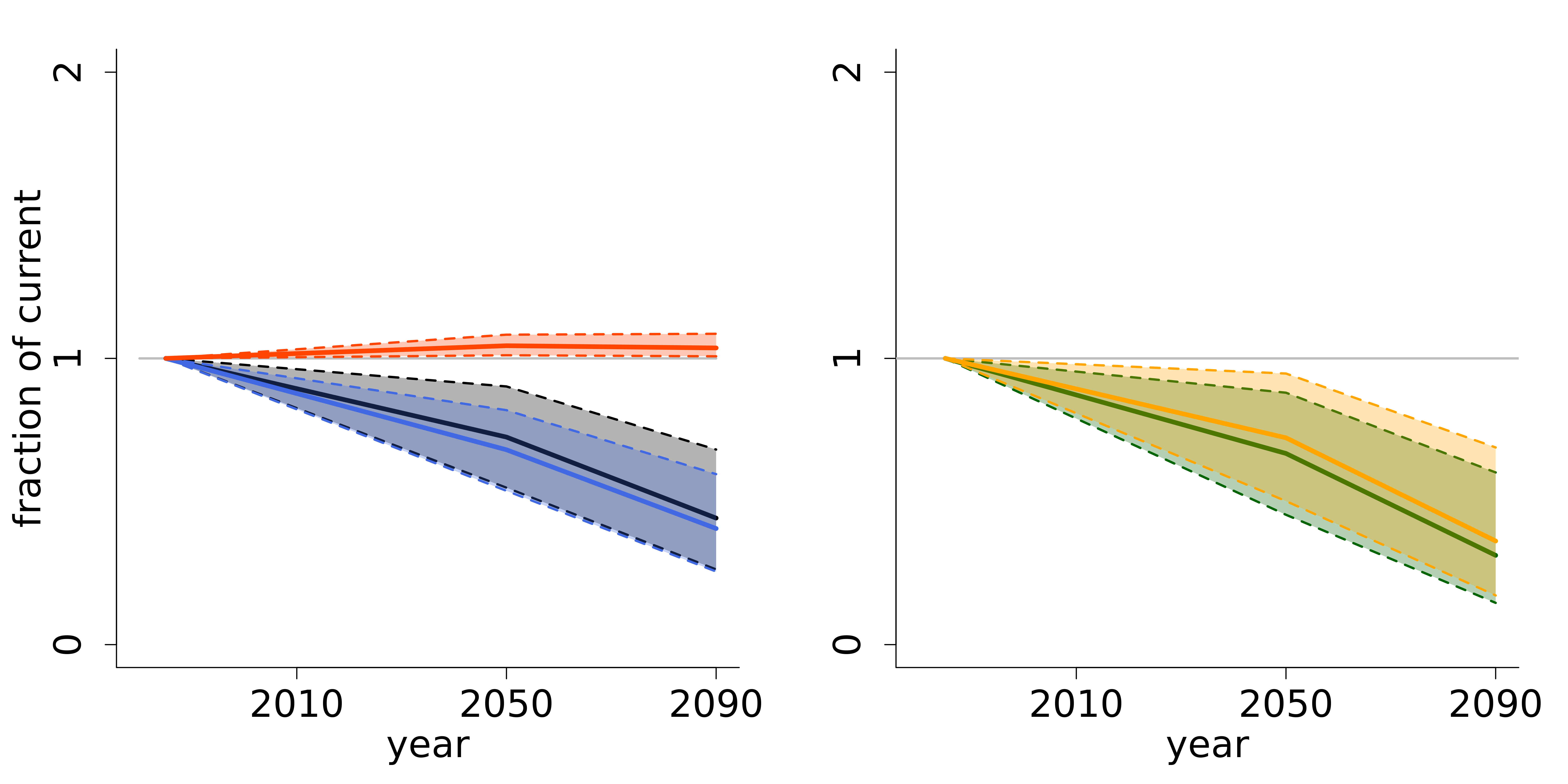

Supplement: S3 Appendix — (ZIP) [file pntd.0014030.s007.zip › Sup. Mat. 6-2 M-Z - Species Trends/Ophryacus_undulatus_CCTrends.png]

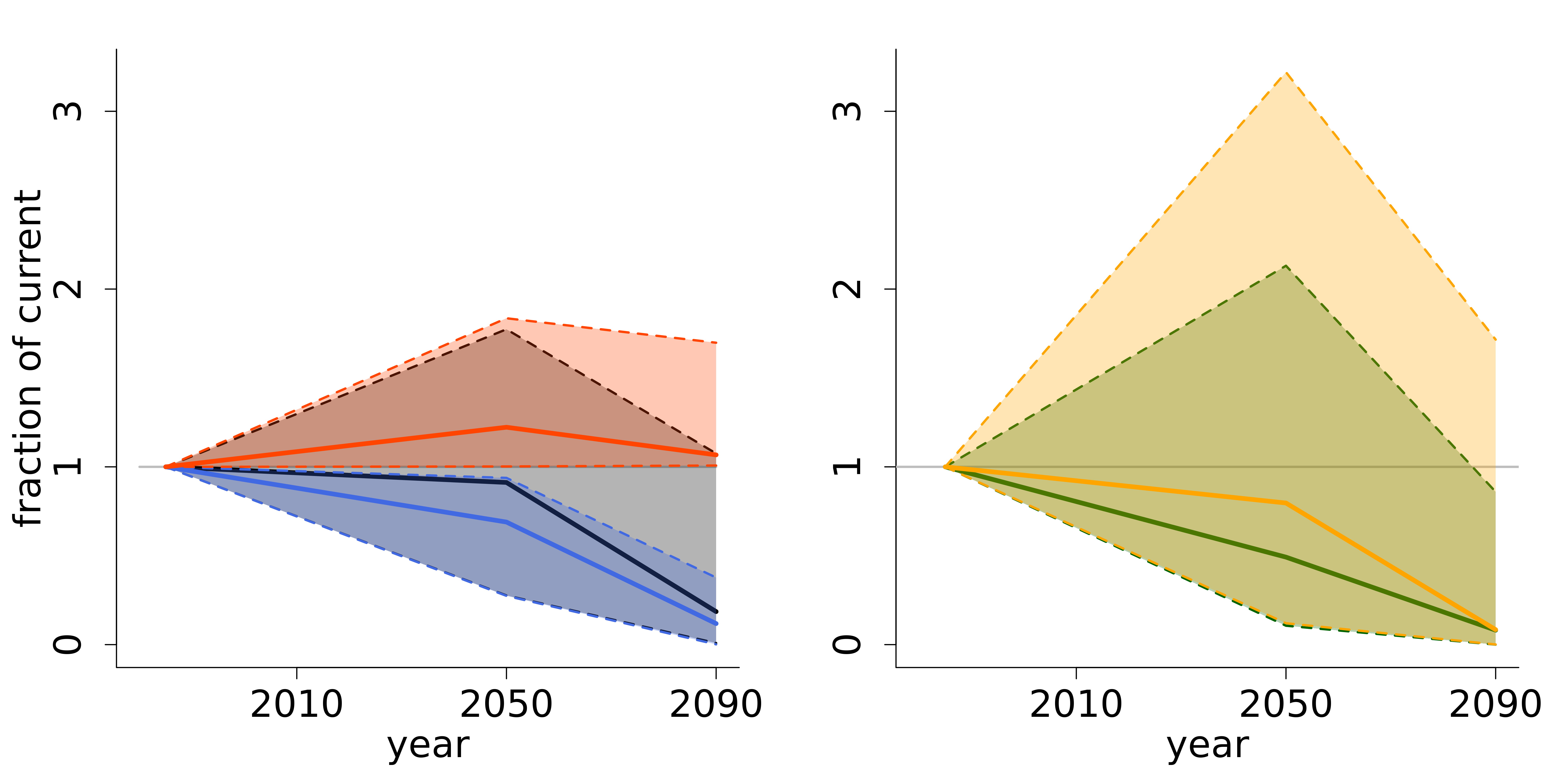

Supplement: S3 Appendix — (ZIP) [file pntd.0014030.s007.zip › Sup. Mat. 6-2 M-Z - Species Trends/Oxyuranus_microlepidotus_CCTrends.png]

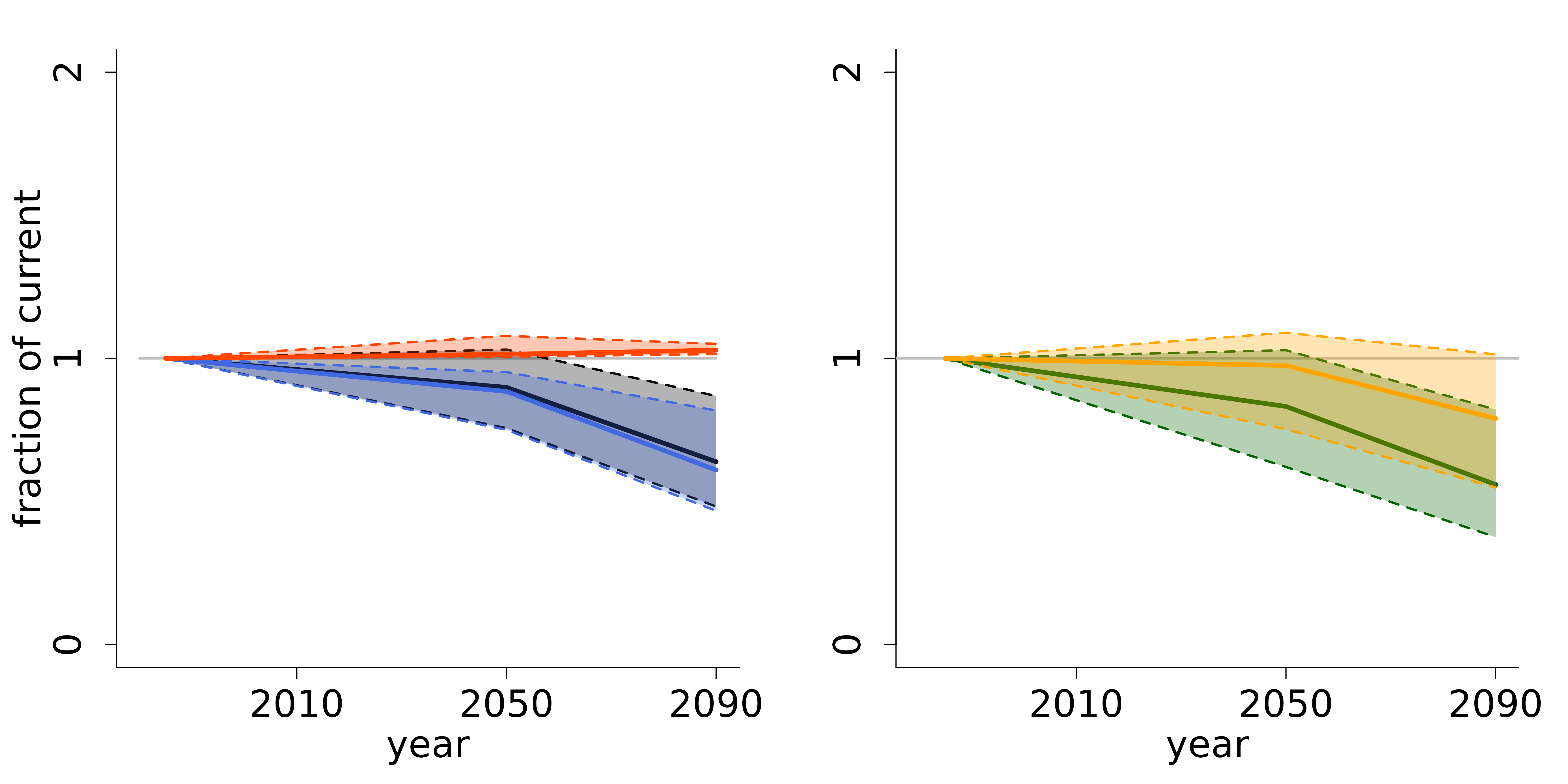

Supplement: S3 Appendix — (ZIP) [file pntd.0014030.s007.zip › Sup. Mat. 6-2 M-Z - Species Trends/Oxyuranus_scutellatus_CCTrends.png]

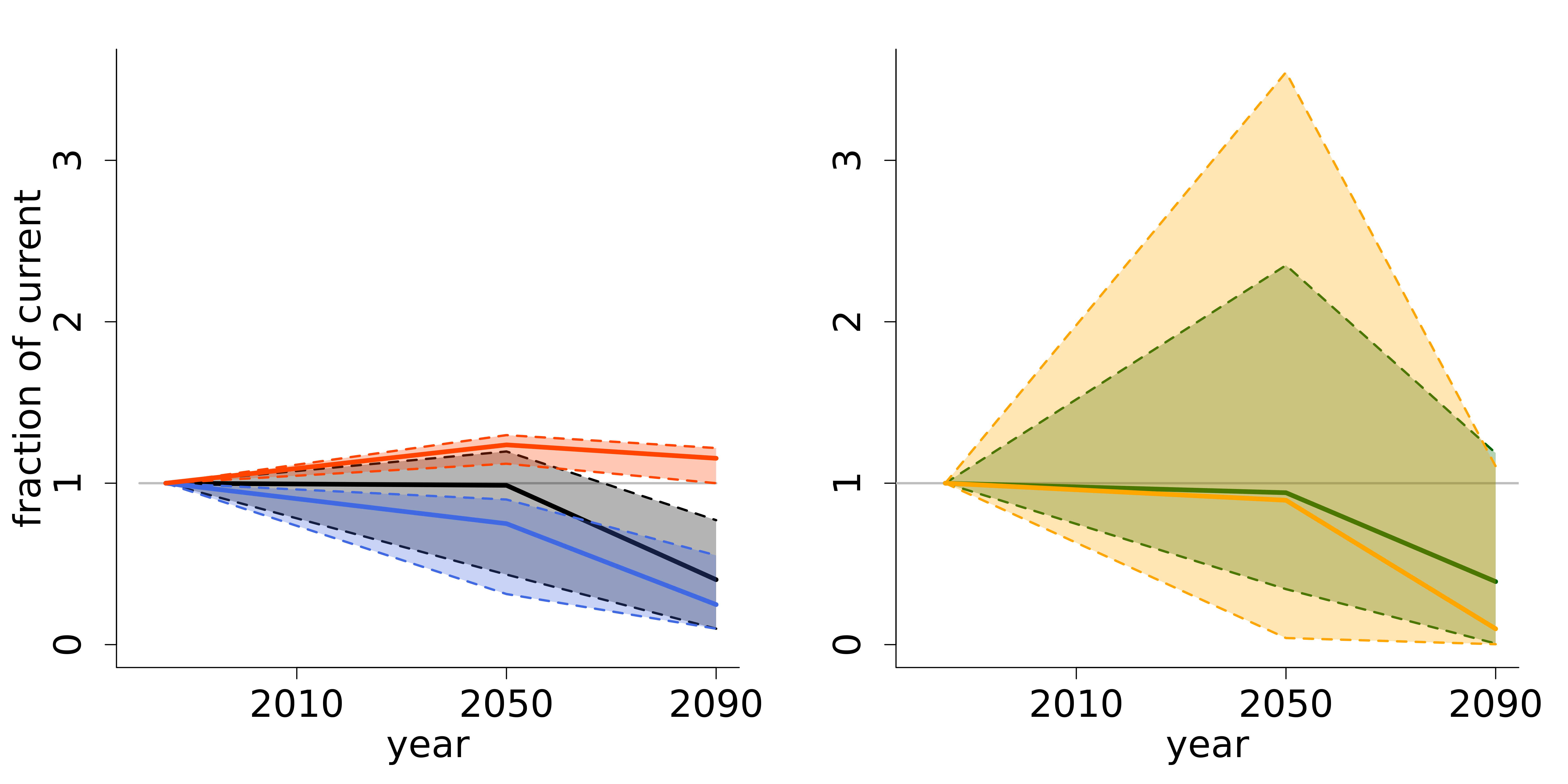

Supplement: S3 Appendix — (ZIP) [file pntd.0014030.s007.zip › Sup. Mat. 6-2 M-Z - Species Trends/Oxyuranus_temporalis_CCTrends.png]

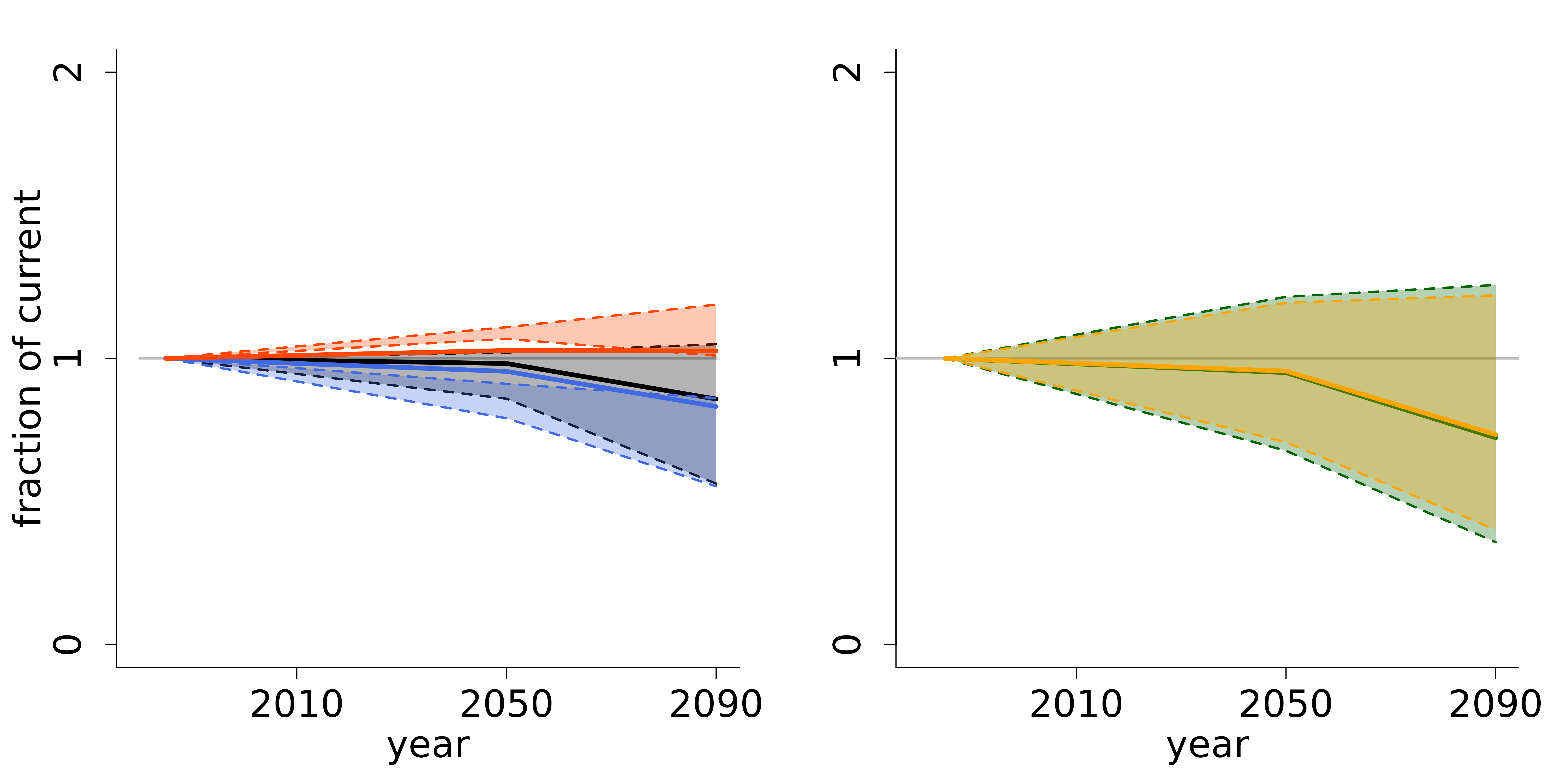

Supplement: S3 Appendix — (ZIP) [file pntd.0014030.s007.zip › Sup. Mat. 6-2 M-Z - Species Trends/Porthidium_arcosae_CCTrends.png]

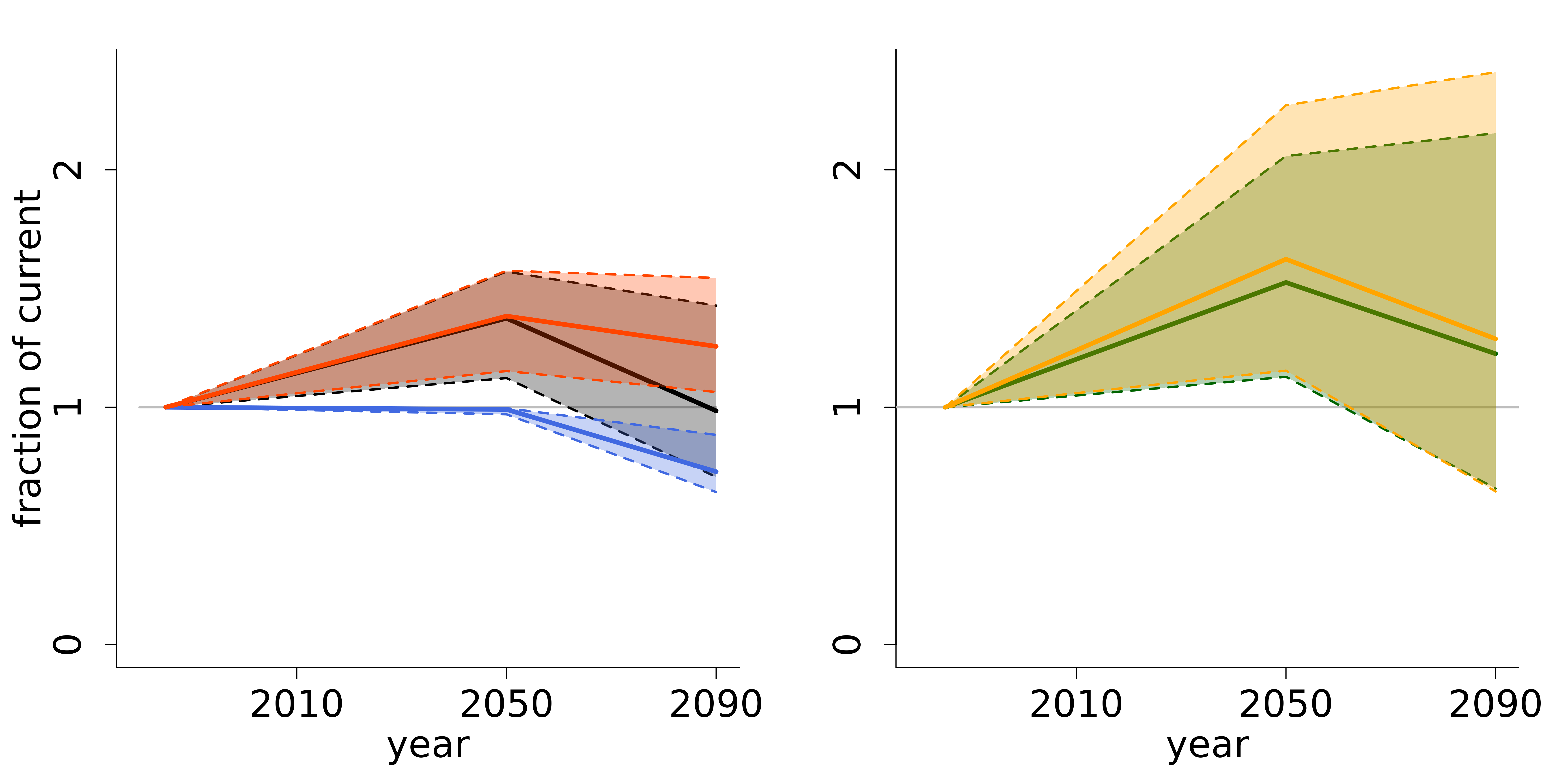

Supplement: S3 Appendix — (ZIP) [file pntd.0014030.s007.zip › Sup. Mat. 6-2 M-Z - Species Trends/Porthidium_dunni_CCTrends.png]

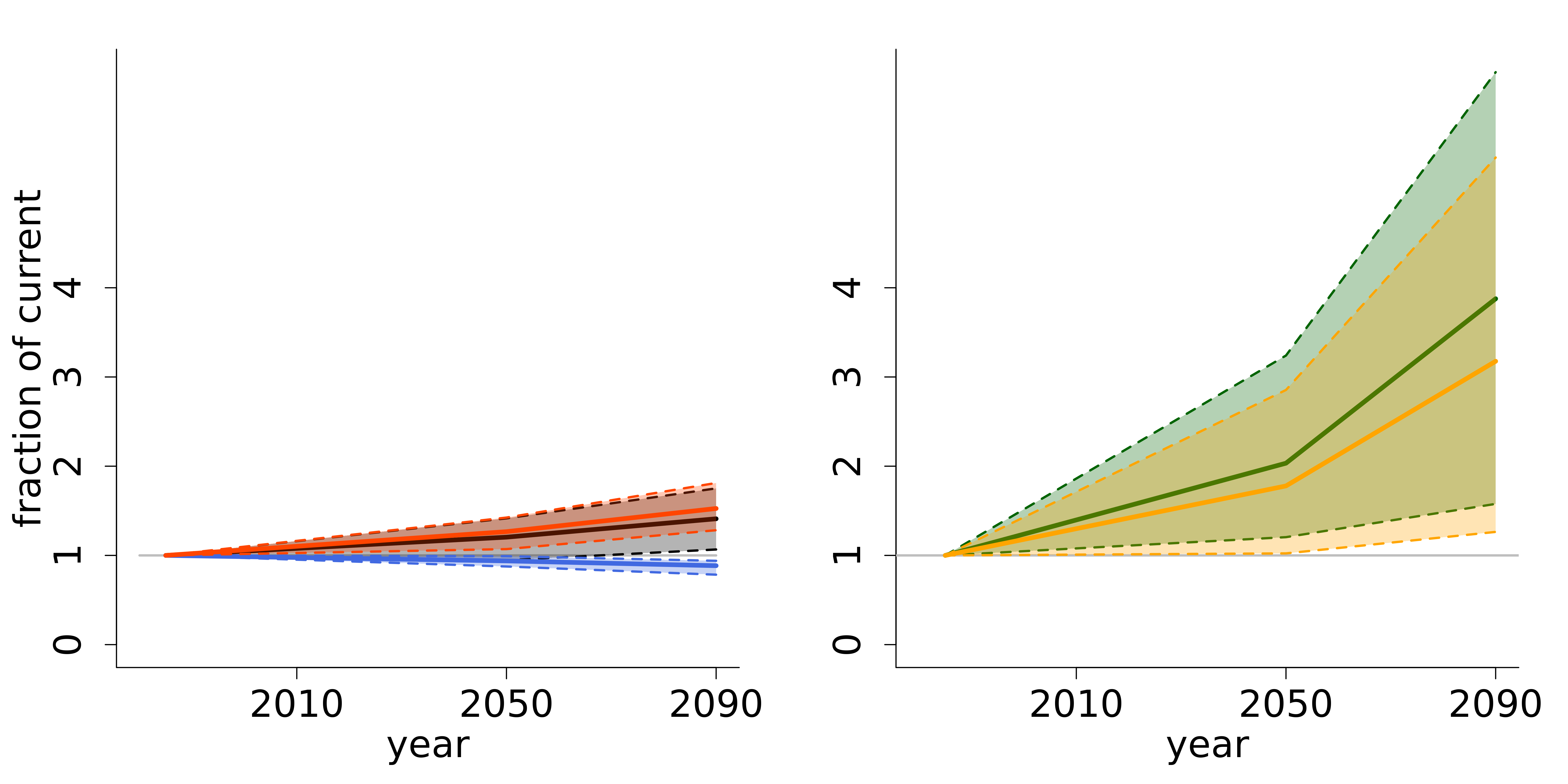

Supplement: S3 Appendix — (ZIP) [file pntd.0014030.s007.zip › Sup. Mat. 6-2 M-Z - Species Trends/Porthidium_hespere_CCTrends.png]

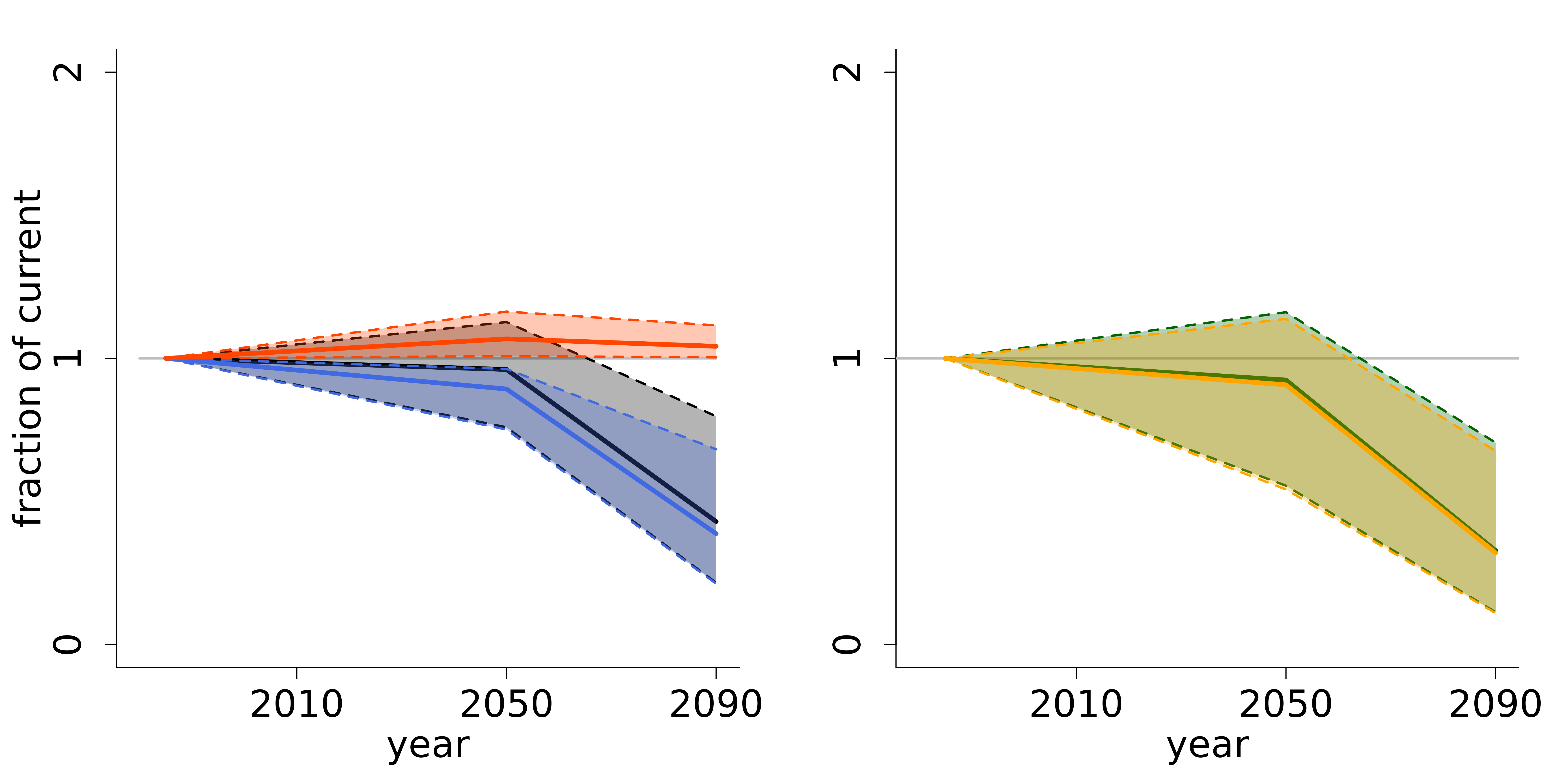

Supplement: S3 Appendix — (ZIP) [file pntd.0014030.s007.zip › Sup. Mat. 6-2 M-Z - Species Trends/Porthidium_lansbergii_CCTrends.png]

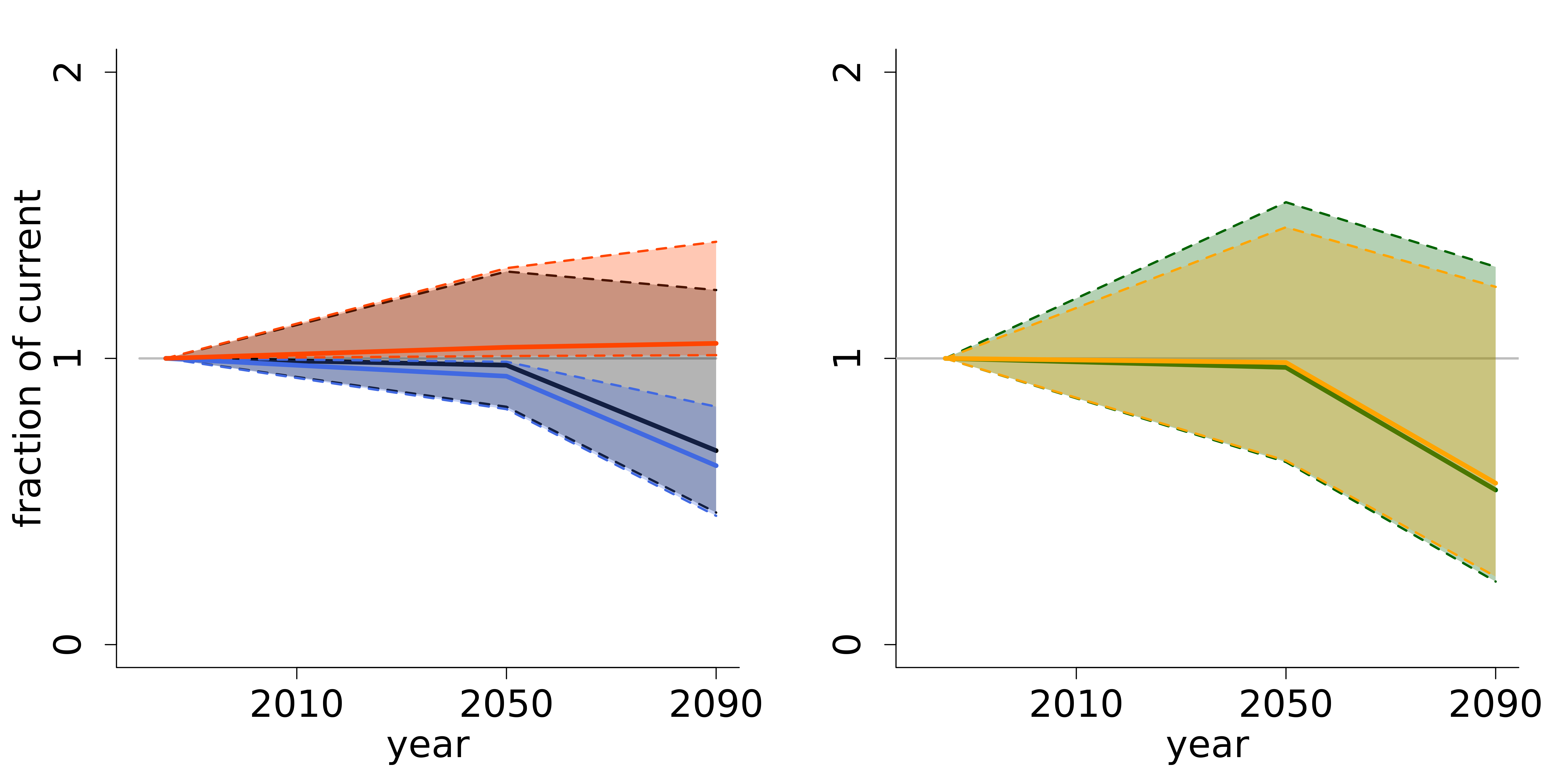

Supplement: S3 Appendix — (ZIP) [file pntd.0014030.s007.zip › Sup. Mat. 6-2 M-Z - Species Trends/Porthidium_nasutum_CCTrends.png]

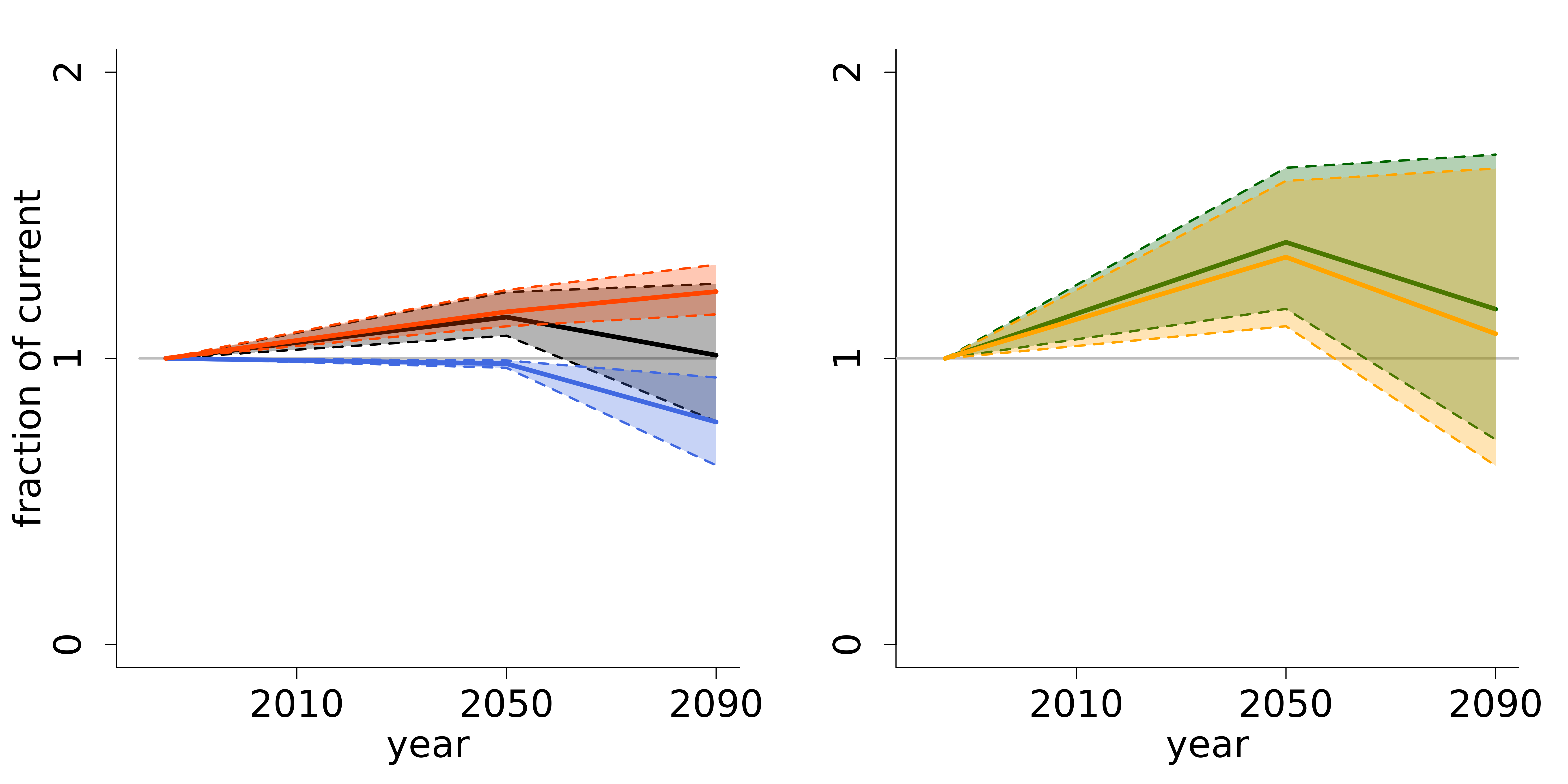

Supplement: S3 Appendix — (ZIP) [file pntd.0014030.s007.zip › Sup. Mat. 6-2 M-Z - Species Trends/Porthidium_ophryomegas_CCTrends.png]

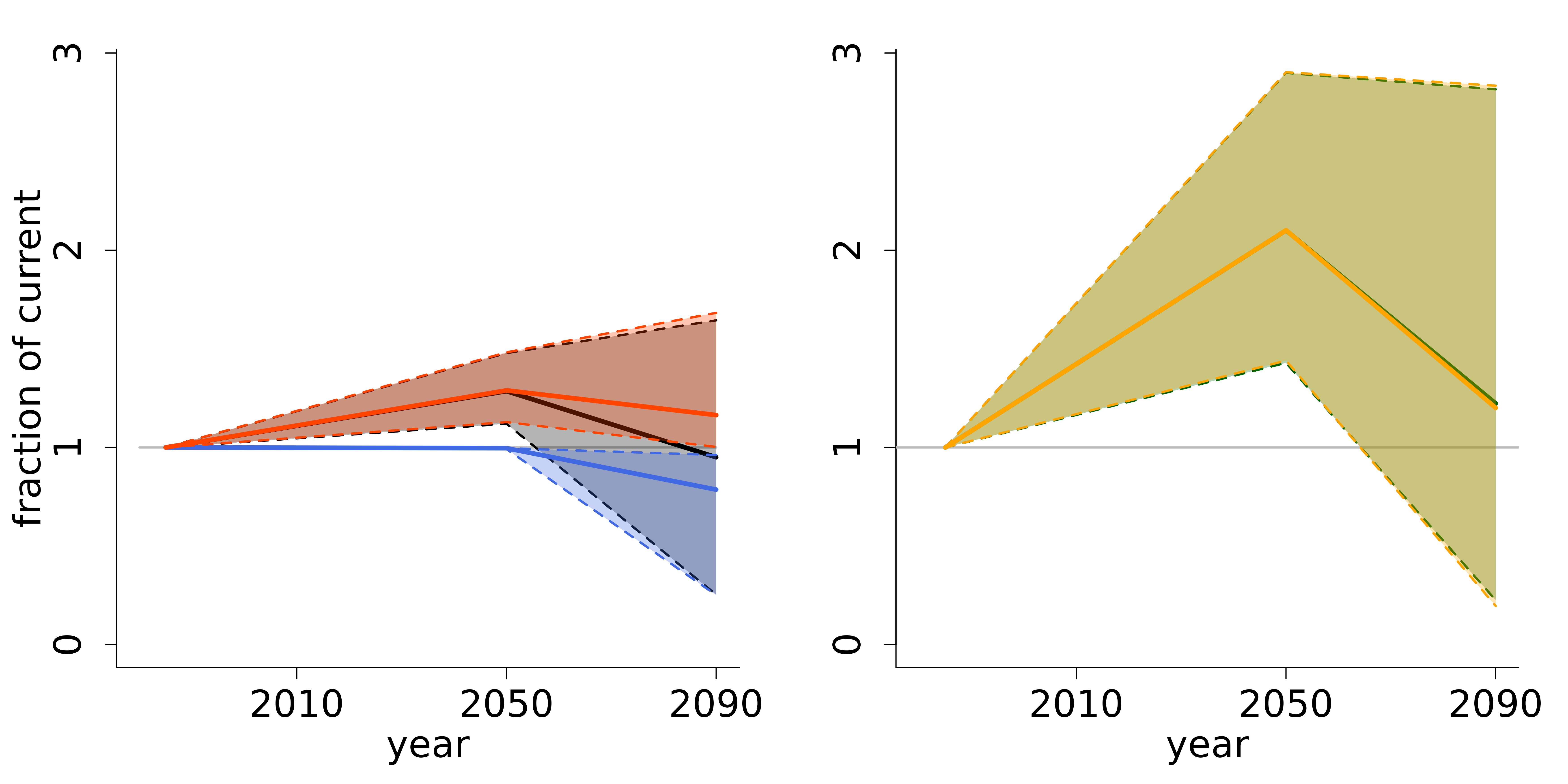

Supplement: S3 Appendix — (ZIP) [file pntd.0014030.s007.zip › Sup. Mat. 6-2 M-Z - Species Trends/Porthidium_porrasi_CCTrends.png]

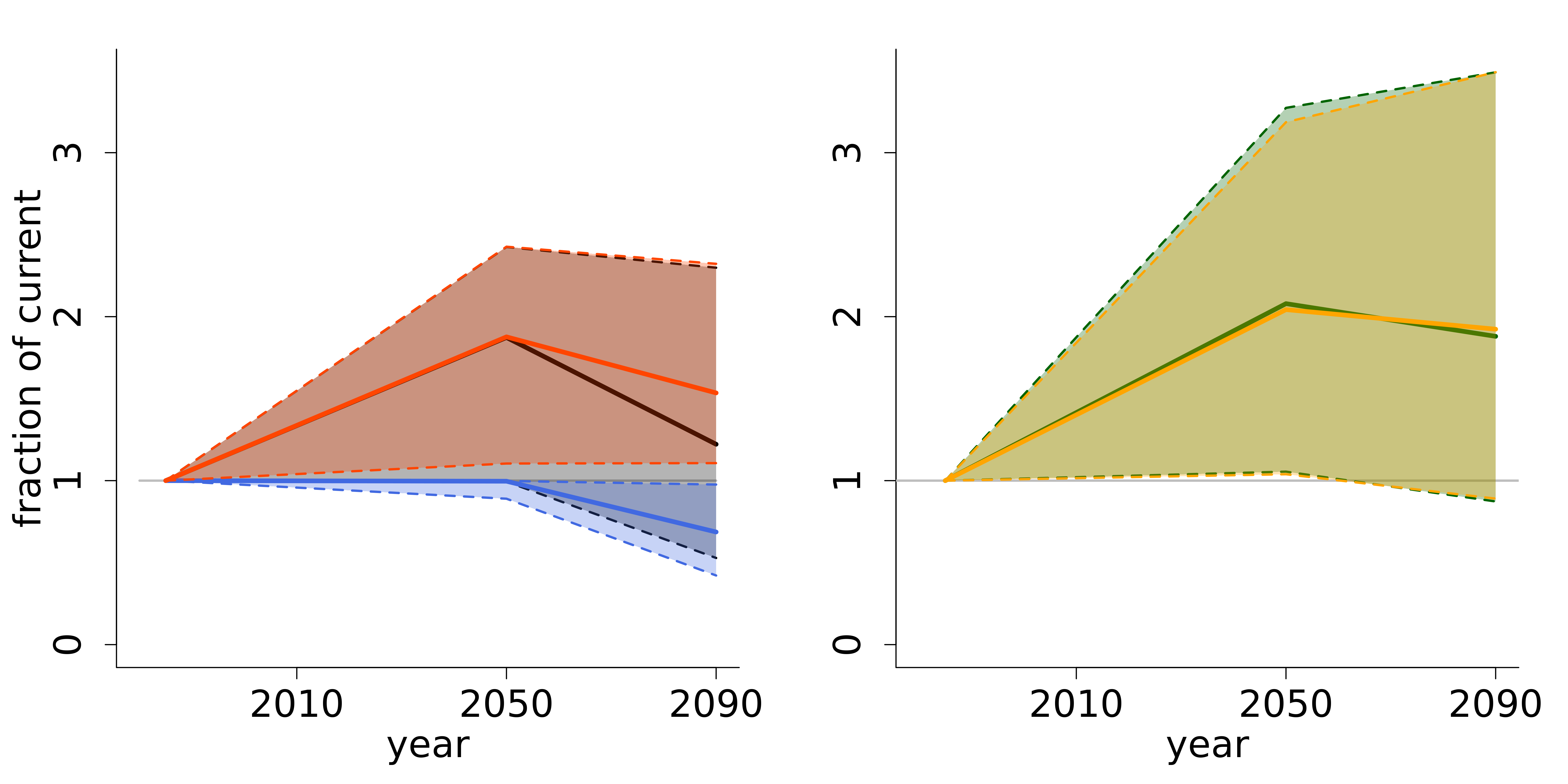

Supplement: S3 Appendix — (ZIP) [file pntd.0014030.s007.zip › Sup. Mat. 6-2 M-Z - Species Trends/Porthidium_volcanicum_CCTrends.png]

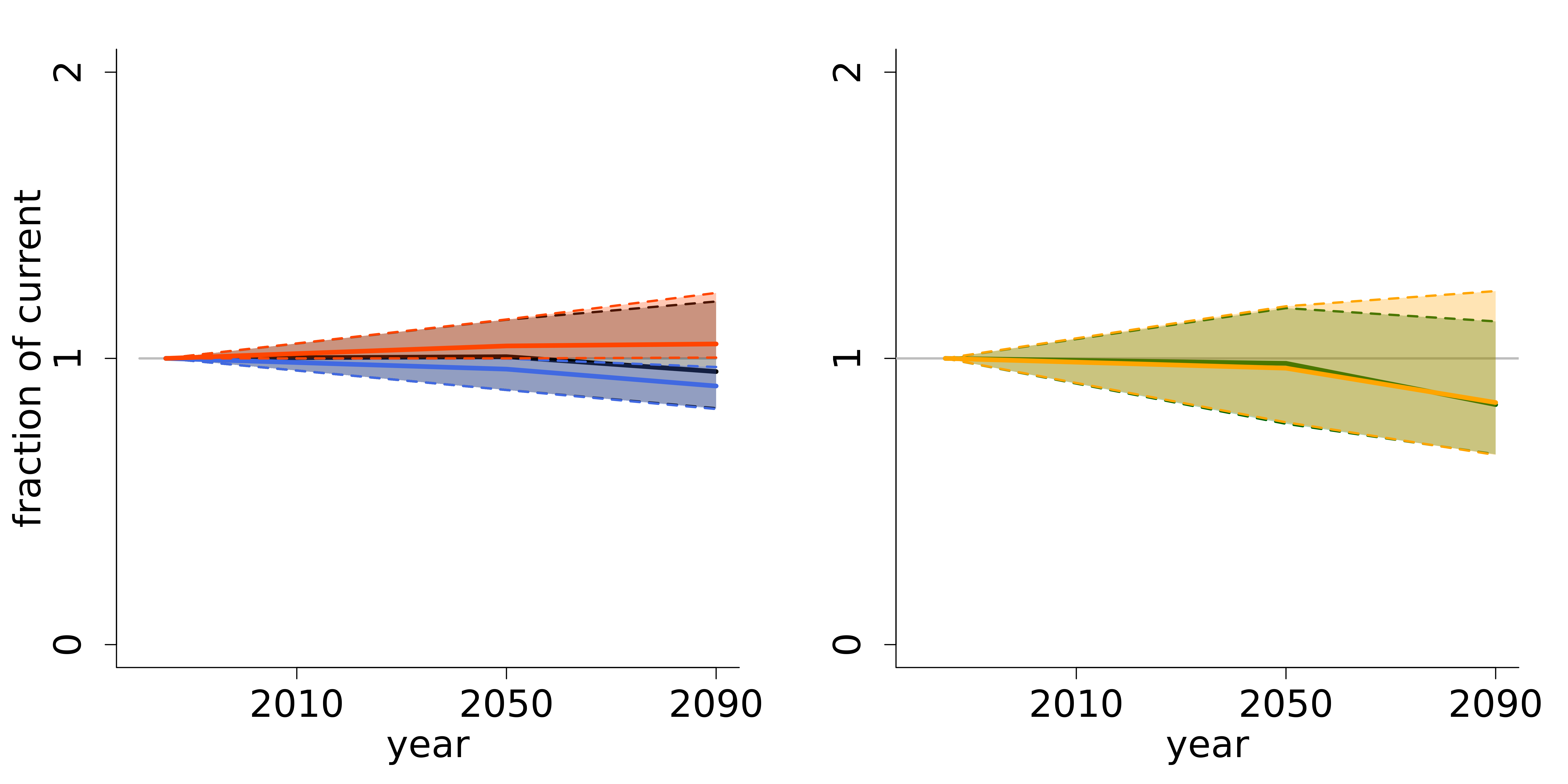

Supplement: S3 Appendix — (ZIP) [file pntd.0014030.s007.zip › Sup. Mat. 6-2 M-Z - Species Trends/Porthidium_yucatanicum_CCTrends.png]

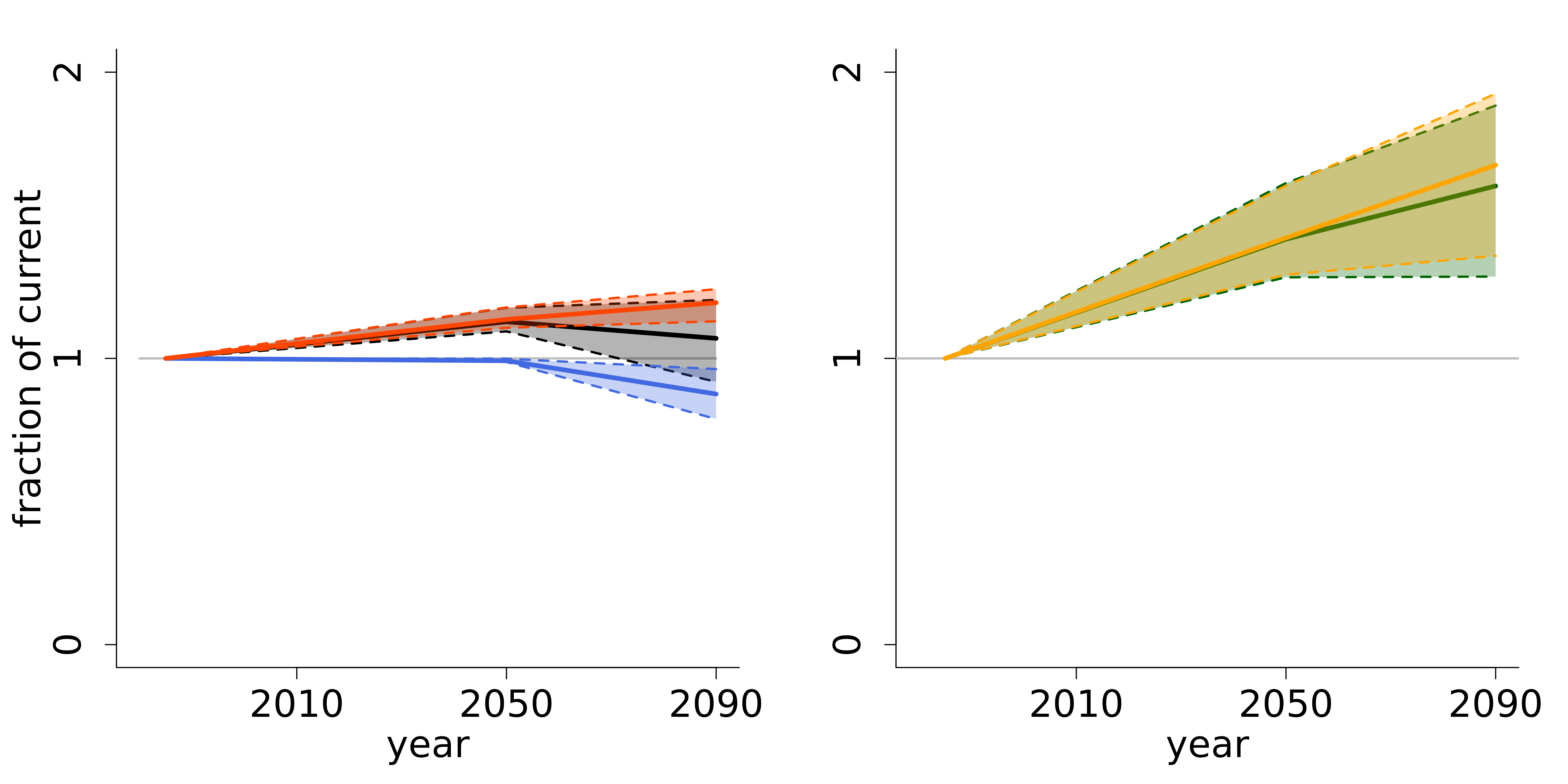

Supplement: S3 Appendix — (ZIP) [file pntd.0014030.s007.zip › Sup. Mat. 6-2 M-Z - Species Trends/Proatheris_superciliaris_CCTrends.png]

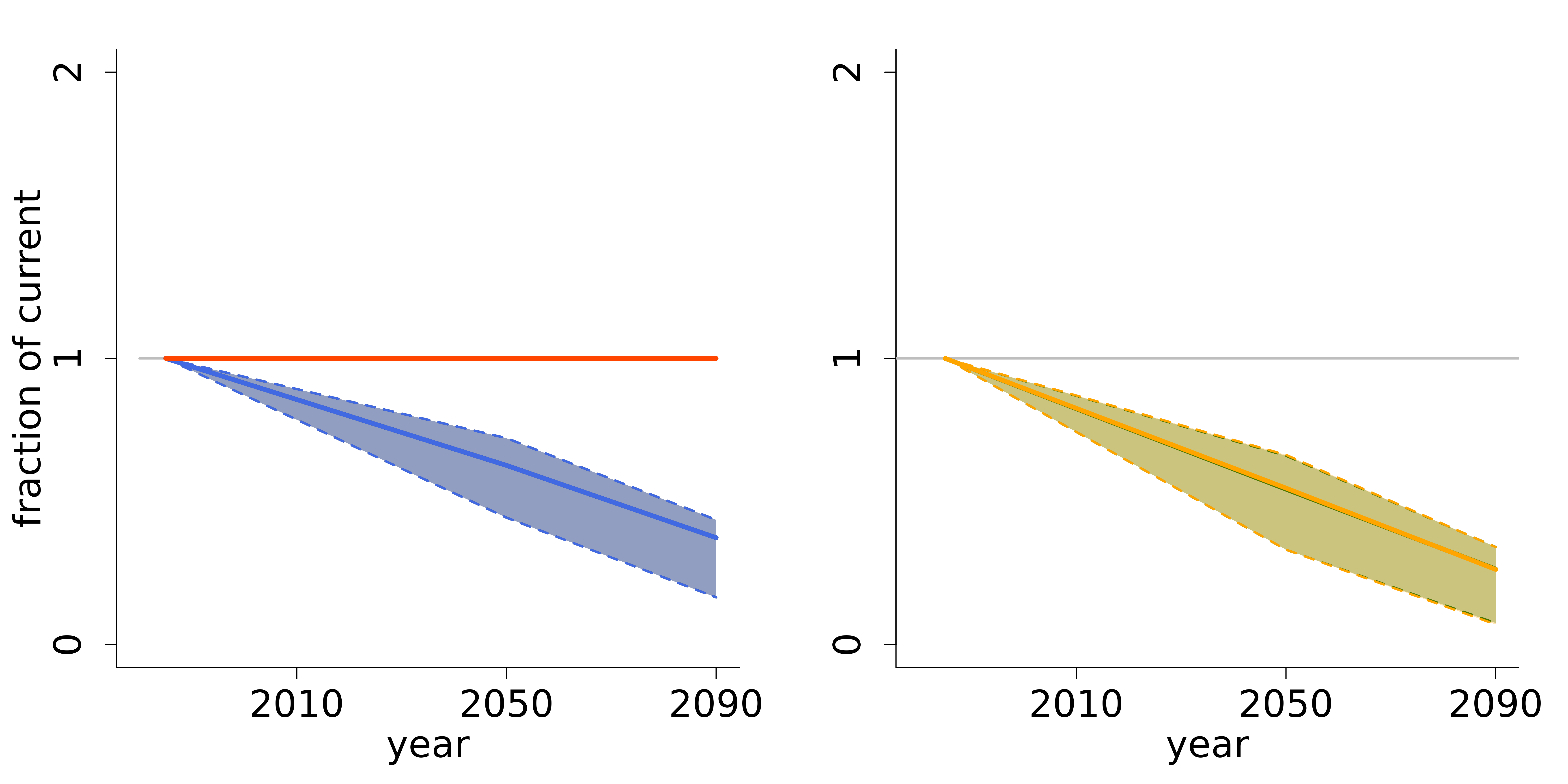

Supplement: S3 Appendix — (ZIP) [file pntd.0014030.s007.zip › Sup. Mat. 6-2 M-Z - Species Trends/Protobothrops_cornutus_CCTrends.png]

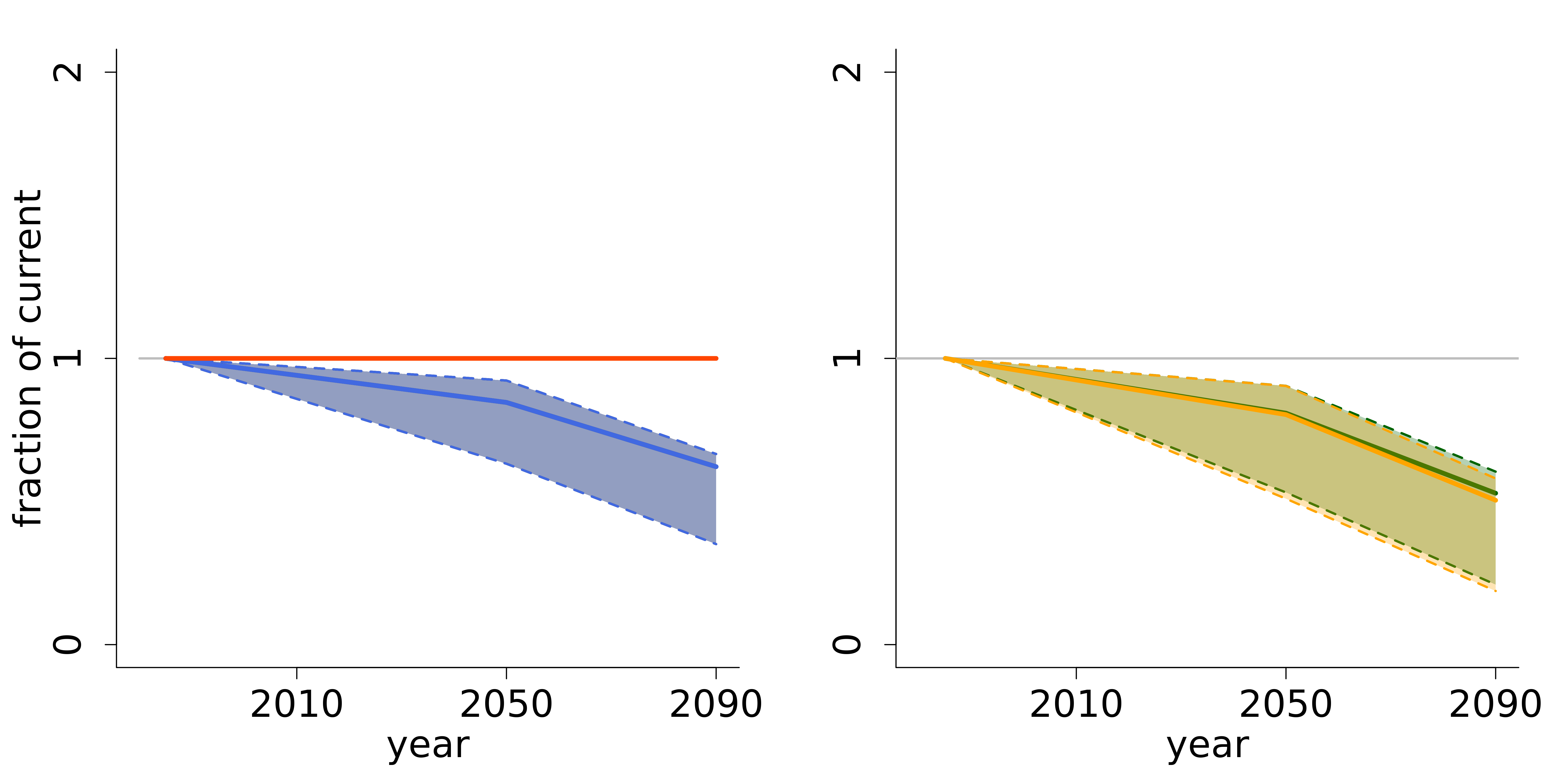

Supplement: S3 Appendix — (ZIP) [file pntd.0014030.s007.zip › Sup. Mat. 6-2 M-Z - Species Trends/Protobothrops_dabieshanensis_CCTrends.png]

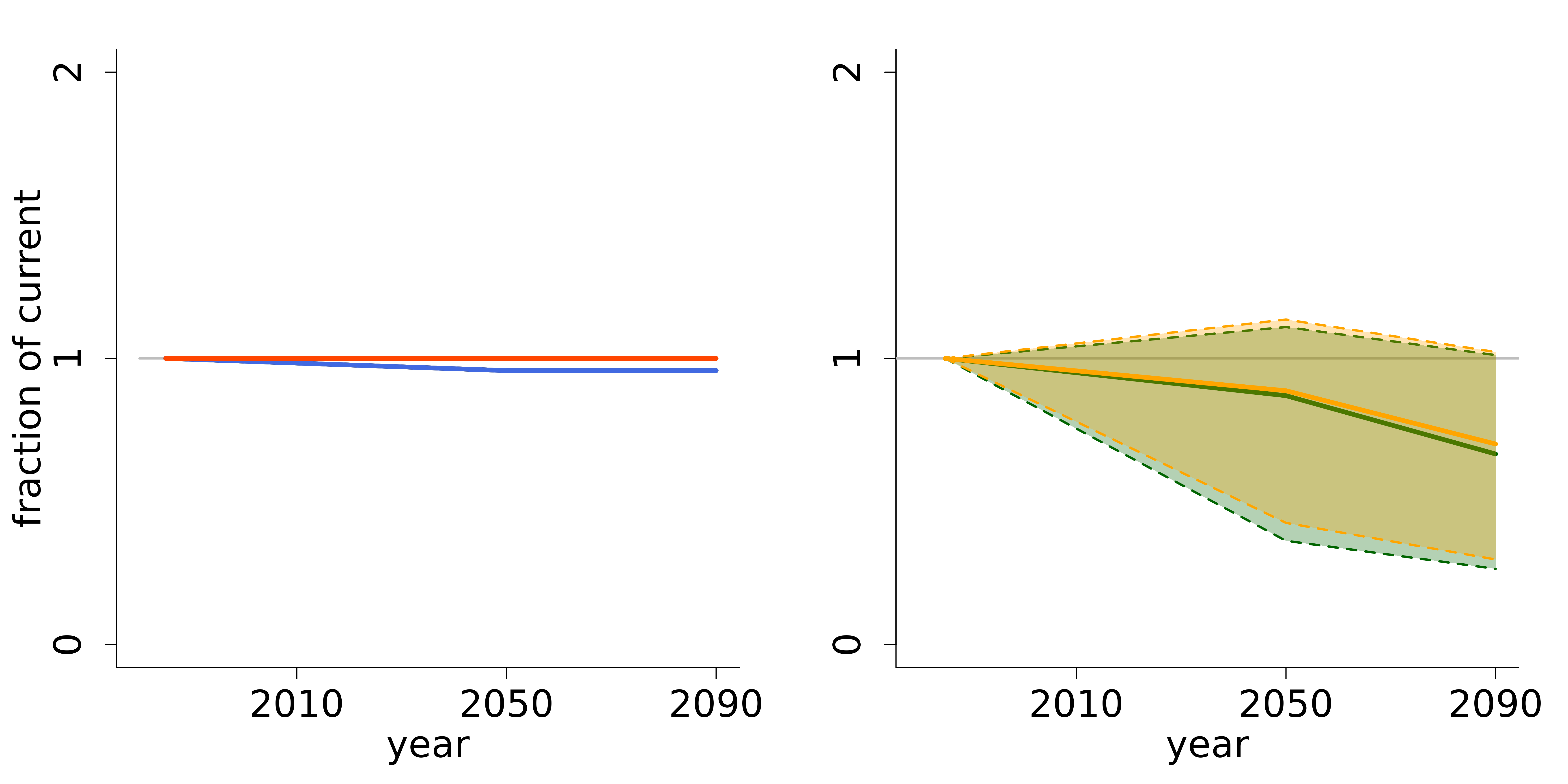

Supplement: S3 Appendix — (ZIP) [file pntd.0014030.s007.zip › Sup. Mat. 6-2 M-Z - Species Trends/Protobothrops_elegans_CCTrends.png]

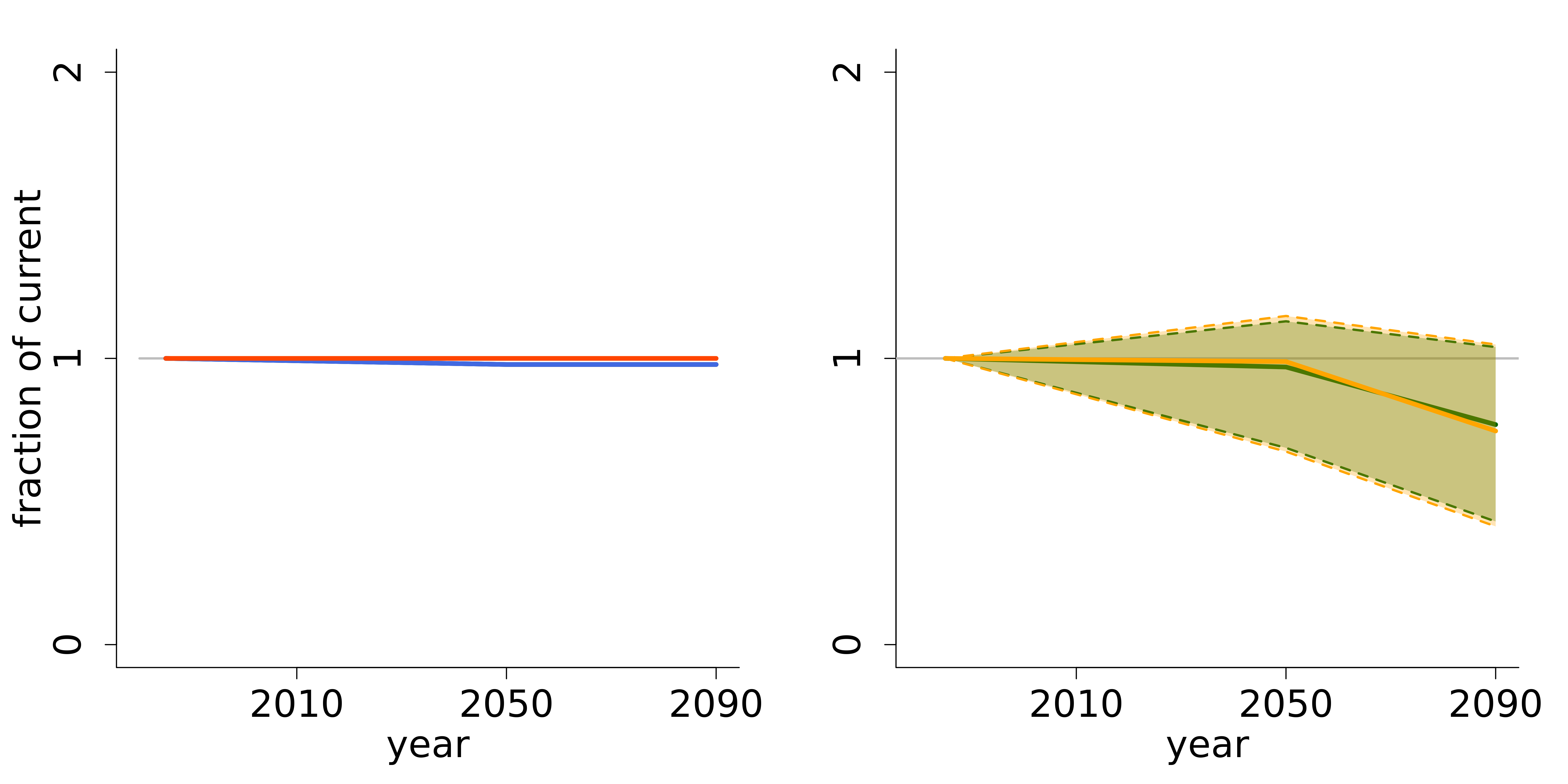

Supplement: S3 Appendix — (ZIP) [file pntd.0014030.s007.zip › Sup. Mat. 6-2 M-Z - Species Trends/Protobothrops_flavoviridis_CCTrends.png]

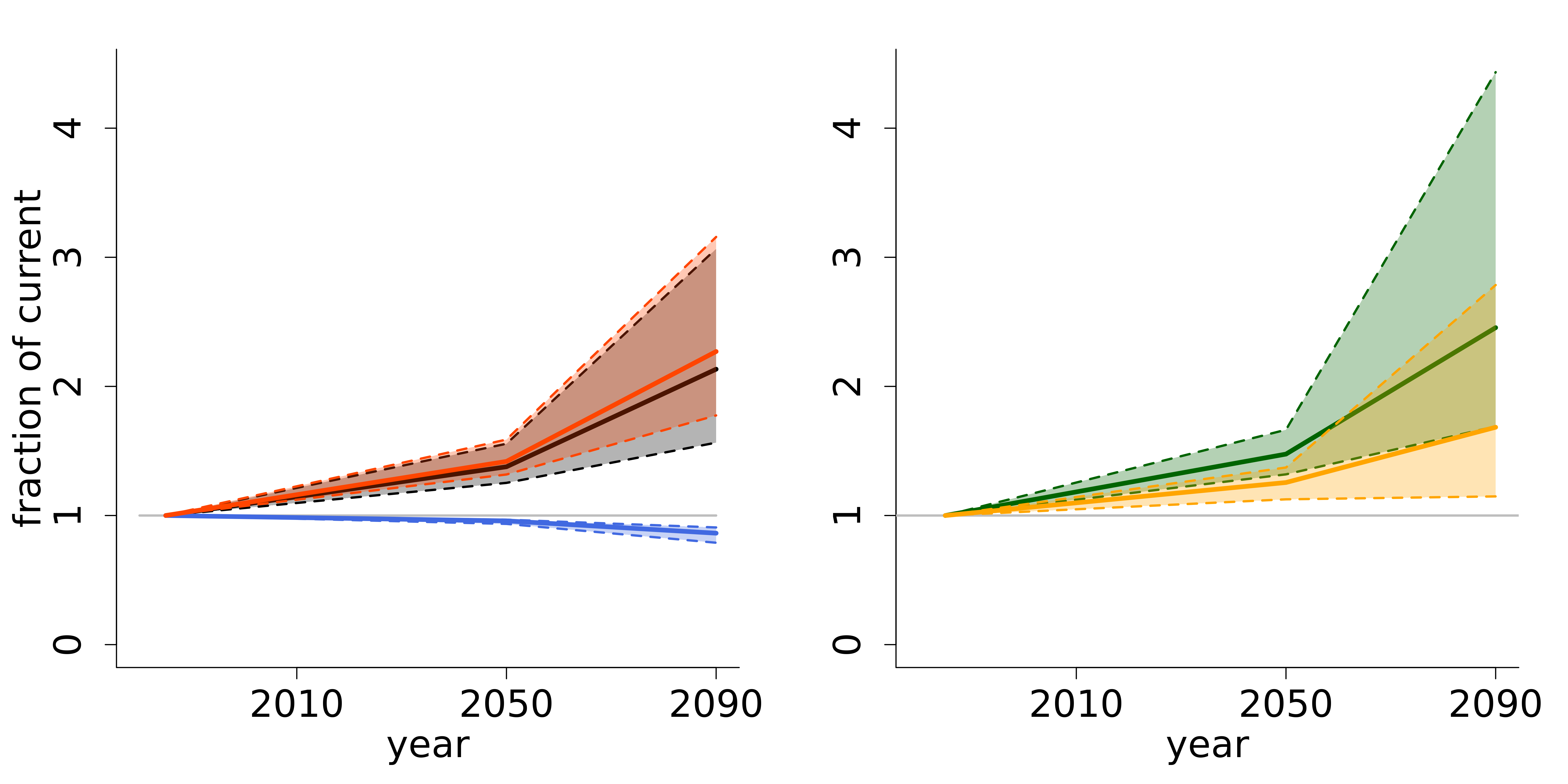

Supplement: S3 Appendix — (ZIP) [file pntd.0014030.s007.zip › Sup. Mat. 6-2 M-Z - Species Trends/Protobothrops_himalayanus_CCTrends.png]

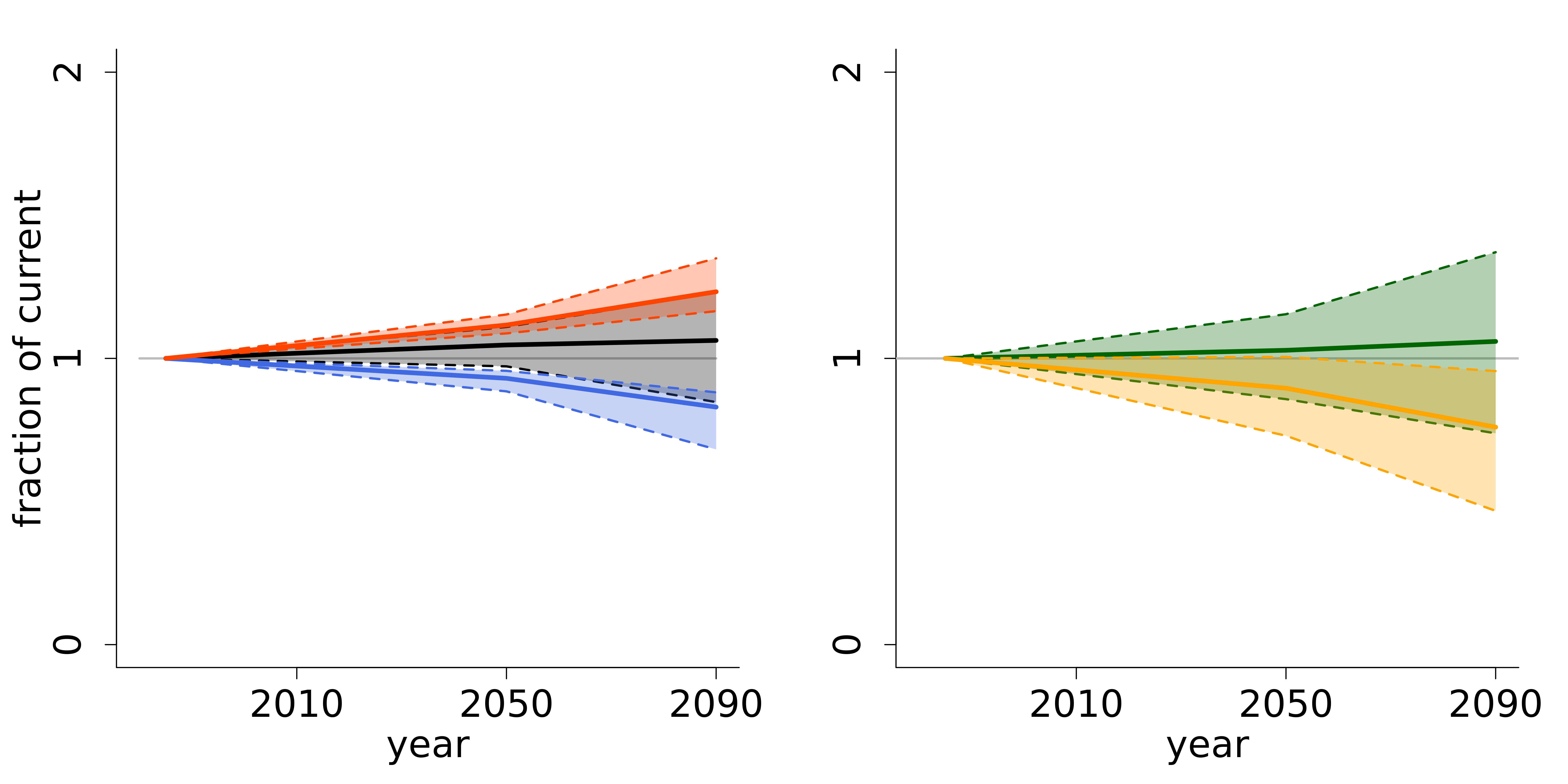

Supplement: S3 Appendix — (ZIP) [file pntd.0014030.s007.zip › Sup. Mat. 6-2 M-Z - Species Trends/Protobothrops_jerdonii_CCTrends.png]

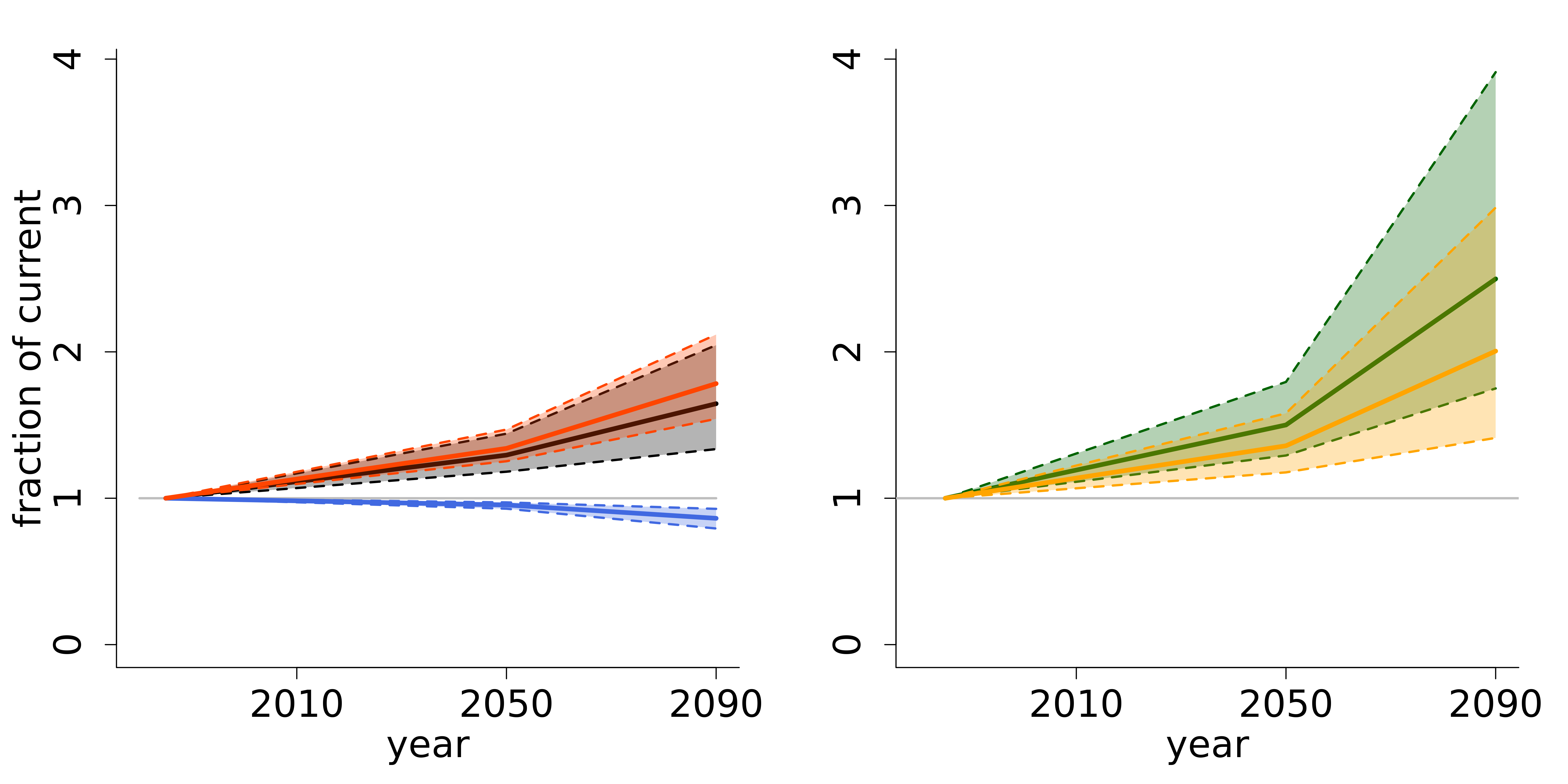

Supplement: S3 Appendix — (ZIP) [file pntd.0014030.s007.zip › Sup. Mat. 6-2 M-Z - Species Trends/Protobothrops_kaulbacki_CCTrends.png]

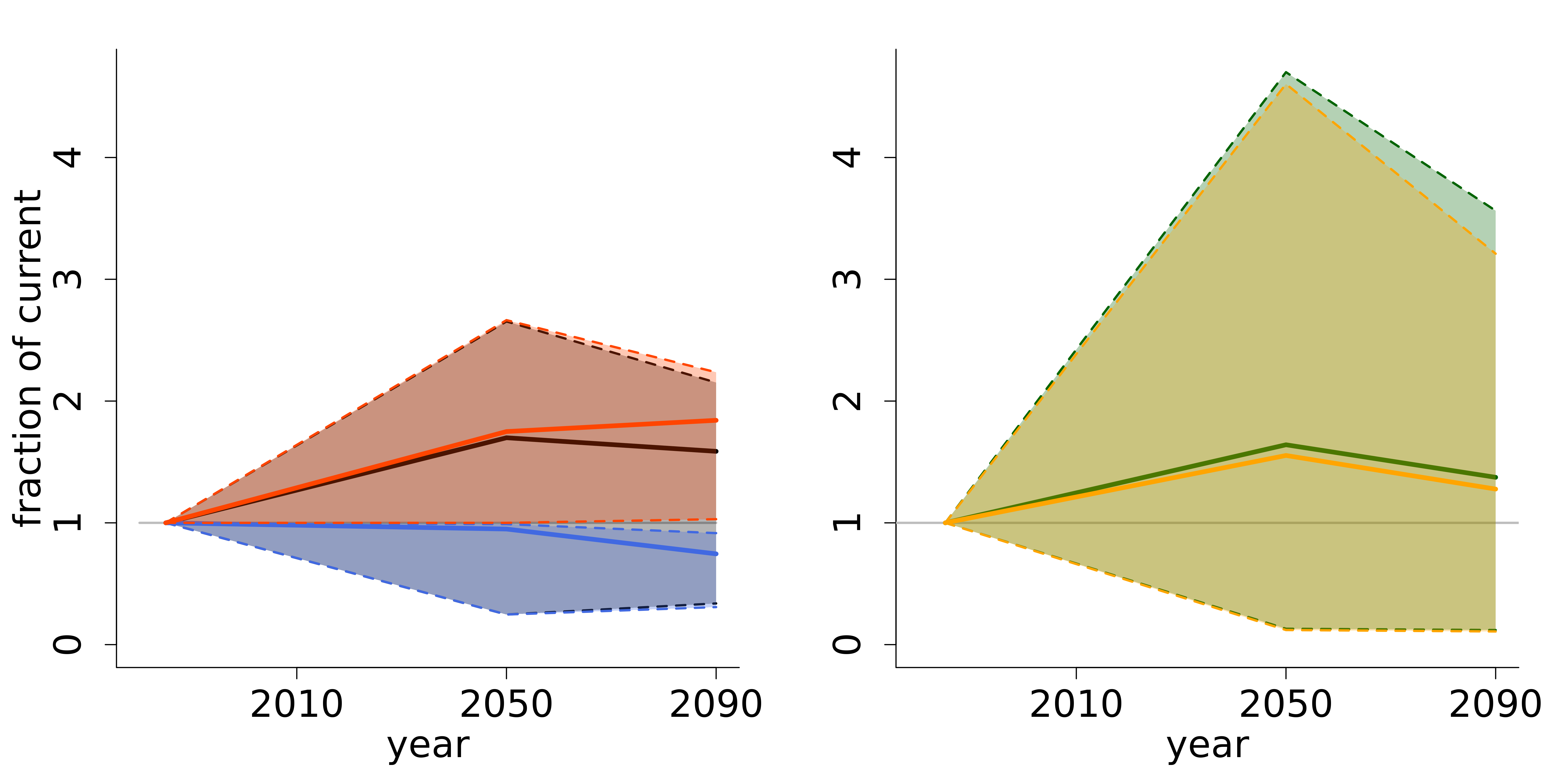

Supplement: S3 Appendix — (ZIP) [file pntd.0014030.s007.zip › Sup. Mat. 6-2 M-Z - Species Trends/Protobothrops_kelomohy_CCTrends.png]

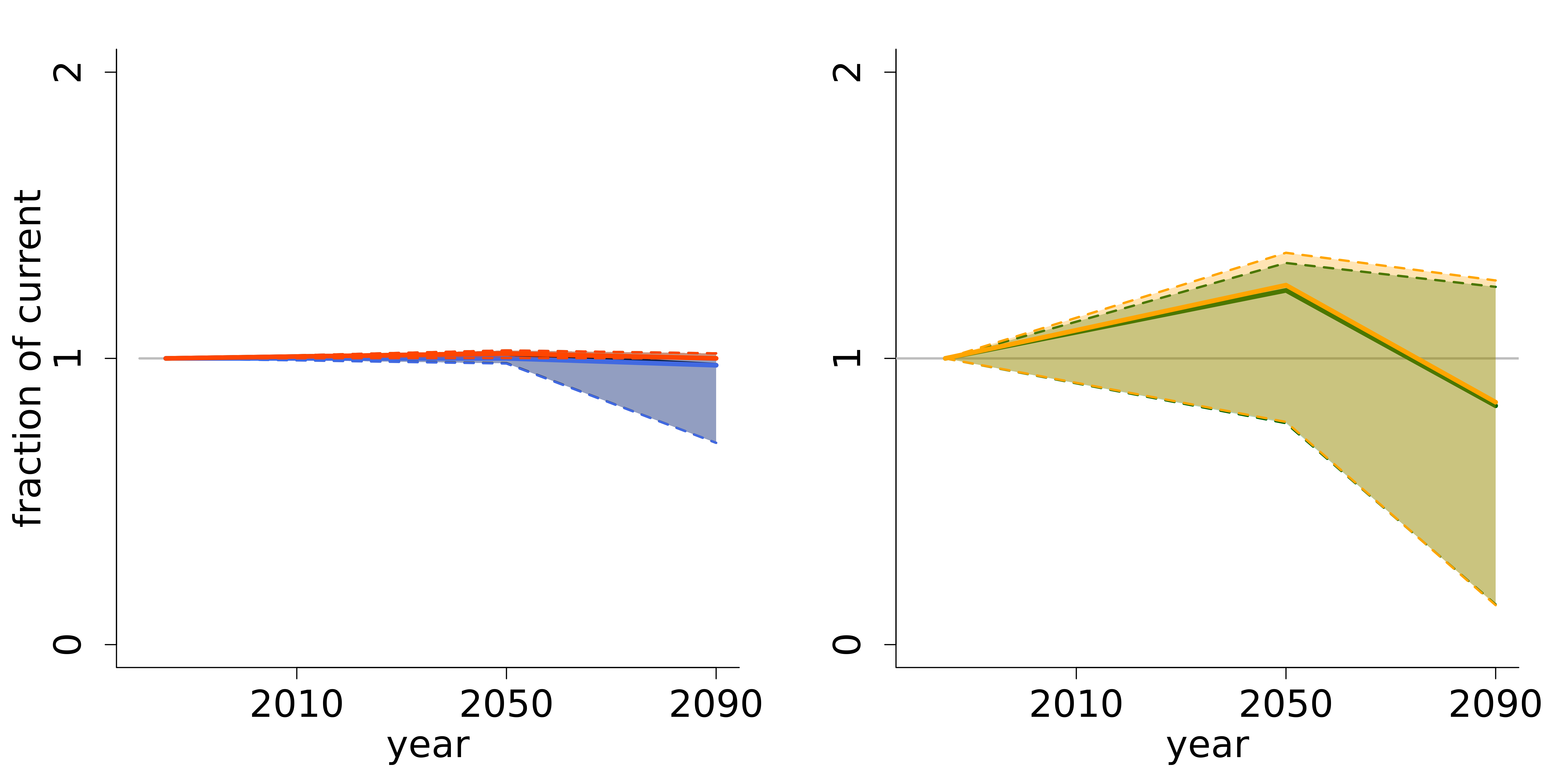

Supplement: S3 Appendix — (ZIP) [file pntd.0014030.s007.zip › Sup. Mat. 6-2 M-Z - Species Trends/Protobothrops_mangshanensis_CCTrends.png]

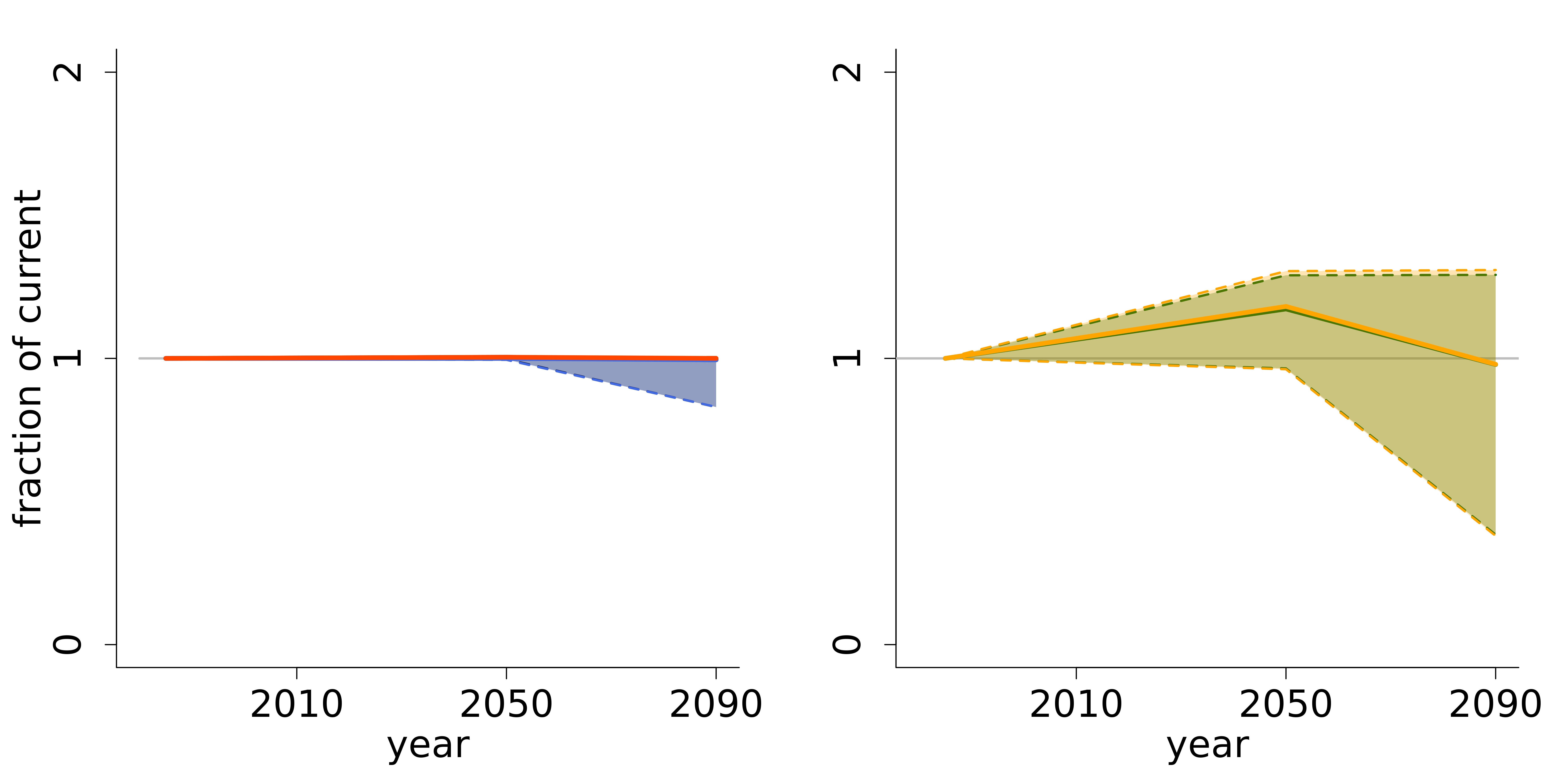

Supplement: S3 Appendix — (ZIP) [file pntd.0014030.s007.zip › Sup. Mat. 6-2 M-Z - Species Trends/Protobothrops_maolanensis_CCTrends.png]

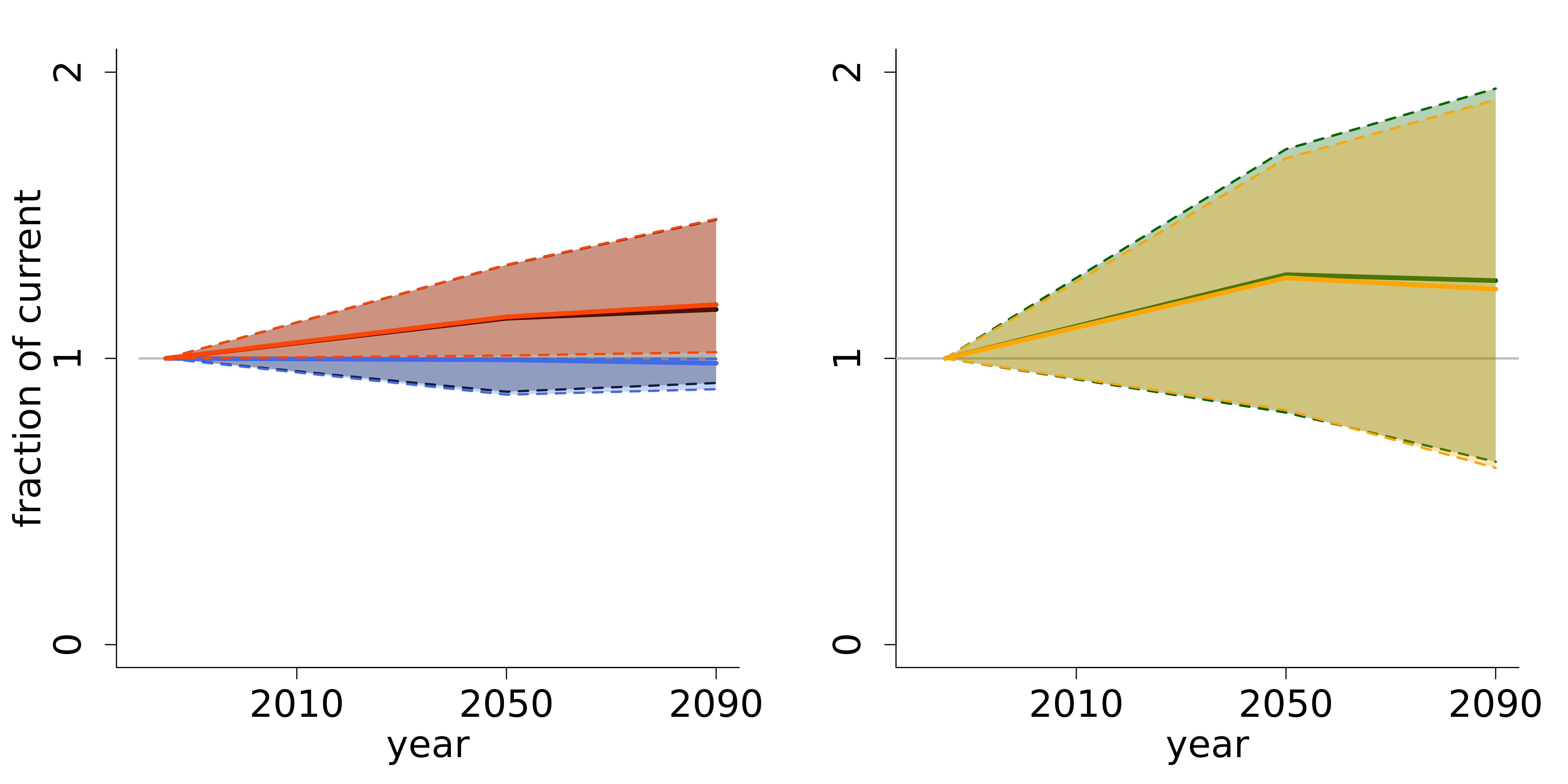

Supplement: S3 Appendix — (ZIP) [file pntd.0014030.s007.zip › Sup. Mat. 6-2 M-Z - Species Trends/Protobothrops_mucrosquamatus_CCTrends.png]

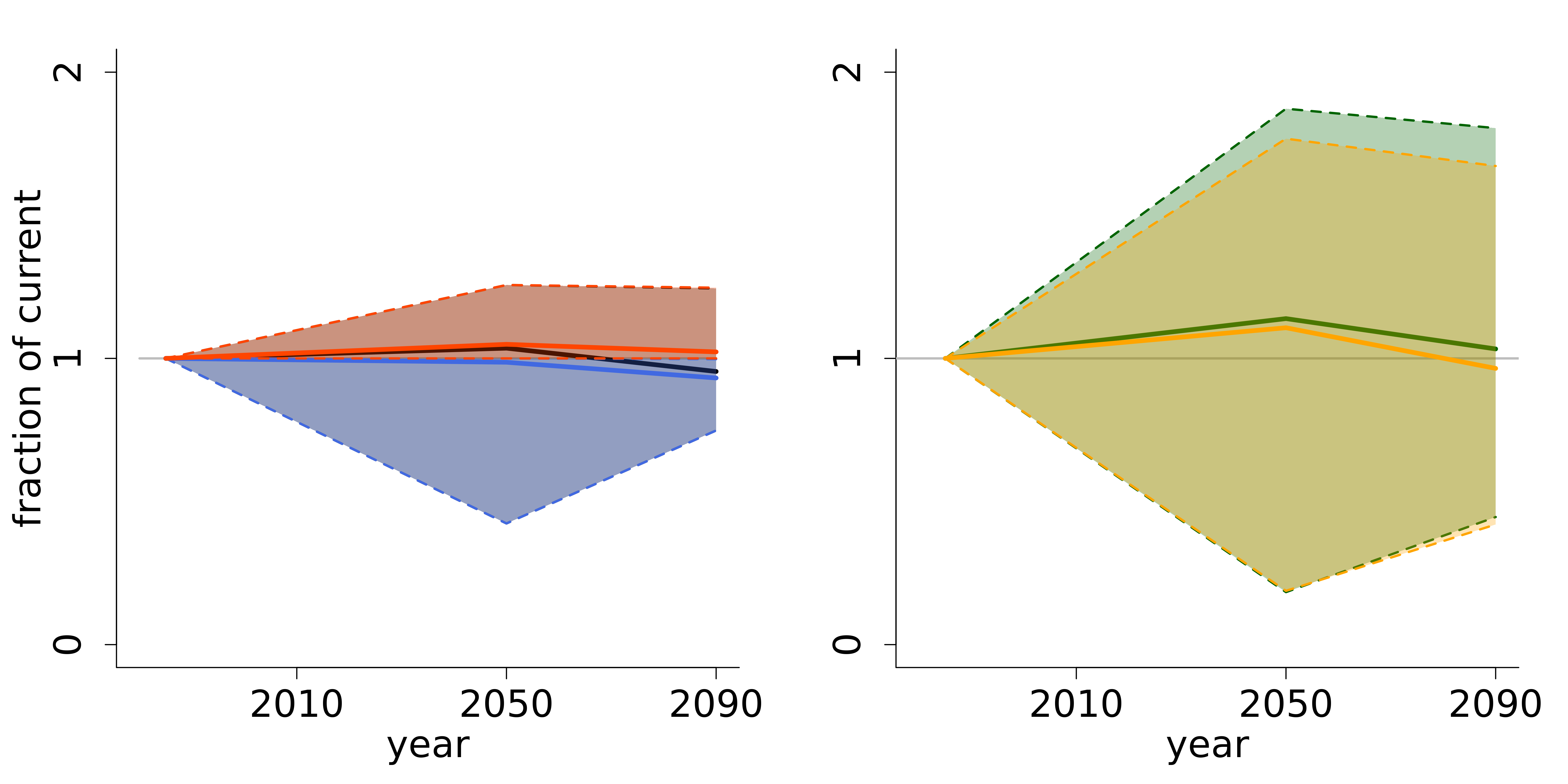

Supplement: S3 Appendix — (ZIP) [file pntd.0014030.s007.zip › Sup. Mat. 6-2 M-Z - Species Trends/Protobothrops_sieversorum_CCTrends.png]

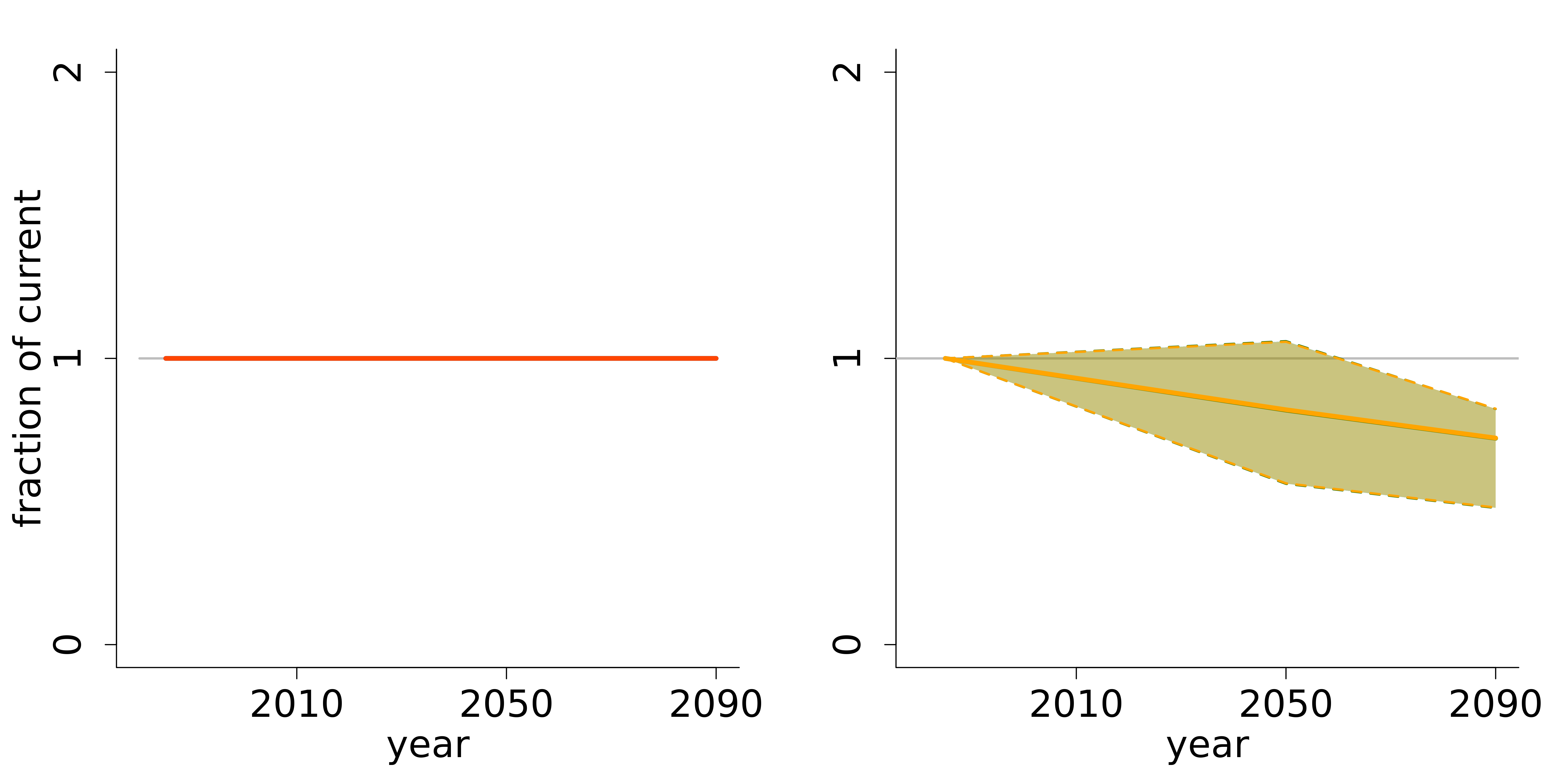

Supplement: S3 Appendix — (ZIP) [file pntd.0014030.s007.zip › Sup. Mat. 6-2 M-Z - Species Trends/Protobothrops_tokarensis_CCTrends.png]

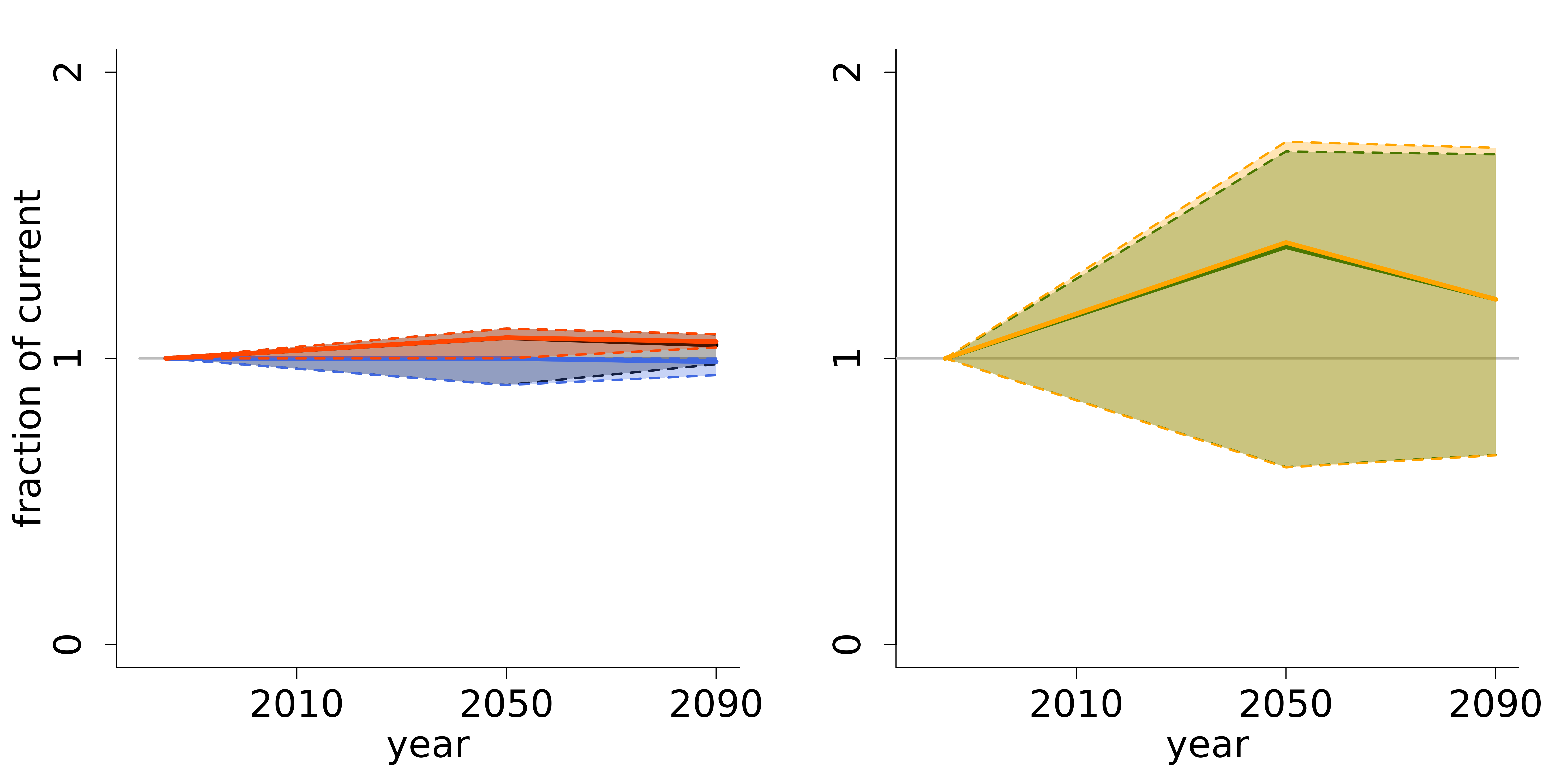

Supplement: S3 Appendix — (ZIP) [file pntd.0014030.s007.zip › Sup. Mat. 6-2 M-Z - Species Trends/Protobothrops_trungkhanhensis_CCTrends.png]

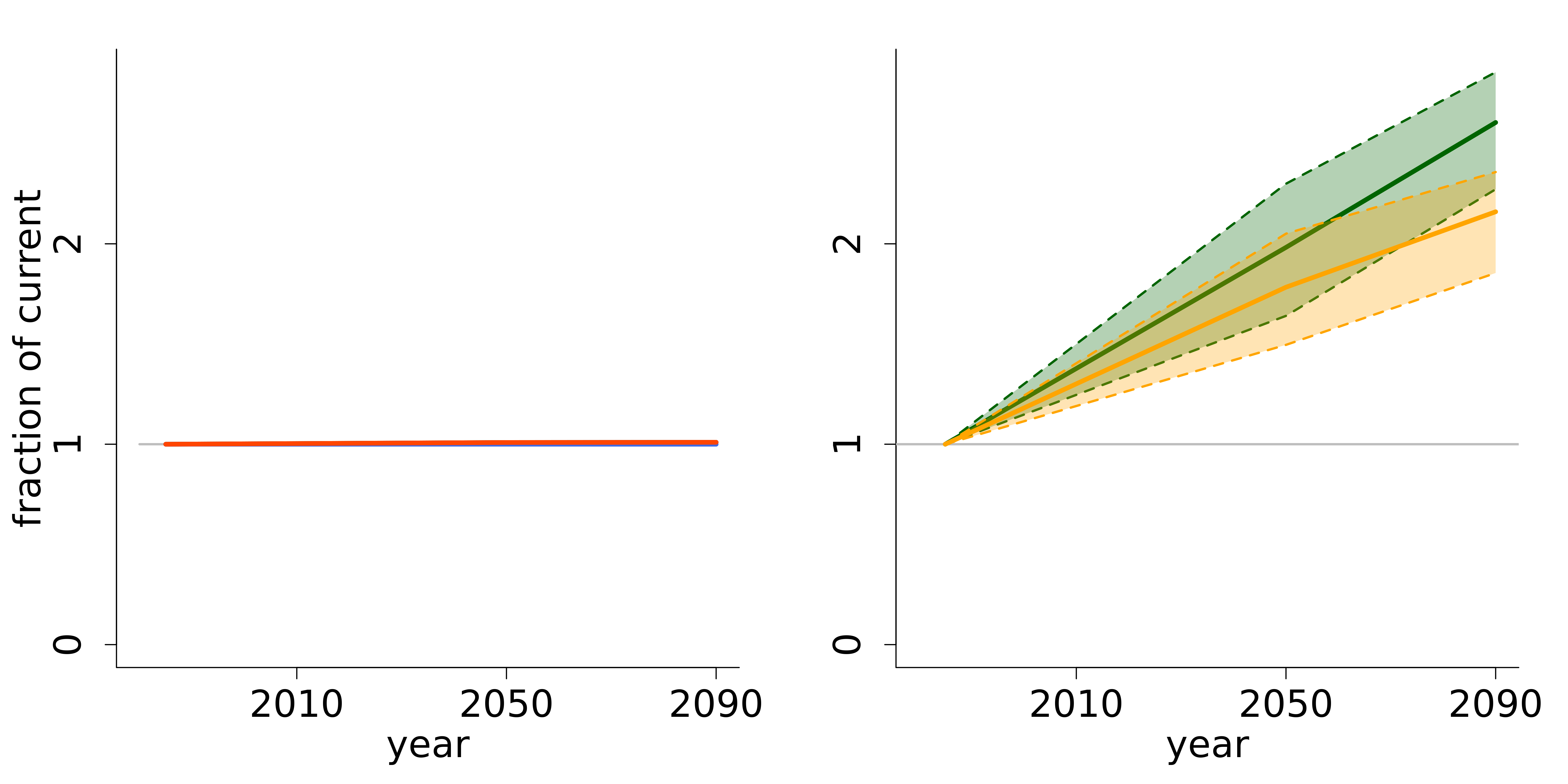

Supplement: S3 Appendix — (ZIP) [file pntd.0014030.s007.zip › Sup. Mat. 6-2 M-Z - Species Trends/Protobothrops_xiangchengensis_CCTrends.png]

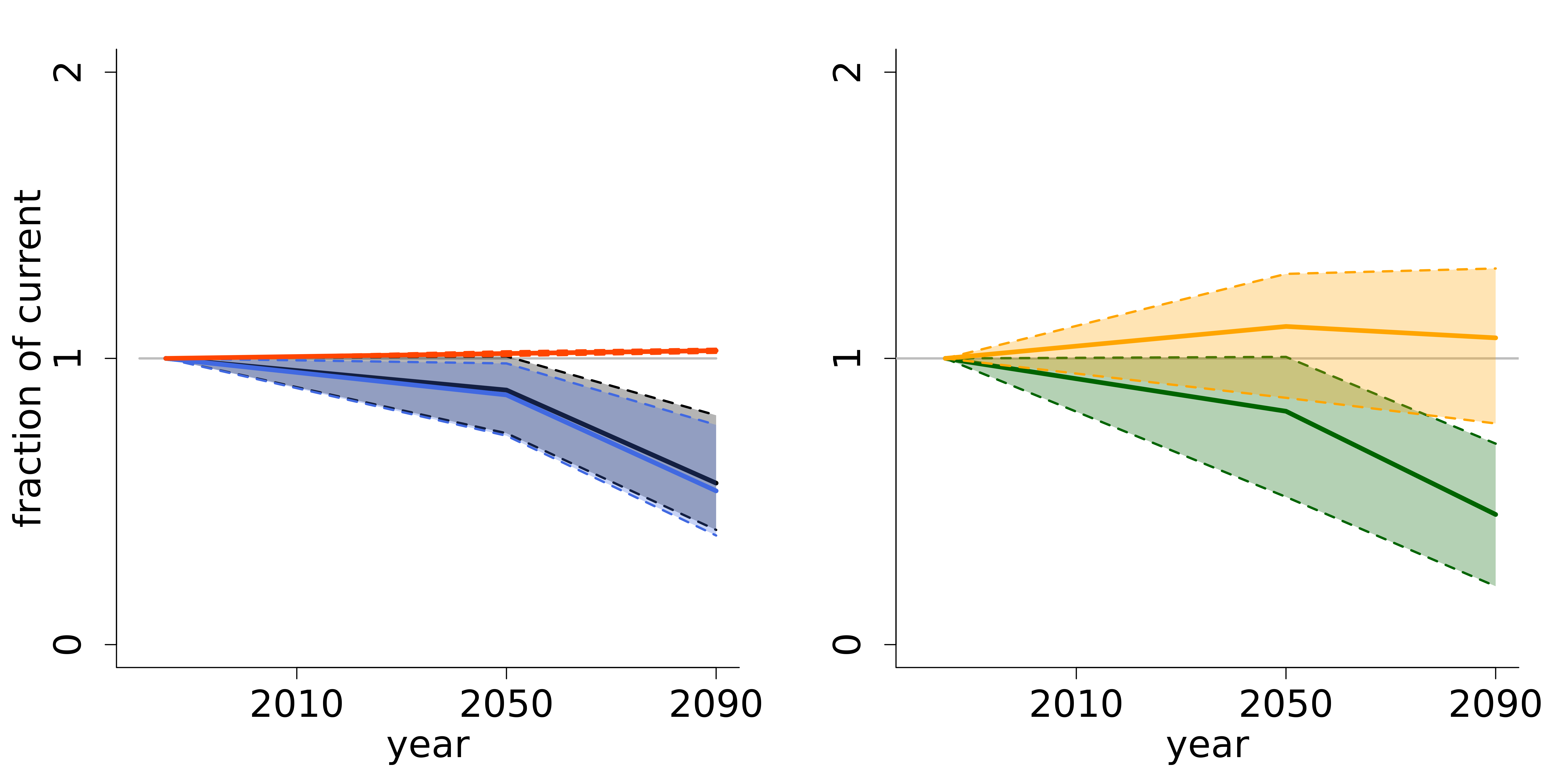

Supplement: S3 Appendix — (ZIP) [file pntd.0014030.s007.zip › Sup. Mat. 6-2 M-Z - Species Trends/Pseudechis_australis_CCTrends.png]

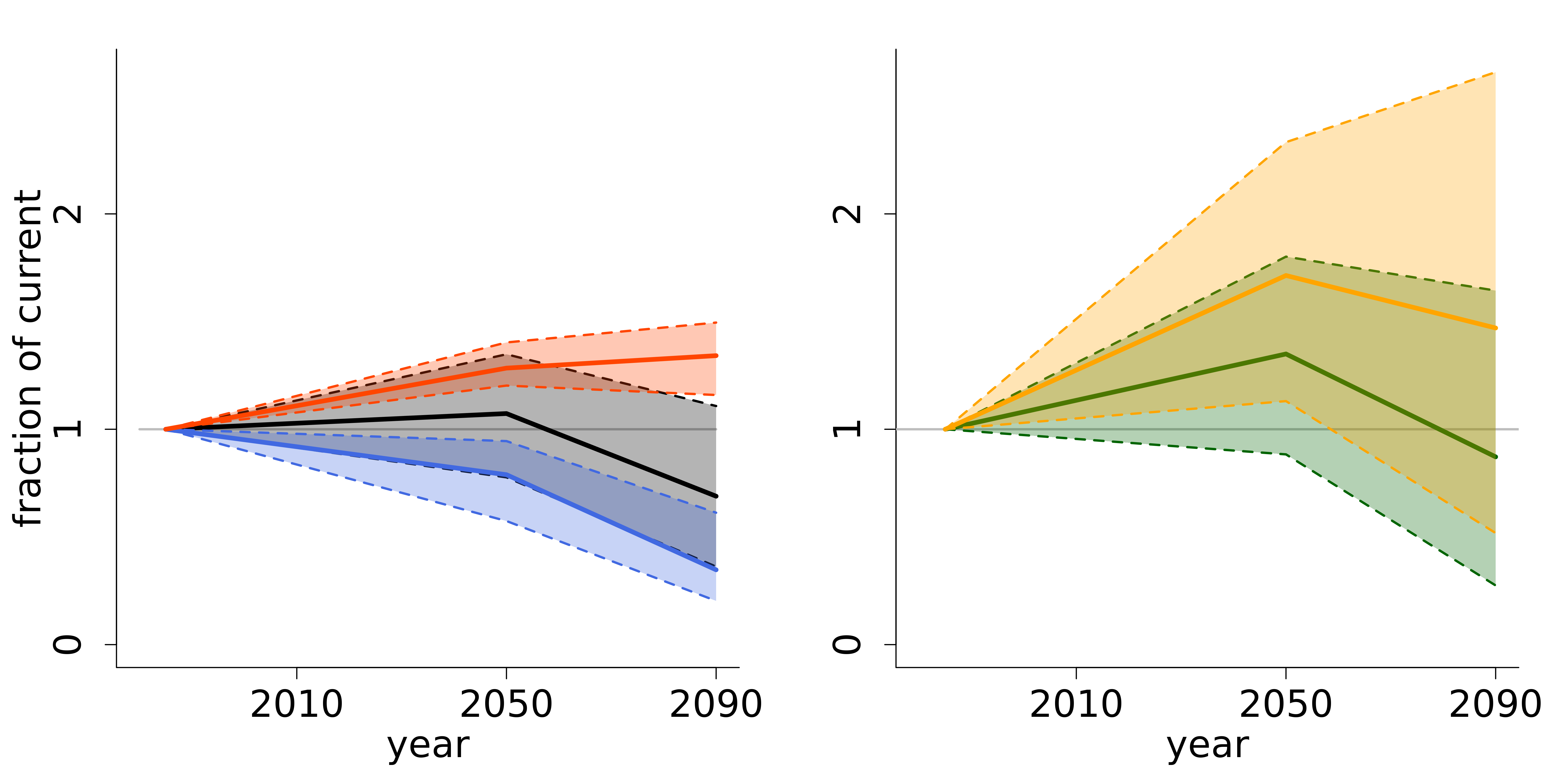

Supplement: S3 Appendix — (ZIP) [file pntd.0014030.s007.zip › Sup. Mat. 6-2 M-Z - Species Trends/Pseudechis_butleri_CCTrends.png]

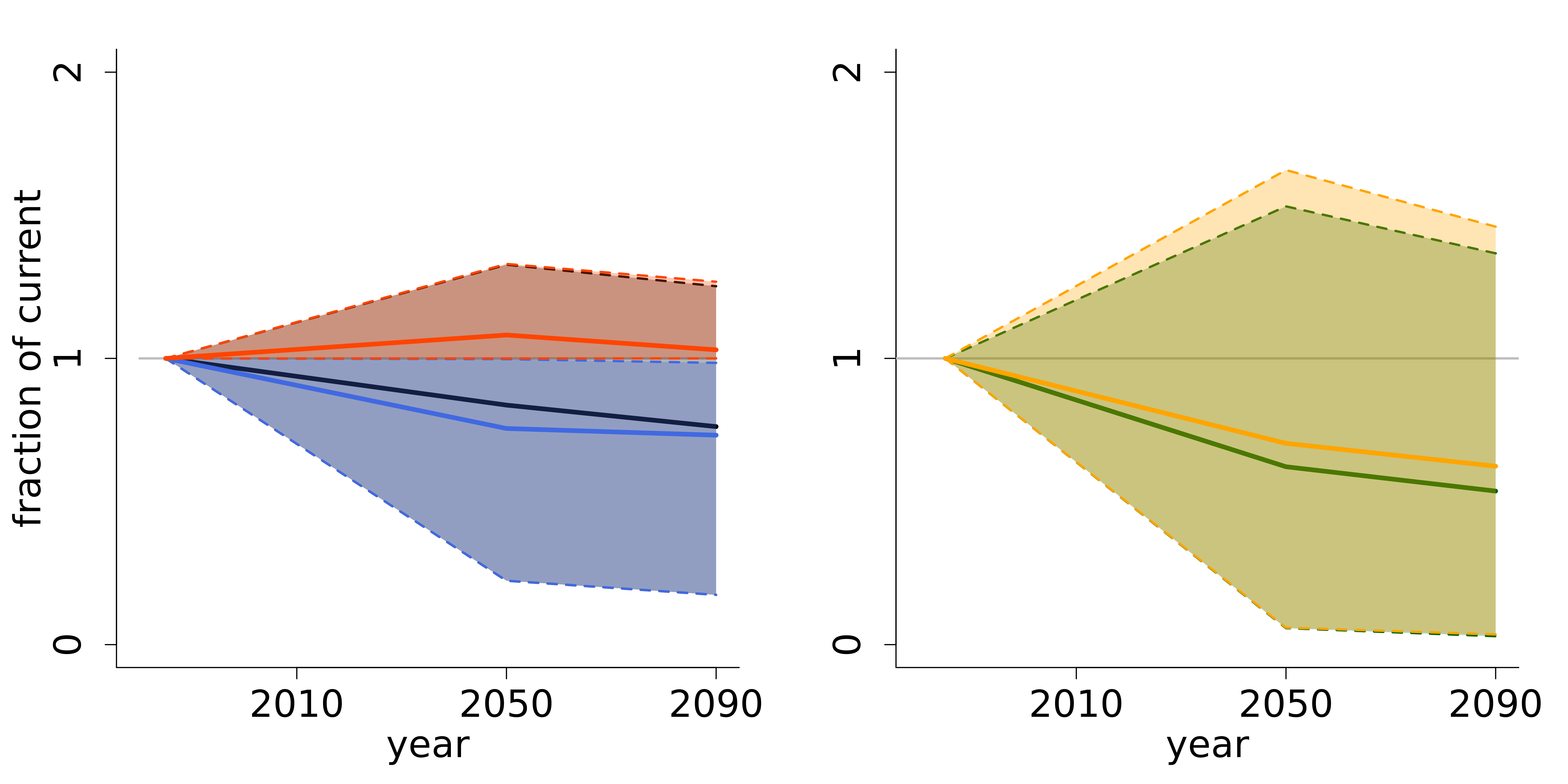

Supplement: S3 Appendix — (ZIP) [file pntd.0014030.s007.zip › Sup. Mat. 6-2 M-Z - Species Trends/Pseudechis_colletti_CCTrends.png]

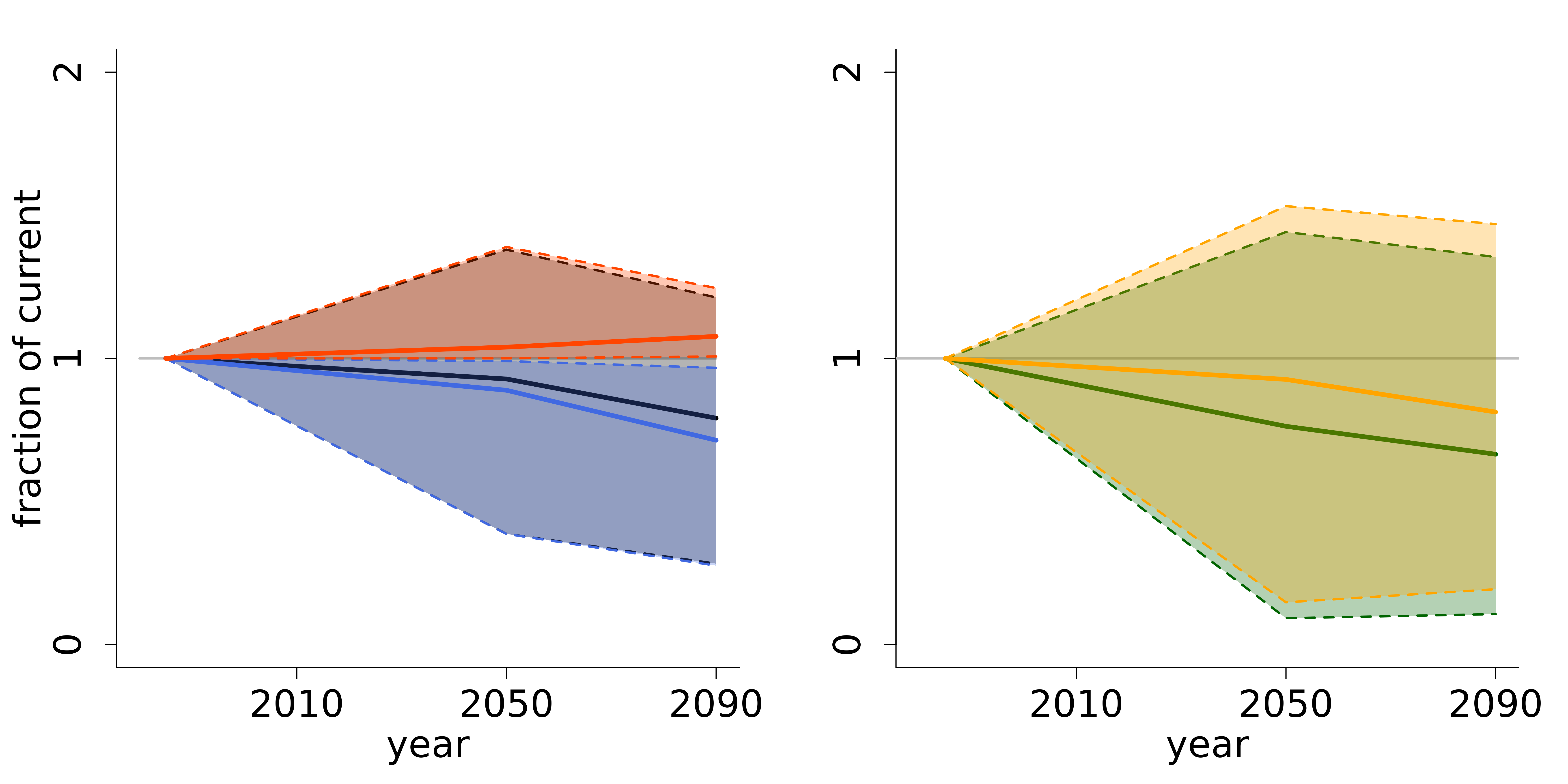

Supplement: S3 Appendix — (ZIP) [file pntd.0014030.s007.zip › Sup. Mat. 6-2 M-Z - Species Trends/Pseudechis_guttatus_CCTrends.png]

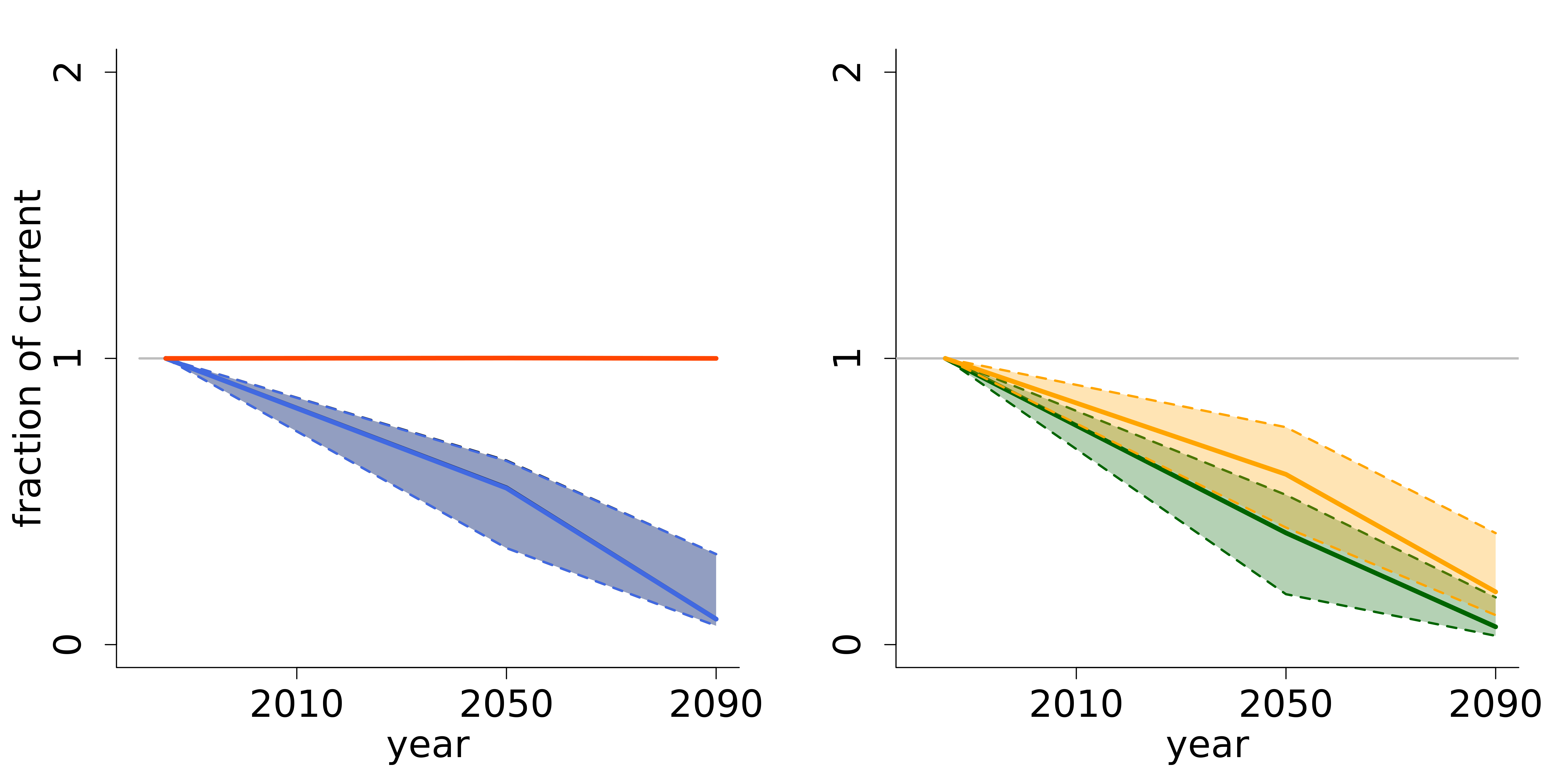

Supplement: S3 Appendix — (ZIP) [file pntd.0014030.s007.zip › Sup. Mat. 6-2 M-Z - Species Trends/Pseudechis_NT_CCTrends.png]

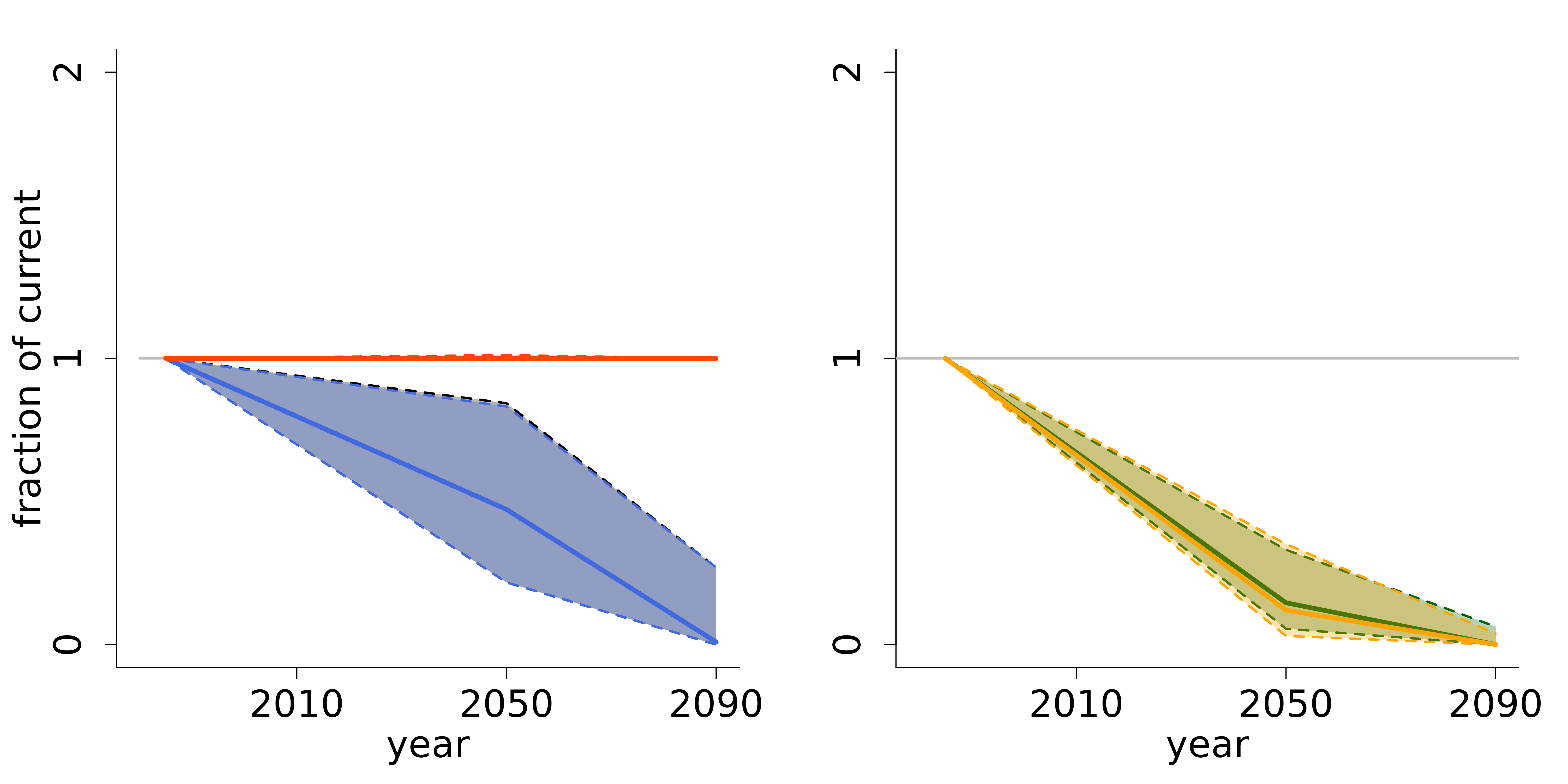

Supplement: S3 Appendix — (ZIP) [file pntd.0014030.s007.zip › Sup. Mat. 6-2 M-Z - Species Trends/Pseudechis_pailsei_CCTrends.png]

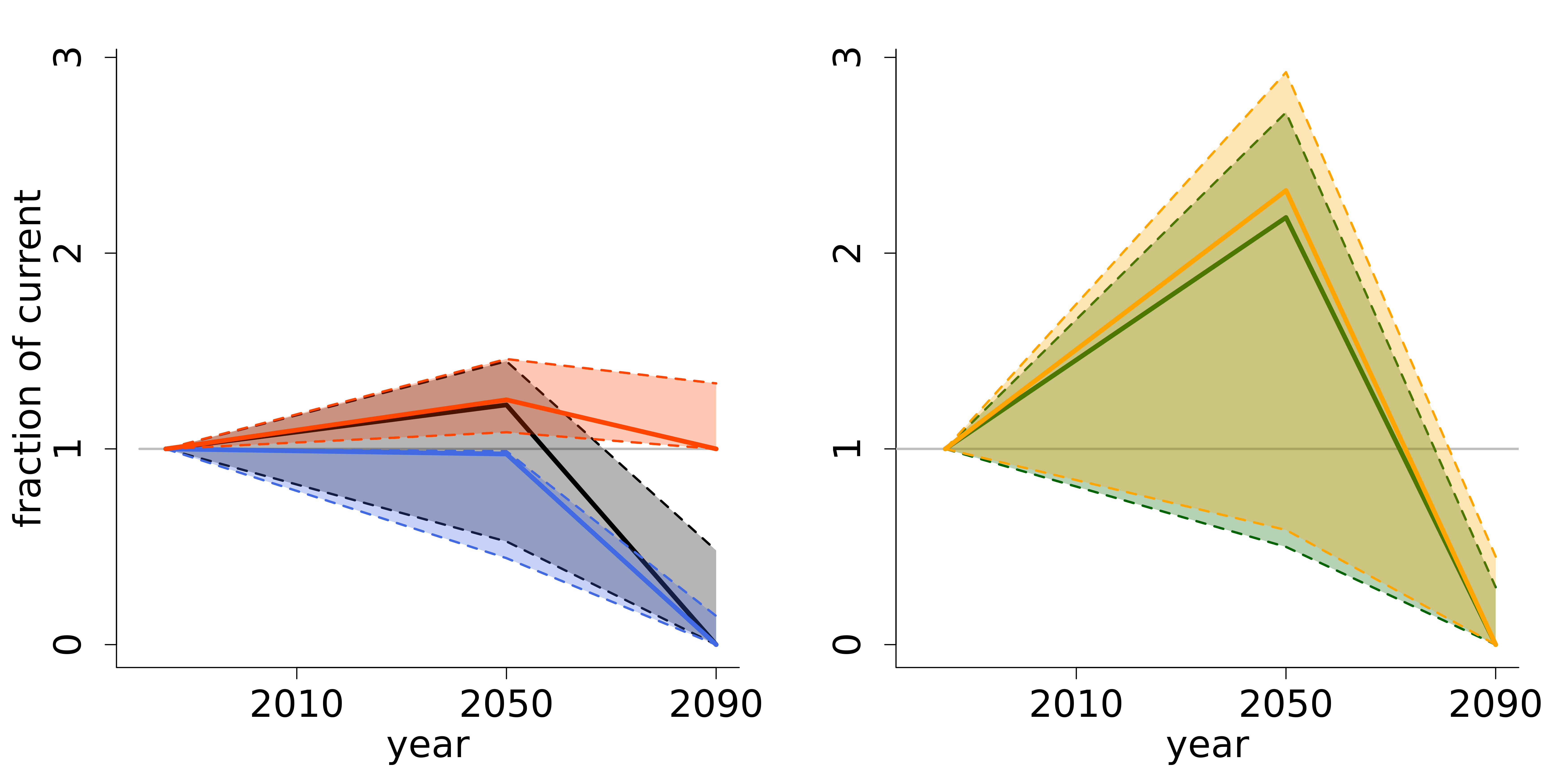

Supplement: S3 Appendix — (ZIP) [file pntd.0014030.s007.zip › Sup. Mat. 6-2 M-Z - Species Trends/Pseudechis_papuanus_CCTrends.png]

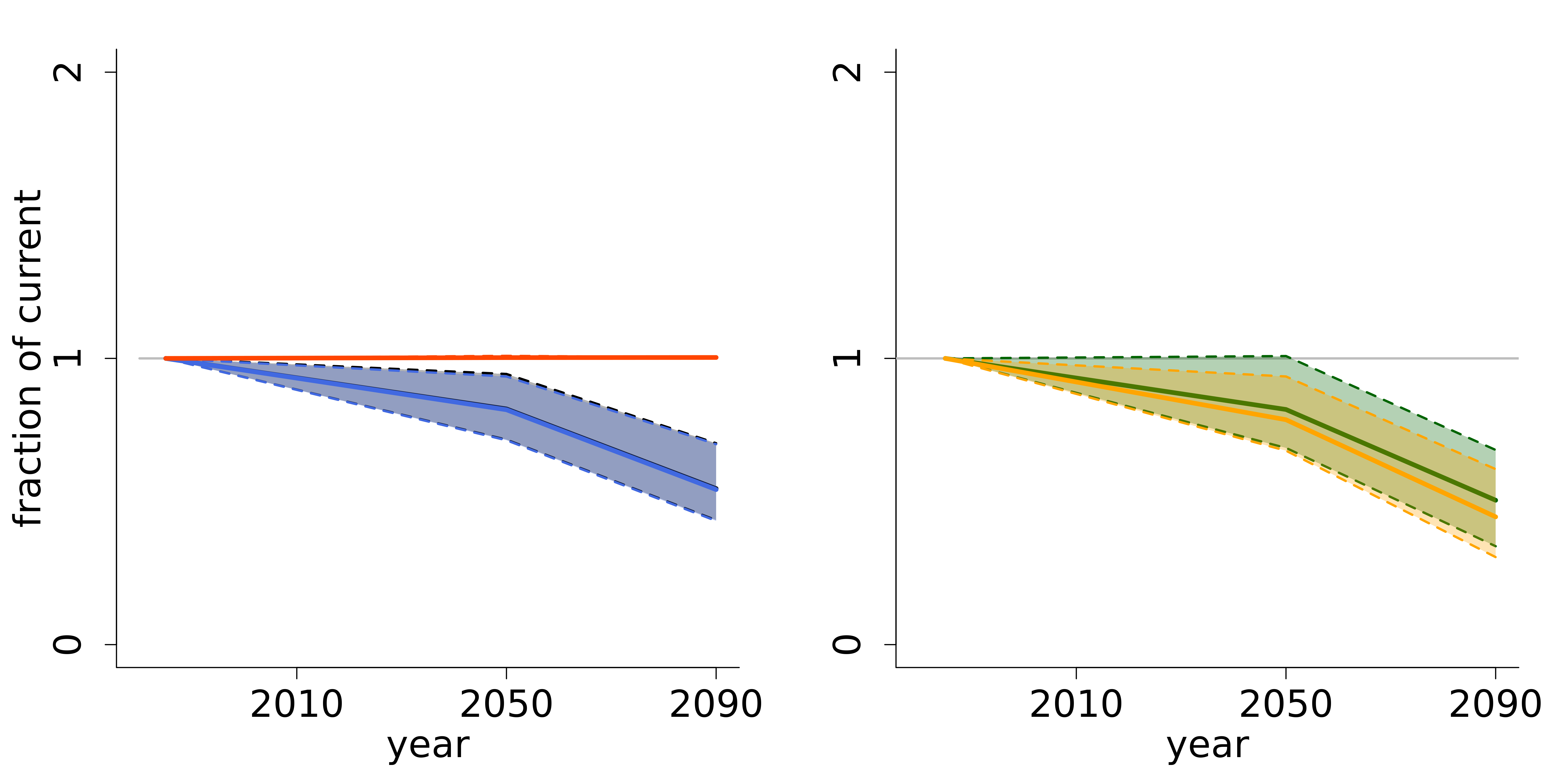

Supplement: S3 Appendix — (ZIP) [file pntd.0014030.s007.zip › Sup. Mat. 6-2 M-Z - Species Trends/Pseudechis_porphyriacus_CCTrends.png]

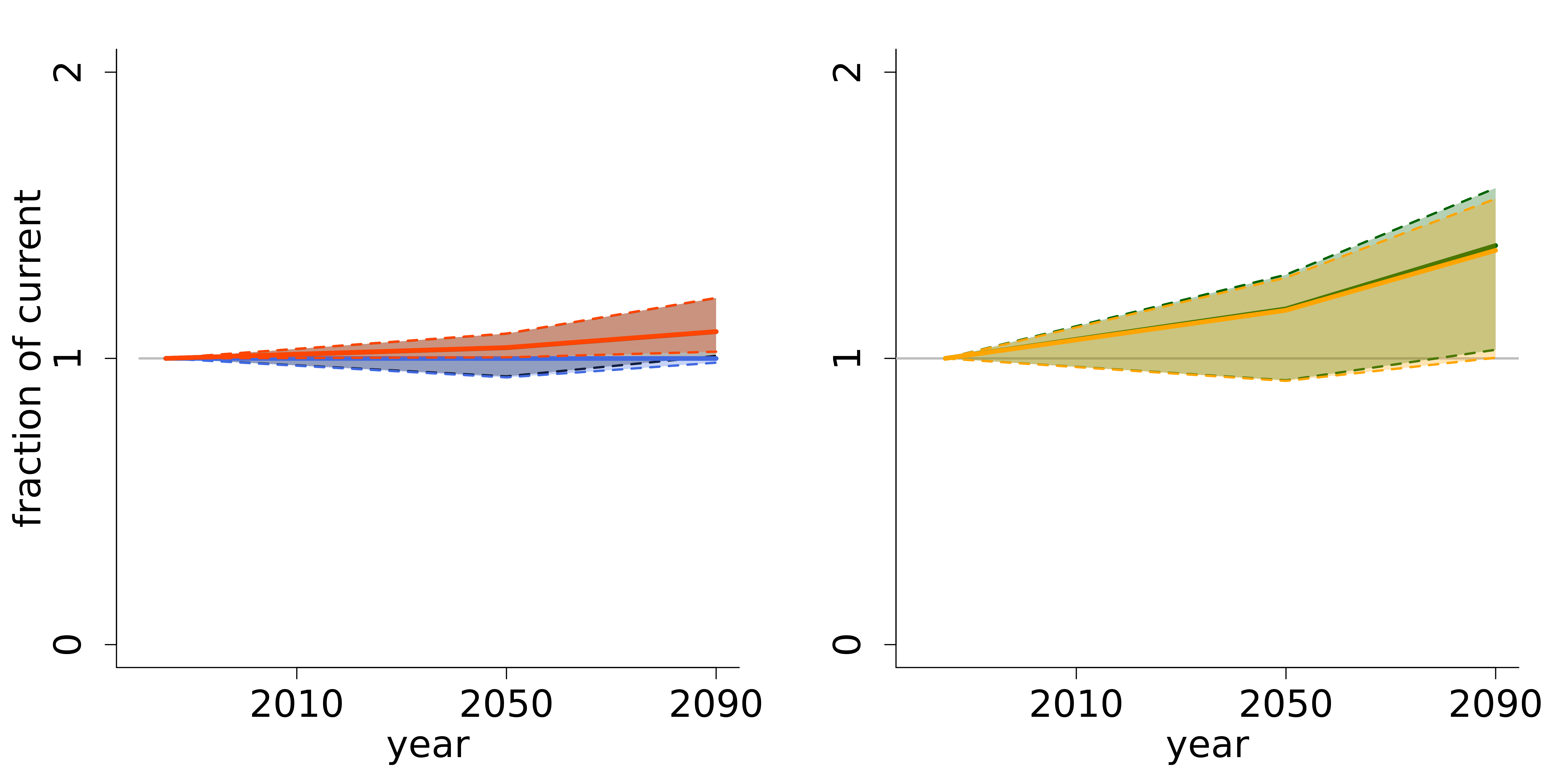

Supplement: S3 Appendix — (ZIP) [file pntd.0014030.s007.zip › Sup. Mat. 6-2 M-Z - Species Trends/Pseudechis_rossignolii_CCTrends.png]

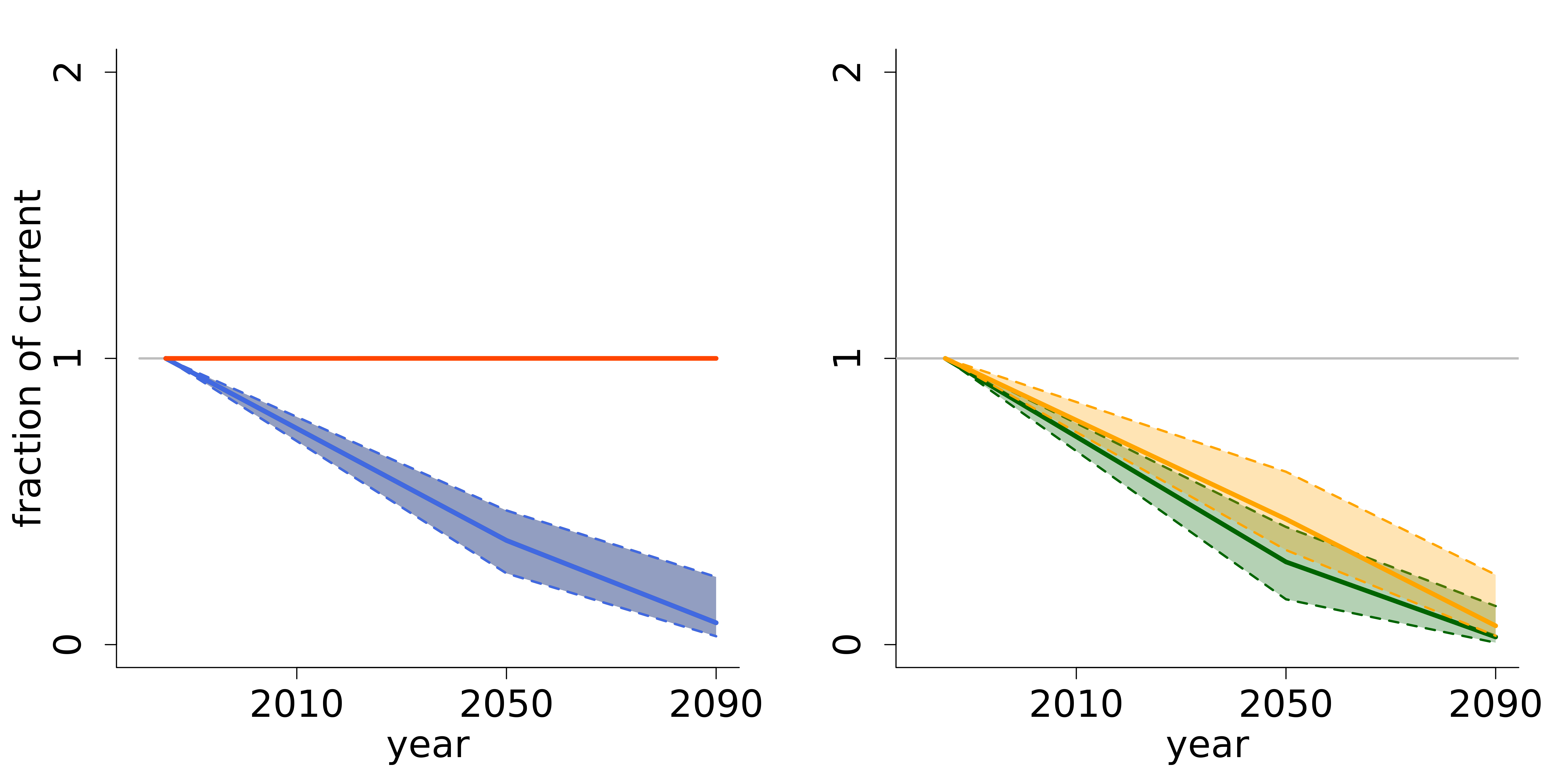

Supplement: S3 Appendix — (ZIP) [file pntd.0014030.s007.zip › Sup. Mat. 6-2 M-Z - Species Trends/Pseudechis_weigeli_CCTrends.png]

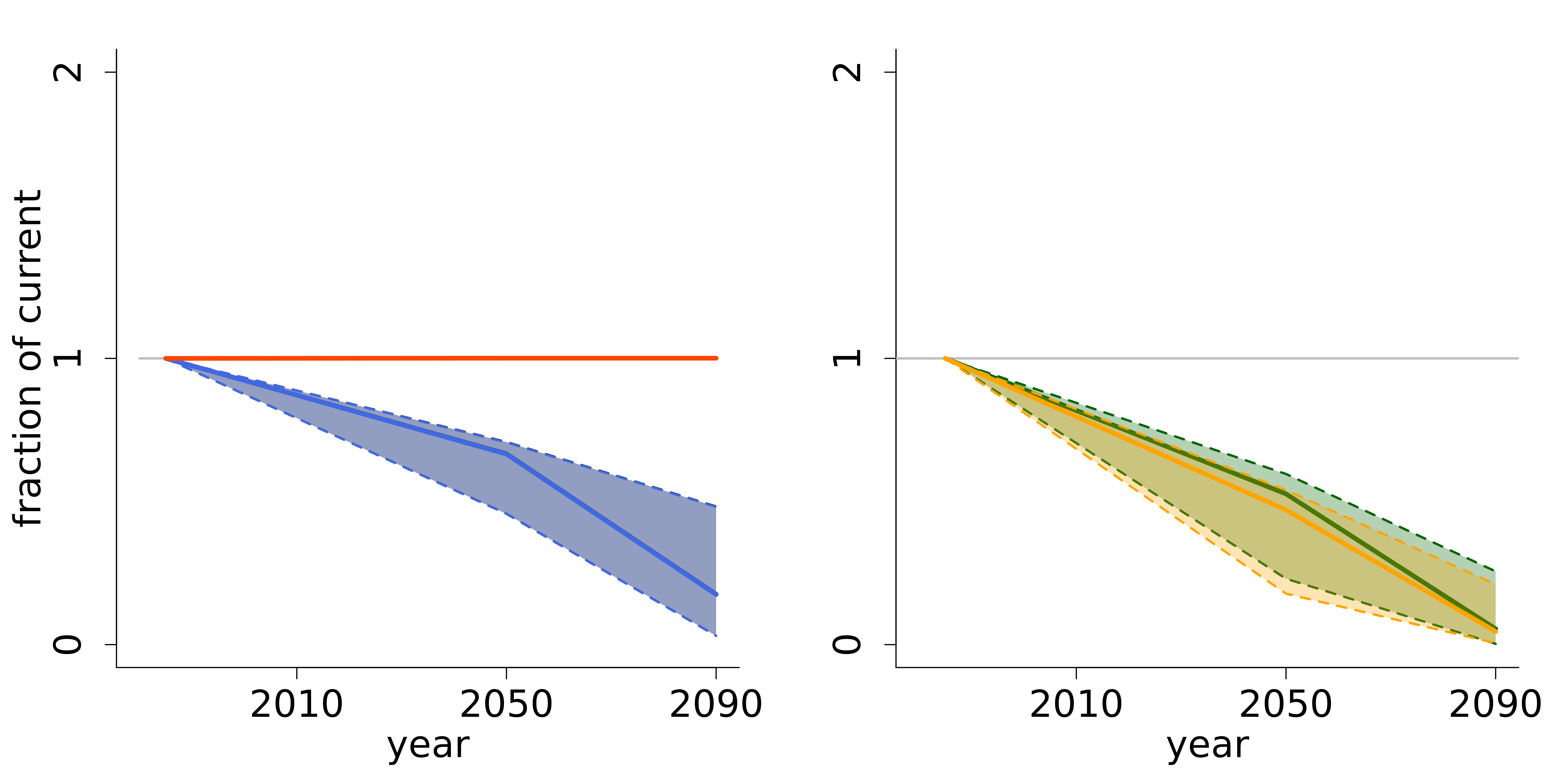

Supplement: S3 Appendix — (ZIP) [file pntd.0014030.s007.zip › Sup. Mat. 6-2 M-Z - Species Trends/Pseudocerastes_fieldi_CCTrends.png]

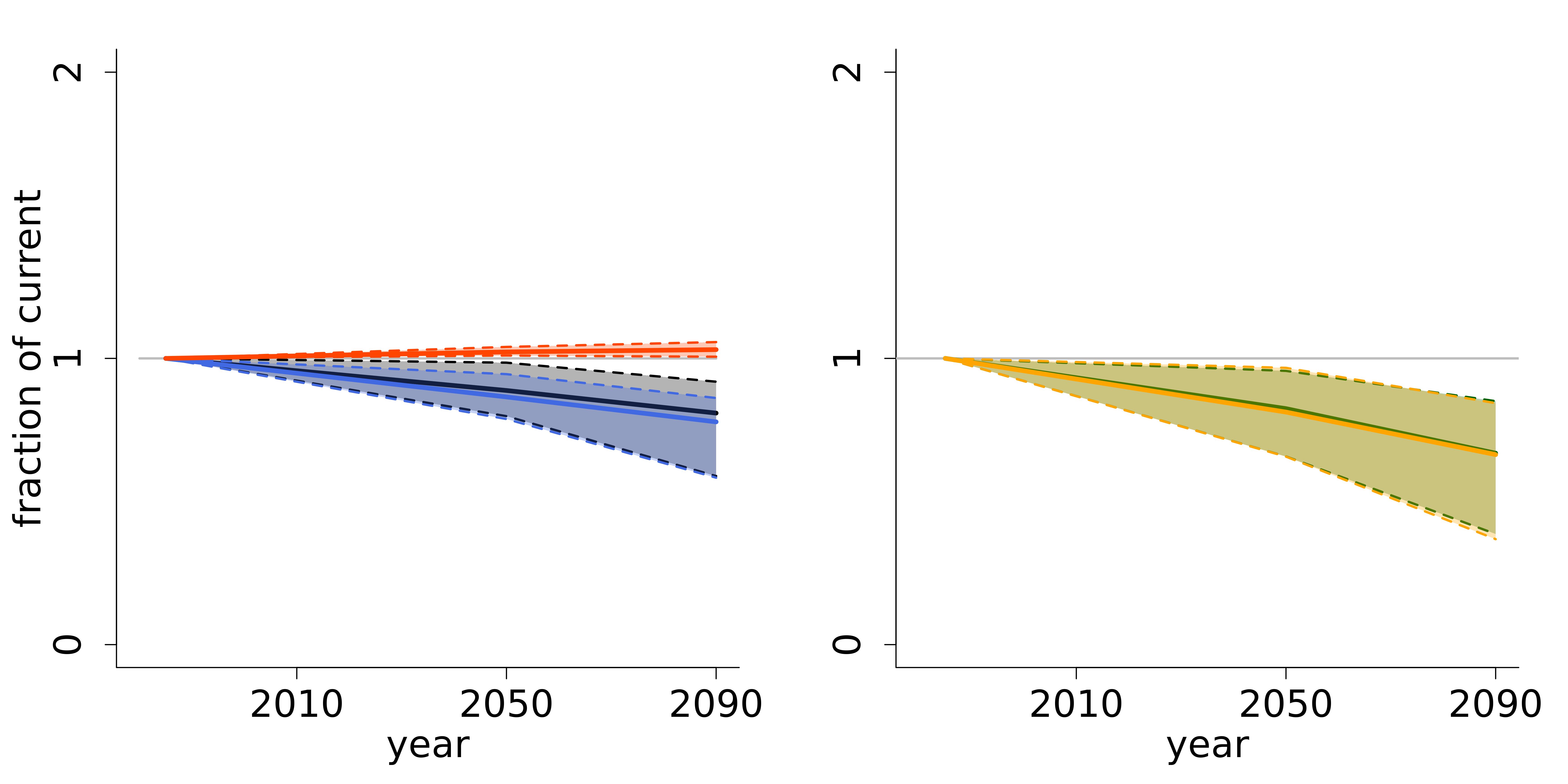

Supplement: S3 Appendix — (ZIP) [file pntd.0014030.s007.zip › Sup. Mat. 6-2 M-Z - Species Trends/Pseudocerastes_persicus_CCTrends.png]

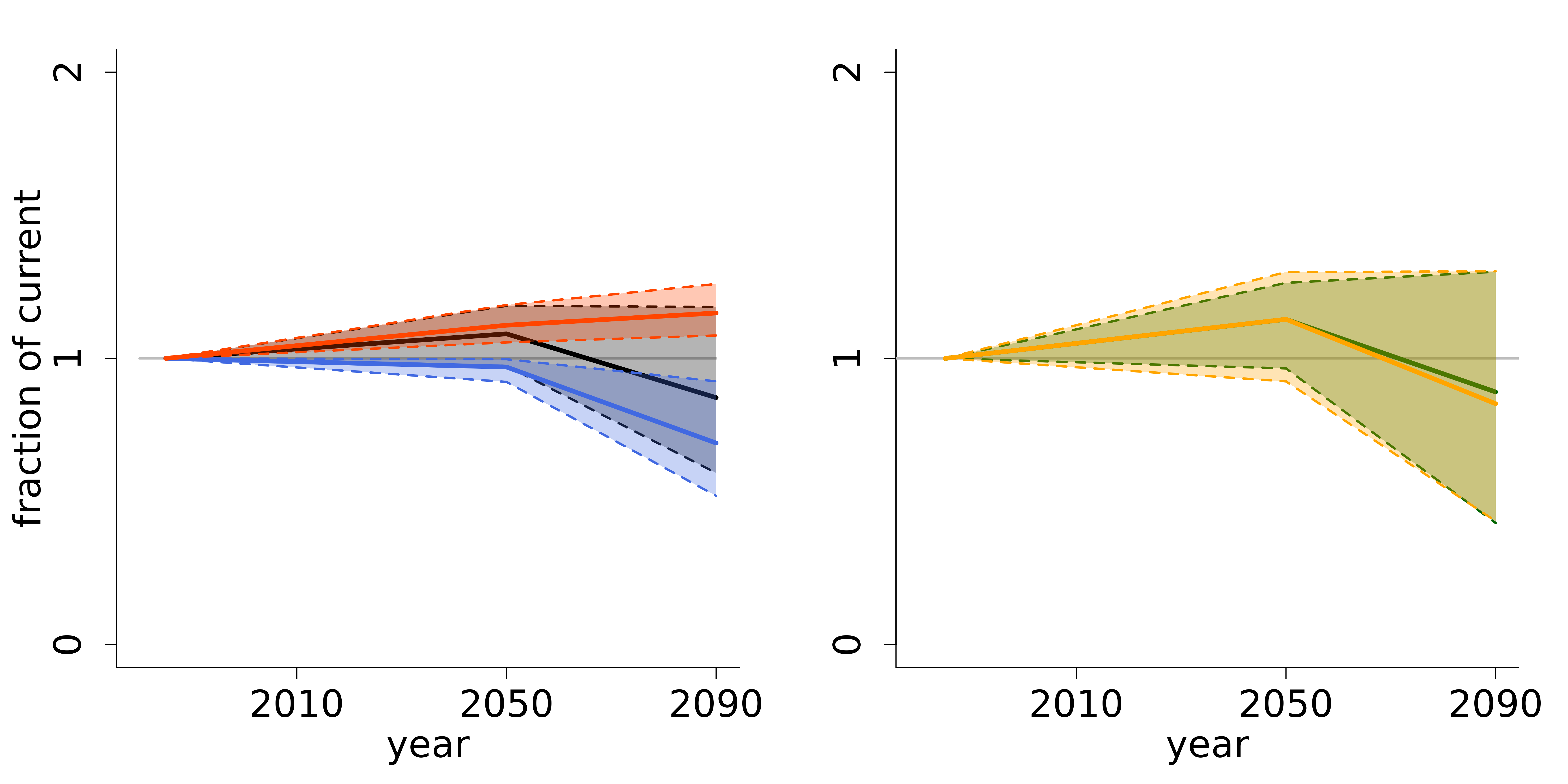

Supplement: S3 Appendix — (ZIP) [file pntd.0014030.s007.zip › Sup. Mat. 6-2 M-Z - Species Trends/Pseudohaje_goldii_CCTrends.png]

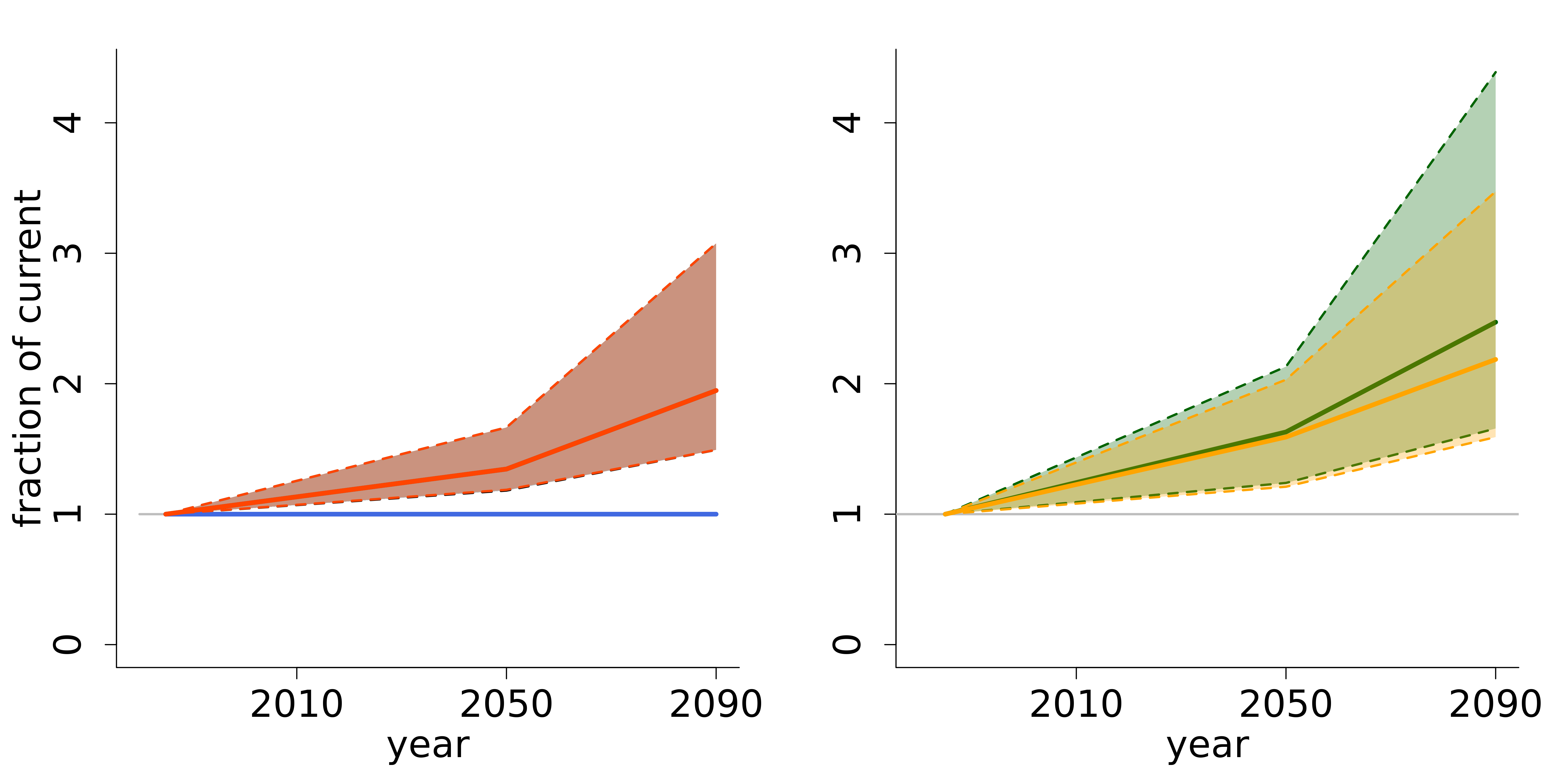

Supplement: S3 Appendix — (ZIP) [file pntd.0014030.s007.zip › Sup. Mat. 6-2 M-Z - Species Trends/Pseudohaje_nigra_CCTrends.png]

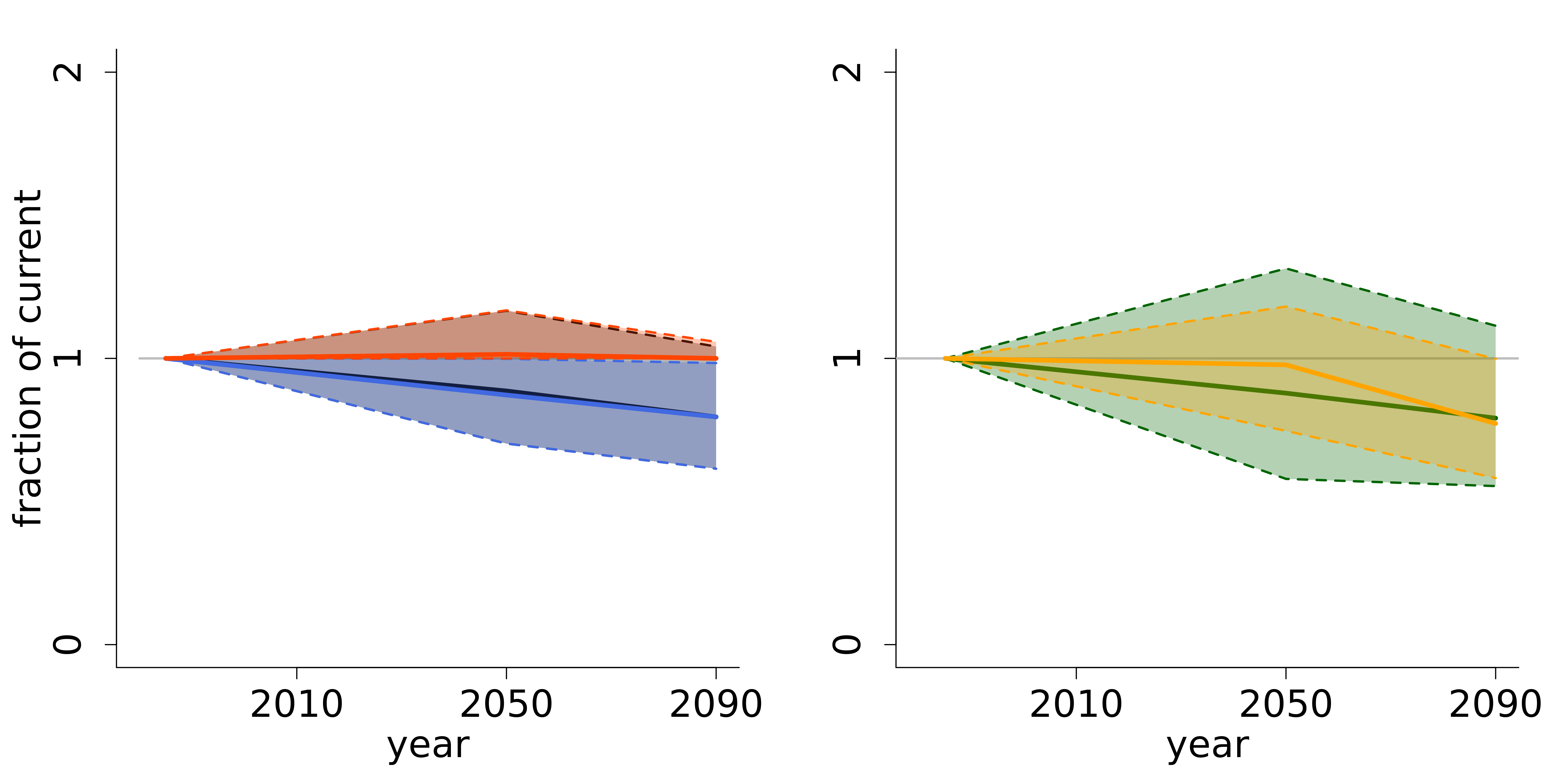

Supplement: S3 Appendix — (ZIP) [file pntd.0014030.s007.zip › Sup. Mat. 6-2 M-Z - Species Trends/Pseudonaja_affinis_CCTrends.png]

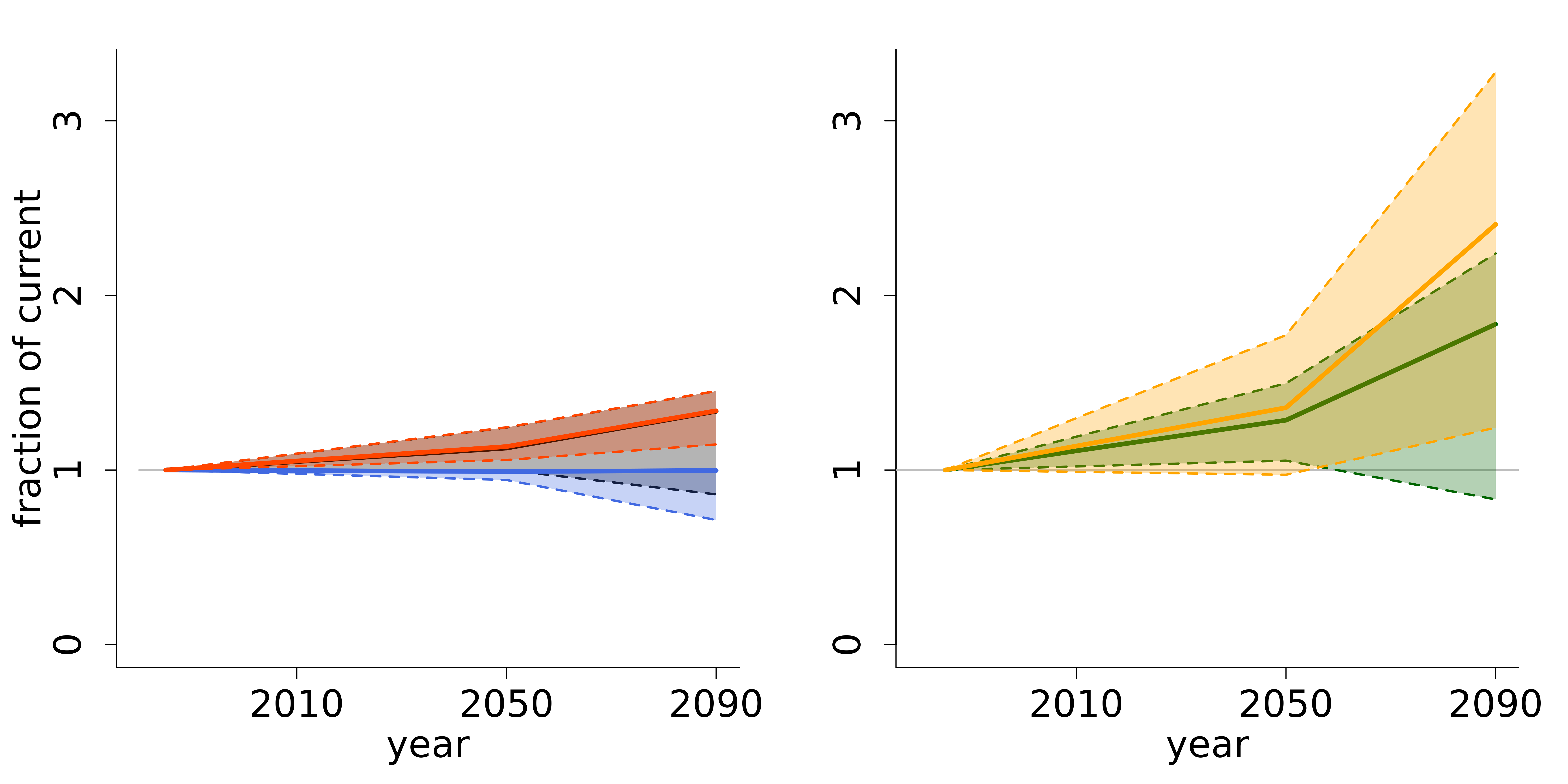

Supplement: S3 Appendix — (ZIP) [file pntd.0014030.s007.zip › Sup. Mat. 6-2 M-Z - Species Trends/Pseudonaja_aspidorhyncha_CCTrends.png]

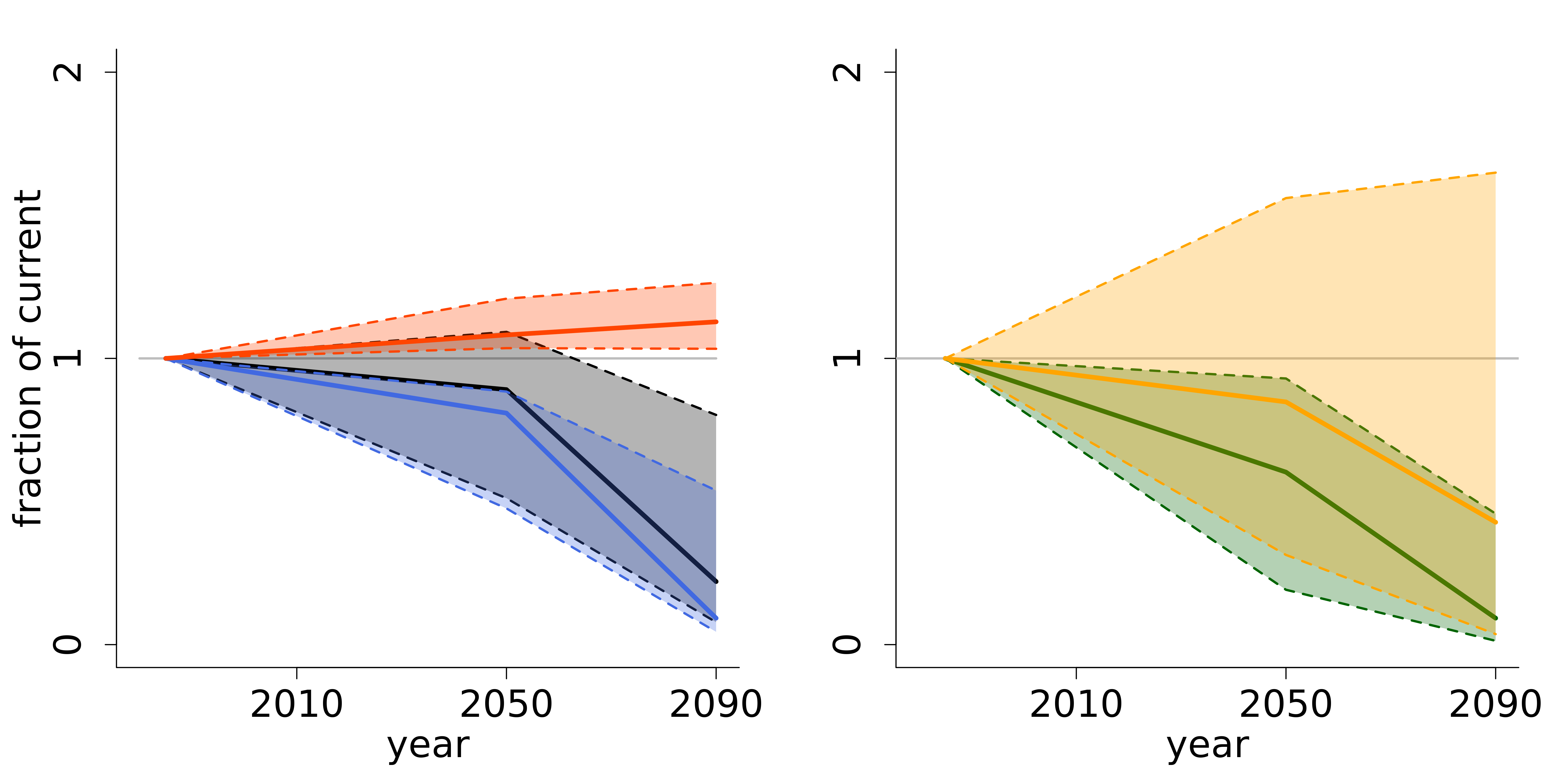

Supplement: S3 Appendix — (ZIP) [file pntd.0014030.s007.zip › Sup. Mat. 6-2 M-Z - Species Trends/Pseudonaja_guttata_CCTrends.png]

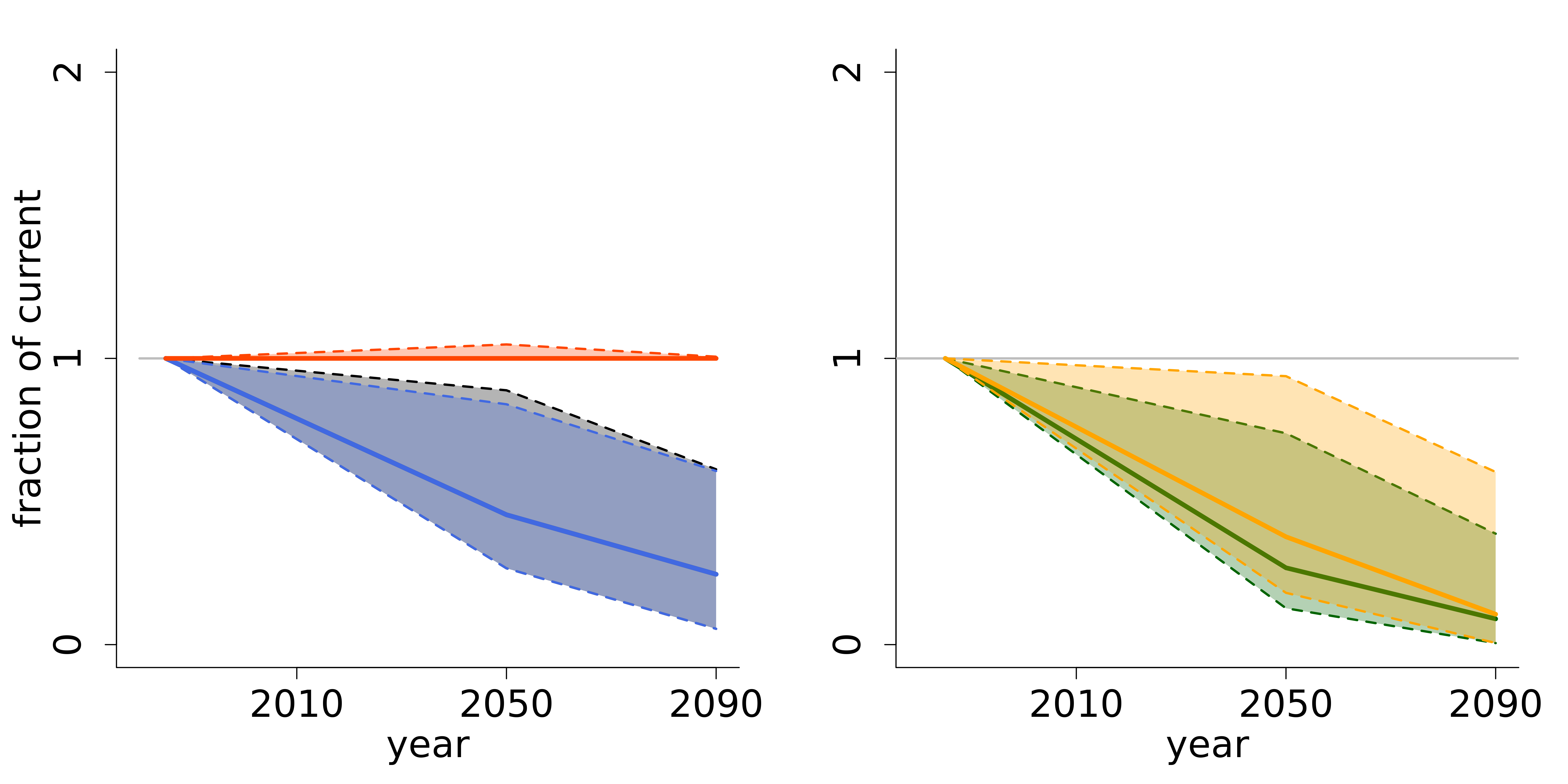

Supplement: S3 Appendix — (ZIP) [file pntd.0014030.s007.zip › Sup. Mat. 6-2 M-Z - Species Trends/Pseudonaja_inframacula_CCTrends.png]

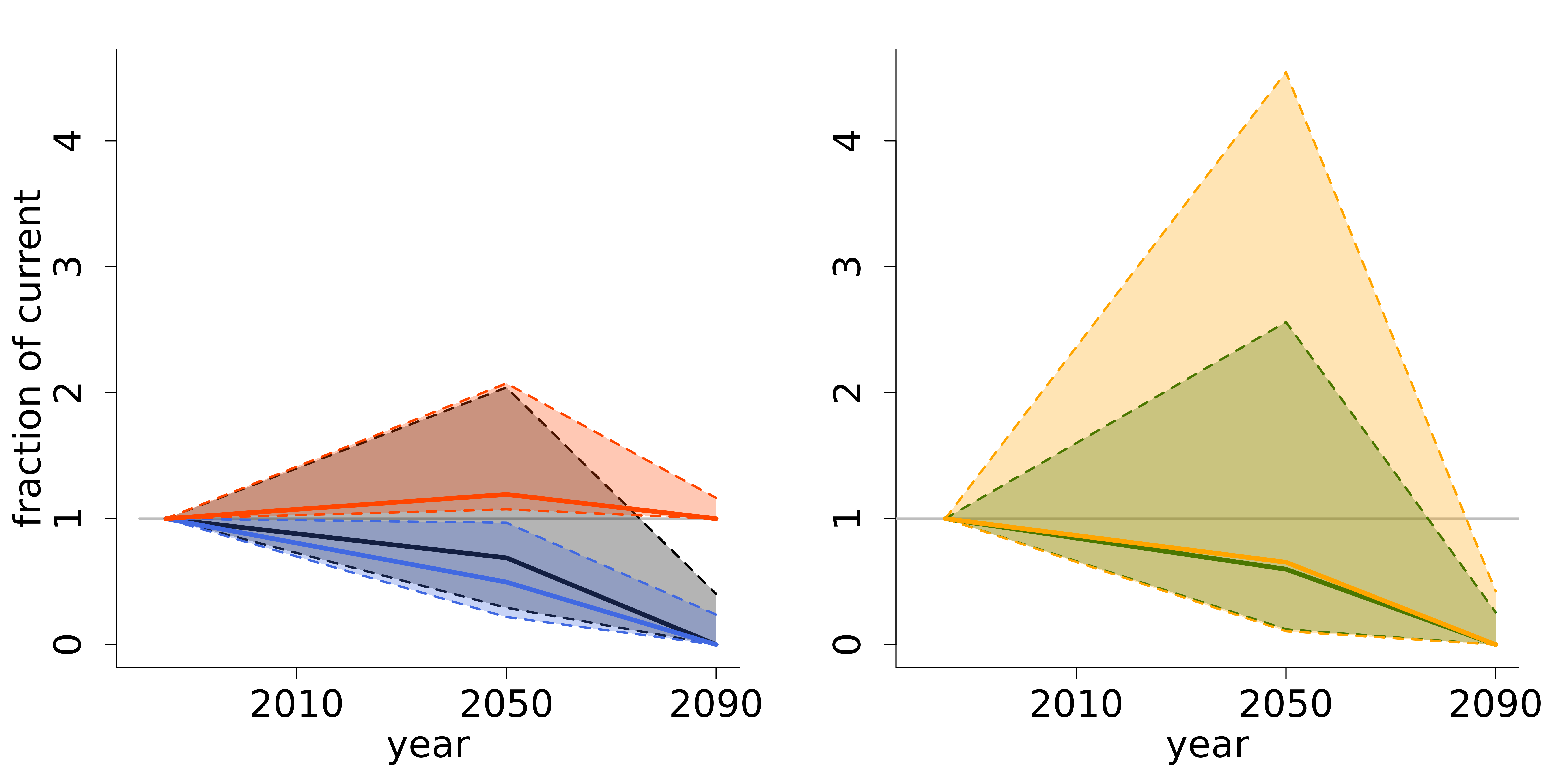

Supplement: S3 Appendix — (ZIP) [file pntd.0014030.s007.zip › Sup. Mat. 6-2 M-Z - Species Trends/Pseudonaja_ingrami_CCTrends.png]

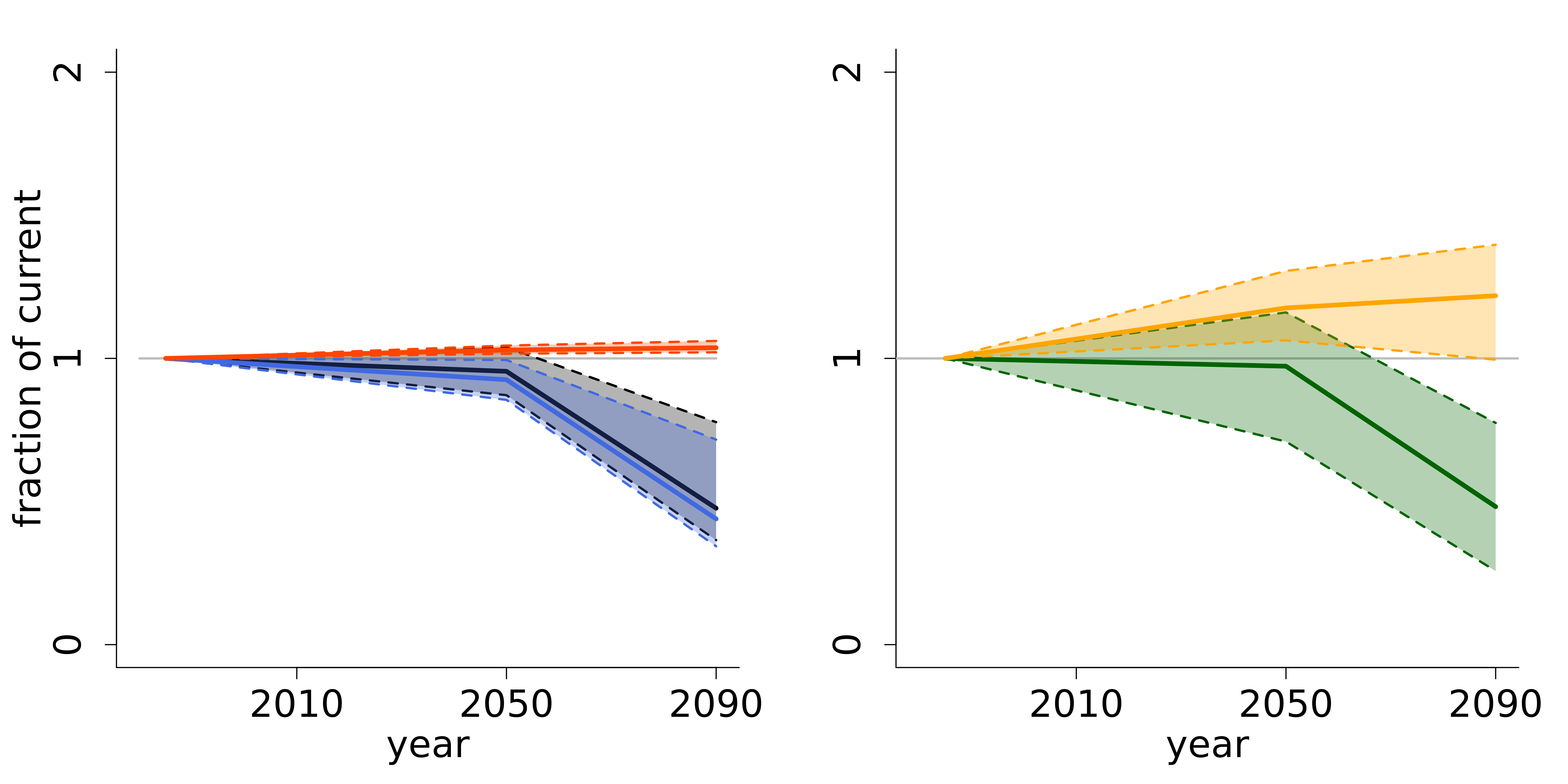

Supplement: S3 Appendix — (ZIP) [file pntd.0014030.s007.zip › Sup. Mat. 6-2 M-Z - Species Trends/Pseudonaja_mengdeni_CCTrends.png]

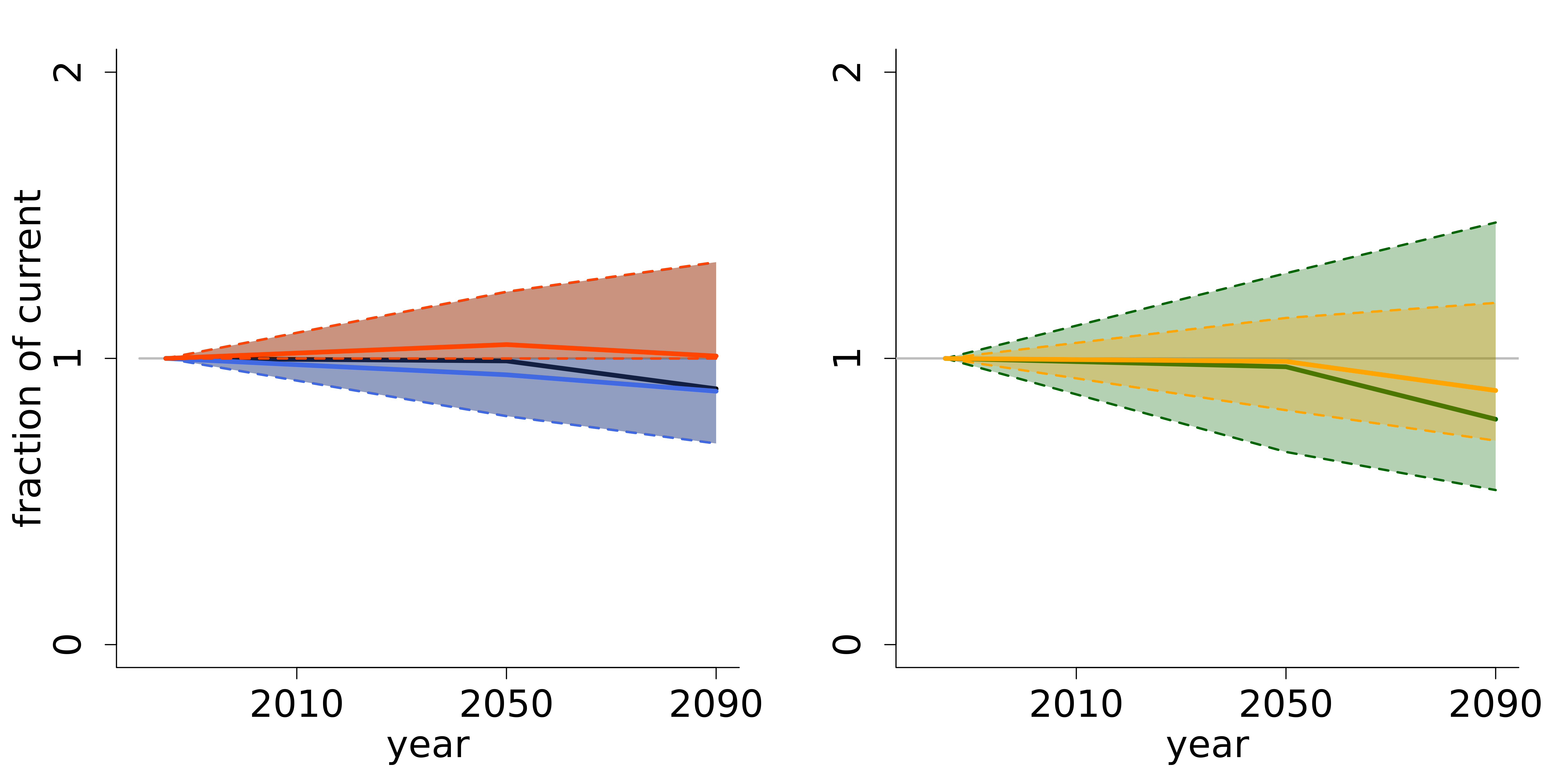

Supplement: S3 Appendix — (ZIP) [file pntd.0014030.s007.zip › Sup. Mat. 6-2 M-Z - Species Trends/Pseudonaja_nuchalis_CCTrends.png]

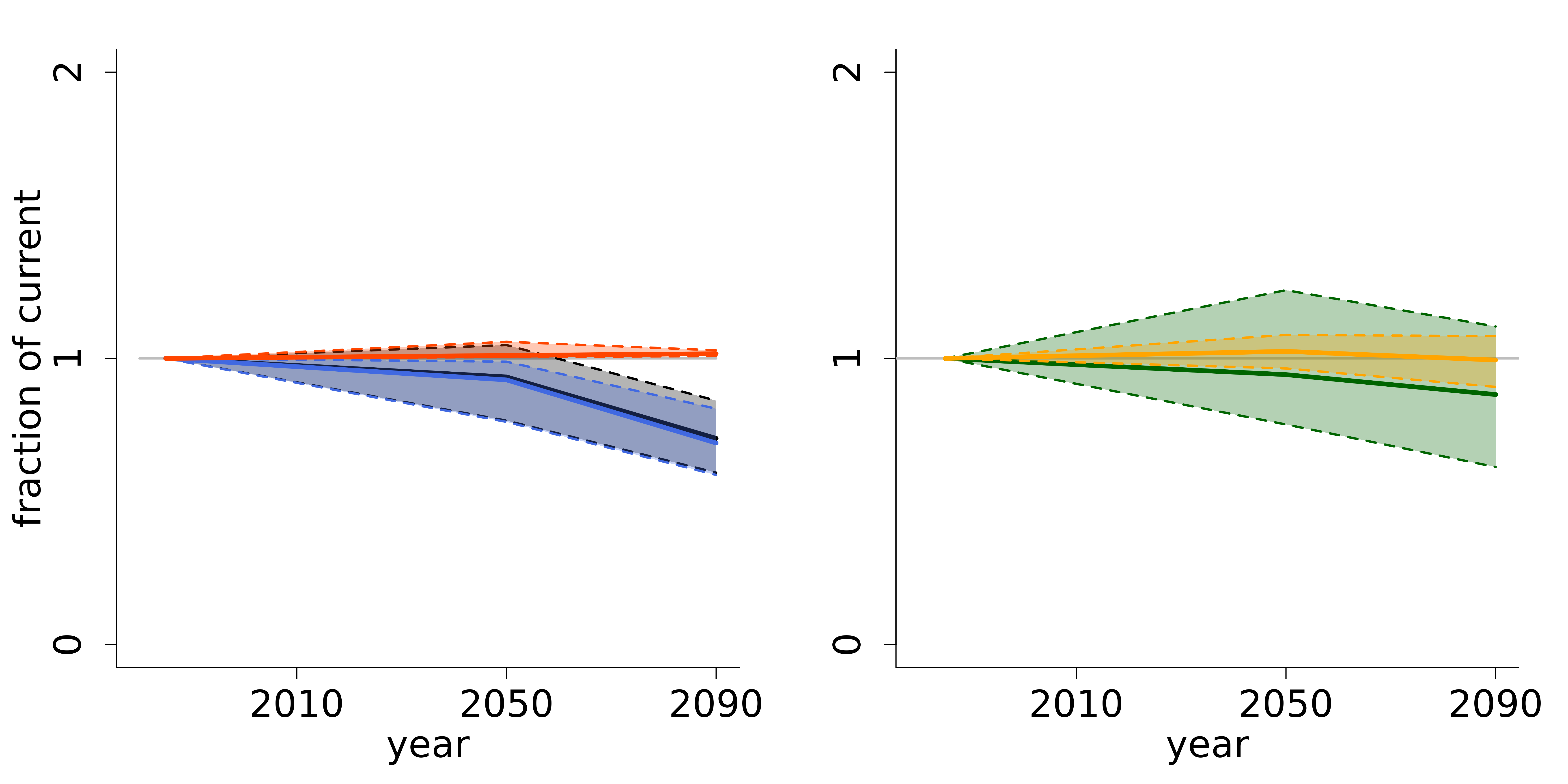

Supplement: S3 Appendix — (ZIP) [file pntd.0014030.s007.zip › Sup. Mat. 6-2 M-Z - Species Trends/Pseudonaja_textilis_CCTrends.png]

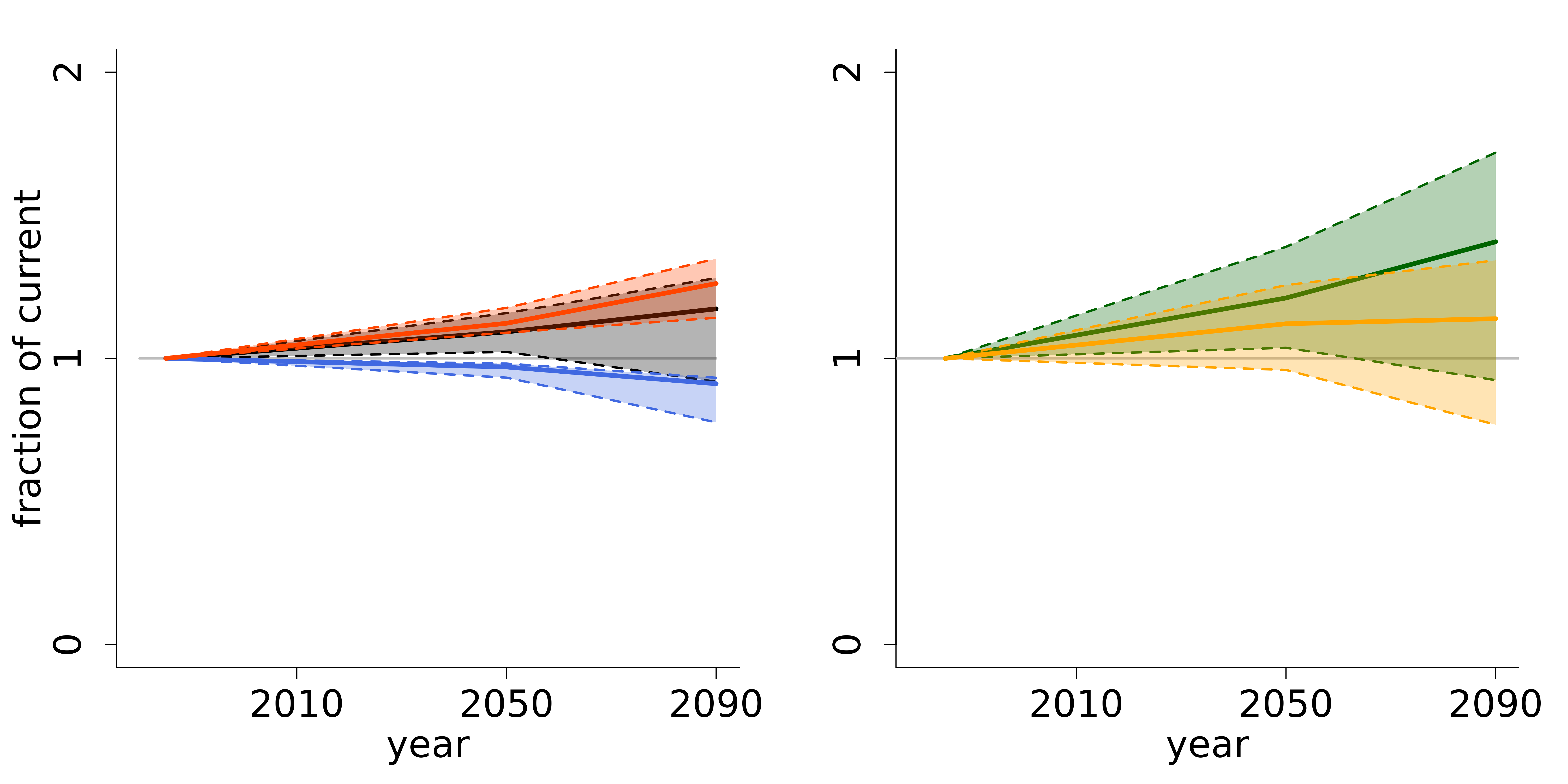

Supplement: S3 Appendix — (ZIP) [file pntd.0014030.s007.zip › Sup. Mat. 6-2 M-Z - Species Trends/Rhabdophis_tigrinus_CCTrends.png]

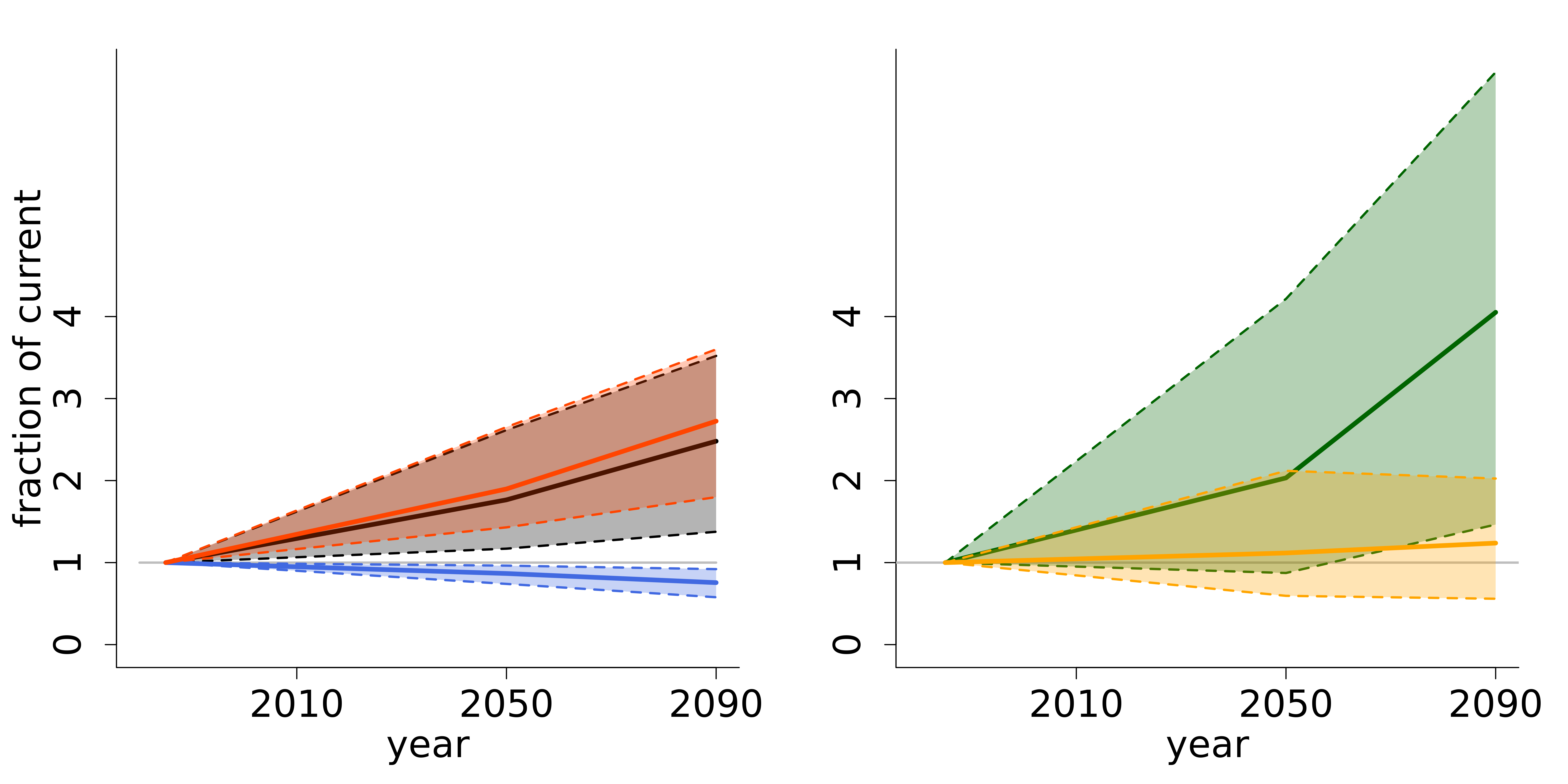

Supplement: S3 Appendix — (ZIP) [file pntd.0014030.s007.zip › Sup. Mat. 6-2 M-Z - Species Trends/Sistrurus_catenatus_CCTrends.png]

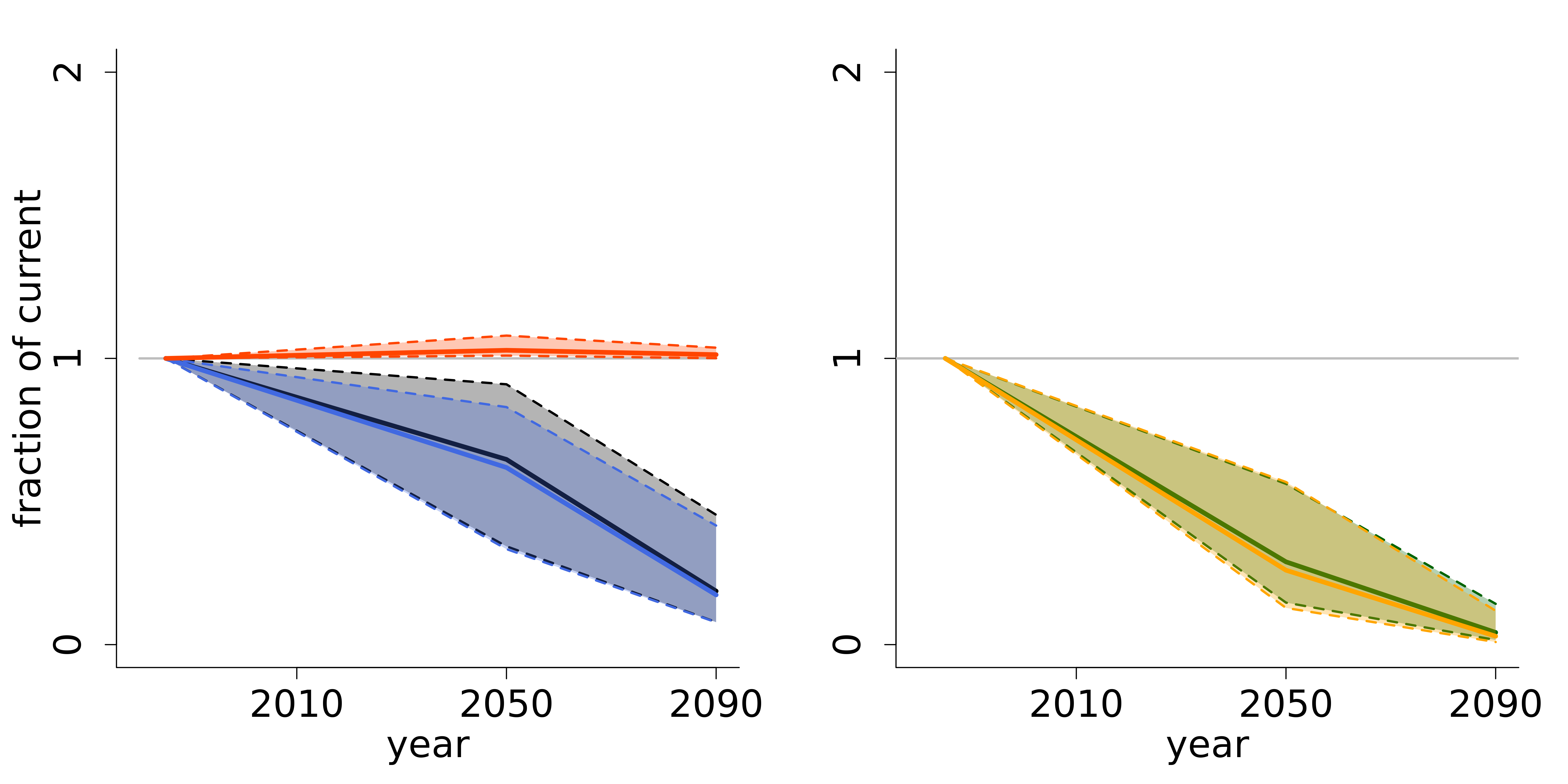

Supplement: S3 Appendix — (ZIP) [file pntd.0014030.s007.zip › Sup. Mat. 6-2 M-Z - Species Trends/Sistrurus_miliarius_CCTrends.png]

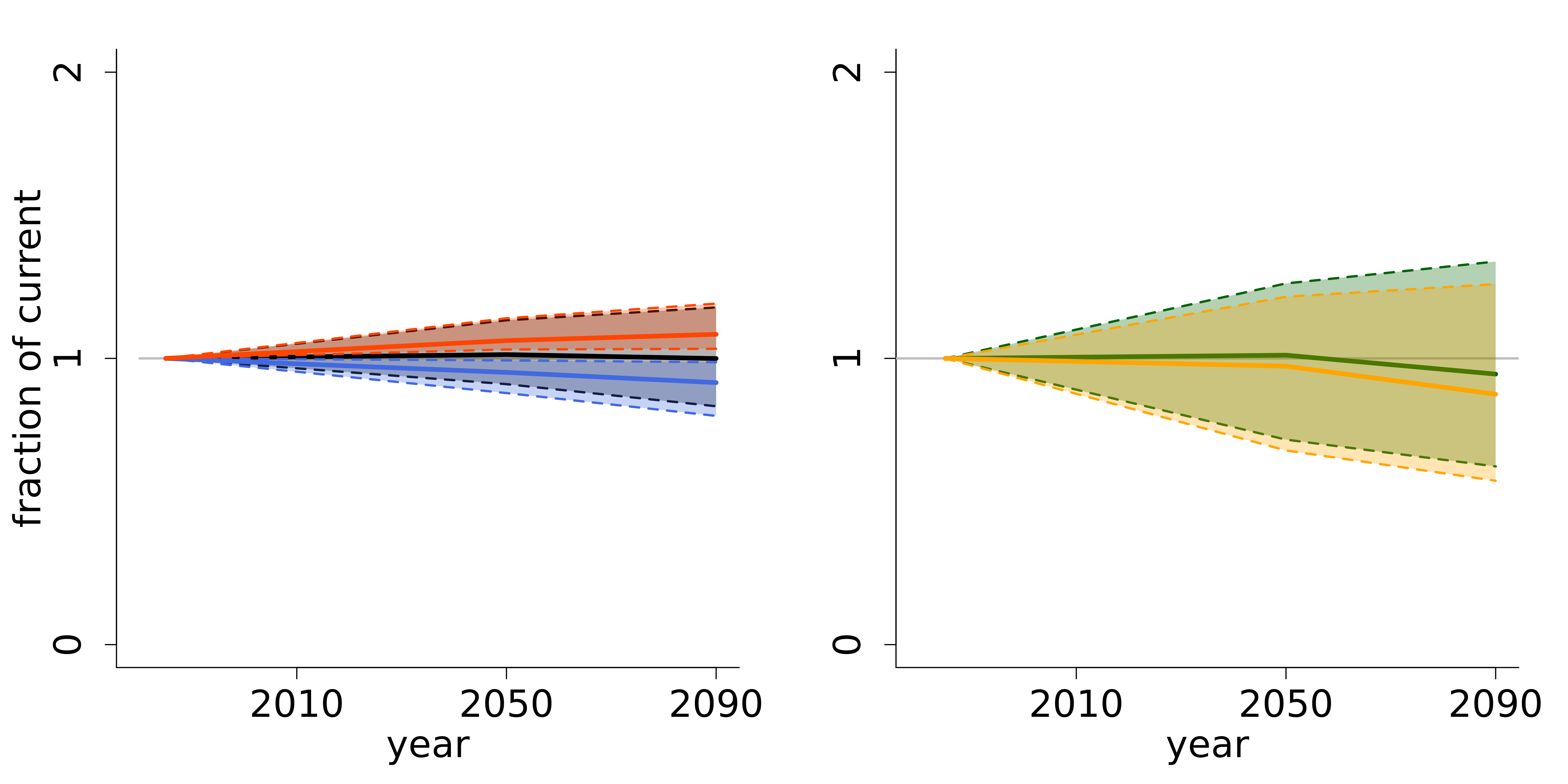

Supplement: S3 Appendix — (ZIP) [file pntd.0014030.s007.zip › Sup. Mat. 6-2 M-Z - Species Trends/Sistrurus_tergeminus_CCTrends.png]

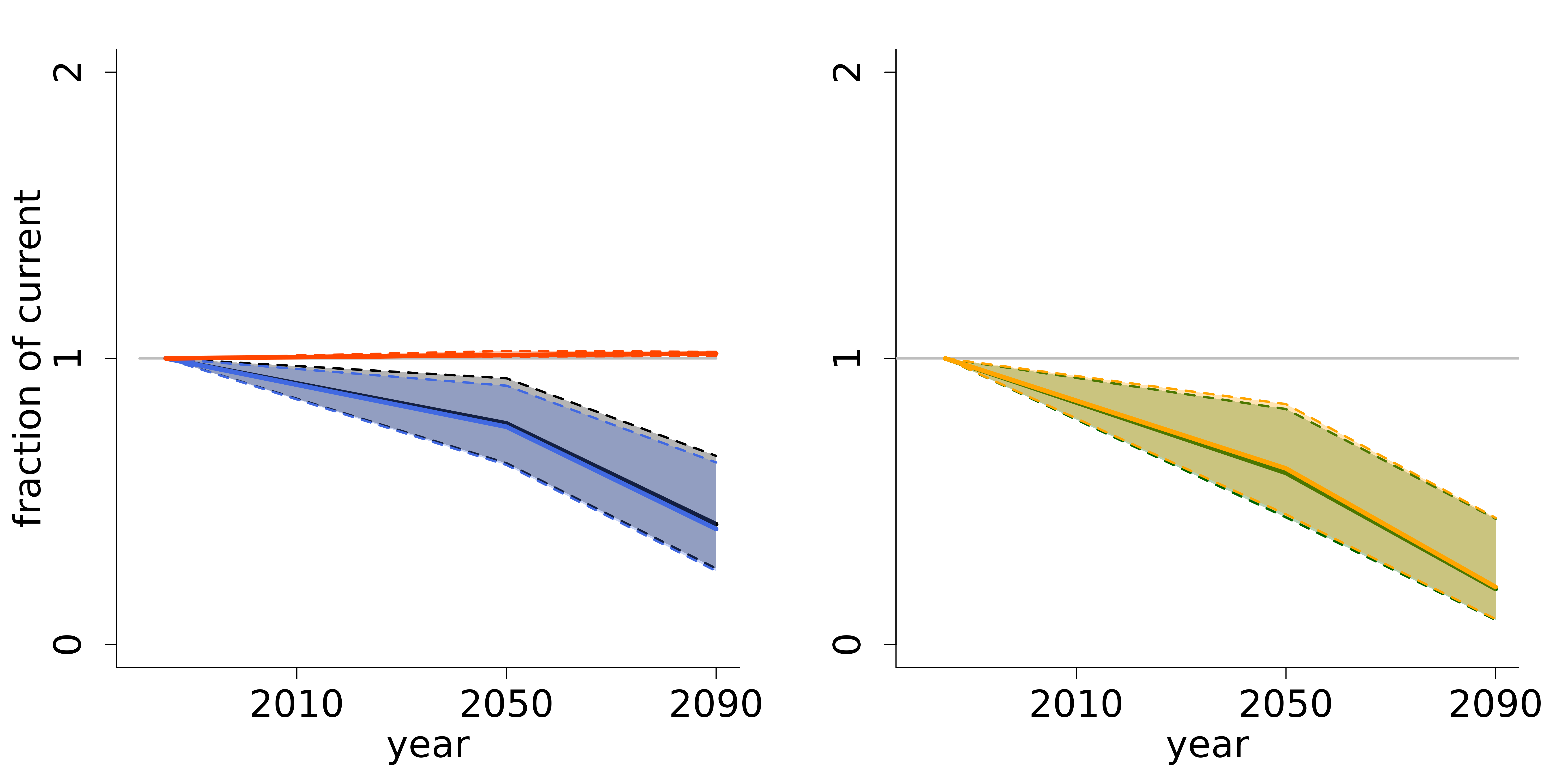

Supplement: S3 Appendix — (ZIP) [file pntd.0014030.s007.zip › Sup. Mat. 6-2 M-Z - Species Trends/Thelotornis_capensis_CCTrends.png]

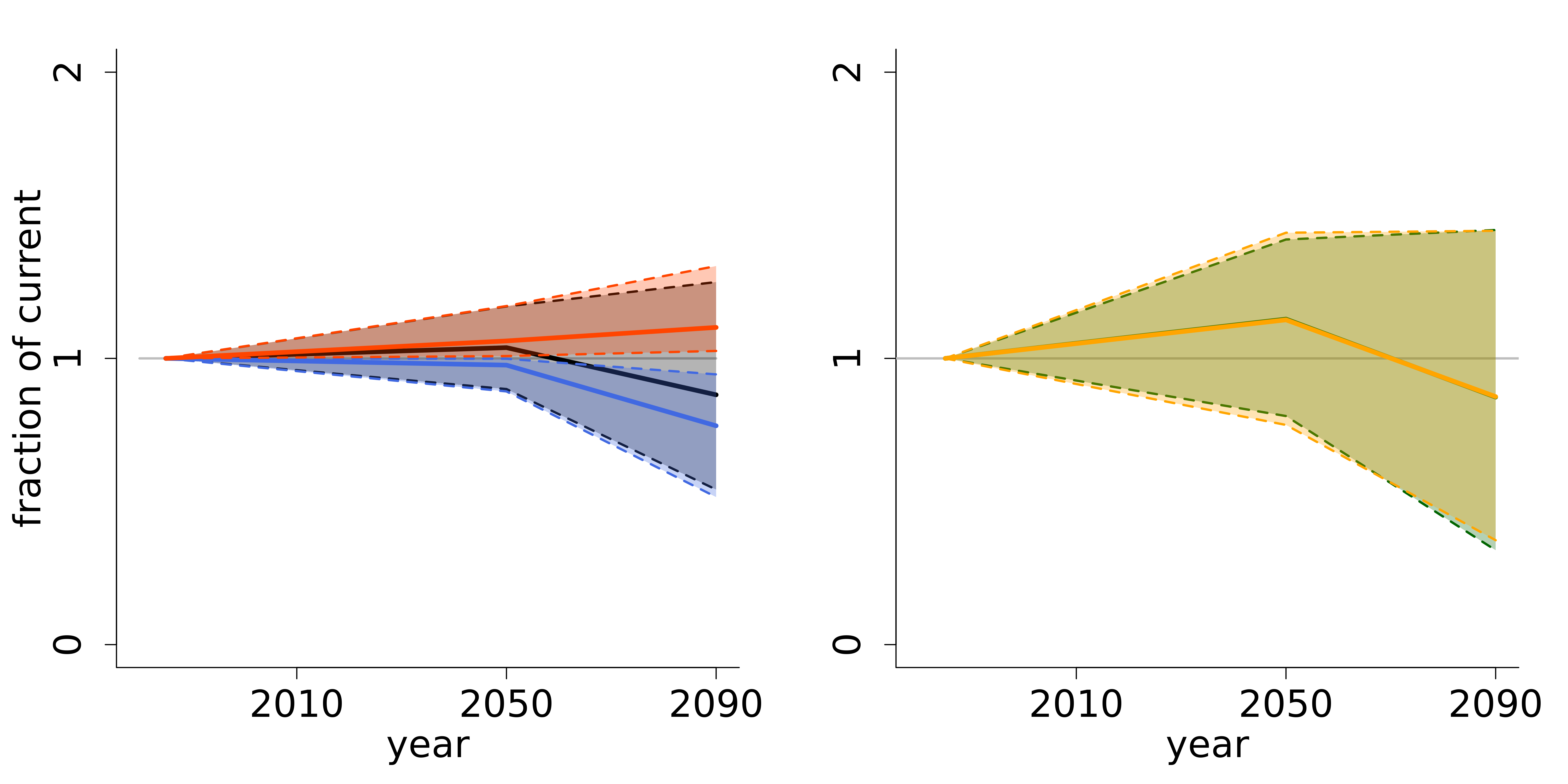

Supplement: S3 Appendix — (ZIP) [file pntd.0014030.s007.zip › Sup. Mat. 6-2 M-Z - Species Trends/Thelotornis_kirtlandii_CCTrends.png]

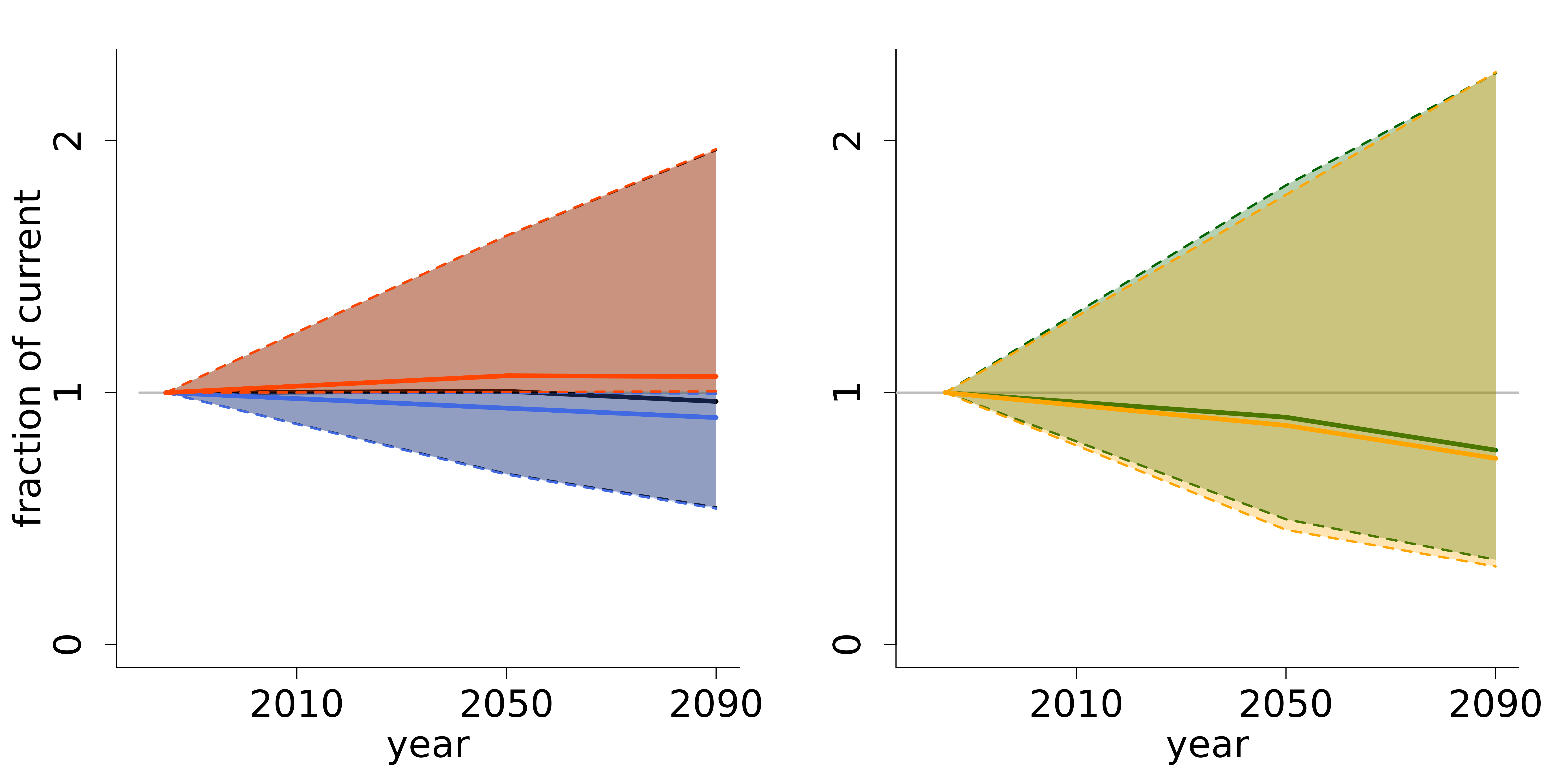

Supplement: S3 Appendix — (ZIP) [file pntd.0014030.s007.zip › Sup. Mat. 6-2 M-Z - Species Trends/Thelotornis_mossambicanus_CCTrends.png]

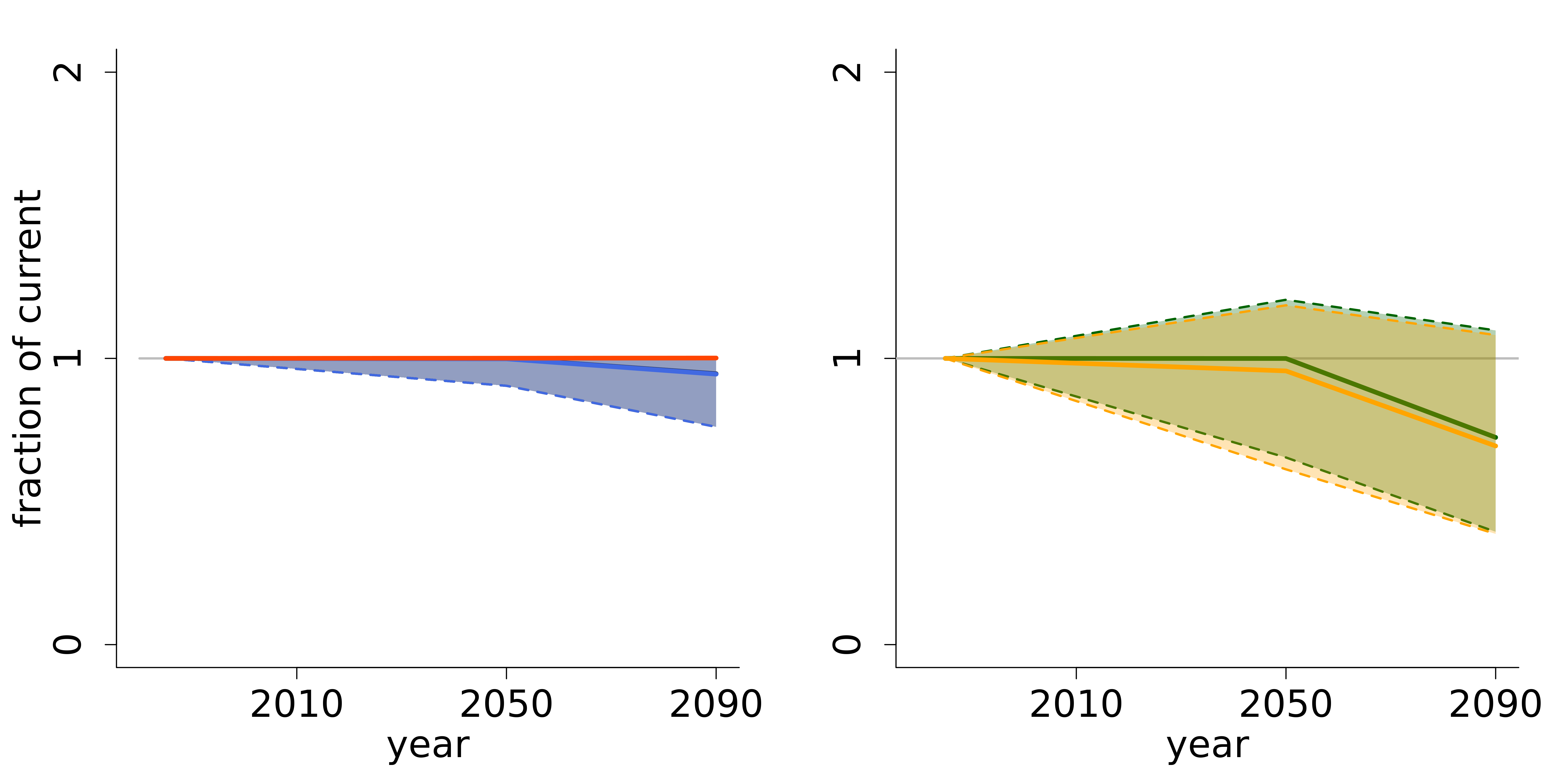

Supplement: S3 Appendix — (ZIP) [file pntd.0014030.s007.zip › Sup. Mat. 6-2 M-Z - Species Trends/Thelotornis_usambaricus_CCTrends.png]

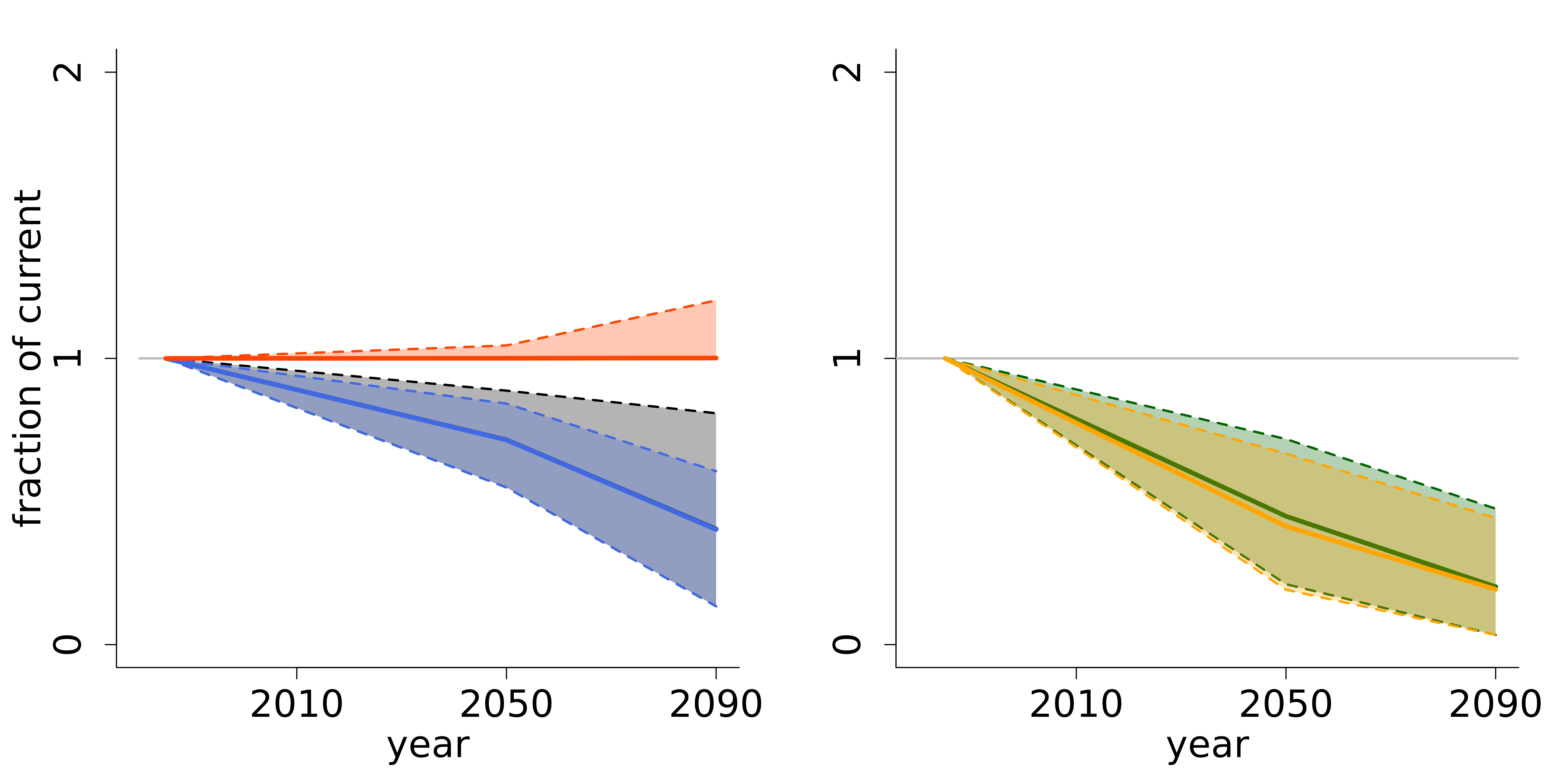

Supplement: S3 Appendix — (ZIP) [file pntd.0014030.s007.zip › Sup. Mat. 6-2 M-Z - Species Trends/Trimeresurus_aff._albolabris_CCTrends.png]

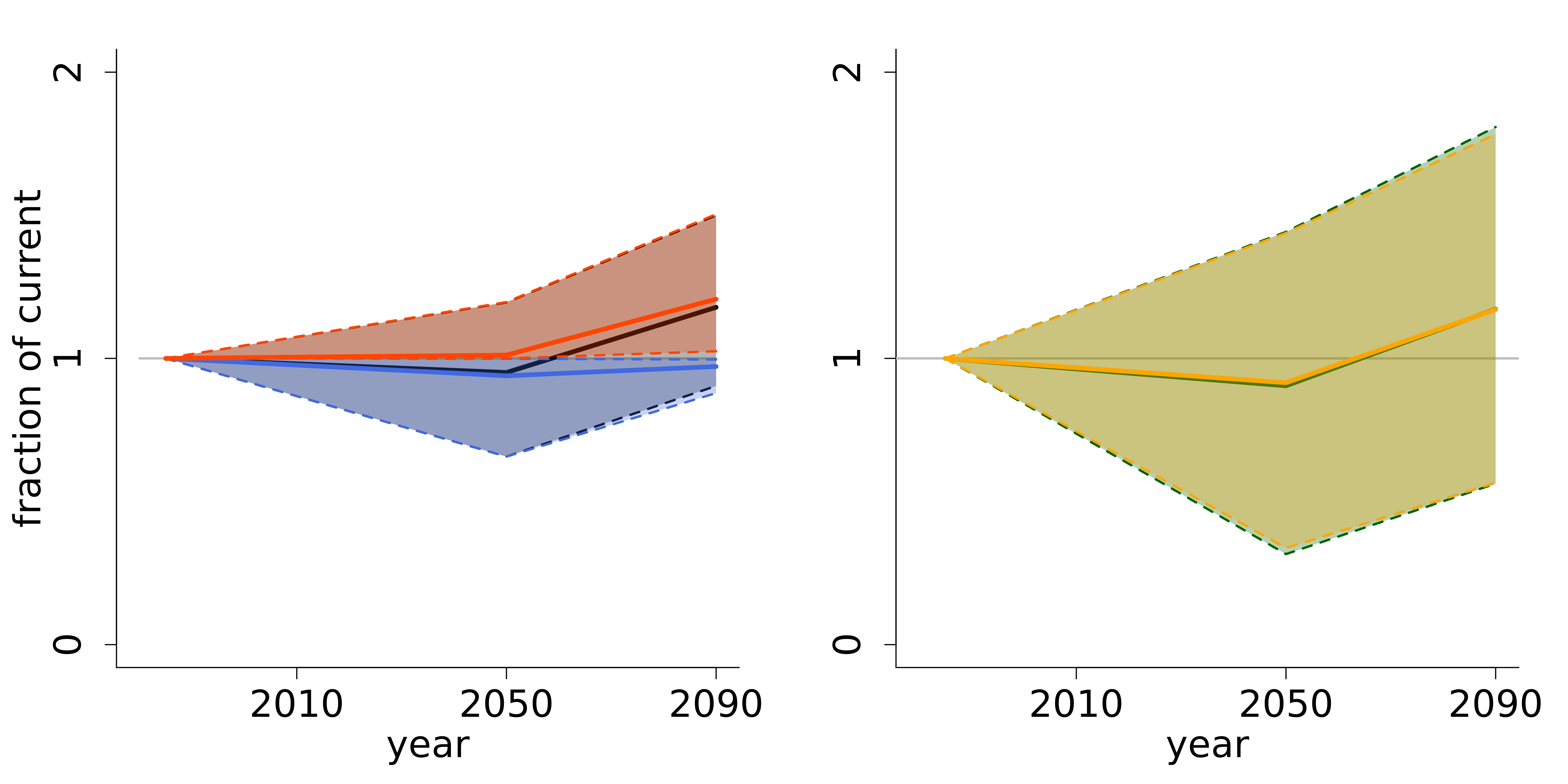

Supplement: S3 Appendix — (ZIP) [file pntd.0014030.s007.zip › Sup. Mat. 6-2 M-Z - Species Trends/Trimeresurus_albolabris_CCTrends.png]

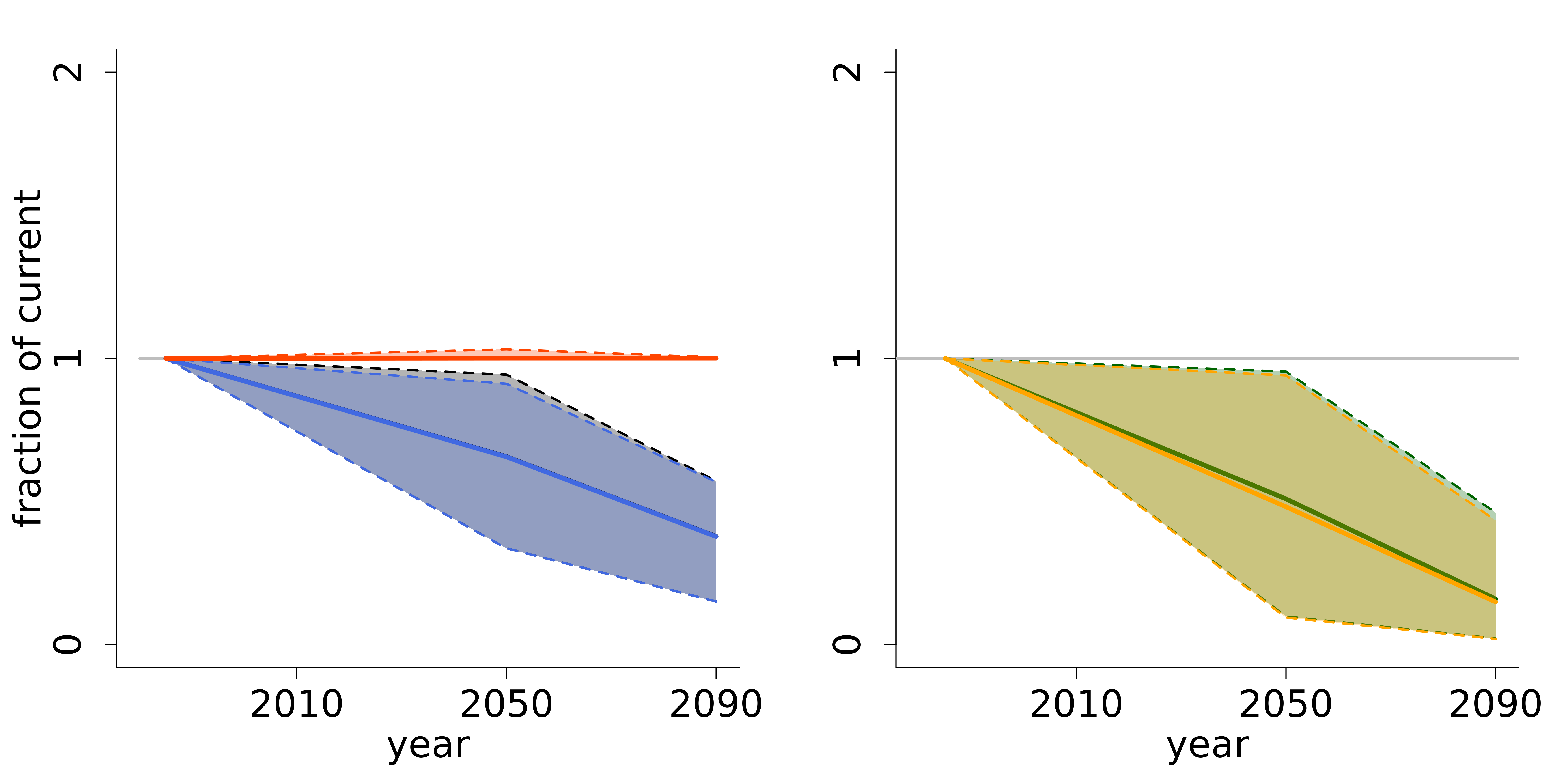

Supplement: S3 Appendix — (ZIP) [file pntd.0014030.s007.zip › Sup. Mat. 6-2 M-Z - Species Trends/Trimeresurus_anamallensis_CCTrends.png]

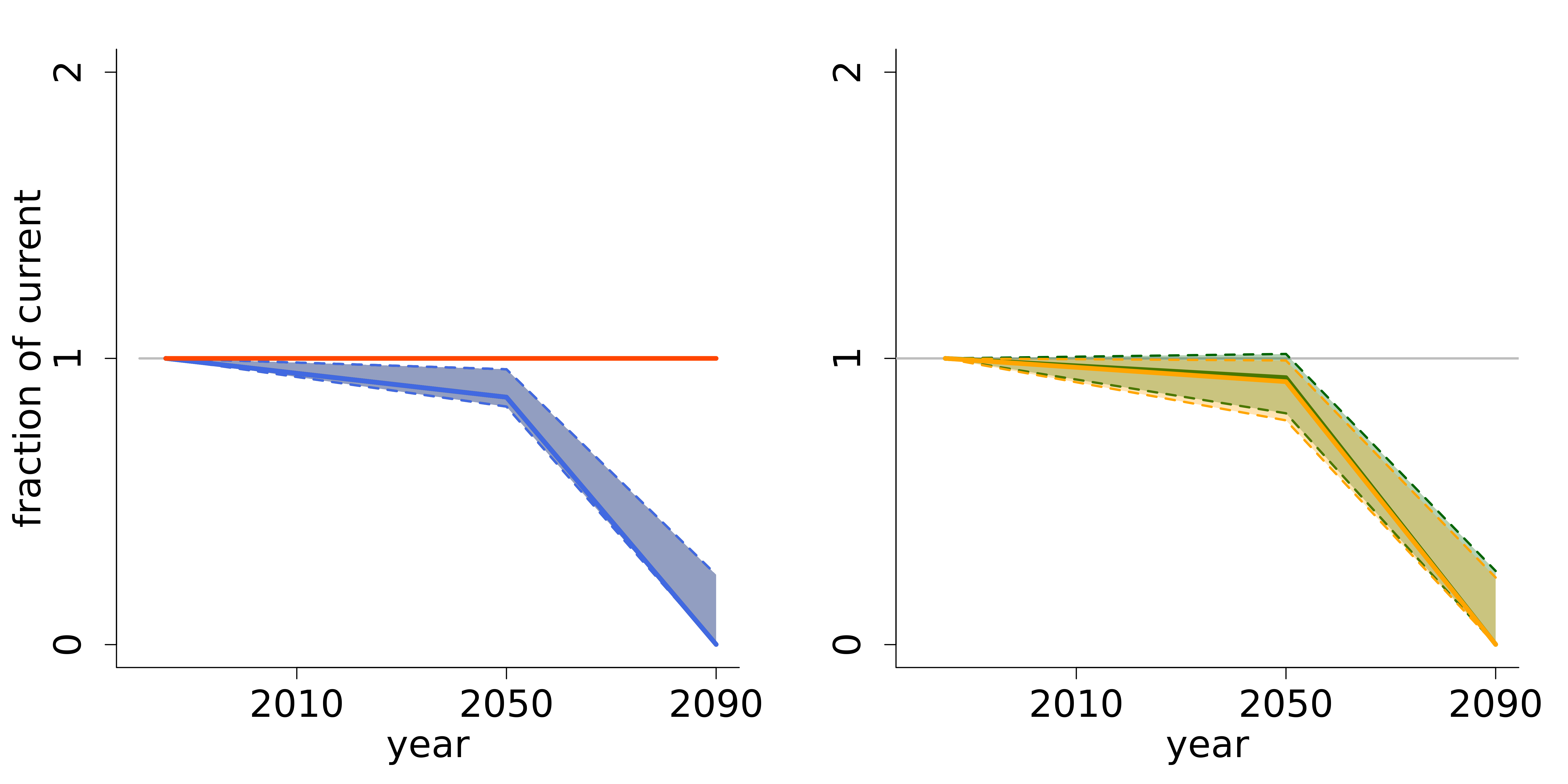

Supplement: S3 Appendix — (ZIP) [file pntd.0014030.s007.zip › Sup. Mat. 6-2 M-Z - Species Trends/Trimeresurus_andersonii_CCTrends.png]

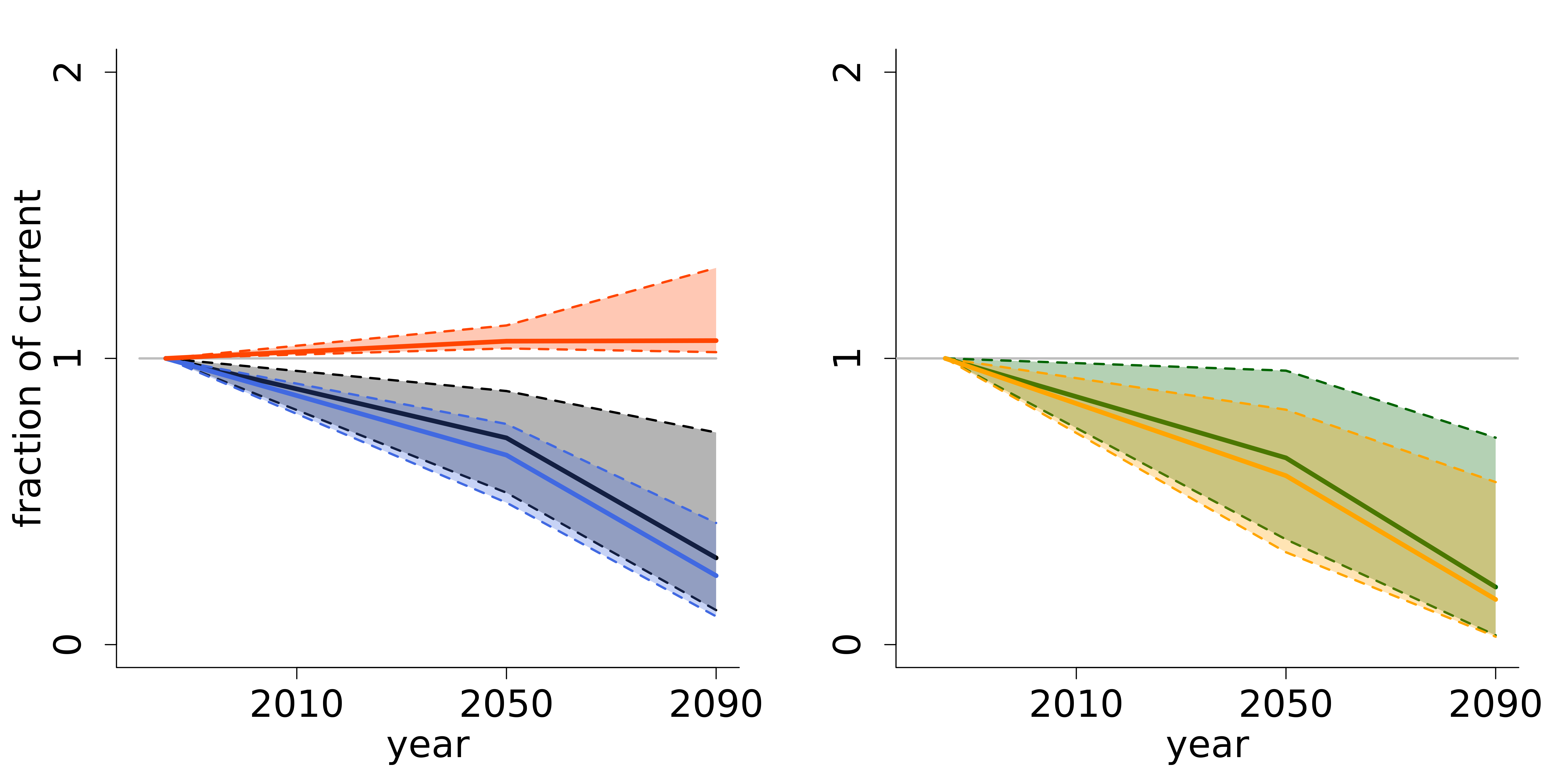

Supplement: S3 Appendix — (ZIP) [file pntd.0014030.s007.zip › Sup. Mat. 6-2 M-Z - Species Trends/Trimeresurus_arunachalensis_CCTrends.png]

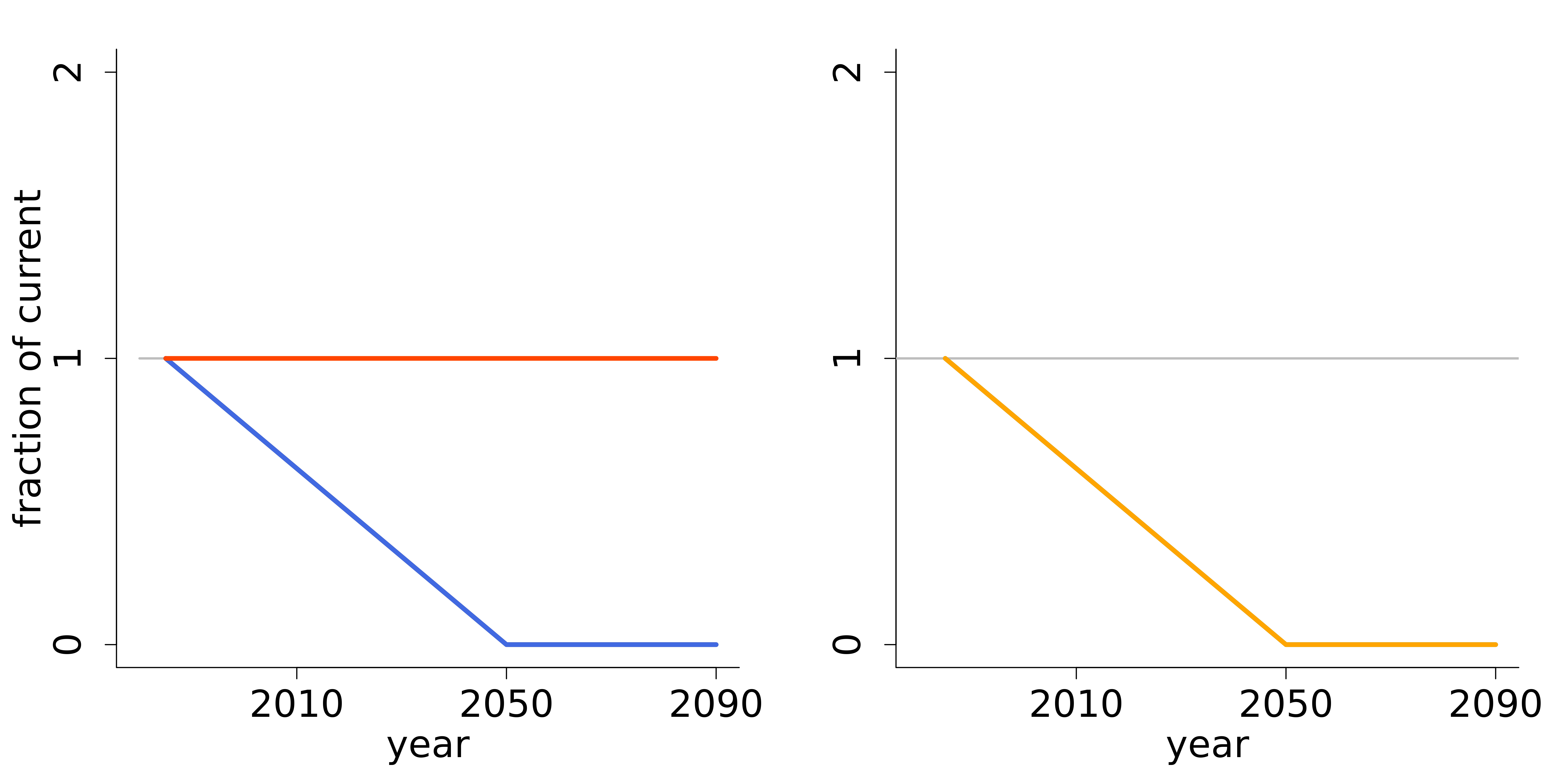

Supplement: S3 Appendix — (ZIP) [file pntd.0014030.s007.zip › Sup. Mat. 6-2 M-Z - Species Trends/Trimeresurus_cantori_CCTrends.png]

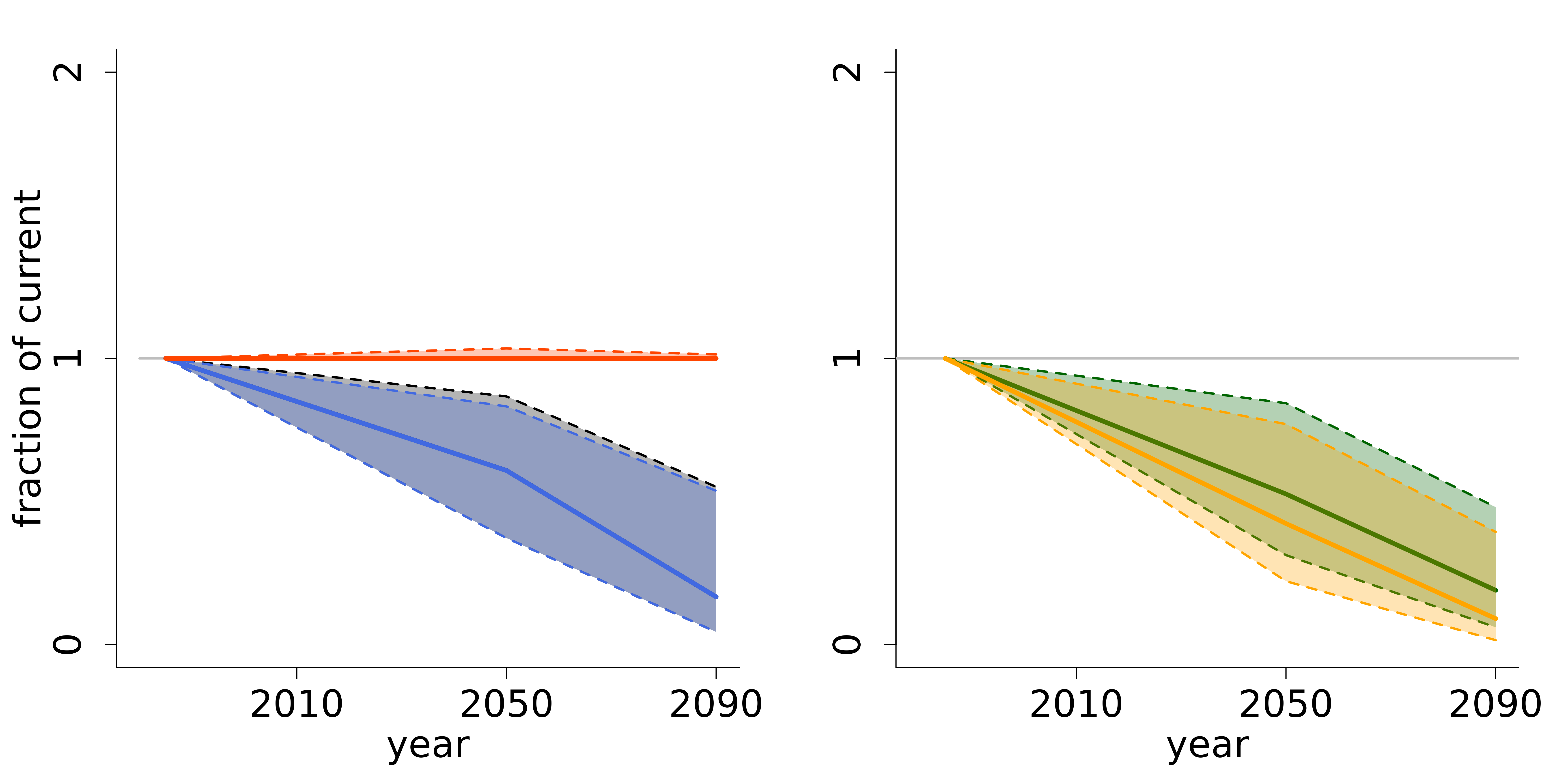

Supplement: S3 Appendix — (ZIP) [file pntd.0014030.s007.zip › Sup. Mat. 6-2 M-Z - Species Trends/Trimeresurus_cardamomensis_CCTrends.png]
