# Supplementary figures and images for: Climate change induced complex shifts in snake distributions expose people to snakebite and threaten biodiversity (part 3 of 4)
Source: PLoS Negl Trop Dis. 2026 May 21;20(5):e0014030. doi: 10.1371/journal.pntd.0014030 (PMC13193456; doi:10.1371/journal.pntd.0014030)

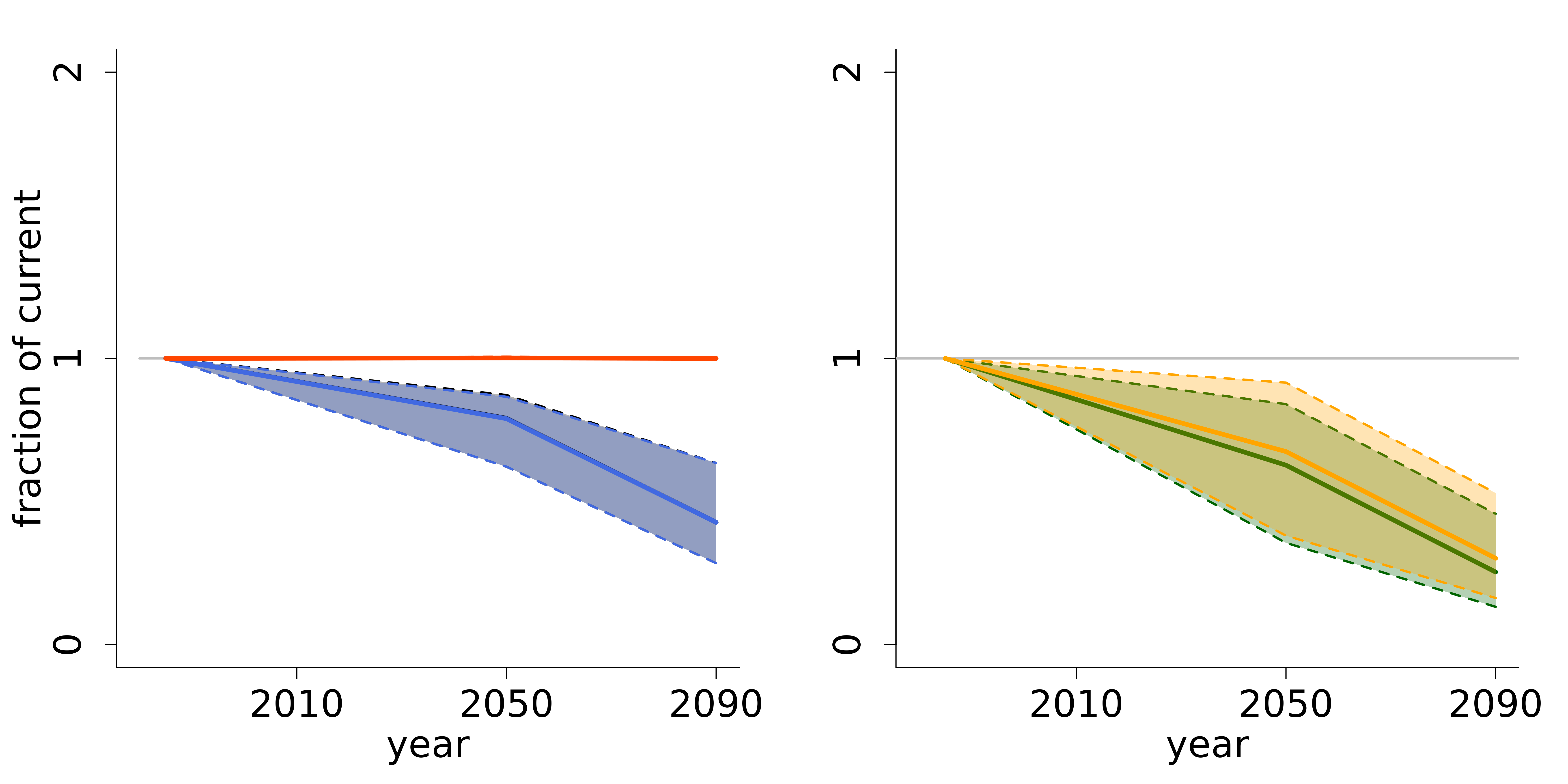

Supplement: S3 Appendix — (ZIP) [file pntd.0014030.s007.zip › Sup. Mat. 6-2 M-Z - Species Trends/Metlapilcoatlus_mexicanus_CCTrends.png]

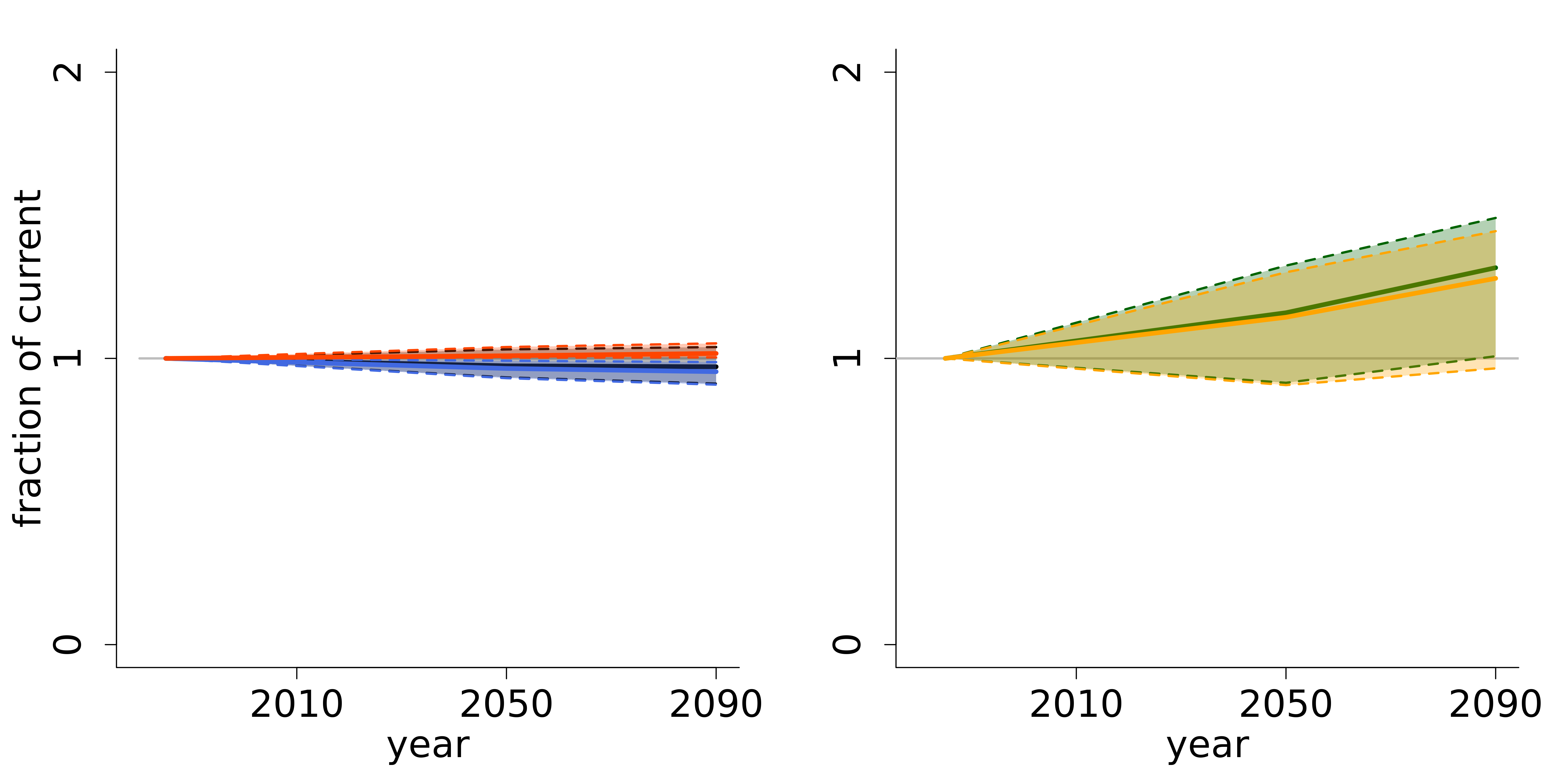

Supplement: S3 Appendix — (ZIP) [file pntd.0014030.s007.zip › Sup. Mat. 6-2 M-Z - Species Trends/Metlapilcoatlus_nummifer_CCTrends.png]

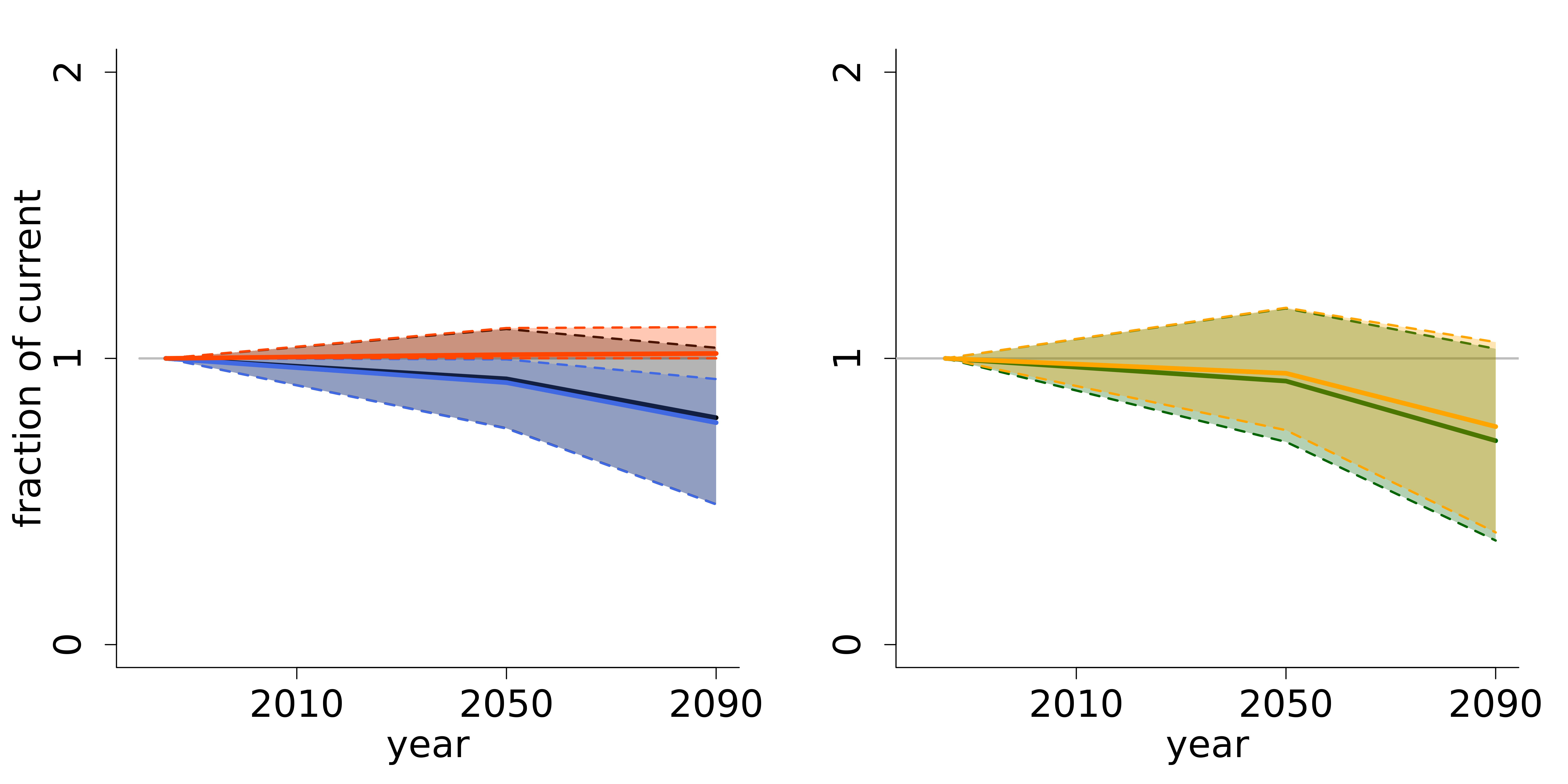

Supplement: S3 Appendix — (ZIP) [file pntd.0014030.s007.zip › Sup. Mat. 6-2 M-Z - Species Trends/Metlapilcoatlus_occiduus_CCTrends.png]

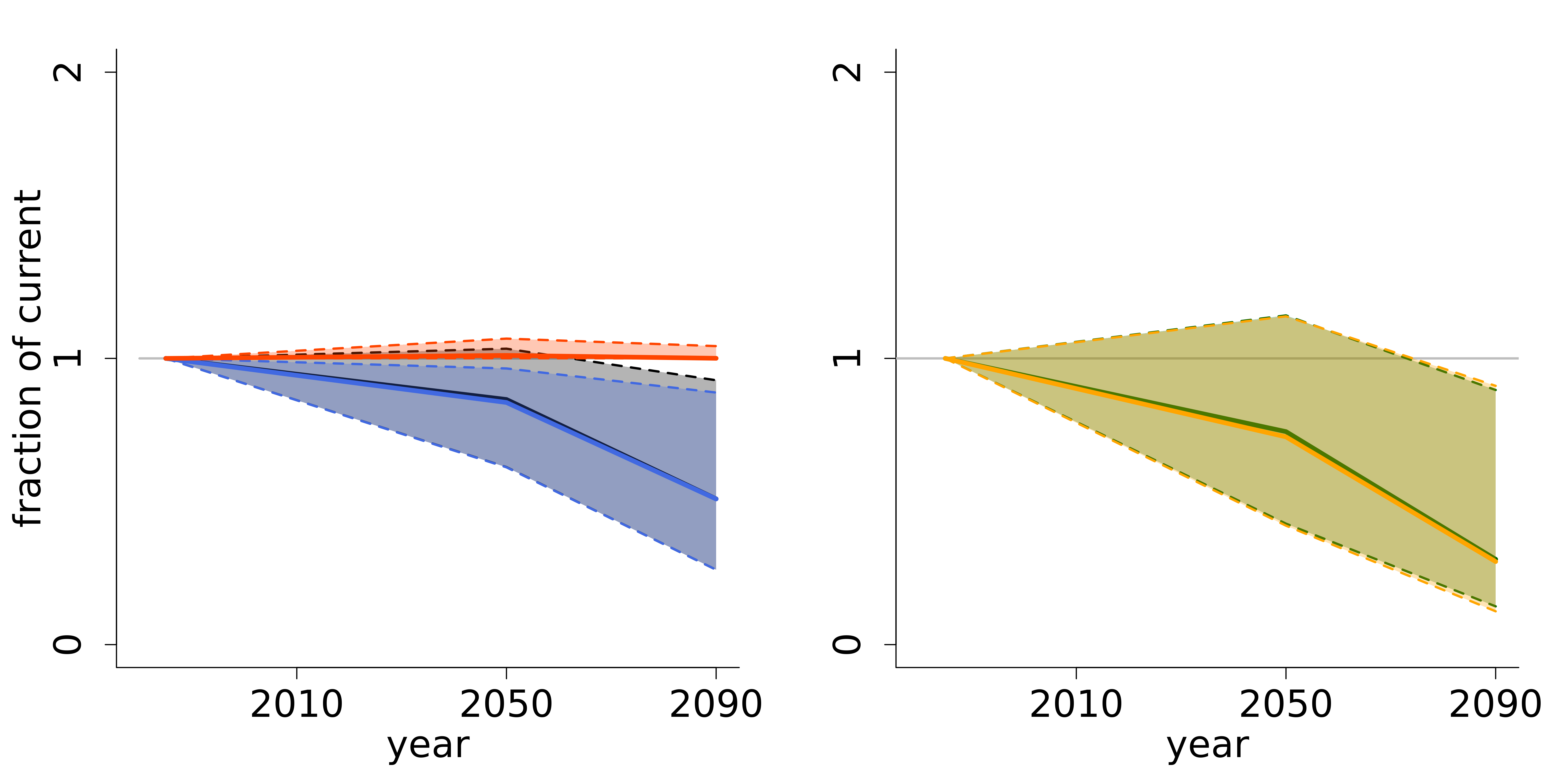

Supplement: S3 Appendix — (ZIP) [file pntd.0014030.s007.zip › Sup. Mat. 6-2 M-Z - Species Trends/Metlapilcoatlus_olmec_CCTrends.png]

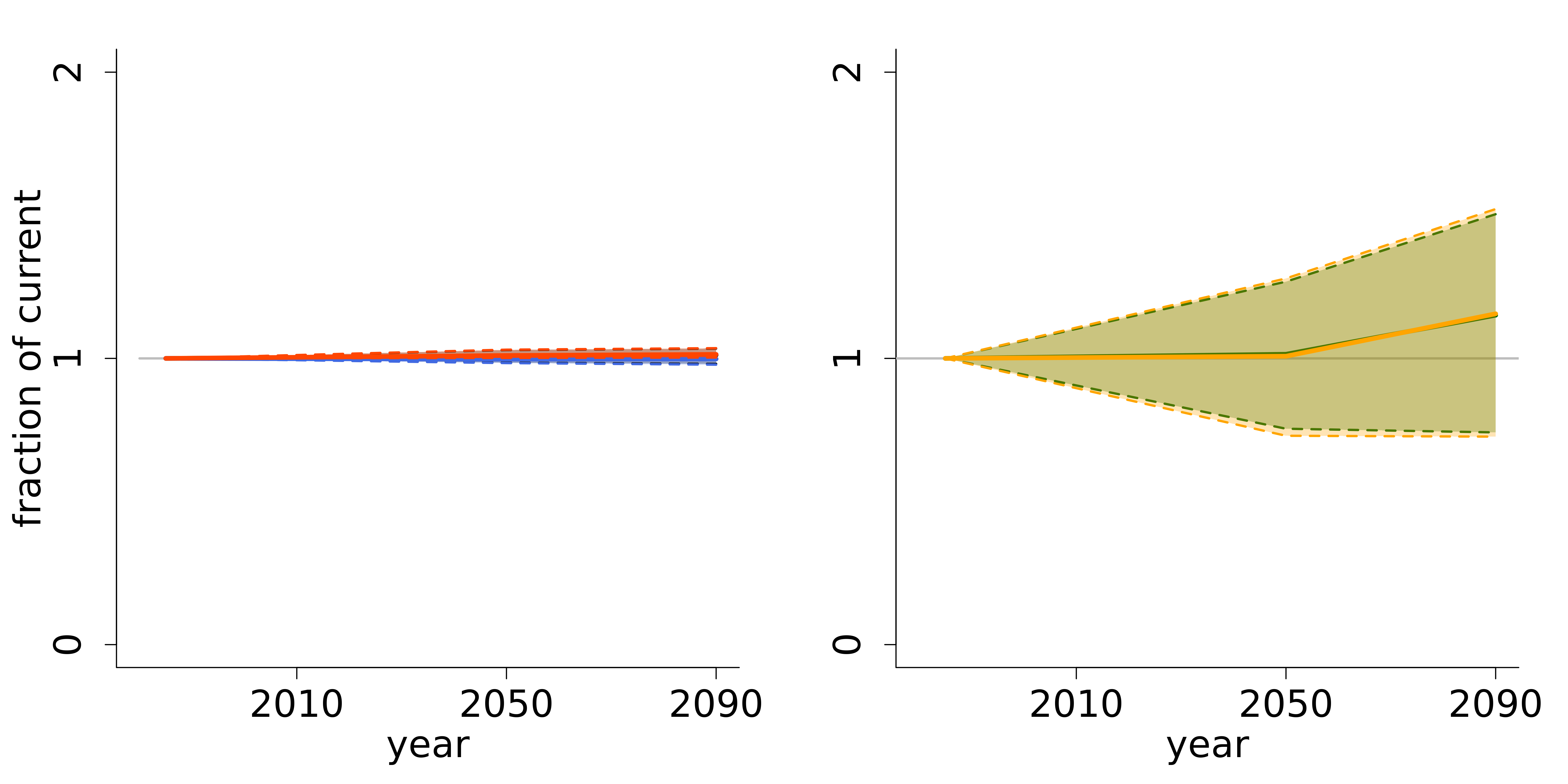

Supplement: S3 Appendix — (ZIP) [file pntd.0014030.s007.zip › Sup. Mat. 6-2 M-Z - Species Trends/Micropechis_ikaheka_CCTrends.png]

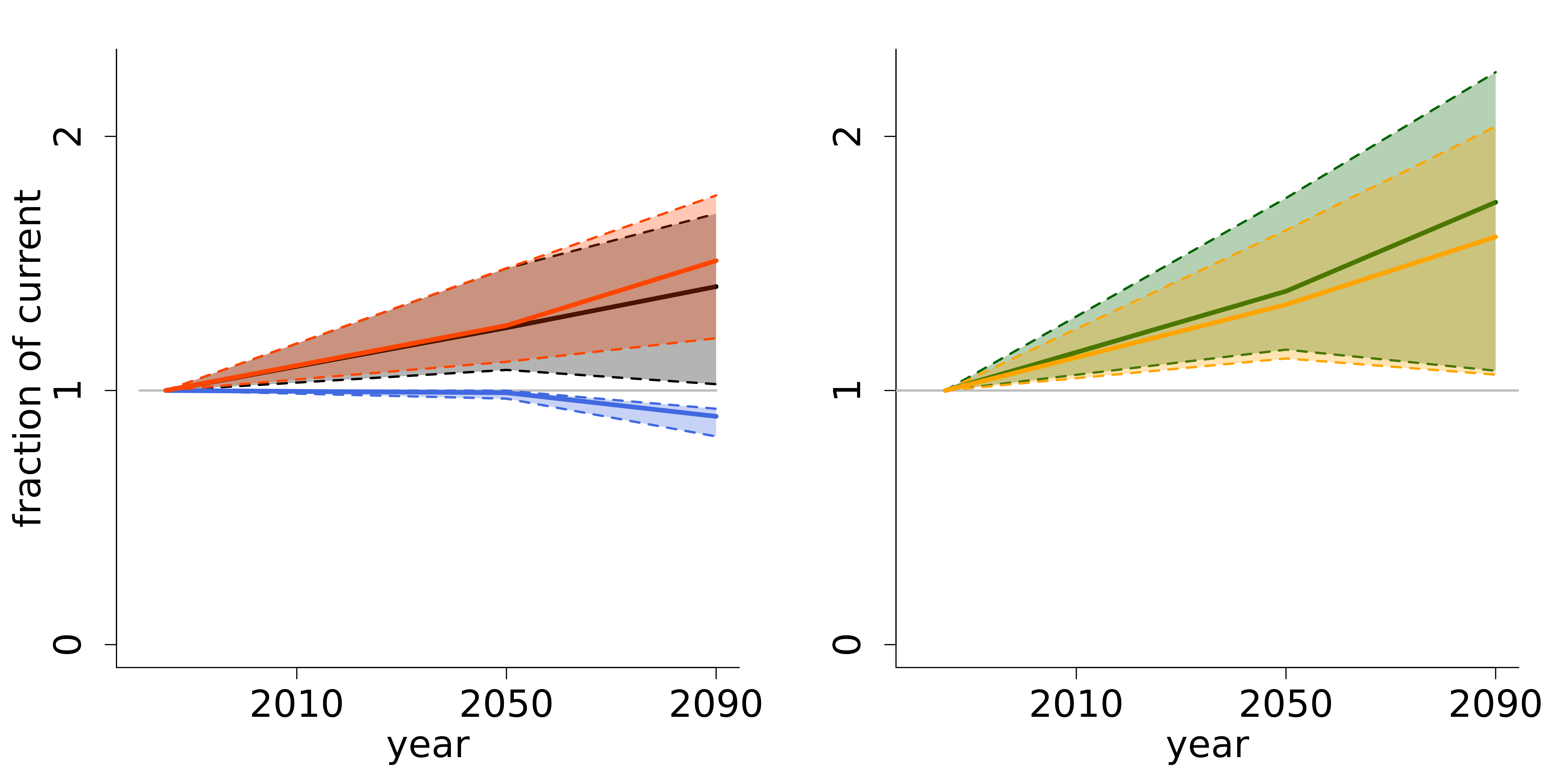

Supplement: S3 Appendix — (ZIP) [file pntd.0014030.s007.zip › Sup. Mat. 6-2 M-Z - Species Trends/Micruroides_euryxanthus_CCTrends.png]

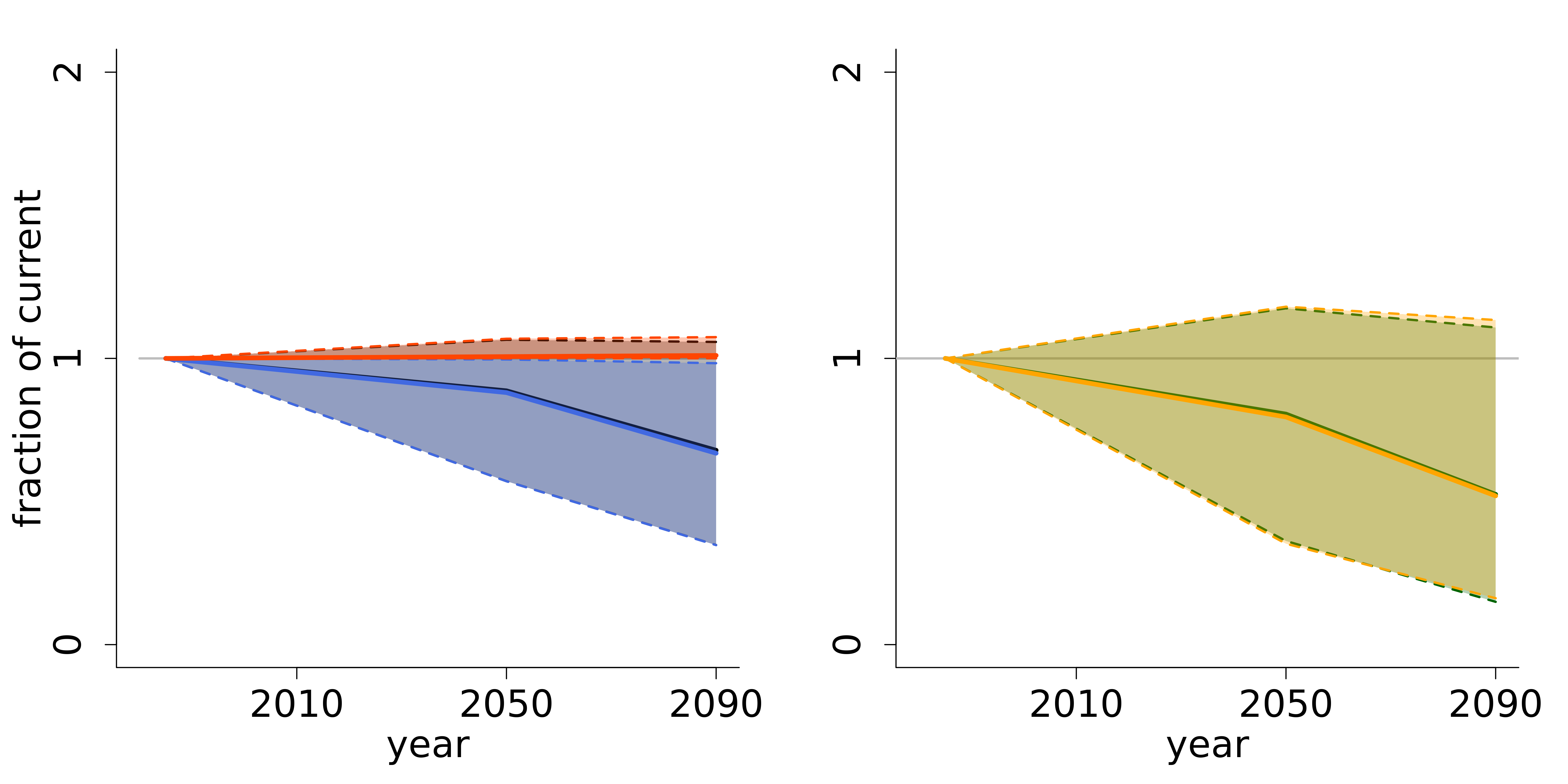

Supplement: S3 Appendix — (ZIP) [file pntd.0014030.s007.zip › Sup. Mat. 6-2 M-Z - Species Trends/Micrurus_albicinctus_CCTrends.png]

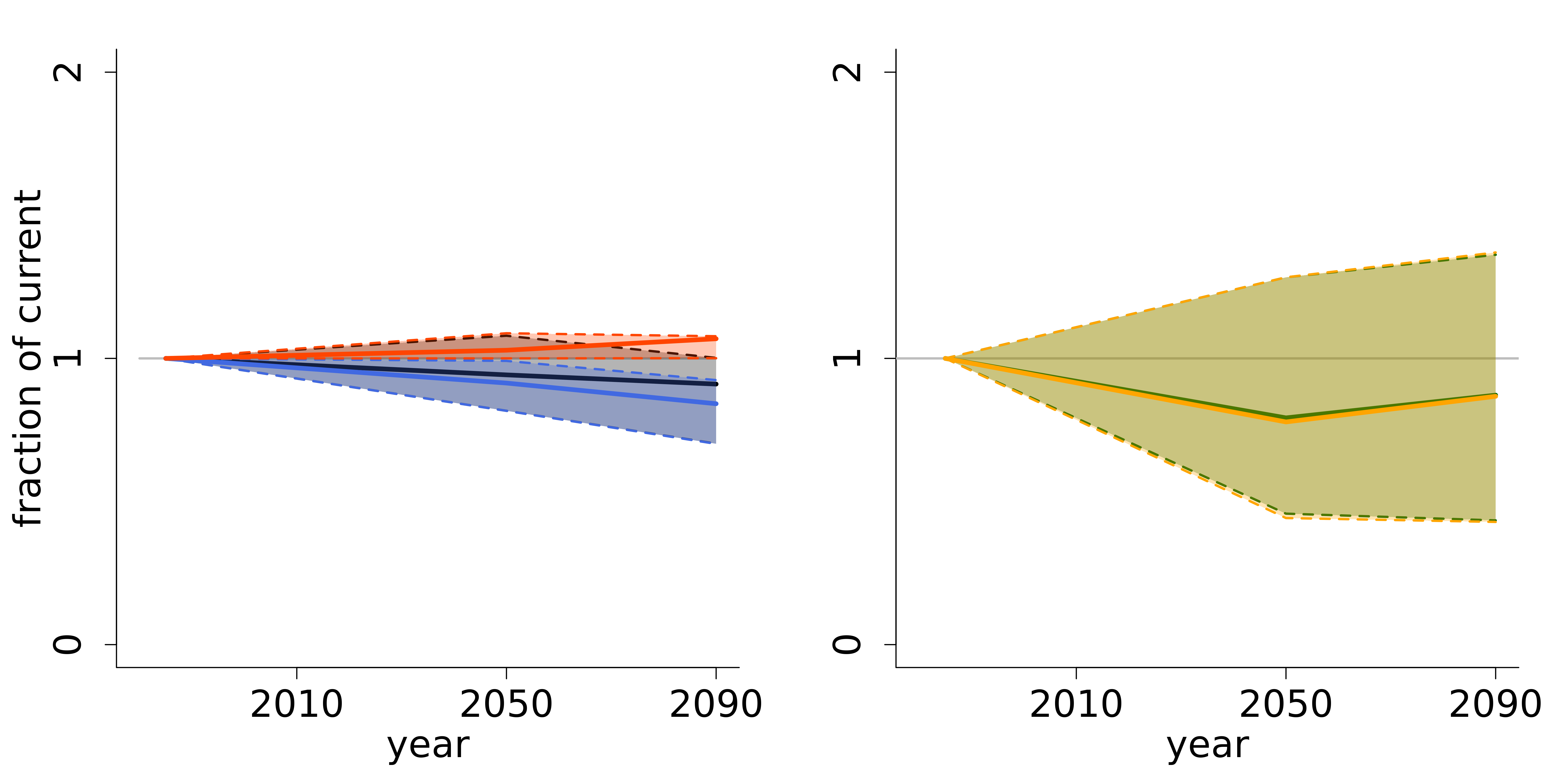

Supplement: S3 Appendix — (ZIP) [file pntd.0014030.s007.zip › Sup. Mat. 6-2 M-Z - Species Trends/Micrurus_alleni_CCTrends.png]

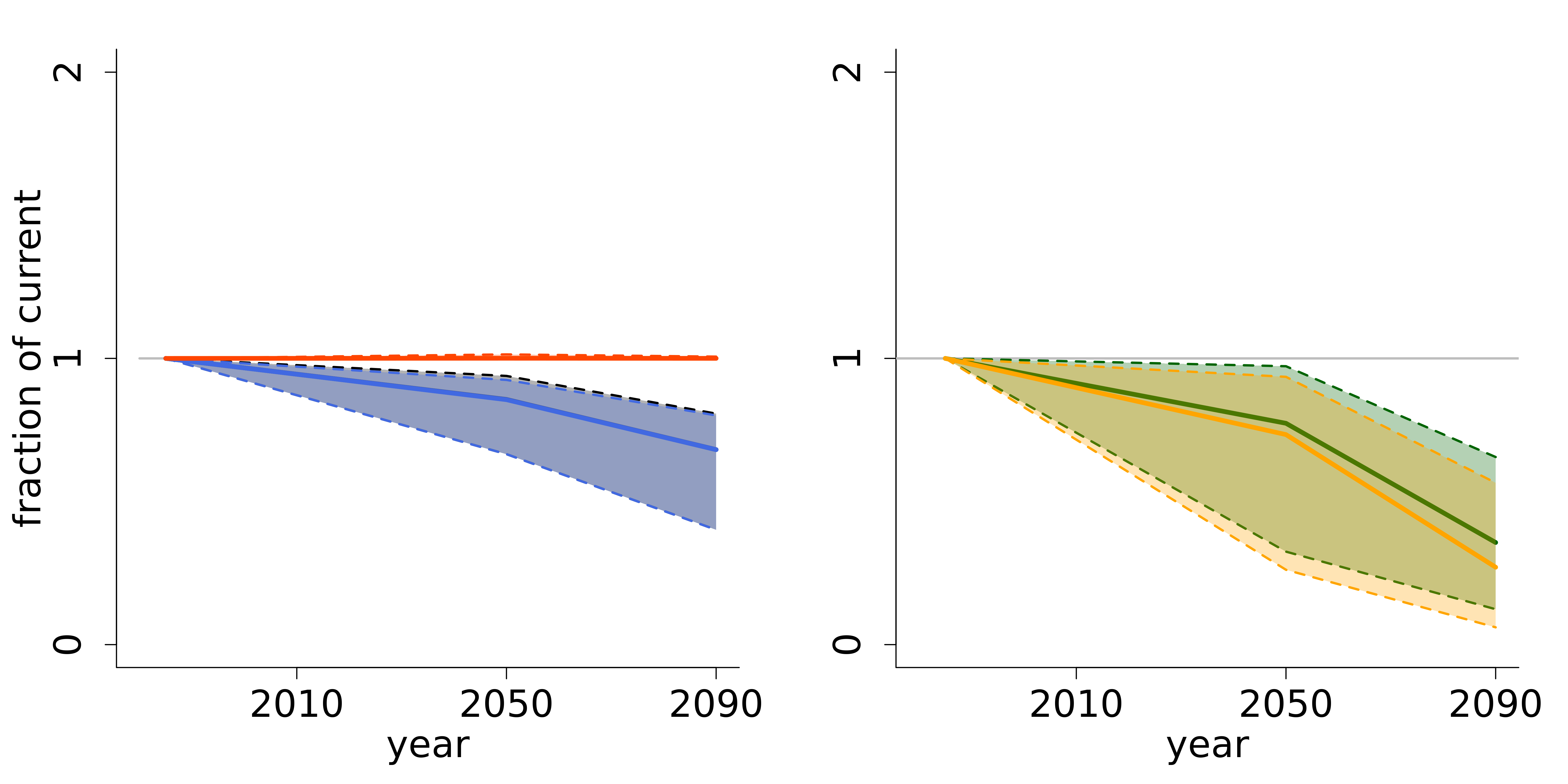

Supplement: S3 Appendix — (ZIP) [file pntd.0014030.s007.zip › Sup. Mat. 6-2 M-Z - Species Trends/Micrurus_altirostris_CCTrends.png]

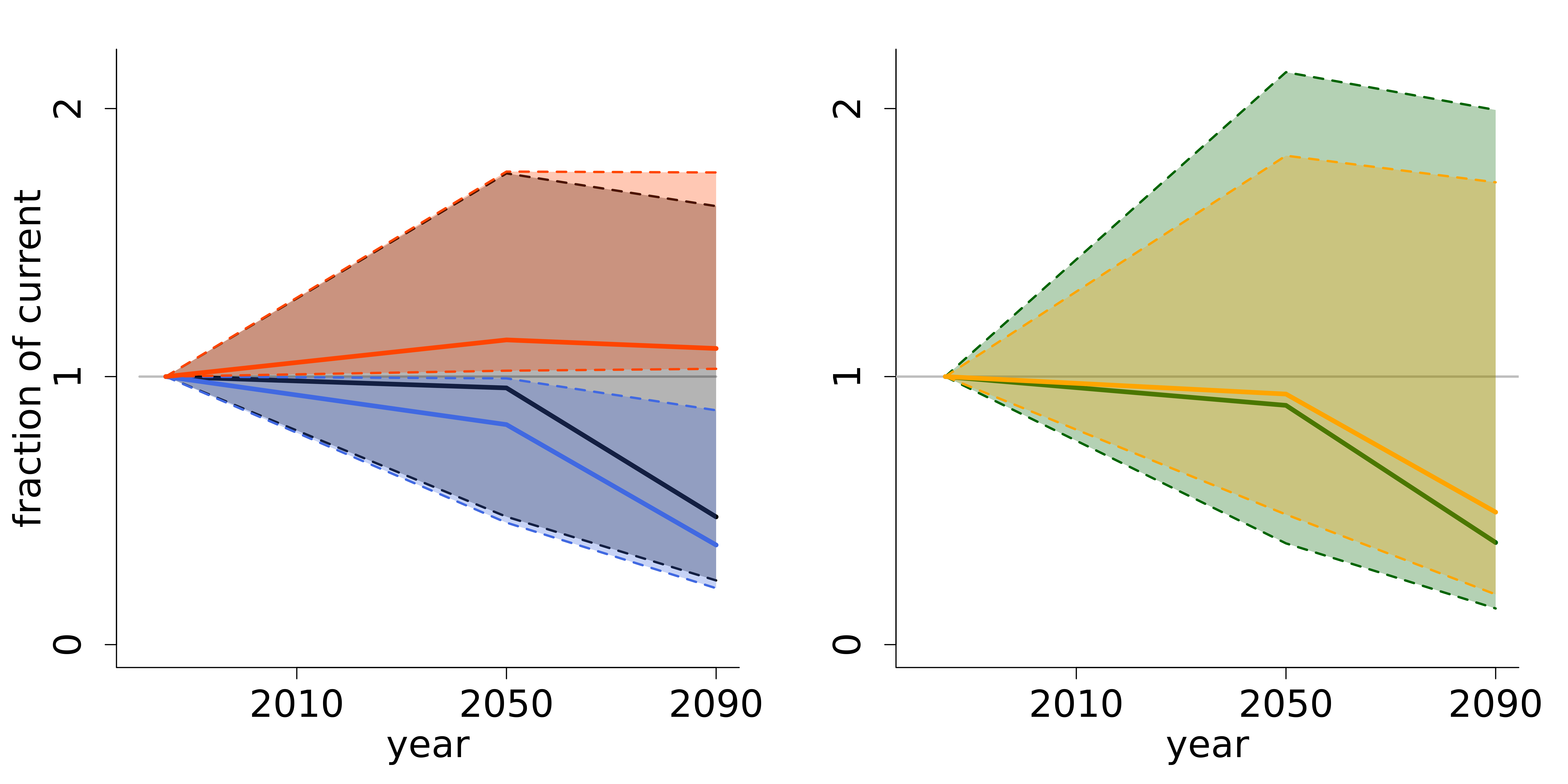

Supplement: S3 Appendix — (ZIP) [file pntd.0014030.s007.zip › Sup. Mat. 6-2 M-Z - Species Trends/Micrurus_ancoralis_CCTrends.png]

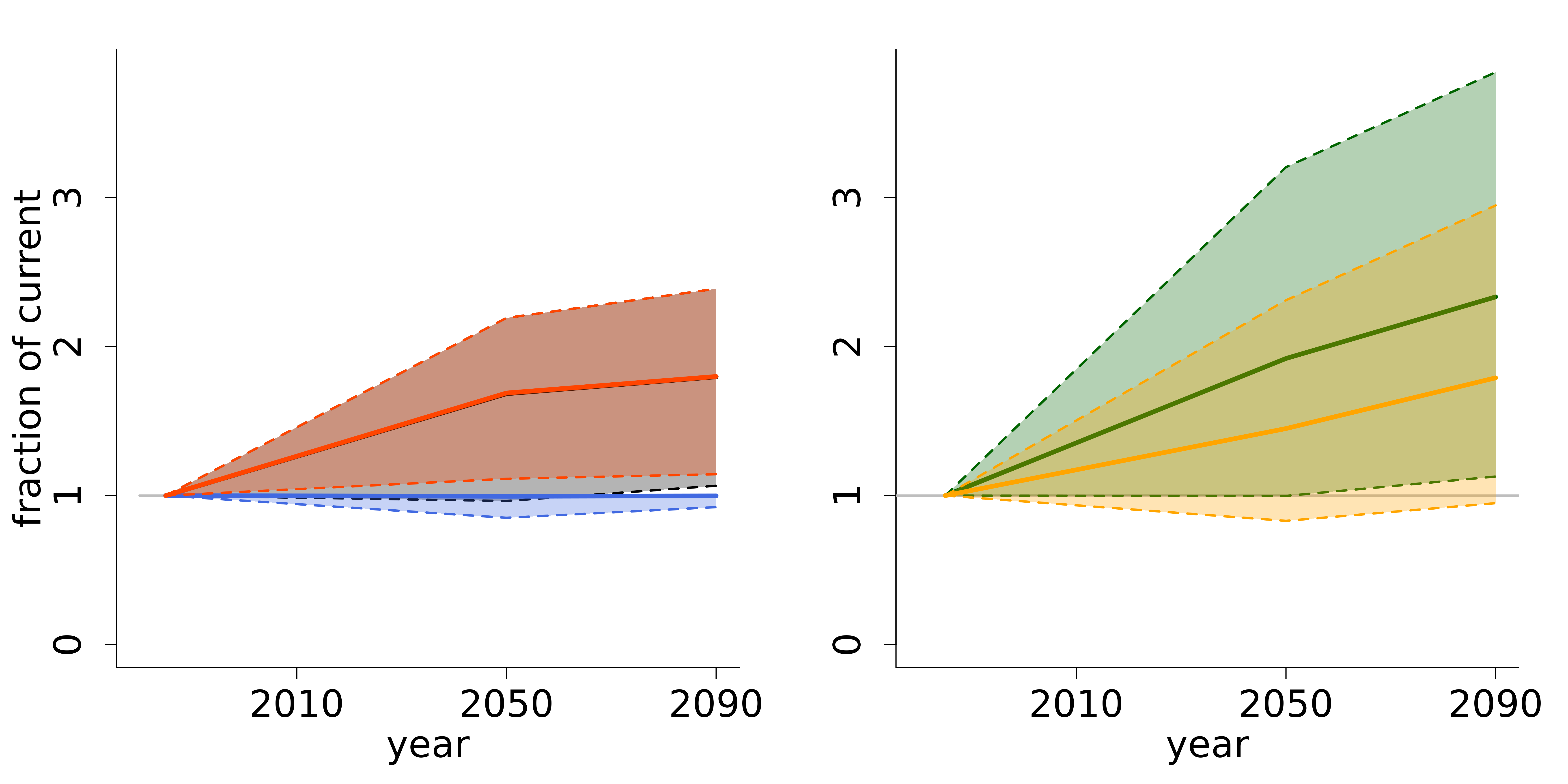

Supplement: S3 Appendix — (ZIP) [file pntd.0014030.s007.zip › Sup. Mat. 6-2 M-Z - Species Trends/Micrurus_annellatus_CCTrends.png]

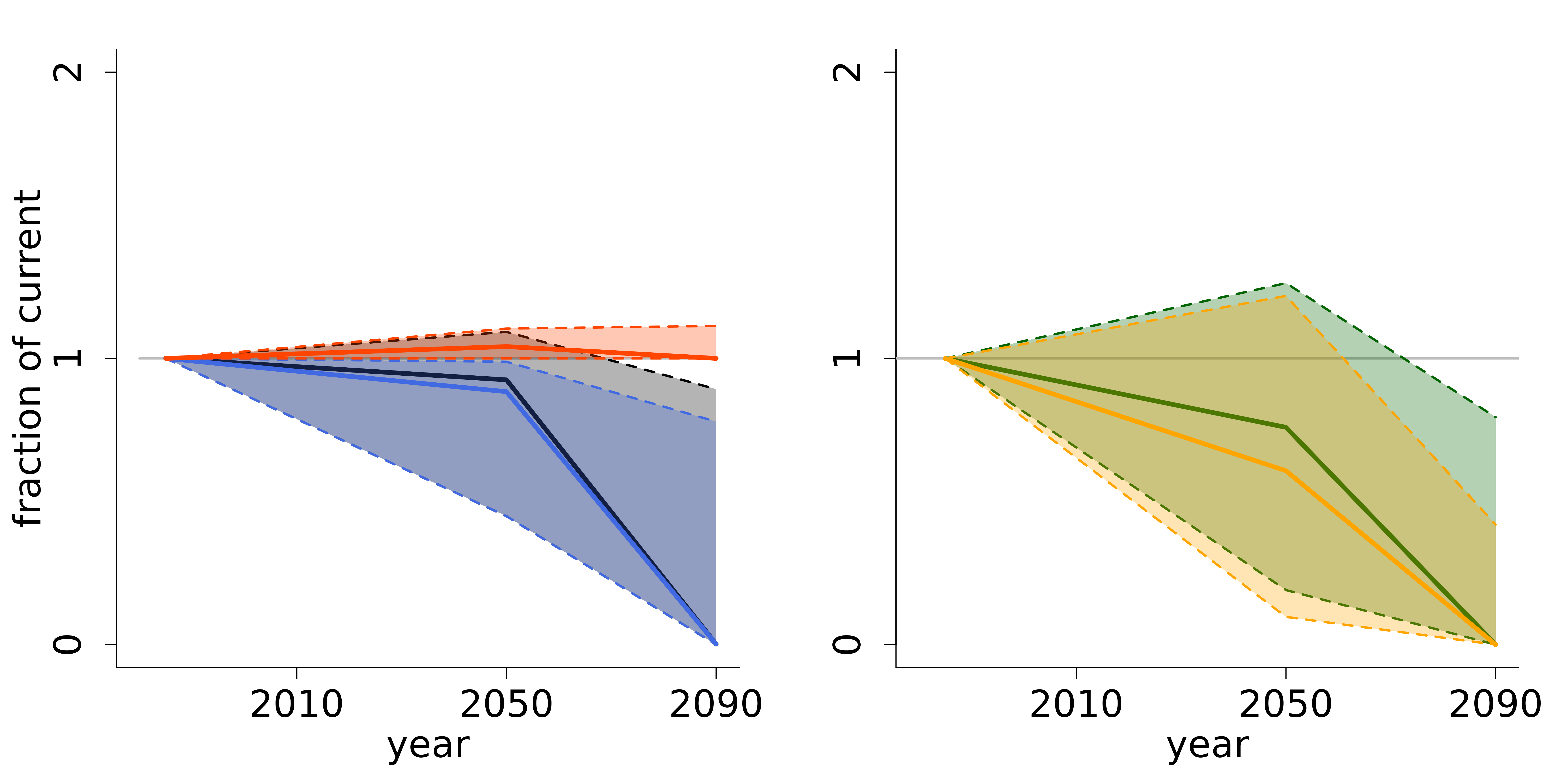

Supplement: S3 Appendix — (ZIP) [file pntd.0014030.s007.zip › Sup. Mat. 6-2 M-Z - Species Trends/Micrurus_averyi_CCTrends.png]

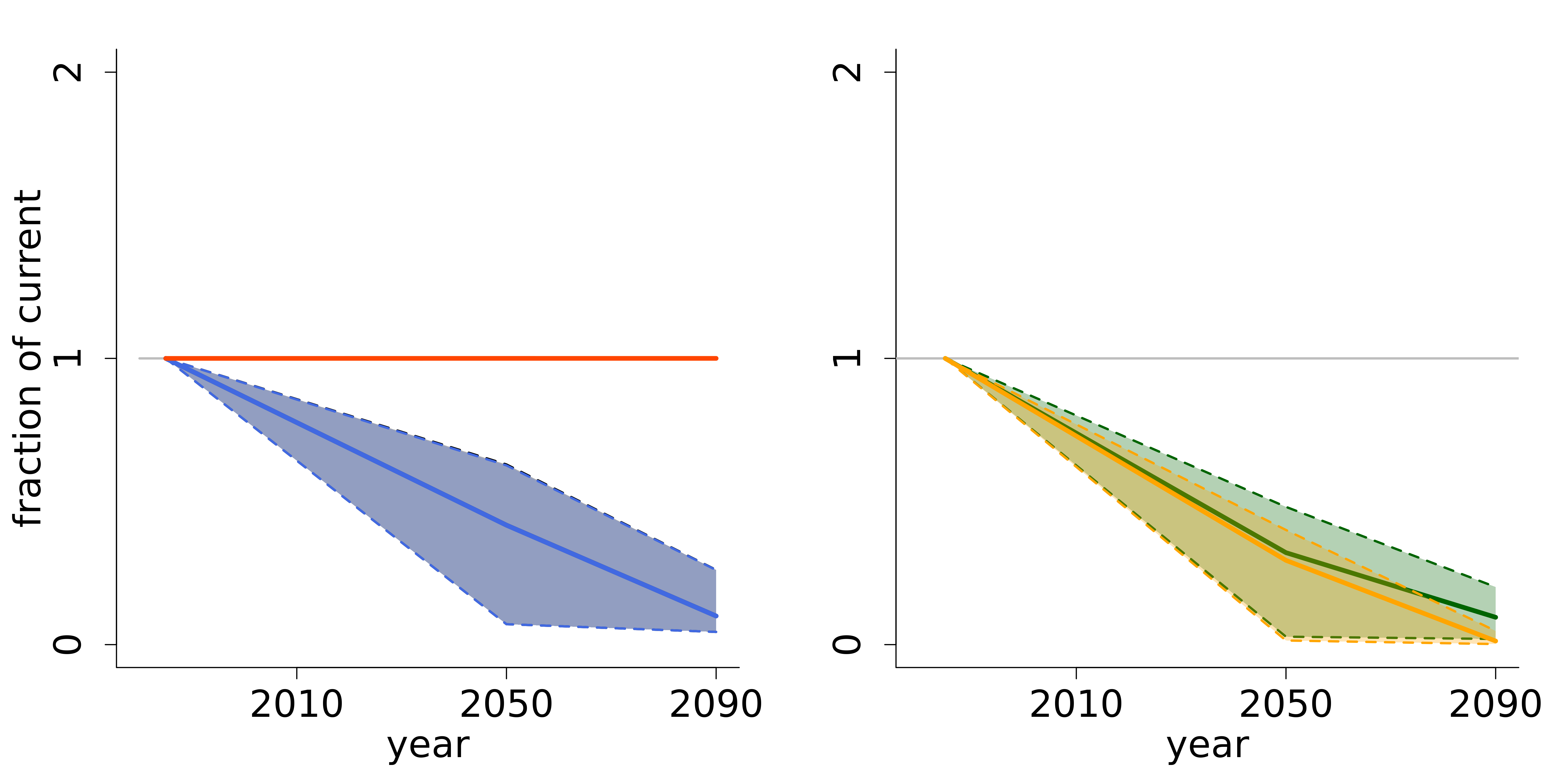

Supplement: S3 Appendix — (ZIP) [file pntd.0014030.s007.zip › Sup. Mat. 6-2 M-Z - Species Trends/Micrurus_baliocoryphus_CCTrends.png]

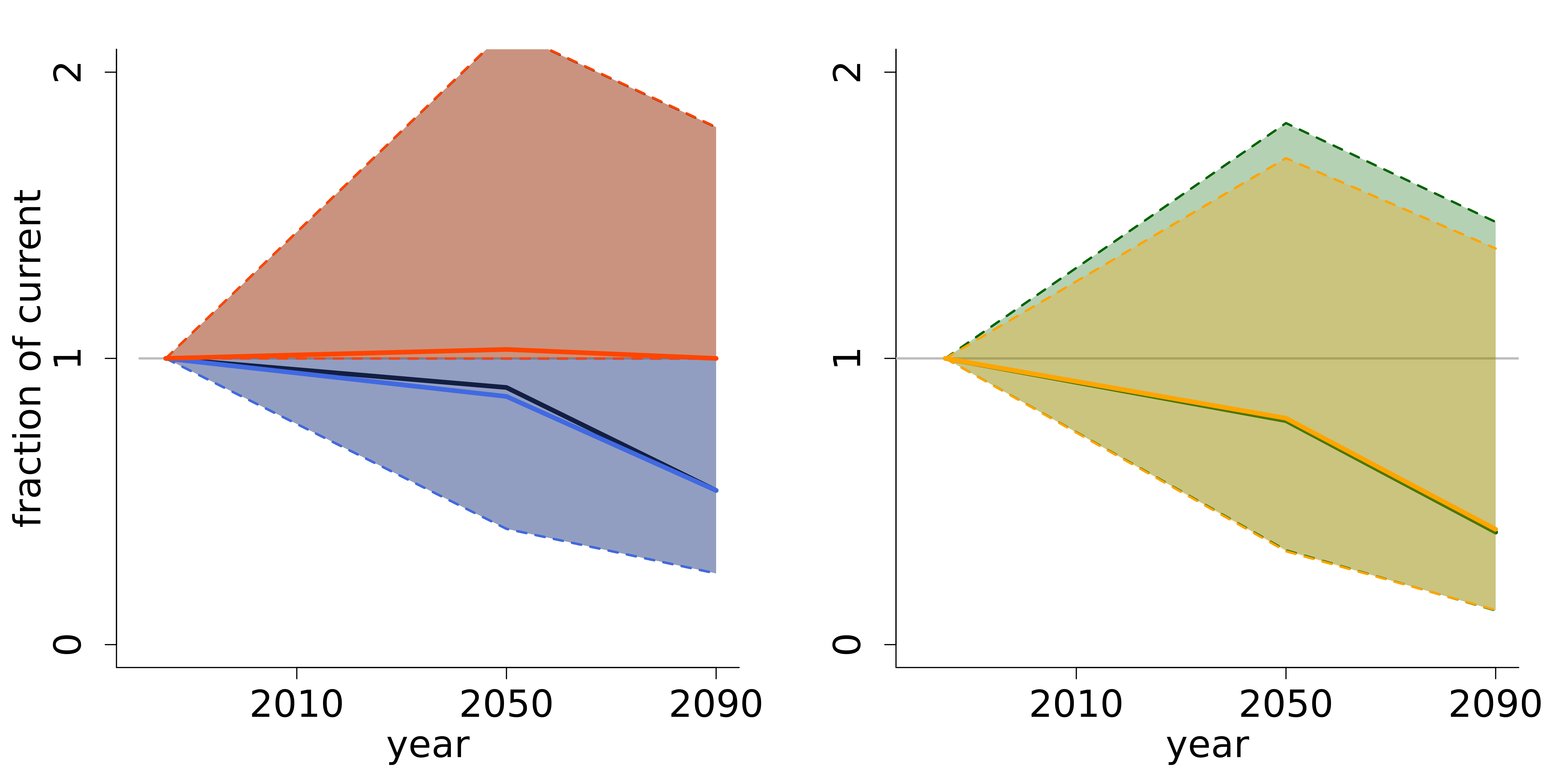

Supplement: S3 Appendix — (ZIP) [file pntd.0014030.s007.zip › Sup. Mat. 6-2 M-Z - Species Trends/Micrurus_bernadi_CCTrends.png]

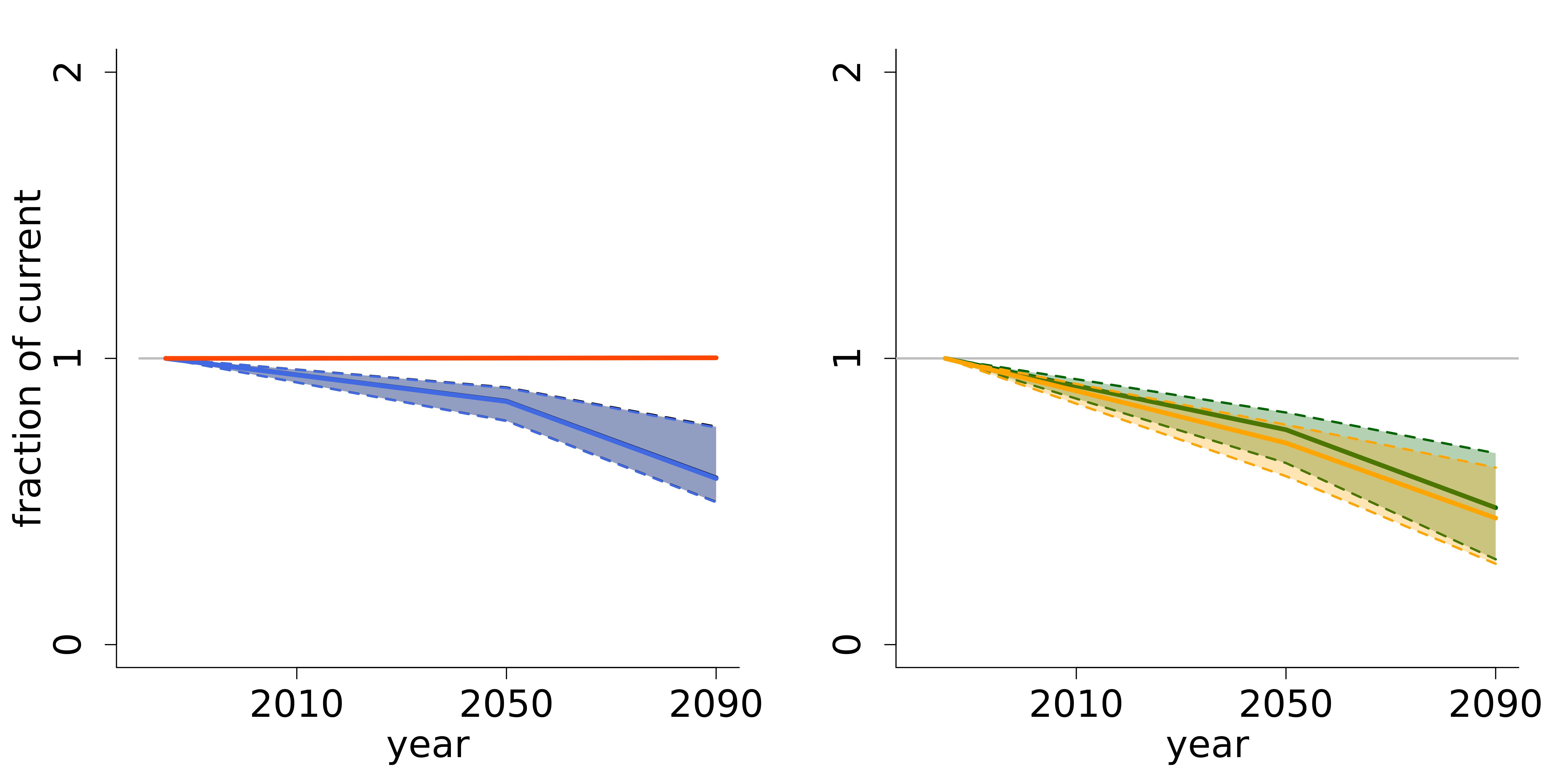

Supplement: S3 Appendix — (ZIP) [file pntd.0014030.s007.zip › Sup. Mat. 6-2 M-Z - Species Trends/Micrurus_bocourti_CCTrends.png]

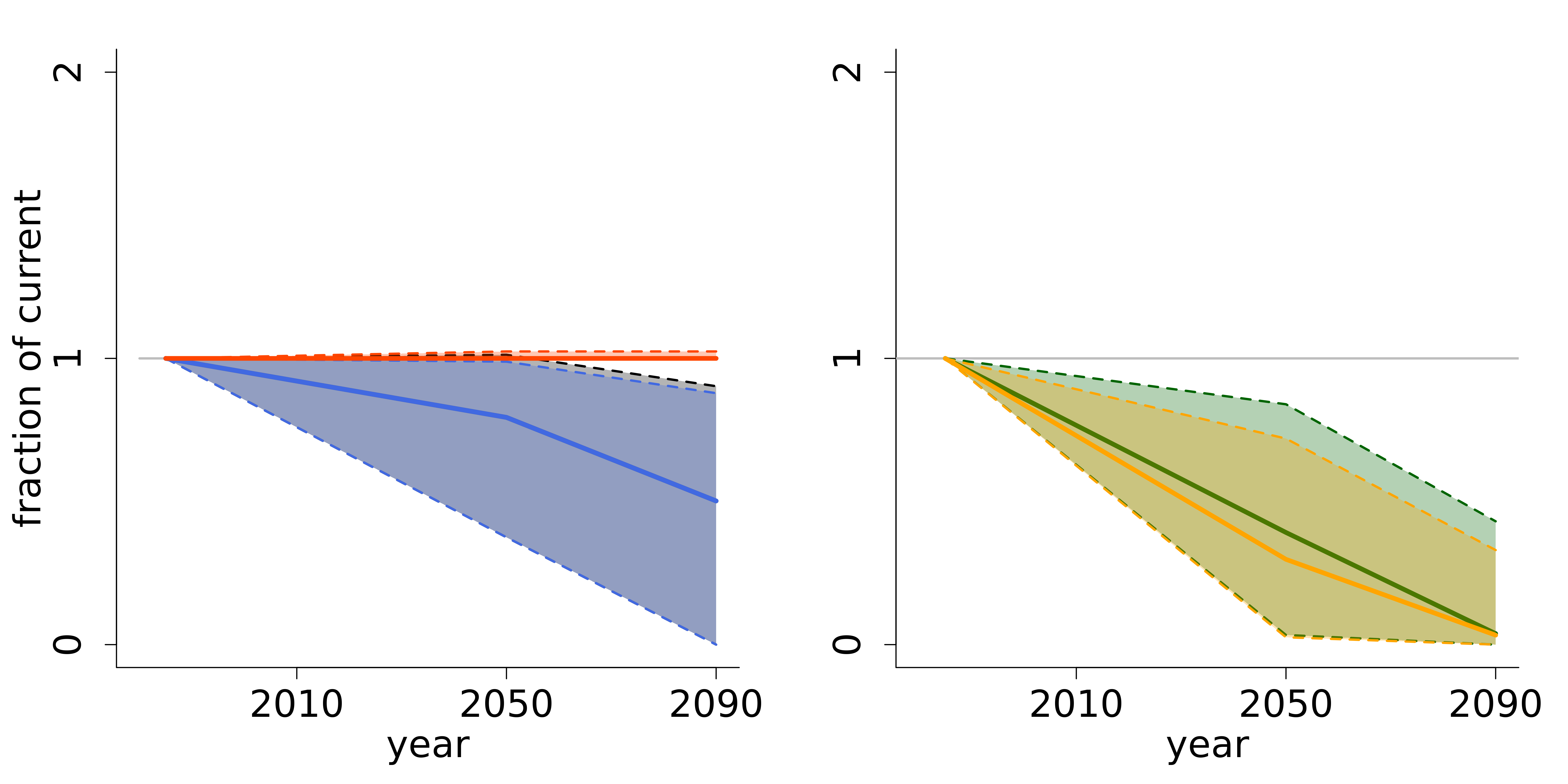

Supplement: S3 Appendix — (ZIP) [file pntd.0014030.s007.zip › Sup. Mat. 6-2 M-Z - Species Trends/Micrurus_boicora_CCTrends.png]

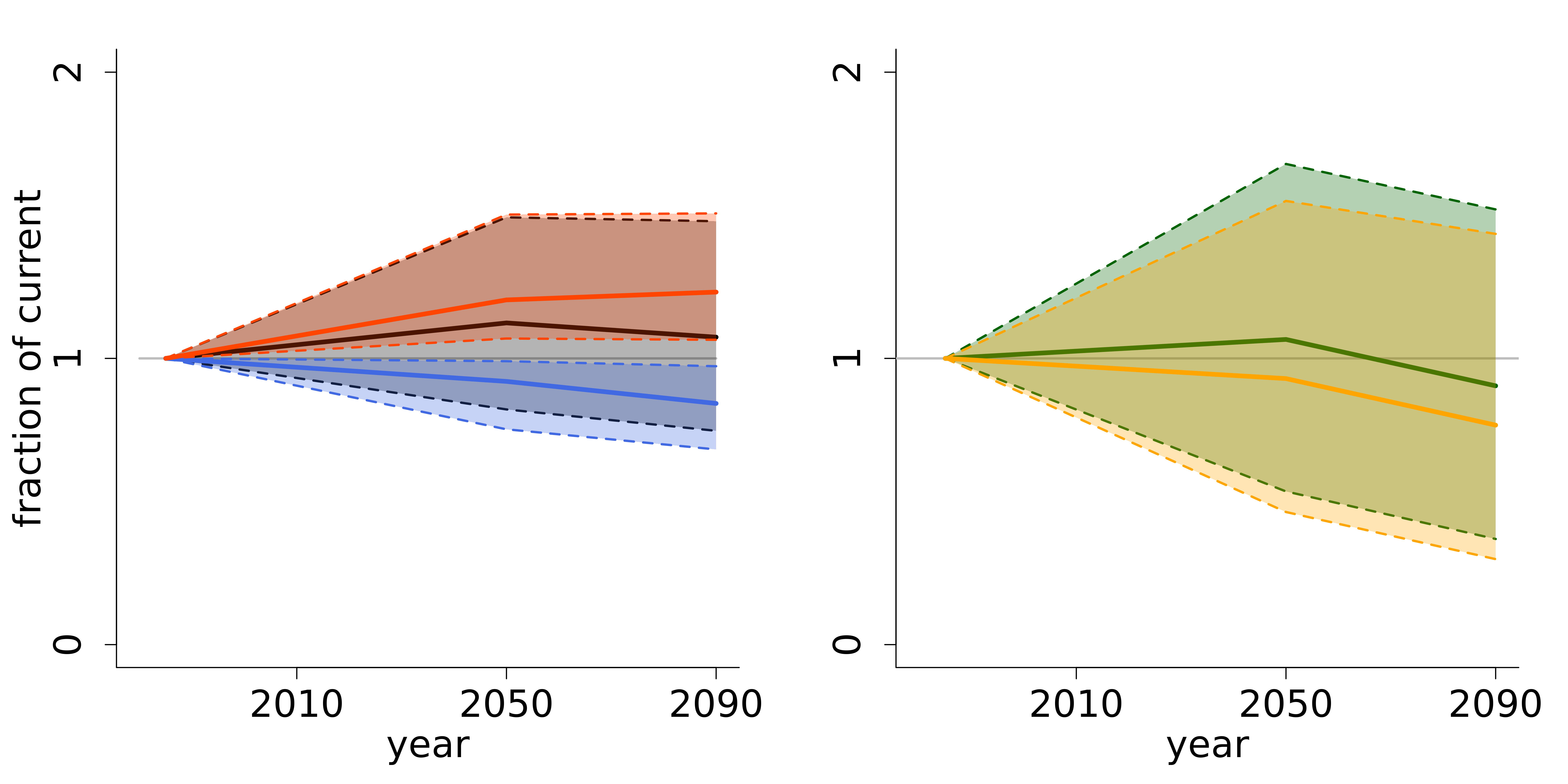

Supplement: S3 Appendix — (ZIP) [file pntd.0014030.s007.zip › Sup. Mat. 6-2 M-Z - Species Trends/Micrurus_brasiliensis_CCTrends.png]

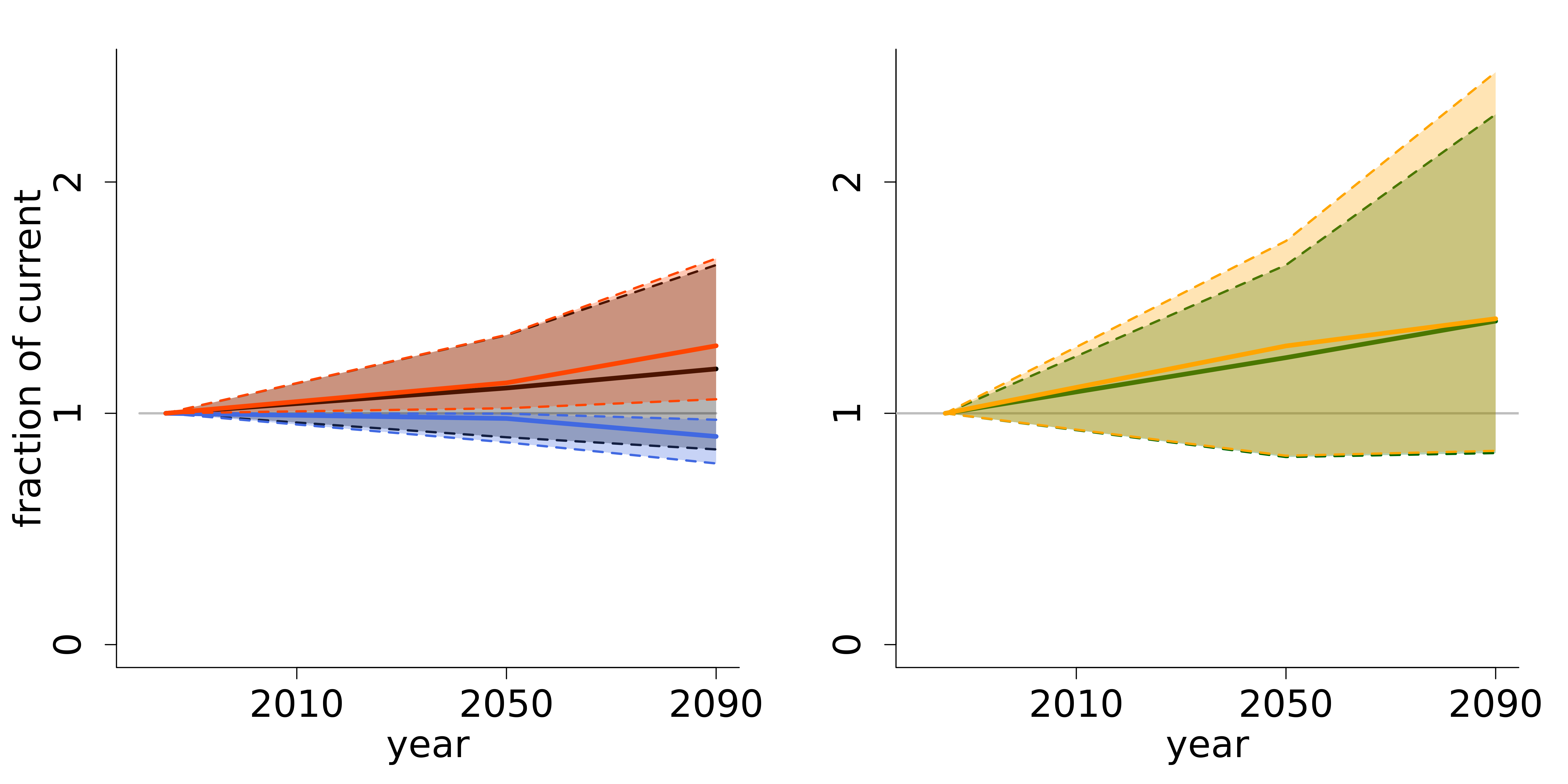

Supplement: S3 Appendix — (ZIP) [file pntd.0014030.s007.zip › Sup. Mat. 6-2 M-Z - Species Trends/Micrurus_browni_CCTrends.png]

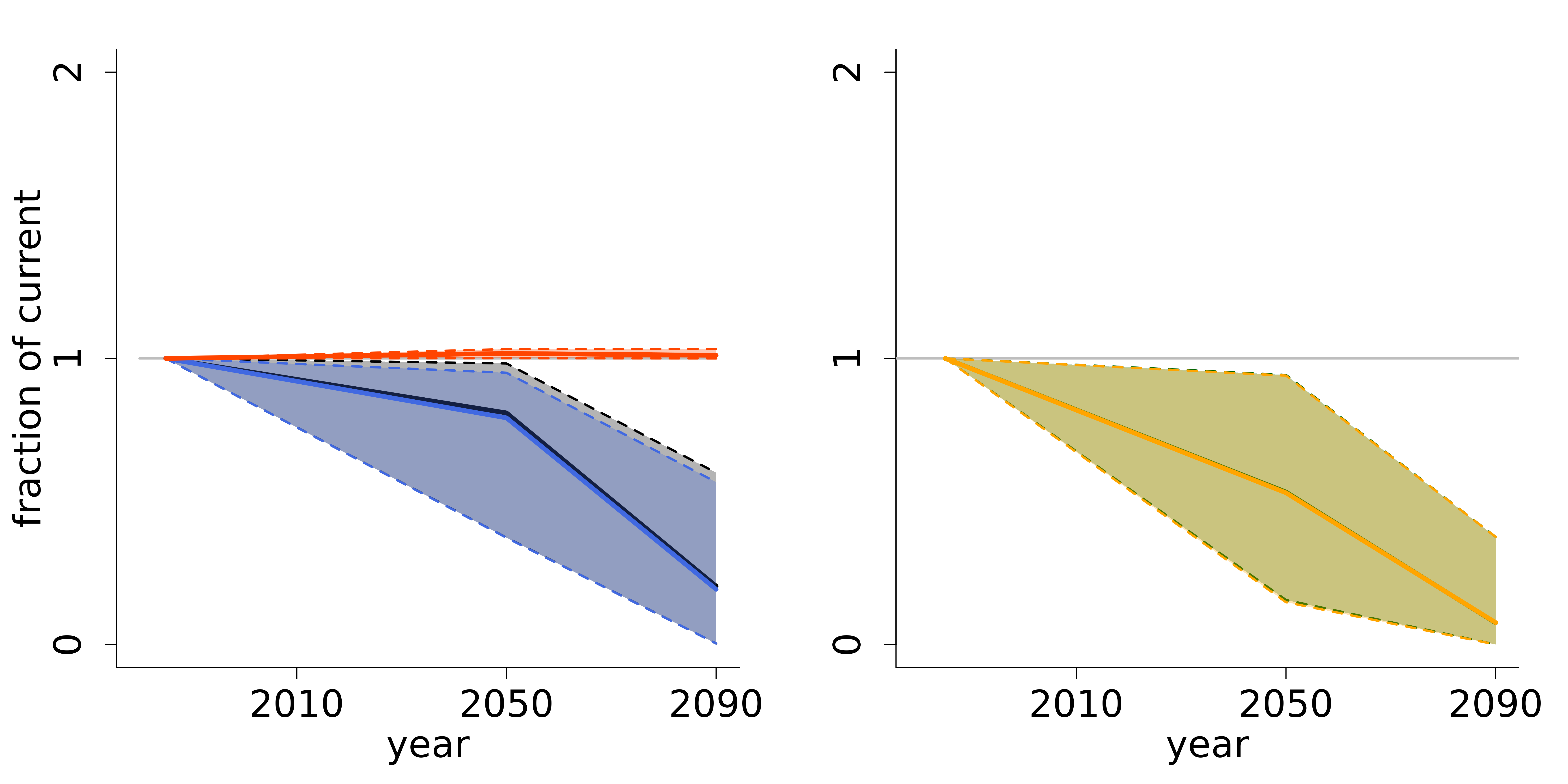

Supplement: S3 Appendix — (ZIP) [file pntd.0014030.s007.zip › Sup. Mat. 6-2 M-Z - Species Trends/Micrurus_camilae_CCTrends.png]

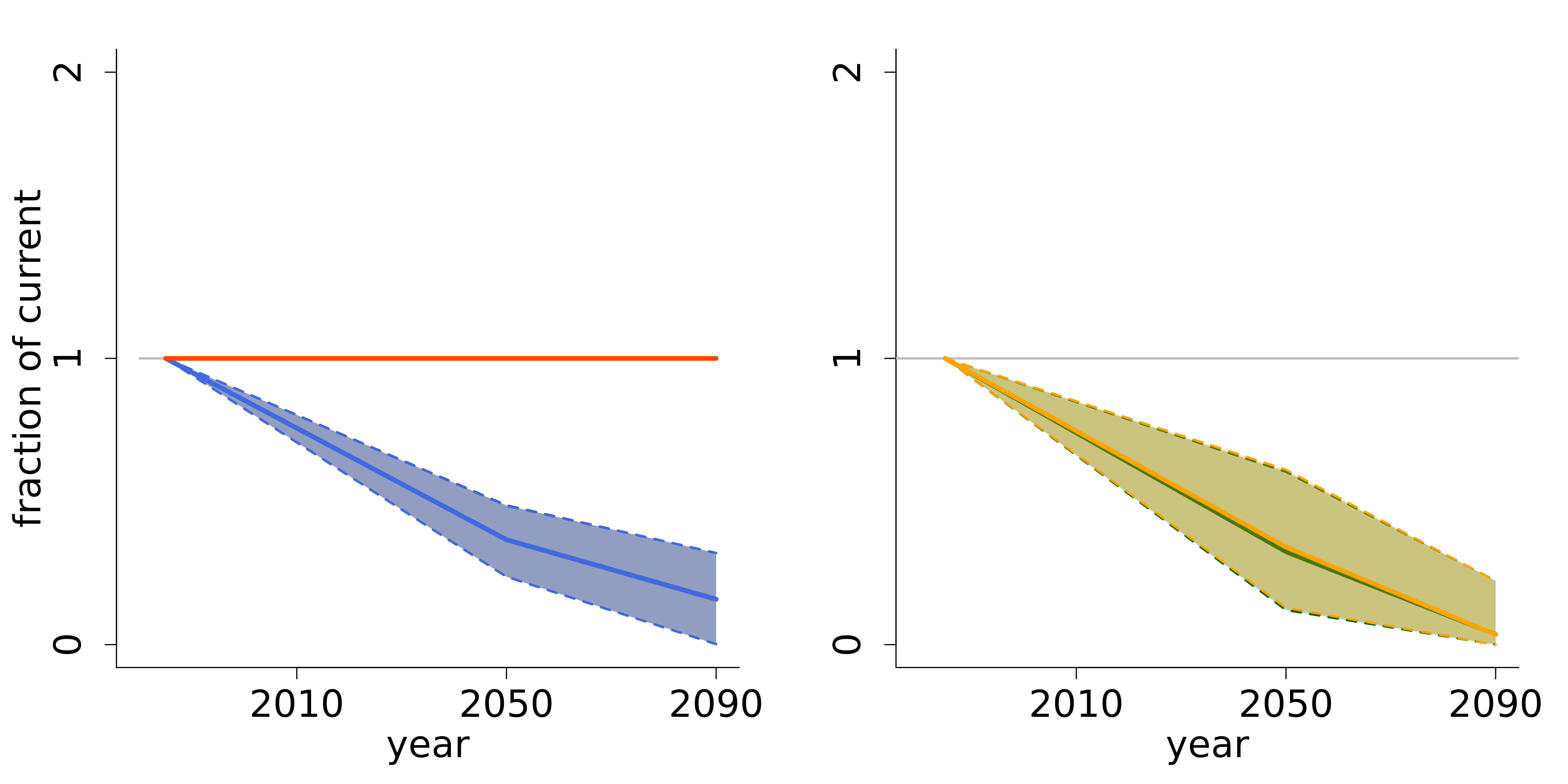

Supplement: S3 Appendix — (ZIP) [file pntd.0014030.s007.zip › Sup. Mat. 6-2 M-Z - Species Trends/Micrurus_circinalis_CCTrends.png]

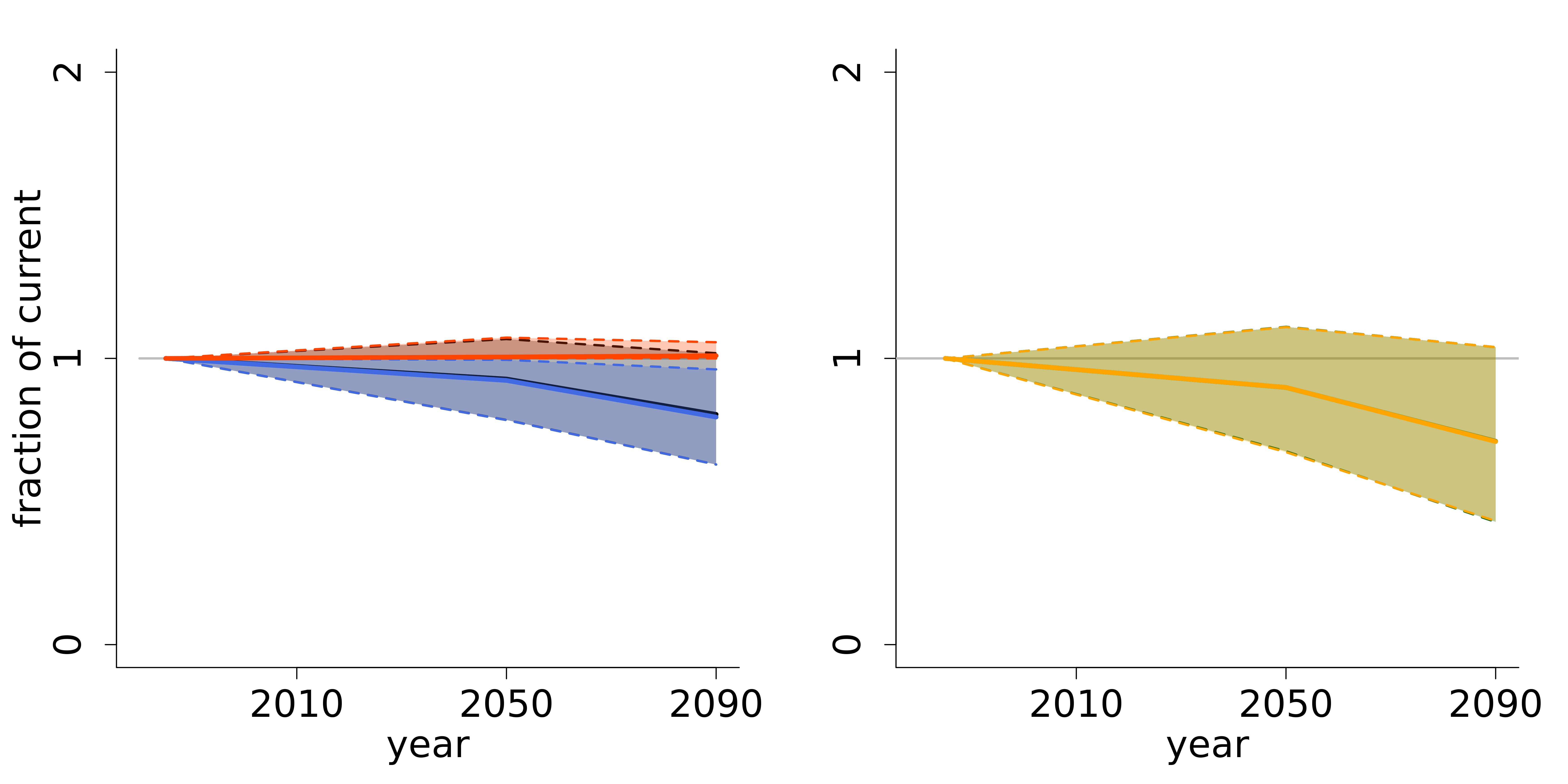

Supplement: S3 Appendix — (ZIP) [file pntd.0014030.s007.zip › Sup. Mat. 6-2 M-Z - Species Trends/Micrurus_clarki_CCTrends.png]

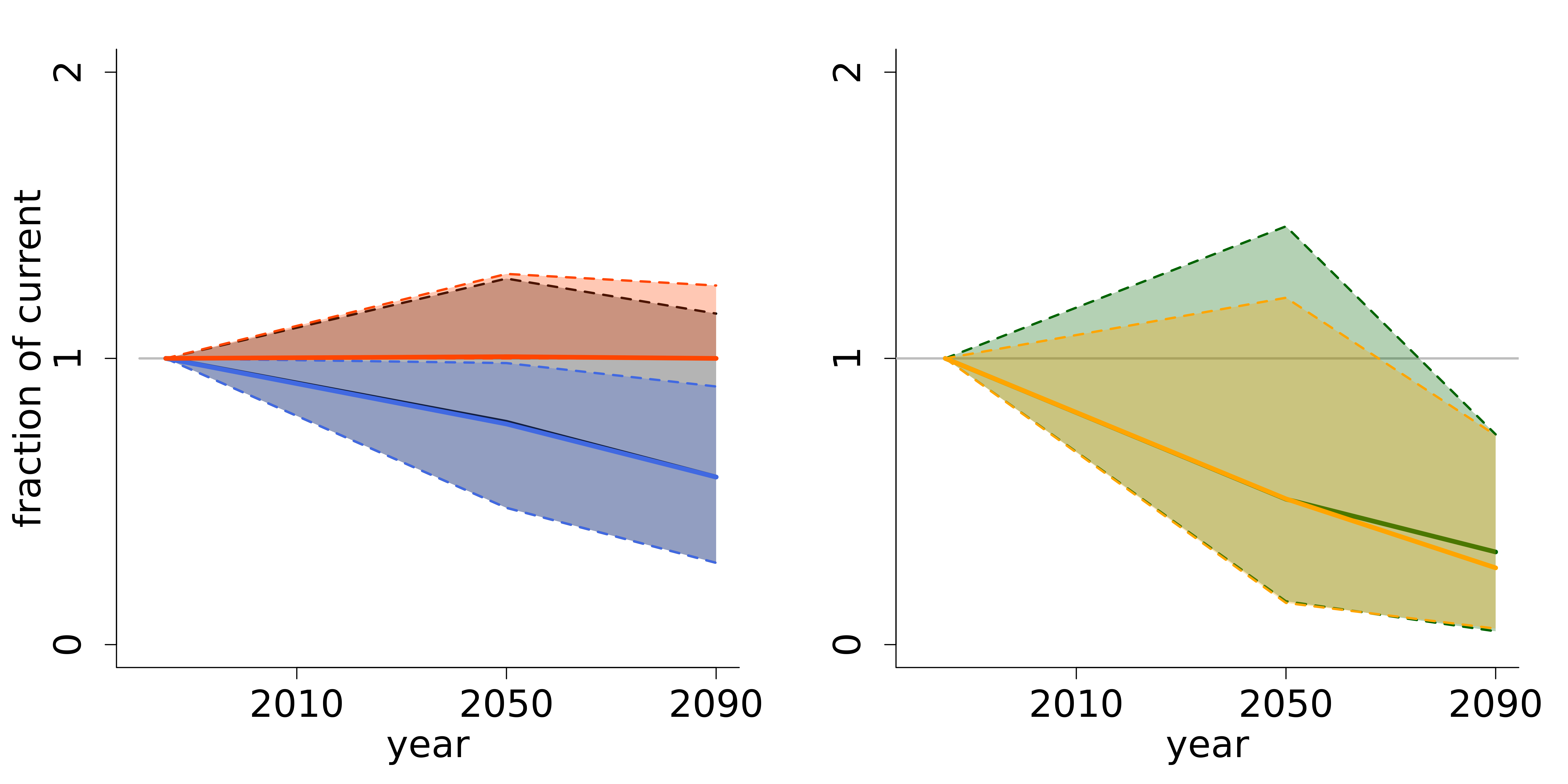

Supplement: S3 Appendix — (ZIP) [file pntd.0014030.s007.zip › Sup. Mat. 6-2 M-Z - Species Trends/Micrurus_collaris_CCTrends.png]

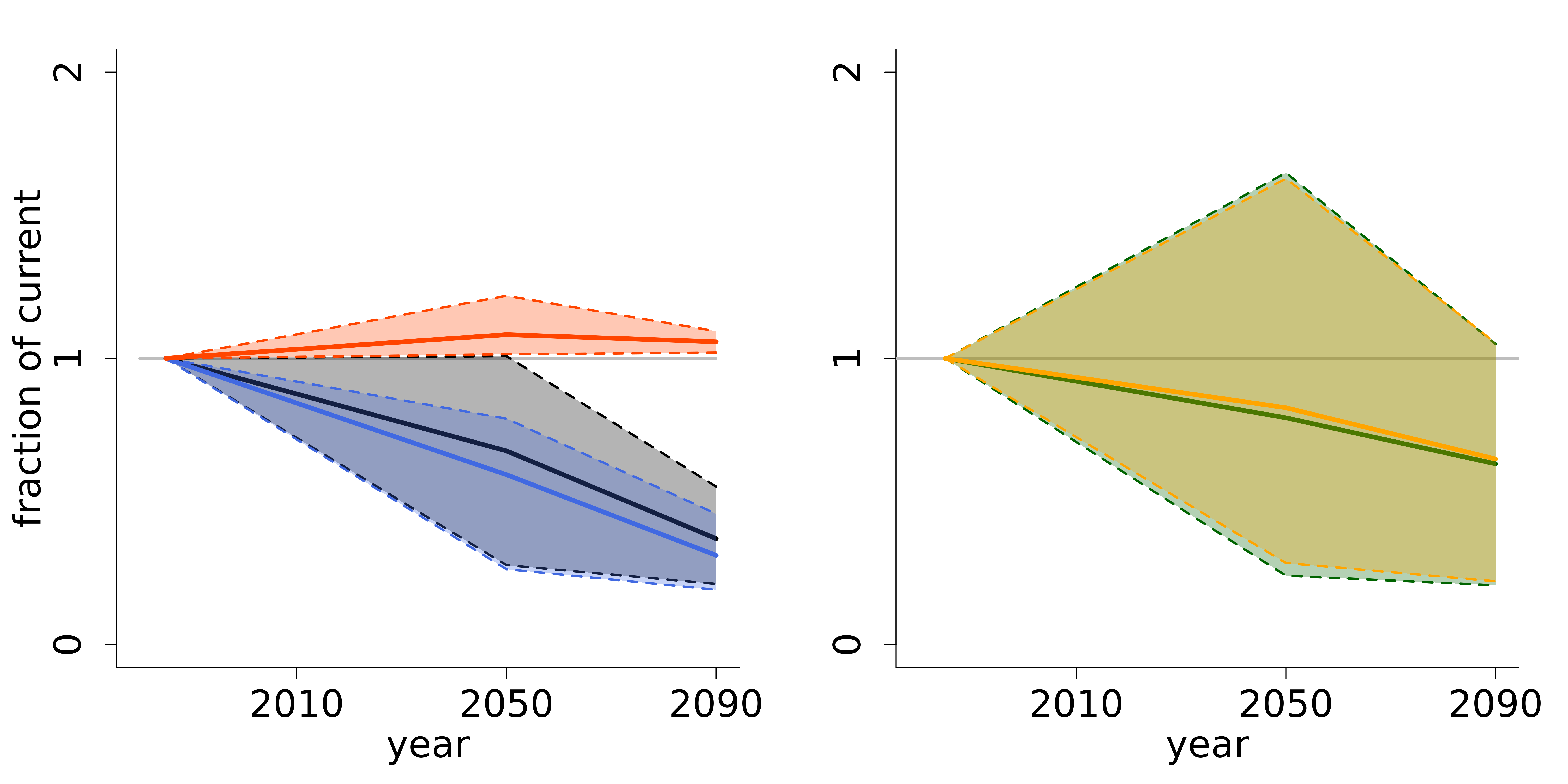

Supplement: S3 Appendix — (ZIP) [file pntd.0014030.s007.zip › Sup. Mat. 6-2 M-Z - Species Trends/Micrurus_corallinus_CCTrends.png]

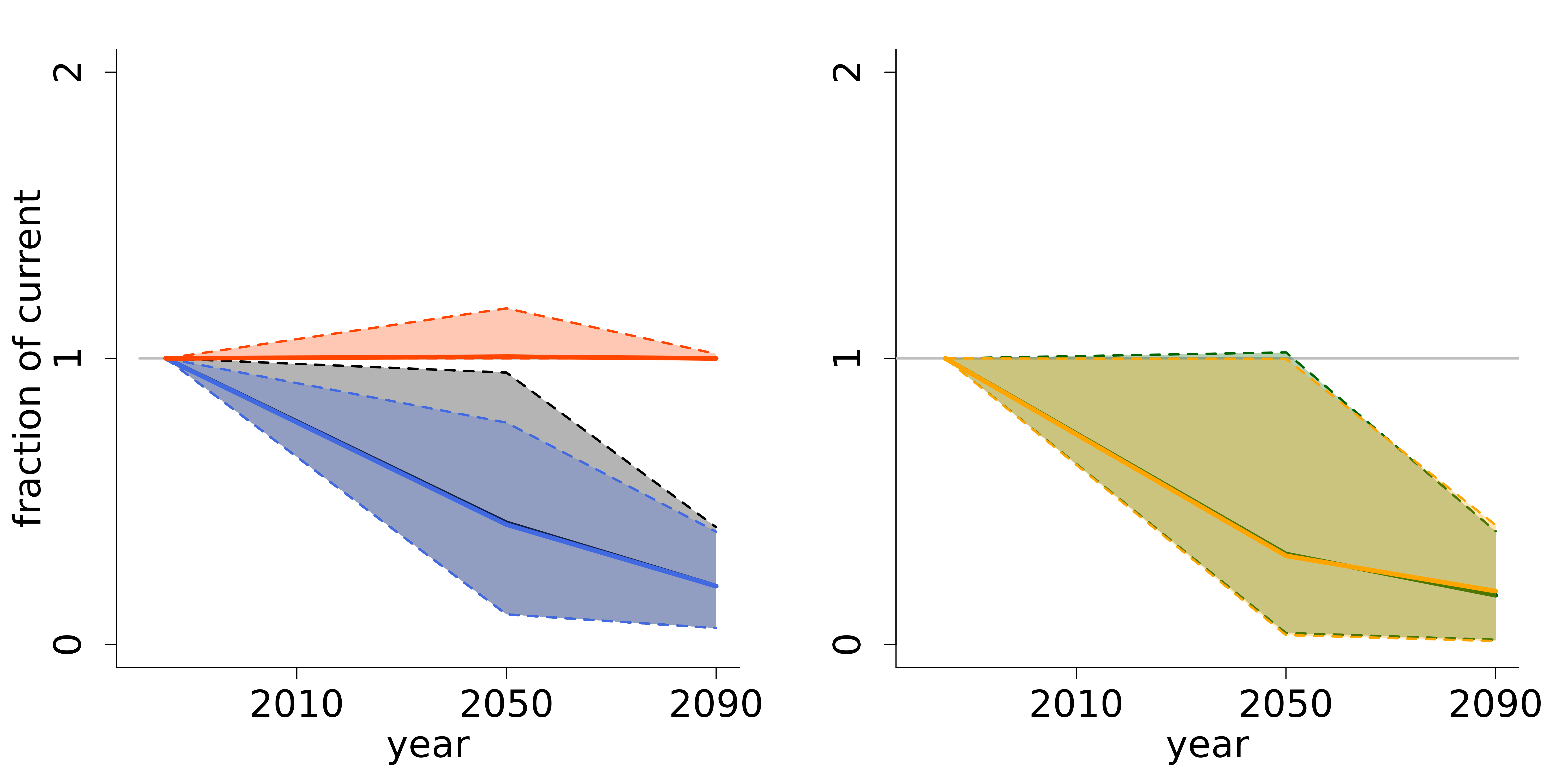

Supplement: S3 Appendix — (ZIP) [file pntd.0014030.s007.zip › Sup. Mat. 6-2 M-Z - Species Trends/Micrurus_decoratus_CCTrends.png]

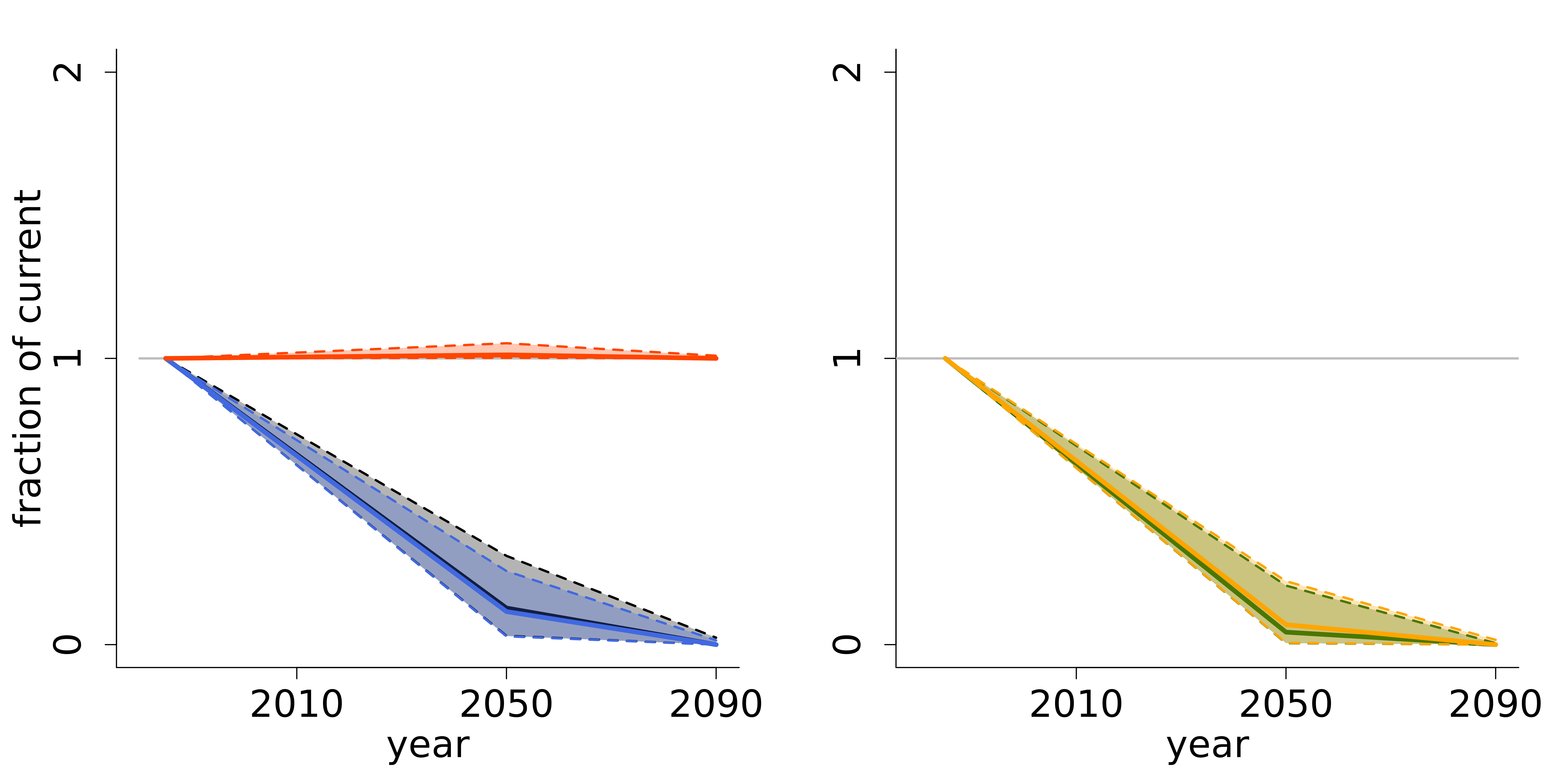

Supplement: S3 Appendix — (ZIP) [file pntd.0014030.s007.zip › Sup. Mat. 6-2 M-Z - Species Trends/Micrurus_diana_CCTrends.png]

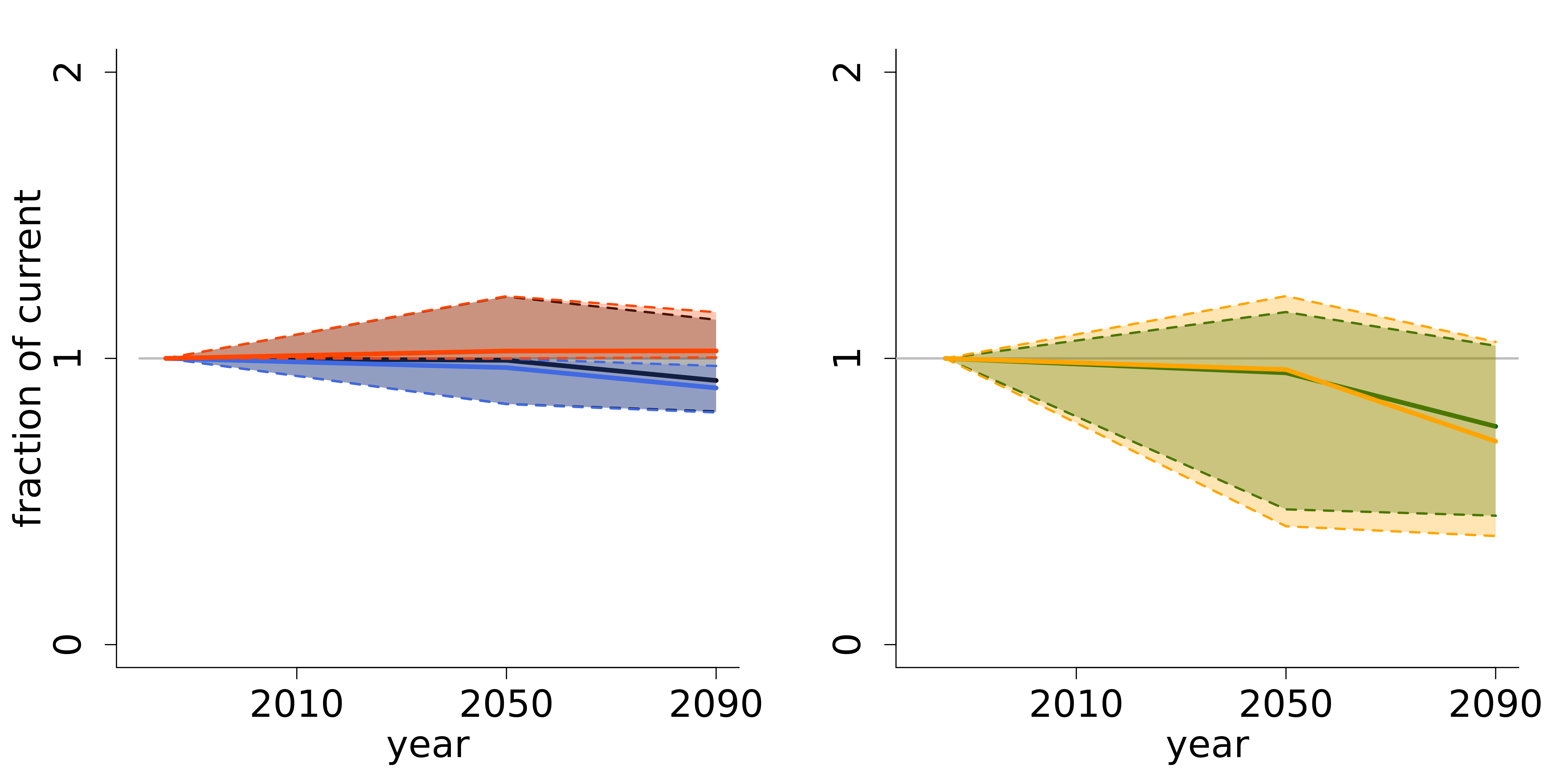

Supplement: S3 Appendix — (ZIP) [file pntd.0014030.s007.zip › Sup. Mat. 6-2 M-Z - Species Trends/Micrurus_diastema_CCTrends.png]

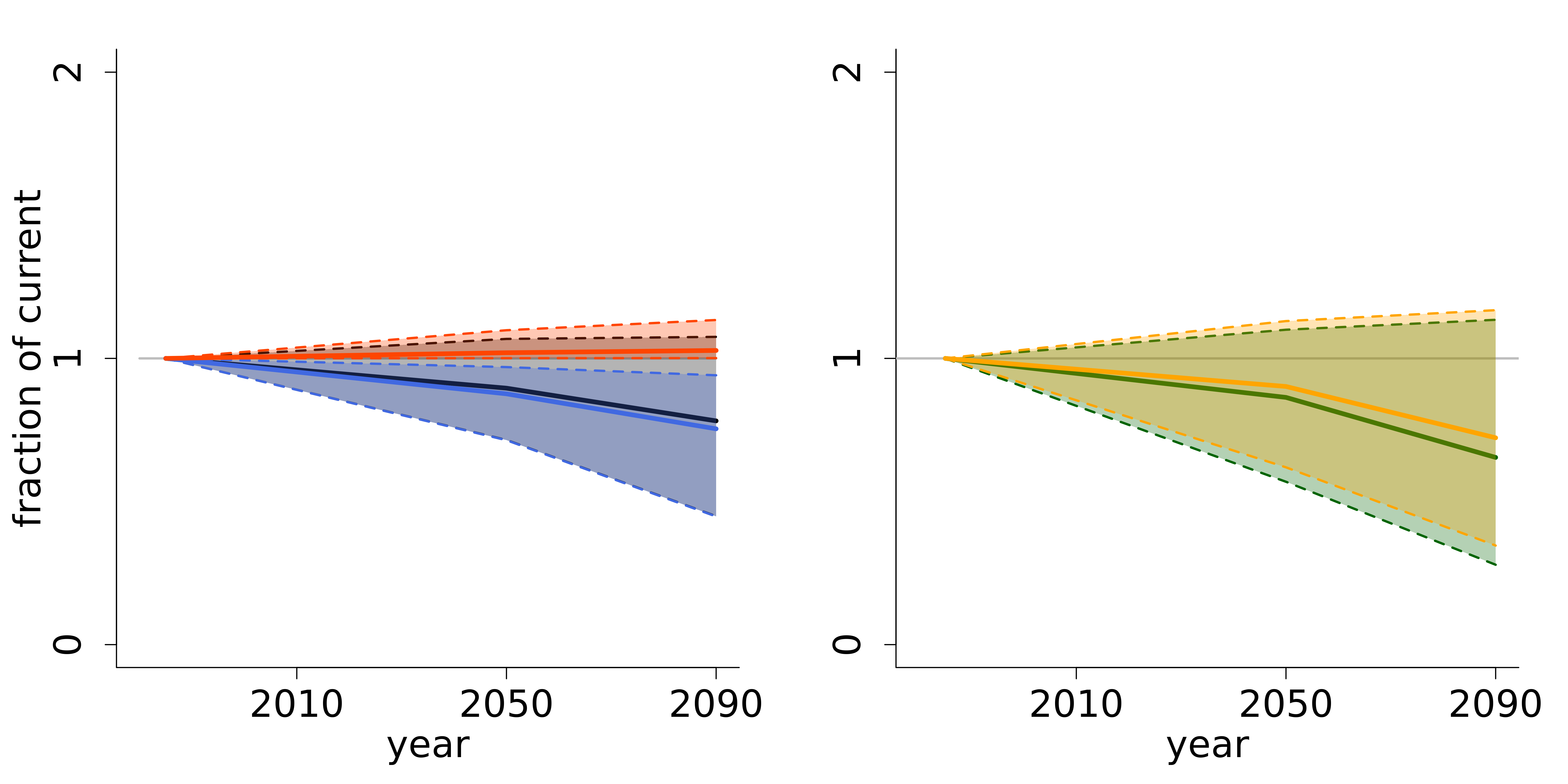

Supplement: S3 Appendix — (ZIP) [file pntd.0014030.s007.zip › Sup. Mat. 6-2 M-Z - Species Trends/Micrurus_dissoleucus_CCTrends.png]

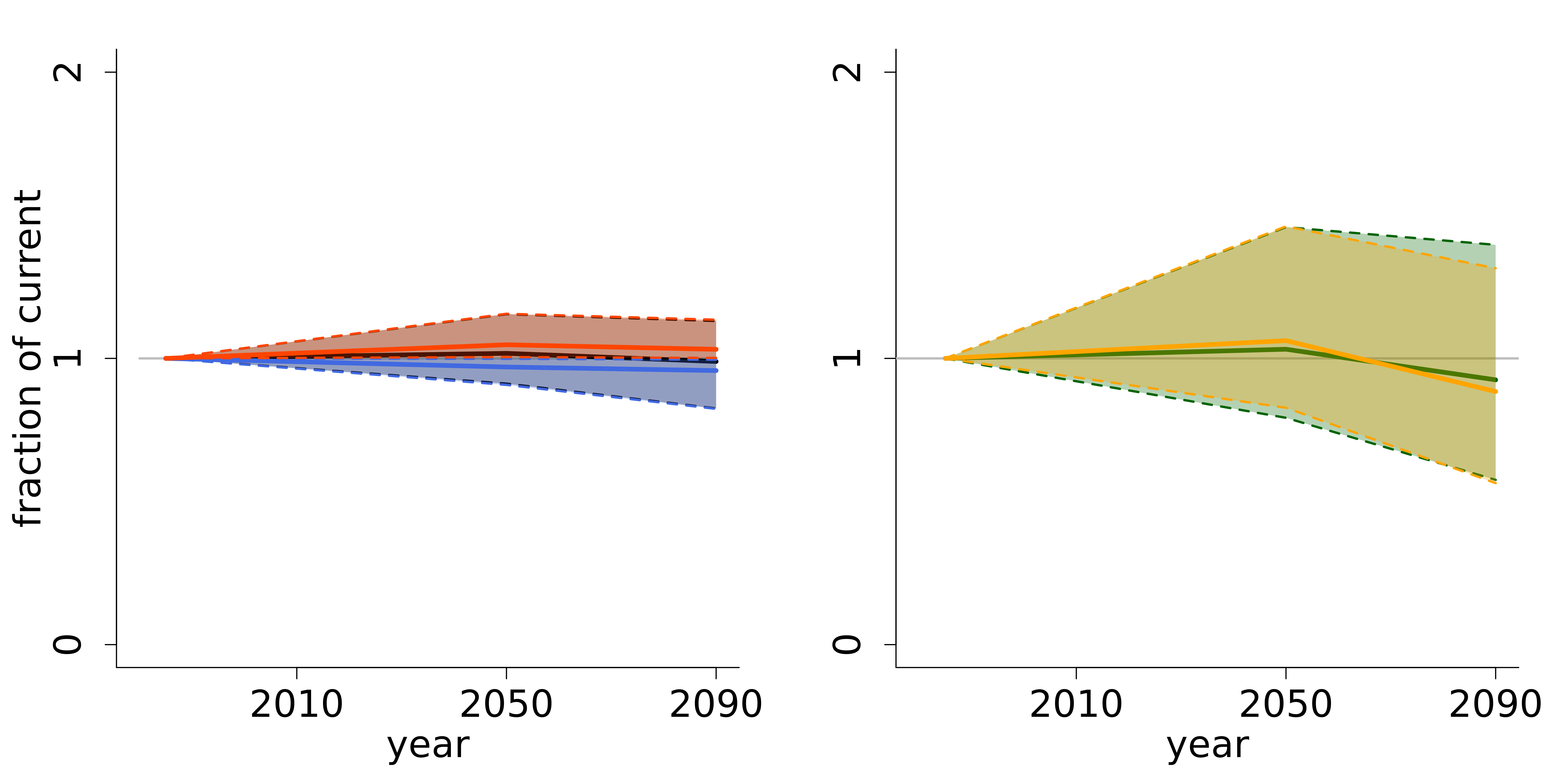

Supplement: S3 Appendix — (ZIP) [file pntd.0014030.s007.zip › Sup. Mat. 6-2 M-Z - Species Trends/Micrurus_distans_CCTrends.png]

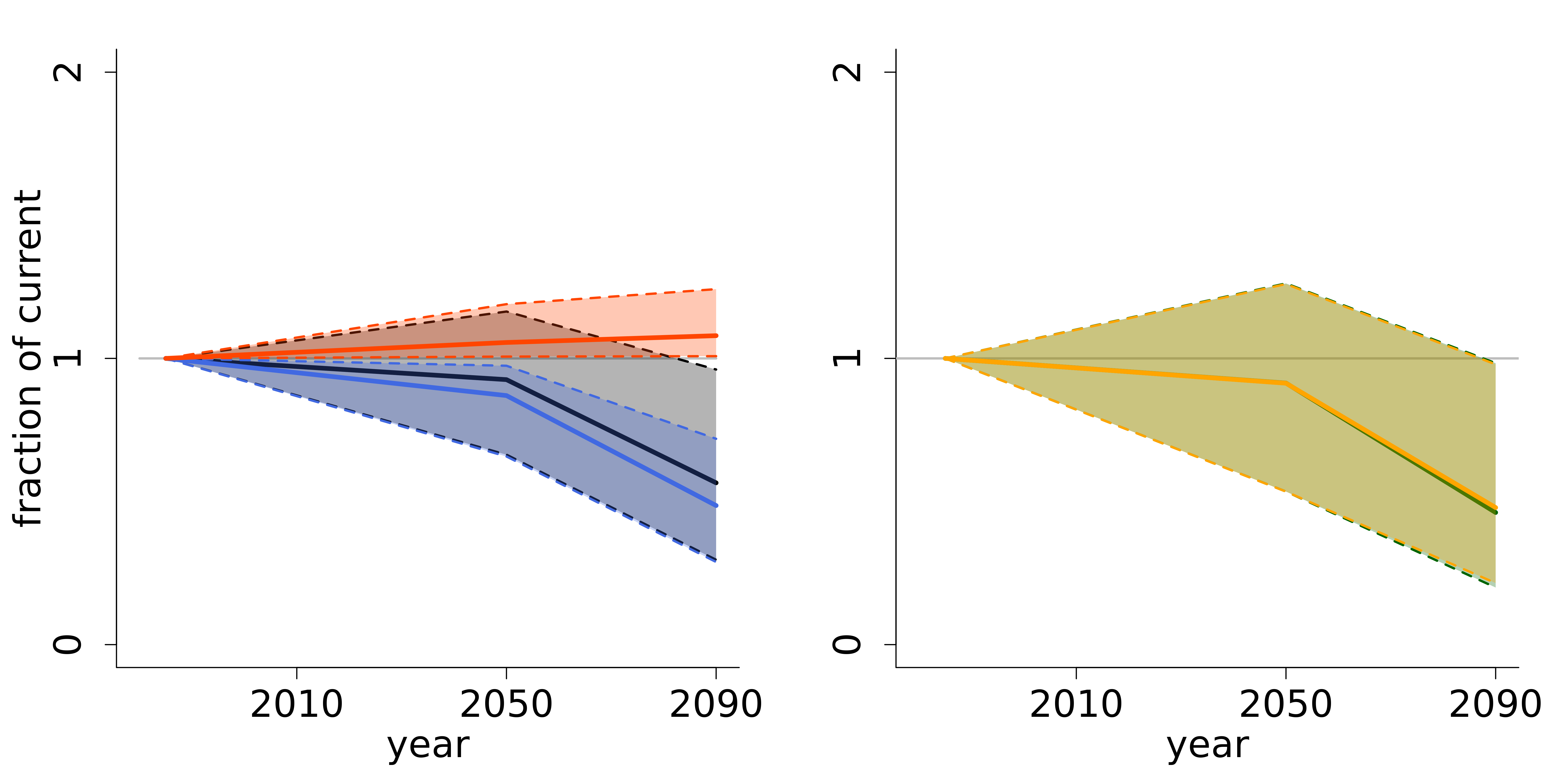

Supplement: S3 Appendix — (ZIP) [file pntd.0014030.s007.zip › Sup. Mat. 6-2 M-Z - Species Trends/Micrurus_dumerilii_CCTrends.png]

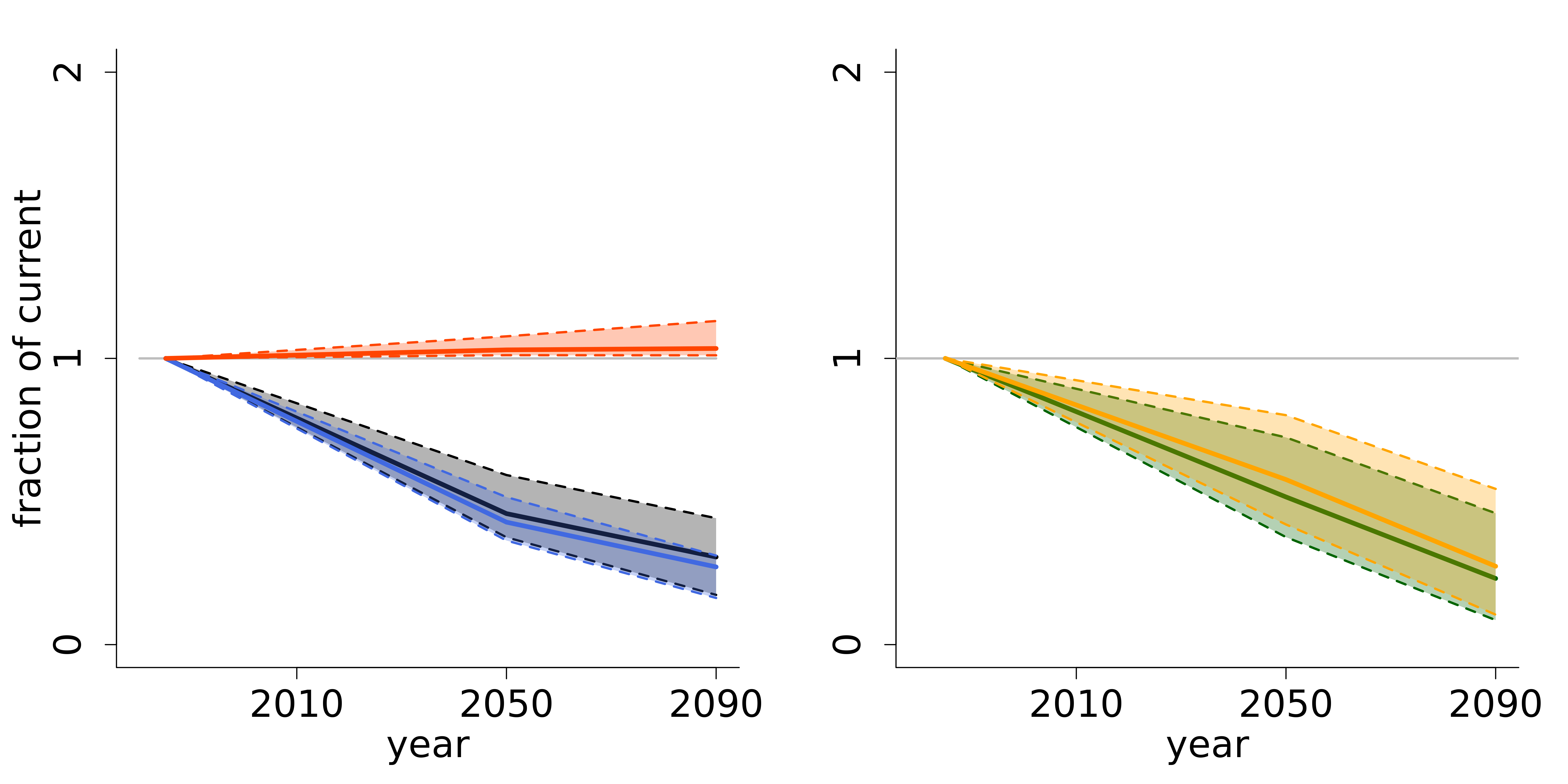

Supplement: S3 Appendix — (ZIP) [file pntd.0014030.s007.zip › Sup. Mat. 6-2 M-Z - Species Trends/Micrurus_elegans_CCTrends.png]

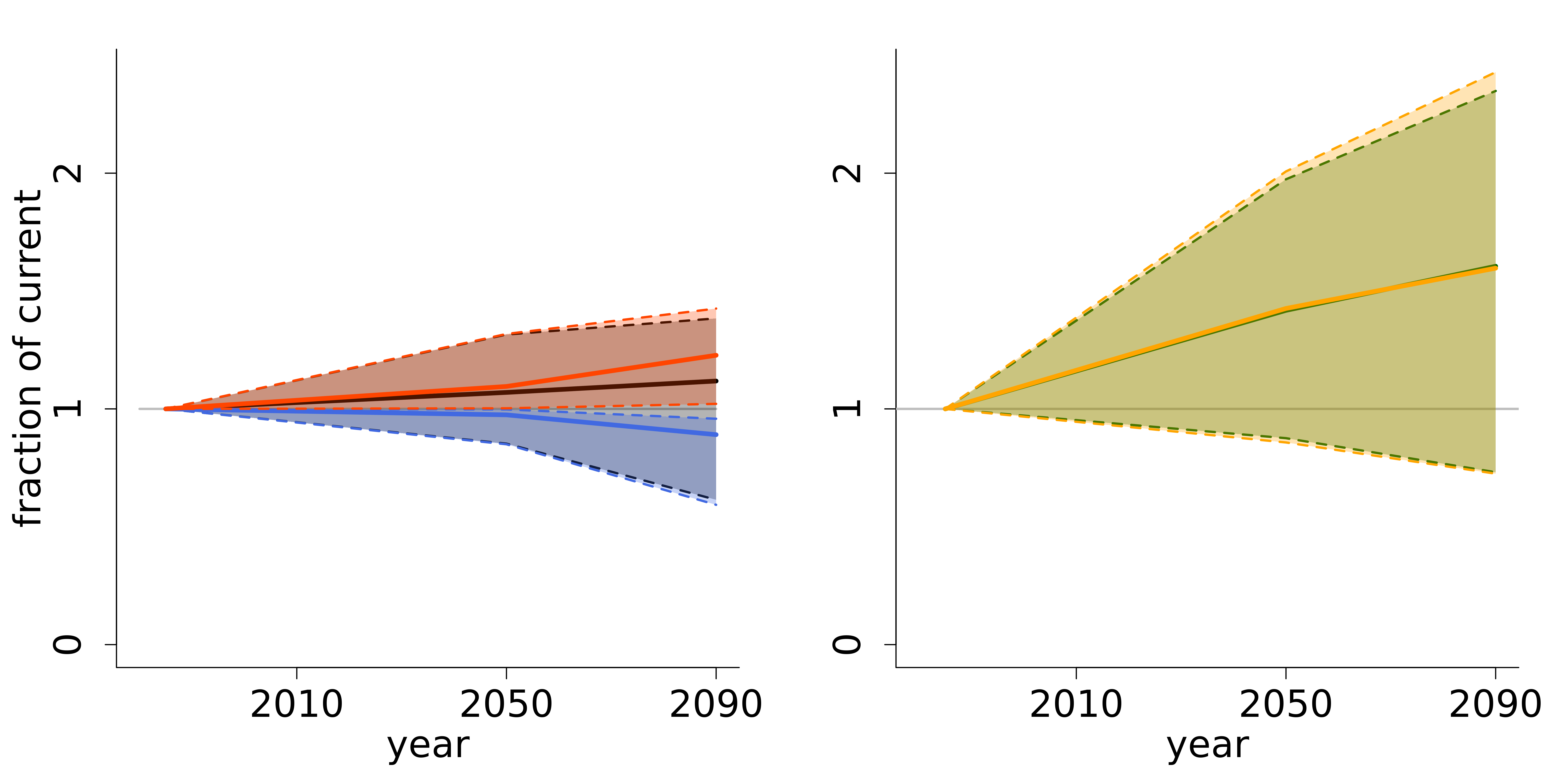

Supplement: S3 Appendix — (ZIP) [file pntd.0014030.s007.zip › Sup. Mat. 6-2 M-Z - Species Trends/Micrurus_ephippifer_CCTrends.png]

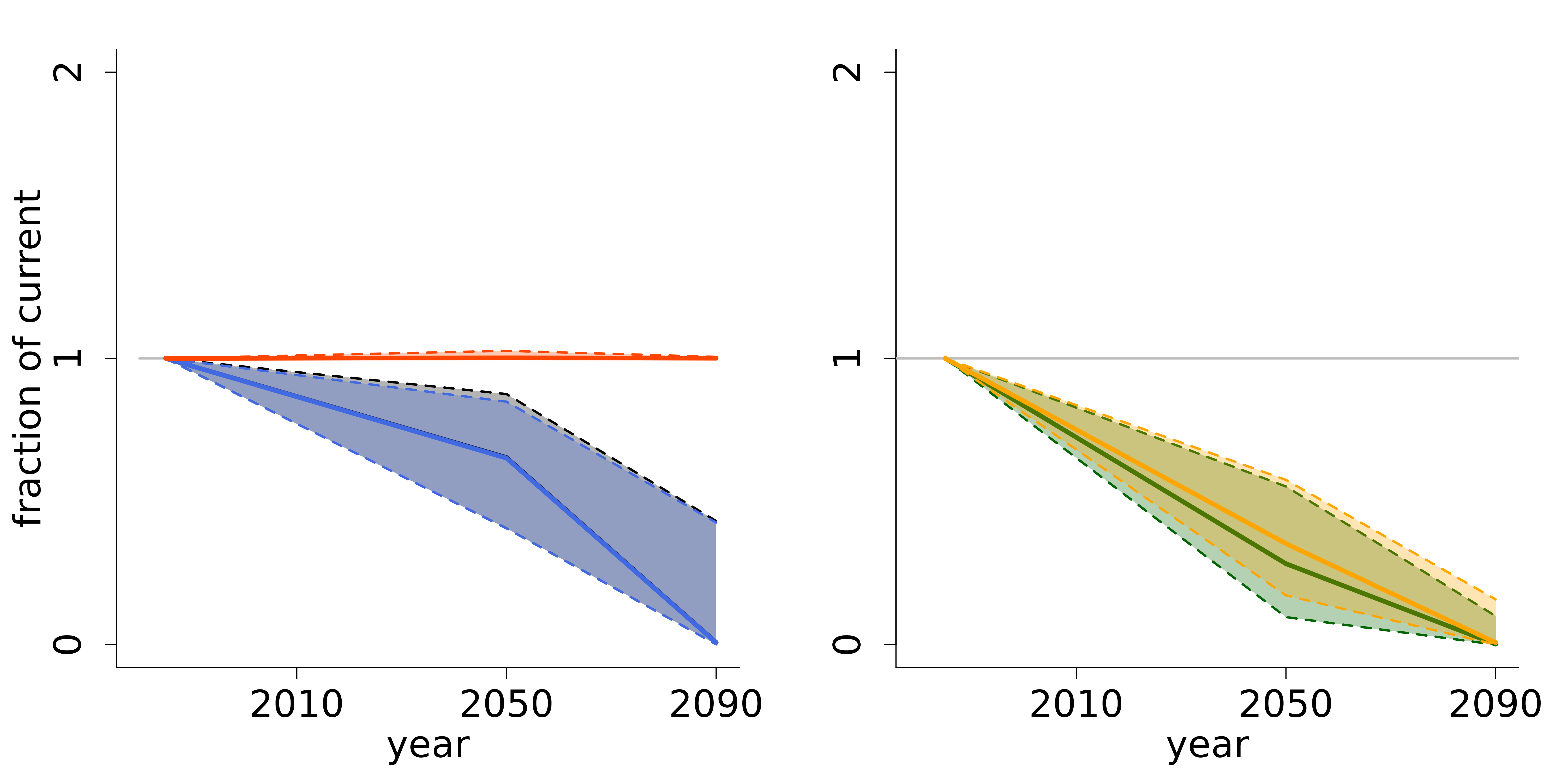

Supplement: S3 Appendix — (ZIP) [file pntd.0014030.s007.zip › Sup. Mat. 6-2 M-Z - Species Trends/Micrurus_filiformis_CCTrends.png]

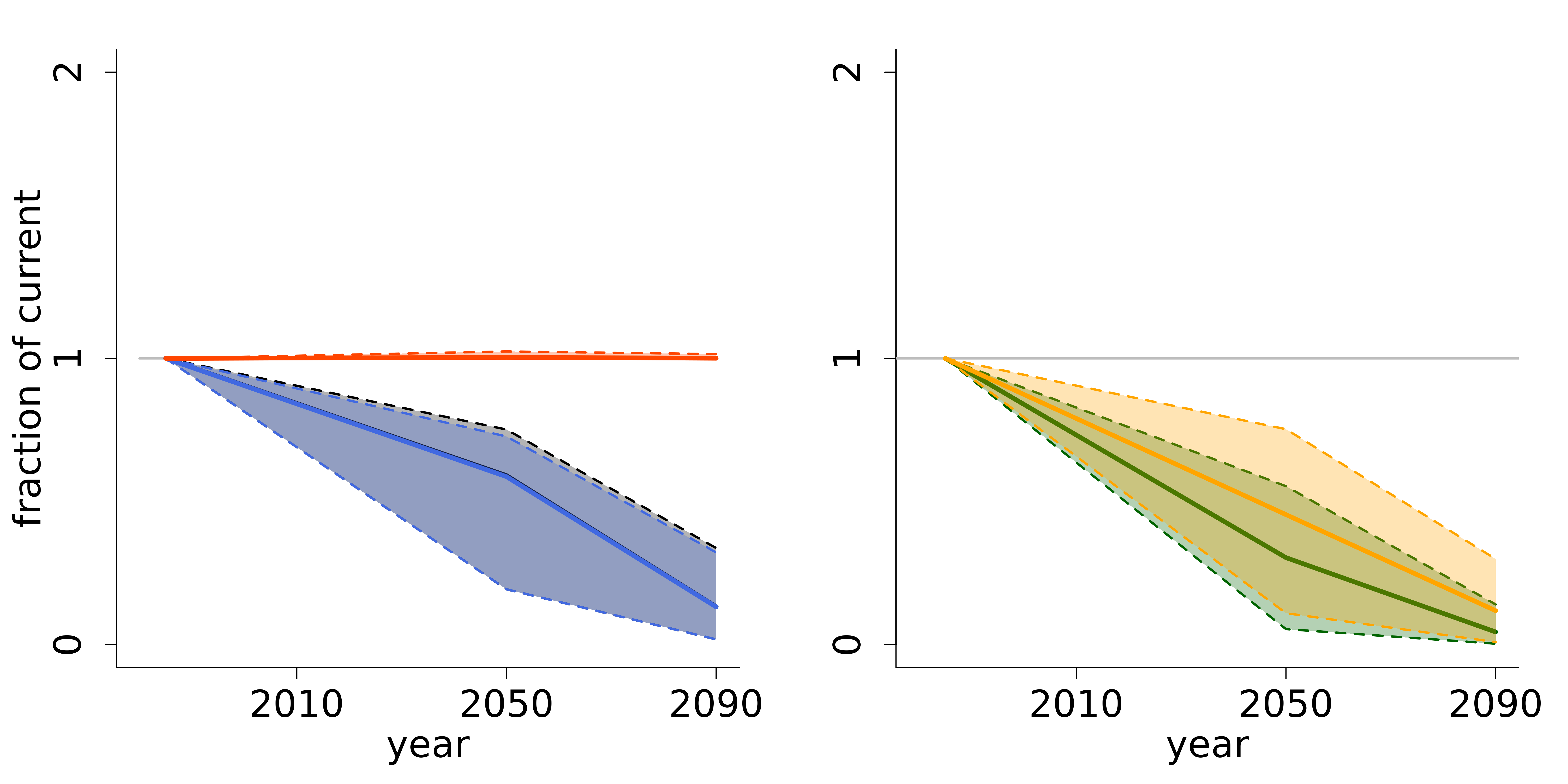

Supplement: S3 Appendix — (ZIP) [file pntd.0014030.s007.zip › Sup. Mat. 6-2 M-Z - Species Trends/Micrurus_frontalis_CCTrends.png]

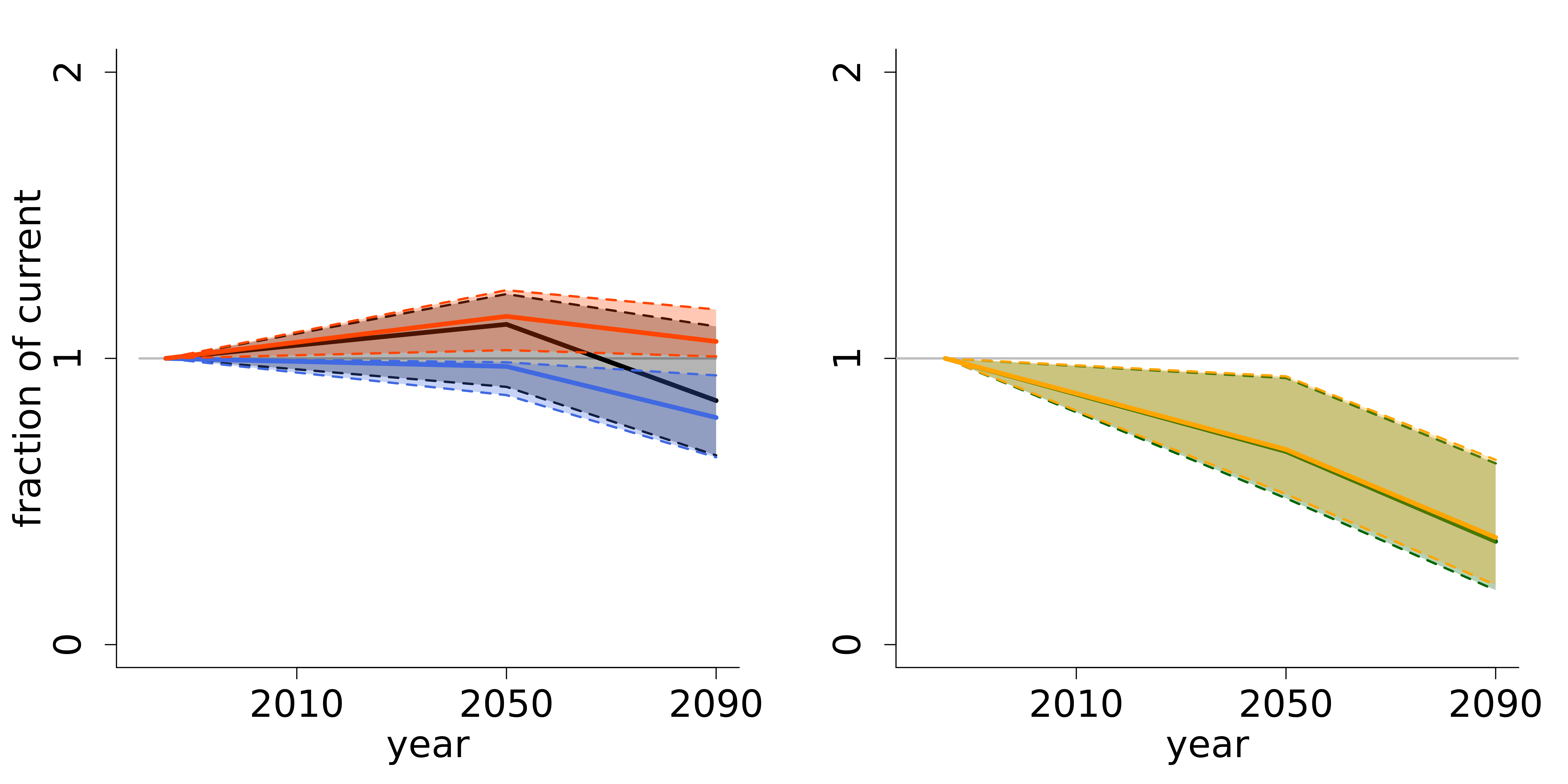

Supplement: S3 Appendix — (ZIP) [file pntd.0014030.s007.zip › Sup. Mat. 6-2 M-Z - Species Trends/Micrurus_fulvius_CCTrends.png]

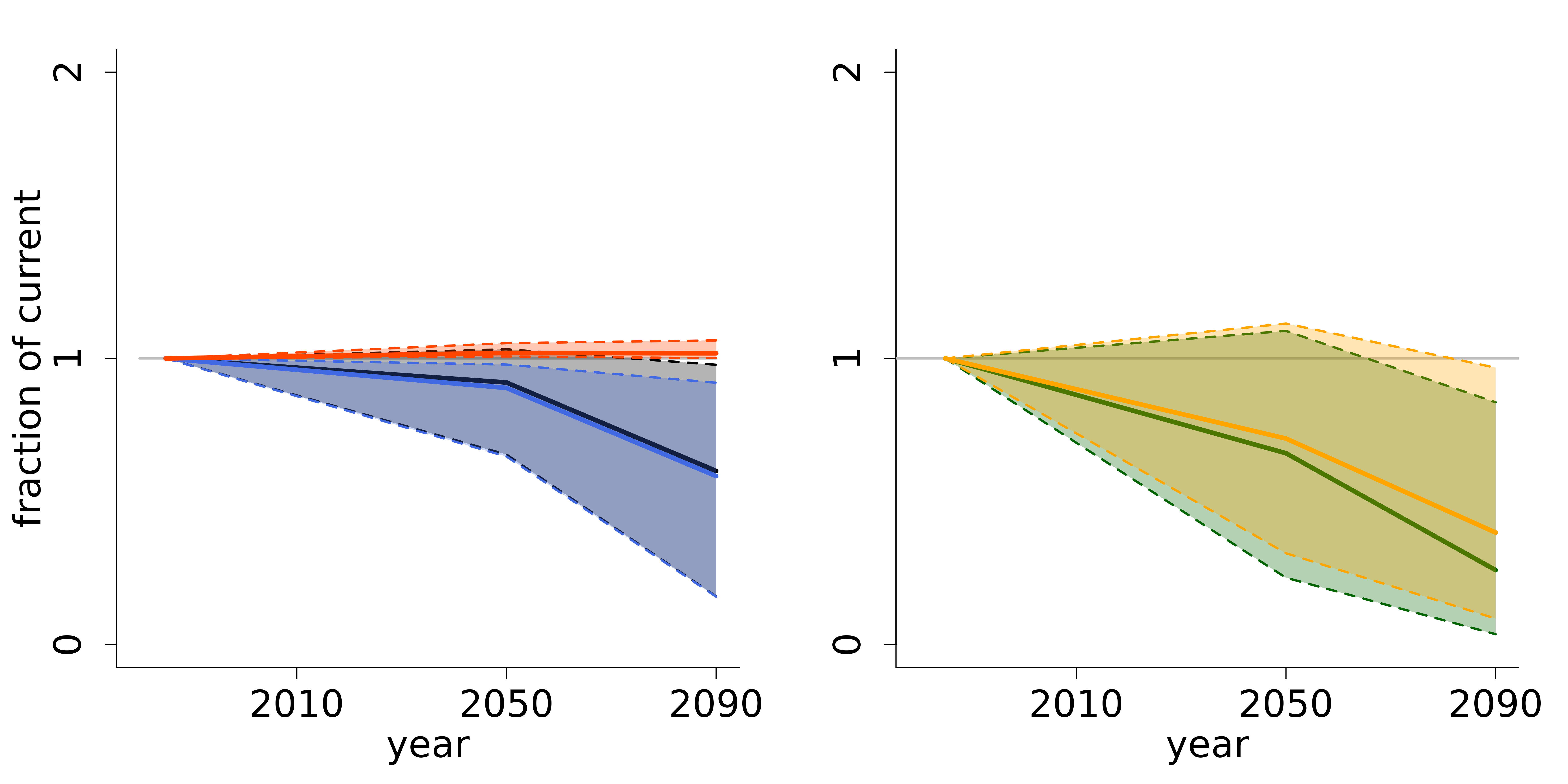

Supplement: S3 Appendix — (ZIP) [file pntd.0014030.s007.zip › Sup. Mat. 6-2 M-Z - Species Trends/Micrurus_hemprichii_CCTrends.png]

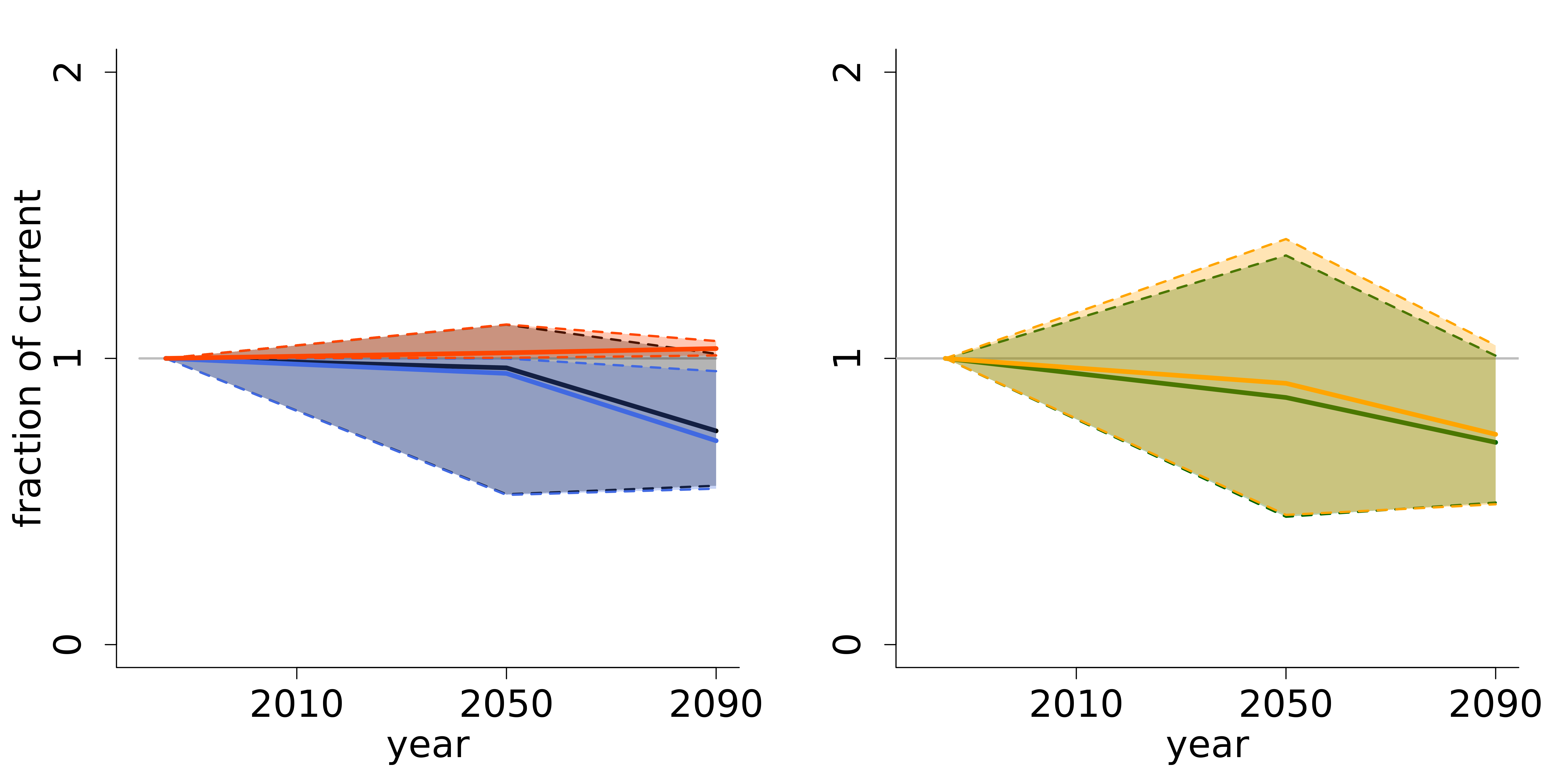

Supplement: S3 Appendix — (ZIP) [file pntd.0014030.s007.zip › Sup. Mat. 6-2 M-Z - Species Trends/Micrurus_hippocrepis_CCTrends.png]

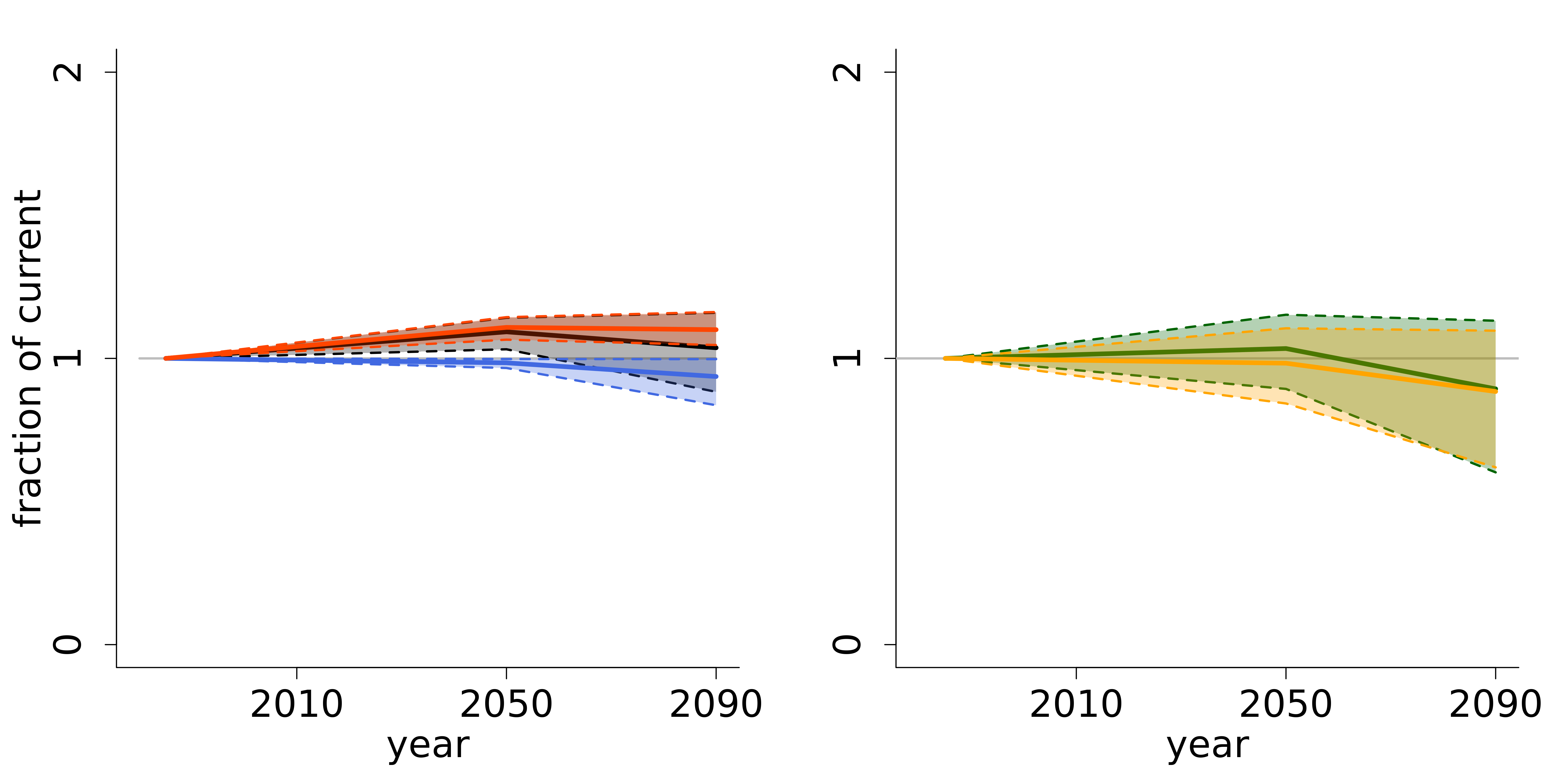

Supplement: S3 Appendix — (ZIP) [file pntd.0014030.s007.zip › Sup. Mat. 6-2 M-Z - Species Trends/Micrurus_ibiboboca_CCTrends.png]

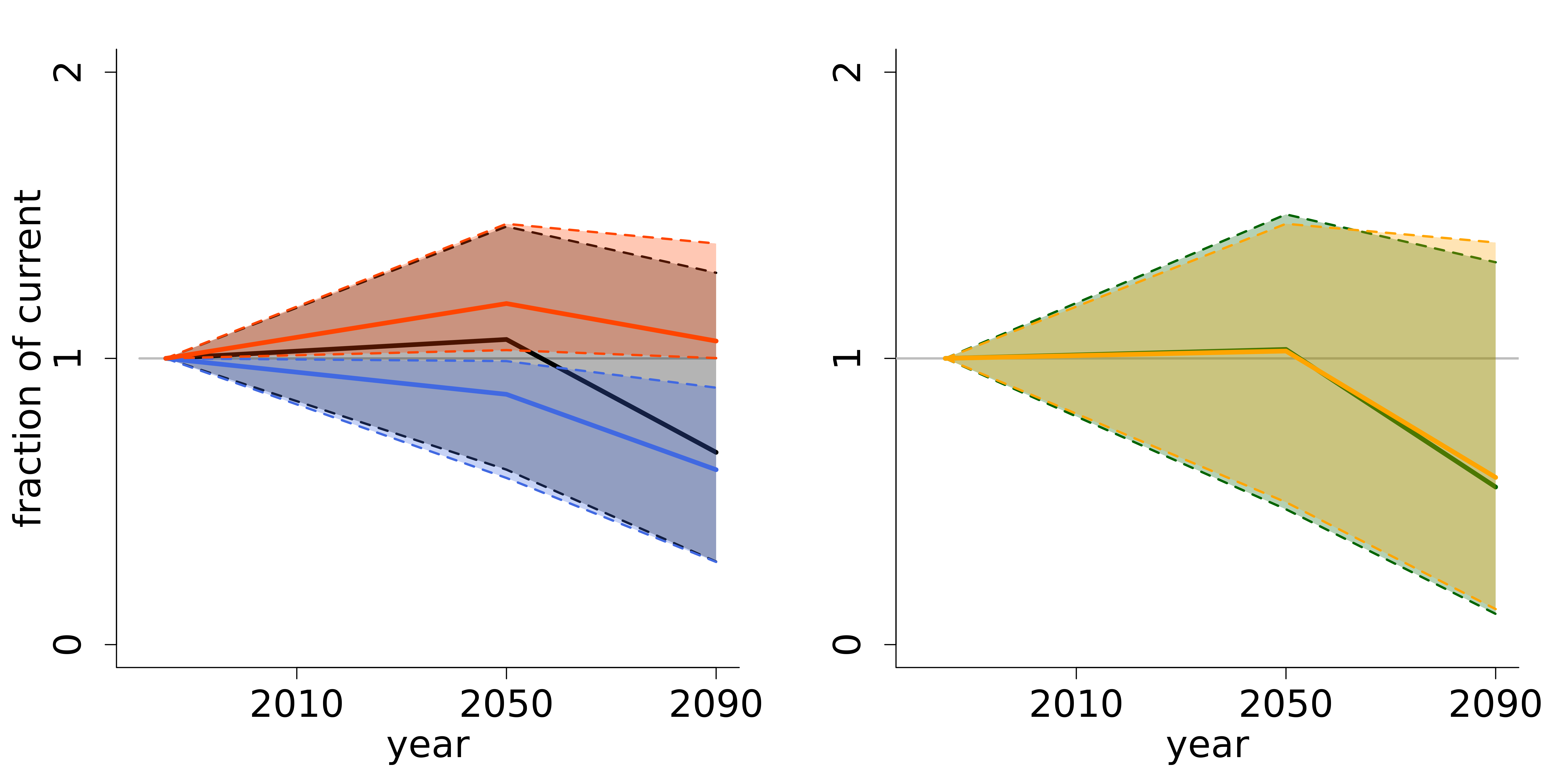

Supplement: S3 Appendix — (ZIP) [file pntd.0014030.s007.zip › Sup. Mat. 6-2 M-Z - Species Trends/Micrurus_isozonus_CCTrends.png]

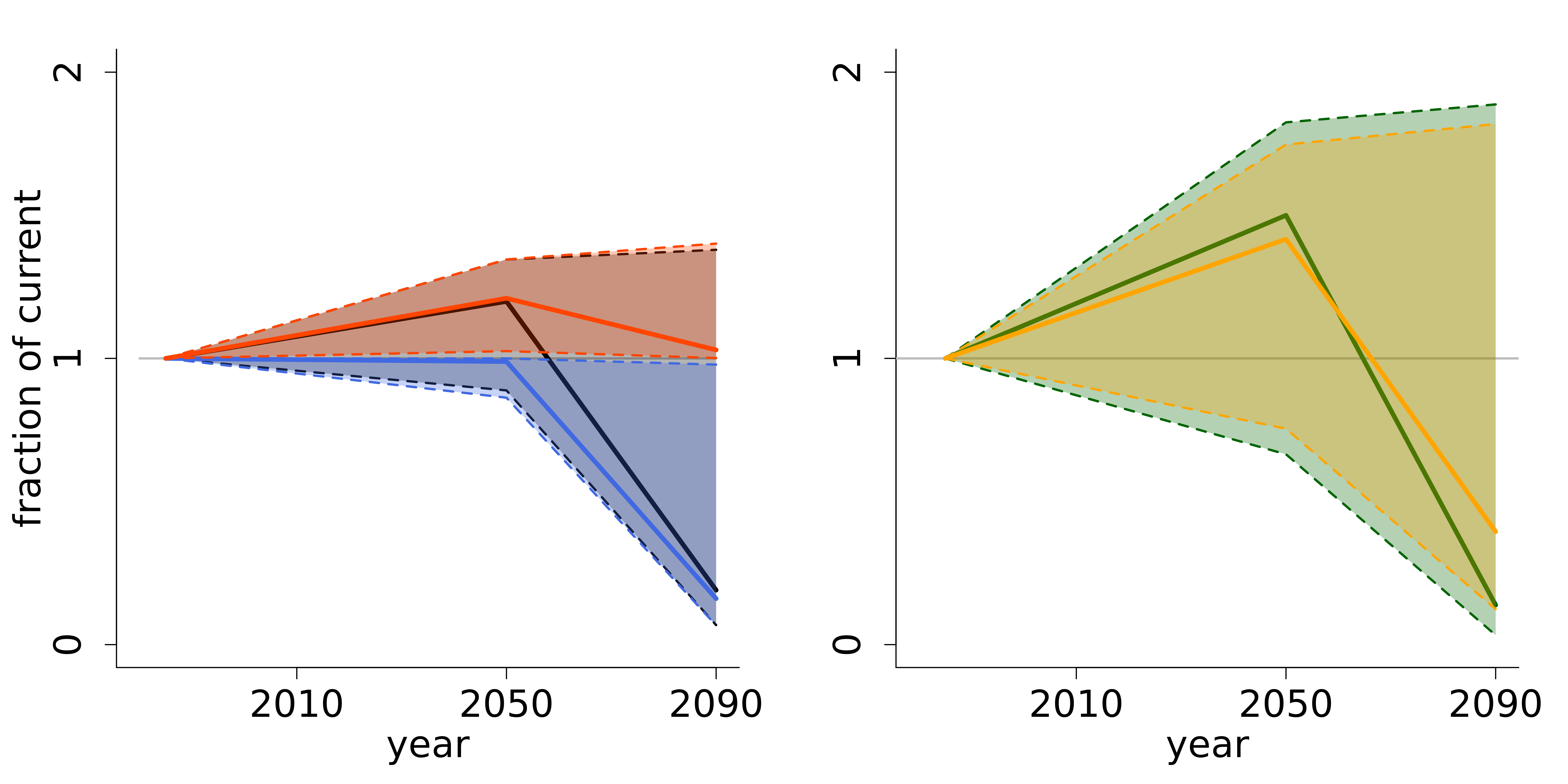

Supplement: S3 Appendix — (ZIP) [file pntd.0014030.s007.zip › Sup. Mat. 6-2 M-Z - Species Trends/Micrurus_langsdorffi_CCTrends.png]

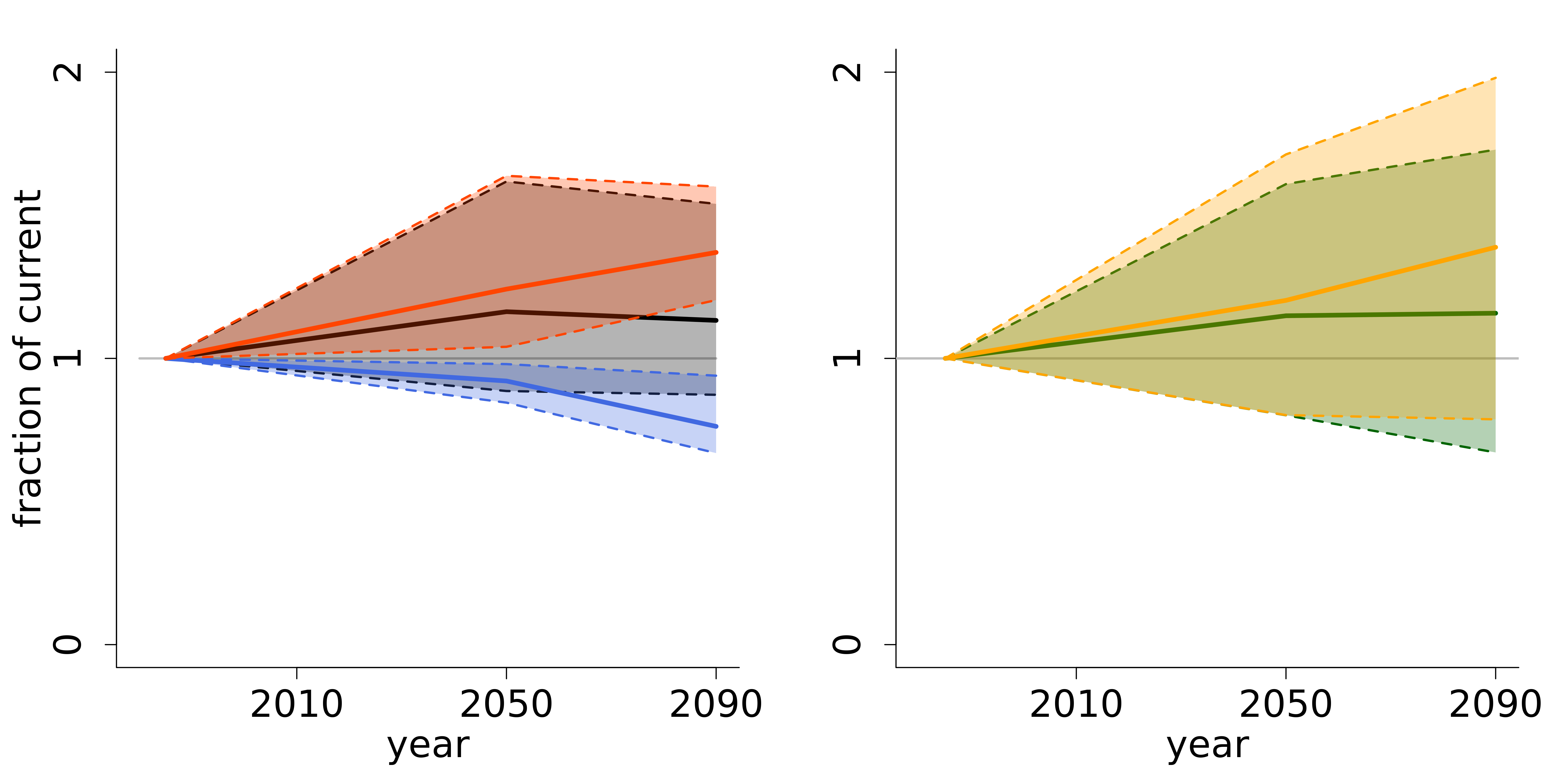

Supplement: S3 Appendix — (ZIP) [file pntd.0014030.s007.zip › Sup. Mat. 6-2 M-Z - Species Trends/Micrurus_laticollaris_CCTrends.png]

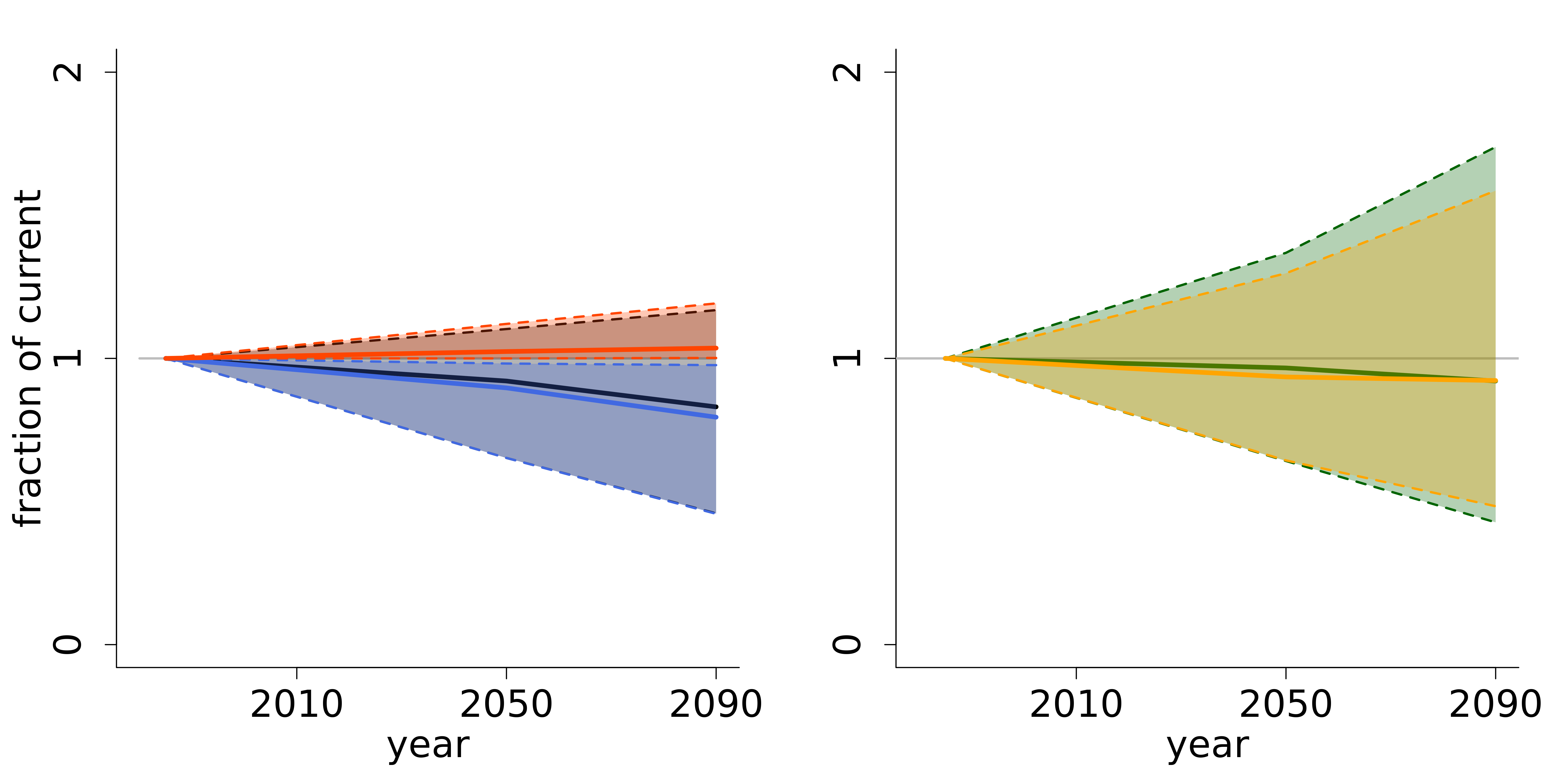

Supplement: S3 Appendix — (ZIP) [file pntd.0014030.s007.zip › Sup. Mat. 6-2 M-Z - Species Trends/Micrurus_latifasciatus_CCTrends.png]

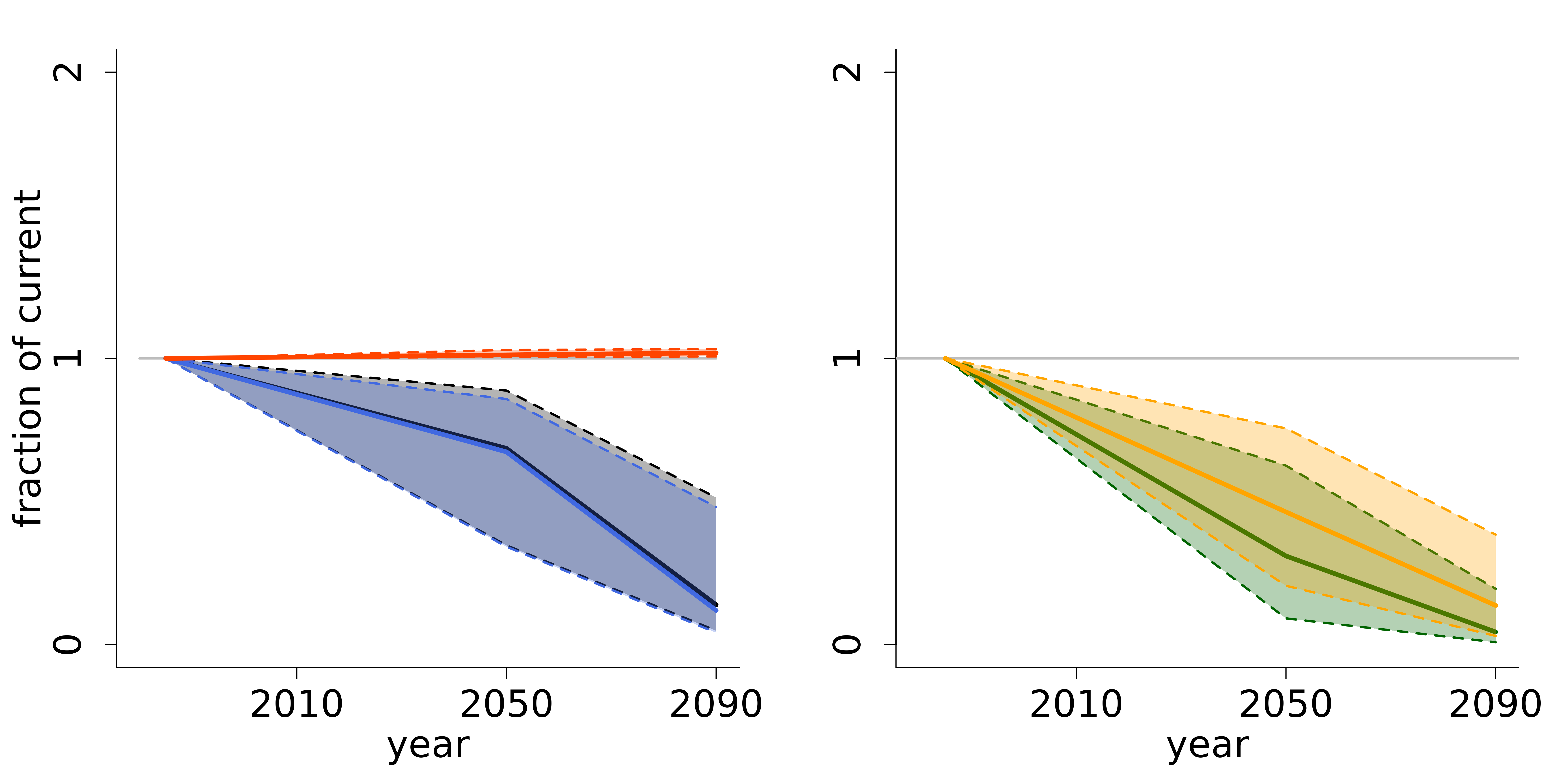

Supplement: S3 Appendix — (ZIP) [file pntd.0014030.s007.zip › Sup. Mat. 6-2 M-Z - Species Trends/Micrurus_lemniscatus_CCTrends.png]

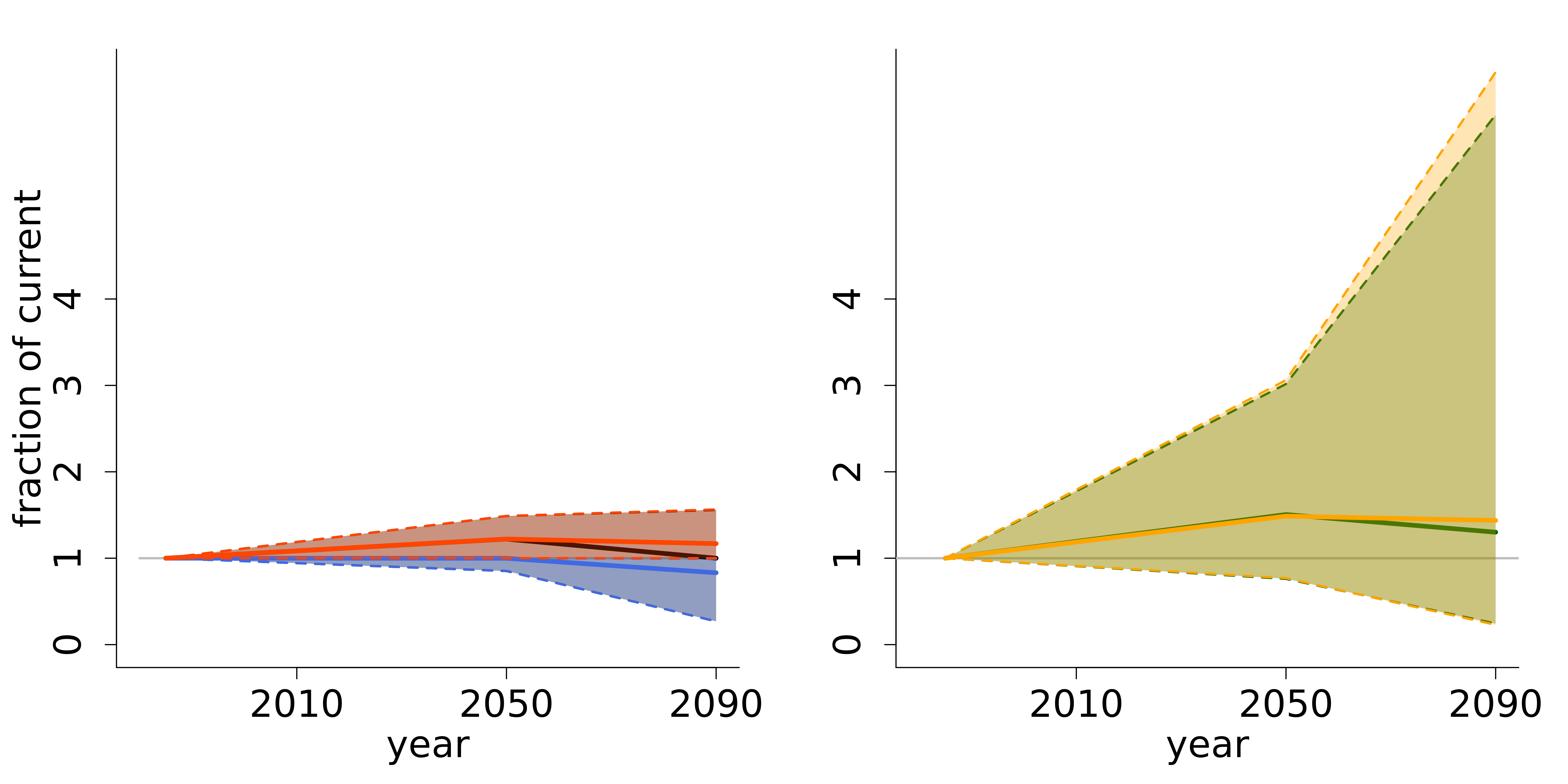

Supplement: S3 Appendix — (ZIP) [file pntd.0014030.s007.zip › Sup. Mat. 6-2 M-Z - Species Trends/Micrurus_limbatus_CCTrends.png]

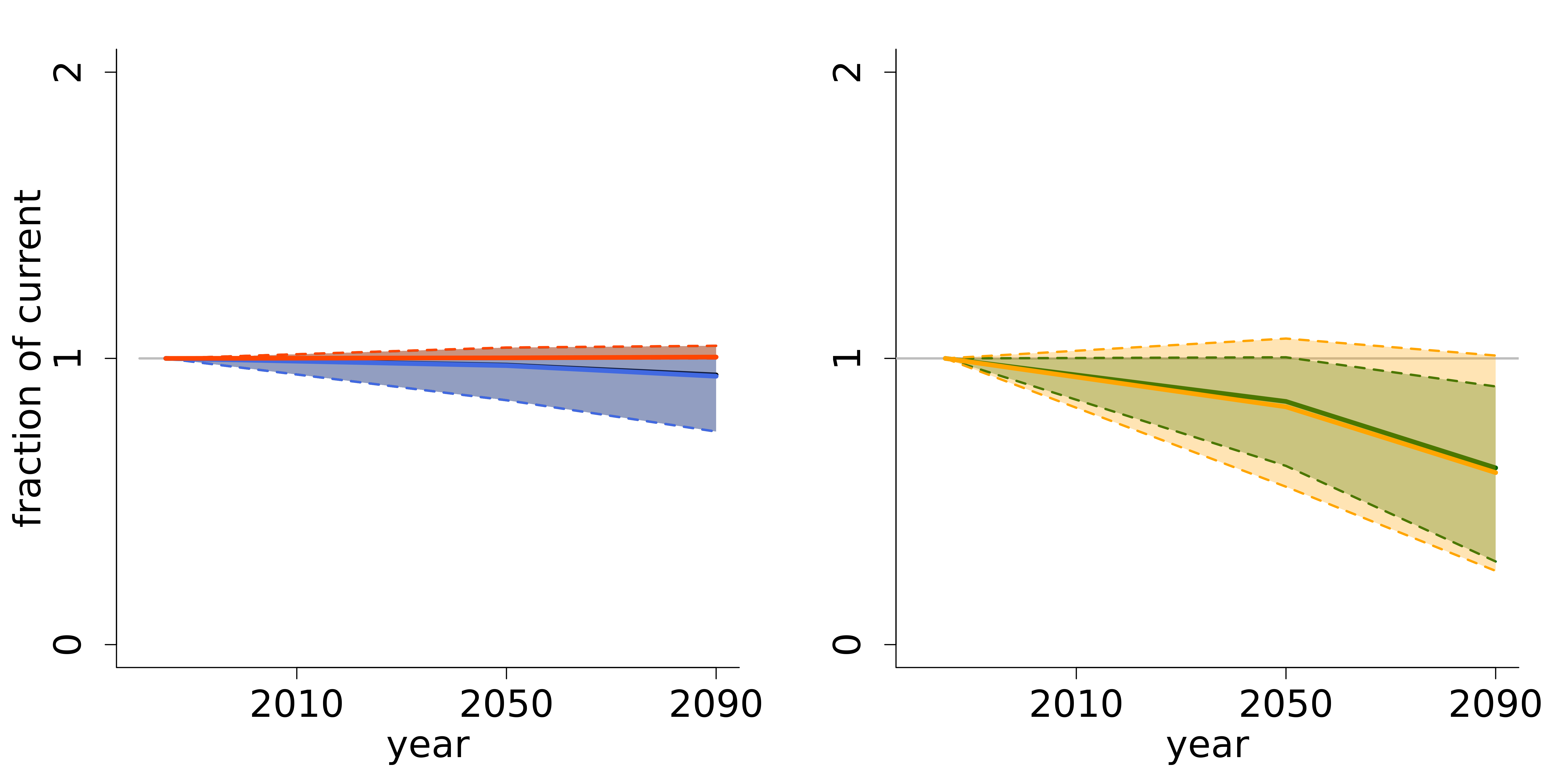

Supplement: S3 Appendix — (ZIP) [file pntd.0014030.s007.zip › Sup. Mat. 6-2 M-Z - Species Trends/Micrurus_margaritiferus_CCTrends.png]

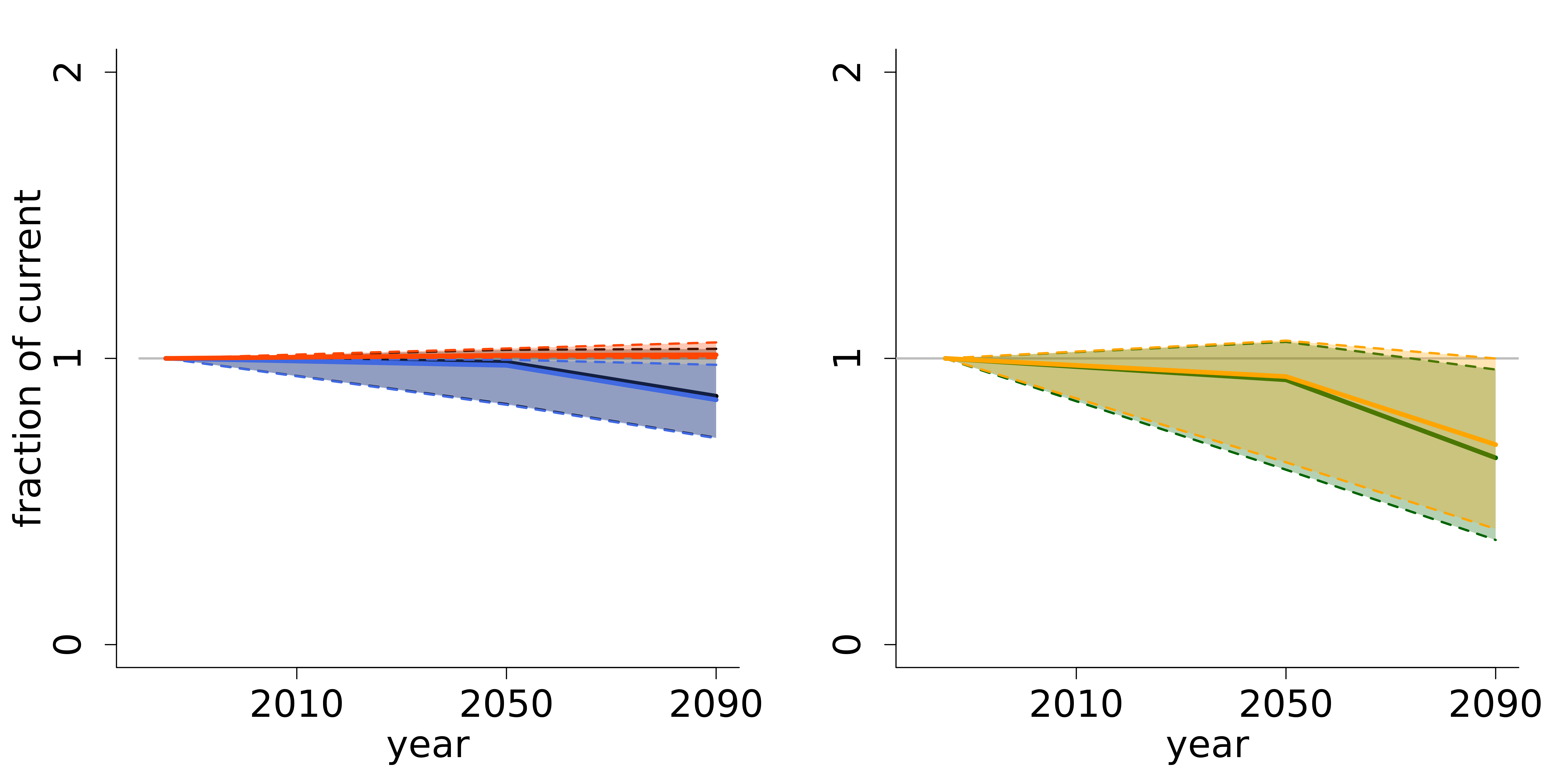

Supplement: S3 Appendix — (ZIP) [file pntd.0014030.s007.zip › Sup. Mat. 6-2 M-Z - Species Trends/Micrurus_medemi_CCTrends.png]

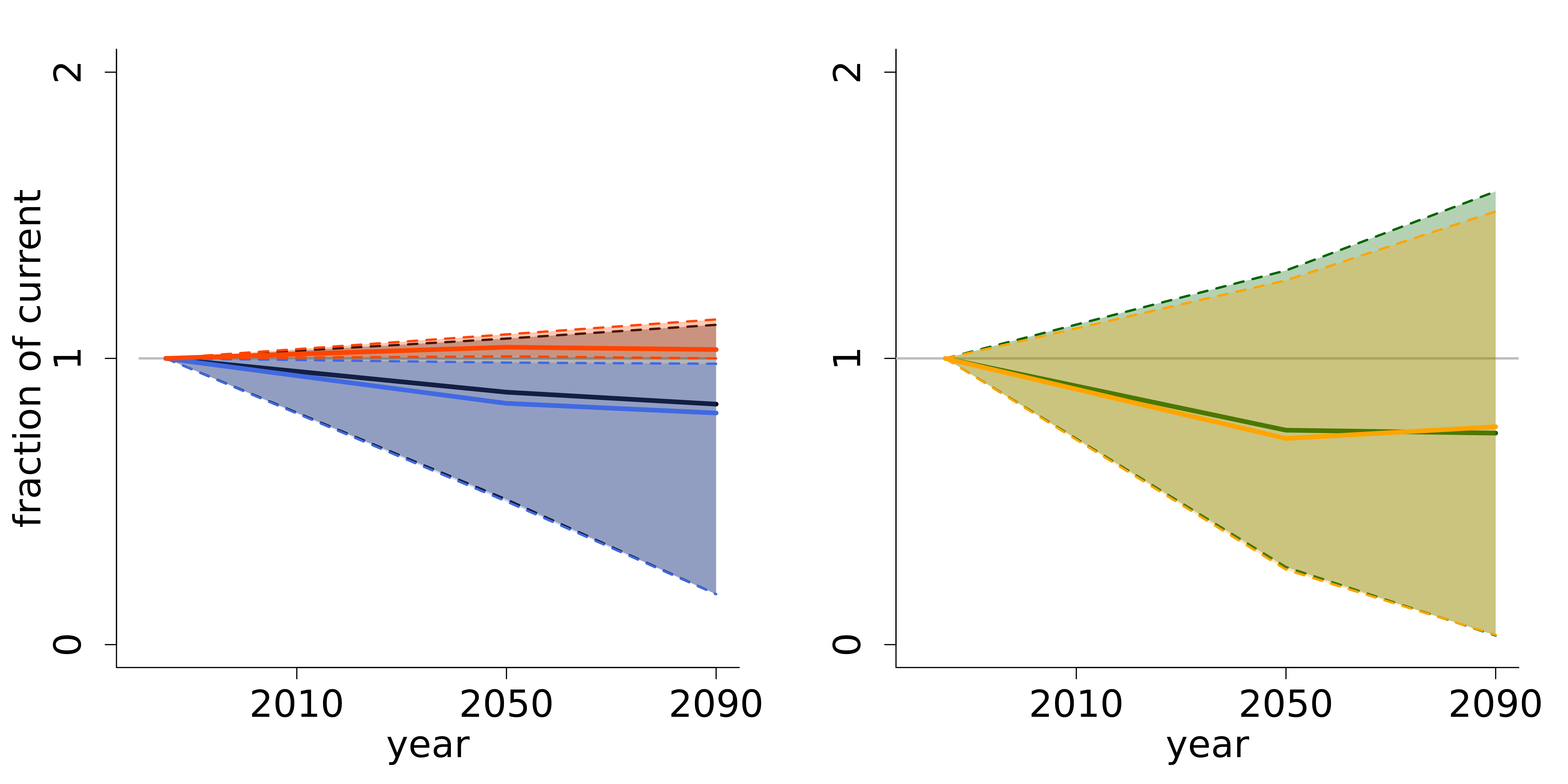

Supplement: S3 Appendix — (ZIP) [file pntd.0014030.s007.zip › Sup. Mat. 6-2 M-Z - Species Trends/Micrurus_meridensis_CCTrends.png]

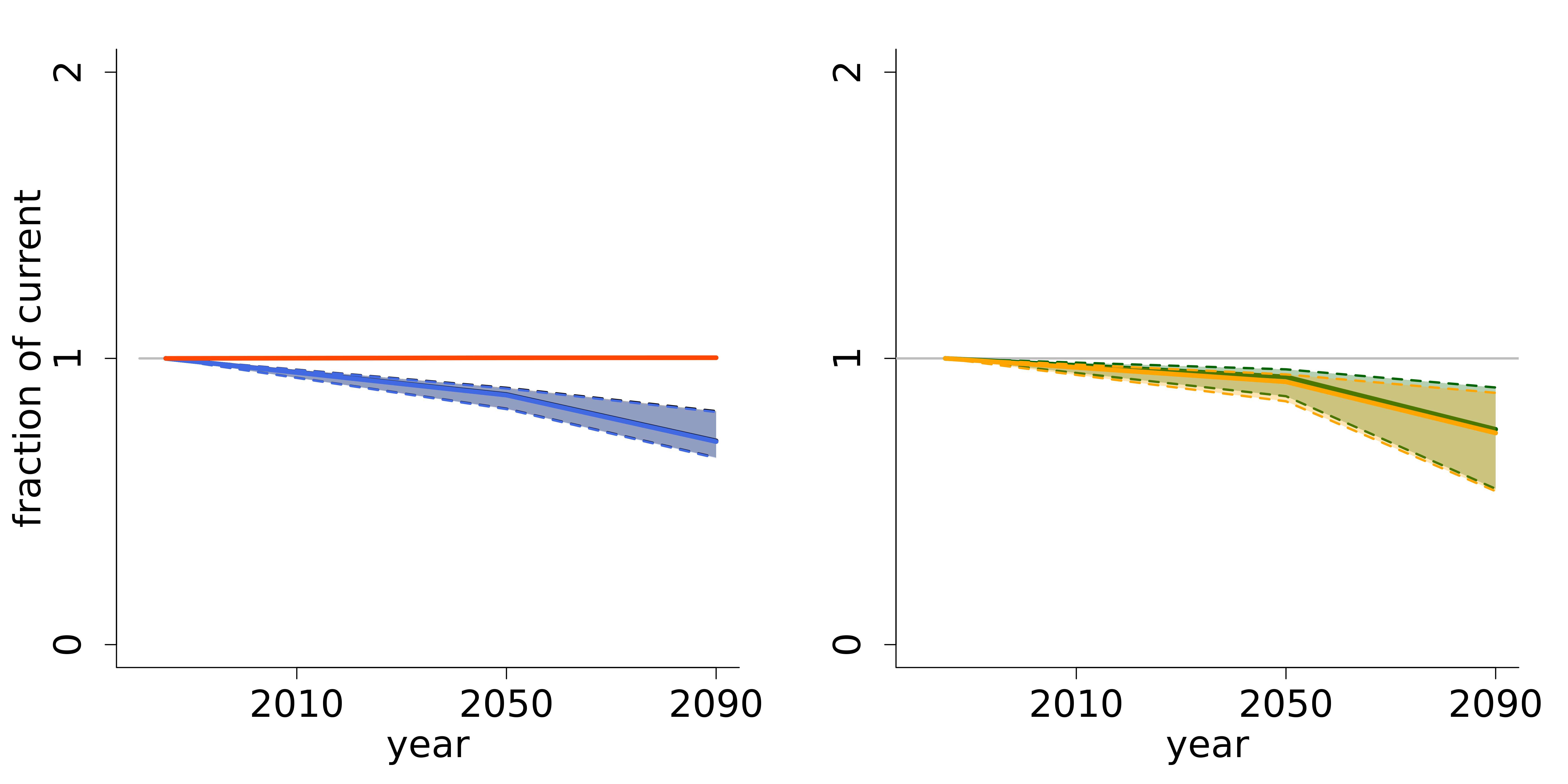

Supplement: S3 Appendix — (ZIP) [file pntd.0014030.s007.zip › Sup. Mat. 6-2 M-Z - Species Trends/Micrurus_mertensi_CCTrends.png]

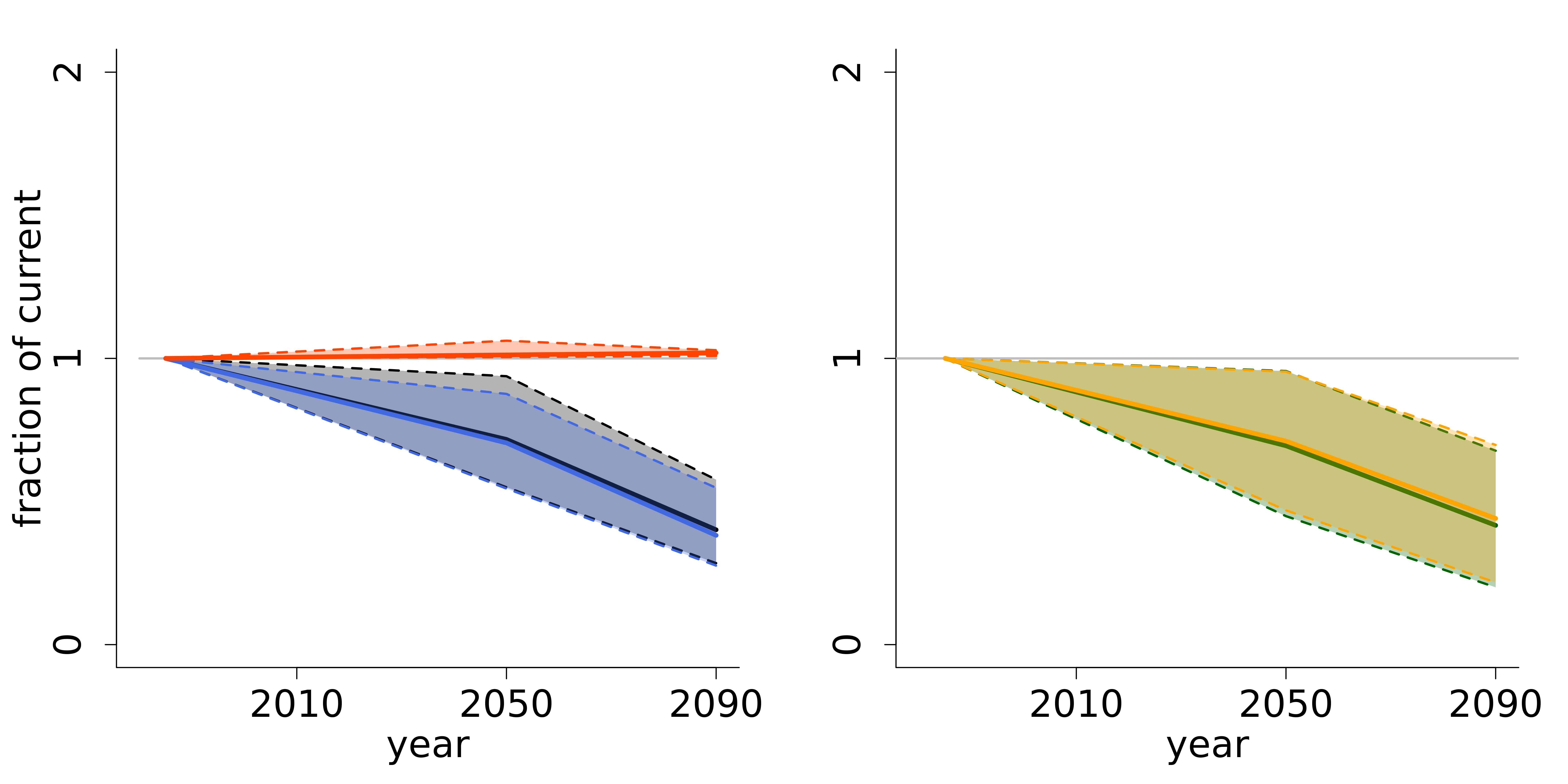

Supplement: S3 Appendix — (ZIP) [file pntd.0014030.s007.zip › Sup. Mat. 6-2 M-Z - Species Trends/Micrurus_mipartitus_CCTrends.png]

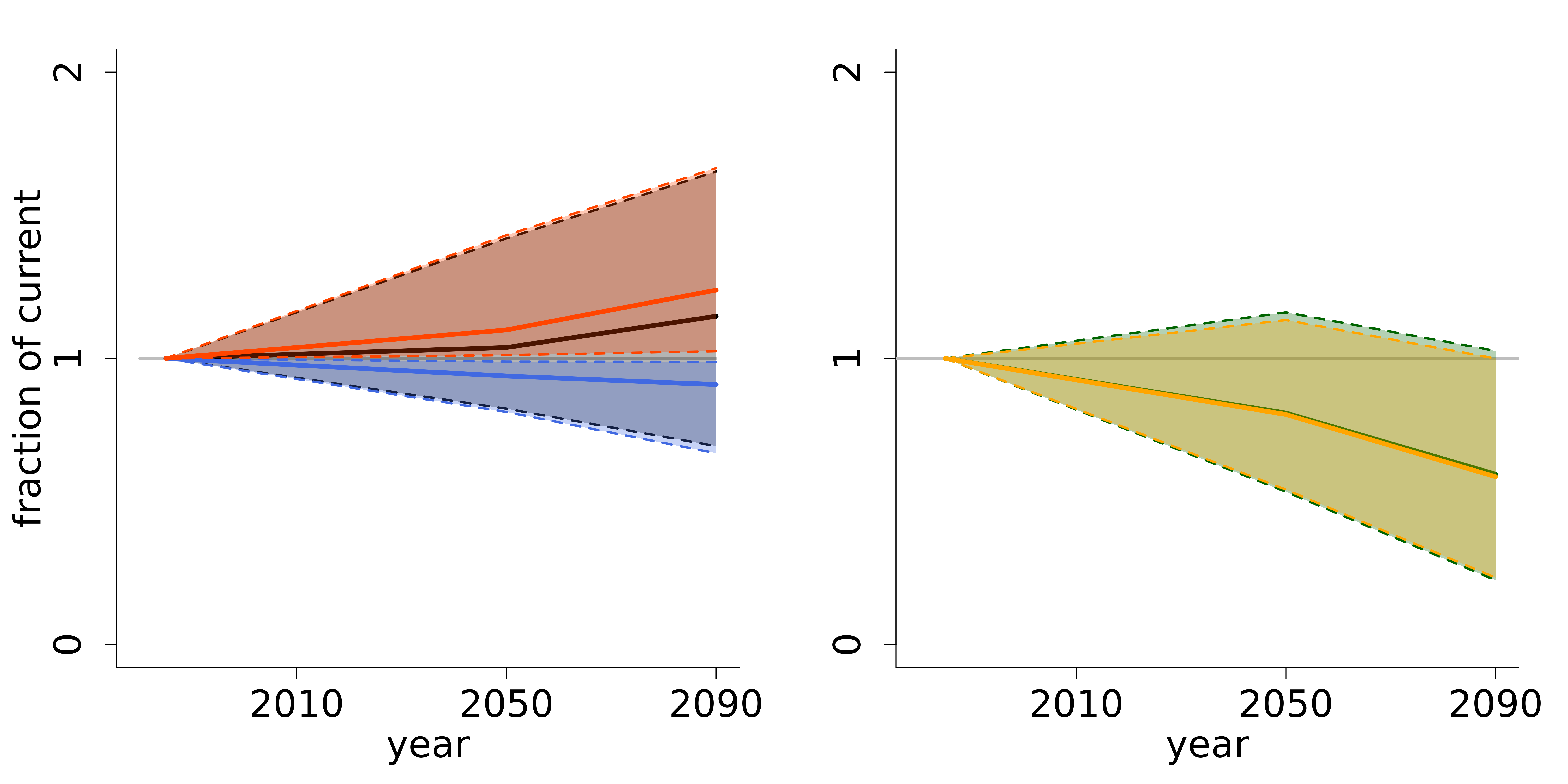

Supplement: S3 Appendix — (ZIP) [file pntd.0014030.s007.zip › Sup. Mat. 6-2 M-Z - Species Trends/Micrurus_mosquitensis_CCTrends.png]

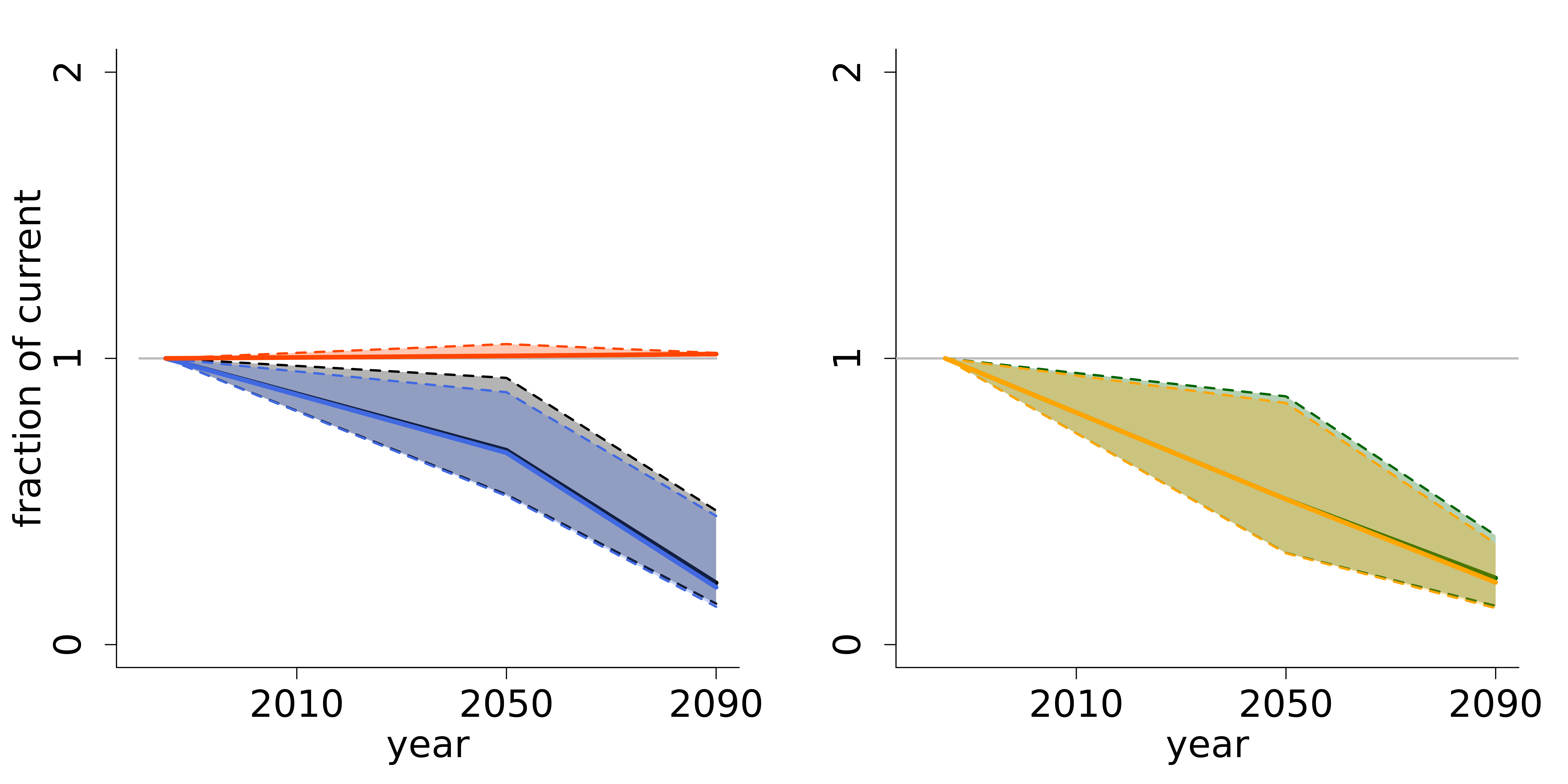

Supplement: S3 Appendix — (ZIP) [file pntd.0014030.s007.zip › Sup. Mat. 6-2 M-Z - Species Trends/Micrurus_multifasciatus_CCTrends.png]

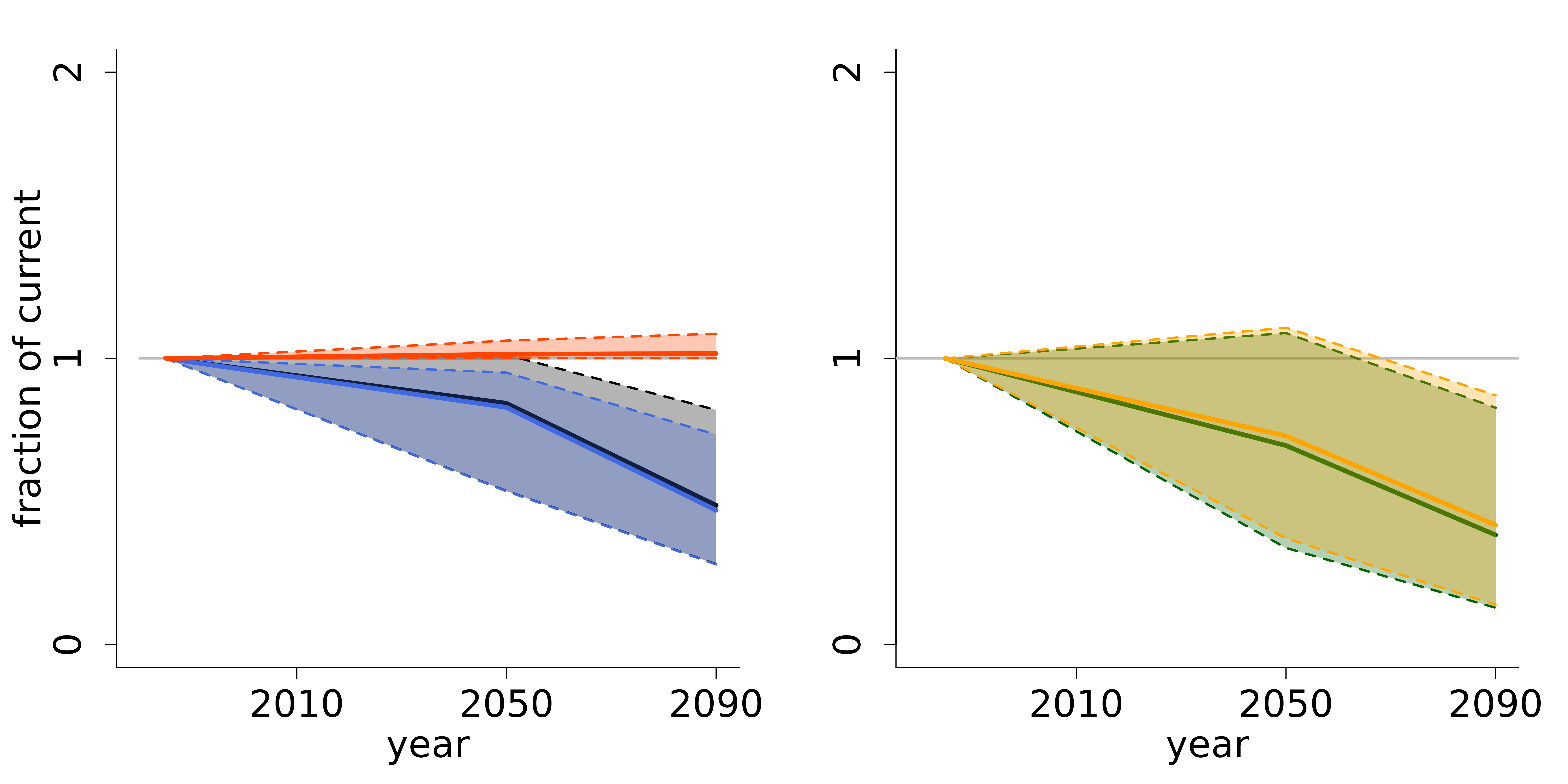

Supplement: S3 Appendix — (ZIP) [file pntd.0014030.s007.zip › Sup. Mat. 6-2 M-Z - Species Trends/Micrurus_multiscutatus_CCTrends.png]

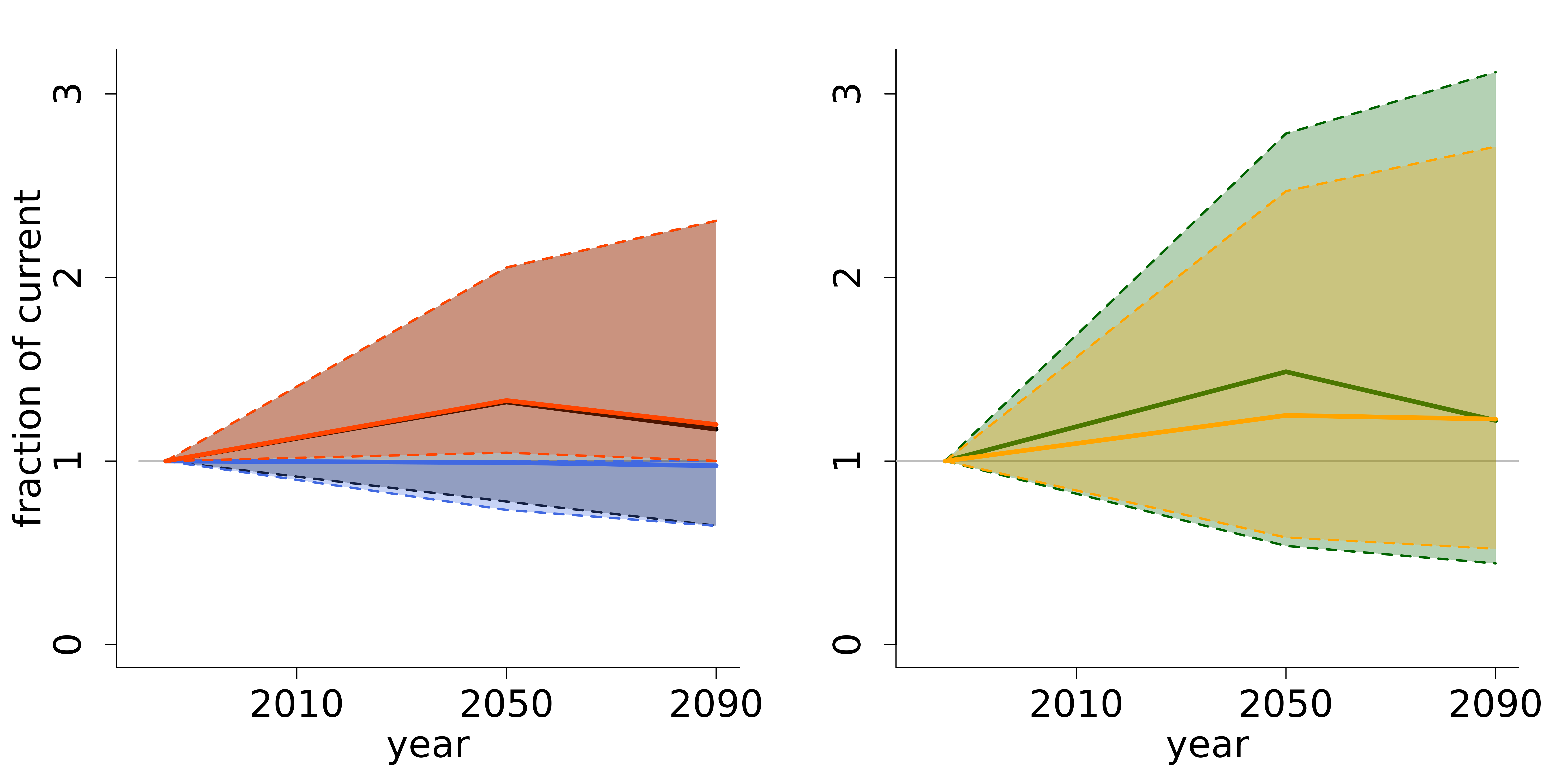

Supplement: S3 Appendix — (ZIP) [file pntd.0014030.s007.zip › Sup. Mat. 6-2 M-Z - Species Trends/Micrurus_narduccii_CCTrends.png]

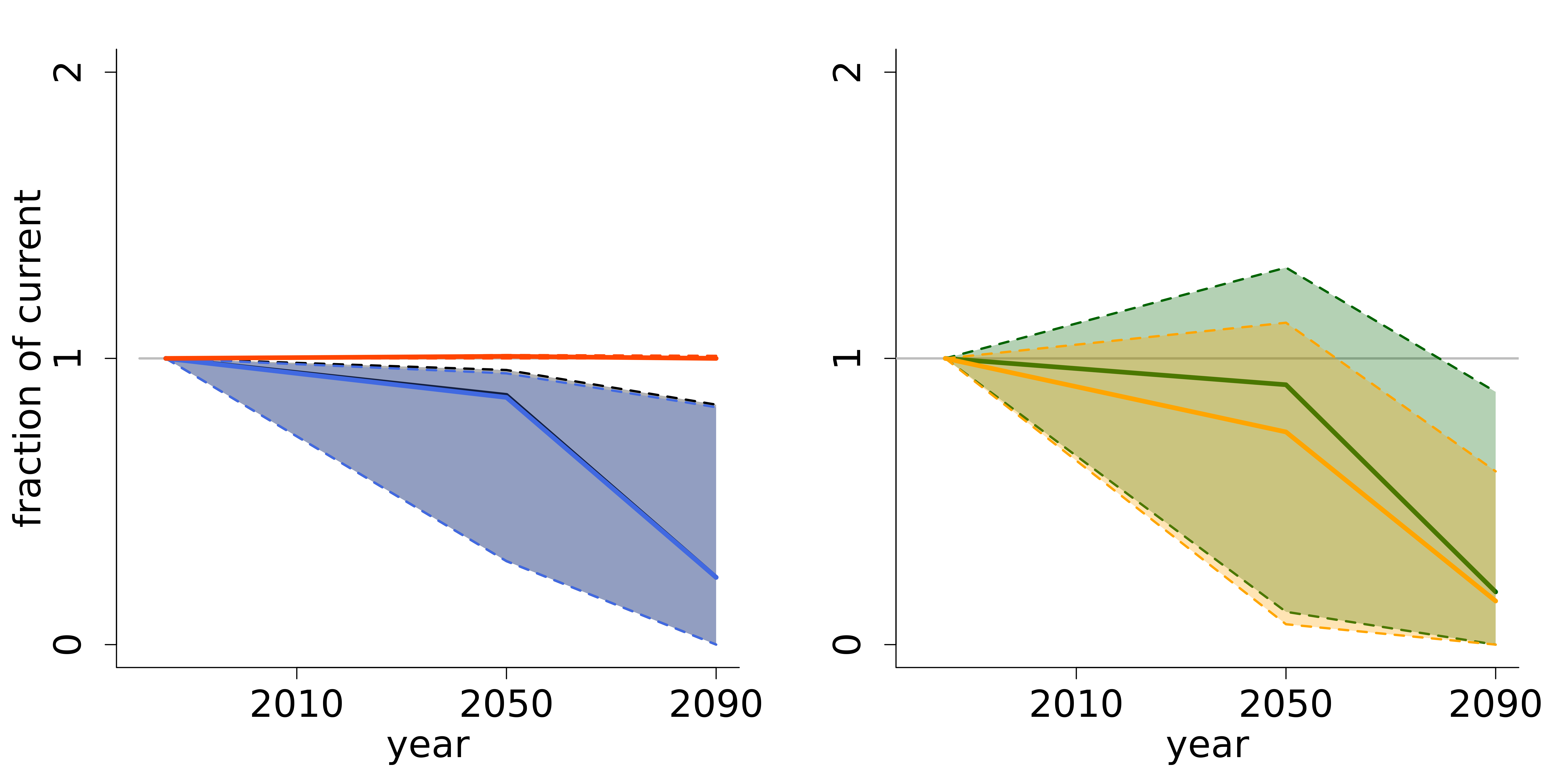

Supplement: S3 Appendix — (ZIP) [file pntd.0014030.s007.zip › Sup. Mat. 6-2 M-Z - Species Trends/Micrurus_nattereri_CCTrends.png]

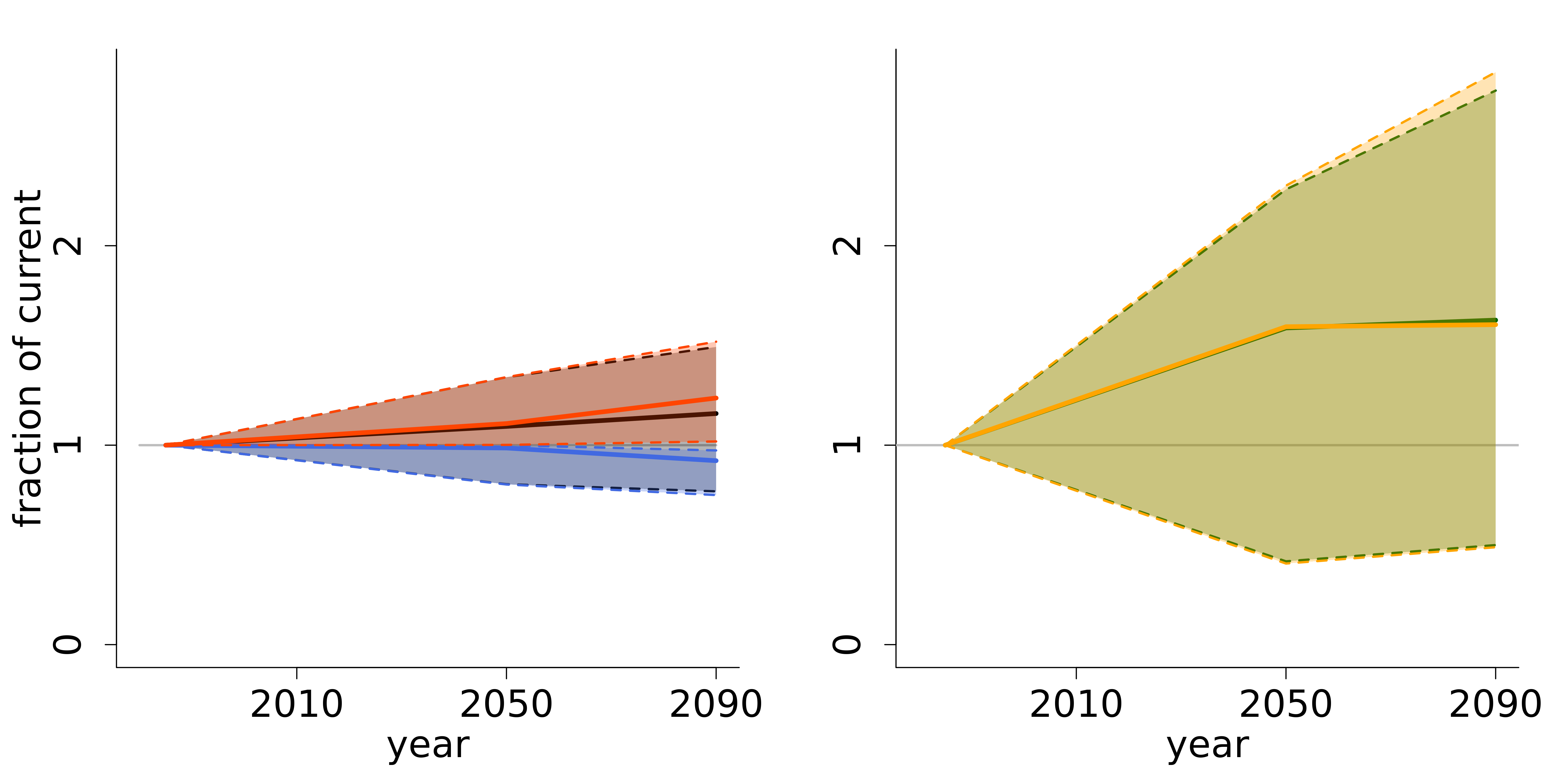

Supplement: S3 Appendix — (ZIP) [file pntd.0014030.s007.zip › Sup. Mat. 6-2 M-Z - Species Trends/Micrurus_nebularis_CCTrends.png]

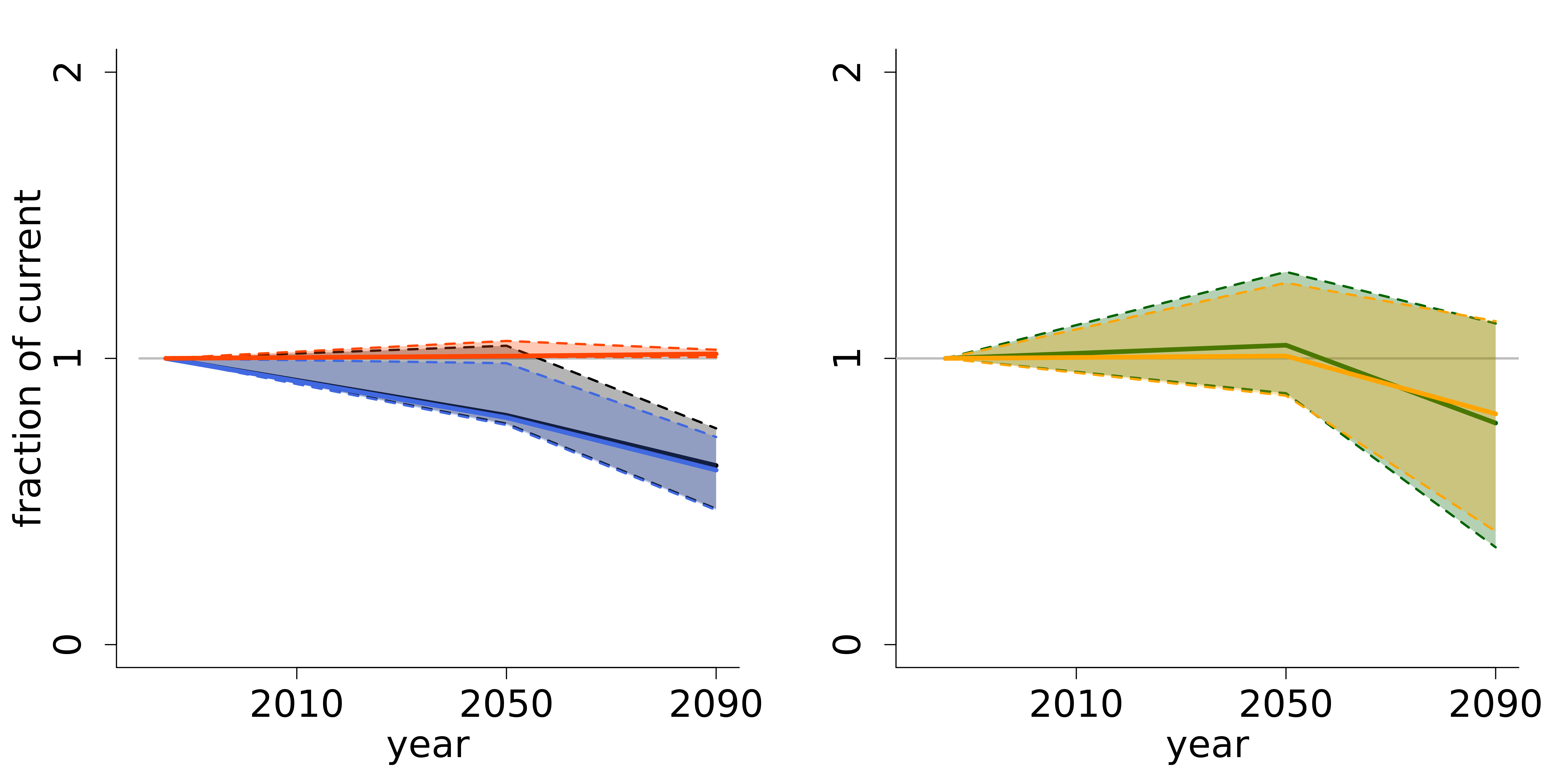

Supplement: S3 Appendix — (ZIP) [file pntd.0014030.s007.zip › Sup. Mat. 6-2 M-Z - Species Trends/Micrurus_nigrocinctus_CCTrends.png]

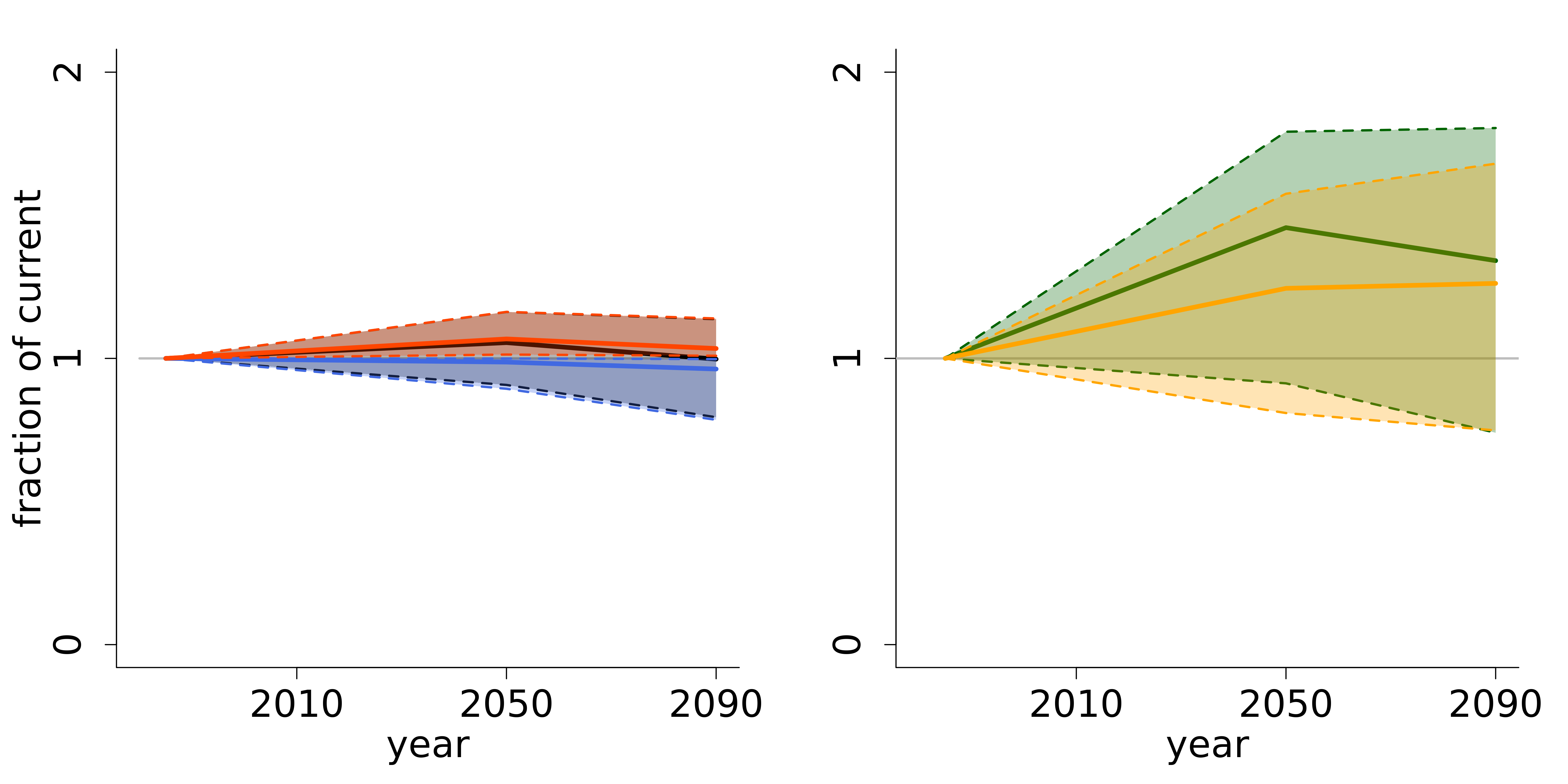

Supplement: S3 Appendix — (ZIP) [file pntd.0014030.s007.zip › Sup. Mat. 6-2 M-Z - Species Trends/Micrurus_obscurus_CCTrends.png]

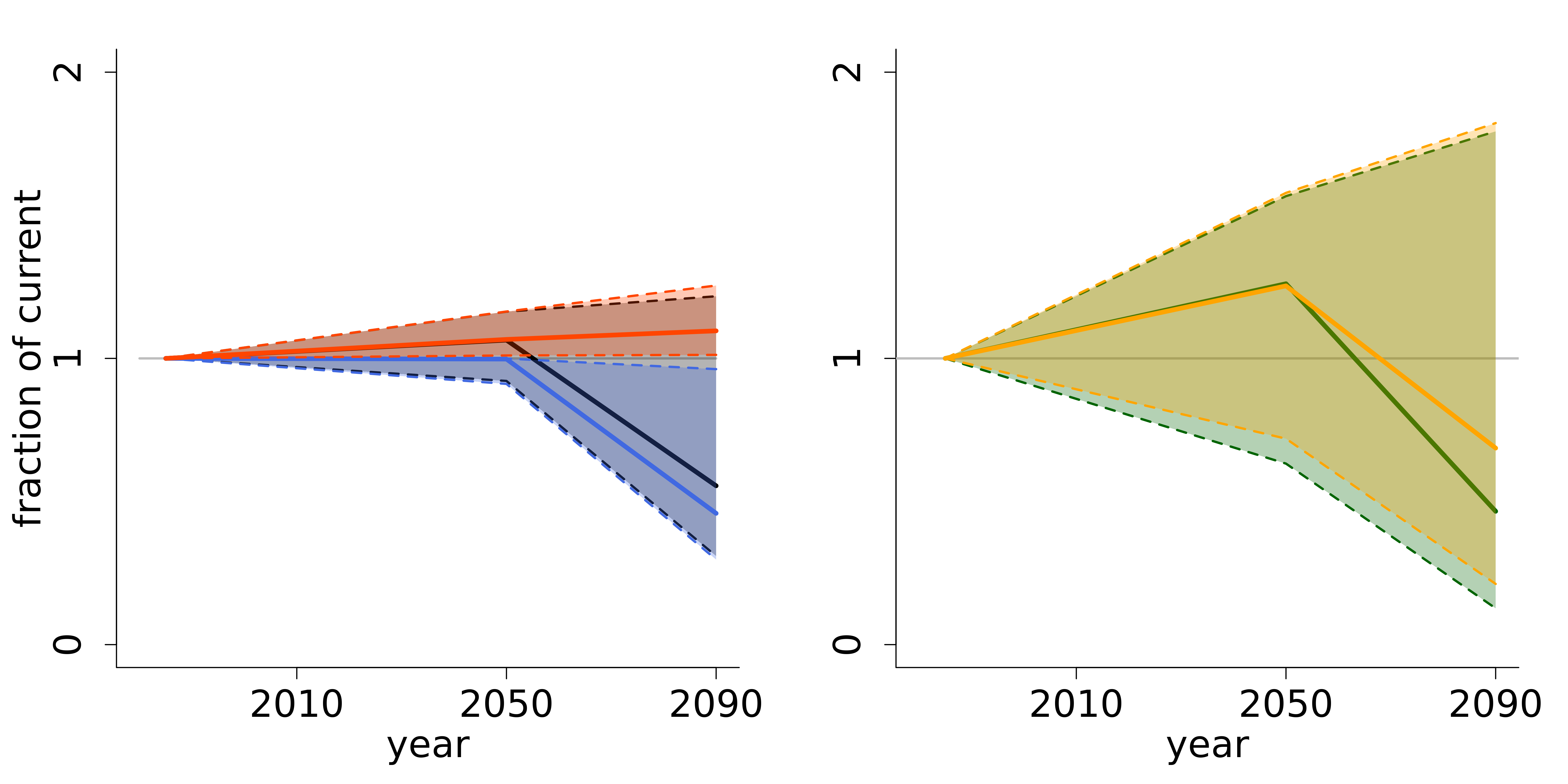

Supplement: S3 Appendix — (ZIP) [file pntd.0014030.s007.zip › Sup. Mat. 6-2 M-Z - Species Trends/Micrurus_ornatissimus_CCTrends.png]

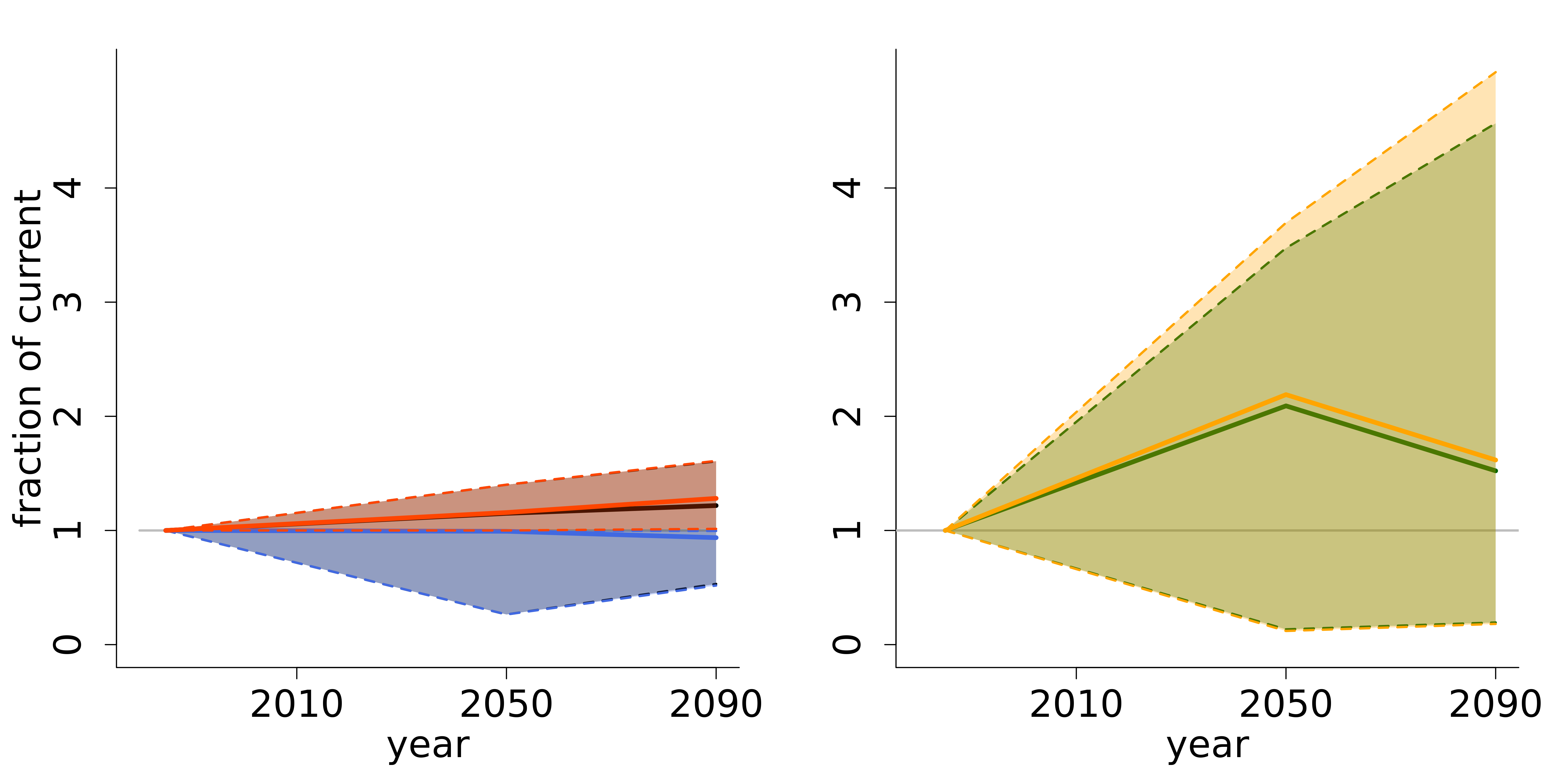

Supplement: S3 Appendix — (ZIP) [file pntd.0014030.s007.zip › Sup. Mat. 6-2 M-Z - Species Trends/Micrurus_pachecogili_CCTrends.png]

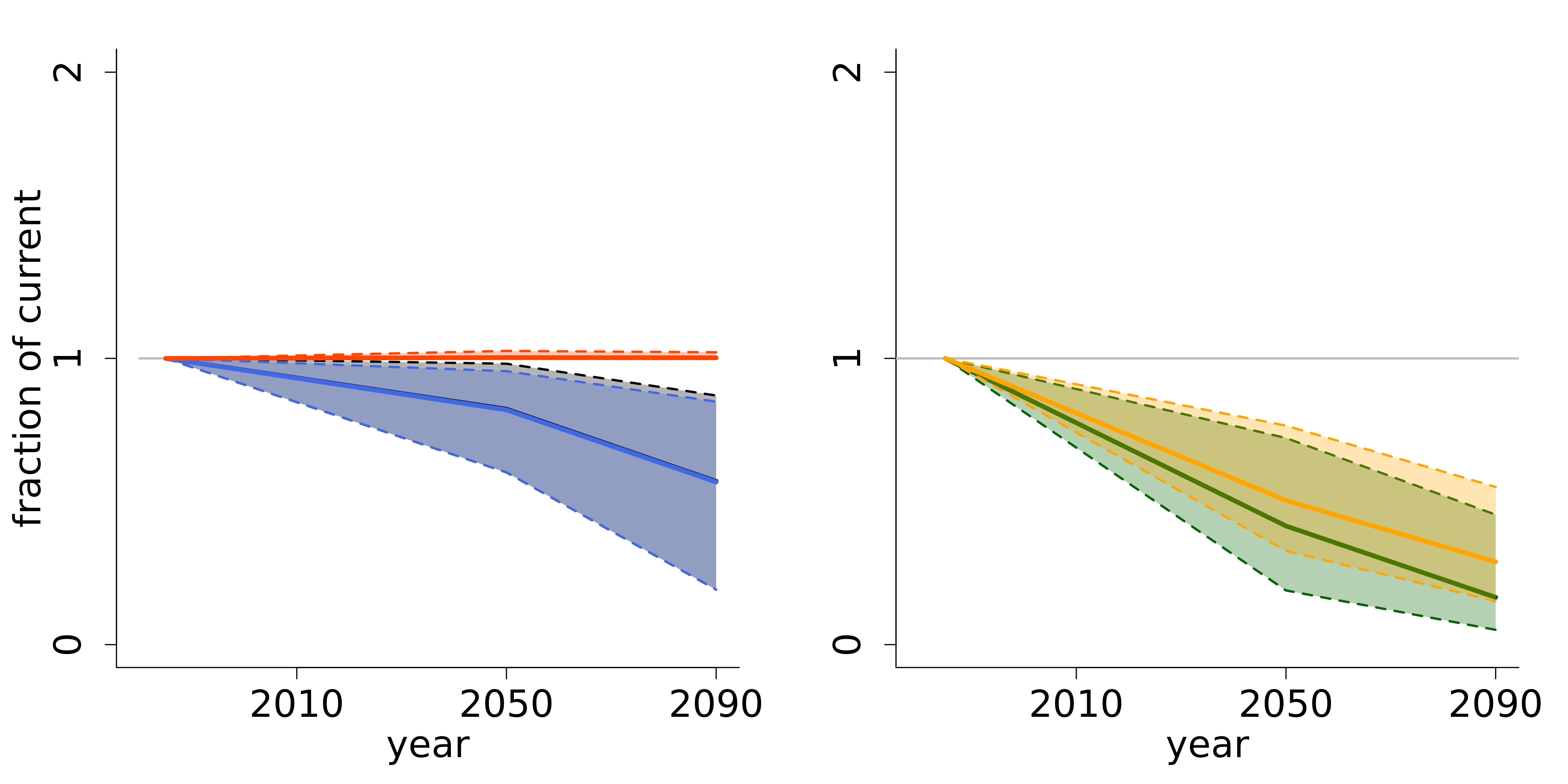

Supplement: S3 Appendix — (ZIP) [file pntd.0014030.s007.zip › Sup. Mat. 6-2 M-Z - Species Trends/Micrurus_paraensis_CCTrends.png]

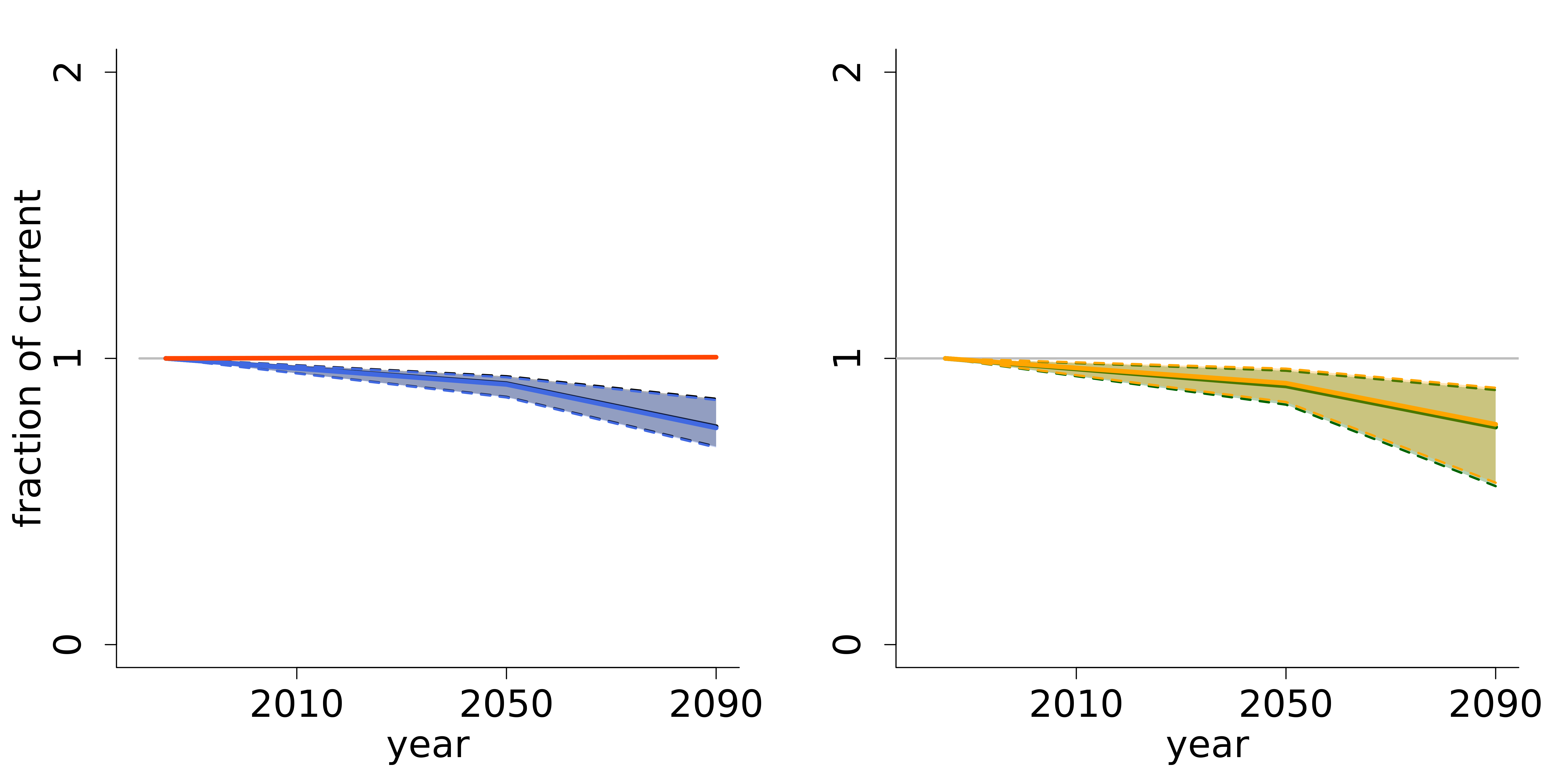

Supplement: S3 Appendix — (ZIP) [file pntd.0014030.s007.zip › Sup. Mat. 6-2 M-Z - Species Trends/Micrurus_peruvianus_CCTrends.png]

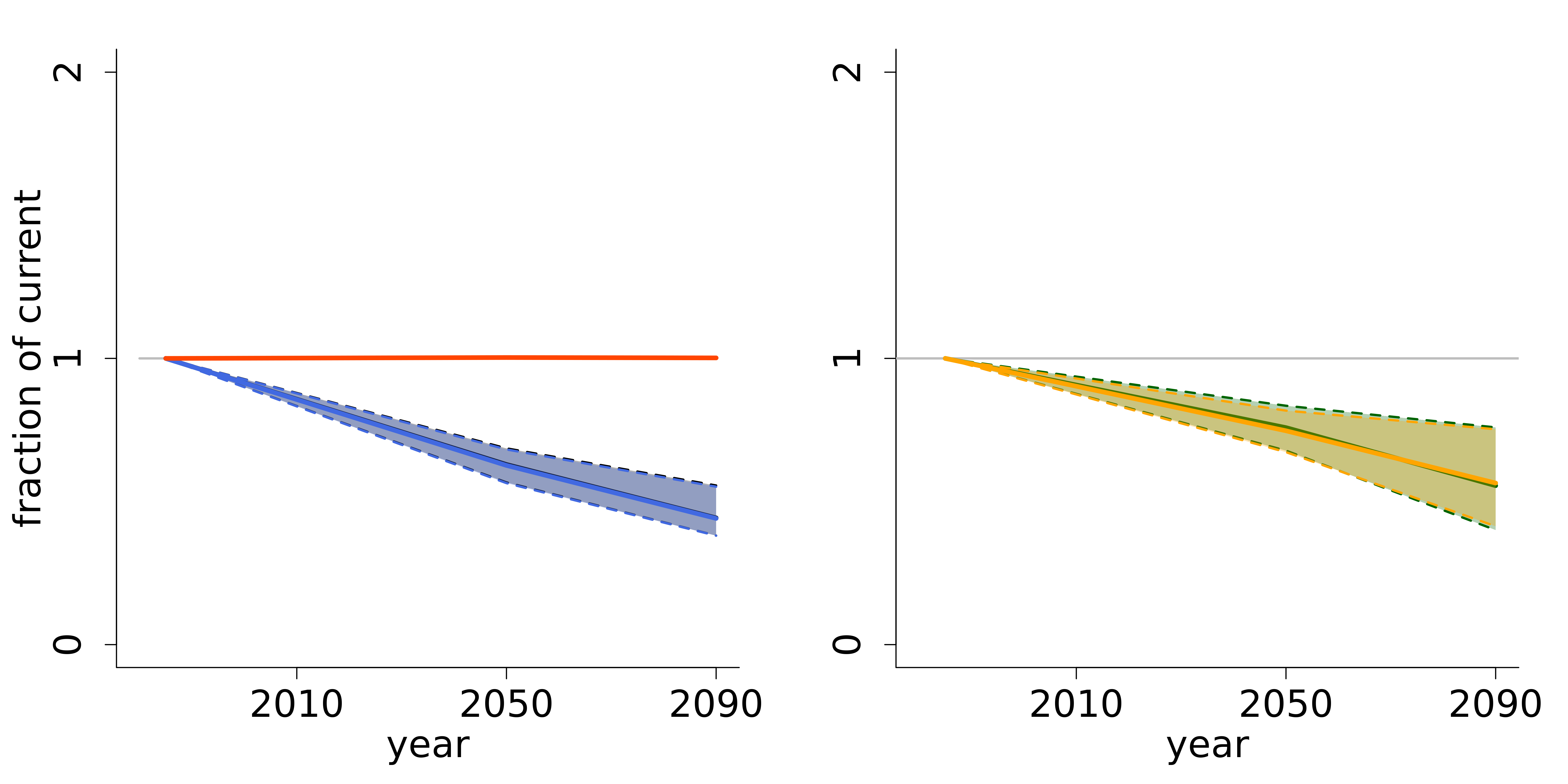

Supplement: S3 Appendix — (ZIP) [file pntd.0014030.s007.zip › Sup. Mat. 6-2 M-Z - Species Trends/Micrurus_petersi_CCTrends.png]

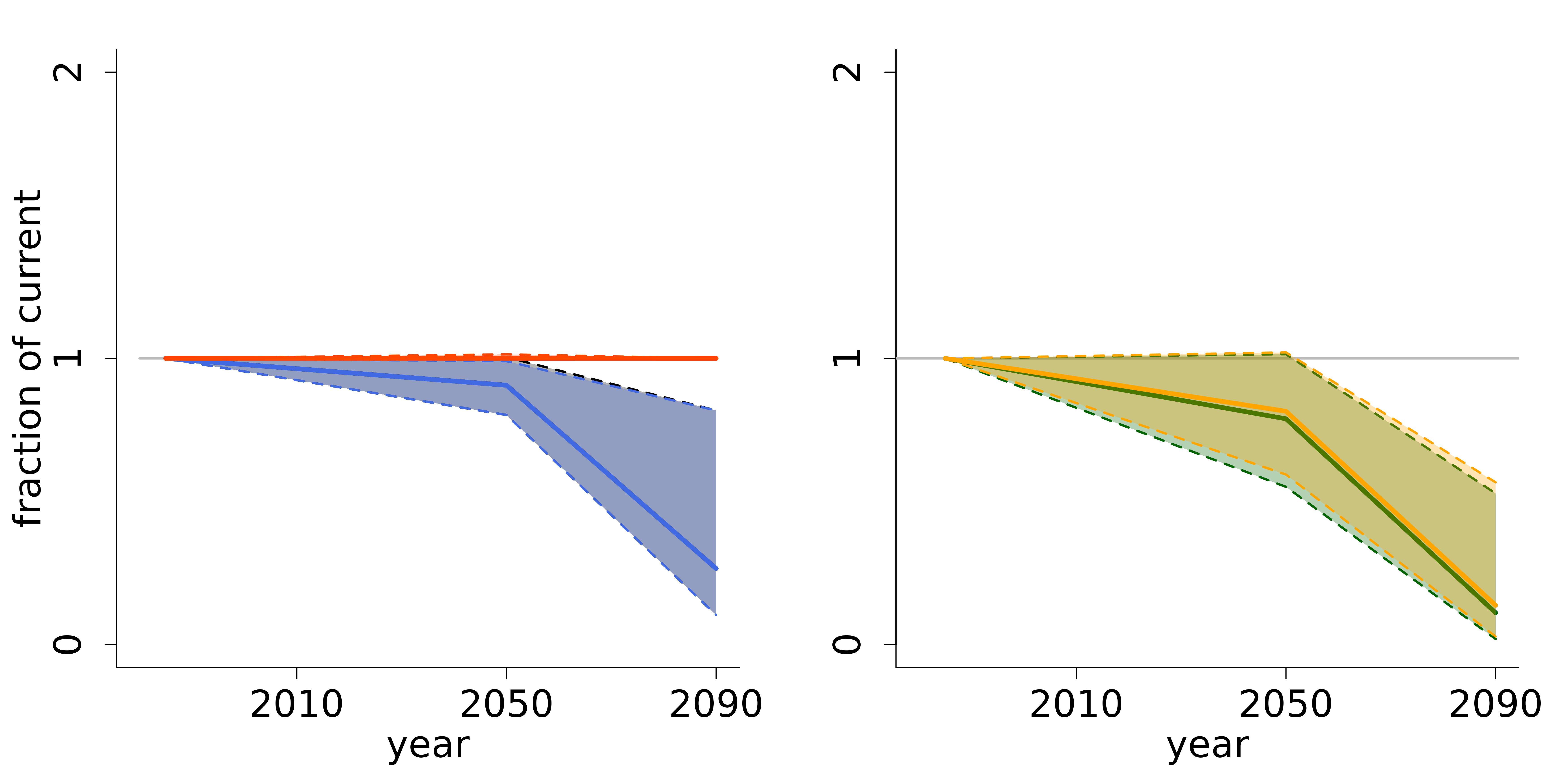

Supplement: S3 Appendix — (ZIP) [file pntd.0014030.s007.zip › Sup. Mat. 6-2 M-Z - Species Trends/Micrurus_potyguara_CCTrends.png]

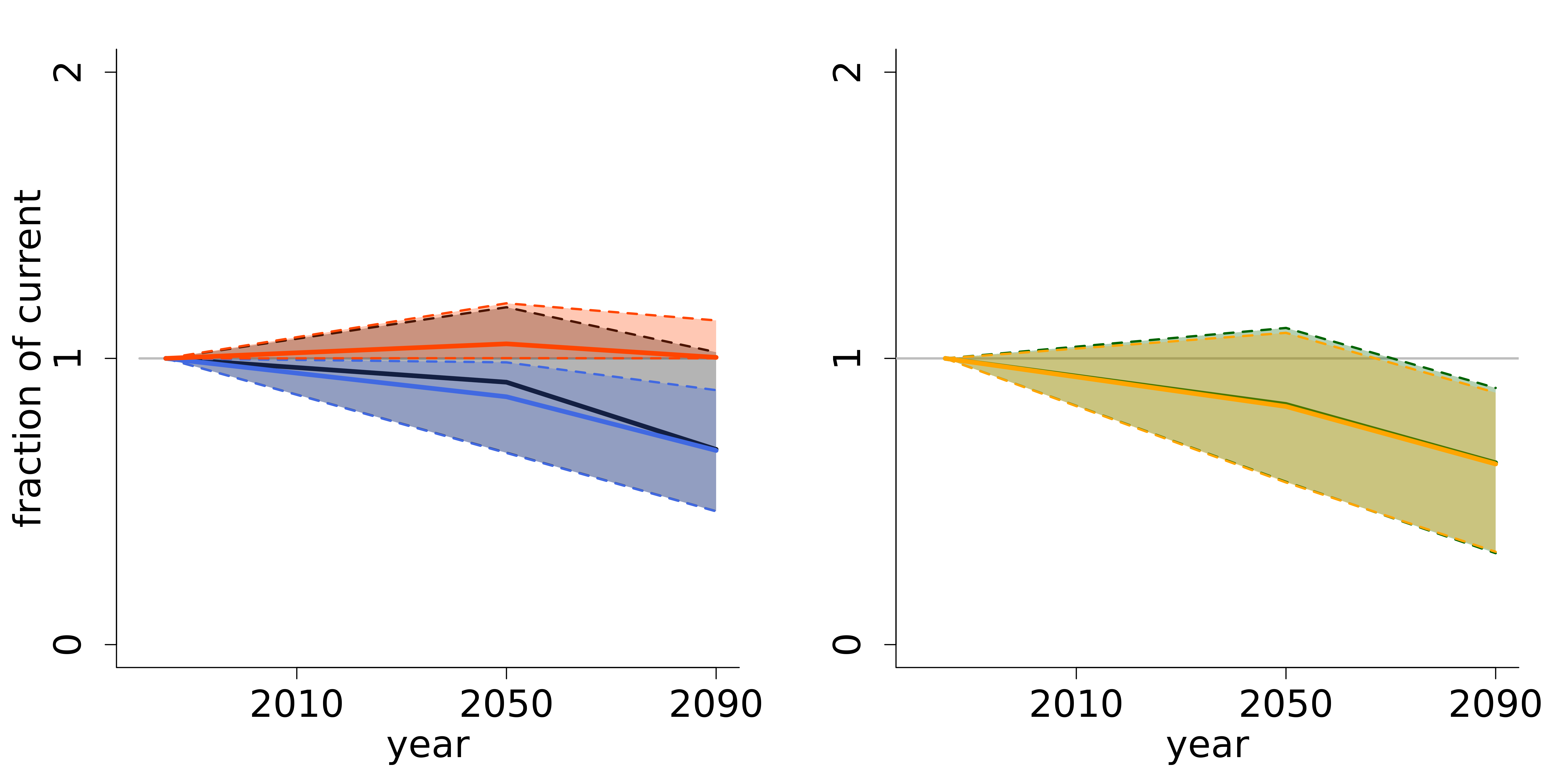

Supplement: S3 Appendix — (ZIP) [file pntd.0014030.s007.zip › Sup. Mat. 6-2 M-Z - Species Trends/Micrurus_proximans_CCTrends.png]

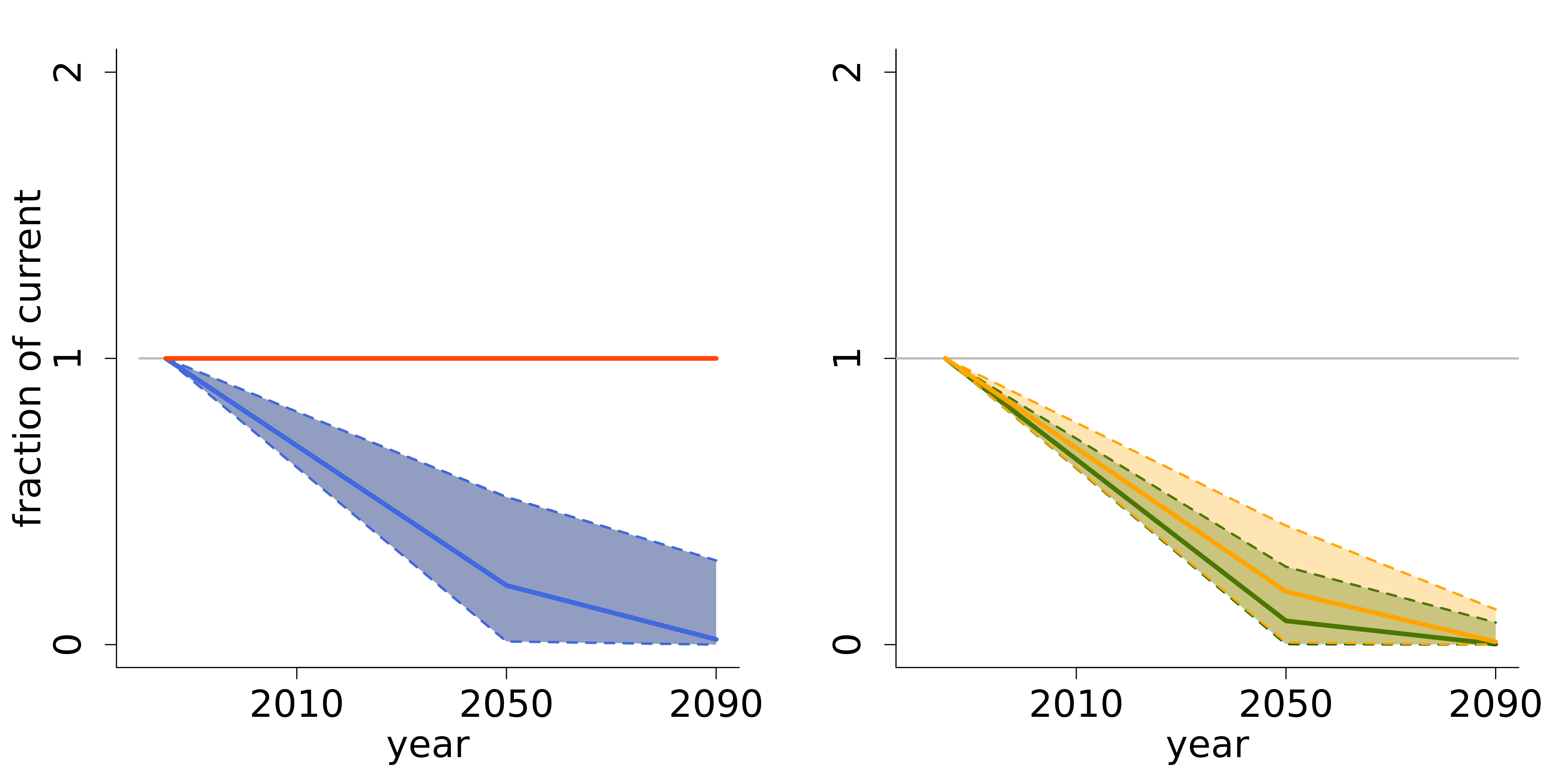

Supplement: S3 Appendix — (ZIP) [file pntd.0014030.s007.zip › Sup. Mat. 6-2 M-Z - Species Trends/Micrurus_psyches_CCTrends.png]

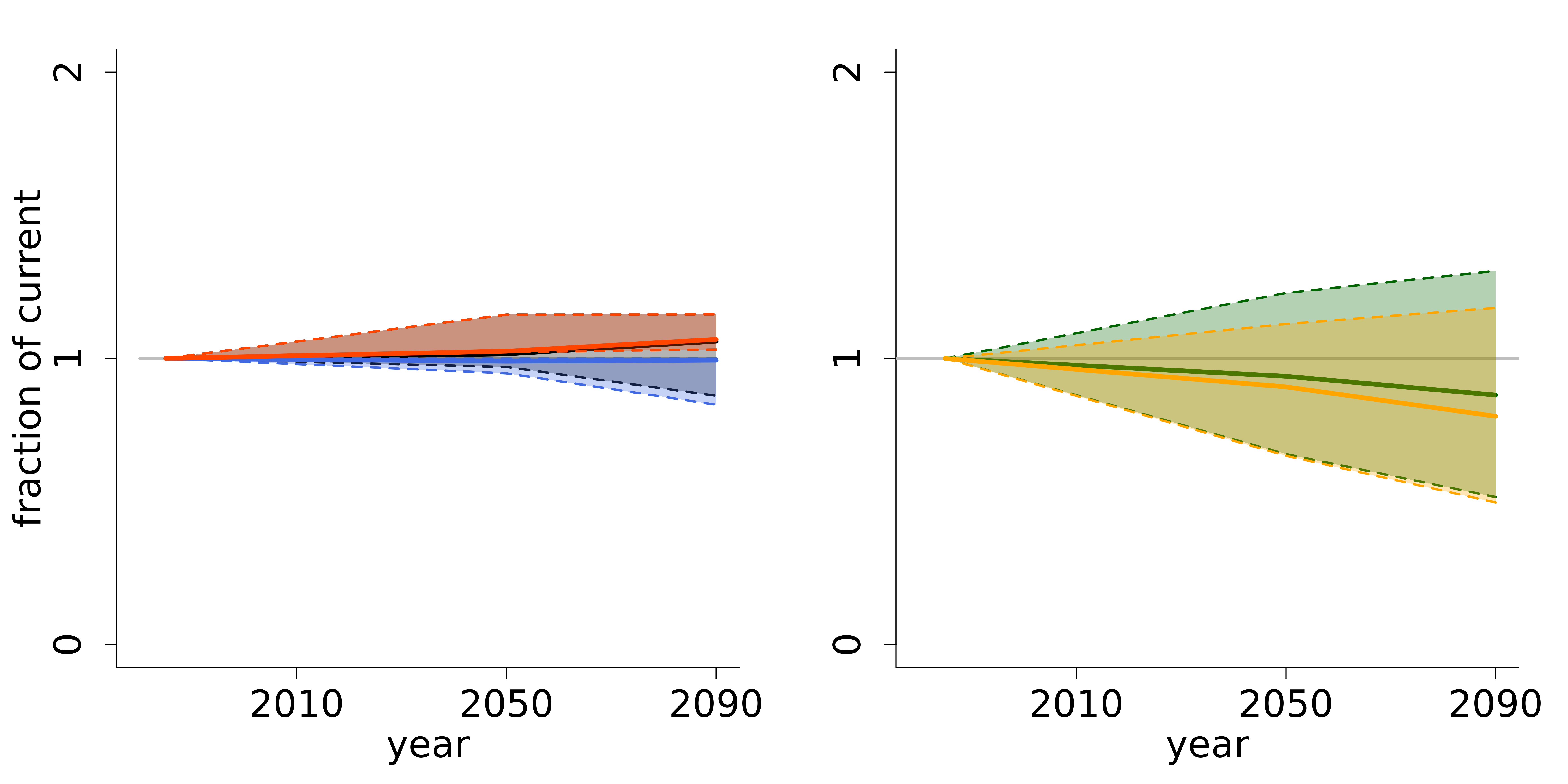

Supplement: S3 Appendix — (ZIP) [file pntd.0014030.s007.zip › Sup. Mat. 6-2 M-Z - Species Trends/Micrurus_putumayensis_CCTrends.png]

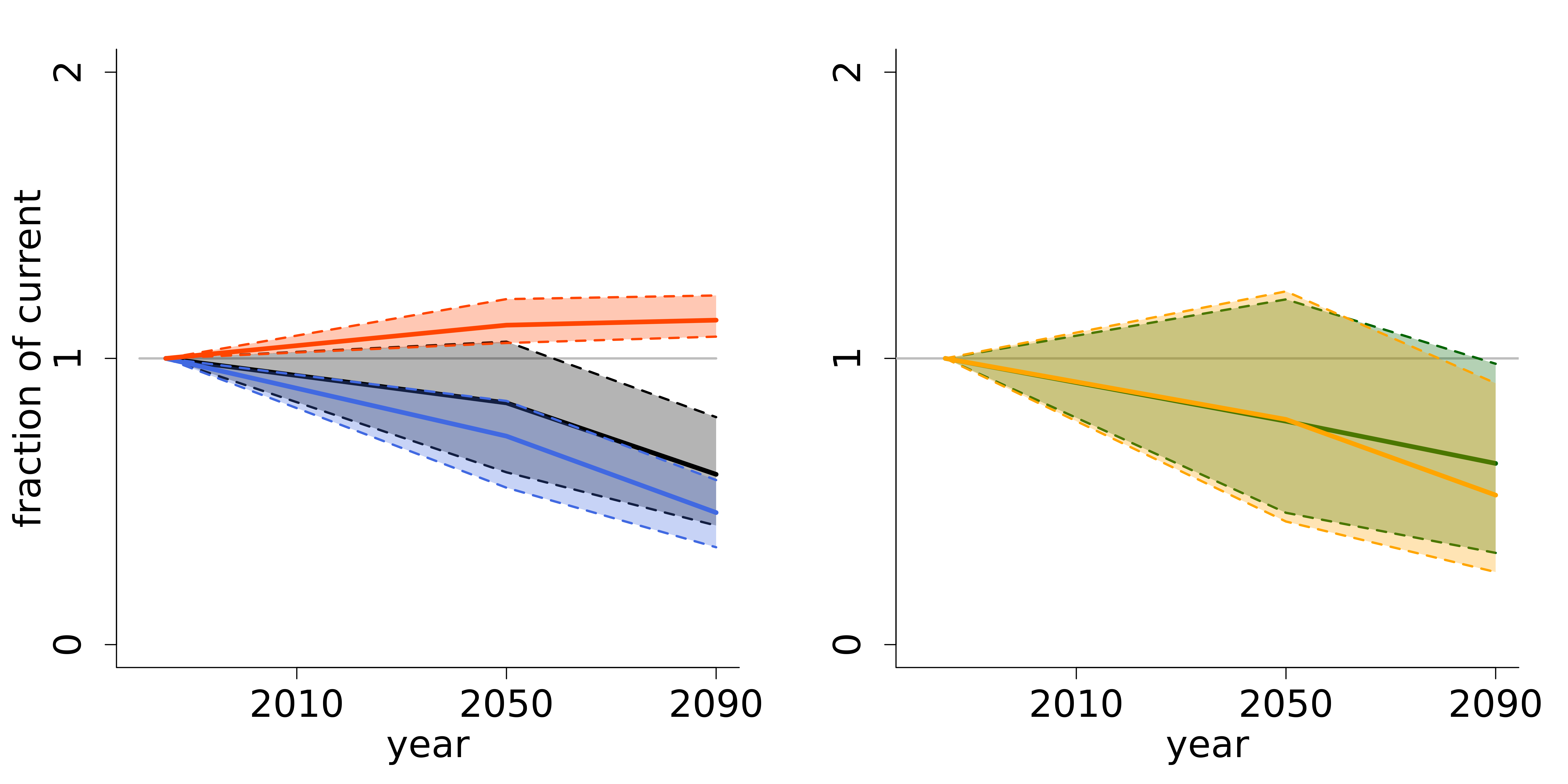

Supplement: S3 Appendix — (ZIP) [file pntd.0014030.s007.zip › Sup. Mat. 6-2 M-Z - Species Trends/Micrurus_pyrrhocryptus_CCTrends.png]

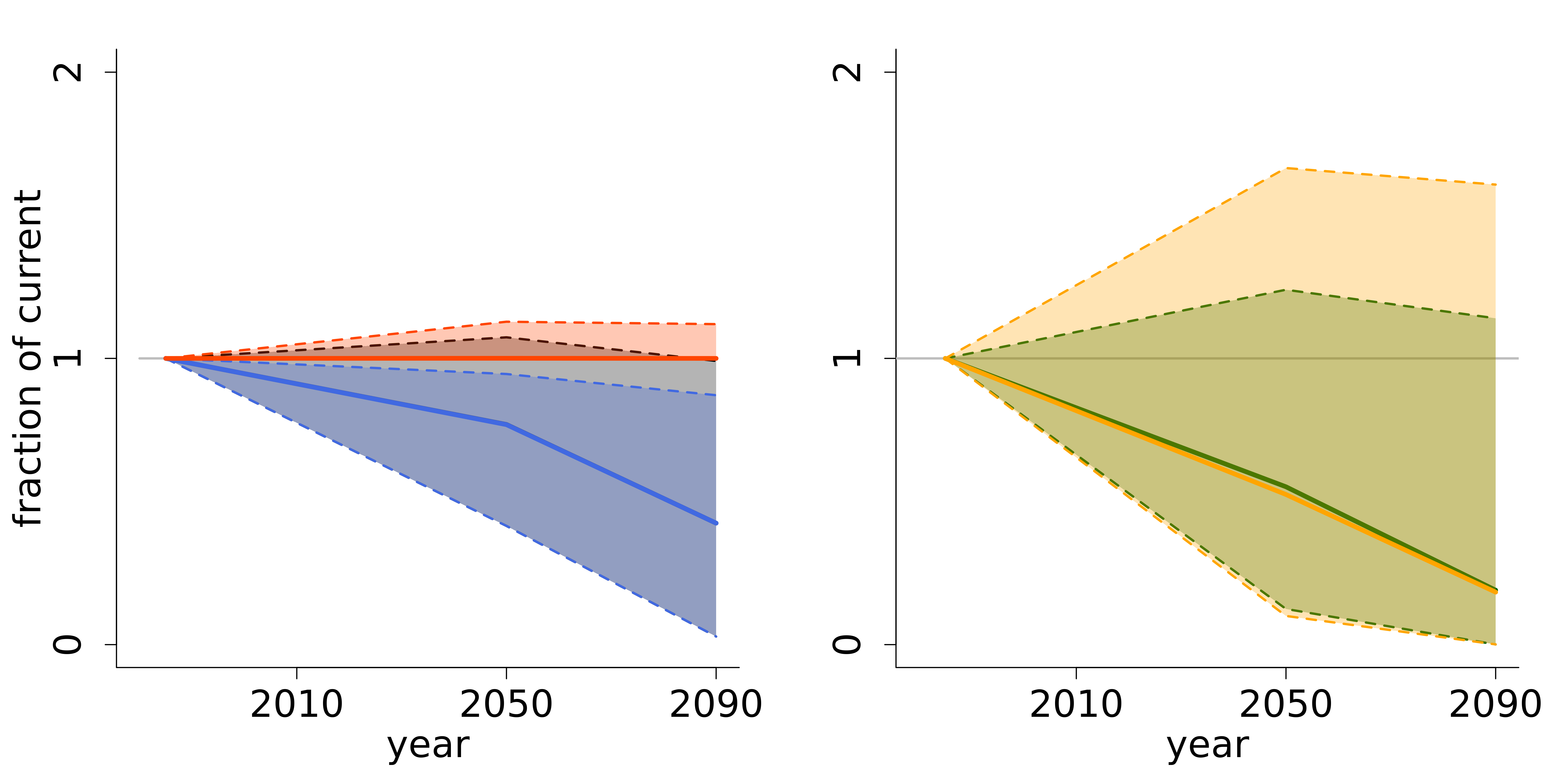

Supplement: S3 Appendix — (ZIP) [file pntd.0014030.s007.zip › Sup. Mat. 6-2 M-Z - Species Trends/Micrurus_remotus_CCTrends.png]

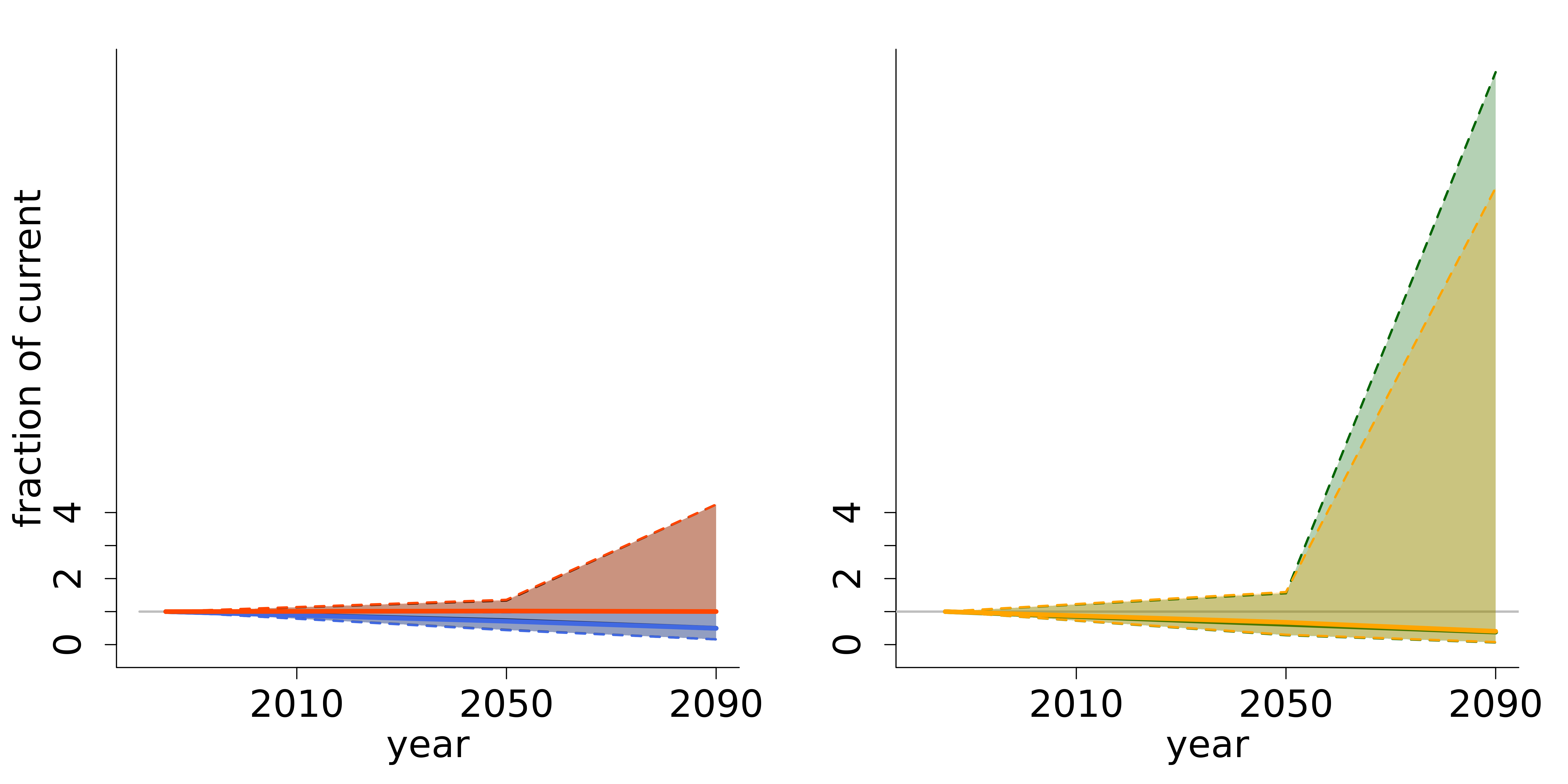

Supplement: S3 Appendix — (ZIP) [file pntd.0014030.s007.zip › Sup. Mat. 6-2 M-Z - Species Trends/Micrurus_renjifoi_CCTrends.png]

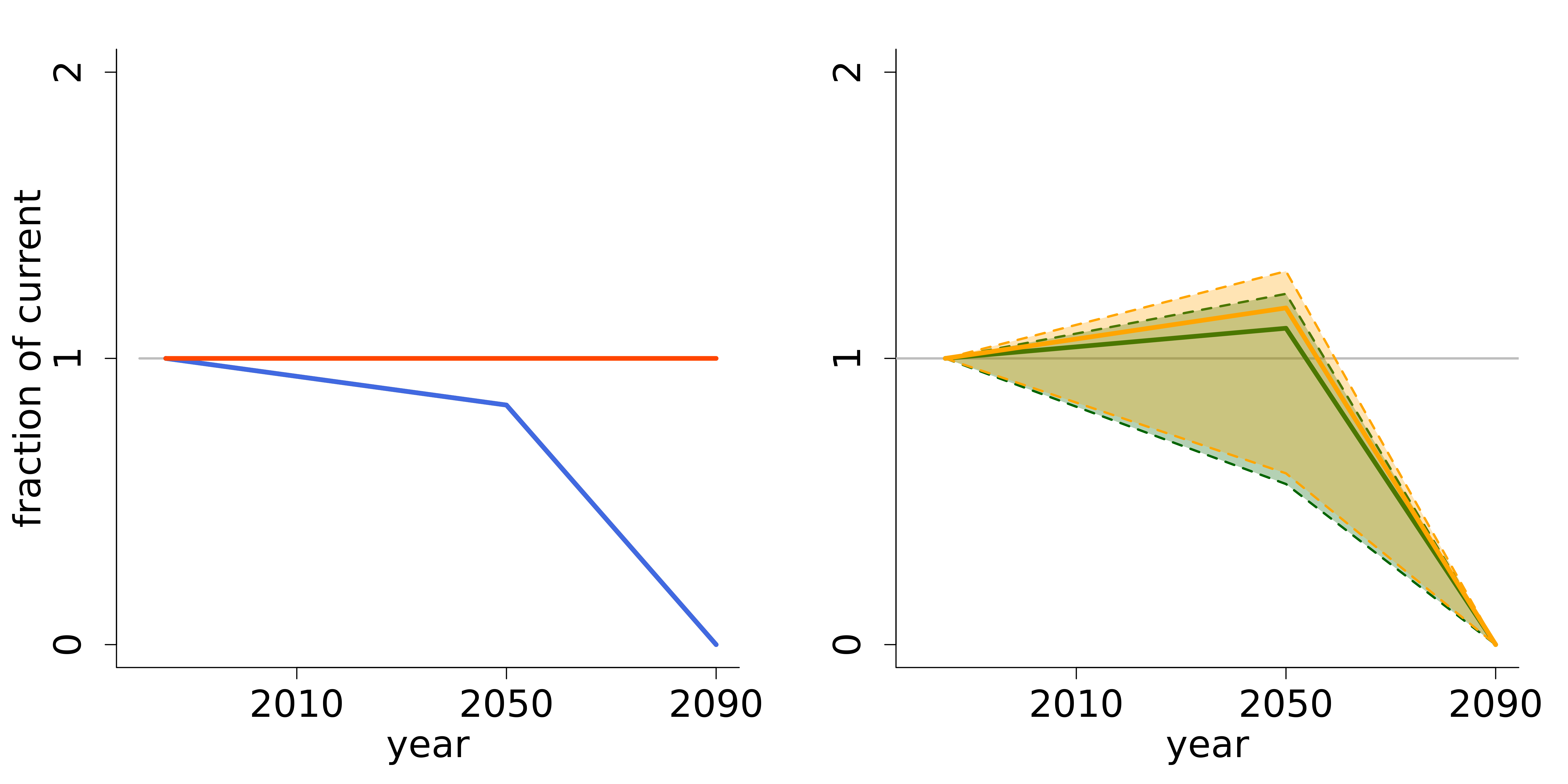

Supplement: S3 Appendix — (ZIP) [file pntd.0014030.s007.zip › Sup. Mat. 6-2 M-Z - Species Trends/Micrurus_ruatanus_CCTrends.png]

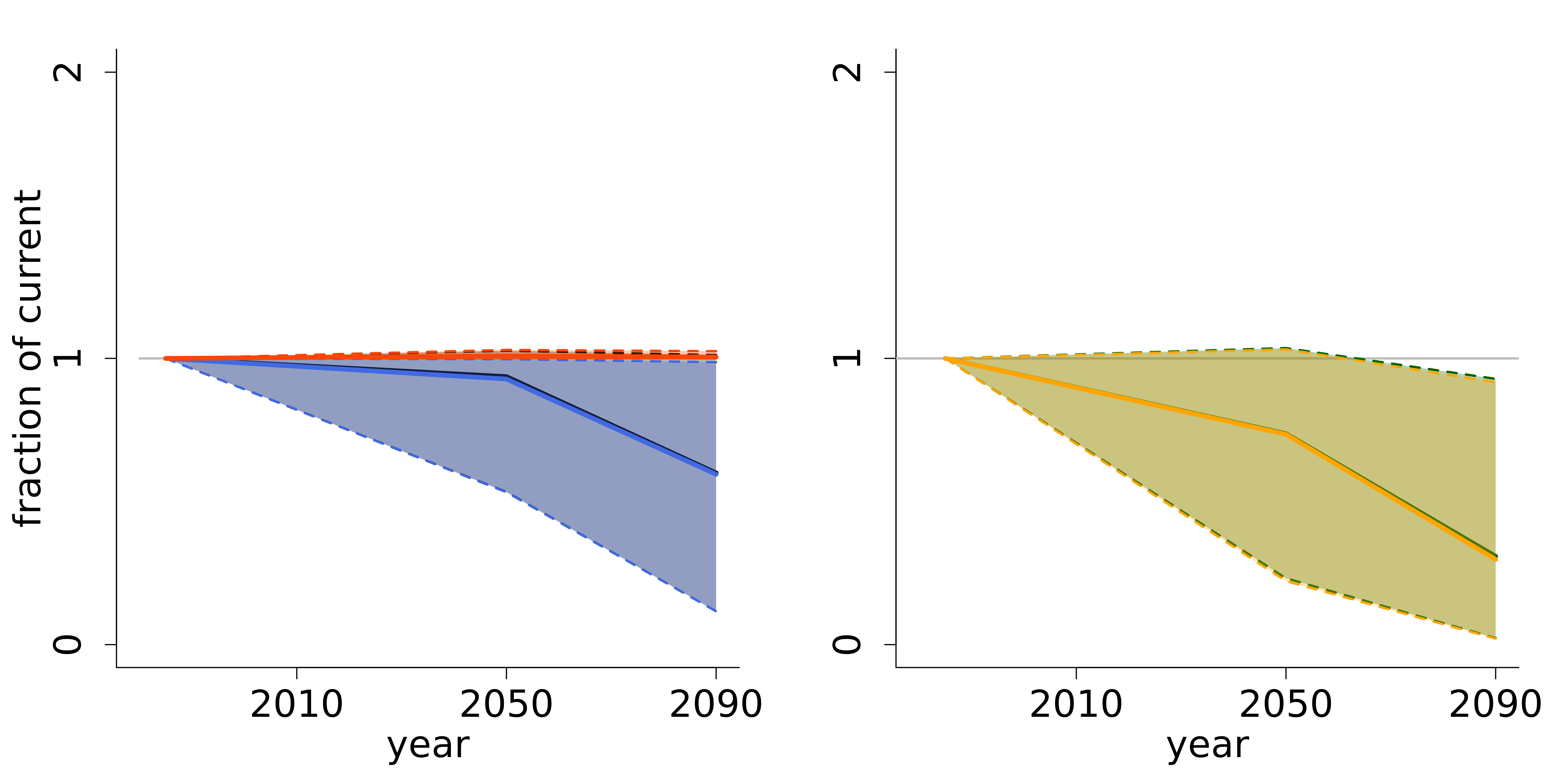

Supplement: S3 Appendix — (ZIP) [file pntd.0014030.s007.zip › Sup. Mat. 6-2 M-Z - Species Trends/Micrurus_sangilensis_CCTrends.png]

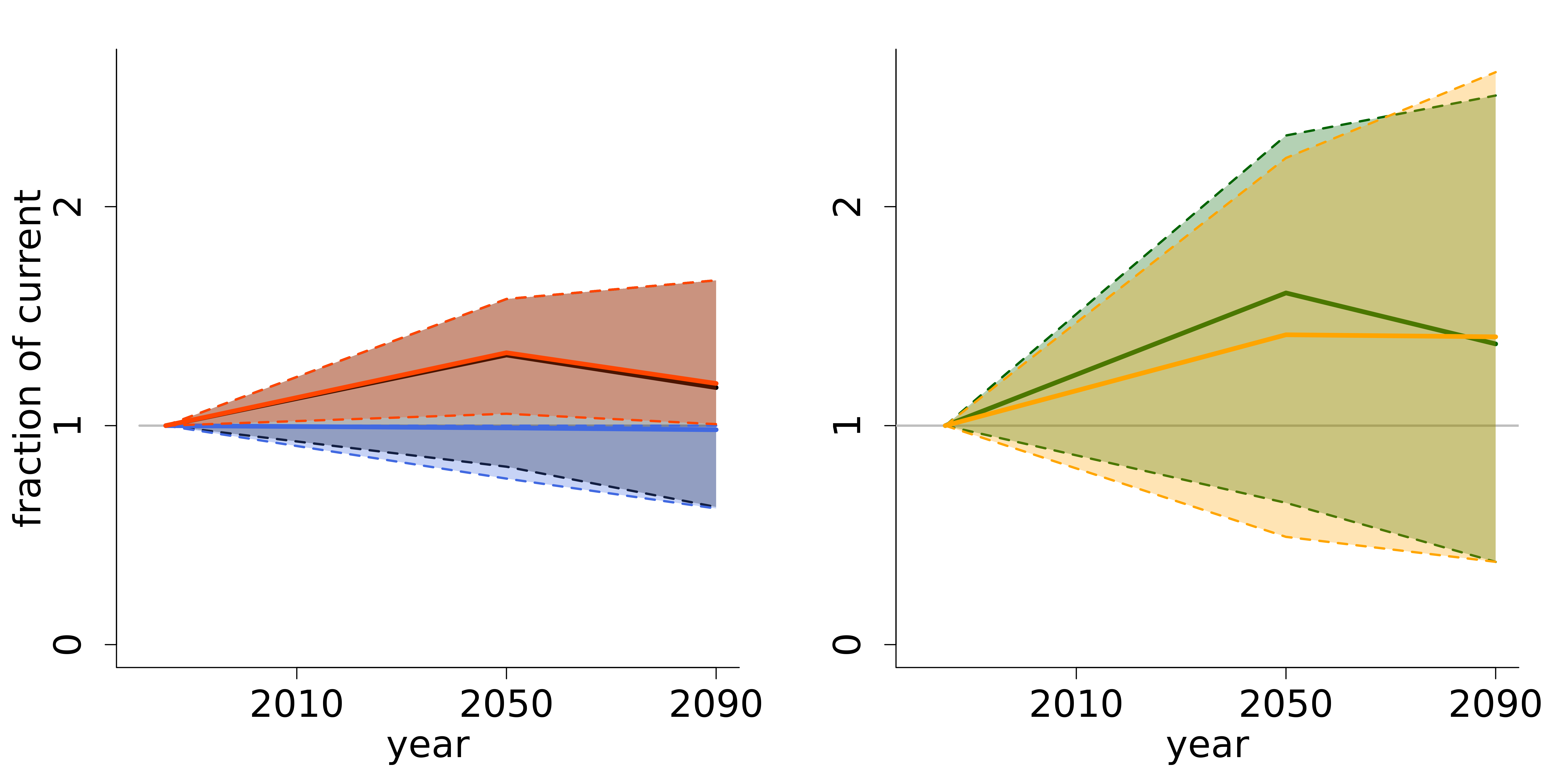

Supplement: S3 Appendix — (ZIP) [file pntd.0014030.s007.zip › Sup. Mat. 6-2 M-Z - Species Trends/Micrurus_scutiventris_CCTrends.png]

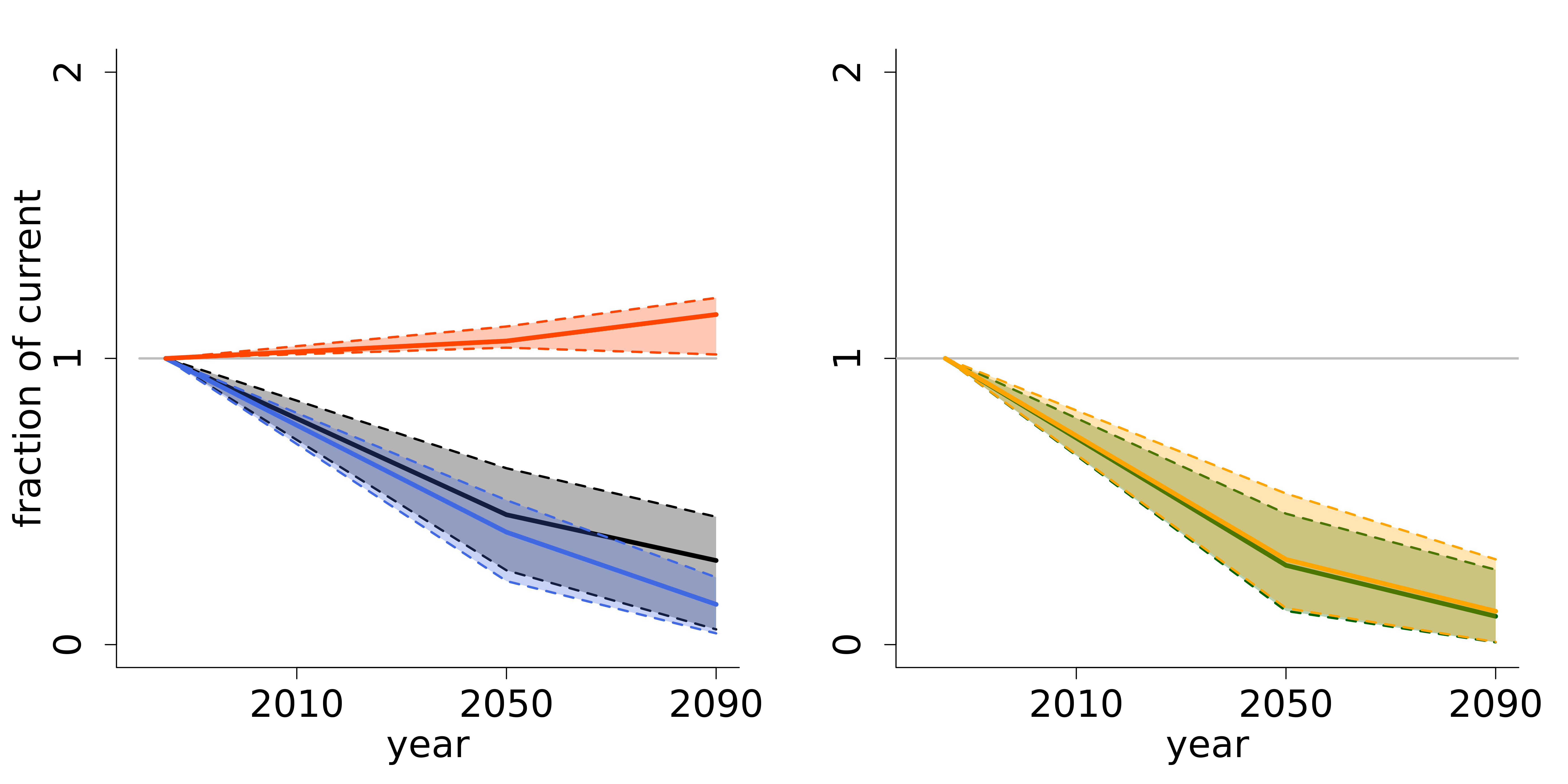

Supplement: S3 Appendix — (ZIP) [file pntd.0014030.s007.zip › Sup. Mat. 6-2 M-Z - Species Trends/Micrurus_serranus_CCTrends.png]

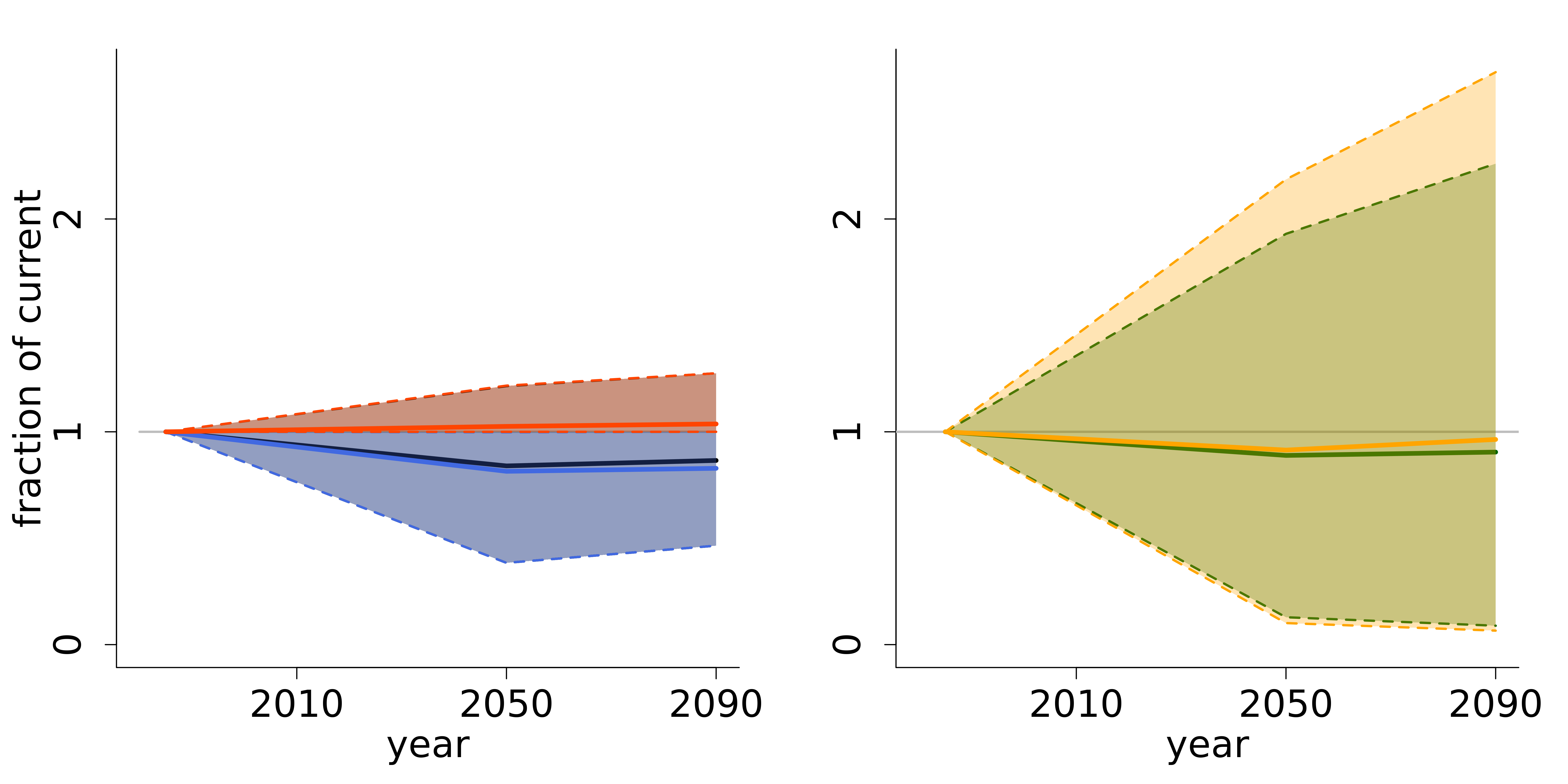

Supplement: S3 Appendix — (ZIP) [file pntd.0014030.s007.zip › Sup. Mat. 6-2 M-Z - Species Trends/Micrurus_silviae_CCTrends.png]

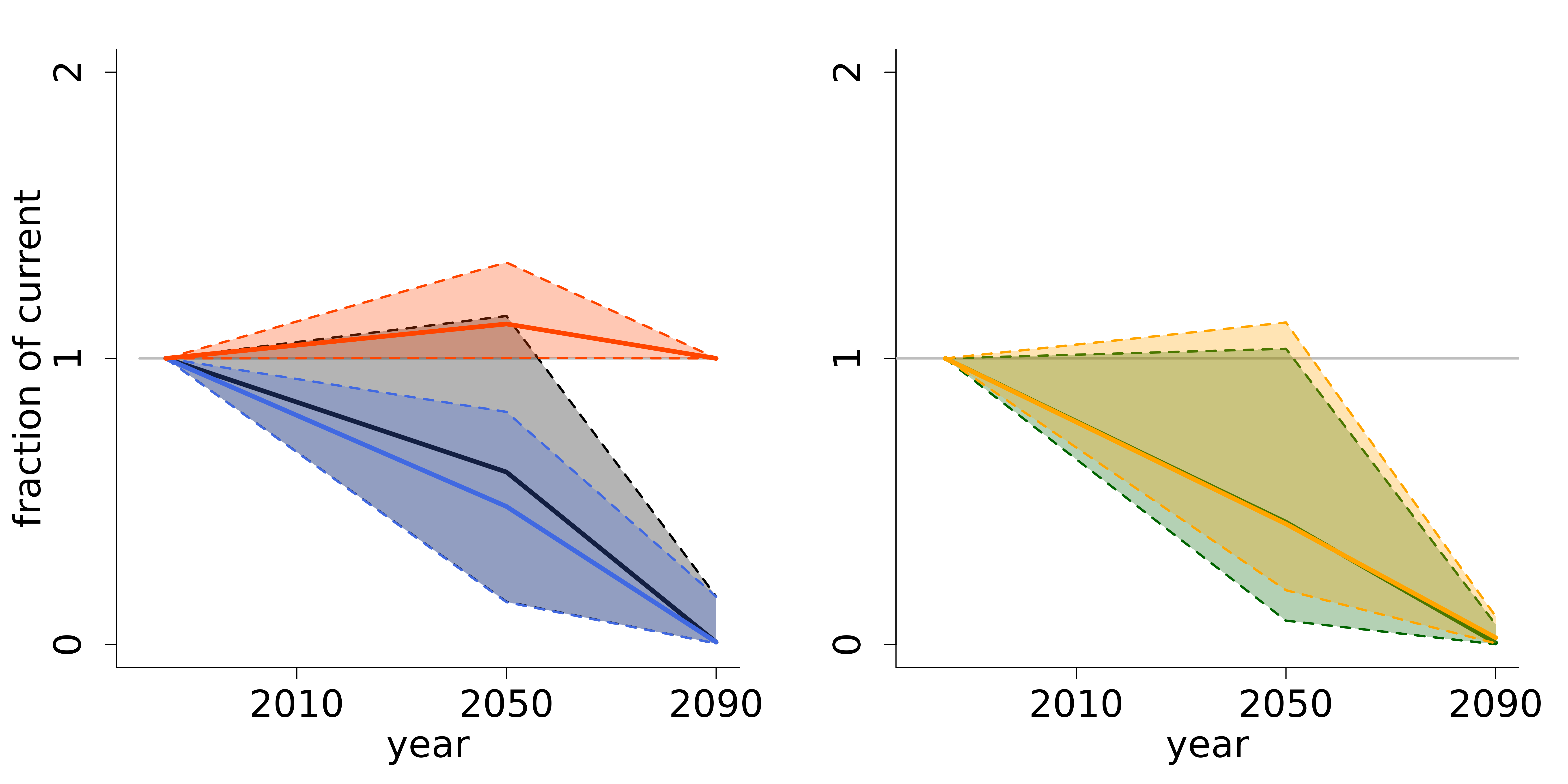

Supplement: S3 Appendix — (ZIP) [file pntd.0014030.s007.zip › Sup. Mat. 6-2 M-Z - Species Trends/Micrurus_spixii_CCTrends.png]

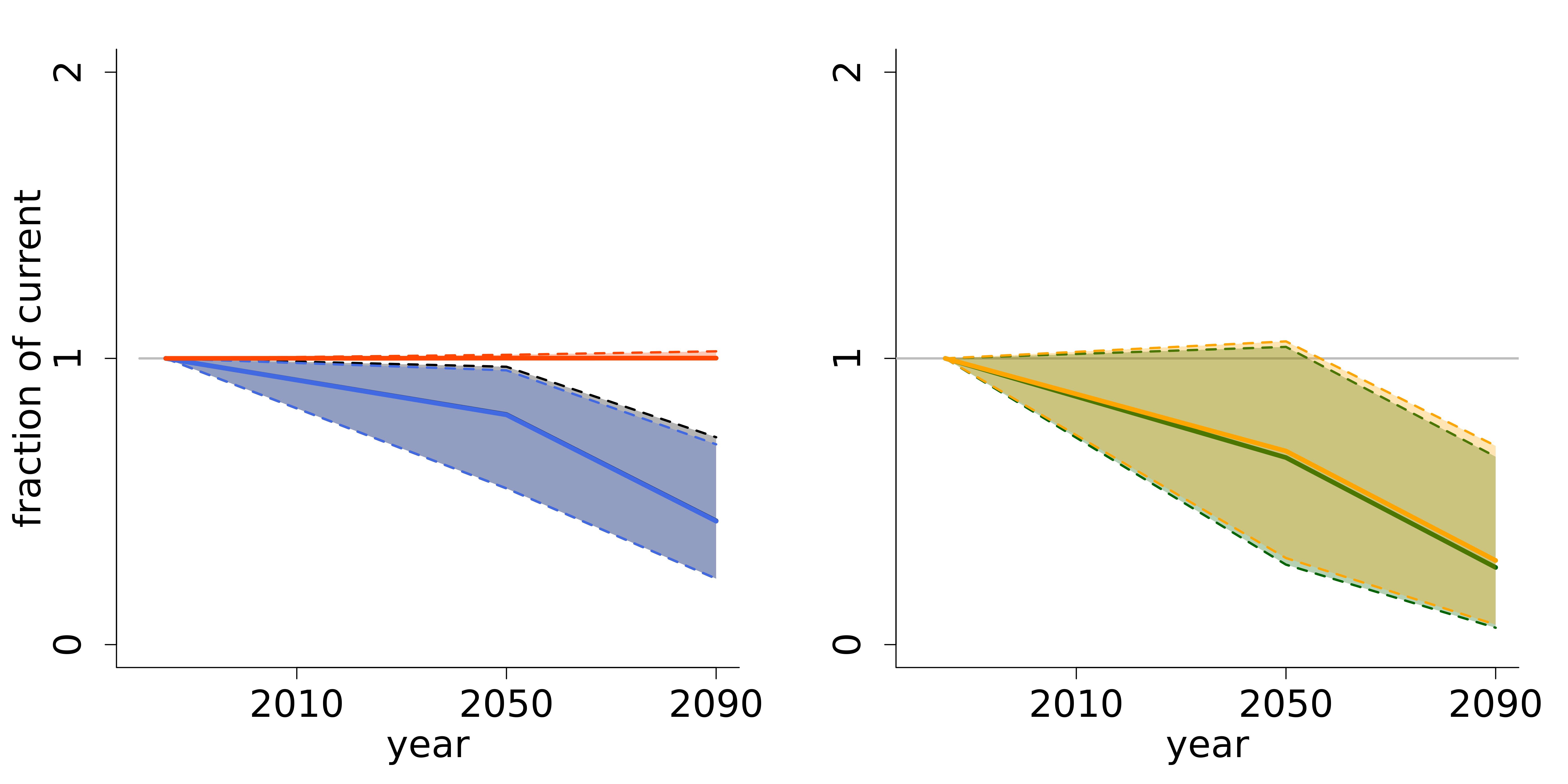

Supplement: S3 Appendix — (ZIP) [file pntd.0014030.s007.zip › Sup. Mat. 6-2 M-Z - Species Trends/Micrurus_spurrelli_CCTrends.png]

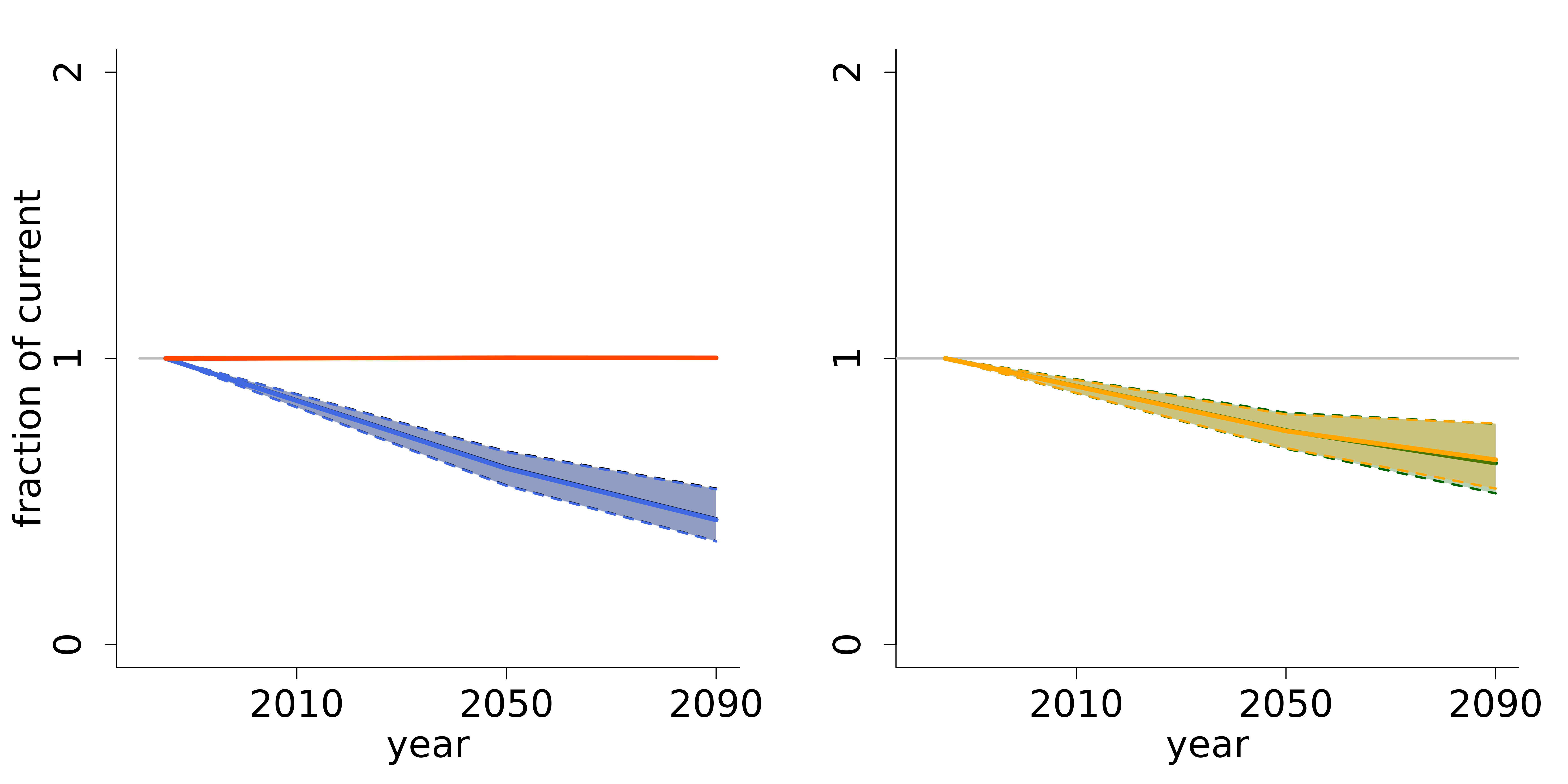

Supplement: S3 Appendix — (ZIP) [file pntd.0014030.s007.zip › Sup. Mat. 6-2 M-Z - Species Trends/Micrurus_steindachneri_CCTrends.png]

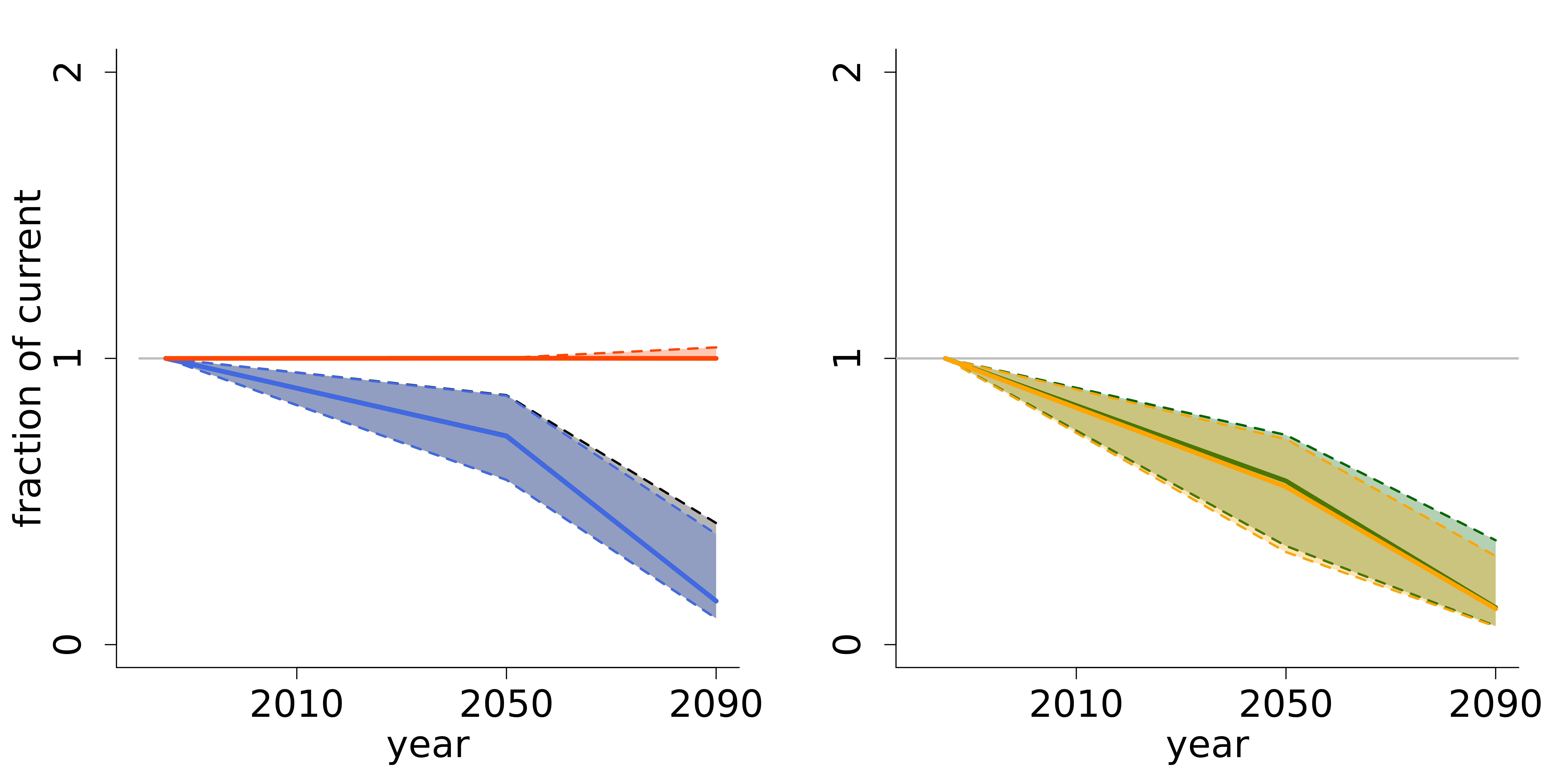

Supplement: S3 Appendix — (ZIP) [file pntd.0014030.s007.zip › Sup. Mat. 6-2 M-Z - Species Trends/Micrurus_stewarti_CCTrends.png]

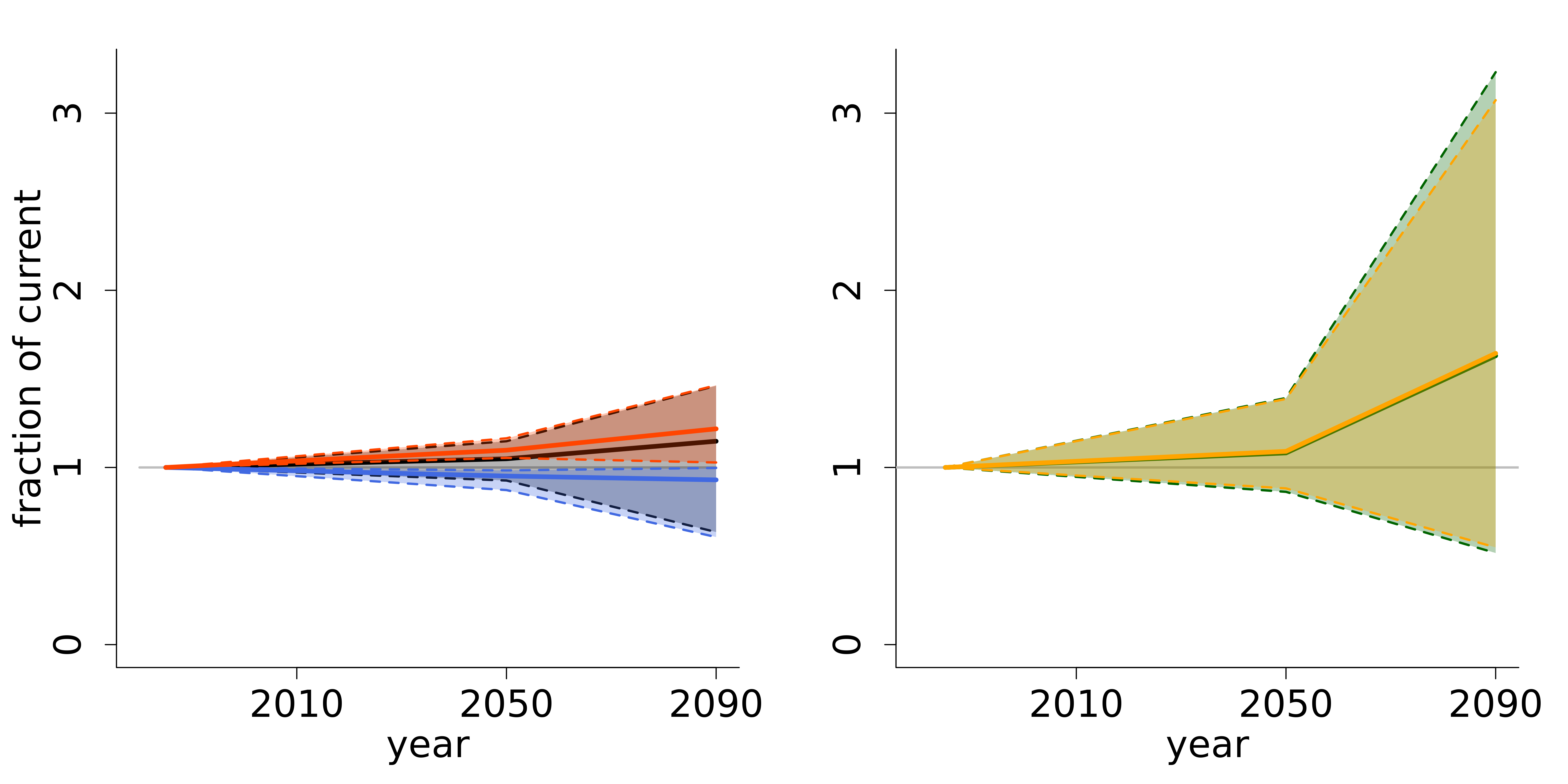

Supplement: S3 Appendix — (ZIP) [file pntd.0014030.s007.zip › Sup. Mat. 6-2 M-Z - Species Trends/Micrurus_stuarti_CCTrends.png]

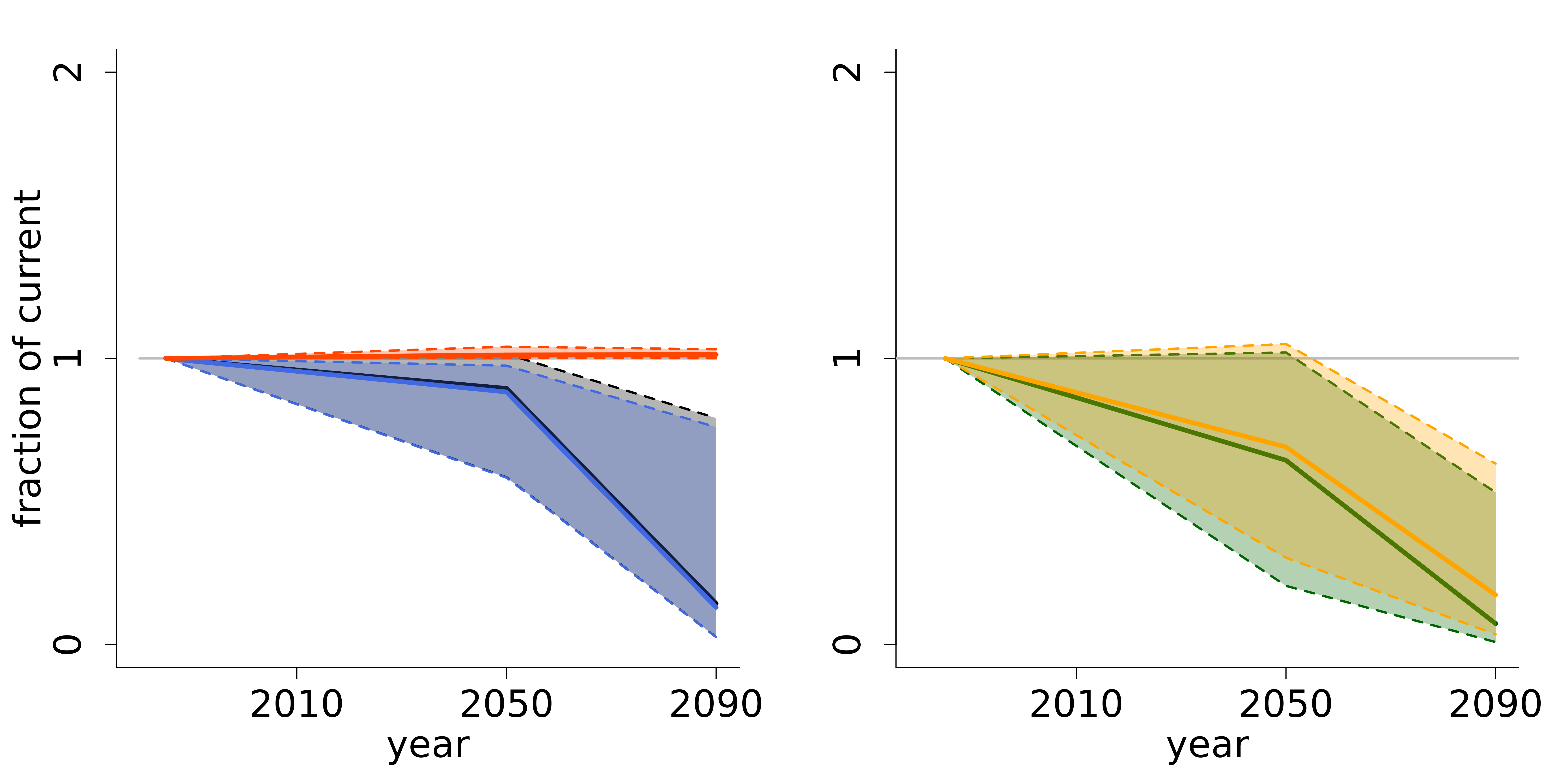

Supplement: S3 Appendix — (ZIP) [file pntd.0014030.s007.zip › Sup. Mat. 6-2 M-Z - Species Trends/Micrurus_surinamensis_CCTrends.png]

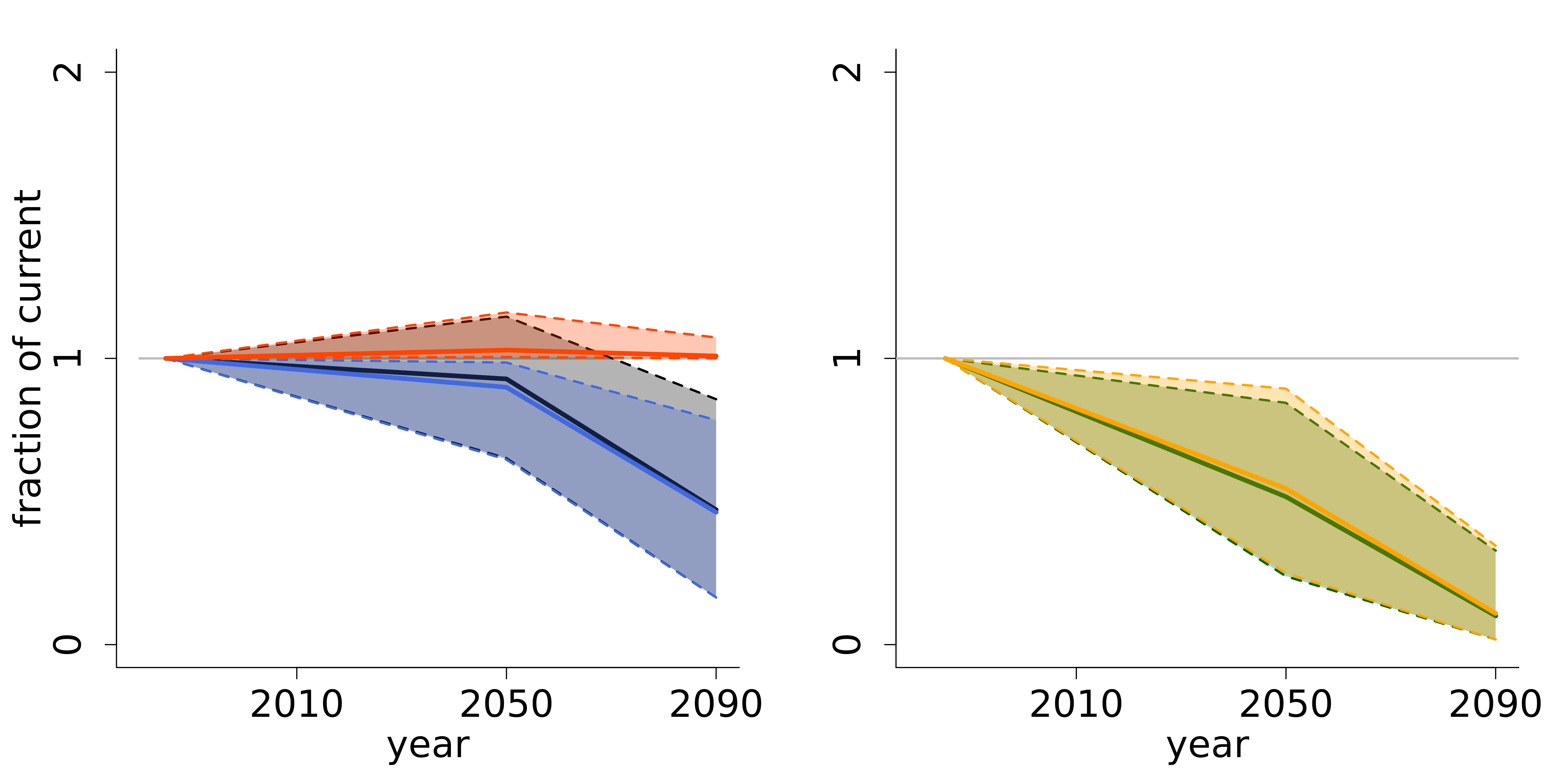

Supplement: S3 Appendix — (ZIP) [file pntd.0014030.s007.zip › Sup. Mat. 6-2 M-Z - Species Trends/Micrurus_tener_CCTrends.png]

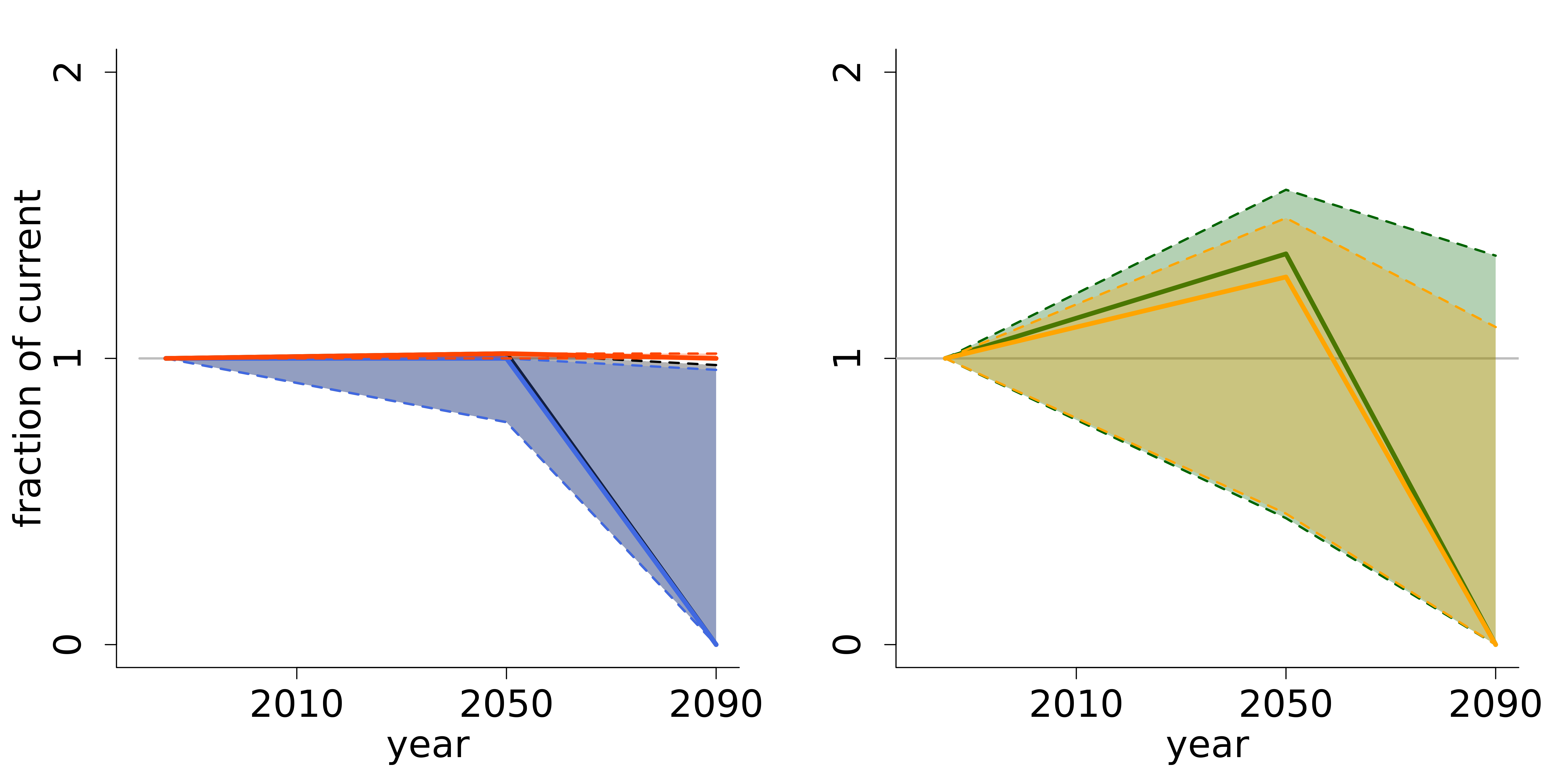

Supplement: S3 Appendix — (ZIP) [file pntd.0014030.s007.zip › Sup. Mat. 6-2 M-Z - Species Trends/Micrurus_tikuna_CCTrends.png]

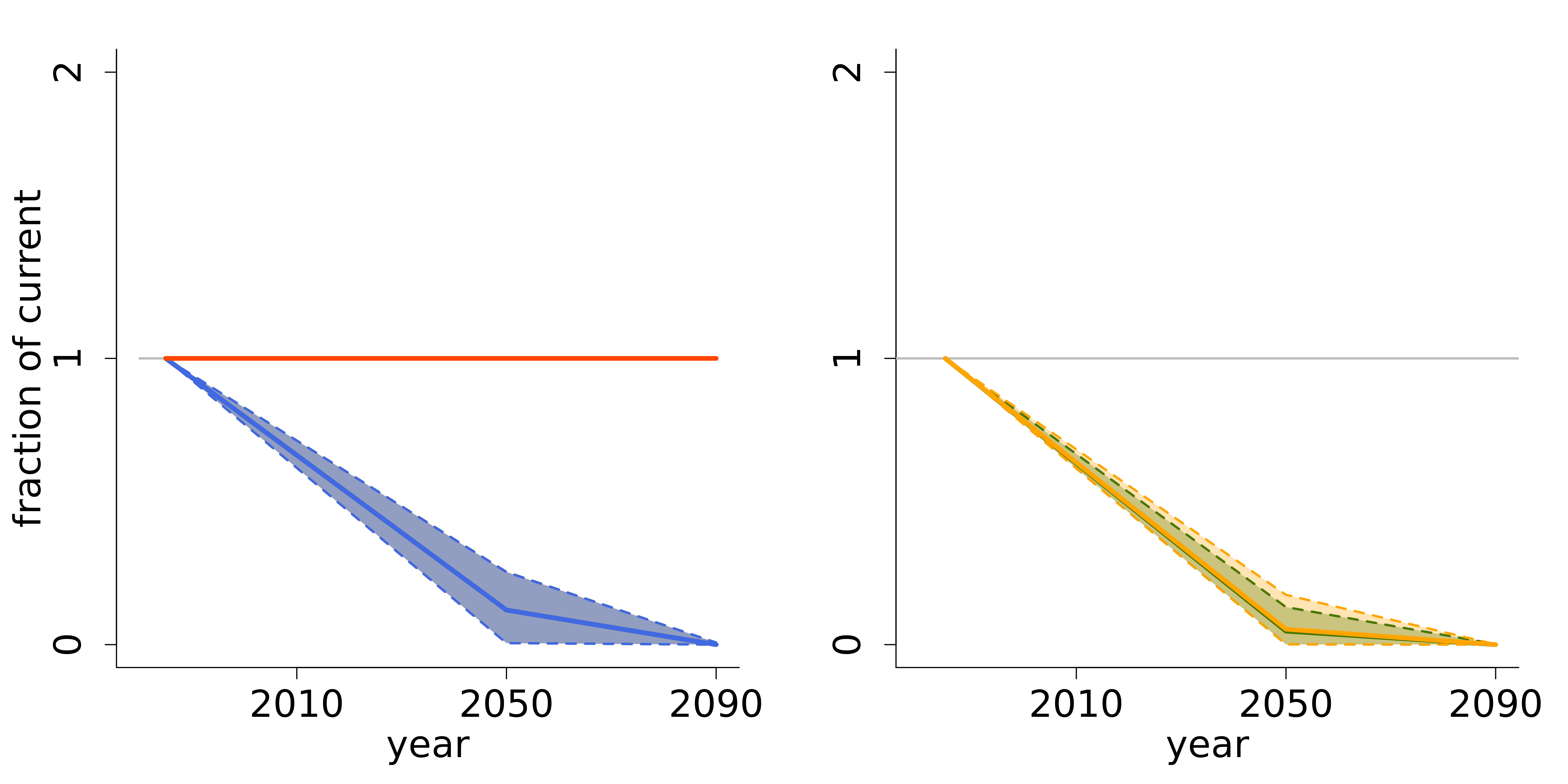

Supplement: S3 Appendix — (ZIP) [file pntd.0014030.s007.zip › Sup. Mat. 6-2 M-Z - Species Trends/Micrurus_tricolor_CCTrends.png]

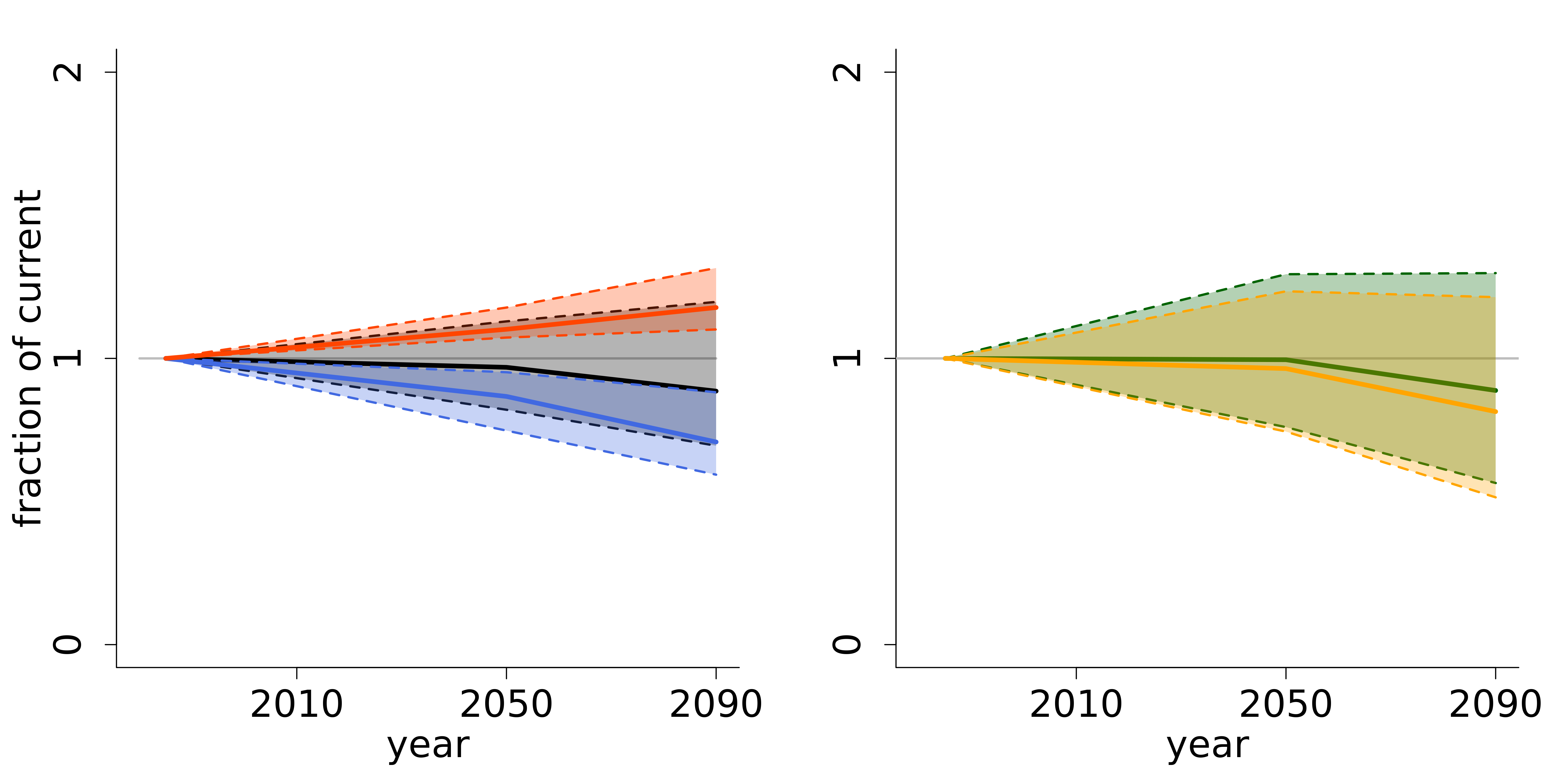

Supplement: S3 Appendix — (ZIP) [file pntd.0014030.s007.zip › Sup. Mat. 6-2 M-Z - Species Trends/Micrurus_tschudii_CCTrends.png]

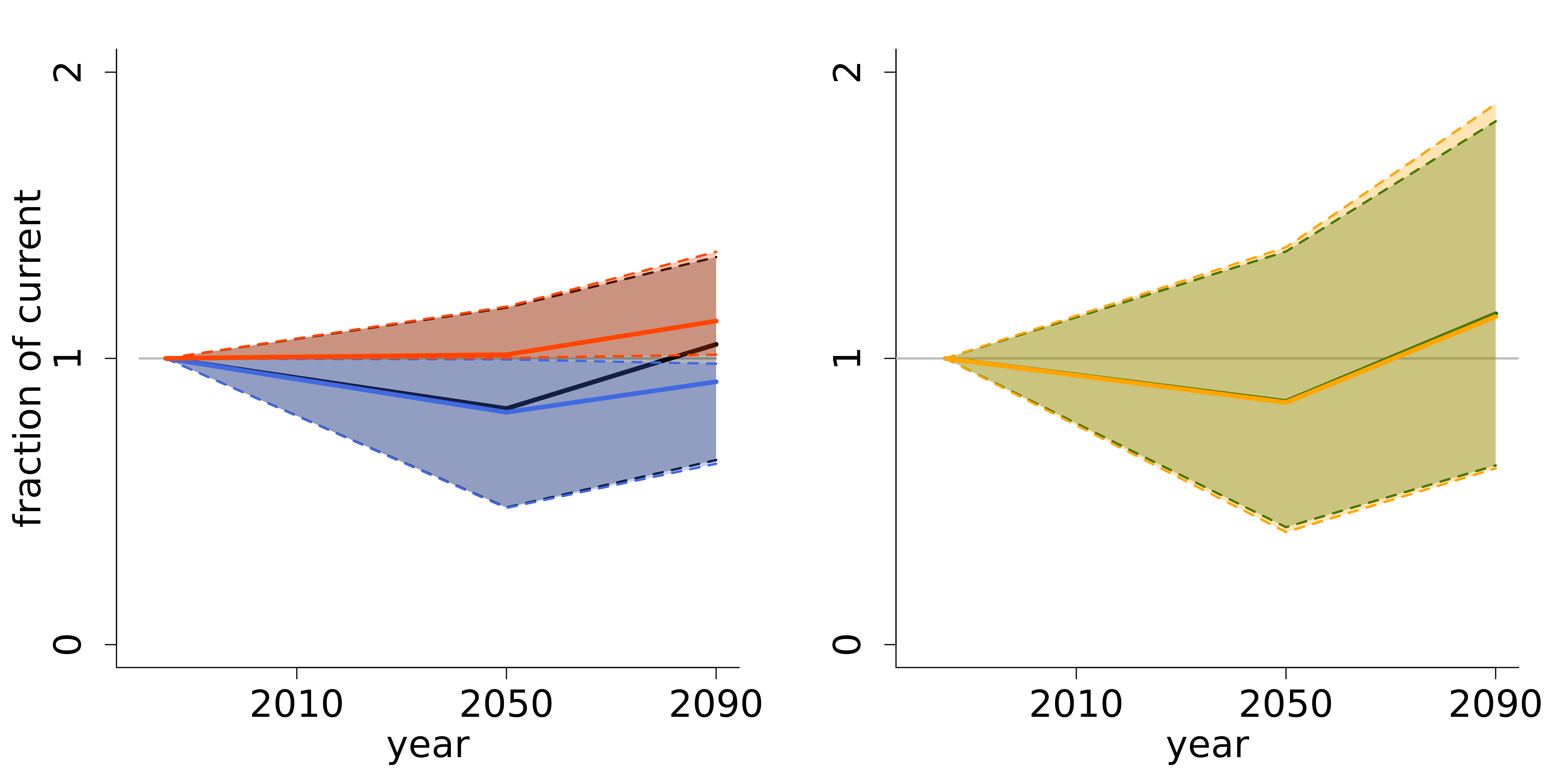

Supplement: S3 Appendix — (ZIP) [file pntd.0014030.s007.zip › Sup. Mat. 6-2 M-Z - Species Trends/Mixcoatlus_barbouri_CCTrends.png]

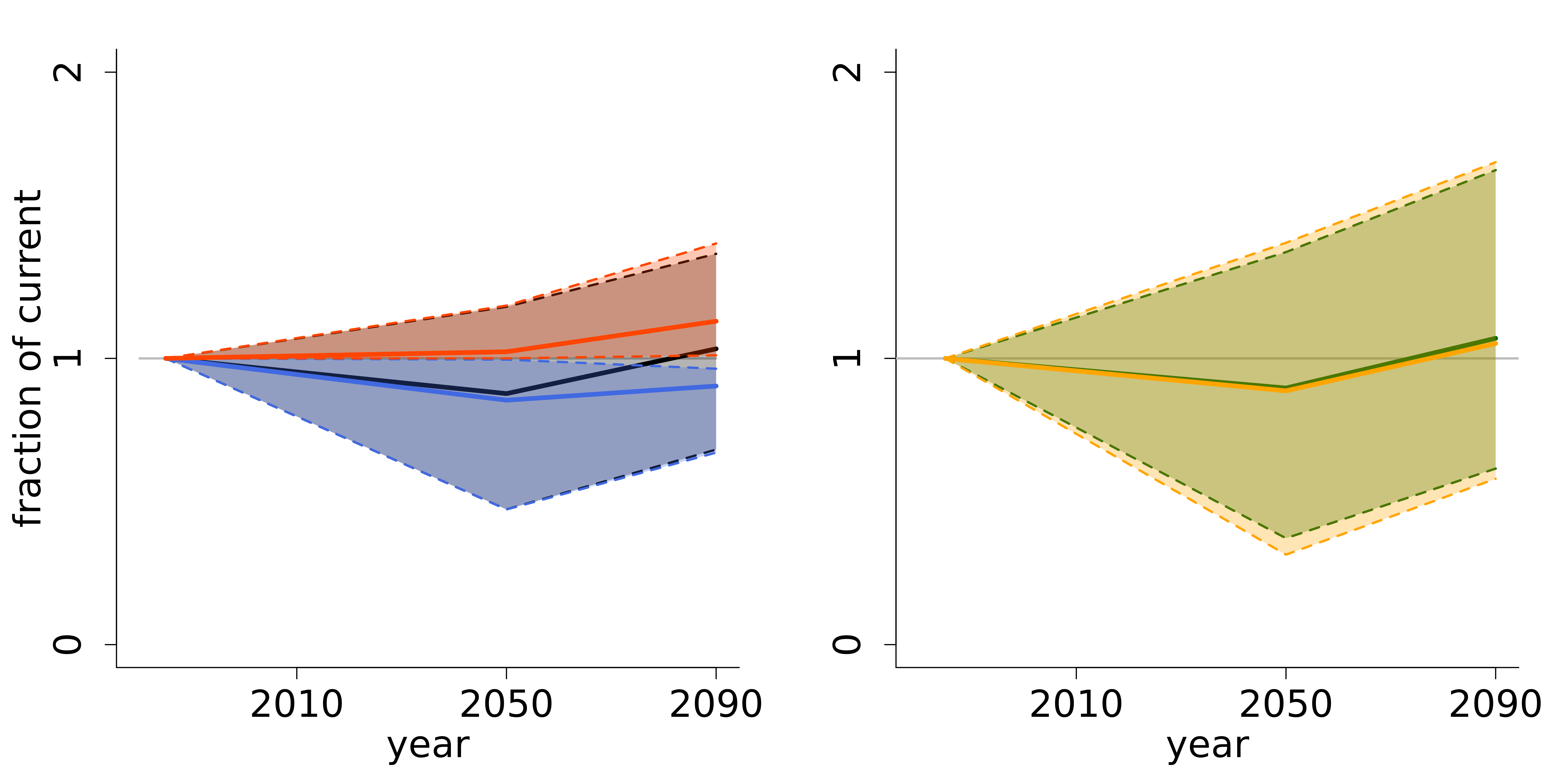

Supplement: S3 Appendix — (ZIP) [file pntd.0014030.s007.zip › Sup. Mat. 6-2 M-Z - Species Trends/Mixcoatlus_browni_CCTrends.png]

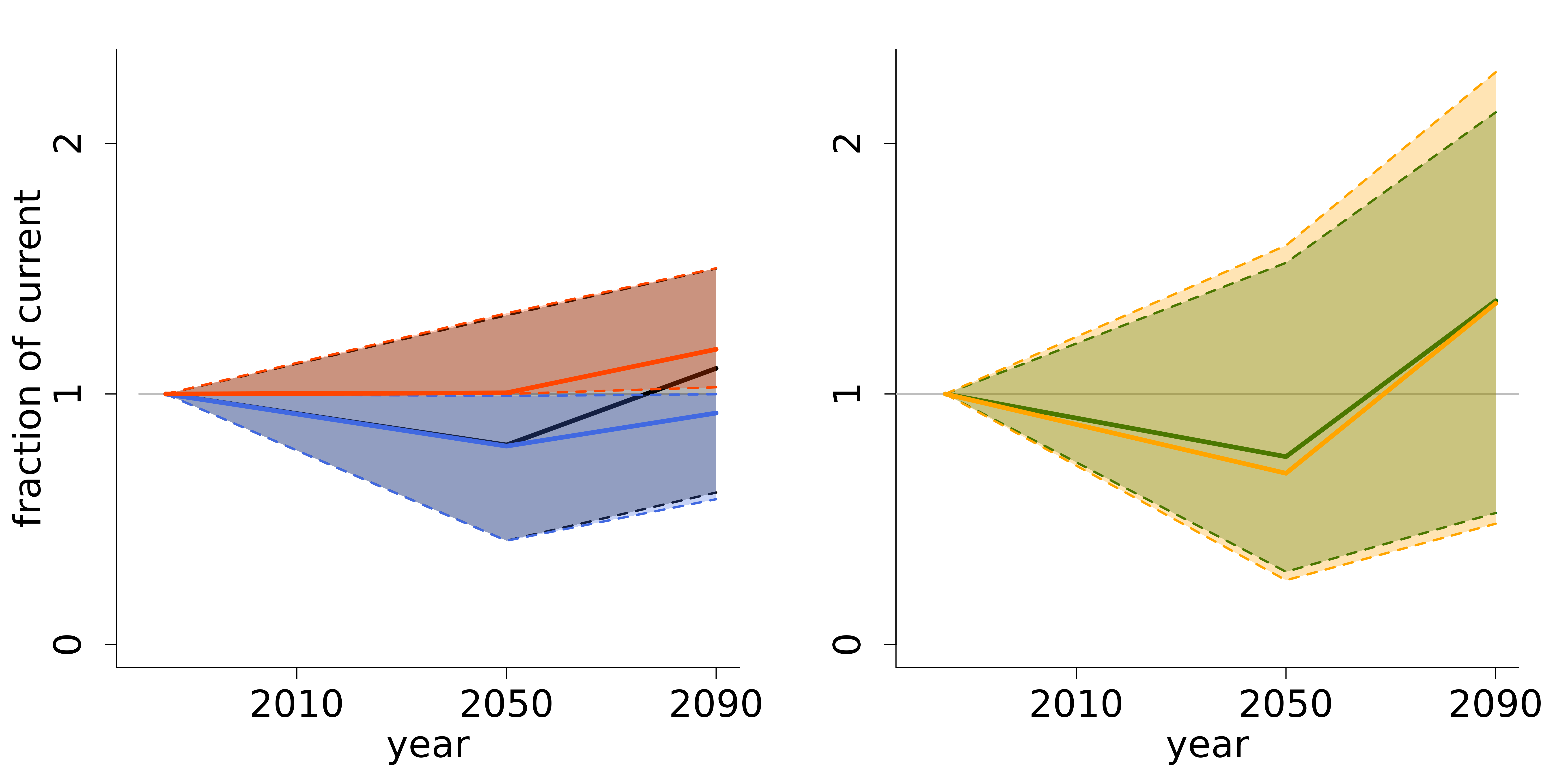

Supplement: S3 Appendix — (ZIP) [file pntd.0014030.s007.zip › Sup. Mat. 6-2 M-Z - Species Trends/Mixcoatlus_melanurus_CCTrends.png]

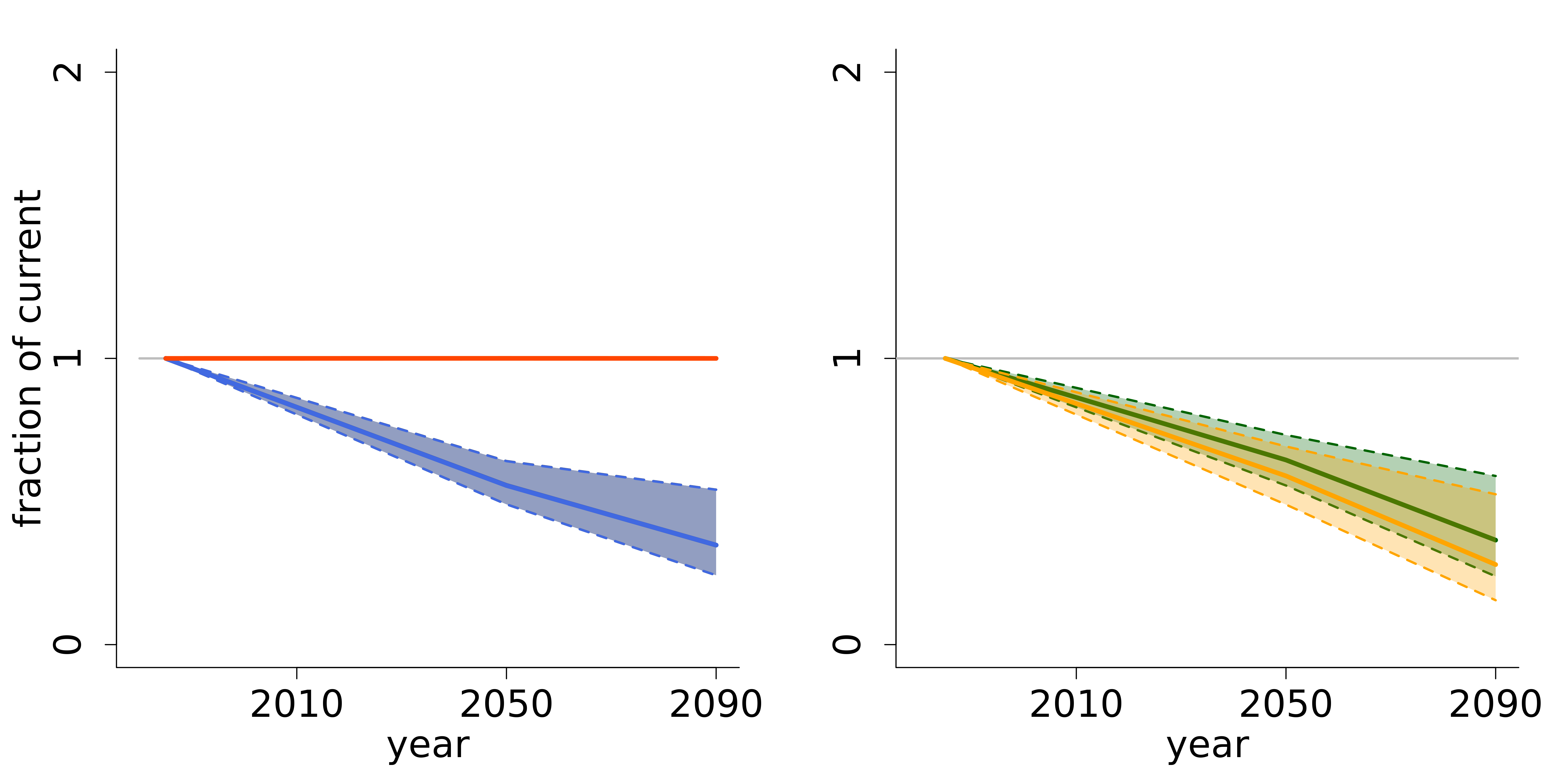

Supplement: S3 Appendix — (ZIP) [file pntd.0014030.s007.zip › Sup. Mat. 6-2 M-Z - Species Trends/Montivipera_bornmuelleri_CCTrends.png]

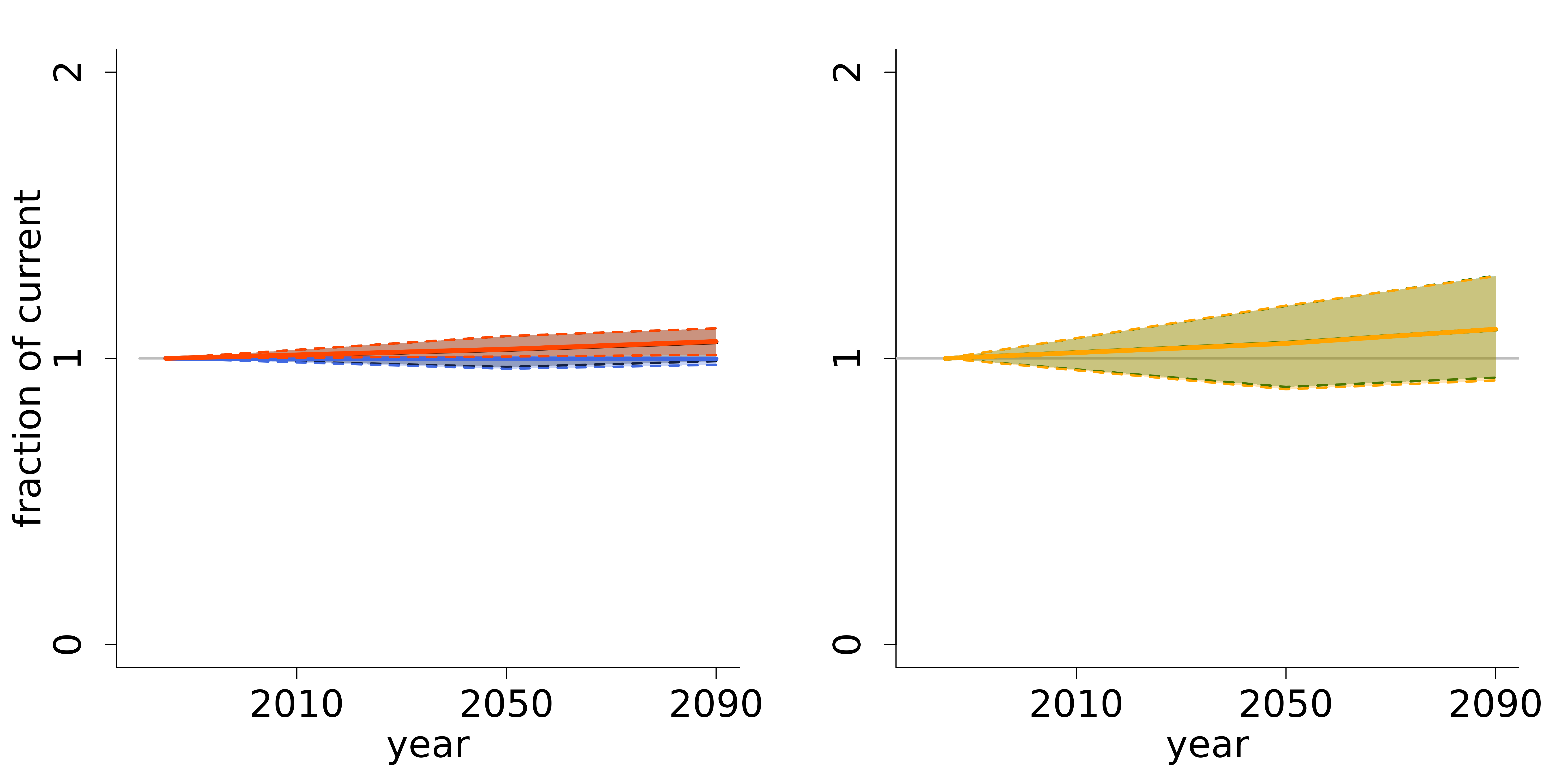

Supplement: S3 Appendix — (ZIP) [file pntd.0014030.s007.zip › Sup. Mat. 6-2 M-Z - Species Trends/Montivipera_bulgardaghica_CCTrends.png]

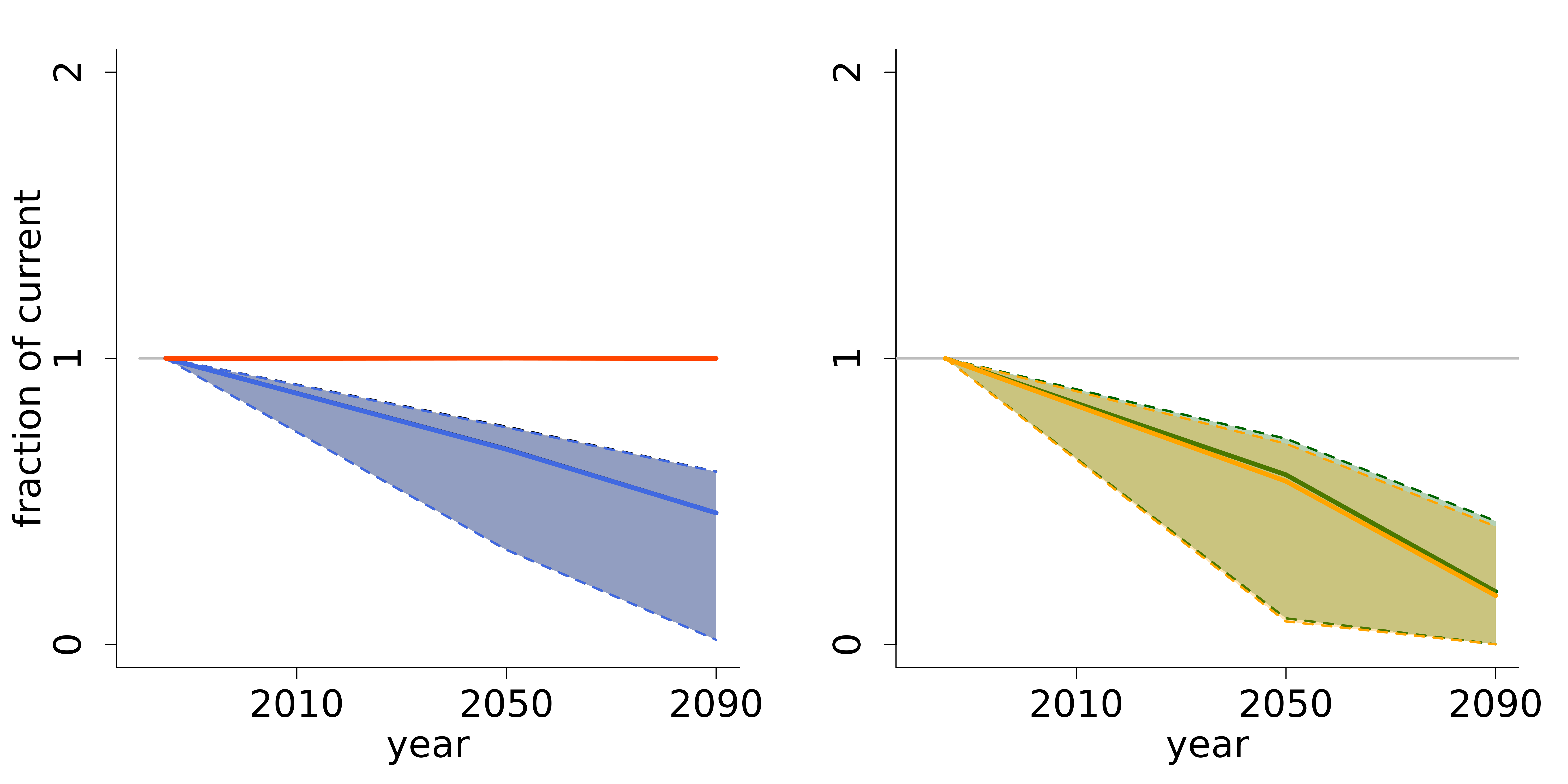

Supplement: S3 Appendix — (ZIP) [file pntd.0014030.s007.zip › Sup. Mat. 6-2 M-Z - Species Trends/Montivipera_kuhrangica_CCTrends.png]

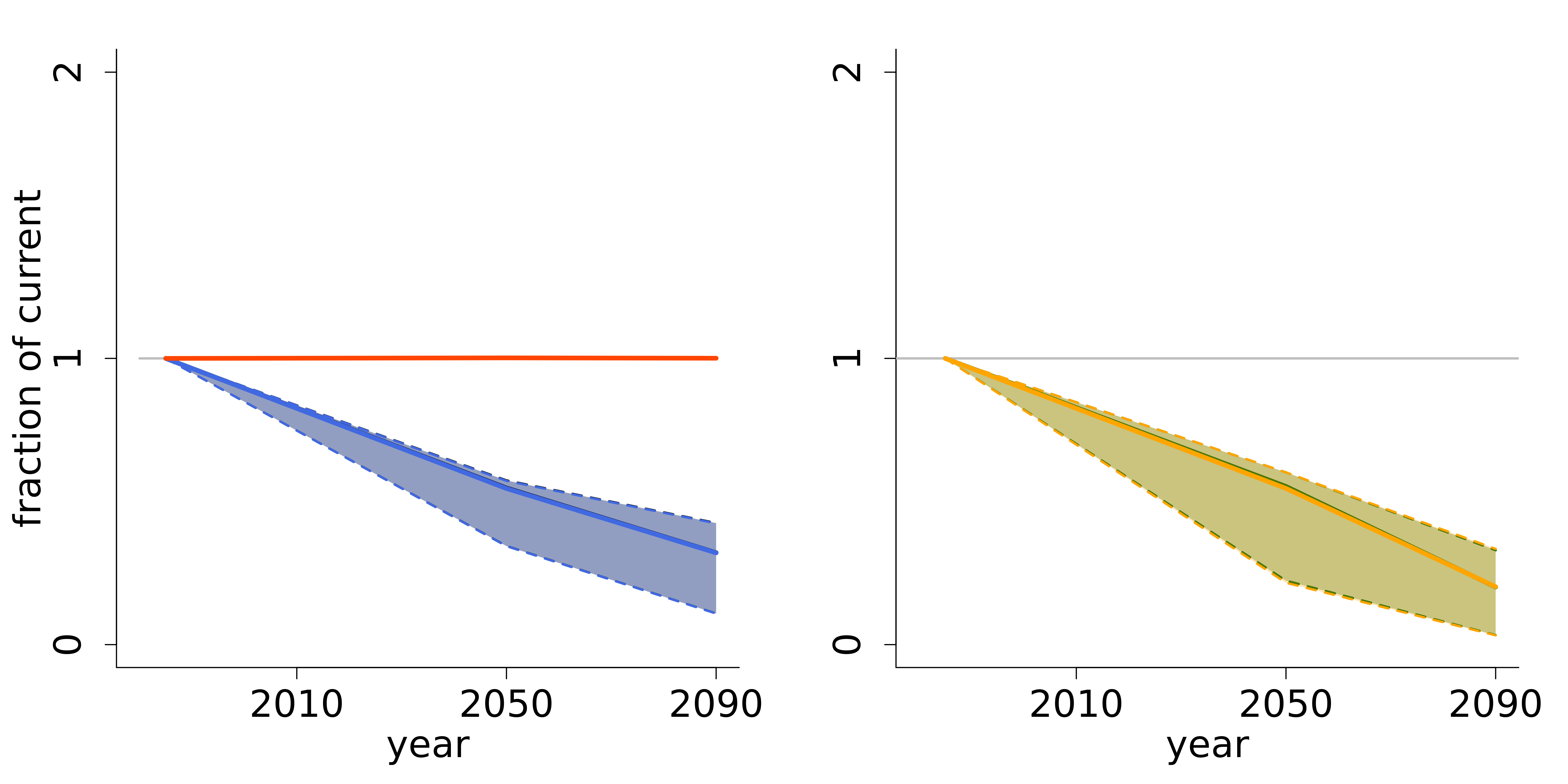

Supplement: S3 Appendix — (ZIP) [file pntd.0014030.s007.zip › Sup. Mat. 6-2 M-Z - Species Trends/Montivipera_latifii_CCTrends.png]

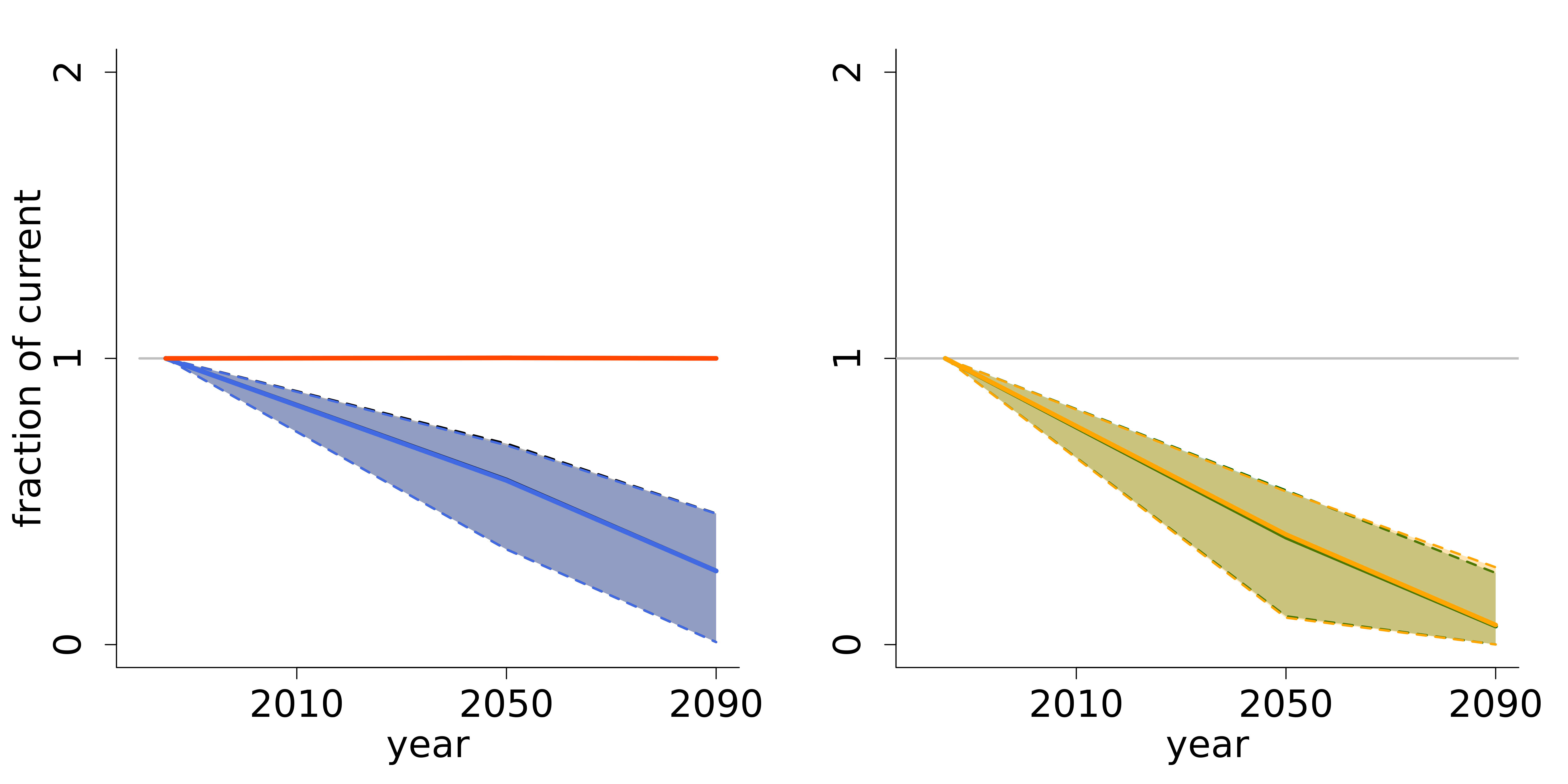

Supplement: S3 Appendix — (ZIP) [file pntd.0014030.s007.zip › Sup. Mat. 6-2 M-Z - Species Trends/Montivipera_raddei_CCTrends.png]

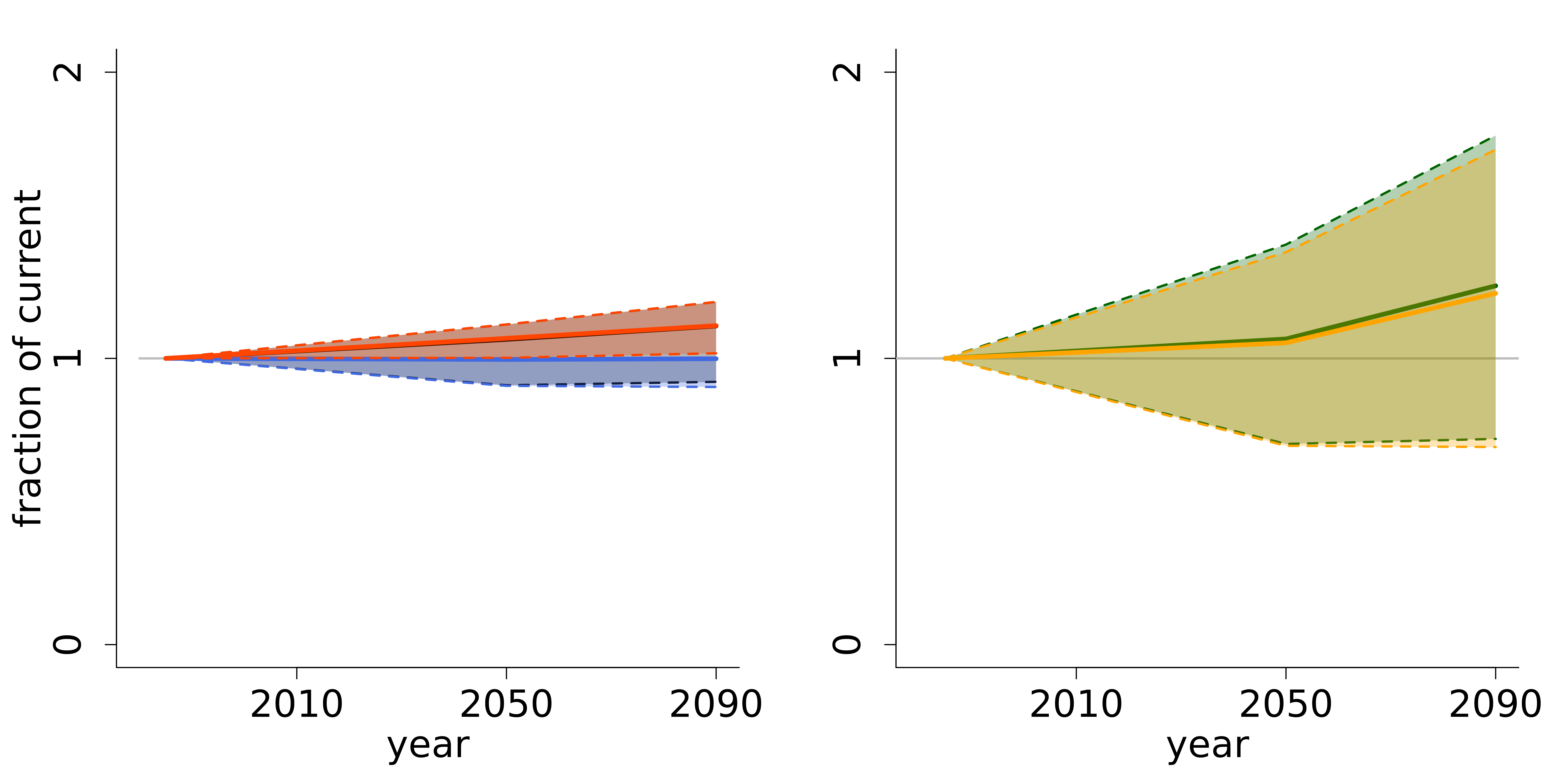

Supplement: S3 Appendix — (ZIP) [file pntd.0014030.s007.zip › Sup. Mat. 6-2 M-Z - Species Trends/Montivipera_wagneri_CCTrends.png]

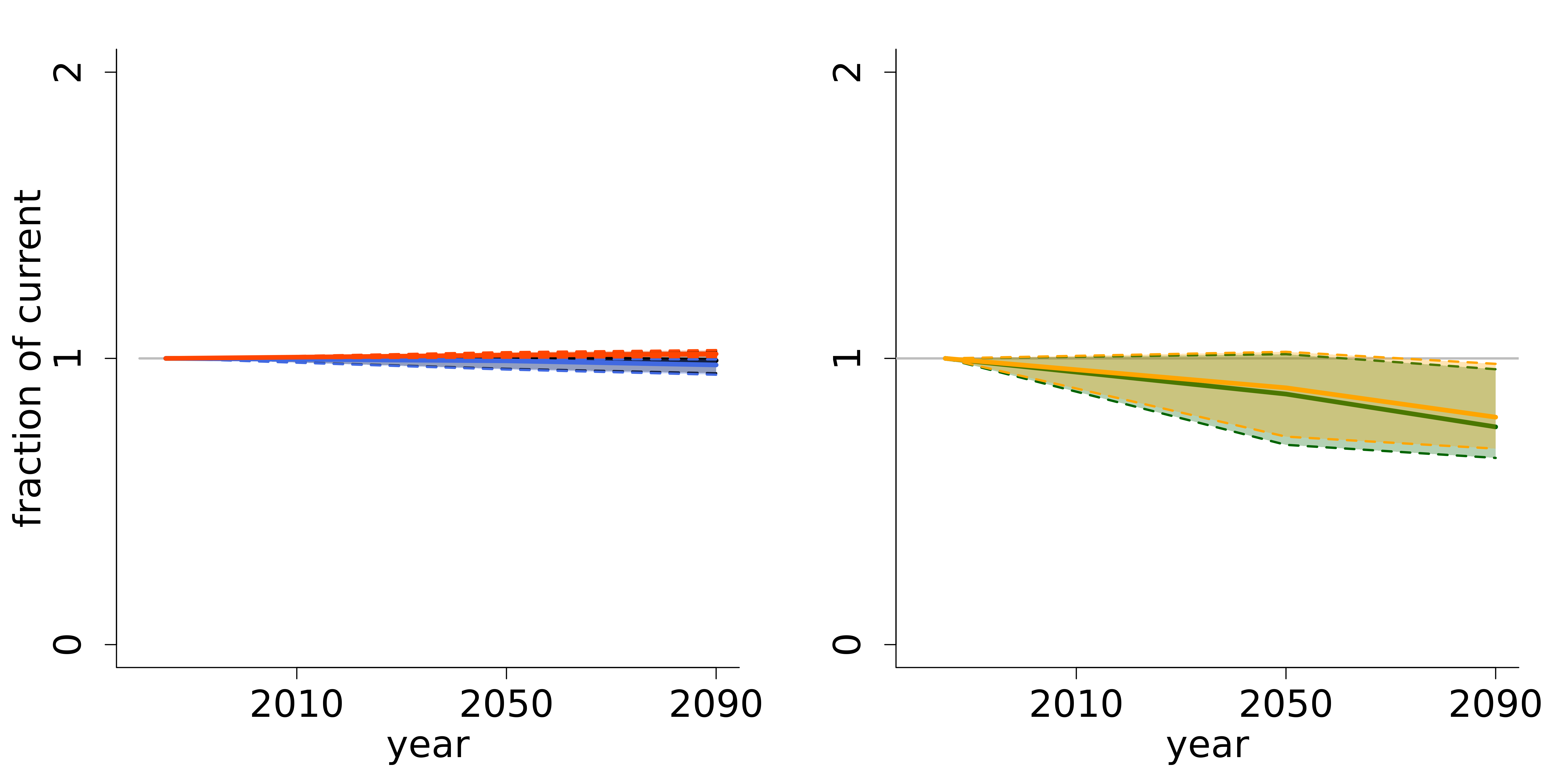

Supplement: S3 Appendix — (ZIP) [file pntd.0014030.s007.zip › Sup. Mat. 6-2 M-Z - Species Trends/Montivipera_xanthina_CCTrends.png]

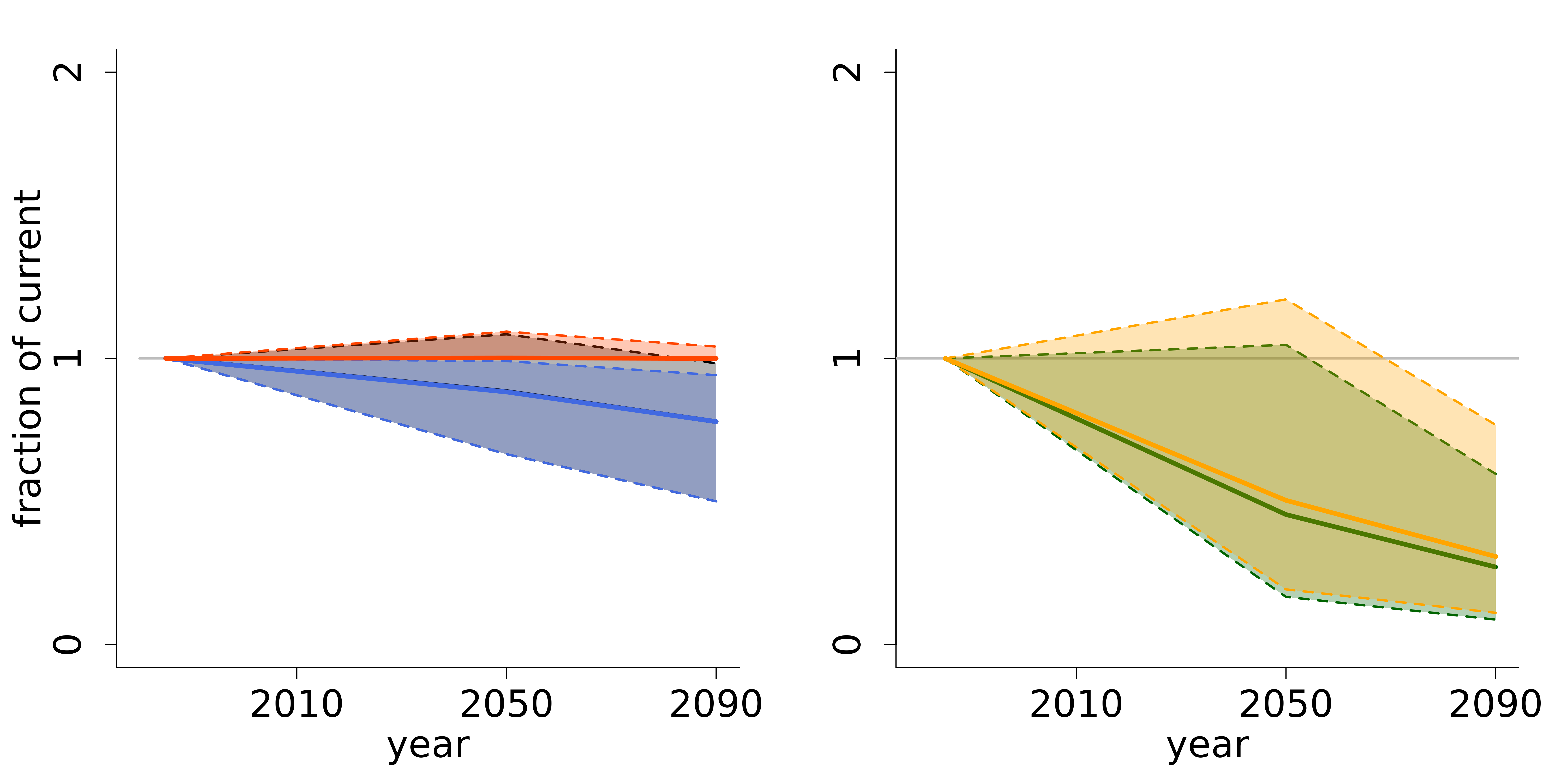

Supplement: S3 Appendix — (ZIP) [file pntd.0014030.s007.zip › Sup. Mat. 6-2 M-Z - Species Trends/Naja_anchietae_CCTrends.png]

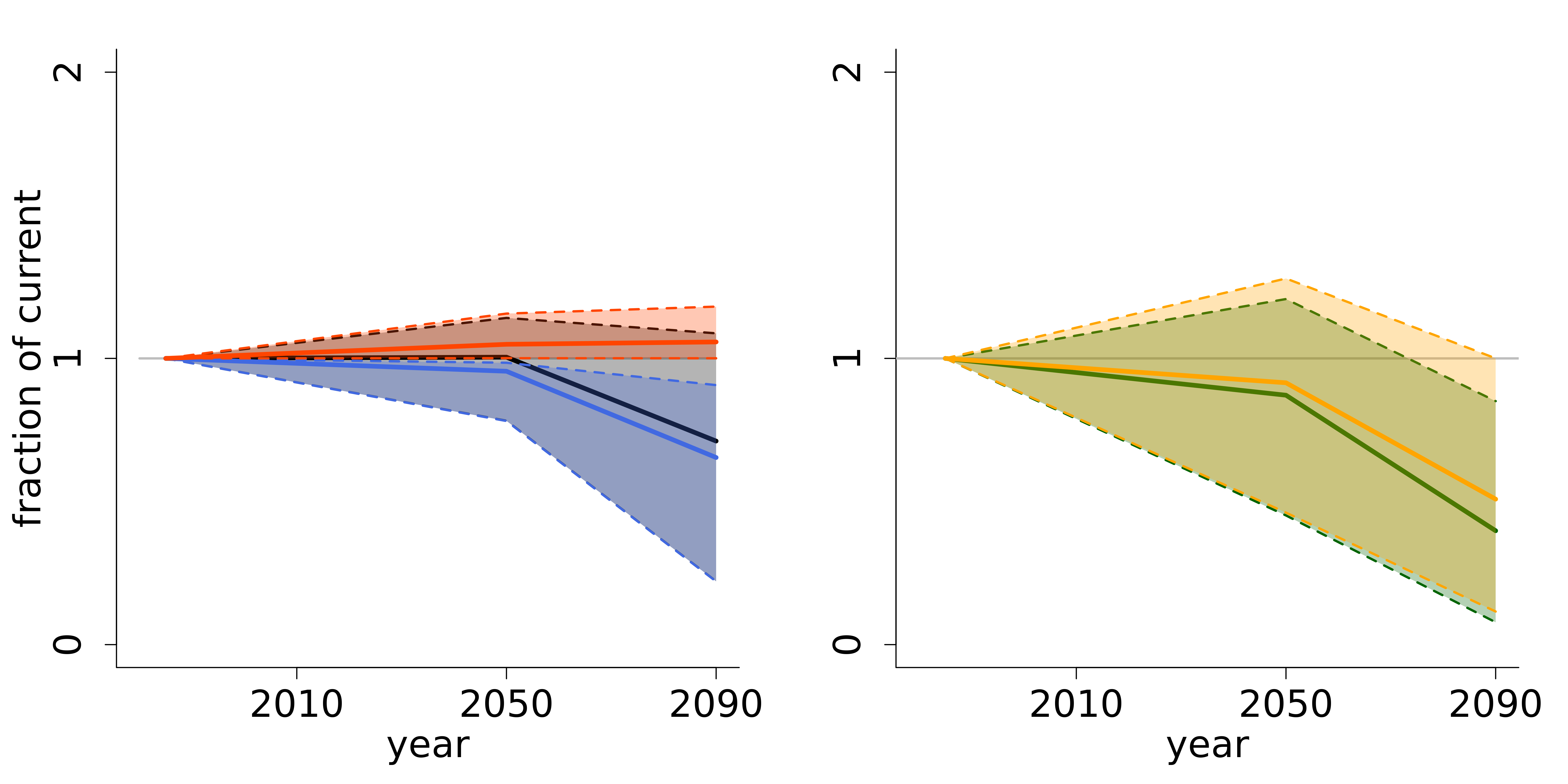

Supplement: S3 Appendix — (ZIP) [file pntd.0014030.s007.zip › Sup. Mat. 6-2 M-Z - Species Trends/Naja_annulata_CCTrends.png]

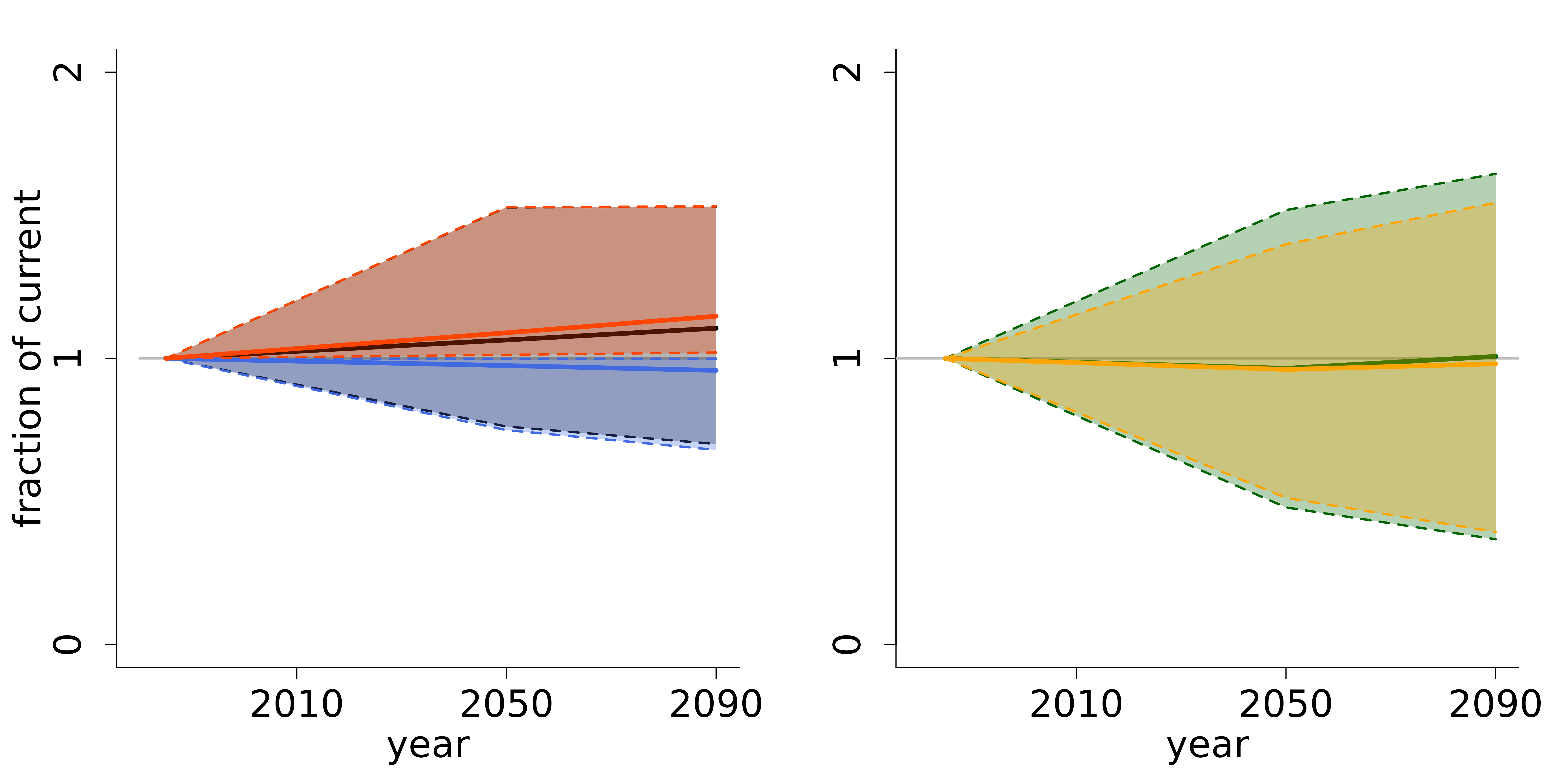

Supplement: S3 Appendix — (ZIP) [file pntd.0014030.s007.zip › Sup. Mat. 6-2 M-Z - Species Trends/Naja_annulifera_CCTrends.png]

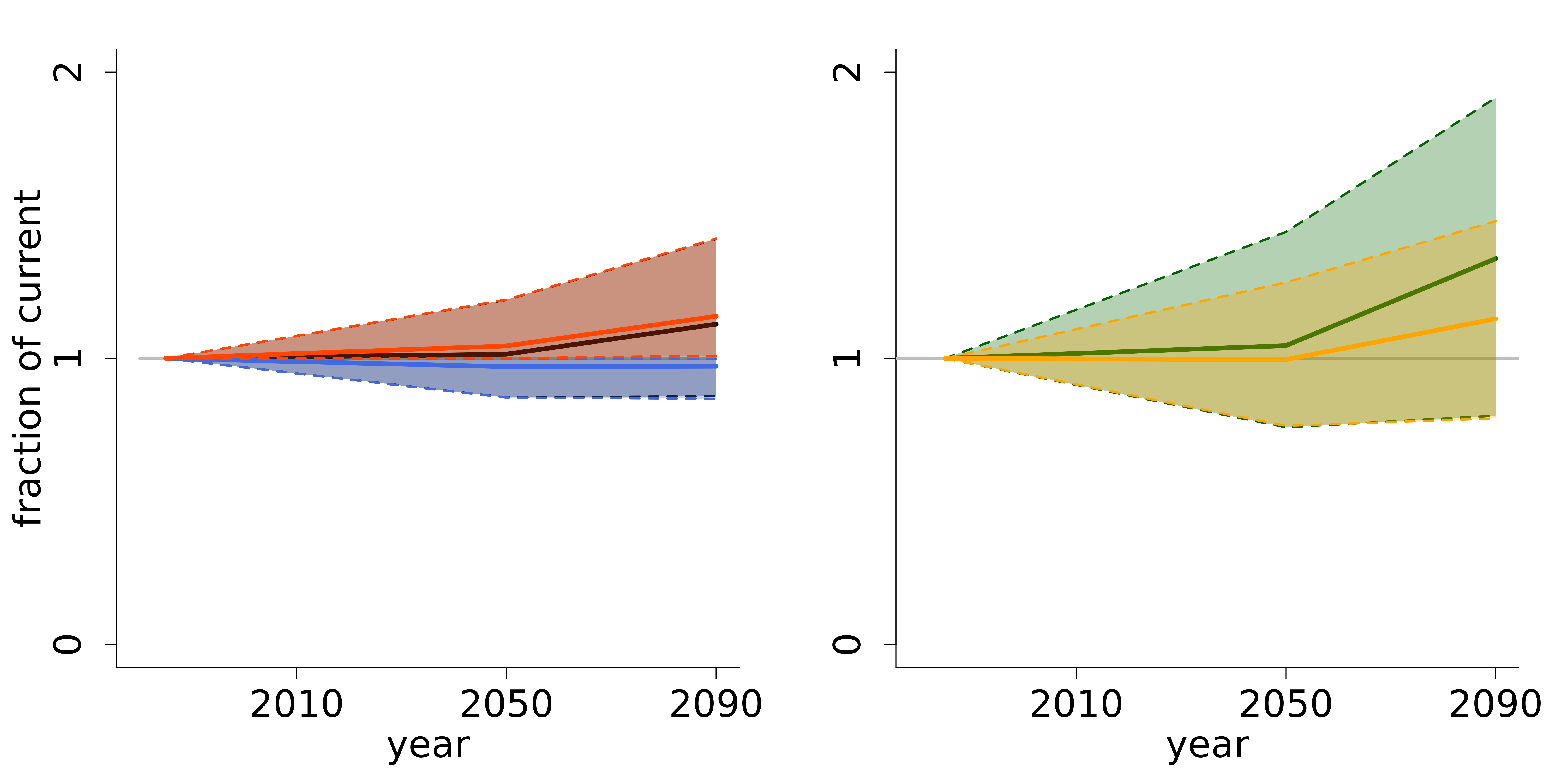

Supplement: S3 Appendix — (ZIP) [file pntd.0014030.s007.zip › Sup. Mat. 6-2 M-Z - Species Trends/Naja_arabica_CCTrends.png]

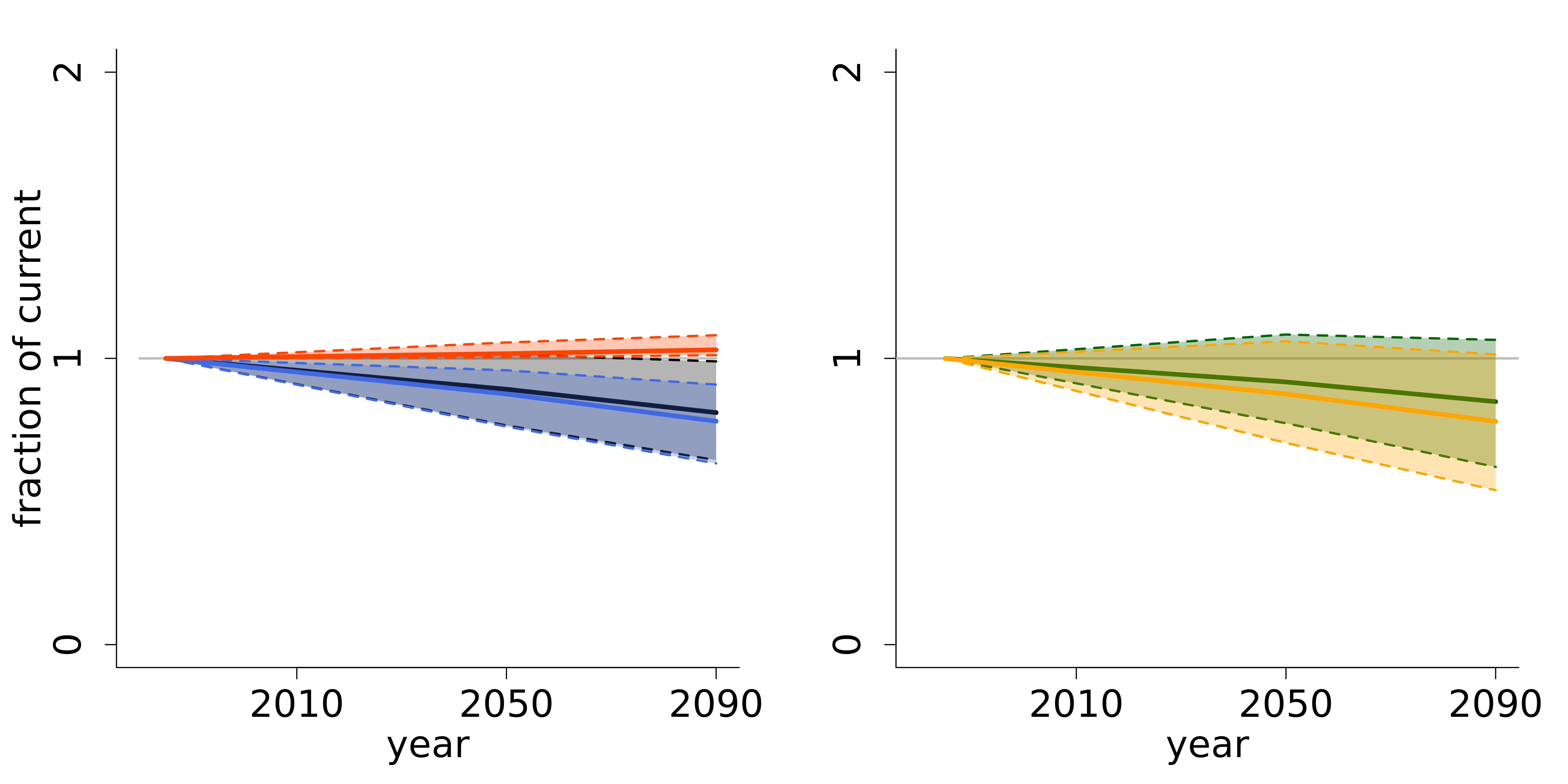

Supplement: S3 Appendix — (ZIP) [file pntd.0014030.s007.zip › Sup. Mat. 6-2 M-Z - Species Trends/Naja_ashei_CCTrends.png]

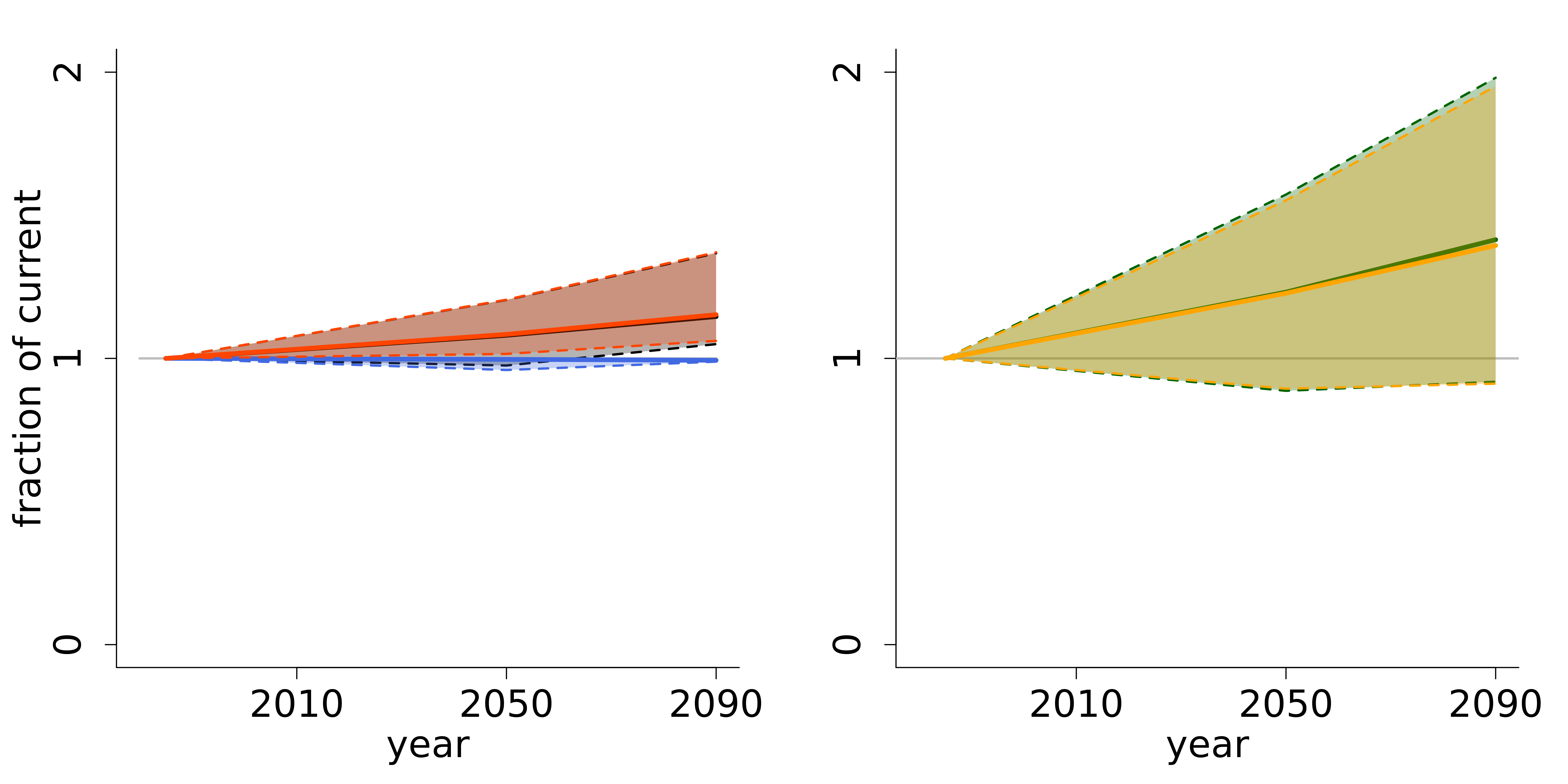

Supplement: S3 Appendix — (ZIP) [file pntd.0014030.s007.zip › Sup. Mat. 6-2 M-Z - Species Trends/Naja_atra_CCTrends.png]

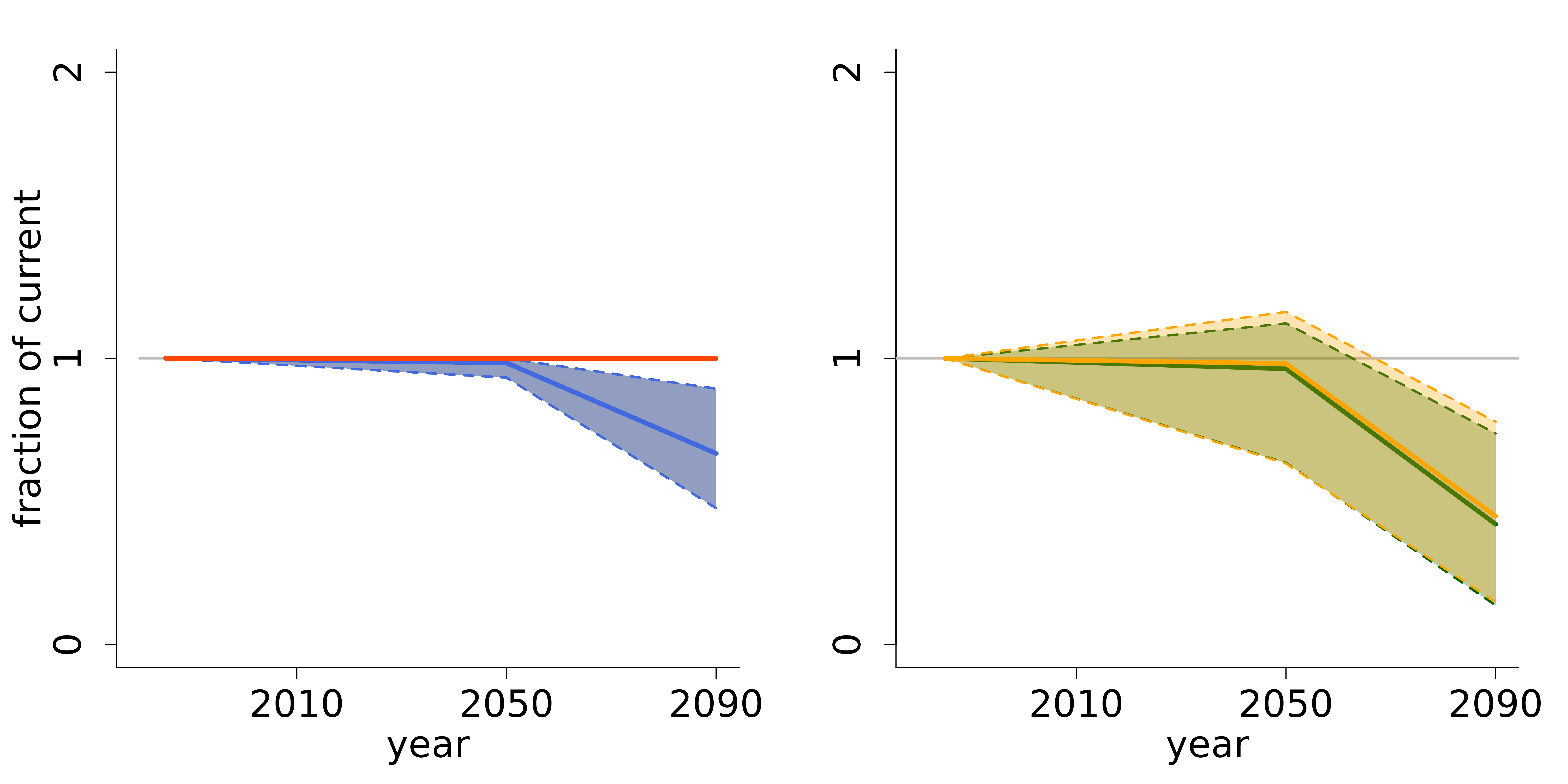

Supplement: S3 Appendix — (ZIP) [file pntd.0014030.s007.zip › Sup. Mat. 6-2 M-Z - Species Trends/Naja_christyi_CCTrends.png]
