# Supplementary material for: Climate change induced complex shifts in snake distributions expose people to snakebite and threaten biodiversity
Source: PLoS Negl Trop Dis. 2026 May 21;20(5):e0014030. doi: 10.1371/journal.pntd.0014030 (PMC13193456; doi:10.1371/journal.pntd.0014030)
Supplement: Attachment — Submitted filename: PLOS NTD Rebuttal letter - final.docx [file pntd.0014030.s010.docx]

Date: 4^th^ of October 2025

**Dr. Anna Pintor**

***Research Scientist***

Department for Neglected Tropical Diseases
World Health Organization
Geneva, Switzerland/ Cairns, Australia

P:      +61 450 452 605
E:       [annafvp@gmail.com](mailto:anna.pintor1@jcu.edu.au)

Adly M.M. Abd-Alla, Prof asso

Section Editor

PLOS Neglected Tropical Disease

**Dear Dr. Adly M.M. Abd-Alla, Prof asso.,**

**Ref:** PNTD-D-25-00575

We thank you for your previous consideration of our manuscript “*Climate change induced complex shifts in snake distributions expose people to snakebite and threaten biodiversity*” for publication in the journal “PLOS Neglected Tropical Diseases”. Please find attached our revised version.

We have reviewed the feedback from reviewers and editors and addressed each comment in detail in the attached response (red highlighted text). We thank the reviewers for their constructive criticisms and believe we have addressed all suggestions to their satisfaction and to the best of our ability. Where changes were not possible, we outline in detail our rational. Notably, some of the suggestions made by reviewer 1 were, unfortunately, outside of the scope if our already comprehensive study. All suggested editorial changes have been made, including establishment of a data repository at <https://doi.org/10.7910/DVN/QVWB4E>, within the Harvard Dataverse.

Our revised manuscript complies with all legal and ethical standards required by your journal. We, the authors, declare that there is no conflict of interest with our institutions, suggested reviewers, funding providers or any other persons or institutions.

Sincerely,

Anna Pintor (on behalf of all authors)

**Response to reviewers:**

Please note that all line references refer to the tracked changes version of the manuscript.

**Reviewer #1:**

As a positive aspect, the authors analyze a large number of species. I consider it important to distinguish between Type I and Type II species in the analyses. It is well known that Type II species have the potential to cause severe envenomations but rarely do so. How many of the species included are Type I and how many are Type II? I raise this point because a high proportion of Type II species may lead to an overestimation of the problem in certain regions. If most of the species included in the study are Type II, the conclusions may not be directly related to actual snakebite incidents.

The author misunderstands. Species are not classified as either category 1 or 2. One species can have different classifications in different countries such that it might be category 1 in some countries and category 2 in others; Echis leucogaster for example. Also, class 2 designation may be because a snake species is rare, but envenomation is very serious, or common but with milder consequences of envenomation, or has a highly patchy distribution. We are well aware of the potential for overestimation, but have chosen to take an inclusive approach at this stage and specificallydiscuss our reasoning in the methods section (L173-177: “Both, category 1 and 2 species were included. Because some species are considered different categories in different countries, it is impossible to separate the two groups. Many category 2 species may have significant impacts on people but are too poorly studied to quantify bite incidence. Including these species provides valuable information on potential underreporting or to identify knowledge gaps”). To address the issue formally will require a substantial follow-on study that is outwith the scope of the current paper. We have added a section tp the discussion in L616-622 to explicitly explain this limitation: “Some of these trait differences are reflected in category 1 versus 2 listings for each species. However, each species category can vary among countries and often reflect lack of knowledge on their impact or differences in abundance or percent of the country covered by their distribution rather than true differences in their impact on people where they do occur. Using these categories to weight species impact is, therefore, not necessarily appropriate and much more research is needed to make it possible to include snake traits in risk analyses.”

Another issue is that the authors state they included several species, but in reality, many of them were modelled as a single entity (modelling units – MUs), and only after modelling the species complexes together did they separate the species (lines 268–279). This approach assumes that all lineages within a species complex share the same climatic niche. However, this is not necessarily true, especially in cases where the complex is distributed along an environmental gradient. Each lineage may respond differently to climate change, and modelling them together implies assuming a single niche for all of them. Additionally, this leads to an overestimation of the number of species modelled, since the actual number is lower due to several species being grouped as one. This is particularly relevant because, in principle, the inclusion of many species is presented as one of the strengths of the manuscript. I consider this approach flawed, and the authors should be fully aware of the limitations of modelling using MUs.

- The excel spreadsheet in the supplementary material (Sup. Mat. 2) clearly outlines which species were included in which MU and our detailed methodology in the supplementary material (Sup. Mat. 1) includes maps showing examples of how distributions, data points and background points were treated for representative example MUs. In this way we are fully transparent as to which species have been combined and how. We only combined species into MUs where it was absolutely necessary and for one of two reasons. firstly, where individual species had so few data points that we could not model them individually, and another, closely related and ecologically similar species with more data was available to combine it with. An example of this would be *Bothriechis marchi*, *B. thalassinus*, and *B. guifarroi,* 3 closely related, Central American pitvipers with adjoining distributions and limited available occurrence data (Fig SM2.3). Second, some species were modelled together because there are ongoing taxonomic revisions in progress and the “true” geographic delineation of the species is not known and estimates are constantly changing. An example of this are *Trimeresurus albolabris*, *T. guoi*, and the varying *T. aff. albolabris* species that have been genetically separated and rejoined over the last few years. We are aware of the reservations with this approach and specifically address these the methods section (L192-198:“Where our minimum ‘standard’ of 20 unique records at 0.01 x 0.01 decimal degree resolution could not be reached, or where taxonomic or geographic delineation between closely related and ecologically similar species was poorly resolved, we ran multi-species models for ‘modelling units’ (MUs). These 314 MUs combine species only when necessary for modelling purposes, i.e. if they are data deficient or too difficult to delineate from each other due to insufficient taxonomic certainty in many areas, and they expected to be similar in their habitat requirements based on existing knowledge of their biology.“) and in we have added a section to the discussion acknowledging the limitations of our approach (L653-647: “Generally, models for MUs performed well, indicating that the way species were combined into multi-species models if necessary did not result in poorer models. However, we acknowledge that using MUs likely estimate a larger umbrella niche for species complexes than individual species models would and recommend that separate models are run as soon as sufficient data point become available and geographic delineation between lineages are reliably resolved.“)

Along these lines, the authors give the example of the inclusion of the five species formerly classified as Naja melanoleuca. In this case, it is unclear whether they were modelled as a complex or separately. If modeed as a complex, it would be similar to modeling a single species (as in previous studies), and if modeled separately, I have some questions. Reviewing Wüster et al. (2018), which describes these five species, it is stated, for example, that Naja melanoleuca and Naja subfulva occur sympatrically in some areas and are difficult to distinguish morphologically:
"Some of these specimens and populations are difficult to differentiate from sympatric N. melanoleuca, necessitating the use of DNA barcoding approaches (Hebert et al., 2003; Clause et al., 2016) to ensure reliable identification."
How did the authors distinguish occurrence points in those locations, especially when using GBIF data? Furthermore, one of the species, Naja peroescobari, is found on an island—how was this species modeled?

Regarding the specific example of the *Naja melanoleuca* complex, we modelled most of the species in the complex separately. The only ones we combined were *N. peroescobari* and *N. melanoleuca* sensu stricto (see table in Sup. Mat. 2). Regarding how we attributed pre-species-split records, we attributed all records up to 2018 that were clearly only within one of the different species’ distributions according to Wüster et al. 2018 to that species. All *N. melanoleuca* records in areas where the different species overlap were excluded from the dataset. The only exception was where the species description of the record clearly stated the subspecies, e.g. *N. melanoleuca subfulva*. We also obtained all species records from Wüster et al. 2018. All records from 2019 onwards were considered as valid because they would have used the updated taxonomy. The resulting dataset was shared with Wolfgang Wüster for comment and clarification. Any records that were dubious according to him were excluded. This is also why expert opinion was crucial for data vetting. Conferring with taxonomist who published species’ most recent taxonomies ensured that we did not attributed existing records incorrectly.

Another relevant issue is the excessive reliance on expert opinion throughout the manuscript. Ideally, information used in a scientific paper should come from published sources, not primarily from expert panels. Some examples include:

The first drafts of the snake expert-derived ranges were entirely based manuscripts and databases in the public domain. The expert panel was used as an additional layer of peer review to clean and vet the data and the distributions afterwards. All environmental niche models are based on occurrence data that needs cleaning but in most cases this is simply done by the authors. Sending our data out to experts for additional cleaning was a more rigorous process than what is commonly done. The list of experts consulted, in addition to those who are co-authors, is given in the acknowledgements. The habitat suitability models were based on the occurrence data, so they are statistical models, not expert opinion. We believe that comparing the expert derived ranges with the statistical models is a strength of our manuscript rather than a weakness because it enables us to see where either may need further information, and we discuss throughout the manuscript how the two relate and where they indicate data gaps that need targeted work. Further details to specific points are outlined below.

Line 140: “Data collation included rigorous vetting by an international expert panel comprised of leading researchers in the fields of snake distributions and taxonomy to guarantee a trustworthy one-stop-shop for researchers, governments, NGOs, and the general public.”

This is not ‘expert opinion’ but using experts to clean and validate data, often their own data, to clarify if any errors had been introduced by our data collation process or if we misrepresented their findings in any way.

Line 178: “All occurrence records were vetted for taxonomic and location accuracy by an expert panel of >30 experts from around the world.”

Again, experts helped us to clarify taxonomy, identify dubious records for in depth checking, and provide additional evidence of occurrence records in under-surveyed regions.

Line 215: “Only two sets of collinear variables were maintained based on expert experience of complementarity of those variables in other analyses despite collinearity.” This is problematic—despite acknowledging serious collinearity issues, the authors retained collinear variables based solely on expert opinion.

Maxent has been shown to deal very well with colinear variables and we do not see an issue with including some collinear variables that may add important information. Maxent will simply disregard the variable that performs more poorly and only retain one of the colinear variables in the final models after variable selection. The variable selection process determines whether the two variables both add sufficient new information to the model and therefore accounts for any duplicity in the starting variables. When the issue was first brought to our attention in the reviewer’s previous assessment of our manuscript (during review at Nature Communications) we added information to the text of the manuscript to clarify this in L227-230: “Collinearity has been shown to be of minor importance to Maxent model performance [31], and our variable selection process eliminates any redundancy in final model predictors. Therefore, including some collinear variables was considered preferably to excluding potentially biological important variables.”

Line 403: “Underpredictions were common where anecdotal expert knowledge suggests occasional presences without accurate location information.”

This line explains that there are areas where some experts believe the species may be present but where no actual occurrence records exist, as an example where our study could potentially identify knowledge gaps for medically important snake specie that have been only poorly surveyed to date. These areas were NOT included in the expert derived ranges because of lack of data. The fact that our models show some habitat suitability in these areas support the hypothesis that the snakes may be present but more sampling in these areas is potentially warranted.

Line 483: “We established a panel of experts to vet all data and outputs, lending additional reliability to our results.”

We asked experts for feedback on potential problems with any of our data and analyses as an additional peer review step before submission of the manuscript to your journal.

My next major concern involves the construction of the species distribution models. The implementation lacks sufficient detail to assess its rigor. Specific concerns include:

We would like to refer the reviewer to the detailed methodology in Sup. Mat. 1, which answers many of their questions. Specific points are answered below.

(i) The authors do not specify how many background points were used per species.

We have added the number of background points, along with already included numbers of species and MU points used to the spreadsheet in the Sup. Mat. 2. to improve transparency

(ii) It is unclear how the test dataset was selected—I assume a random 30% split. If so, this is suboptimal due to strong spatial autocorrelation. A geographically structured cross-validation approach (e.g., Muscarella et al., 2014; <https://doi.org/10.1111/2041-210X.12261>) should be used to reduce spatial bias.

Our methods specify that “Maxent was run with 10-fold cross validation using 70% of subsampled training and 30% test data” (L279-280). We also specify in the detailed methodology in the supplementary information that “Only unique occurrence locations per 0.01 x 0.01 decimal degree grid cell were used in model”, i.e. records were thinned to 1 per 1x1km grid cell, which reduces spatial autocorrelation. The evidence for impact of spatial autocorrelation on environmental niche models is ambiguous in the literature and thinning occurrence data further would likely be more detrimental to model performance in cases of data sparse species than the presence of some residual autocorrelation. (e.g. <https://doi.org/10.1016/j.ecolmodel.2016.02.021>). In some similar cases “spatial and environmental thinning led to a general decrease in model performances across all SDM methods” (<https://doi.org/10.1002/ecs2.4703>)

(iii) The authors should filter out points with high climatic autocorrelation.

See our point above, explaining how environmental thinning similar to spatial thinning, does not necessarily improve models, especially for data deficient species.

(iv) It is not clear how the authors selected among linear, quadratic, product, or hinge feature types. Was it based on AIC? AUC?

We specify in the detailed methodology in Sup. Mat. 1 that ”We allowed for linear, quadratic, product, and hinge features except for species that were both, data sparse (<80 records) and restricted (background buffer <1000km). For these, no product features were allowed to avoid overfitting”. Beyond this, we allowed Maxent to choose which features were appropriate as this is an inbuilt function in Maxent.

(v) The rm hyperparameter in Maxent should be evaluated.

We disagree with the reviewer and agree with Merrow et al 2013 (A practical guide to MaxEnt for modelling species’ distributions: what it does, and why inputs and settings matter) that “the default regularization values have been chosen based on performance across a range of taxonomic groups (Phillips and Dudik 2008), and may be useful when building models for many species simultaneously, when species-specific tuning is impractical.” Also, our complete database is already nearly 2Tb in size, beyond the scale that public repositories such as Dryad can accept. Evaluation of the rm parameter is computationally greedy, which would increase this large number even further, and for little benefit. Rm evaluation may be of utility in some contexts but is not practical for large multi-species datasets such as ours.

(vi) Variable elimination was based on permutation importance, but if there were only 20 presence points and 10,000 background points (exact numbers are not given), removing 15 predictors does not produce a balanced model.

Exact numbers of background points are given in the spreadsheet in Sup. Mat. 2. The number of background points varies depending on the species because the raw target background was cut back to an area proportional to the species range size, i.e. restricted species had a smaller background than wide-ranging species.

(vii) Some variables were kept static even though they are expected to change in the future. How does this impact projections? Would using only climatic variables yield different results? A sensitivity analysis is needed.

Variables such as vegetation, land use, and human population density will likely change in the future, but these will need to be subjects for future investigations and are beyond the scope of this already substantive manuscript, which focuses on assessing the effects of climate change specifically. We added a section to the discussion addressing this limitation in L738-741: “Future anthropogenic land use change and climate change driven vegetation changes will likely further impact snake distributions, while human population growth may increase SHOI further. While we only examine the impact of climate change in our study, these additional drivers of SHOI should be examined further in future studies”

(viii) Line 306: How was the cost of 500 determined? Based on what criteria? Many decisions seem arbitrary and lack clear justification.

The value of 500 is a very lenient threshold that mostly excludes completely disconnected habitat patches that are not known to be occupied and areas of low suitability far from known locations. We provide the full, uncut model outputs along with the cut ones, as well as the R code used for cutting so users can replicate our results with a threshold that suits them or use the full models. The fact that most of our models predict low suitability habitat beyond the expert derived ranges shows that the value of 500 is very lenient and only excludes habitat that is highly unlikely to be occupied, and we discuss how EDRs relate to cost distance restricted ENMs in our manuscript to clarify any impacts of uncertainties in this methodology. The value was not changed between current and future predictions, so it does not impact the comparisons of current to future distributions. The only impact that our choice of value potentially has is that it might still overestimate species outer distribution limits slightly, which is preferrable to overestimating distributions to areas that are simply unreachable.

(ix) More than one climate scenario should be included—standard practice involves using at least three (RCP2.6, RCP4.5, RCP8.5).

We explained our rationale for using only one scenario in our manuscript in L244-252: “We only used one future pathway, often considered the ‘business as usual’ pathway, because of computational limitations projecting over 300 MUs to two future timesteps at our comparatively high resolution of 0.01 decimal degrees (~1km) across seven GCMs. Using SSP5-8.5 allows us to plan for the likely worst-case scenario. Real changes may be less severe in the long term (2090) if climate change prevention strategies are ultimately adapted more widely, but ‘real’ effects are likely to lie somewhere between current conditions and SP5-8.5 in the shorter term (2050). For example, Capellán-Pérez et al. estimate that there is a 92% chance we will surpass total radiative forcing considered in pathway RCP-4.5 (most similar to current pathway SSPS-4.5), one of the most commonly used ‘intermediate’ emission scenarios [35].”

(x) Why was Maxent selected? While I do not object to its use, a stronger justification is required. Why not use ensemble models?

We explained this in our manuscript in L254-260: “Habitat suitability of each MU was modelled using Maxent 3.4.4 [36]. Maxent has frequently been shown to outperform many other approaches to modelling habitat suitability [37]. Some studies have recently used ensemble approaches to estimate the uncertainty involved in the selection of modelling method; however, these approaches may also artificially introduce uncertainty by including less reliable methods [38, 39]. Given the enormous computational requirements involved in modelling over 300 MUs and projecting them to 2 future time steps under 7 GCMs each at a very high resolution of ~1km, we limited our models to one method that is known to perform well.”

My final methodological concern relates to the use of human population density. While its inclusion is conceptually interesting, the authors fail to distinguish which segments of the population are actually at risk (e.g., rural workers, rural populations). Large metropolitan areas may have high habitat suitability for snakes and high human density, but still experience low incidence of snakebite. Moreover, the study assumes a static human population density for future projections, which is unrealistic. Future human density models should be incorporated into the projections.

Our study aims to estimate the overlap of humans and snakes as a proxy for the baseline potential of human-snake conflict. We do not attempt to quantify actual snakebite risk as discussed in depth in L623-633. We decided against changing human population density in the future because we wanted to specifically examine the effects of climate change and related shifts in species’ distributions. Adding other drivers of SHOI was beyond the scope if this already expansive study.

Lines 331–334: The study by Martin et al. is well conducted and finds an association between estimated abundance and incidence. However, their approach is based on Point Process Models, which differ significantly from the method used in this article. In contrast, the work by Yañez-Arenas et al. (2016) does find an association between the sum of suitabilities (binary maps) and snakebite incidents, although only in certain countries. Therefore, I believe it would be beneficial to incorporate an analysis that can more robustly support the validity of that claim. Once again, including only Type I species in this estimate could be helpful.

We did not aim to correlate snake habitat suitability with risk in our manuscript but rather correlate it with snake abundance. Maxent habitat suitability has been shown to correlate with abundance in multiple studies (see for example VanDerWal et al. 2015: Abundance and the environmental niche: environmental suitability estimated from niche models predicts the upper limit of local abundance). Snakebite risk, however, is thought to be a function of the contact rate between humans and snakes through the law of mass action (see e.g. Bravo-Vega et al. 2023: A generalized framework for estimating snakebite underreporting using statistical models: A study in Colombia) and our outputs are consequently a valid basis for future studies to calculate snakebite risk based on human and snake abundance proxies and other additionally factors that potentially increase conflict.

Overall, the results are presented appropriately, and the figures are of good quality.

The authors should avoid including references in the results section; this section should be rewritten to prevent mixing results with the discussion.

We have removed the text including 3 references in the results and limited their mention to the discussion.

One concern is that many of the conclusions are not new. For example, the main conclusion in the abstract states: “We predict substantial, short- and long-term shifts in snake distributions, including range contractions for many threatened species and increased human exposure to species of major public health concern.” However, upon reviewing Martines et al. (2024), their abstract presents a very similar conclusion: “Losses of potentially suitable areas for the survival of most venomous snake species will occur by 2070. However, some species of high risk to public health could gain climatically suitable areas for habitation.”

This overlap is also evident in the discussion, where the authors highlight key snake species responsible for envenomations (e.g., Bungarus caeruleus, Daboia russelii, Naja naja, Echis carinatus, Crotalus durissus, Bothrops atrox, Bothrops asper, Naja nigricollis, Bitis arietans, among others), most of which were already included in Martines et al. (2024). Similarly, the conclusion that snake distributions will contract in the Congo and Amazon rainforests is also consistent with the findings of Martines et al. (2024).

I believe the manuscript would benefit greatly if the authors clearly emphasize what the inclusion of new data contributes beyond what has already been demonstrated in previous studies.

Our study is the first to provide distribution estimates and habitat suitability estimates for every single medically important snake species. It does so at a higher resolution than any previous studies, based on the biggest venomous snake occurrence dataset collated to date. We provide the first detailed estimates of how all these snake species overlap with people, and include lists of ranked species for every country in the supplementary material, giving countries tools to decide on antivenom usage and, based on our maps, antivenom placement for the correct species. It comes with a large dataset of distributions for each individual snake species. We believe this is a significant contribution to the literature and an unprecedented tool for other researchers to use. We believe that agreement of our predicted trends with the lower resolution predictions of previous studies for species subsets is a positive outcome because it confirms that a more complete analysis including all relevant species verifies the trends and shows that these predictions are robust and reliable. We have updated the abstract to clarify these advances.

One possible improvement would be to incorporate the adaptive capacity of species to climate change, as demonstrated by Diniz-Filho et al. (2019) (<https://doi.org/10.1111/ecog.04264>) and Souza et al. (2023) (10.3389/fevo.2023.1038018). This addition would strengthen the manuscript by providing a more accurate assessment of whether species projected to lose habitat will actually decline or potentially adapt to new conditions. Simply increasing the number of species included may not constitute sufficient novelty for a high-impact journal such as PNTD.

This is an excellent idea for future studies, that will take substantive funding, time and will benefit considerably from use of the data we have collated and present in the current manuscript. But it is a substantially different study beyond the scope of what we are able to describe here.

The manuscript titled "Climate change induced complex shifts in snake distributions expose people to snakebite and threaten biodiversity" addresses an important topic: the effect of climate change on the distribution of venomous snake species. I had previously reviewed this manuscript for another journal, and I am sincerely disappointed that the authors have not incorporated any of the suggestions I made during that earlier review. Therefore, my current evaluation remains the same as before.

We were very grateful for the suggestions from the review of a previous version of the MS and did indeed make a number of changes in the text, and also in the supplementary material that the reviewer appears, unfortunately, not to have reread. Some changes, as articulated above, were not possible because the MS, with associated data is already extremely large and suggestions for further studies cold not be incorporated without making the datasets too unwieldy

The paper deals with an interesting subject: the effect of climate change on the distribution of snake species that pose a risk to humans. The strongest aspect of the study is the inclusion of a larger number of species compared to previous studies. However, from a methodological perspective, the work presents some limitations, and its conclusions are constrained.

We thank the reviewer for the feedback.

**Reviewer #2:**

The paper is very well written and meets the review criteria needed for publication.

The results are presented clearly and are based on the analysis that was conducted. The figures are of high quality and enable easy interpretation of the data presented within the results section.

The conclusions drawn from the data are clearly thought through. Potential limitations are discussed and incorporated into the explanation while addressing the potential impact of the work from a public health perspective.

I have no suggestions for data modifications.

I felt this paper was very well written and I have no concerns to raise after reading through it. The work presented here could be used to help guide stakeholders involved in the prevention of snakebite (as discussed).

We thank the reviewer for the positive feedback.

**Reviewer #3:**

For ease of understanding, we have separated the reviewer’s response into two sections: “Positive feedback”, which does not need to be addressed, and “Concerns”, which we address in detail below:

**Positive feedback:**

The manuscript articulates clear objectives, namely to improve global understanding of the distribution of medically important venomous snakes (MIVS) and to model the overlap between snake distributions and human populations under climate change. The hypothesis — that climate change will shift snake ranges and alter human exposure — is well grounded and testable.

The study design is appropriate and innovative, utilizing expert-derived ranges (EDRs), extensive occurrence records, and environmental niche models (ENMs) at high spatial resolution (~1 km). The iterative process for taxonomy validation and model vetting adds rigor and credibility.

Key strengths:
- Extensive dataset covering 508 MIVS and 314 modeling units (MUs).
- Expert vetting of occurrence data and spatial layers.
- Use of robust modeling techniques (Maxent, cost distance thresholds)
- Application of a "One Health" lens.

The results are thoroughly presented and supported by high-quality figures (e.g., maps of current and future species distributions, Snake-Human Overlap Index). The results align with the methods and hypotheses and present a convincing case for shifts in both biodiversity and human exposure.

Noteworthy aspects incude:
- Over 90% of ENMs had AUC > 0.85, indicating strong predictive performance.
- Clear documentation of which species are expected to see range contractions vs. expansions.
- Stratification of results by biogeographic region adds policy relevance.
- Detailed analysis of agreement/disagreement between ENMs and EDRs demonstrates transparency and robustness.

The conclusions are strongly supported by the data and analysis. The authors correctly highlight the dual implications for public health and conservation. They do not overstate their findings and are appropriately cautious regarding uncertainties.

Highlights include:

- Emphasis on the dynamic and updating nature of the OPHIDS database is timely and commendable.
- Appropriate call for region-specific One Health interventions.
- Clear articulation of how this work supports WHO's 2030 snakebite goals.

The study uses secondary, non-human data, meaning there are no ethical concerns regarding subject use or data acquisition.

Strengths:
- Unprecedented global scope and resolution for MIVS modeling.
- Integration of updated taxonomy and vetted occurrence data.
- Methodologically robust ENMs and well-articulated analytical pipeline.
- High policy relevance for climate adaptation, conservation, and snakebite management.

Novelty and Significance:
- Highly original contribution with direct implications for WHO targets.
- Sets a new benchmark for predictive modelling in the NTD and biodiversity domains.

We thank the reviewer for the positive feedback.

**Concerns:**

While the choice of Maxent is justified due to computational limits, lack of ensemble modeling may affect robustness in areas of ecological uncertainty. The authors should acknowledge this trade-off more clearly.

Provide a justification for the lack of ensemble modelling or include a brief sensitivity analysis where feasible.

While we do mention in the detailed methodology in Sup. Mat. 1 that ensemble models were outside of the scope of our study, we appreciate that this would be a great avenue for the future and have added text to our discussion L762-764: “Furthermore, as high computing power becomes more accessible, we encourage further research into snake distributions using a broader variety of model approaches (e.g. ensemble models), and other approaches that are computationally demanding and were outside of the scope of our study.”

Inclusion of only one SSP pathway (SSP5-8.5) for climate change limits policy utility; a short justification is offered, but considering recent shifts in emissions policy, it would be beneficial to clarify limitations further.

Reliance on a single climate scenario limits forward-looking adaptability.

Discuss limitations of projecting only SSP5-8.5 in more detail and its implications for planning.

We explained our rationale for using only one scenario in our manuscript in L244-252: “We only used one future pathway, often considered the ‘business as usual’ pathway, because of computational limitations projecting over 300 MUs to two future timesteps at our comparatively high resolution of 0.01 decimal degrees (~1km) across seven GCMs. Using SSP5-8.5 allows us to plan for the likely worst-case scenario. Real changes may be less severe in the long term (2090) if climate change prevention strategies are ultimately adapted more widely, but ‘real’ effects are likely to lie somewhere between current conditions and SP5-8.5 in the shorter term (2050). For example, Capellán-Pérez et al. estimate that there is a 92% chance we will surpass total radiative forcing considered in pathway RCP-4.5 (most similar to current pathway SSPS-4.5), one of the most commonly used ‘intermediate’ emission scenarios [35].”

We would like to add other scenarios, but it is simply not feasible considering computational limits at this time.

The cutoff of <20 occurrence records per species/MU for full modeling is reasonable but could be supplemented by uncertainty metrics or maps to explicitly convey model confidence.

We discuss model performance for data deficient MUs in L634-657 and provide a figure comparing model performance to number of records used (Fig. 1), which shows that performance was good even for data poor taxa. All incidence of sub-optimal performance for any MUs are discussed in the manuscript and we encourage readers to be aware of the limitations of those few models.

Better distinguish between "exposure risk" (overlap) and actual bite risk, which includes behavioral and cultural factors.

Human behavioral variability (e.g., use of protective equipment, awareness) not integrated into SHOI or modelling.

We agree that human behaviour and snake traits are crucial to snakebite risk, and we discuss this issue throughout the manuscript. However, quantifying SHOI in itself is a major task, and all other snake and human behaviours can only cause snakebite risk if snakes and humans co-occur to start with. Therefore, we decided to start with SHOI and then conduct further research to investigate how exactly it translates into snakebite risk, and which factors modulate it. For that, much more data is needed, including incidence data to ground truth risk predictions. We added a sentence into the discussion encouraging researchers to build on SHOI by including such modulating drivers of snakebite risk L757-764: “We recommend future studies to further build on the dataset we collated, to enable individual models for some of the more data deficient species, and to fill survey gaps and verify our predictions in poorly sampled regions. We hope our data collection and models will present an opportunity for the broader research community to combine efforts and improve our understanding of the spatial distribution of snakebite risk, by incorporating drivers of snake-human conflict in areas of co-occurrence.” We also clarified this in the abstract L70-74: “In combination with other drivers of increased snake-human conflict, such as human behaviours and snake traits, our predictions can be used to decide where to stockpile which antivenom, how to ensure adequate capacity of individual health facilities, how to improve health care accessibility of remote at-risk communities, and where to focus conservation efforts for threatened snake species.”

Improve clarity of the abstract. Currently too dense and jargon-heavy; streamline and define SHOI.

Improve abstract readability and figure clarity.

Figures are high quality but sometimes dense; perhaps include regional insets or simplify overlays for readability.

Expand legends and titles in some figures to clarify differences between present and future projections.

We have revised the abstract accordingly. We also revised the figure legends and aimed to user simpler language and longer descriptions. Regarding figures, we understand that they are information dense but believe all the information shown is too important to each figure to be removed. Some regional insets are already provided. We are open to more specific suggested changes for individual figures.

Assumes dispersal without addressing species-specific barriers in detail.

We would argue that there are two types of species-specific dispersal limitations: 1. Barriers that cannot be crossed, and 2. General dispersal ability in the sense of how far species can travel per day or year.

The first one is exactly what we aimed to address by restricting models to a certain cost-distance from known occurrences. Essentially the distribution model will predict low suitability for grid cells that act as barriers, such as lakes for terrestrial species, or mountains for lowland species. But even grid cells with low habitat suitability because of temperatures that are too high or vegetation types that are disfavoured will act as barriers. Our cost-distance approach gives such areas a high cost of travelling through them and, therefore, uses them as barriers to dispersal. The same barriers to current dispersal are also used for future dispersal, so species can only expand their range in a direction where suitability is better than it was before, i.e. where dispersal barriers have been reduced.

The second limitation is how far a species can travel in general, assuming no barriers, and this will vary among species. Unfortunately, we do not have a good understanding of species-specific dispersal rates for snakes. We do want to address this limitation in the future but it requires a lot of additional data collection and is outside the scope of this study at this point.

Add a concise user guide or example on how policymakers or local health agencies can use the database.

We have added a short user guide document to the data directory and files available in the database. Unfortunately, providing a detailed user guide, while being a valuable addition, is outside the scope of this manuscript at this point. We are working on a case study for one country that will show how the data can be applied and plan to use it as a blueprint for other countries. Unfortunately, this incorporates some additional, country-specific analyses and data and will, therefore, be a separate study.

A table summarizing species with highest projected increase in SHOI per region would enhance usability by health policymakers.

More explicitly state how governments or researchers can use these outputs — e.g., in hospital planning or antivenom supply chains.

We thank the reviewer for this constructive idea and have added figures of future SHOI and SHOI changes for each individual country to our supplementary material (Sup. Mat. 7) We’ve added test to the discussion L728-730: “Detailed SHOI change predictions for individual countries are provided in Sup. Mat. 7 and should be used by policy makers to guide long term decisions on antivenom procurement and placement.” We have also added text to the abstract L71-74: “…our predictions can be used to decide where to stockpile which antivenom, how to ensure adequate capacity of individual health facilities, how to improve health care accessibility of remote at-risk communities, and where to focus conservation efforts for threatened snake species.”

Minor typographical and grammatical inconsistencies throughout (e.g., spacing, abbreviations).

We have done our best to correct these errors but appreciate any additional input.

Consider including a glossary of terms (SHOI, ENM, MU) for broader accessibility.

We think this is a great idea and have added a glossary box of key terms after the author summary to improve accessibility.
