# Supplementary material for: Climate change induced complex shifts in snake distributions expose people to snakebite and threaten biodiversity
Source: PLoS Negl Trop Dis. 2026 May 21;20(5):e0014030. doi: 10.1371/journal.pntd.0014030 (PMC13193456; doi:10.1371/journal.pntd.0014030)
Supplement: Attachment — Submitted filename: Responses to reviewers.docx [file pntd.0014030.s011.docx]

We thank the reviewers for their feedback. Please find below our responses to each comment, highlighted in red.

Please also note that we have made minor editorial changes to the figures as requested by WHO to correctly update territorial boundaries on all maps.

Reviewer responses:

Reviewer 1:

First, I would like to thank the authors for addressing my questions and comments on the manuscript. Some of these concerns were resolved through the responses and modifications in the text. However, I believe that some methodological and writing issues still remain.

1- Regarding collinearity among variables.
The authors argue that collinearity does not affect the Maxent model (based on reference 31). This is true, but collinearity can affect the model’s projections in new environments. I provide a passage from the abstract of reference 31:

“… (c) collinearity shift and environmental novelty can negatively affect Maxent model transferability. We therefore recommend to quantify and report collinearity shift and environmental novelty to better infer model accuracy when models are spatially and/or temporally transferred.”

Following what the authors cite, I recommend showing whether collinearity shifts exist in the future. It is not necessary to do this for each species model, but at least for each global bioregion. Another valid option is to model without collinear variables.

If we were extrapolating from our models the reviewer would have a valid point. We explicitly state however, that we have run our models **without** extrapolation (L278) and so projection to novel environments is minimal. Therefore, we persist in our assessment that the benefits of including some collinear biologically relevant variables whenever this leads to improved model performance outweigh the risk of collinearity shifts.

2- Regarding the test dataset.
Having an independent test dataset is fundamental to evaluate model performance and determine whether the model can make accurate predictions. I consider that the authors should report the AUC (and at least one additional metric, such as TSS or KAPPA) for the test dataset of each model, and not only for the training dataset. Ideally, the test dataset should be partially independent of the training dataset, meaning it should not be just a random 30% of the records.

Our test data in each of the 10 model runs is a subset of 30% of the whole dataset. This subset is removed from the training data. As such, the test dataset is independent of the training data each time (L277-278). It is very rare in the literature to use completely geographically separate datasets for testing, especially so in large studies of many species. We already present test as well as training AUC in Fig.1, and our database in the Harvard Dataverse already includes full maxent results including test and training AUCs for each individual model run (<https://doi.org/10.7910/DVN/QVWB4E>). We also already present partial ROC as an additional metric, as suggested by Peterson et al. 2008 (“Rethinking receiver operating characteristic analysis applications in ecological niche modelling”).

3- Use of multiple climate scenarios.
This is practically mandatory in climate projection studies. The authors themselves note that climate change is likely to exceed the expectations of the RCP4.5 scenario; it is more likely to fall between RCP4.5 and RCP8.5. Therefore, I strongly suggest presenting models for both scenarios and using at least two uncorrelated Global Circulation Models, which is the minimum recommended in climate change studies. A valid alternative is to use 2.5 arcmin grid cells, which still allows for a clear representation of the margins of change in distributions between the two scenarios; more detailed information could be included in the supplementary material. For a global study, using 1 km resolution is not necessary. Today, there are cloud computing options that allow these analyses to be performed, so computational capacity does not seem to be a valid limitation.

One of the main goals of our study was to model snake distributions at a scale useful for health systems microplanning, which no other study has previously done (L62; L83-83; L106-110; L244; L257; L601; L671; among others). Modelling at 2.5 arcmin resolution would defeat this purpose. As it stands, we are already using high performance computing systems, taking up 100s of GB of working memory, and producing over 4TB of baseline, interim, and final data files. We similarly already struggled finding a data repository that would store our much reduced, essential resulting data files. We are looking at options to expand our usage and storage but at this point we are very much limited by computing power and have been told by several other researchers that they were surprised at the scope of what we were able to achieve computationally. We maintain that the RCP8.5 scenario is the most relevant in the context of future proofing mitigation strategies, as they need to meet current and worst-case future scenarios.

Citation of previous studies.
The authors state in their response:

“We believe that agreement of our predicted trends with the lower resolution predictions of previous studies for species subsets is a positive outcome because it confirms that a more complete analysis including all relevant species verifies the trends and shows that these predictions are robust and reliable.”

If previous studies have already reached similar conclusions, the authors should cite them and discuss how their results align with previous findings. As written, it gives the impression that this study is the first to reach these conclusions, which could be considered a careless acknowledgment of the existing literature, whereas referencing previous studies would strengthen the interpretation and relevance of the results.

We already discuss the mentioned recent publication on the topic repeatedly in our manuscript (L127-132; L569-573; L556-589). While some average global trends across species may be similar between the studies, our study provides a very different aim and context, focusing on detailed high resolution distribution maps of all individual relevant species that can be used for fine scale local, regional, as well as coarser scale planning depending on end-user needs. We do not just assess the global impact of the most wide-ranging species but fill important knowledge gaps on the complete set of species present in each country, based on large occurrence data sets far beyond what is available in online data bases.

Reviewer 3:

The study has a very clear goal: to map where 508 venomous snakes live now and where they might move in the future due to climate change. The authors also want to see how much these snakes will overlap with human populations (the Snake-Human Overlap Index, or SHOI).
The design is appropriate. They used a computer modeling program called Maxent to predict suitable habitats. Because some snake species are rare and have little data, the authors grouped them into "Modelling Units" (MUs). This was a smart and necessary step to make sure all species were included. To ensure accuracy, they checked their computer models against maps drawn by experts.
The population is clearly described: they included every single medically important snake species listed by the WHO (Category 1 and 2). They addressed previous concerns about Category 2 snakes effectively, noting that these snakes can still be very dangerous but are often just less studied.
A main limitation is that they only looked at one future climate scenario (SSP5-8.5, or "business as usual"). However, they explained that running multiple scenarios for over 300 groups of snakes would require too much computer power. They also assumed human populations stay in the same place (using 2020 data), which is a weakness, but understandable for the scope of this study. There are no ethical concerns since they used existing data.

The results follow the plan exactly. The authors present the data clearly, showing which snakes will see their homes shrink (contractions) and which will expand into new areas.
The analysis is honest. They show that most of their models worked very well (AUC score > 0.85), but they admit that models for very wide-ranging snakes or data-poor snakes were less perfect. This honesty makes the results more trustworthy.
The figures are high quality. Figure 5 is especially helpful because it uses color shading to show "uncertainty"—basically, how sure they are about the future predictions. Figure 7 gives a great quick view of the world, showing that snake-human overlap will likely increase in places like the USA, China, and India. As requested in previous reviews, they moved references out of the Results section to keep it clean.

The paper is well written. The authors added a "Glossary" to explain technical terms like "SHOI" and "MUs," which makes the paper much easier to read for non-experts.
They also created a public database called OPHIDS so other researchers can use their data. This is a great contribution to science. They added new graphs for individual countries in the supplementary material (Sup. Mat. 7), which is very helpful for local policymakers who need specific data for their region. No further major changes are needed.

The conclusions match the data. The authors predict that many snake species will shift their ranges toward the poles (away from the equator) to find cooler temperatures. They correctly identify that this will expose new human populations to snakes they haven't dealt with before.
The authors are careful not to overstate their findings. They explain that just because snakes and humans overlap (SHOI), it doesn't automatically guarantee more bites—behavior and healthcare access matter too.
They do a good job discussing the limitations, such as the fact that they grouped some species into "Modelling Units," which might hide small differences between closely related snakes. They strongly argue that this data is vital for public health, specifically for deciding where to store antivenoms in the future.

This is a landmark study. Unlike previous papers that only looked at a few snakes or specific regions, this study attempts to model all 508 medically important snakes globally. This fills a massive gap in our knowledge. The only weakness is the use of only one climate scenario ("business as usual") and static human population numbers. While this limits the precision of the 2090 predictions, the authors justified it well based on the massive computing power required to do more

We thank the reviewer for the positive comments.
